# Zingiberis Rhizoma Ethanol Extract Attenuates Recognition Memory Deficits and Neuroinflammation in a Mouse Model of Bilateral Common Carotid Artery Stenosis

**DOI:** 10.3390/molecules31203634

**Published:** 2026-10-12

**Authors:** Taeyoung Huh, Chiyeon Lim, Sehyun Lim, Kyung-Min Kim, Suin Cho

**Affiliations:** 1 Department of Korean Medicine, College of Korean Medicine, Dong-Eui University, Busan 47227, Republic of Korea; tyhuh11@gmail.com; 2 Center for Advanced Medical Computing and Analysis, Massachusetts General Hospital and Harvard Medical School, Boston, MA 02114, USA; clim8@mgh.harvard.edu; 3 School of Public Health, Far East University, Eumseong 27601, Republic of Korea; favor@kdu.ac.kr; 4 Department of Korean Medicine, School of Korean Medicine, Pusan National University, Yangsan 50612, Republic of Korea

**Keywords:** vascular dementia, bilateral common carotid artery stenosis, Zingiberis Rhizoma, neuroinflammation

## Abstract

Chronic cerebral hypoperfusion (CCH), a key contributor to vascular dementia (VaD), causes white matter injury and neuroinflammation. We evaluated the neuroprotective effects of 6-gingerol-rich Zingiberis Rhizoma ethanol extract (ZRex) in a mouse bilateral common carotid artery stenosis (BCAS) model. BCAS-operated C57BL/6 mice received oral ZRex (30, 100, or 300 mg/kg, three times weekly). Spatial working memory, recognition memory, glial activation, oligodendrocyte and myelin markers, RNA sequencing (RNA-seq), and 6-gingerol molecular docking were evaluated. ZRex partially restored recognition memory in the novel object recognition test without altering Y-maze spatial working memory. BCAS significantly increased platelet-derived growth factor receptor alpha-positive cell counts, which ZRex restored toward sham levels. ZRex also markedly attenuated cluster of differentiation 68 immunoreactivity. RNA-seq identified 479 BCAS-responsive candidate genes (fold-change-filtered) and demonstrated that ZRex selectively restored downregulated transcripts. Molecular docking revealed the strongest predicted binding of 6-gingerol to Mup19 (Vina score: −9.4 kcal/mol), followed by Serpina3, Saa2, Mup1, and F10. Overall, ZRex attenuated BCAS-induced recognition memory deficits and neuroinflammation by modulating metabolic and inflammatory pathways, potentially mediated by direct 6-gingerol interactions with candidate targets. These findings support ZRex as a promising botanical candidate for VaD.

## 1. Introduction

Vascular dementia (VaD), the second most common form of dementia after Alzheimer’s disease (AD), is characterized by progressive cognitive decline associated with cerebrovascular insufficiency. Chronic cerebral hypoperfusion (CCH) disrupts neuronal homeostasis and contributes to white matter lesions, glial activation, blood–brain barrier disruption, and synaptic dysfunction [1,2]. The bilateral common carotid artery stenosis (BCAS) model is widely used in rodents to reproduce the CCH associated with human small vessel disease. In this model, microcoils or ligatures placed around both common carotid arteries induce sustained hypoperfusion, particularly in the hippocampus and white matter tracts. During the subsequent weeks, animals subjected to BCAS develop myelin rarefaction, astrocytosis, microglial activation, neuronal loss, and deficits in spatial working memory and executive function [1,2,3,4].

Ginger (*Zingiber officinale* Roscoe) has long been used in culinary and medicinal traditions and contains bioactive gingerols, shogaols, and paradols with potent antioxidant and anti-inflammatory properties [5,6]. Dried ginger (Zingiberis Rhizoma, ZR) is a cornerstone of East Asian medicine. Classical texts describe it as a pungent, warming herb that harmonizes the middle burner, dispels cold, restores *Yang energy*, and unblocks meridians [7,8]. ZR is traditionally included in formulas such as *Li Zhong-Wan* and *Si Ni-Tang* to treat gastrointestinal disorders, *Yang deficiency*, and *fluid retention*. Modern evidence increasingly supports the biological activities underlying these uses. In rodents, 6-shogaol attenuates scopolamine-induced memory deficits and amyloid pathology [9], whereas clinical trials have reported improved working memory and attention in adults receiving ginger supplementation [10]. Experimental studies further indicate that ginger extract reduces amyloid-β toxicity, oxidative stress, and proinflammatory cytokine expression in models of AD and ischemia [11,12]. Ethanol extraction notably enriches lipophilic gingerols and shogaols, thereby enhancing blood–brain barrier penetration and neural bioavailability. Despite this evidence, determining the optimal dose remains essential for translation because botanical extracts commonly exhibit bell-shaped dose–response relationships [13,14].

We hypothesized that ZR ethanol extract (ZRex), enriched in bioactive gingerols, protects against CCH-induced VaD by concurrently suppressing neuroinflammation and normalizing systemic and cerebral metabolic signaling. We therefore pursued three aims: (1) to determine the dose-dependent effects of ZRex at 30, 100, and 300 mg/kg on deficits in spatial working memory and recognition memory in the BCAS mouse model; (2) to assess whether ZRex attenuates BCAS-induced microglial and astrocytic activation and preserves white matter and oligodendrocyte integrity; and (3) to characterize the transcriptomic changes and active compound–target interactions associated with ZRex-mediated cerebrovascular protection using RNA sequencing, protein–protein interaction (PPI) networks, and molecular docking of 6-gingerol.

## 2. Results

### 2.1. Network Pharmacology and Target Prediction Analysis

**Figure 1:**
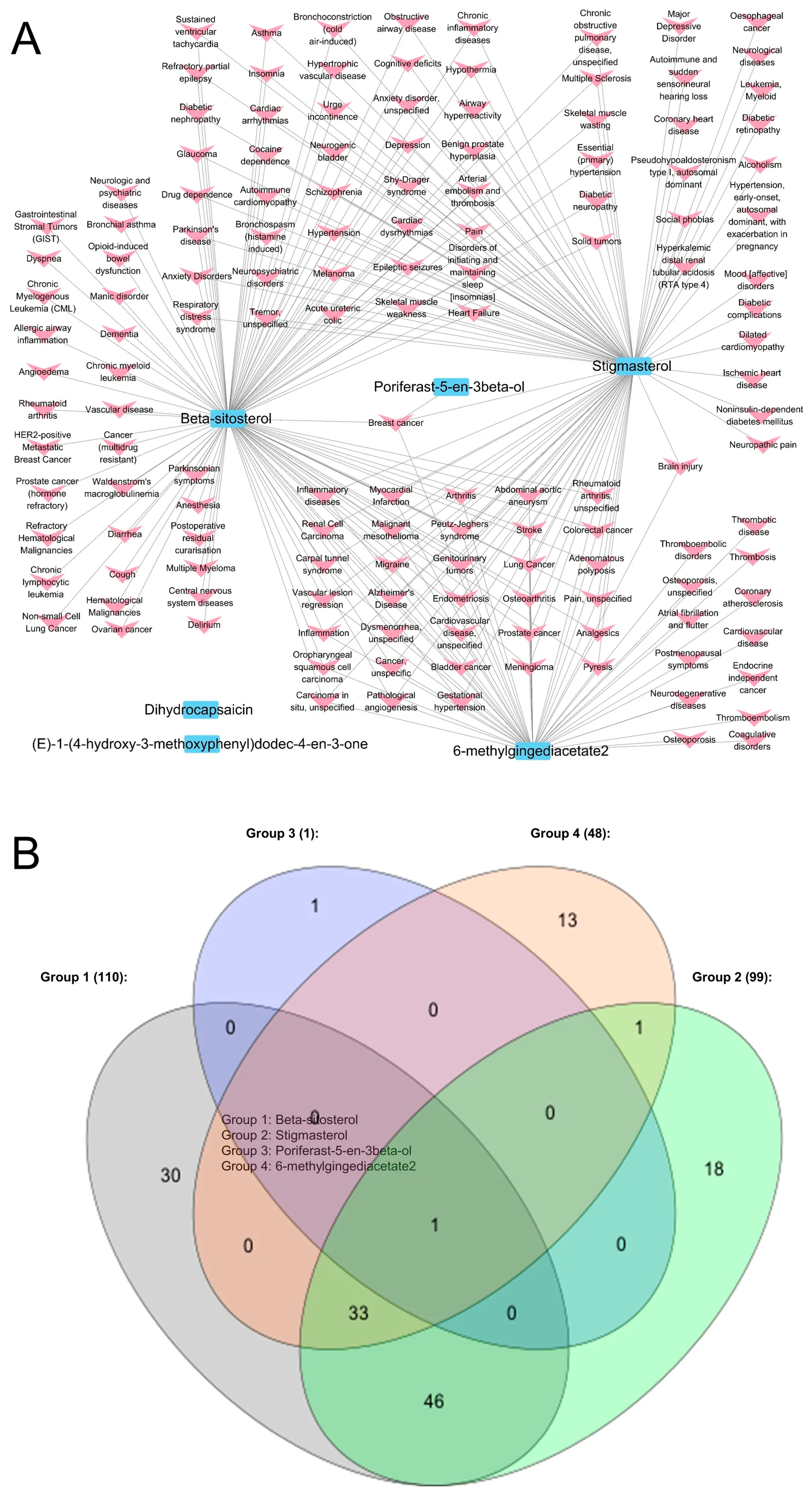
Network pharmacology analysis of active compounds in Zingiberis Rhizoma (ZR). (**A**) Compound-disease interaction network based on Traditional Chinese Medicine Systems Pharmacology Database and Analysis Platform (TCMSP) predictions for compounds selected according to ADME and physicochemical criteria. Blue rounded rectangles represent candidate bioactive compounds in ZR, and red V-shaped nodes represent predicted target diseases. Highly connected compounds are linked to a broad spectrum of diseases. (**B**) Four-set Venn diagram showing the unique and shared predicted disease associations among compound clusters derived from the TCMSP analysis. Group labels indicate the representative compounds assigned to each cluster.

**Figure 2:**
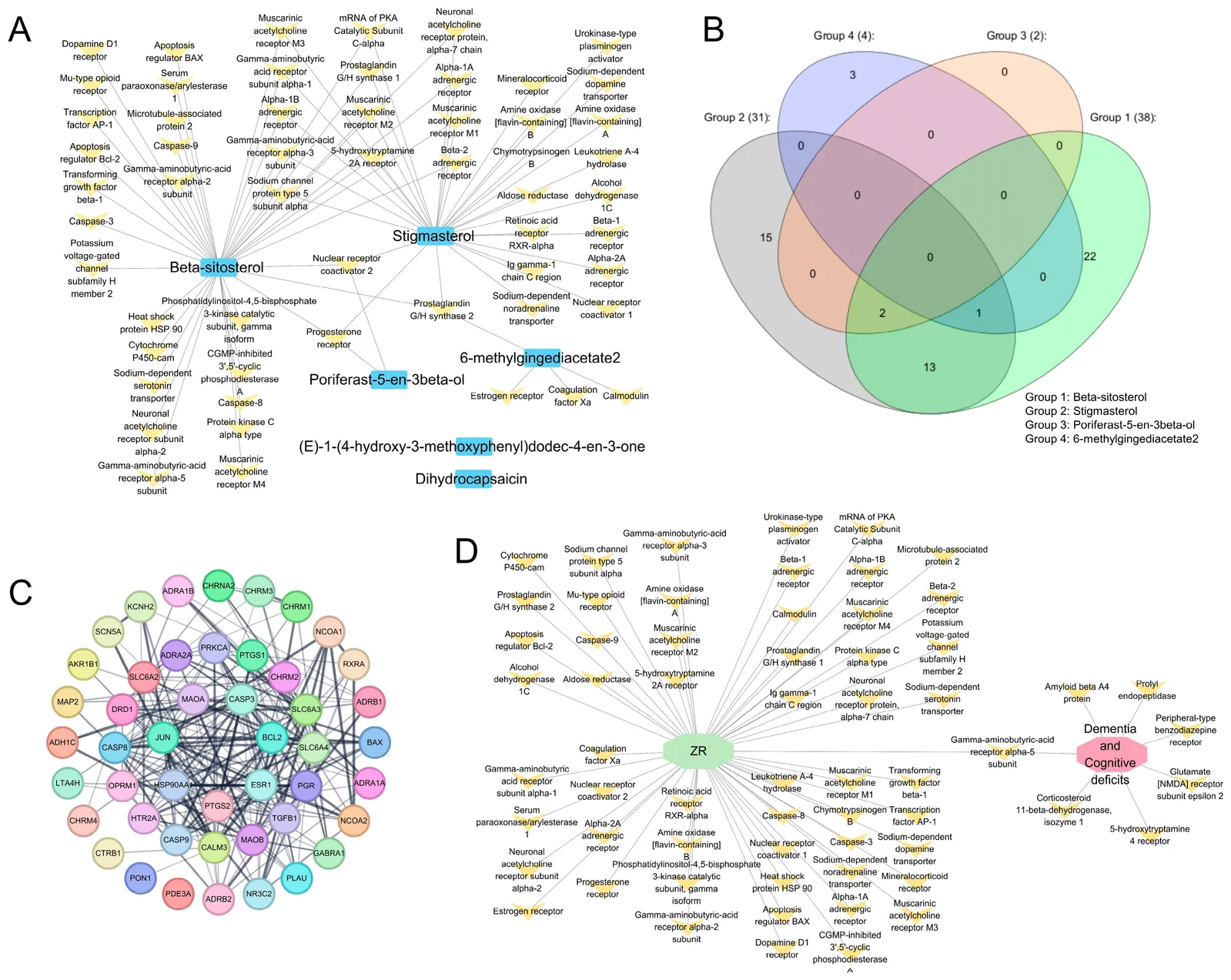
Compound–target networks generated from the TCMSP analysis. (**A**) Bipartite network showing interactions between candidate bioactive compounds in ZR (blue rounded rectangles) and predicted protein targets (dark-yellow V-shaped nodes). (**B**) Venn diagram showing target overlap among representative groups of ZR compounds. (**C**) STRING-derived protein–protein interaction (PPI) network arranged concentrically. Inner nodes represent highly connected hub proteins, including Ptgs2, Esr1, and Bcl2, whereas outer nodes represent targets with fewer interactions. (**D**) Comparative mapping of ZR compound targets and TCMSP dementia or cognitive-deficit targets. The limited direct overlap suggests that ZR may act through indirect regulatory mechanisms.

Network pharmacology screening identified six active compounds in ZR, including beta-sitosterol and stigmasterol, on the basis of molecular weight, oral bioavailability (OB), Caco-2 permeability, and drug-likeness (DL) (Table 1). We compared the predicted compound targets with disease-associated targets and identified overlapping elements (Figure 1A), which are summarized in a Venn diagram (Figure 1B). Group 1 had the largest unique set (30 elements), followed by Group 2 (18) and Group 4 (13). To investigate potential pharmacological mechanisms, we constructed a compound–target network (Figure 2A) and used a Venn diagram to compare the predicted targets of four representative compounds (Figure 2B). Beta-sitosterol and stigmasterol emerged as key bioactive constituents with potential therapeutic relevance.

PPI analysis using STRING identified central nodes in the target network (Figure 2C). Ptgs2, Esr1, and Bcl2 showed high connectivity, suggesting central roles in the pharmacological activity of ZR. These highly connected nodes occupied central positions in the network, whereas less connected nodes were located at the periphery. This arrangement illustrated the hierarchical influence of the predicted molecular targets.

We compared the targets of ZR compounds derived from the Traditional Chinese Medicine Systems Pharmacology Database and Analysis Platform (TCMSP) with dementia-associated targets and identified only one shared node (Figure 2D). The ZR-associated nodes formed a distinct cluster with minimal overlap with the dementia-associated targets, indicating limited direct convergence. Therefore, the potential effects of ZR on cognitive disorders may involve indirect pathways or mechanisms that are underrepresented in TCMSP. This finding underscores the need for experimental validation and complementary target-mapping approaches.

### 2.2. Physiological Measurements

Plasma electrolyte measurements indicated systemic stability across the groups. Mean sodium (Na^+^), potassium (K^+^), and chloride (Cl^−^) concentrations remained within physiological ranges and did not differ significantly among the sham, BCAS, and ZRex-treated groups (Supplementary Material File S1, Figure S1). Thus, systemic electrolyte imbalance was unlikely to have confounded the behavioral or histological outcomes.

### 2.3. Behavioral Outcomes

**Figure 3:**
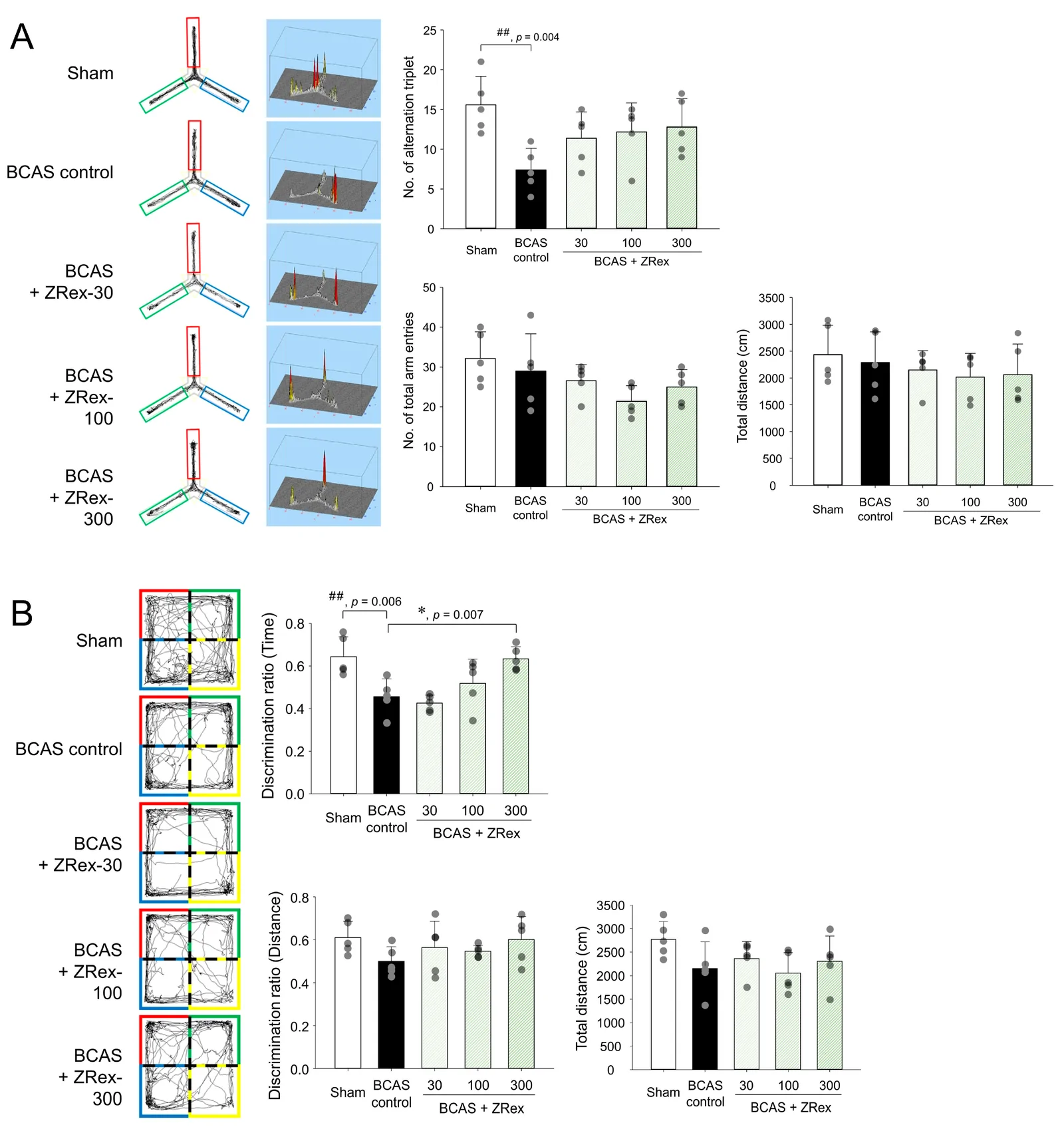
Behavioral performance in the Y-maze and novel object recognition test (NORT). (**A**) Y-maze assessment of spatial working memory and locomotor activity. Bilateral common carotid artery stenosis (BCAS) significantly reduces the number of alternation triplets relative to the sham group, and none of the Zingiberis Rhizoma ethanol extract (ZRex) doses significantly restores this measure. Total arm entries and distance traveled do not differ among the groups. (**B**) NORT assessment of recognition memory. BCAS reduces the time-based discrimination ratio relative to the sham group, whereas ZRex at 300 mg/kg significantly increases this ratio relative to the BCAS control group. The distance-based discrimination ratio and total distance traveled do not differ significantly among the groups. Values are presented as the mean ± standard deviation (SD). ^##^ *p* < 0.01 vs. the sham group; * *p* < 0.05 vs. the BCAS control group.

Spatial working memory and recognition memory were assessed using the Y-maze and novel object recognition test (NORT), respectively. In the Y-maze, mice subjected to BCAS completed significantly fewer alternation triplets than sham-operated mice, indicating impaired spatial working memory. None of the tested ZRex doses significantly increased alternation triplets relative to the BCAS control group. Total distance traveled and arm entries did not differ among the groups, indicating that general locomotor activity was preserved (Figure 3A).

In the NORT, mice subjected to BCAS had a significantly lower time-based discrimination ratio than sham-operated mice, indicating impaired recognition memory. ZRex at 300 mg/kg significantly increased the time-based discrimination ratio relative to the BCAS control group, suggesting partial recovery of recognition memory. However, the distance-based discrimination ratio and total distance traveled during the test phase did not differ among the groups (Figure 3B).

### 2.4. Immunofluorescence Analysis

**Figure 4:**
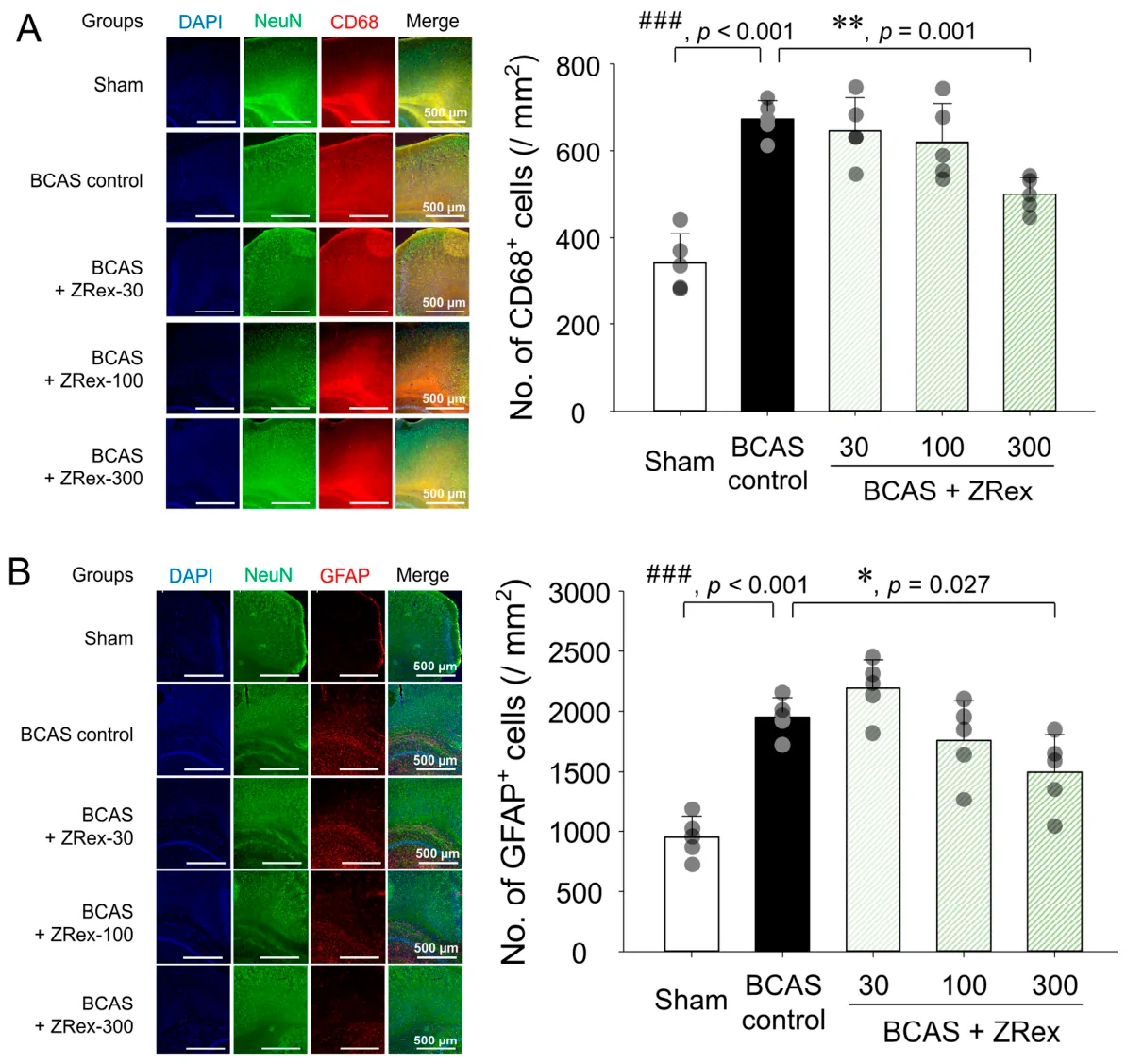
ZRex attenuates BCAS-induced microglial and astrocytic activation in the cerebral cortex and *corpus callosum*. (**A**) Representative immunofluorescence images of cluster of differentiation 68 (CD68)-positive cells (red), neuronal nuclei labeled with NeuN (green), and cell nuclei labeled with DAPI (blue) in the cerebral cortex and adjacent white matter. Scale bars = 500 µm. The bar graph shows CD68-positive cell density (cells/mm^2^). BCAS significantly increases CD68-positive cell density relative to the sham group, whereas ZRex at 300 mg/kg significantly reduces this increase relative to the BCAS control group. (**B**) Representative immunofluorescence images of glial fibrillary acidic protein (GFAP)-positive reactive astrocytes (red), NeuN-positive neuronal nuclei (green), and DAPI-labeled cell nuclei (blue). Scale bars = 500 µm. The bar graph shows GFAP-positive astrocyte density (cells/mm^2^). ZRex at 300 mg/kg significantly attenuates the BCAS-induced increase in GFAP-positive astrocyte density. Data are presented as the mean ± SD (*n* = 5 mice per group). Uncompressed, full-resolution source images are available in the Supplementary Raw Data package. ^###^ *p* < 0.001 vs. the sham group; * *p* < 0.05 and ** *p* < 0.01 vs. the BCAS control group, as determined by one-way analysis of variance followed by the Holm–Sidak *post hoc* test.

Immunofluorescence staining showed pronounced neuroinflammatory changes in the BCAS control group, and these changes were attenuated by ZRex. Immunoreactivity for cluster of differentiation 68 (CD68), a marker of microglial activation, was significantly higher in the BCAS group than in the sham group but was reduced by ZRex at 300 mg/kg (Figure 4A). Immunoreactivity for glial fibrillary acidic protein (GFAP), a marker of astrocytic activation, was also significantly increased by BCAS and reduced by ZRex at 300 mg/kg, indicating suppression of reactive gliosis (Figure 4B).

**Figure 5:**
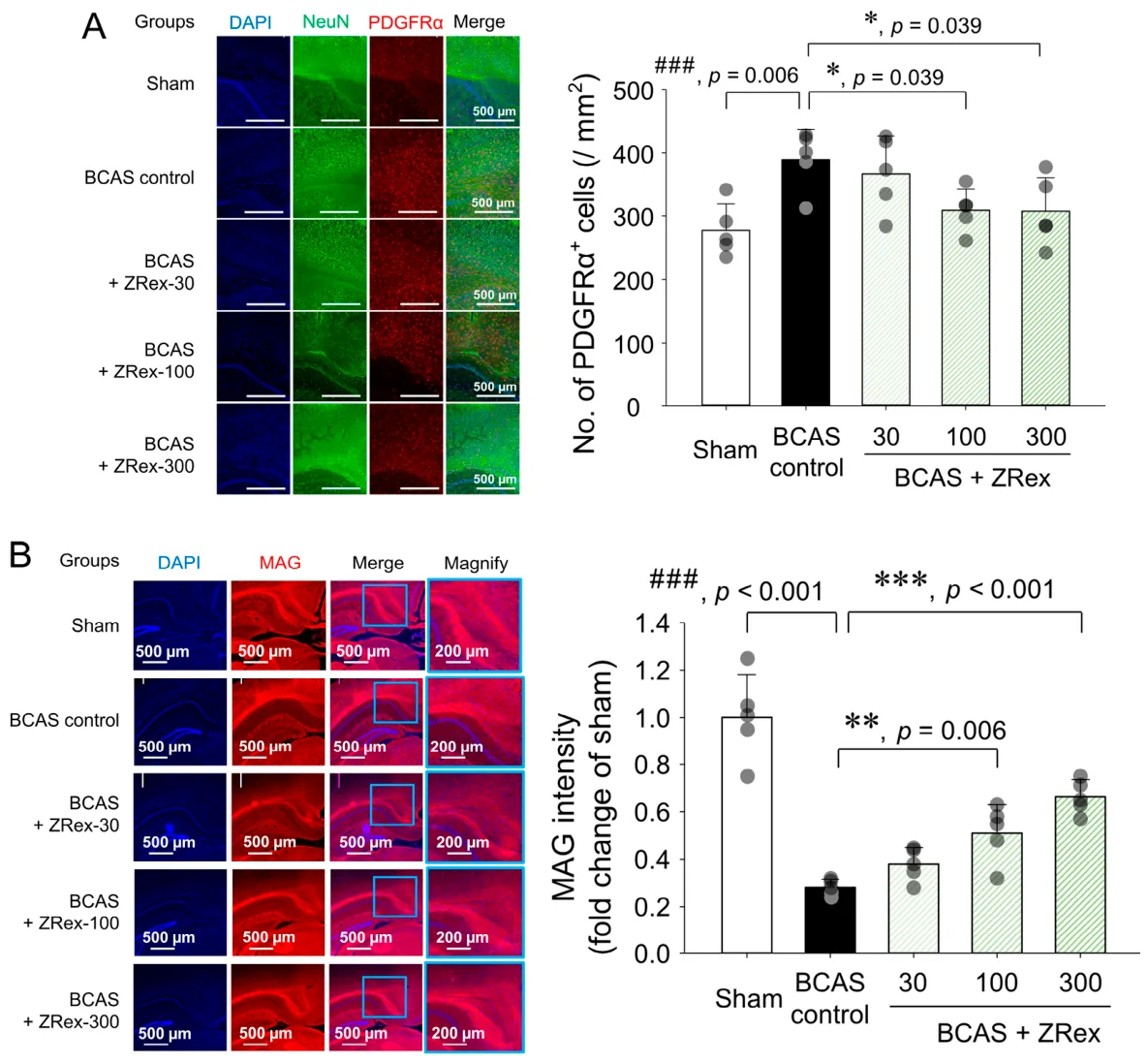
ZRex normalizes oligodendrocyte progenitor cell and myelin-associated markers after BCAS. (**A**) Representative images of platelet-derived growth factor receptor alpha (PDGFRα) immunostaining and quantification of PDGFRα-positive cell density per unit area. BCAS alters PDGFRα-positive cell counts, whereas ZRex at 100 and 300 mg/kg partially restores the counts toward sham levels. (**B**) Representative images of myelin-associated glycoprotein (MAG) immunofluorescence and quantification of relative fluorescence intensity normalized to the sham group. MAG immunoreactivity is preserved in the groups treated with ZRex at 100 and 300 mg/kg. Scale bars = 500 µm; inset scale bars = 200 µm. Data are presented as the mean ± SD. ^###^ *p* < 0.001 vs. the sham group; * *p* < 0.05, ** *p* < 0.01, and *** *p* < 0.001 vs. the BCAS control group.

BCAS also altered markers of oligodendrocyte and myelin integrity. BCAS significantly increased the number of platelet-derived growth factor receptor alpha (PDGFRα)-positive oligodendrocyte progenitor cells (OPCs). ZRex at 100 and 300 mg/kg significantly attenuated this increase and restored PDGFRα-positive cell counts toward sham levels (*p* = 0.039). ZRex also significantly preserved myelin-associated glycoprotein (MAG) immunoreactivity, indicating dose-dependent recovery of oligodendrocyte-lineage and myelin-associated proteins (Figure 5A,B). Collectively, these findings indicate that ZRex attenuated BCAS-induced microglial and astrocytic activation and normalized oligodendrocyte and myelin markers.

### 2.5. RNA Sequencing and PPI Network Analysis

**Figure 6:**
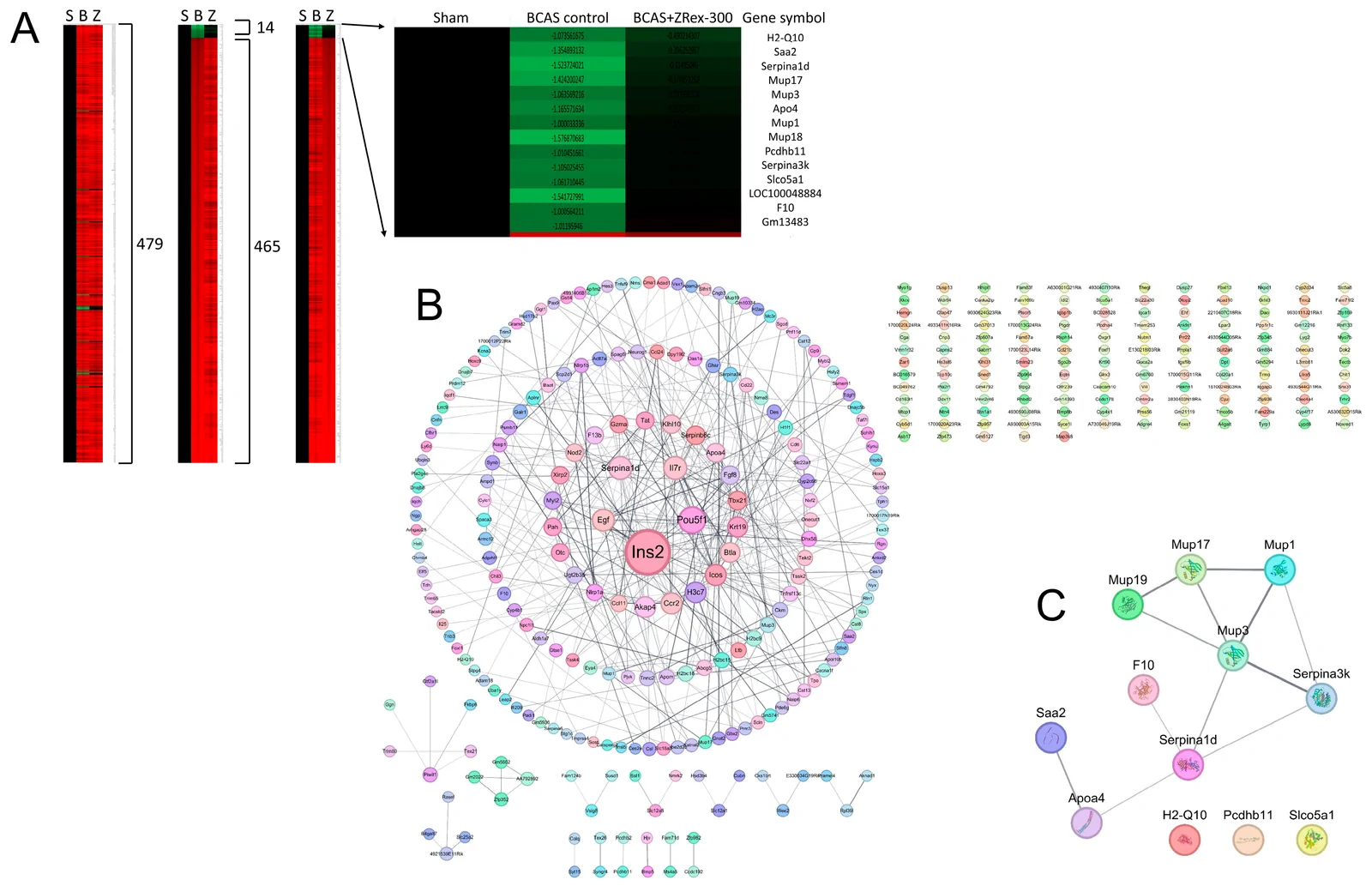
RNA sequencing analysis of genes modulated by BCAS and ZRex. (**A**) Heatmaps showing sequential transcriptomic filtering across the experimental groups (S, sham; B, BCAS control; Z, BCAS + ZRex at 300 mg/kg): global unsorted expression profiles for 16,086 genes (left); clustered expression of 479 genes meeting the fold-change thresholds of a ≥5.0-fold increase or ≤0.2-fold decrease relative to the sham group (middle); and an enlarged cluster of BCAS-downregulated genes normalized by ZRex (right). Hierarchical clustering was generated using MultiExperiment Viewer (MeV) based on Euclidean distance and average linkage clustering. Complete expression data are provided in Supplementary Material File S2. (**B**) Global PPI network of the 479 BCAS-responsive genes showing a modular architecture. (**C**) Subnetwork of core ZRex-responsive targets enriched in metabolic, transport, and acute-phase regulatory proteins, including Mup family members, Serpina3k, Serpina1d, Saa2, Apoa4, and F10.

RNA sequencing (RNA-seq) of the cerebral cortex and adjacent white matter (*n* = 3 per group) generated expression profiles for 16,086 transcripts. Applying the fold-change thresholds (≥5.0-fold increase or ≤0.2-fold decrease vs. the sham group) identified 479 BCAS-responsive candidate genes, most of which were upregulated (Figure 6A). ZRex at 300 mg/kg attenuated the expression changes in a subset of genes downregulated by BCAS; no genes upregulated by BCAS met the attenuation criterion.

PPI analysis of the BCAS-responsive genes revealed a densely connected network. Hub genes occupied central positions in the circular layout, and the linear representation showed a modular organization (Figure 6B). In contrast, the network of ZRex-responsive genes contained distinct interaction partners, including major urinary protein (Mup) family members and acute-phase or transport proteins such as Serpina3k, Serpina1d, Saa2, and Apoa4 (Figure 6C) [15,16,17,18]. Relative to the BCAS-responsive network, the ZRex-responsive network had a concentrated core of treatment-sensitive nodes, suggesting selective modulation of metabolic, inflammatory, and transport pathways.

### 2.6. Molecular Docking of 6-Gingerol

**Figure 7:**
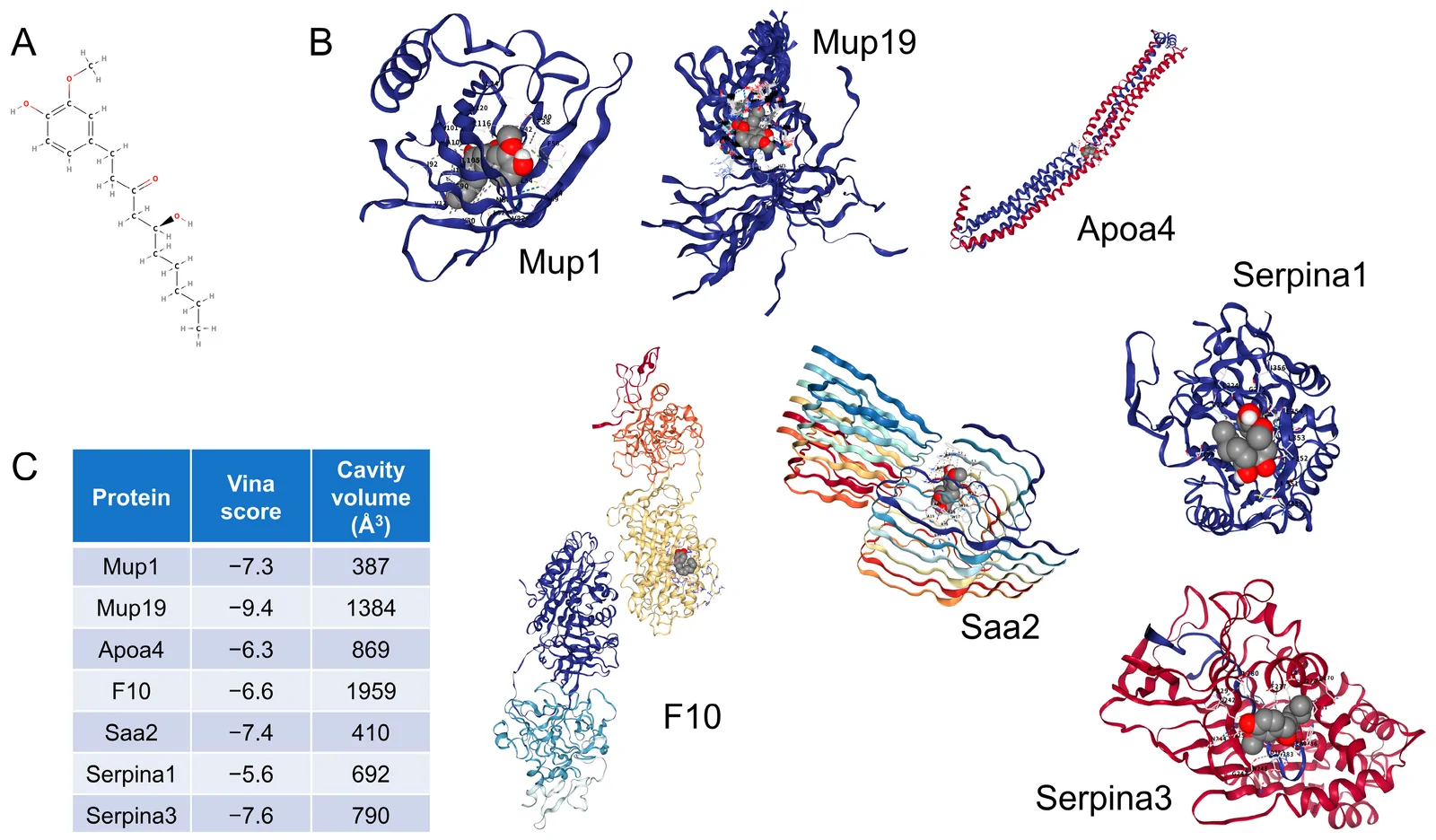
*In silico* docking of 6-gingerol to the principal ZRex-modulated targets identified by RNA sequencing. (**A**) Two-dimensional structure of 6-gingerol, a principal bioactive constituent of ZRex. (**B**) Predicted three-dimensional binding poses of 6-gingerol within the binding pockets of Mup1, Mup19, Apoa4, F10, Saa2, Serpina1, and Serpina3, generated using CB-DOCK2. Key amino acid residues in each predicted binding cavity are shown. (**C**) Molecular docking parameters, including the Vina score (kcal/mol) and predicted cavity volume (Å^3^). Lower Vina scores indicate stronger predicted binding. The most favorable predicted interactions are reported for Mup19 (Vina score, −9.4 kcal/mol) and coagulation factor F10 (Vina score, −6.6 kcal/mol), suggesting potential direct interactions relevant to cerebrovascular and metabolic pathways.

To investigate potential direct molecular interactions, 6-gingerol was docked using CB-DOCK2 to targets identified by RNA sequencing. Predicted binding poses and Vina scores were generated for each protein (Figure 7). The strongest predicted affinities were observed for Mup19 and coagulation factor F10, whose larger cavity volumes were consistent with favorable steric complementarity. Moderate predicted binding was observed for Mup1, Saa2, and Apoa4, whereas members of the Serpina family had weaker scores (Figure 7B,C).

These in silico findings suggest that 6-gingerol may interact directly with proteins modulated by ZRex, providing a potential mechanistic link between a defined constituent and the transcriptomic changes observed in vivo. However, the docking predictions are hypothesis-generating and require validation in biophysical or cellular assays. On the basis of the strongest predicted interactions, Mup19 and F10 are high-priority candidates for follow-up studies examining whether 6-gingerol binding alters their activity and contributes to the effects of ZRex on inflammatory, metabolic, and vascular processes in the BCAS model.

## 3. Discussion

ZR, the dried rhizome of ginger, has been extensively investigated for its potential effects on cerebrovascular disease and cognitive decline. Its therapeutic activity is attributed to several bioactive compounds, particularly shogaols, which are formed from gingerols and become concentrated during drying [19]. Preclinical and ethnopharmacological studies have highlighted the cerebrovascular protective properties of ginger constituents, especially 6-gingerol. Network-based analyses, for example, have identified 6-gingerol as a quality marker associated with anti-inflammatory, antioxidant, and vascular protective activities [20]. Studies of gingerol-containing formulations have reported improved cerebral perfusion, preserved neuronal architecture, increased extracellular matrix deposition, upregulated angiogenic markers, reduced glial activation, and attenuated oxidative stress in models of hypoxia-related brain injury [21]. Together, these findings indicate multimodal activity that warrants biochemical validation and translational investigation.

Gingerols may protect the neurovascular unit by improving cerebral blood flow and microcirculation, and by preserving endothelial integrity. Gingerol-based preparations may also counteract CCH and stabilize cerebral perfusion by reducing vascular stiffness and modulating platelet aggregation [21,22,23]. In addition, ZR has potent antioxidant and anti-inflammatory activities relevant to ischemic brain injury. CCH is a major pathophysiological driver of VaD and promotes progressive neuroinflammation, white matter rarefaction, and cognitive decline [1,2,3,4]. Persistent oxidative stress and secondary inflammation further accelerate neuronal injury after CCH. Shogaols effectively scavenge reactive oxygen species (ROS) and activate endogenous antioxidant defenses, including nuclear factor erythroid 2-related factor 2 (Nrf2) signaling [24]. In parallel, ZR constituents suppress central inflammatory pathways by inhibiting nuclear factor kappa B (NF-κB) activation and downregulating proinflammatory cytokines. Thus, ZR targets several fundamental processes underlying vascular cognitive impairment, including vascular dysfunction, oxidative damage, and neuroinflammation [22,23].

As the principal pungent phenolic constituent of ginger, 6-gingerol combines antioxidant and anti-inflammatory activities through ROS scavenging, activation of endogenous defenses, and suppression of cytokine signaling. These complementary mechanisms may reduce oxidative injury and chronic neuroinflammation, supporting the investigation of 6-gingerol as a candidate treatment for ischemic and hypoperfusion-related brain injury [21,22,24]. However, studies evaluating whole ginger extracts remain limited.

In this study, oral ZRex administration partially protected against BCAS-induced cognitive impairment and neuroinflammation. Behavioral recovery was most evident in the NORT at 300 mg/kg and was accompanied by reduced microglial and astrocytic activation (Figure 4A,B) and partial normalization of oligodendrocyte and myelin markers (Figure 5A,B). Transcriptomic profiling showed widespread BCAS-induced perturbations, whereas ZRex selectively modulated a subset of downregulated genes that formed a compact PPI network enriched in metabolic and transport functions (Figure 6A–C). Molecular docking further predicted plausible interactions between 6-gingerol and several ZRex-regulated proteins, with Mup19 and F10 showing the strongest predicted affinities. Collectively, these findings suggest that ZRex exerts neuroprotective effects through anti-inflammatory activity and selective transcriptomic modulation, with 6-gingerol as a potential mechanistic contributor.

The combined immunostaining, transcriptomic, and docking findings suggest that ZRex acts on discrete molecular modules rather than globally reversing BCAS-induced transcriptional changes. Specifically, ZRex modulated inflammatory and metabolic pathways associated with oligodendrocyte and myelin homeostasis and glial reactivity. Among the ZRex-responsive nodes, the prominence of acute-phase and transport proteins, including Saa2, members of the Serpina and Mup families, and Apoa4, suggests systemic metabolic and inflammatory communication between peripheral organs and the brain. The predicted interaction between 6-gingerol and coagulation factor F10 further raises the possibility that vascular and coagulation pathways contribute to the cerebrovascular effects of ZRex. Transcriptomic profiling and molecular docking identified candidate targets that provide specific neurobiological links to ischemic pathophysiology. F10 is an important mediator of microvascular thrombosis and thrombin-mediated disruption of the neurovascular unit during CCH [25]. Saa2 and Apoa4 regulate acute-phase inflammation and lipid transport and participate in cerebrovascular endothelial activation and blood–brain barrier integrity [26,27]. In addition, Mup19 may function as part of a systemic metabolic signaling hub that modulates susceptibility to neuroinflammation under ischemic stress [28].

Neuroinflammation mediated by activated microglia and reactive astrocytes is central to CCH-induced brain injury [1,2,21]. Following ischemia, microglia rapidly adopt a proinflammatory phenotype and release ROS and inflammatory cytokines, whereas reactive astrocytes contribute to neurovascular unit dysfunction and scar formation [21,22]. In the present study, BCAS markedly increased CD68, a marker of lysosomal activity in activated microglia, and GFAP, a marker of astrocyte reactivity, in the cerebral cortex and adjacent white matter tracts. ZRex at 300 mg/kg significantly reduced both CD68 and GFAP immunoreactivity, demonstrating a pronounced antigliotic effect [21]. ZRex at 100 and 300 mg/kg also normalized PDGFRα-positive oligodendrocyte progenitor cell counts and preserved MAG intensity, indicating protection against hypoperfusion-induced alterations in oligodendrocytes and myelin [3,4]. Because white matter injury correlates with executive and memory deficits in VaD [1,3], preservation of oligodendrocyte and myelin markers may contribute to the improvement observed in the NORT. The differential responses in the NORT and Y-maze may reflect distinct circuit vulnerabilities during CCH. Spatial working memory in the Y-maze depends strongly on long-range prefrontal-hippocampal projections, which are susceptible to disconnection caused by extensive white matter rarefaction [29,30,31]. In contrast, novel object recognition primarily depends on perirhinal and entorhinal cortical networks [32,33]. Thus, within the study period, ZRex may have preferentially preserved cortical recognition memory without fully restoring long-range hippocampal circuitry.

Transcriptomic profiling was performed to investigate the broader molecular mechanisms associated with ZRex. Differential gene expression in the CCH model indicated two interconnected pathological features: localized neuroinflammation and systemic metabolic disruption. Upregulated gene clusters included chemokines, such as Ccl11 and Ccl24, and inflammasome components, including Nlrp1a and Nlrp1b, consistent with persistent ischemic inflammation; T-cell regulatory genes were also elevated (Supplementary Material File S2). Increased expression of neurodevelopmental genes, including Neurog1 and Hoxc9, may reflect unsuccessful attempts at neuroplastic adaptation (Supplementary Material File S2). Conversely, downregulation of Mup family members and Apoa4 may suggest potential alterations in systemic metabolic cross-talk and lipid homeostasis. While these brain transcriptomic changes highlight candidate pathways, further systemic and hepatic studies are warranted to confirm peripheral organ involvement. Therapeutic strategies may therefore need both to inhibit the NLRP1 and chemokine axis, thereby reducing neuroinflammation, and to restore Apoa4- and Mup-related metabolic balance.

The targets predicted by TCMSP-based network pharmacology did not directly overlap with the RNA-seq findings. This discrepancy underscores the limitations of current herbal compound–target databases and the value of empirical transcriptomic profiling. Potential explanations include indirect regulation of transcript abundance, the limited cell-type resolution of bulk RNA-seq, and the in vivo metabolic transformation of bioactive compounds. Molecular docking offers one approach for linking 6-gingerol to the observed molecular changes, but biochemical validation remains necessary.

A major strength of this study is its multilevel design, which integrates behavioral, histological, transcriptomic, network, and molecular-docking analyses to provide complementary perspectives on the effects of ZRex in a validated CCH model. The study is limited by modest group sizes, the use of bulk RNA-seq, and reliance on predictive rather than experimentally validated docking scores. Moreover, ZRex is a complex mixture, and focusing exclusively on 6-gingerol may underestimate the contributions of other constituents. Future studies should use binding assays, targeted quantitative polymerase chain reaction, Western blotting, cell-type-resolved transcriptomics, pharmacokinetic profiling, and pathway-modulation experiments to establish causal links between compound–target interactions and functional recovery.

## 4. Materials and Methods

### 4.1. In Silico Network Pharmacology Analysis

Bioactive compounds in ZR were identified using TCMSP [34]. Compounds were screened according to the following absorption, distribution, metabolism, and excretion (ADME) criteria: OB ≥ 20%, DL ≥ 0.18, and Caco-2 permeability ≥ 0. Common targets and disease associations were extracted to identify core targets. Cytoscape version 3.10.3 was used to construct compound-disease and compound–target networks and to generate Venn diagrams.

### 4.2. Animals and Housing

Male C57BL/6 mice (6 weeks old; 20–22 g) were purchased from a licensed breeder and acclimated for 2 weeks. The mice were housed five per cage under a 12 h light-dark cycle at 24 ± 4 °C, with *ad libitum* access to standard chow and water. All procedures were approved by the Pusan National University Institutional Animal Care and Use Committee (PNU-2025-0655).

### 4.3. Induction of CCH

**Figure 8:**
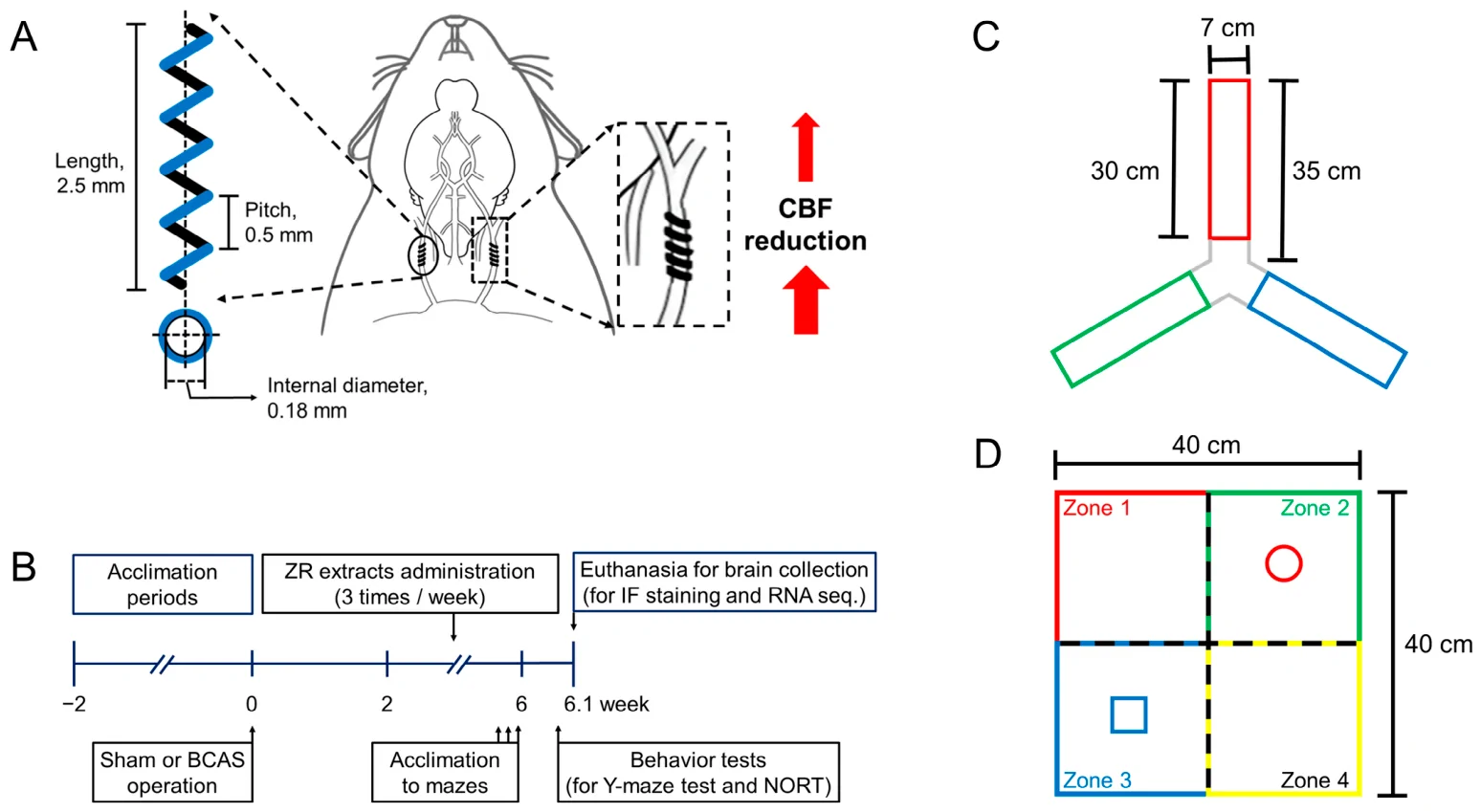
Experimental design and ZRex treatment groups in the BCAS model. (**A**) Schematic of the BCAS procedure and treatment schedule. Mice receive ZRex or vehicle orally three times per week after BCAS and undergo behavioral testing before tissue collection. (**B**) Timeline of acclimation, BCAS or sham surgery, repeated oral administration, behavioral testing, and tissue collection for immunofluorescence analysis and RNA sequencing. (**C**,**D**) Schematic representations of the Y-maze and the open-field arena used for the NORT, respectively.

CCH was induced using the BCAS model. Under isoflurane anesthesia (3% for induction and 1.5% for maintenance in 70% N_2_O and 30% O_2_), a midline cervical incision was made to expose both common carotid arteries (CCAs). Each CCA was encircled with a microcoil of 0.18 mm internal diameter (Figure 8A). Rectal temperature was maintained at 37 ± 0.5 °C throughout surgery using a thermostatically controlled heating pad.

### 4.4. Reagents

The following reagents were used: physiological saline (Joongwae Pharmaceutical, Seoul, Republic of Korea); optimal cutting temperature (OCT) compound (Bio Basic, Markham, ON, Canada); phosphate-buffered saline (PBS; Thermo Fisher Scientific, Waltham, MA, USA); ethanol (99.9%, general reagent grade; CAS No. 64-17-5; C_2_H_5_OH; DUKSAN, Yongin, Gyeonggi, Republic of Korea); bovine serum albumin (BSA; Probumin^®^ BSA, Fraction V; Sigma-Aldrich, St. Louis, MO, USA); and TRIzol reagent (cat. no. 15596018; Thermo Fisher Scientific, Waltham, MA, USA).

### 4.5. Preparation and Dose Determination of ZRex

ZR was obtained from Gwangmyung Pharmaceutical Co., Ltd. (Ulsan, Republic of Korea), and a voucher specimen (No. 24-ZR-G) was deposited in the herbarium of the Pusan National University School of Korean Medicine. High-performance liquid chromatography (HPLC) confirmed compliance with Korean Pharmacopeia standards, with a quantified 6-gingerol content of 0.52% in the experimental ZRex batch (exceeding the standard threshold of ≥0.4%; Supplementary Material File S1, Figure S2). ZRex was prepared by refluxing powdered ZR in 99.9% ethanol, followed by filtration and solvent evaporation under reduced pressure. The extraction yield was 11.2% by dry weight.

Dose selection was based on traditional use and extraction yield. A standard adult dose of ZR (12 g/day for a 60 kg adult, approximately 0.2 g/kg) corresponds to 22.4 mg/kg ZRex. We adjusted for murine metabolism using a conversion factor of 13 [26,35], which yielded an equivalent dose of 291.2 mg/kg. Accordingly, three doses were selected: 30 mg/kg (ZRex-30), 100 mg/kg (ZRex-100), and 300 mg/kg (ZRex-300). The extracts were freshly prepared in distilled water before administration.

### 4.6. Treatment Groups and Administration

Mice were randomly assigned to five groups (*n* = 8 per group; zero mortality): sham + vehicle, BCAS + vehicle, BCAS + ZRex-30, BCAS + ZRex-100, and BCAS + ZRex-300. Within each group, five mice were randomly allocated to perfusion fixation for immunofluorescence staining, and the remaining three mice were allocated to fresh-tissue collection for RNA sequencing. ZRex or vehicle was administered orally three times per week (Figure 8B). Sham-operated mice received vehicle and underwent identical handling procedures.

### 4.7. Behavioral Assessments

Y-maze test: Spatial working memory was assessed in a Y-shaped maze comprising three arms positioned at 120° angles (Figure 8C). Mice were habituated to the maze for 5 min/day on 2 consecutive days. On the test day, each mouse was allowed to explore the maze freely for 10 min. Locomotor activity, including distance traveled and number of arm entries, was recorded by video tracking. Alternation behavior was quantified as the number of successful triplets, defined as consecutive entries into all three arms without repetition, and was used as a direct measure of working memory [29,30,31].

Novel object recognition test: Recognition memory was evaluated in a 40 × 40 × 40 cm open-field arena (Figure 8D). After 2 days of habituation, mice were exposed to two identical objects for 10 min during the familiarization phase, followed by a 30 min retention interval. During the test phase, one familiar object was replaced with a novel object. Object exploration was defined as orientation of the nose toward an object at a distance of ≤2 cm or contact with the object using the snout or whiskers. Object types and locations were counterbalanced across mice [32,33,36], and no baseline object preference was detected during familiarization (*p* > 0.05). An investigator blinded to treatment allocation scored all behavioral data. The time-based discrimination ratio (DR time) was designated as the primary measure of active recognition memory, whereas the distance-based discrimination ratio (DR distance) was analyzed as a secondary exploratory measure [37]. Discrimination ratios were calculated as follows:

DR distance = distance to novel object/(distance to novel object + distance to familiar object)

DR time = time exploring novel object/(time exploring novel object + time exploring familiar object)

The distance-based ratio quantifies novelty preference based on movement toward the novel object, whereas the time-based ratio quantifies active exploration of it [29,30,31].

### 4.8. Plasma Electrolyte Measurement

To assess systemic changes associated with surgery and VaD modeling, plasma electrolytes were measured under deep anesthesia. Whole blood was collected and centrifuged at 3000× *g* for 15 min at 4 °C to isolate plasma. Sodium (Na^+^), potassium (K^+^), and chloride (Cl^−^) concentrations were measured using an automated analyzer (Dri-Chem 3500i, Fuji, Chiryu, Japan). Electrolyte balance is essential for neuronal excitability, synaptic transmission, and blood–brain barrier homeostasis. Accordingly, disturbances in sodium and potassium concentrations are associated with cognitive impairment, mood changes, confusion, and memory deficits [38]. Hyponatremia, in particular, is associated with an increased risk of dementia [39]. Plasma electrolyte monitoring therefore helped determine whether systemic imbalance confounded the behavioral outcomes.

### 4.9. Euthanasia, Cardiac Perfusion, and Tissue Sectioning

After behavioral testing, the mice were euthanized. For immunofluorescence analysis (*n* = 5 per group), mice were deeply anesthetized and perfused transcardially. After a thoracic incision exposed the heart, a 21-gauge needle was inserted into the ascending aorta, and the right atrium was opened to permit drainage. Mice were perfused first with ice-cold PBS until the effluent was clear and then with 4% paraformaldehyde in PBS. Brains were cryoprotected sequentially in 10%, 20%, and 30% sucrose, embedded in OCT compound, and stored at −80 °C. Coronal sections (25 µm) were prepared using a cryostat and stored at −80 °C until histological analysis. For transcriptomic analysis (*n* = 3 per group), mice were euthanized by CO_2_ inhalation, and fresh cerebral cortex and adjacent white matter were rapidly collected and snap-frozen.

### 4.10. Immunofluorescence Staining

Frozen brain sections were air-dried and blocked with 5% BSA for 1 h at 25 °C. After washing with PBS, the sections were incubated overnight at 4 °C with primary antibodies against neuronal nuclei (NeuN; Cell Signaling Technology, Danvers, MA, USA; cat. no. 94403), CD68 (Cell Signaling Technology; cat. no. 97778), glial fibrillary acidic protein (GFAP; Cell Signaling Technology; cat. no. 12389), myelin-associated glycoprotein (MAG; Cell Signaling Technology; cat. no. 9043), and platelet-derived growth factor receptor alpha (PDGFRα; Abcam, Cambridge, UK; ab203491). After washing, species-specific fluorescent secondary antibodies (Thermo Fisher Scientific, A-11008 and A-11005; Abcam, ab150135; Sigma-Aldrich, T5393) were applied for 2 h at 25 °C. Sections were mounted using DAPI-containing antifade medium (Abcam; ab104139) and sealed. Images were acquired using a Nikon Ni-U fluorescence microscope (Nikon Corporation, Tokyo, Japan) and processed with ImageJ (version 1.54g; National Institutes of Health, Bethesda, MD, USA). Immunoreactivity was quantified by counting positive cells per unit area and measuring fluorescence intensity using Digimizer software (version 4.6.1; MedCalc Software Ltd., Ostend, Belgium). For immunofluorescence quantification, four coronal sections per mouse brain were evaluated. In each section, three non-overlapping microscopic fields within the cerebral cortex and *corpus callosum* were randomly captured. Quantitative metrics from the twelve total fields per animal were averaged to obtain a single representative value for each biological replicate (*n* = 5 per group).

### 4.11. RNA Sequencing, Visualization, and Molecular Docking

The cerebral cortex and adjacent white matter were dissected after CO_2_ euthanasia and snap-frozen. Total RNA was extracted using TRIzol reagent, and RNA integrity and purity were confirmed using capillary electrophoresis on an Agilent 2100 Bioanalyzer (Agilent, Santa Clara, CA, USA). High-throughput sequencing was conducted following mRNA isolation and cDNA library construction.

Raw sequencing reads were quality-checked using FastQC v0.12.1 and aligned to the mouse reference genome (GRCm38/mm10) using the STAR aligner (v2.7.9a). Gene expression levels were quantified using ExDEGA software (version 1.6.8; E-Biogen, Seoul, Republic of Korea) and normalized to the sham group baseline. Candidate genes were selected based on a fold-change threshold (≥5.0-fold increase or ≤0.2-fold decrease vs. sham). Hierarchical clustering of fold-change-filtered candidate genes was performed using MultiExperiment Viewer (MeV, v4.9.0; Dana-Farber Cancer Institute, Boston, MA, USA). Distance matrix calculation was based on Euclidean distance, and cluster agglomeration was achieved using the Average linkage method. Visual representation and Z-score color mapping were rendered based on MeV output matrices. Differential expression was determined relative to the sham group. Normalized expression values were visualized as heatmaps in Microsoft Excel using conditional formatting. PPI networks for candidate genes were retrieved from STRING [40] and analyzed using Cytoscape version 3.10.3. Selected protein targets were subjected to molecular docking using CB-DOCK2 [41]. Three-dimensional crystal structures of the candidate target proteins were retrieved from the Protein Data Bank (PDB): Mup1 (PDB ID: 1MUP), Mup19 (PDB ID: 1I04), Apoa4 (PDB ID: 3S84), F10 (PDB ID: 1C5M), Saa2 (PDB ID: 4PVI), Serpina1 (PDB ID: 1QLP), and Serpina3 (PDB ID: 1AS4) [42]. Blind molecular docking simulations were performed using CB-DOCK2. 6-Gingerol was selected for target docking as the principal active marker constituent of ZRex with demonstrated brain bioavailability, expanding upon systemic TCMSP screening.

### 4.12. Statistical Analysis

Data are presented as the mean ± standard deviation. Normality was assessed using the Shapiro–Wilk test in SigmaPlot 12.0. Normally distributed variables (*p* > 0.05) were analyzed using one-way analysis of variance (ANOVA), followed by Holm–Sidak *post hoc* comparisons. All tests were two-tailed, and statistical significance was defined as *p* < 0.05.

## 5. Conclusions

ZRex attenuated key immunopathological and cognitive alterations in the BCAS model. Integrative analyses implicated selective modulation of metabolic, inflammatory, and vascular pathways as plausible mechanisms underlying these effects. However, the network pharmacology predictions did not consistently overlap with the RNA sequencing findings, and the effects of 6-gingerol were not evaluated directly. Further studies should use 6-gingerol as a marker compound to validate the predicted protein targets and determine its contribution to the effects of ZRex on neuroinflammation and vascular function in models of vascular cognitive impairment.

## Figures and Tables

**Table 1:** Candidate active compounds in Zingiberis Rhizoma (ZR) and their physicochemical and ADME (absorption, distribution, metabolism, and excretion) properties.

Mol ID	Molecule Name	MW	OB (%)	Caco-2	DL
MOL000358	Beta-sitosterol	414.79	36.91	1.32	0.75
MOL006129	6-methylgingediacetate2	394.56	48.73	0.55	0.32
MOL006133	(E)-1-(4-hydroxy-3-methoxyphenyl)dodec-4-en-3-one	304.47	23.77	1.1	0.19
MOL000449	Stigmasterol	412.77	43.83	1.44	0.76
MOL001771	Poriferast-5-en-3beta-ol	414.79	36.91	1.45	0.75
MOL008698	Dihydrocapsaicin	307.48	47.07	0.98	0.19

The six candidate bioactive compounds are selected from ZR by Traditional Chinese Medicine Systems Pharmacology Database and Analysis Platform (TCMSP) screening. The reported prioritization parameters include molecular weight (MW), oral bioavailability (OB), Caco-2 permeability, and drug-likeness (DL).

## Data Availability

The data supporting the findings of this study are available in the article and its Supplementary Materials.

## Supplementary Materials

The following supporting information can be downloaded at: https://www.mdpi.com/article/10.3390/molecules31203634/s1. Supplementary Material File S1: Figure S1, Physiological assessments following BCAS and ZRex treatment; Figure S2, Representative quality-control data for ZRex. Supplementary Material File S2: RNA-seq Gene Expression.xlsx.
