# Supplementary material for: Zingiberis Rhizoma Ethanol Extract Attenuates Recognition Memory Deficits and Neuroinflammation in a Mouse Model of Bilateral Common Carotid Artery Stenosis
Source: Molecules. 2026 Oct 12;31(20):3634. doi: 10.3390/molecules31203634 (PMC13707571; doi:10.3390/molecules31203634)
Supplement: Supplementary file 1 [file molecules-31-03634-s001.zip › Supplementary Material S1.pdf]

Zingiberis Rhizoma Ethanol Extract Attenuates Recognition Memory Deficits and Neuroinflammation in a Mouse Model of Bilateral Common Carotid Artery Stenosis

List of supplementary Figures

Figure S1. Physiological assessments following BCAS and ZRex treatment

Figure S2. Representative quality-control data for ZRex

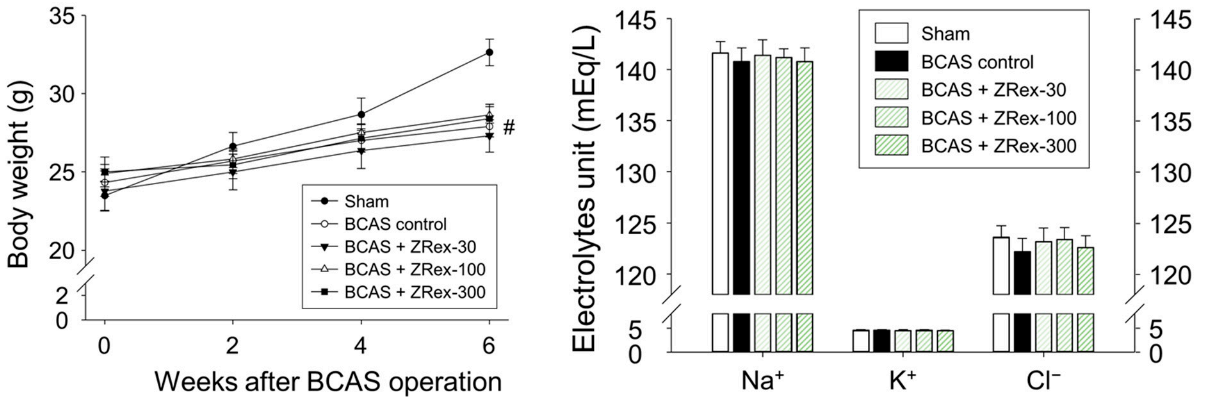

Figure S1. Physiological assessments following BCAS and ZRex treatment. (A) Body weight trajectories measured biweekly for 6 weeks after surgery in each experimental group. Data are plotted as mean ± SD at baseline and at two subsequent 2-week intervals. The sham group maintained stable body weight over the observation period; BCAS groups showed variable weight loss with partial dose-dependent attenuation in ZRex-treated animals. $^{\#}$, statistical significance compared to sham group ($p < 0.05$). (B) Plasma sodium (Na^+), potassium (K^+), and chloride (Cl^-) concentrations are presented. Values are mean ± SD; no significant intergroup differences were observed, indicating preserved systemic electrolyte homeostasis.

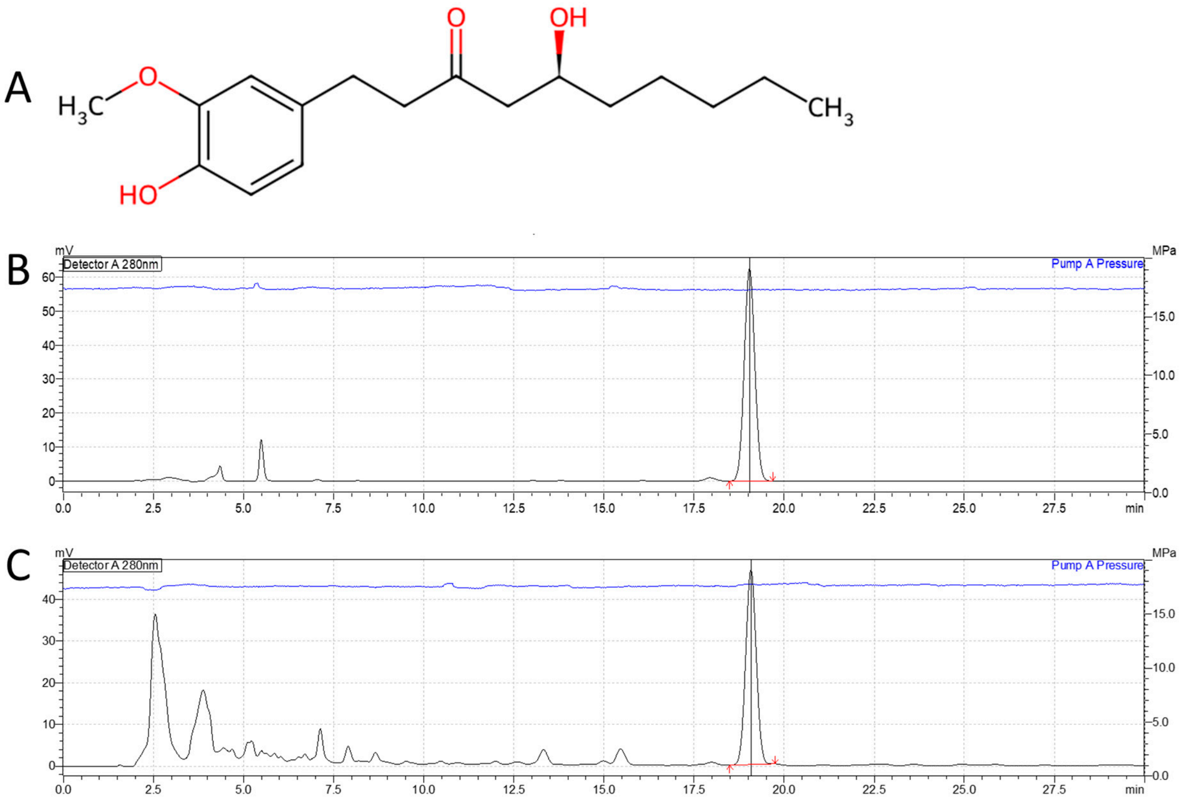

Figure S2. Representative quality-control data for ZRex. (A) Chemical structure of the marker compound 6-gingerol. (B) HPLC chromatogram of the 6-gingerol reference standard. (C) HPLC chromatogram of the ZRex sample showing the corresponding 6-gingerol peak. HPLC conditions: SHIMADZU system (pump LC-20AD; autosampler SIL-20A; CBM-20A communication module; detector SPD-20A, detection wavelength 280 nm; oven CTO-20A); mobile phase water: acetonitrile (55:45); column YMC-Triart C18; column temperature 34 ℃; flow rate 1.0 mL/min; injection volume 10 µL.
