# Supplementary material for: Zingiberis Rhizoma Ethanol Extract Attenuates Recognition Memory Deficits and Neuroinflammation in a Mouse Model of Bilateral Common Carotid Artery Stenosis
Source: Molecules. 2026 Oct 12;31(20):3634. doi: 10.3390/molecules31203634 (PMC13707571; doi:10.3390/molecules31203634)
Supplement: Supplementary file 1 [file molecules-31-03634-s001.zip › Supplementary Material S2-1 Expression Value.pdf]

Filter: 16086			Normalized data		2299	Btg2	6.471552402	6.137019261	6.58048477	4599	Eftud1	5.965443994	5.941516349	6.390039939	6899	Hnrnpab	9.713441828	9.780769846	9.612965264	9199	Nav1	9.677901507	9.183753605	9.399983992	11499	Rbck1	8.254948048	8.104380765	8.164895669	13799	Tbc1d32	6.901220271	6.935499661	6.874630804
ID	Gene symbol	Sham	BCAS control	Zrex	2300	Btg3	5.252834418	5.729278697	5.337291622	4600	Eftud2	7.894506604	7.977857014	8.019014203	6900	Hnrnpc	10.76445246	10.68629224	10.6942114	9200	Nav2	6.650640593	6.951030954	6.549544349	11500	Rbfa	6.456106597	6.832412246	6.519178628	13800	Tbc1d4	5.939095551	5.90192355	6.390517496
1	0610005C13Rik	2.049719566	1.894937247	1.571460045	2301	Btg3	5.633256772	5.701126854	5.471725914	4601	Egf	0.035158901	1.894880372	0.985633704	6901	Hnrnpd	9.389146647	9.567645474	9.627495436	9201	Nav3	8.488491951	8.436364766	8.359592224	11501	Rbfox1	11.70860253	11.78726088	11.75146674	13801	Tbc1d5	6.589380722	6.577126562	6.825764951
2	0610007P14Rik	8.801270193	8.63196153	8.728216621	2302	Btk	3.055914394	2.453518628	1.98206193	4602	Egfem1	5.879435268	4.726216216	5.504402172	6902	Hnrnpdl	9.724645148	9.964806232	9.960158122	9202	Nbas	6.292644995	6.784800825	6.503461457	11502	Rbfox2	9.301811808	9.099656891	9.206424929	13802	Tbc1d7	5.755713773	6.236389652	5.734137443
3	0610009B22Rik	6.524654304	6.35584726	6.638191383	2303	Btla	0.013869992	0.921284268	1.575588721	4603	Egfl6	3.891695546	3.827610283	4.937731679	6903	Hnrnpf	7.32869718	7.415979672	6.956532849	9203	Nbea	11.07341379	10.87044293	10.98210149	11503	Rbfox3	10.92669739	10.82091987	10.81683603	13803	Tbc1d8	6.571206868	6.750215656	6.487865554
4	0610009L18Rik	4.544053947	5.209254891	4.623218441	2304	Btn1a1	0.021985855	1.493079661	1.989545276	4604	Egfl7	7.068893582	7.301214029	7.067529711	6904	Hnrnph1	8.993886536	9.210886356	9.283831278	9204	Nbeal1	8.360722772	8.000942069	8.499898483	11504	Rbks	2.88667639	4.048980291	3.151479753	13804	Tbc1d8b	6.034030537	6.321213273	5.734087264
5	0610009O20Rik	5.941700414	6.531148773	6.246909078	2305	Btrc	9.4702488	9.247212452	9.184520091	4605	Egfl8	8.36603183	8.630688004	8.354997546	6905	Hnrnph2	8.14052101	7.951258684	8.199394614	9205	Nbeal2	2.873621197	3.040302973	1.979379149	11505	Rbl1	1.098992686	2.162879393	1.979590506	13805	Tbc1d9	8.285283216	8.598001551	8.29737322
6	0610010B08Rik	4.885631436	4.641076531	4.787475721	2306	Bub1b	2.085312429	3.461790994	3.570257949	4606	Egflam	3.530939166	2.111608202	3.567300871	6906	Hnrnph3	8.592198084	8.629445204	8.645392431	9206	Nbl1	7.866822913	7.516554546	7.680567844	11506	Rbl2	8.672718077	8.509769515	8.690878076	13806	Tbc1d9b	10.47912392	10.33472468	10.44252137
7	0610010F05Rik	8.178728512	8.163884647	8.368160647	2307	Bub3	9.011177498	8.927744195	8.945755711	4607	Egfr	4.836553325	4.404442209	5.150910319	6907	Hnrnpk	11.04490943	10.98685185	11.02232286	9207	Nbn	7.240103841	6.963682422	7.504065837	11507	Rbm10	7.787911684	7.899652913	7.645128482	13807	Tbca	10.18761702	9.982276567	10.11699285
8	0610010K14Rik	7.92643607	8.138210545	7.991196732	2308	Bud13	6.32803891	6.48568684	6.06681448	4608	Egln1	9.063613095	9.037267894	9.225619935	6908	Hnrnpl	8.63522815	8.739181369	8.69081672	9208	Nbr1	8.552821856	8.929057106	8.822403564	11508	Rbm11	6.365317066	6.716069874	6.42323397	13808	Tbcb	9.180807416	9.020236853	9.021646724
9	0610011F06Rik	6.912461144	6.743738527	6.438559601	2309	Bud31	8.045213467	7.910140384	7.659244643	4609	Egln2	8.531015475	8.361598214	8.4395163	6909	Hnrnpll	7.20038961	7.642626925	7.20860417	9209	Ncald	12.19041977	12.42428349	12.16980135	11509	Rbm12	5.910593779	5.99494908	5.594341108	13809	Tbcc	7.603422742	7.538826028	7.511396776
10	0610012G03Rik	7.999203096	8.184156045	7.761102142	2310	Bves	2.412078801	3.564780839	2.787057683	4610	Egln3	4.666187554	4.795025583	4.438176656	6910	Hnrnpm	9.973821612	10.03897767	9.88685478	9210	Ncam1	11.92241823	11.67898959	11.52878811	11510	Rbm12b1	4.841554039	5.120970365	5.228053968	13810	Tbccd1	5.469736542	5.331657849	4.885291215
11	0610030E20Rik	5.810795116	5.810703238	6.284616531	2311	Bvht	3.536186016	3.545561624	4.153495844	4611	Egr1	10.21115139	10.10667747	10.36048399	6911	Hnrnpr	8.838930958	8.906699539	8.94869209	9211	Ncam2	9.520854994	9.240789062	9.45785053	11511	Rbm12b2	6.305632895	5.87955727	5.979960821	13811	Tbcd	6.862847075	7.207655218	7.024484556
12	0610037L13Rik	6.999234949	7.3102722	7.109527035	2312	Bysl	8.051568911	8.038381926	7.880776421	4612	Egr2	3.777771813	2.074207652	3.888166078	6912	Hnrnpu	10.54117624	10.64507037	10.74652079	9212	Ncan	10.00664624	9.921468204	9.947241096	11512	Rbm14	5.606091921	5.769141318	5.83826707	13812	Tbce	6.037034558	6.23020825	6.088110857
13	0610038B21Rik	2.873588504	2.434788571	3.445022391	2313	Bzrap1	8.215720196	8.422971265	8.587484069	4613	Egr3	8.643052321	8.69566645	8.803414933	6913	Hnrnpul1	4.660757417	4.706282074	4.839838993	9213	Ncapd2	6.507782895	6.619482853	6.787762596	11513	Rbm15	4.403810549	4.940548315	4.301084758	13813	Tbcel	8.81198696	9.070207366	8.774348002
14	0610039K10Rik	0.01933481	0.910016002	0.989104339	2314	Bzw1	8.907466389	8.977071464	9.218497345	4614	Egr4	7.00892913	6.401326959	6.957733574	6914	Hnrnpul2	9.476974352	9.398219051	9.412541373	9214	Ncapd3	6.49048373	6.151608112	6.519085592	11514	Rbm15b	7.209300737	6.954005478	7.302207286	13814	Tbck	7.853824054	7.443445484	7.430955784
15	0610040B10Rik	4.786404673	5.551423477	5.228446322	2315	Bzw2	8.531250719	8.445538993	8.2836621	4615	Ehbp1	8.741875669	8.648696104	8.768019133	6915	Hoga1	3.073884356	3.448399813	2.981142251	9215	Ncapg	3.062583432	3.198408601	2.790652208	11515	Rbm17	8.897031717	8.747835743	8.673429561	13815	Tbk1	6.768967935	6.626139809	6.87456974
16	0610040F04Rik	2.88437922	2.408538818	4.446230462	2316	C030006K11Rik	5.605190894	5.696186098	6.151354932	4616	Ehbp1l1	6.503499771	6.380819782	6.456404781	6916	Homer1	9.791438092	9.804966189	10.11065326	9216	Ncapg2	4.830483444	4.479035935	3.559361632	11516	Rbm18	7.122950015	7.278423638	6.623269027	13816	Tbkbp1	8.016649164	8.101708801	8.024520675
17	0610040J01Rik	3.673994933	4.665659716	3.979555446	2317	C030018K13Rik	4.240427255	3.746058381	3.68028578	4617	Ehd1	8.156698851	8.142755536	7.714324623	6917	Homer2	10.2326826	10.20876568	10.16735659	9217	Ncaph2	8.691472287	8.70540397	8.673485213	11517	Rbm19	5.663803372	5.538068916	5.652787205	13817	Tbl1x	6.752446316	6.535312481	7.025081536
18	0610043K17Rik	5.254820779	4.814304052	5.471616868	2318	C030023E24Rik	3.663806022	3.415441889	3.888345559	4618	Ehd2	3.984623434	4.171701118	3.980640395	6918	Homer3	7.847320018	7.715632212	7.487612729	9218	Ncbp1	8.245491942	8.18157049	8.484034511	11518	Rbm20	3.254268585	3.757079082	3.681661976	13818	Tbl1xr1	9.135867132	9.224043131	9.346420091
19	1010001N08Rik	0.027264343	0.894795718	1.572875532	2319	C030029H02Rik	5.861483637	6.204103078	6.089303859	4619	Ehd3	10.37152822	10.31686027	9.891278208	6919	Homez	5.936236639	5.862731646	5.813043551	9219	Ncbp2	8.607997848	8.425411633	8.913919714	11519	Rbm22	7.068522092	7.028395451	6.799788006	13819	Tbl2	4.882760662	5.062469688	4.226151937
20	1110001J03Rik	9.455307593	9.244797425	9.269978671	2320	C030034I22Rik	6.367580171	6.258739362	6.813553556	4620	Ehd4	6.910478222	6.633043679	6.838531714	6920	Hook1	6.573112082	6.323389265	6.825883288	9220	Ncdn	12.60439482	12.6693157	12.41068061	11520	Rbm24	7.252381243	7.359237403	7.80691982	13820	Tbl3	5.780998632	6.093221621	5.761277686
21	1110002L01Rik	8.784605562	8.851962732	8.844180217	2321	C030037D09Rik	3.982894244	3.74402574	4.375080294	4621	Ehf	0.021972348	0.904828531	0.988490874	6921	Hook2	5.031945067	5.344070787	5.303607052	9221	Nceh1	10.02591564	9.854339075	10.11574559	11521	Rbm25	10.62058509	10.52785301	10.4637884	13821	Tbp	5.935104749	5.995474049	5.405268592
22	1110004E09Rik	9.071771758	9.023045051	9.007925246	2322	C030039L03Rik	5.214946443	5.798824891	5.503418425	4622	Ehhadh	1.669315995	2.870852696	2.984606123	6922	Hook3	9.822122641	9.813478064	10.01207665	9222	Ncf1	5.832829957	5.796025143	5.935130204	11522	Rbm26	8.647014864	8.692226167	8.841121476	13822	Tbpl1	8.871434971	8.981060611	8.659377187
23	1110004F10Rik	10.00815806	9.947833872	9.961579592	2323	C130026L21Rik	3.863432654	3.0303517	2.982668486	4623	Ehmt1	8.01646812	7.968027537	7.880862951	6923	Hopx	8.332970546	8.446470355	7.892461554	9223	Ncf2	2.676104743	3.554077318	3.149972127	11523	Rbm27	9.048359211	8.676595368	8.609231978	13823	Tbr1	8.494068069	8.785361097	8.74177182
24	1110007C09Rik	5.122330835	5.089658727	5.19033099	2324	C130030K03Rik	5.213636589	5.650544111	5.681215306	4624	Ehmt2	8.19392905	8.390818525	8.189523747	6924	Hoxa3	0.016582233	1.921635936	0.98976491	9224	Nck1	7.473641068	7.473018199	7.659557068	11524	Rbm28	8.658092228	8.751085511	8.771210469	13824	Tbrg1	7.905110251	7.579554141	7.549829897
25	1110008F13Rik	8.438166162	8.507090699	8.380799158	2325	C130036L24Rik	3.409396343	4.018713566	4.684478695	4625	Ei24	8.26437489	8.17446307	8.024372316	6925	Hoxaas2	0.016582233	1.921635936	0.98976491	9225	Nck2	7.091136464	7.309736553	6.850021532	11525	Rbm3	6.420613854	6.901427114	6.389244355	13825	Tbrg3	4.324835631	3.825008439	4.228349693
26	1110008L16Rik	9.234832291	8.953854737	9.204100374	2326	C130046K22Rik	4.719665293	3.713043061	4.839598262	4626	Eid1	11.52454287	11.52301208	11.56213118	6926	Hoxc9	0.005608334	0.940318714	0.992698372	9226	Nckap1	10.82202731	10.89525704	10.65325975	11526	Rbm33	7.75972465	7.867421989	7.881086923	13826	Tbrg4	6.584471115	6.282072677	6.504291563
27	1110008P14Rik	9.988726019	9.972635716	9.965873653	2327	C130050O18Rik	1.040695909	0.915564801	0.989761512	4627	Eid2	8.919830671	9.119473111	8.971525523	6927	Hp	4.470960698	3.097991785	3.977985746	9227	Nckap1l	4.669114606	4.63704673	5.110248736	11527	Rbm34	7.375165459	7.320969747	7.857219155	13827	Tbx15	2.649206974	2.175312017	1.564331221
28	1110012L19Rik	5.718102299	5.192670213	4.785223352	2328	C130060K24Rik	3.23441912	1.812770347	2.789368484	4628	Eid2b	6.270715392	6.362673387	6.355170862	6928	Hp1bp3	10.92284678	10.72028599	10.8410719	9228	Nckap5	5.690805906	5.80327573	5.788732984	11528	Rbm38	2.411420517	2.404039455	2.980838233	13828	Tbx18	4.169879854	3.910869222	4.566293021
29	1110015O18Rik	2.066969598	2.188700292	1.566345711	2329	C130071C03Rik	4.838086383	4.685221094	5.5365889	4629	Eif1	10.69528381	10.66589457	10.69680182	6929	Hpca	13.09248156	13.17938918	12.53694347	9229	Nckap5l	4.251828172	4.546100446	4.789341403	11529	Rbm39	10.38190462	10.19525776	10.15862288	13829	Tbx2	1.077144622	2.193522216	2.306615668
30	1110017D15Rik	5.208026763	6.16883348	5.266043431	2330	C130074G19Rik	6.489582324	6.18980901	7.181237499	4630	Eif1a	8.374429052	8.390095969	8.007687413	6930	Hpcal1	8.010131052	7.866541286	7.673319247	9230	Nckipsd	7.299528328	7.149011464	7.346434937	11530	Rbm4	7.144880178	6.859089008	6.747785797	13830	Tbx21	0.024613799	2.72877025	1.573377654
31	1110019D14Rik	3.254754127	3.6528233	4.443684766	2331	C130080G10Rik	2.070409968	2.181812778	2.569287462	4631	Eif1ad	7.539144059	7.504333384	7.283557259	6931	Hpcal4	10.92564827	10.68052363	10.37012324	9231	Ncl	12.1614042	12.06443569	12.09761693	11531	Rbm41	5.781878167	5.612961549	5.707440543	13831	Tbx3	3.41448374	4.091145437	4.790896274
32	1110020A21Rik	2.398277536	2.86165878	1.978866051	2332	C130083M11Rik	1.6346526	0.889892566	1.988213603	4632	Eif1ax	8.135547918	8.303270863	7.962853718	6932	Hpdl	3.246712045	3.667322892	2.562027969	9232	Ncln	6.541682168	6.950292202	6.946267779	11532	Rbm42	8.698399914	8.56934966	8.463735778	13832	Tbx6	2.371509885	1.464723341	2.309358706
33	1110028F11Rik	0.035156703	1.894883254	0.985634158	2333	C1d	8.485057359	8.14480043	8.350702673	4633	Eif1b	10.48879107	10.36426676	10.39043519	6933	Hpgd	4.78285474	4.835999739	4.224661307	9233	Ncmap	3.085692544	2.814498452	4.57013832	11533	Rbm43	5.75251979	5.561434553	5.438413273	13833	Tbxa2r	2.068177636	2.686420195	1.982625321
34	1110032A03Rik	8.249964647	8.159997049	8.25624134	2334	C1galt1	5.157608493	4.752426052	4.43901967	4634	Eif2a	8.102137722	7.836846207	8.119746538	6934	Hpgds	5.292668786	5.650244812	5.788440115	9234	Ncoa1	9.194108807	9.177969409	9.080626922	11534	Rbm45	5.513797368	6.217517972	6.088740523	13834	Tbxas1	1.690263632	3.019440301	2.564148426
35	1110032F04Rik	5.88825202	5.989353564	6.48937316	2335	C1galt1c1	6.574137064	6.630034006	6.934568617	4635	Eif2ak1	8.402237671	8.26774713	8.337536204	6935	Hpn	1.088527264	1.413531194	2.792050065	9235	Ncoa2	6.711678356	7.019489502	7.057100125	11535	Rbm46	4.159957149	3.84157852	3.680110744	13835	Tc2n	2.051263415	2.220019788	0.985105203
36	1110034G24Rik	6.183222207	5.53325436	5.680304796	2336	C1qa	8.31857731	8.168283149	8.114294002	4636	Eif2ak2	4.402414144	4.706243451	4.372168899	6936	Hprt	9.845339883	9.9190331	9.793768797	9236	Ncoa3	6.456781323	6.735604785	6.826055163	11536	Rbm47	6.007823761	6.638592628	4.220620793	13836	Tcaf1	7.166287695	6.777499366	7.088782473
37	1110037F02Rik	7.968477038	7.643437022	7.734772905	2337	C1qb	8.322580921	8.178674073	8.056794824	4637	Eif2ak3	5.786635301	6.230163405	6.265488996	6937	Hps1	2.897197188	3.948742918	3.889610893	9237	Ncoa4	6.999356071	6.934971116	7.170152098	11537	Rbm48	7.454871885	7.483394355	7.609754974	13837	Tcaf2	2.661045439	2.852893928	3.153171236
38	1110038B12Rik	5.939522287	6.199495512	5.979871013	2338	C1qbp	8.73863232	8.757062145	8.645282145	4638	Eif2ak4	6.20717653	5.60939821	6.302817096	6938	Hps3	5.363847046	5.230136788	4.785438073	9238	Ncoa5	7.481274346	7.391617436	7.422876086	11538	Rbm4b	6.369771245	6.862138671	7.099655207	13838	Tcaim	7.230492123	7.394236588	7.35519425
39	1110038F14Rik	7.779379915	7.620481205	7.557487465	2339	C1qc	8.040119667	8.018368351	7.844040886	4639	Eif2b1	6.924201449	6.732899708	6.707882416	6939	Hps4	4.829681389	4.419646749	4.371685802	9239	Ncoa6	8.182890834	8.236666411	7.837793295	11539	Rbm5	8.935103051	8.637656745	8.620077507	13839	Tcap	3.079635194	2.391989348	3.441369165
40	1110046J04Rik	4.157730673	3.534692462	3.56468149	2340	C1ql1	6.247051258	6.029261816	5.886643569	4640	Eif2b2	8.125433499	8.454188642	8.40221404	6940	Hps5	4.885994796	5.532277561	4.67999423	9240	Ncoa7	9.841895243	9.635249303	9.707904417	11540	Rbm6	8.397779332	8.329542645	8.406304495	13840	Tcea1	7.822089101	7.893701409	8.088819367
41	1110051M20Rik	8.411047821	8.144921971	8.232580164	2341	C1ql2	9.080220481	9.057521723	8.283246674	4641	Eif2b3	6.366289752	6.541093187	6.47203062	6941	Hps6	5.204374884	4.053174327	4.734280713	9241	Ncor1	11.58683219	11.49512977	11.43427413	11541	Rbm7	7.547280821	7.453057266	7.542433346	13841	Tcea2	6.554452024	6.541475734	6.001275152
42	1110059E24Rik	7.70112491	7.391558104	7.557562912	2342	C1ql3	8.119377908	7.991721449	7.916414603	4642	Eif2b4	6.977250002	7.203967509	7.237409243	6942	Hpse	1.649444296	2.473237514	2.307781469	9242	Ncor2	8.485098621	8.500842187	8.241993653	11542	Rbm8a	9.927552523	9.837698832	9.841056208	13842	Tcea3	3.244135078	4.045913191	2.980203619
43	1110059G10Rik	7.61940544	7.671887934	7.659598515	2343	C1qtnf1	4.832097229	4.890369269	4.935405239	4643	Eif2b5	7.999155301	8.043300091	8.046193734	6943	Hpx	5.988364745	3.418353629	5.733822132	9243	Ncs1	10.39101743	10.26516378	10.27805397	11543	Rbms1	6.015769959	6.751381422	6.72224194	13843	Tceal1	7.780997602	7.707430229	7.910889941
44	1110065P20Rik	8.971572151	8.744896639	8.714694455	2344	C1qtnf2	3.865364828	2.648099914	2.788467558	4644	Eif2d	6.030683746	5.817679097	5.934880367	6944	Hr	5.918071422	6.246138604	6.456137779	9244	Ncstn	4.994786742	5.453025887	5.653174827	11544	Rbms2	6.229482969	6.214062488	6.456516351	13844	Tceal3	10.81513235	10.62731067	10.65240907
45	1190002F15Rik	2.64826223	3.052440219	2.568844349	2345	C1qtnf4	8.021744325	7.695049884	7.767888396	4645	Eif2s1	8.313637443	8.23050758	8.169792802	6945	Hras	9.209680662	9.208817732	9.15241878	9245	Ndc1	3.868392995	3.779913655	3.568582207	11545	Rbms3	8.059290141	8.31287005	8.748460891	13845	Tceal5	9.437580308	9.247428836	9.04058745
46	1190002N15Rik	6.796656973	6.955889123	7.311454532	2346	C1qtnf5	5.984721063	6.292696525	5.021429808	4646	Eif2s2	10.96772329	10.79406118	10.84879945	6946	Hrasls	6.458218443	6.950169462	6.911132889	9246	Nde1	8.473521065	8.878021875	8.269665042	11546	Rbmx	7.385617673	7.537718531	7.609377013	13846	Tceal6	10.15450648	9.942249822	10.1310413
47	1190005I06Rik	5.907106519	5.452779355	5.735878467	2347	C1qtnf6	2.398300725	3.465260859	2.983530944	4647	Eif2s3x	7.681492197	7.876833926	8.057029147	6947	Hrasls5	1.650926493	3.720171856	1.568178126	9247	Ndel1	9.292468404	9.244864156	9.479922458	11547	Rbmx2	5.811112841	5.786383616	6.170270268	13847	Tceal7	2.884243273	3.679279491	1.557832917
48	1190007I07Rik	6.487582006	6.314516836	6.550567402	2348	C1qtnf7	2.869842124	3.204139524	1.563722826	4648	Eif2s3y	5.935799235	6.014769267	5.911309548	6948	Hrct1	2.645619467	1.422264849	2.792702101	9248	Ndfip1	6.710185513	6.385731315	7.035890967	11548	Rbmxl1	6.846999128	6.521773065	6.774664745	13848	Tceal8	8.611272335	8.603901489	8.292741631

49	1200014J11Rik	7.069882514	7.065380615	6.979655053	2349	C1ra	3.784823365	4.167038338	3.786400387	4649	Eif3a	11.54151517	11.46485995	11.30694912	6949	Hrg	3.087088721	0.648384755	4.153385915	9249	Ndfip2	10.37311631	10.48956828	10.71810218	11549	Rbp1	7.262151208	7.672555727	7.455630833	13849	Tceanc	3.254586444	4.186097408	2.783443938
50	1300002E11Rik	5.333517361	6.016432389	5.438602541	2350	C1rb	3.079092379	3.165461022	3.15059809	4650	Eif3b	7.807354541	7.572083549	7.694347844	6950	Hrh1	5.560868314	4.885016504	5.267219034	9250	Ndn	10.21613338	10.02868925	9.923903885	11550	Rbp4	6.692374972	6.135812539	6.227432262	13850	Tceanc2	6.572677136	6.909295254	6.748513556
51	1300017J02Rik	2.070326134	1.849069884	2.305275351	2351	C1rl	2.862309769	2.888272849	1.566706963	4651	Eif3c	10.56629228	10.36216035	10.35173156	6951	Hrh3	8.02133477	7.689503861	8.067922453	9251	Ndnf	6.270887436	6.22825574	6.30187499	11551	Rbpj	6.961784982	6.588967129	6.922761651	13851	Tceb1	8.795238554	8.974969087	8.960134002
52	1500004A13Rik	6.571560131	6.242139367	6.31939614	2352	C1s1	1.062704149	2.48313082	0.984103092	4652	Eif3d	8.607584649	8.600177211	8.435271828	6952	Hrk	9.948736986	9.776618005	9.211030839	9252	Ndnl2	6.810699686	7.389096916	7.013290203	11552	Rbpms	5.285092306	4.633047362	5.024569915	13852	Tceb2	10.68318629	10.58850999	10.49565957
53	1500009C09Rik	8.897695647	8.943697237	8.988437812	2353	C2	2.113516712	3.425703679	4.740506883	4653	Eif3e	9.328218791	9.175299395	9.310957555	6953	Hrsp12	8.205129956	8.284698793	8.213505616	9253	Ndor1	4.156974812	3.284636334	3.888402892	11553	Rbpms2	4.837767841	5.202506262	4.93402641	13853	Tceb3	7.666197413	7.756386509	7.868835604
54	1500009L16Rik	6.664842556	6.807374809	6.301160935	2354	C230004F18Rik	3.780816961	4.097678936	3.980982137	4654	Eif3f	9.250284381	9.21961492	9.174747548	6954	Hs1bp3	5.07520586	4.530062482	5.110699005	9254	Ndp	5.398585539	5.160087581	5.338263149	11554	Rbsn	7.874694623	7.785833636	7.793729952	13854	Tcerg1	9.483566385	9.512139405	9.50355812
55	1500011B03Rik	8.539433853	8.584002324	8.337487166	2355	C230014O12Rik	1.080546231	0.82695665	2.570017142	4655	Eif3g	8.567756465	8.1982962	8.538535528	6955	Hs2st1	6.877533703	7.46341413	7.456161826	9255	Ndrg1	9.422501571	9.519684733	9.348486291	11555	Rbx1	9.774105764	9.883806013	9.803349836	13855	Tcerg1l	5.434120536	5.121499132	5.189494252
56	1500011K16Rik	8.332969886	8.203625716	8.423076173	2356	C230035I16Rik	1.681331809	3.187982678	3.305970383	4656	Eif3h	9.908622764	9.816415493	9.819173685	6956	Hs3st1	5.915364976	6.399577118	6.373152585	9256	Ndrg2	12.46780908	12.25444619	12.41668413	11556	Rc3h1	8.234957158	8.132394675	8.114384682	13856	Tcf12	8.735011107	8.505631246	8.771220136
57	1500012F01Rik	8.599873475	8.623217332	8.491723185	2357	C230037L18Rik	3.671375307	3.924257898	4.683590569	4657	Eif3i	9.424505862	9.210467386	9.2063655	6957	Hs3st2	5.866287831	6.5307705	6.876411836	9257	Ndrg3	10.26174193	10.32977814	10.42192892	11557	Rc3h2	9.604138856	9.557029022	9.739927162	13857	Tcf15	3.652432158	3.959981848	2.7863418
58	1500012K07Rik	1.064655938	1.88205879	1.569745136	2358	C230072F16Rik	3.977489249	3.94310533	3.301050221	4658	Eif3j2	7.579928958	7.545680252	7.638397213	6958	Hs3st3a1	3.673758657	4.306361981	4.505563043	9258	Ndrg4	11.58092293	11.56868122	11.5089146	11558	Rcan1	7.981356295	8.067556416	8.078146456	13858	Tcf19	3.676518404	5.035189673	3.978981185
59	1500015A07Rik	4.328141308	4.759840967	4.736709642	2359	C230091D08Rik	8.507278645	8.635470386	8.707960187	4659	Eif3k	9.146446401	9.162463317	8.898741328	6959	Hs3st3b1	3.553633828	3.726501127	3.785378732	9259	Ndst1	8.726922164	8.662839307	8.422932535	11559	Rcan2	9.464710743	9.525466538	9.919645536	13859	Tcf20	8.285867009	8.492572603	8.731742329
60	1500015L24Rik	2.394048119	2.16683797	1.563049712	2360	C2cd2	6.187813215	5.736114847	6.067405974	4660	Eif3l	8.228715206	7.983310347	7.714459921	6960	Hs3st4	10.86245229	10.77412358	10.47081716	9260	Ndst2	5.169484496	5.040253391	5.709477483	11560	Rcan3	6.900922231	6.666368856	7.035494533	13860	Tcf25	12.12088405	12.07195685	12.10215652
61	1500015O10Rik	7.641466199	8.535191553	6.404154537	2361	C2cd2l	9.091414799	9.074457075	8.910695052	4661	Eif3m	8.208570021	7.967369935	7.80636721	6961	Hs3st5	3.907885056	4.971752868	5.506073959	9261	Ndst3	6.861664317	6.286922404	6.825458912	11561	Rcbtb1	8.080337507	8.133322039	8.072787425	13861	Tcf3	6.07902069	6.41777914	6.024588904
62	1500017E21Rik	5.090448243	5.994648655	5.680330677	2362	C2cd3	6.419385185	6.393813289	6.456058798	4662	Eif4a1	9.279595985	9.173933113	8.95722231	6962	Hs3st6	0.027292386	0.894743702	1.988643456	9262	Ndst4	5.540964727	5.447983996	5.623894338	11562	Rcbtb2	6.694291157	6.697698171	6.638302563	13862	Tcf4	13.20694147	13.01238297	12.84047423
63	1500026H17Rik	4.719676374	5.106376522	4.736086343	2363	C2cd4a	1.695186417	2.836055523	3.304040069	4663	Eif4a2	10.87340493	10.85381489	10.92542578	6963	Hs6st1	7.702672994	7.757153418	7.905049832	9263	Ndufa1	9.918185269	9.839022947	9.769464582	11563	Rcc1	6.360764777	6.254852213	5.886774558	13863	Tcf7	1.62878847	0.904825457	1.573892473
64	1600002H07Rik	4.932520166	4.592932363	4.888160686	2364	C2cd4b	4.608495212	5.311380497	5.151130476	4664	Eif4a3	8.451493883	8.576301249	8.487752698	6964	Hs6st2	8.092991372	7.98953211	8.694503838	9264	Ndufa10	10.8167668	10.54854438	10.49566041	11564	Rcc2	8.828679623	8.998749357	8.92844022	13864	Tcf7l1	5.639328953	6.029669056	5.980095908
65	1600002K03Rik	4.532517607	3.916753432	4.790158672	2365	C2cd4c	4.664022199	5.021896381	4.839058	4665	Eif4b	9.061064691	9.291444857	9.315432877	6965	Hsbp1	10.98657636	10.81365401	10.67779054	9265	Ndufa11	9.675079791	9.853427857	9.731505385	11565	Rccd1	1.05881054	0.876047484	1.571010436	13865	Tcf7l2	7.805787392	8.016814556	9.20898059
66	1600010M07Rik	0.008386612	0.933563535	0.991897072	2366	C2cd4d	1.65652324	3.218260473	1.566724069	4666	Eif4e	9.413399645	9.274566849	9.272308727	6966	Hsbp1l1	3.539797423	3.538165115	3.982102053	9266	Ndufa12	10.52344851	10.31528281	10.49864568	11566	Rce1	5.829414048	4.909503709	5.624645187	13866	Tcfl5	3.544665145	4.87375317	3.437654092
67	1600012H06Rik	8.098244334	8.647614422	8.283746993	2367	C2cd5	6.526051693	6.827125476	6.99163373	4667	Eif4e2	8.406666625	8.2720569	8.439525048	6967	Hscb	4.838231693	5.272426741	5.06773128	9267	Ndufa13	10.66183477	10.791374	10.64441109	11567	Rchy1	7.861935079	7.978465074	7.825424999	13867	Tchh	4.671778672	5.126656981	5.536489073
68	1600014C10Rik	6.590709991	6.958659675	6.837957867	2368	C3	4.776536149	3.491849919	4.371571452	4668	Eif4e3	7.33628638	7.396313165	6.747424364	6968	Hsd11b1	6.215222088	6.971174753	6.579554876	9268	Ndufa2	10.95701839	11.01793568	10.75885988	11568	Rcl1	7.189801723	7.513111384	7.355137958	13868	Tchp	5.505523011	5.422860853	5.372127519
69	1600020E01Rik	5.563671774	4.736171218	4.979967692	2369	C330006A16Rik	9.444711634	9.359074166	9.493732404	4669	Eif4ebp1	4.334664615	4.475195911	4.887875156	6969	Hsd17b10	7.93197933	8.024178151	7.637890892	9269	Ndufa3	10.32366978	10.27037816	10.20396833	11569	Rcn1	6.695745895	6.862145576	7.08921947	13869	Tcirg1	4.320290075	4.243123835	3.679345342
70	1600029I14Rik	2.650003749	2.173357573	2.985002439	2370	C330007P06Rik	7.842428286	7.92550356	8.051624824	4670	Eif4ebp2	8.214539491	8.09980274	8.265547836	6970	Hsd17b11	8.950703042	8.851738735	8.819151353	9270	Ndufa4	9.774055448	9.768537564	9.762977191	11570	Rcn2	8.285240324	8.325775132	8.306409334	13870	Tcn2	6.036264913	6.381677756	5.185932526
71	1600029O15Rik	4.311401715	3.946953643	3.565995683	2371	C330013E15Rik	3.389949626	3.318747662	3.151911004	4671	Eif4ebp3	5.436356381	4.997371	5.472389411	6971	Hsd17b12	8.787731687	8.866530415	8.784158774	9271	Ndufa4l2	3.781075817	3.527107496	3.146221477	11571	Rcn3	5.802276577	5.571614131	5.06626787	13871	Tcof1	7.504940657	7.102780729	7.472240909
72	1700001C02Rik	4.302358723	3.319673397	2.299590958	2372	C330013J21Rik	1.064707216	0.862937454	1.985886503	4672	Eif4enif1	7.594396296	7.362030048	7.389504023	6972	Hsd17b2	0.078893865	0.808785092	2.985401148	9272	Ndufa5	11.21108563	11.10808549	10.91216138	11572	Rcor1	6.708402203	6.509404876	6.694564276	13872	Tcp1	9.883014253	9.771311909	9.865690254
73	1700001C19Rik	1.038055615	1.507502904	0.990436694	2373	C330018D20Rik	5.961986413	5.969557836	5.910856881	4673	Eif4g1	10.002359	9.96839104	10.06216461	6973	Hsd17b4	7.261606444	7.328434066	7.774917598	9273	Ndufa6	10.26175943	10.14125482	10.22075366	11573	Rcor2	5.752337743	5.817960823	5.623558292	13873	Tcp10a	1.07027208	2.473082325	1.568537197
74	1700001J11Rik	0.008382678	0.933572806	0.991898172	2374	C330021F23Rik	10.75982526	10.44592376	10.37226295	4674	Eif4g2	11.06789012	11.09277036	11.14048301	6974	Hsd17b7	7.114324996	7.186951004	7.109175772	9274	Ndufa7	9.794717152	9.819639098	9.676926722	11574	Rcor3	6.619458196	6.522989032	6.735201761	13874	Tcp10c	0.035146772	2.223054124	1.987395727
75	1700001K19Rik	1.638225878	2.913732642	1.571462345	2375	C330022C24Rik	1.632792088	0.894664264	0.987292015	4675	Eif4g3	10.56849585	10.70594142	10.73738958	6975	Hsd3b3	1.052524248	1.901872155	0.986728868	9275	Ndufa8	9.433596888	9.298986141	9.453727434	11575	Rcsd1	4.170292556	4.427809406	3.97842719	13875	Tcp11l1	4.254851199	4.069598972	4.839509766
76	1700001L05Rik	3.655027823	3.864783049	2.785471764	2376	C330027C09Rik	3.264176636	4.379951968	4.683952102	4676	Eif4h	9.590430771	9.711497844	9.692533079	6976	Hsd3b4	0.019314373	0.910056763	1.574428035	9276	Ndufa9	8.591563455	8.345303867	8.265268363	11576	Rd3	1.680139059	4.604005878	2.301617992	13876	Tcp11l2	5.839617614	6.300105669	6.228082049
77	1700001L19Rik	6.342464304	6.355105915	6.06787002	2377	C3ar1	4.934842587	4.531359472	5.06886311	4677	Eif5	10.46222272	10.60605798	10.6166177	6977	Hsd3b7	5.564448806	5.006769273	5.338698383	9277	Ndufab1	5.88438653	5.218120052	5.59459416	11577	Rd3l	1.091692768	2.675242739	2.984971568	13877	Tcta	5.936474741	5.862259667	5.623309545
78	1700001O22Rik	3.989300247	4.816414388	4.626220997	2378	C430049B03Rik	3.071634834	3.180366397	3.443150049	4678	Eif5a	10.67580888	10.72591439	10.63461442	6978	Hsdl1	7.189016716	6.877649539	7.54283589	9278	Ndufaf1	6.779108685	6.187310414	6.875467099	11578	Rdh10	5.128278412	5.680226819	5.301793433	13878	Tcte1	9.736521636	9.531478344	9.544161261
79	1700003D09Rik	3.897418599	4.545207306	4.565930469	2379	C4a	3.249953652	3.430507028	3.149479602	4679	Eif5a2	6.254492624	6.463985883	6.800546772	6979	Hsdl2	7.507553831	7.405086489	7.774607579	9279	Ndufaf2	7.626687876	7.319356155	7.565041365	11579	Rdh11	4.828800472	4.421604184	4.439361535	13879	Tcte2	2.87719037	3.190415107	3.153655727
80	1700003E16Rik	6.225931283	6.404352438	6.24789002	2380	C4b	5.572299804	5.41858018	5.438958645	4680	Eif5b	11.23726615	11.19865142	11.12908287	6980	Hsf1	6.206409856	6.165331288	6.089006875	9280	Ndufaf3	7.969274013	8.096413601	7.892788875	11580	Rdh12	4.331842541	4.895077281	4.788558309	13880	Tcte3	2.661066826	3.029082336	2.982512018
81	1700003F12Rik	1.704944322	5.425478987	3.14991905	2381	C4bp-ps1	1.045682117	0.90477886	0.988485005	4681	Eif6	7.580728099	7.793736968	7.80039364	6981	Hsf2	7.532404231	7.63875383	7.825735051	9281	Ndufaf4	8.584500718	8.513111116	8.793903875	11581	Rdh13	6.36285753	5.808161267	6.407114473	13881	Tctex1d1	1.657984595	1.428279781	2.986413905
82	1700003M02Rik	2.638712055	2.896020628	0.981687871	2382	C530005A16Rik	3.42131662	3.820981385	4.440385567	4682	Elac1	6.57315259	6.605208407	6.800507229	6982	Hsf2bp	3.380597226	0.780136621	3.570944276	9282	Ndufaf5	7.437576936	7.345452726	7.363698008	11582	Rdh14	7.595664682	7.40653538	7.511383201	13882	Tctex1d2	6.327485785	6.486757916	6.423420366
83	1700003M07Rik	7.513675429	7.378130425	7.389542282	2383	C530008M17Rik	7.410611052	7.469539888	7.372448478	4683	Elac2	7.047708824	7.306525882	7.179574634	6983	Hsf4	4.177155784	4.645670489	4.887970045	9283	Ndufaf6	5.605221956	5.696125548	5.652305052	11583	Rdh5	4.946372435	6.462289786	5.762552278	13883	Tctex1d4	5.119354874	4.408946006	4.838202309
84	1700007B14Rik	1.073760418	2.468500802	2.987309277	2384	C530044C16Rik	0.019335859	0.910013909	0.989104091	4684	Elavl1	7.867672967	7.833313659	7.806644641	6984	Hsf5	1.095914438	2.440060433	3.307516257	9284	Ndufaf7	6.794499964	6.744420261	6.862848648	11584	Rdh9	2.052749201	1.464744041	2.795966632	13884	Tctn1	4.473232542	4.939900218	4.565269095
85	1700007F19Rik	3.991789111	5.428931412	4.302371571	2385	C5ar2	2.392140692	3.04761316	1.563699297	4685	Elavl2	11.20138196	11.01076289	11.04539153	6985	Hsfy2	0.016587399	1.921627602	1.574997457	9285	Ndufb10	9.777421682	9.813988463	9.636377846	11585	Rdm1	5.286626315	5.269982817	5.109406323	13885	Tctn2	5.690178426	5.455906518	5.565766857
86	1700007G11Rik	2.086082755	3.977233509	2.982898361	2386	C630031E19Rik	3.089220384	3.420314771	3.565412129	4686	Elavl3	11.65745967	11.49465869	11.50108989	6986	Hsp90aa1	13.63600245	13.41977711	13.26305792	9286	Ndufb11	9.823359129	9.580853537	9.51715833	11586	Rdx	10.04629557	10.13276121	9.804871228	13886	Tctn3	4.875112486	4.433630103	4.067142943
87	1700007J10Rik	6.148009671	6.175712646	6.610221153	2387	C630043F03Rik	6.012005348	6.105419717	6.209204985	4687	Elavl4	11.08884336	10.80275833	11.07145264	6987	Hsp90ab1	14.01841755	13.8782234	13.6405231	9287	Ndufb2	9.116534278	9.13520626	9.075405125	11587	Rec114	10.00241723	10.14079187	10.10801065	13887	Tdg	3.655702841	3.664134336	3.302646146
88	1700007K13Rik	4.711153836	4.310087661	2.779882393	2388	C730002L08Rik	2.072546097	2.67903701	3.687996701	4688	Elf1	5.808544071	5.970942556	5.651864491	6988	Hsp90b1	11.68838036	11.69576879	11.62202305	9288	Ndufb3	10.70071442	10.41487093	10.52008824	11588	Rec8	1.664539007	2.177312621	1.564633051	13888	Tdgf1	0.069742001	2.457598339	1.982642472
89	1700007P06Rik	2.86984878	2.872609702	1.980677786	2389	C77080	4.8923399	5.431019407	5.596309958	4689	Elf2	7.147562189	7.01406667	7.218297324	6989	Hspa12a	9.734778963	9.401586401	9.397784831	9289	Ndufb4	9.668979229	9.527890983	9.561209046	11589	Reck	3.532435329	3.664093889	3.566929636	13889	Tdh	0.024651824	1.909326982	1.573370351
90	1700008J07Rik	4.525704928	4.729063007	4.069079545	2390	C77370	9.947541286	9.676777497	9.683858347	4690	Elf4	2.082620495	2.861632532	2.566700066	6990	Hspa12b	2.648305421	2.177520863	1.981488717	9290	Ndufb5	10.39235608	10.37005523	10.40624228	11590	Recql	7.366079849	7.415663088	7.46393043	13890	Tdo2	5.166547351	5.234926872	4.786084608
91	1700008K24Rik	0.037685527	3.084264298	1.987010466	2391	C86187	1.641654508	1.464809416	1.986625421	4691	Elf5	0.04289448	1.461068556	1.570141259	6991	Hspa13	8.091239578	7.997891069	8.306777951	9291	Ndufb6	8.63902408	8.638648648	8.601996794	11591	Recql4	3.397492199	3.433349996	2.561461022	13891	Tdp1	5.437957817	5.293208919	5.264762857
92	1700008O03Rik	9.360585214	9.322875011	9.317763031	2392	C87436	5.335100242	5.743869039	5.838168877	4692	Elfn1	7.443557902	7.143781315	6.991198247	6992	Hspa14	7.547577036	7.772177864	7.397744881	9292	Ndufb7	8.996084189	8.972799799	8.812834925	11592	Recql5	5.118440805	4.841604681	4.887555281	13892	Tdp2	5.632693736	5.325595772	5.863964136
93	1700010I14Rik	2.392147298	2.171114521	2.791430166	2393	C8b	2.055563047	1.457627752	2.308706318	4693	Elfn2	8.259455078	8.086201723	8.184721685	6993	Hspa1a	5.663268828	5.185678258	5.371804203	9293	Ndufb8	11.12288469	11.01265123	11.0813022	11593	Reep1	8.451150163	8.521490964	8.471880411	13893	Tdrd1	1.08691004	3.210094372	1.981773324
94	1700010K23Rik	8.498771554	8.754652798	8.884085255	2394	C8g	3.232569474	3.193046735	1.978631588	4694	Elk1	8.102437847	8.091313031	7.727839957	6994	Hspa1b	5.660484022	4.912192643	5.472418062	9294	Ndufb9	11.01716248	10.83057341	10.8356079	11594	Reep2	7.53984258	7.421644931	7.616759845	13894	Tdrd3	6.807133565	6.7339056	6.969335292
95	1700011E24Rik	0.008386612	0.933563535	0.991897072	2395	C920006O11Rik	3.559067725	4.599983839	4.624882059	4695	Elk3	4.609309176	5.309960591	5.567431889	6995	Hspa1l	5.326564976	6.184341781	5.023640296	9295	Ndufc1	10.24538736	10.1449488	10.16237408	11595	Reep3	8.141240297	8.290276562	8.104069867	13895	Tdrd5	1.690437687	2.641394226	3.79205554
96	1700011I03Rik	0.05030081	2.475589973	3.448565035	2396	C920009B18Rik	0.005608334	0.940318714	0.992698372	4696	Elk4	6.206300883	6.050476624	6.002621413	6996	Hspa2	8.583925683	8.643202784	8.20374659	9296	Ndufc2	10.71535696	10.68015106	10.70450031	11596	Reep4	3.973113074	3.157348264	3.14960574	13896	Tdrd6	2.391084877	4.15373889	1.564057725
97	1700012B09Rik	3.964821826	3.321802937	2.788013942	2397	C920021L13Rik	2.877903124	2.855818912	3.444122298	4697	Ell	6.169410201	6.263518465	6.775590238	6997	Hspa4	10.76087545	10.54750035	10.65328213	9297	Ndufs1	9.863401622	9.641572705	9.83019718	11597	Reep5	11.25038911	11.18333528	10.95938593	13897	Tdrd7	6.606048232	7.2711289	6.734704779
98	1700012D01Rik	4.397121581	3.825959493	4.302794268	2398	Caap1	6.101050841	6.089000722	6.32089525	4698	Ell2	7.070257203	7.485396905	7.406600721	6998	Hspa4l	8.724774838	8.741141379	8.928411854	9298	Ndufs2	9.250503633	9.2407685	9.286061936	11598	Reep6	5.60237171	5.649710595	5.471798495	13898	Tdrd9	1.04568072	0.904781889	1.989543431

99	1700012D14Rik	1.641616496	0.871728342	0.984605843	2399	Cab39	9.217936447	9.417233293	9.499672758	4699	Ell3	3.669598841	4.379724603	3.679466315	6999	Hspa5	11.66782888	11.42028751	11.32313793	9299	Ndufs3	8.455701999	8.740355289	8.471828089	11599	Rel	2.102744421	3.559088995	3.1506683	13899	Tdrkh	5.573640824	5.722089516	5.623717118
100	1700012P22Rik	0.005608334	0.940318714	0.992698372	2400	Cab39l	7.564235903	8.037791871	7.49561468	4700	Elmo1	7.251880875	7.080759888	7.969394095	7000	Hspa8	11.82954312	11.5800207	11.44194407	9300	Ndufs4	9.751046783	9.553459883	9.639975668	11600	Rela	6.819332186	6.495222733	6.826140589	13900	Tdrp	5.687050751	4.827860392	5.109205507
101	1700013F07Rik	1.082057354	3.711451863	1.566023293	2401	Cabin1	8.223853286	8.262762887	8.05158527	4701	Elmo2	8.236181946	8.61710189	8.36361925	7001	Hspa9	9.940162748	9.906393091	10.06481982	9301	Ndufs5	9.116414606	9.010339474	9.056840743	11601	Relb	5.880036081	5.078382943	5.227583795	13901	Tead1	7.316494312	7.198386563	6.773938708
102	1700013G24Rik	0.050211184	3.824553348	0.982650202	2402	Cables1	6.035795203	5.89860093	6.189634528	4702	Elmo3	1.652433092	1.867391113	2.57104345	7002	Hspb1	7.239915721	6.932288931	7.002032855	9302	Ndufs6	9.874754345	9.689428015	9.690772471	11602	Rell1	7.809470807	8.399524385	8.368393985	13902	Tead2	3.974928409	3.948419032	3.789606629
103	1700015G11Rik	0.078751052	4.156006773	2.792087991	2403	Cables2	6.310846391	6.406887968	6.487885365	4703	Elmod1	9.686273553	9.796286684	9.941476377	7003	Hspb11	6.305334379	5.787605726	5.862309275	9303	Ndufs7	8.874768677	8.713826169	8.774378803	11603	Rell2	7.932520454	8.002992731	8.26107462	13903	Tec	2.098734369	3.444520239	3.684182672
104	1700016K19Rik	6.397399532	7.097952163	5.734143339	2404	Cabp1	8.804922012	8.923577025	9.078242094	4704	Elmod2	5.832518956	5.182328711	5.761825731	7004	Hspb2	0.064944352	2.690500705	2.306335219	9304	Ndufs8	9.381580688	9.170974148	9.080592661	11604	Reln	7.230136979	6.843555859	7.519666509	13904	Tecpr1	7.737350727	7.7043143	7.337272722
105	1700017B05Rik	5.600246086	5.190874049	5.337538093	2405	Cabp5	1.056778193	0.880533924	1.57145247	4705	Elmod3	5.917421432	6.229928143	6.423419276	7005	Hspb3	2.075656178	2.44382111	2.984812444	9305	Ndufv1	9.960203411	9.969603178	10.02301961	11605	Relt	4.995153154	5.358465344	5.935754332	13905	Tecpr2	7.967991605	7.786855738	7.856678403
106	1700017N19Rik	0.074260254	3.994685908	2.305286363	2406	Cabs1	2.721305537	6.757493989	4.502875974	4706	Elmsan1	6.011130359	6.235481839	6.20941698	7006	Hspb6	5.782493728	6.323196576	5.980250036	9306	Ndufv2	10.45024093	10.44314379	10.53361493	11606	Rem2	5.752250683	5.994870943	5.761550945	13906	Tecr	10.86038708	10.96706652	10.9693739
107	1700018B08Rik	1.064613801	3.372778432	0.983608529	2407	Cabyr	6.92426316	6.828285374	6.969192774	4707	Eln	4.886829639	4.691060605	4.787171989	7007	Hspb8	7.147766818	7.562211805	7.098565591	9307	Ndufv3	10.36000334	10.27571201	10.1297057	11607	Renbp	4.389504312	2.353008166	3.299834459	13907	Tectb	0.055149208	3.225233594	1.568187259
108	1700018B24Rik	0.115536729	5.159249005	1.559744303	2408	Cacfd1	7.199439198	7.273588266	6.95681832	4708	Elof1	8.079353652	7.888150437	8.04632665	7008	Hspbap1	4.076791022	4.17011122	4.441895291	9308	Neat1	8.657920497	8.95602348	8.751564258	11608	Rep15	3.059805175	2.443559439	2.984786075	13908	Teddm1b	2.659575359	3.03180594	2.982847769
109	1700018L02Rik	3.232079466	2.661776378	2.302384985	2409	Cachd1	7.354327448	7.357996679	6.945579348	4709	Elovl1	6.504050424	6.766704742	5.811542621	7009	Hspbp1	9.33532911	9.268488111	9.290636488	9309	Neb	3.983604218	4.016072997	4.442301289	11609	Repin1	7.894932442	7.929813062	8.040711677	13909	Teddm2	3.072095922	3.783712823	1.976095613
110	1700019D03Rik	2.904153139	3.647514854	4.152530568	2410	Cacna1a	9.871802376	9.971101403	10.12215977	4710	Elovl2	6.325725561	6.26807962	6.265893875	7010	Hspd1	9.674906619	9.539513472	9.568649225	9310	Nebl	5.964386368	5.761157509	6.08859663	11610	Reps1	6.821576367	6.97037102	6.875025275	13910	Teddm3	2.40252838	2.422390533	2.565695545
111	1700019G17Rik	2.948422904	5.551511064	4.678613468	2411	Cacna1b	8.050591901	7.66975928	8.013728646	4711	Elovl4	8.485838674	8.606557066	8.511355999	7011	Hspe1	9.054954525	8.989836373	9.021628435	9311	Necab1	8.90662529	8.714159127	8.982969061	11611	Reps2	9.048810433	8.823272613	9.054230365	13911	Tef	10.68411315	10.55456137	10.62113751
112	1700019G24Rik	2.644621924	2.457692105	0.979051828	2412	Cacna1c	7.692667601	7.255167018	6.886108771	4712	Elovl5	8.756653044	8.698326602	8.645203721	7012	Hspg2	3.661406221	4.835789706	2.559375693	9312	Necab2	7.407648833	7.217491583	6.533723533	11612	Rer1	8.499358645	8.918023213	8.844210884	13912	Tefm	4.60265829	4.418800758	4.148048538
113	1700019L03Rik	3.656556767	3.765491868	2.784958516	2413	Cacna1d	7.753247799	7.80363974	7.968881396	4713	Elovl6	8.603667172	8.258323466	8.697848259	7013	Hsph1	10.34505046	10.30117666	10.33201382	9313	Necab3	5.446642734	6.368347241	6.581318064	11613	Rere	9.453540484	9.457854068	9.623892165	13913	Tek	3.680452636	4.707958879	4.681871295
114	1700020A23Rik	0.013879255	0.92126446	0.990437367	2414	Cacna1e	8.705117707	8.604200368	8.301832293	4714	Elovl7	7.739903546	8.614630267	7.594223316	7014	Htatip2	4.783786201	5.090424103	5.150745389	9314	Necap1	9.351812963	9.129596771	9.337580361	11614	Rerg	5.542714574	5.793567929	5.533880055	13914	Tekt1	4.720499716	5.322211017	3.559635009
115	1700020I14Rik	8.544585306	8.581972281	8.934358204	2415	Cacna1f	0.005608334	0.940318714	0.992698372	4715	Elp2	9.750739414	9.545379866	9.431166908	7015	Htatsf1	9.8780149	9.659320991	9.793783854	9315	Necap2	6.708718654	6.494296162	6.455397465	11615	Resp18	8.255040158	7.917923462	8.223233668	13915	Tekt2	0.03249203	1.898414943	0.986188704
116	1700020L24Rik	0.047798258	2.478045794	1.98554506	2416	Cacna1g	6.387119964	6.696482545	6.761578962	4716	Elp3	8.649663388	8.411274648	8.459783234	7016	Htr1a	5.883130226	5.750492735	5.405786403	9316	Nedd1	2.693226413	4.383102678	3.88746464	11616	Rest	6.749567998	6.609939184	6.519429916	13916	Tekt4	3.246653501	4.339686992	2.979512289
117	1700021F05Rik	7.472836637	7.510785036	7.616837519	2417	Cacna1h	6.097419535	6.171811463	5.471134759	4717	Elp4	6.972236572	6.951936398	6.405704806	7017	Htr1b	2.046562262	0.88990511	1.572384477	9317	Nedd4	10.67311984	10.77370547	10.81761839	11617	Ret	3.242752857	2.63476875	2.786928666	13917	Tekt5	5.337985894	5.9876092	6.228617818
118	1700021K19Rik	8.084839905	7.877320597	8.008125304	2418	Cacna1i	6.293598806	6.829934351	7.003152497	4718	Elp5	8.24952232	8.249013528	7.996746551	7018	Htr1d	2.885396641	2.840133006	3.684535977	9318	Nedd4l	9.766969152	9.765776628	9.801795097	11618	Retn	1.639948853	2.220094438	2.796268165	13918	Telo2	5.501156873	5.550316387	5.067199442
119	1700021N21Rik	1.63465133	0.889895836	1.988213892	2419	Cacna2d1	10.98208568	10.65929466	10.99960222	4719	Elp6	4.938179371	5.010669542	4.369700961	7019	Htr1f	4.88314956	4.749380941	4.981523242	9319	Nedd8	11.24669004	11.23555579	11.12016441	11619	Retsat	6.29060486	6.469708243	6.149368798	13919	Ten1	7.446160947	7.490776109	7.488019744
120	1700023F06Rik	3.397891986	3.862754166	2.296491843	2420	Cacna2d2	7.875650303	7.881017895	8.251713525	4720	Emb	7.309734077	7.642128425	7.572777045	7020	Htr2a	4.59090722	3.12647344	4.683985627	9320	Nedd9	5.510609904	5.502832684	6.248183616	11620	Rev1	6.557466641	6.521424815	6.81317926	13920	Tenm1	7.663610791	6.908124316	7.631450828
121	1700024G13Rik	1.048007179	2.236113659	0.987888909	2421	Cacna2d3	8.168315491	8.293606069	8.218371716	4721	Emc1	8.804595619	8.65159155	8.631017787	7021	Htr2c	8.488991764	8.450369353	7.996522741	9321	Nefh	9.354968662	9.20461907	9.903400605	11621	Rev3l	8.745713519	8.676964659	8.841187061	13921	Tenm2	10.27356605	9.930676493	9.79051653
122	1700024P16Rik	4.58434787	3.291088512	3.565627903	2422	Cacna2d4	2.409942225	2.130674982	2.981141134	4722	Emc10	9.696724602	9.543652129	9.503552905	7022	Htr3a	4.314126833	3.75336803	3.565144689	9322	Nefl	11.91663821	11.73245968	11.98420543	11622	Rexo1	7.497010484	7.469471654	7.208659703	13922	Tenm3	9.426853372	9.223278715	9.616754104
123	1700025G04Rik	9.568320927	9.403435039	9.607555854	2423	Cacnb1	9.353729866	9.382145567	8.907647003	4723	Emc2	8.950941595	9.142522206	8.985628642	7023	Htr4	4.078509733	4.558376253	3.886413043	9323	Nefm	10.95035727	10.78852002	11.44251385	11623	Rexo2	9.426692335	9.402595648	9.242074186	13923	Tenm4	8.961602284	8.535188533	8.534623989
124	1700026D08Rik	4.539498411	5.419504546	3.675825308	2424	Cacnb2	7.473315892	7.45888509	7.623961936	4724	Emc3	8.383479218	8.424704908	8.018729856	7024	Htr5a	5.125273588	5.367947025	5.189610494	9324	Negr1	9.770358491	9.620719138	9.853510296	11624	Rexo4	8.11255444	7.749922576	7.89295679	13924	Tep1	3.884682942	3.529802464	4.151892037
125	1700026J14Rik	0.016591912	0.915573532	0.989762546	2425	Cacnb3	8.903408135	9.006765464	8.690759828	4725	Emc4	8.77037269	8.621922426	8.877953921	7025	Htr5b	2.410009844	3.318342239	1.975403071	9325	Neil2	3.404809078	4.107178077	3.98218332	11625	Rfc1	9.247453732	9.114325048	8.850227446	13925	Terf1	5.984600636	5.64186711	5.838200965
126	1700026L06Rik	2.654881808	3.586544812	0.974282426	2426	Cacnb4	11.1181841	11.07843229	11.56405371	4726	Emc6	9.754556404	9.722264577	9.522964926	7026	Htr6	1.070166534	2.203516351	2.571596436	9326	Nek1	8.074183625	7.870729539	7.968722325	11626	Rfc2	7.090368365	7.399376406	6.761008758	13926	Terf2	8.406531013	8.423772821	8.376672159
127	1700027A15Rik	0.050213561	3.919167847	0.982649747	2427	Cacng2	10.07736271	9.958449603	9.958744984	4727	Emc7	8.623571371	8.56199148	8.683942138	7027	Htr7	5.288521136	4.777927429	5.18941495	9327	Nek10	1.062793037	1.461121608	1.986248358	11627	Rfc3	5.749082622	5.421277789	5.788409578	13927	Terf2ip	8.442293869	8.496813778	8.487871534
128	1700028B04Rik	2.893745271	4.119290021	3.566931674	2428	Cacng3	7.752708914	7.522303246	8.024791765	4728	Emc8	7.774254169	8.049552487	7.767829334	7028	Htra1	8.665808142	8.723339308	8.623739775	9328	Nek11	2.093868916	3.782892646	3.304859246	11628	Rfc4	5.748373937	5.359495108	5.439476827	13928	Tes	4.770782604	4.492357857	4.373150478
129	1700028K03Rik	4.88717738	4.88338781	5.110334122	2429	Cacng4	3.879407622	4.028984395	3.439372775	4729	Emc8-1190005i06rik	5.932023426	5.452507408	5.762403196	7029	Htra2	7.229890432	7.363964788	7.045833611	9329	Nek2	3.057428386	3.05248755	2.568851076	11629	Rfc5	5.634124213	5.912847031	5.838881509	13929	Tesc	8.003597068	7.949155305	7.630973852
130	1700028P14Rik	3.391486751	3.964231766	3.684299945	2430	Cacng5	6.102411716	6.234007091	6.320565576	4730	Emc9	7.393958405	7.66663794	7.31064025	7030	Htra3	3.9909057	4.001304235	3.885462659	9330	Nek3	4.242327446	4.563974668	3.679726387	11630	Rfesd	7.11075368	6.757085733	6.40545377	13930	Tesk1	8.5761174	8.715788425	8.47981964
131	1700029H14Rik	1.075385233	3.71718926	2.570798117	2431	Cacng7	2.878658219	2.654111556	3.30593398	4731	Emcn	4.534331683	4.904403936	3.885105667	7031	Htra4	2.670176599	4.047428925	2.298508017	9331	Nek4	7.595968426	7.436398924	7.844587219	11631	Rffl	6.286352843	6.200447916	5.837522924	13931	Tesk2	5.941689892	6.063613775	6.595727499
132	1700029J07Rik	4.532894367	5.036167554	3.785446359	2432	Cactin	6.438297417	6.467395129	6.652888663	4732	Emd	8.303874412	8.164423799	8.088448366	7032	Htt	7.309869274	7.287828286	7.414668312	9332	Nek5	3.867947834	3.780861984	2.787543994	11632	Rfk	8.966003312	9.000872655	8.822297512	13932	Tet1	6.914813764	7.188105334	7.024269712
133	1700030C10Rik	3.653339275	3.668991398	2.979750426	2433	Cacul1	8.397433408	8.322090111	8.189362049	4733	Eme1	7.347527458	7.547318377	7.423008486	7033	Hunk	6.935189509	6.579418417	6.623964416	9333	Nek6	7.056080379	6.989856099	6.388286762	11633	Rfng	7.022433755	7.208076123	7.180111458	13933	Tet2	6.753960944	6.882544455	7.088965771
134	1700030J22Rik	5.207582989	5.086240705	4.733408253	2434	Cacybp	9.588231705	9.395784481	9.607557423	4734	Eme2	6.262682939	5.819653848	5.53419022	7034	Hus1	6.554529187	6.40812918	6.188900654	9334	Nek7	7.547782548	7.614096118	7.673749167	11634	Rft1	5.572373958	5.724545612	5.5649279	13934	Tet3	8.052022196	7.950810702	8.134915531
135	1700030K09Rik	7.260789282	7.534190331	7.472111567	2435	Cad	5.405235822	5.565067506	5.471417045	4735	Emg1	7.385431289	7.758962039	7.463662346	7035	Huwe1	10.34509733	10.46451758	10.57900463	9335	Nek8	4.149243616	3.435787586	3.567218159	11635	Rftn1	6.204330265	6.074216969	5.680154507	13935	Tex10	6.210623395	6.138507492	6.504132231
136	1700031M16Rik	0.100867515	4.54975275	2.566756762	2436	Cadm1	11.60921077	11.04045936	10.9841821	4736	Emid1	3.25839223	4.322246962	3.147289515	7036	Hvcn1	3.545136508	3.930734847	4.229968656	9336	Nek9	7.41159008	7.717713116	7.534739696	11636	Rftn2	7.924682133	7.911721957	7.487503855	13936	Tex11	2.878460993	3.688508726	1.977701245
137	1700034E13Rik	1.085211794	4.158242888	1.565354552	2437	Cadm2	11.88117771	12.04869535	12.33809526	4737	Emilin1	2.39821759	3.584122247	2.5667178	7037	Hyal1	3.770690899	3.299224681	3.149406579	9337	Nelfa	7.068503269	7.058227023	7.265930011	11637	Rfwd2	7.701908941	7.5093875	7.463618871	13937	Tex14	1.082151107	3.602165859	1.982642967
138	1700034G24Rik	4.072688275	3.747668233	4.891829729	2438	Cadm3	10.11494914	9.878240561	9.831732299	4738	Emilin2	3.876757327	2.37724355	3.440133625	7038	Hyal2	5.027387205	4.477432359	4.623982837	9338	Nelfb	6.888807123	7.318224826	7.129993699	11638	Rfwd3	6.860898334	6.707476868	6.957712441	13938	Tex15	4.460358044	4.09784855	4.627512283
139	1700034H15Rik	2.056951126	0.8587391	1.985533344	2439	Cadm4	8.385890385	9.038426073	9.10167833	4739	Eml1	7.250608746	7.541748711	7.480183104	7039	Hyal3	2.070393372	2.181846034	2.305259731	9339	Nelfcd	8.223360236	8.255194433	7.819158849	11639	Rfx1	6.517623247	6.145053586	5.593355997	13939	Tex2	7.901557568	8.123692898	8.114460827
140	1700034I23Rik	2.393032245	3.472908933	1.980399994	2440	Cadps	9.523707187	9.658089206	9.841157089	4740	Eml2	10.61768691	10.48791872	10.55458612	7040	Hydin	4.245199329	5.16015644	3.678881323	9340	Nelfe	5.965751295	6.316649832	6.228068595	11640	Rfx2	3.982109908	3.277917763	3.787703839	13940	Tex21	0.008381654	1.518469423	0.991898458
141	1700034P13Rik	5.807587635	5.444229225	6.190793978	2441	Cadps2	8.028732206	8.028011859	8.394049901	4741	Eml3	5.574862389	6.556013952	5.502688583	7041	Hyi	6.188585681	6.628946415	6.338064985	9341	Nell1	6.927542198	6.624293614	7.355459461	11641	Rfx3	8.314882485	8.328476259	8.159572516	13941	Tex26	0.040301499	1.46476982	2.795968656
142	1700037C18Rik	4.591745289	4.242630969	4.229453039	2442	Cage1	3.879685333	4.456615395	3.439293044	4742	Eml4	7.611875575	7.509192173	7.511232903	7042	Hykk	3.411276185	3.838879211	3.887270255	9342	Nell2	11.16259666	11.17288606	10.83871362	11642	Rfx4	6.057648911	5.922386479	5.910314264	13942	Tex261	7.410709916	7.338175872	7.431414532
143	1700037H04Rik	8.010369367	7.797507541	7.774328877	2443	Cahm	4.661849068	3.810666476	4.565416675	4743	Eml5	8.552154572	8.562563806	8.435229955	7043	Hyls1	3.878775675	4.030227468	3.681751776	9343	Nemf	9.576323528	9.459637058	9.57059182	11643	Rfx5	6.419752153	6.262035386	6.488111058	13943	Tex264	8.446531823	8.464318169	8.279118751
144	1700040L02Rik	4.385681294	4.576006044	2.977351002	2444	Calb1	8.861866161	8.710524128	8.822320519	4744	Eml6	6.012593938	5.942178222	6.338146403	7044	Hyou1	7.103997184	7.370863602	7.311014326	9344	Nenf	8.615059722	8.441728411	8.414623701	11644	Rfx7	7.261751838	7.399454924	7.838531966	13944	Tex30	5.537929266	5.454058881	5.762588863
145	1700047A11Rik	4.669085454	5.053240517	4.837847044	2445	Calb2	7.876189416	8.187033881	8.083221909	4745	Emp1	3.085816697	3.857511047	3.983016757	7045	Hypk	9.217458438	9.199871251	9.220800852	9345	Neo1	8.706455752	8.832561945	8.977314558	11645	Rfx8	1.643287831	1.461192355	0.98408996	13945	Tex33	2.093796222	4.807706733	3.568979082
146	1700047E10Rik	1.648044099	0.854507424	0.982609241	2446	Calca	3.245077354	3.309645753	2.786246231	4746	Emp2	7.114010762	7.266182501	7.15997263	7046	Hypm	0.076482464	4.232508483	1.565033254	9346	Nes	5.690146991	4.996939772	5.736261298	11646	Rfxank	5.326446782	5.429535342	5.440319953	13946	Tex37	0.005608334	0.940318714	0.992698372
147	1700047I17Rik2	8.731367306	8.850202145	8.579836445	2447	Calcoco1	8.040468533	8.448650424	7.934079124	4747	Emp3	4.600727889	3.904295357	4.936346279	7047	Iah1	5.833025124	5.975084607	5.371264979	9347	Net1	6.9620575	6.730947833	6.680091499	11647	Rfxap	7.238026402	6.935868828	6.945791327	13947	Tex40	4.599834965	4.600807583	4.56583729
148	1700047M11Rik	4.094464347	4.409611739	5.191129986	2448	Calcrl	6.245201893	6.436666532	5.47063603	4748	Emx1	5.811703275	6.180383149	6.520383369	7048	Iars	7.146344464	6.975390576	7.189590986	9348	Neto1	9.191154726	9.200826231	8.898793639	11648	Rgag4	6.382478211	5.939925065	6.639278014	13948	Tex9	6.756128907	7.291295708	7.320175457

149	1700048O20Rik	4.880290078	4.704775923	3.783833332	2449	Cald1	8.438126746	8.542422028	8.056419782	4749	Emx2	7.537770035	7.306671162	7.07776375	7049	Iars2	8.47262059	8.171460662	8.523123669	9349	Neto2	8.157895318	8.238385938	8.476026941	11649	Rgcc	6.767734668	6.628499772	7.046490762	13949	Tfam	7.774539722	7.854518777	8.094000012
150	1700049G17Rik	4.249628371	4.157696846	4.066749025	2450	Calhm2	3.660712477	3.757211155	3.301238764	4750	Emx2os	4.482107798	4.83325255	5.26655144	7050	Iba57	6.029422178	5.723117991	5.594598894	9350	Neu1	7.8347523	7.726754162	7.728203493	11650	Rgl1	7.239091678	6.890400317	7.098991923	13950	Tfap2a	2.382174982	2.463982508	1.983549973
151	1700049L16Rik	0.013878694	0.921265659	2.31336084	2451	Calm1	15.00323861	14.9385653	15.03707761	4751	En2	1.693975839	3.014782663	3.568369142	7051	Ibsp	3.376039036	1.410673268	2.791838202	9351	Neu2	4.158855181	4.250637454	3.680428005	11651	Rgl2	4.665418866	4.644505923	5.02587815	13951	Tfap2b	5.706402512	4.5829534	7.180830091
152	1700052K11Rik	4.546029238	5.408476429	5.266941489	2452	Calm2	14.01069304	13.91505359	13.96741587	4752	Enah	10.73337501	10.51787917	10.75889641	7052	Ibtk	7.656869534	7.422119498	7.694554123	9352	Neu3	2.654162407	3.198396817	1.97962164	11652	Rgl3	1.672745799	2.666906348	2.984063348	13952	Tfap4	3.989809373	4.664211466	4.505331816
153	1700056E22Rik	1.054644603	1.47627119	2.574024831	2453	Calm3	13.04258389	13.05921596	12.91789765	4753	Enc1	12.3616317	12.3354005	12.19489672	7053	Ica1	7.18939978	7.281441049	7.337719759	9353	Neu4	5.084873764	5.190534641	5.023065305	11653	Rgma	7.738831883	7.82156145	7.916839949	13953	Tfb1m	6.183424659	5.908346112	5.623472392
154	1700063D05Rik	1.05880811	2.488364803	2.309709398	2454	Calml4	5.984977592	6.937751693	4.141839953	4754	Endod1	8.788583984	8.859344158	9.216113641	7054	Ica1l	6.404925288	6.886420738	6.863118685	9354	Neurl1a	9.992993983	9.811129496	9.7332121	11654	Rgmb	3.900597359	4.849666643	5.069791113	13954	Tfb2m	6.80879941	6.814598123	7.07849142
155	1700064M15Rik	1.060813603	0.871609982	1.986623894	2455	Caln1	9.410986896	9.193180094	9.256144692	4755	Endog	6.421279698	6.510357395	6.624131107	7055	Icam1	3.247931465	3.554304975	2.979161227	9355	Neurl1b	11.16312793	10.86716412	10.8649243	11655	Rgn	0.055259663	1.444105961	1.984521932	13955	Tfcp2	6.05414532	5.587907388	5.707710097
156	1700065J18Rik	0.047764385	1.454263284	2.308398406	2456	Calr	11.03247311	10.93723341	10.77681233	4756	Endou	2.398331778	1.821042385	3.306698987	7056	Icam2	3.089065699	3.420598272	2.977198507	9356	Neurl2	2.873724177	3.338524551	2.567089986	11656	Rgp1	5.692641292	5.675203095	5.681020901	13956	Tfcp2l1	4.770486372	3.823446356	4.373231956
157	1700066M21Rik	6.437565621	6.453872633	6.45589626	2457	Calr3	2.088916148	4.214455821	2.982380289	4757	Endov	6.264795124	5.928295889	5.9577426	7057	Icam4	3.537546295	3.853918318	3.439494729	9357	Neurl4	7.752033021	7.776504736	7.587311487	11657	Rgs10	7.854867106	7.663285249	7.991497882	13957	Tfdp1	6.122779803	6.029600969	5.862361181
158	1700071K01Rik	1.649523921	0.850477551	2.794609333	2458	Calr4	3.067949278	2.854295385	2.982679635	4758	Eng	5.207758799	5.523013594	5.338524885	7058	Icam5	9.337689345	9.446077603	8.880644652	9358	Neurod1	7.504896961	7.347243648	7.024003247	11658	Rgs11	3.68163154	4.706151629	5.026935242	13958	Tfdp2	8.451219998	8.399761997	8.333029436
159	1700071M16Rik	1.060810357	0.871617186	1.986624511	2459	Calu	8.646862002	8.741820464	8.731535386	4759	Engase	4.326720833	4.49063494	4.302218996	7059	Ice1	7.874579097	7.750203605	7.838015046	9359	Neurod2	11.10349615	11.03887735	10.69761615	11659	Rgs12	8.427022026	8.053305596	7.892832049	13959	Tfe3	6.437334579	6.295796739	6.775390215
160	1700084C01Rik	5.638549095	6.071058154	5.787023299	2460	Caly	10.29355442	10.26318504	10.14864115	4760	Enho	8.502691675	8.477783302	8.389258948	7060	Ice2	6.143249626	5.904766016	6.150753179	9360	Neurod4	3.06327706	2.665299253	3.893881525	11660	Rgs13	2.637720561	0.850283868	0.982122419	13960	Tfeb	4.542510449	4.696338756	5.069039396
161	1700084E18Rik	2.070399194	1.84890536	1.56531421	2461	Camk1	8.838543954	8.85797564	8.780900713	4761	Enkd1	4.836901183	4.968603751	5.303394411	7061	Ick	7.328253184	7.491379941	7.179453954	9361	Neurod6	11.29349248	11.19283026	10.73815355	11661	Rgs14	8.848231327	8.947858637	8.397598511	13961	Tfg	8.085817241	7.897498443	8.056856459
162	1700084J12Rik	2.643795262	1.856422835	2.306035612	2462	Camk1d	10.83545548	10.96987183	10.87177422	4762	Enkur	6.124089259	6.644372478	6.355869069	7062	Icmt	6.900121479	7.109004653	7.002710584	9362	Neurog1	0.019296608	1.49768673	1.574431675	11662	Rgs16	5.039039893	5.039079795	5.227643067	13962	Tfip11	7.69457803	7.572464941	7.542173028
163	1700086L19Rik	6.078517218	6.050469987	6.390720826	2463	Camk1g	7.375901884	7.531872011	7.565069559	4763	Eno1b	8.798121413	9.061040452	8.655862294	7063	Icos	0.021963161	2.239668044	1.989549256	9363	Neurog2	2.062208403	2.198213298	1.567769213	11663	Rgs17	9.863701354	9.51598789	10.12597314	13963	Tfpi	5.494554753	3.978677478	4.623515038
164	1700086O06Rik	6.342669189	5.946363868	6.130284013	2464	Camk2a	14.19116784	14.35741416	14.0972916	4764	Eno1b	8.763752906	9.043532136	8.758010567	7064	Icosl	4.467046883	4.813962878	4.228256495	9364	Nexn	6.800567496	7.190474495	7.850722017	11664	Rgs19	6.073487703	5.621575367	5.76212086	13964	Tfpt	5.690917251	5.454391122	5.337344697
165	1700088E04Rik	4.830133876	5.104020287	4.565109522	2465	Camk2b	11.73210244	11.84907488	11.64485752	4765	Eno2	11.61635966	11.6366566	11.72061191	7065	Ict1	8.188315764	8.201316086	7.780590273	9365	Nf1	7.724748407	7.597381421	8.155362027	11665	Rgs2	10.09865865	9.830247385	10.16242442	13965	Tfr2	4.233922647	3.296079846	3.440041057
166	1700094D03Rik	6.469730184	6.804040593	5.956585276	2466	Camk2d	8.475526843	7.634379965	8.447959194	4766	Eno3	3.384711368	1.81054106	3.153333625	7066	Id1	7.554629049	7.454439464	7.15963656	9366	Nf2	8.877856115	8.934213758	8.81923494	11666	Rgs20	6.974020571	6.948490781	7.266267833	13966	Tfrc	8.568534366	8.798995368	8.800315337
167	1700094J05Rik	6.556303526	6.748172389	6.851018707	2467	Camk2g	8.778379709	9.034776409	9.449827831	4767	Eno4	4.394089596	4.310450081	3.298244949	7067	Id2	11.33326171	11.28634747	11.54653468	9367	Nfam1	2.905038831	3.846497757	4.37566323	11667	Rgs22	2.906179839	5.009457316	3.887983646	13967	Tg	1.235386454	5.959408827	4.438221158
168	1700096K18Rik	4.671177738	5.340426325	4.369397154	2468	Camk2n1	14.91459283	15.04985787	15.47439586	4768	Enoph1	7.259654898	7.196021137	7.346653309	7068	Id3	8.481245665	8.503681974	8.495744609	9368	Nfasc	10.15605064	10.43181551	10.09500735	11668	Rgs3	4.613258258	5.686812905	5.373304131	13968	Tgds	3.877308411	3.760923341	2.97802343
169	1700099I09Rik	3.87873295	1.743290842	3.68176314	2469	Camk2n2	8.835546268	8.529335703	9.213827625	4769	Enox1	6.419153923	5.959241744	6.319876926	7069	Id4	7.740618687	8.294645711	8.13482788	9369	Nfat5	8.151547958	7.966372255	8.15002277	11669	Rgs4	10.95562858	10.96115354	11.32098655	13969	Tgfa	3.266036841	4.010580775	4.374311343
170	1700101I11Rik	5.395831471	5.089485078	4.886521854	2470	Camk4	9.981428311	10.02157095	10.23745591	4770	Enox2	7.122801127	6.999881683	6.579256656	7070	Ide	7.239926747	7.150396628	6.99086761	9370	Nfatc1	4.248038867	3.825518687	3.785574497	11670	Rgs5	7.926571381	7.91355174	8.165007458	13970	Tgfb1i1	5.211841957	5.425286045	6.069815161
171	1700101O22Rik	0.024618663	2.924119565	0.987891894	2471	Camkk1	8.068777406	8.023170637	7.837801667	4771	Enpep	1.672836242	1.394207486	1.979597107	7071	Idh1	8.450423921	8.401251786	8.218361674	9371	Nfatc2	5.044403473	5.64537637	5.708146529	11671	Rgs6	4.541500728	4.284348309	5.340302333	13971	Tgfb2	8.135221595	8.364410321	7.479139596
172	1700102H20Rik	2.869890235	2.673676612	2.568161204	2472	Camkk2	9.913437665	9.950397057	10.18465131	4772	Enpp1	4.60872929	5.012659335	5.025060998	7072	Idh2	7.531022	7.717875859	7.098376266	9372	Nfatc2ip	5.070255391	4.597681218	4.50377369	11672	Rgs7	9.028878232	9.030222761	9.03528362	13972	Tgfb3	5.723078307	5.772318603	5.735234414
173	1700102P08Rik	1.054653008	2.226185488	2.574023494	2473	Camkmt	4.178397335	4.473727498	4.838598736	4773	Enpp2	12.59411396	13.56117061	11.91619226	7073	Idh3a	10.18380096	10.15639796	10.25614262	9373	Nfatc3	7.539757956	7.383255146	7.624022654	11673	Rgs7bp	12.20925894	12.24181749	12.38672031	13973	Tgfbi	4.390842184	4.31722765	3.680025424
174	1700109H08Rik	4.0772149	2.962325787	4.68373554	2474	Camkv	10.22490903	10.0931131	9.894335359	4774	Enpp3	2.046569445	1.480236035	1.572382377	7074	Idh3b	9.3936083	9.424482535	9.431175181	9374	Nfatc4	2.102521713	3.559362811	2.562504078	11674	Rgs8	8.142729674	8.51375413	8.781098788	13974	Tgfbr1	6.228151396	5.552860304	6.067508969
175	1700109K24Rik	0.042815083	0.867280315	0.984088381	2475	Caml	6.938703303	6.96725007	7.407074009	4775	Enpp4	7.807808748	7.856384061	7.511109644	7075	Idh3g	9.102800176	9.064299006	9.159959454	9375	Nfe2l1	9.924424581	10.00179828	9.995439097	11675	Rgs9	6.236889505	7.173098154	7.284526248	13975	Tgfbr2	5.401598012	5.543866214	5.337510644
176	1700110K17Rik	3.050049499	1.867374043	1.984186791	2476	Camsap1	9.282387183	9.009574334	9.182123969	4776	Enpp5	8.960377535	8.833922784	9.387449077	7076	Idi1	7.14753114	7.378458664	7.149701346	9376	Nfe2l2	7.270826626	7.416317553	7.246622911	11676	Rgs9bp	1.07023508	2.203417613	2.30776791	13976	Tgfbr3	6.23251212	6.467899621	6.337290612
177	1700112J16Rik	3.073745772	3.318716671	3.442680216	2477	Camsap2	10.41132045	10.17411403	10.29060266	4777	Enpp6	6.101760375	6.535501499	6.150330819	7077	Idi2	0.016604741	0.915547107	1.574993764	9377	Nfe2l3	4.941309992	5.834896226	5.3026777	11677	Rgsl1	2.383229825	2.888234562	2.570269214	13977	Tgfbr3l	1.660660363	2.88267854	0.978590699
178	1700113A16Rik	9.094164805	8.998002951	8.9254199	2478	Camsap3	6.31003323	6.689432515	6.681190861	4778	Ensa	10.15858564	10.12800972	10.05412355	7078	Idnk	6.752452761	6.745589107	6.638062844	9378	Nfia	11.15600397	11.24739967	11.02368288	11678	Rhbdd1	4.715402845	4.712929061	4.440472811	13978	Tgfbrap1	6.6957313	6.756887316	6.748175988
179	1700120C14Rik	2.382142842	2.69250766	0.980338437	2479	Camta1	11.2931503	11.38175081	11.52447499	4779	Enthd2	4.887998292	5.375805203	4.437283665	7079	Ido1	2.896607832	3.428424963	3.149201061	9379	Nfib	11.45499947	11.46548252	11.19913157	11679	Rhbdd2	8.420118851	8.408143069	8.507611769	13979	Tgif1	3.768818601	4.039639918	2.561608689
180	1700120K04Rik	0.011144214	0.927240035	1.576210158	2480	Camta2	9.37284216	9.626755204	9.383012055	4780	Entpd1	4.726790969	4.927447641	4.837871423	7080	Ido2	2.069266983	3.057265848	1.982342403	9380	Nfic	10.14416743	10.39064479	10.57717297	11680	Rhbdd3	6.013444904	6.365091896	6.001796618	13980	Tgif2	4.072105416	4.102869753	4.375587508
181	1700123L14Rik	0.06253749	3.219880728	1.567065072	2481	Cand1	9.017090822	8.791048257	9.088580628	4781	Entpd2	5.571939942	5.388008978	5.40575757	7081	Ids	11.09861877	10.97427629	10.94937943	9381	Nfil3	5.359886767	4.633176438	5.30318432	11681	Rhbdf1	4.591580967	3.124344479	4.151148708	13981	Tgm1	1.040709515	0.915535432	0.989758031
182	1700123M08Rik	2.073612669	2.67723507	1.981212299	2482	Cand2	10.70077522	10.76201156	10.59743129	4782	Entpd3	5.441929835	5.818091115	5.98087356	7082	Idua	5.258135083	5.615359916	5.623382823	9382	Nfix	11.64935716	11.6445801	11.42551569	11682	Rhbdl1	6.040162559	6.899982222	6.42270077	13982	Tgm2	6.014132488	6.022490164	6.45626427
183	1700123O20Rik	6.665477985	6.758406483	6.638142905	2483	Cant1	7.346277129	7.418184928	7.346565545	4783	Entpd4	7.412292717	7.335176333	7.787695317	7083	Ier2	3.89012519	4.438321867	3.979885573	9383	Nfkb1	4.252065921	4.708565976	4.504317498	11683	Rhbdl2	0.021959717	1.913290581	0.988493771	13983	Tgm3	1.080579351	1.428223781	3.447176048
184	1700124L16Rik	5.533067349	5.124717269	5.338542439	2484	Canx	11.27779763	11.20949318	11.26132849	4784	Entpd5	6.807524132	6.874151289	6.282708519	7084	Ier3	6.48890882	6.154687177	6.992592292	9384	Nfkb2	11.17402949	11.54074326	11.29683847	11684	Rhbdl3	6.488301031	6.393627148	6.001394326	13984	Tgm4	2.676074272	3.767983504	3.440926972
185	1700125H20Rik	1.038054777	0.921260594	1.991049881	2485	Cap1	9.105659002	9.134959861	8.996735986	4785	Entpd6	5.859749025	5.841447431	5.40518997	7085	Ier3ip1	10.12076893	10.10355537	10.01614439	9385	Nfkbia	9.889111667	9.300126755	9.281312902	11685	Rheb	9.280382325	9.39066602	9.406279268	13985	Tgoln1	10.02288804	9.848926537	9.803318252
186	1810010H24Rik	4.077224644	4.441480189	3.980323672	2486	Cap2	9.855435799	10.00045919	9.968689448	4786	Entpd7	5.337257153	6.009609511	6.696258064	7086	Ier5	8.556976191	8.8467071	8.825517835	9386	Nfkbib	8.919655734	9.105759574	8.982868737	11686	Rhebl1	5.808074854	6.053930264	5.887224164	13986	Tgoln2	8.576230784	8.348365228	8.652518019
187	1810011H11Rik	1.647969487	2.703209741	1.985196728	2487	Capg	2.662393171	3.026659656	3.305520759	4787	Eny2	9.124864874	9.169117949	9.196795028	7087	Ier5l	5.331661969	5.868714791	5.981414644	9387	Nfkbid	2.658107114	3.034486419	2.301970389	11687	Rhno1	6.264213561	5.5603064	5.680153207	13987	Tgs1	8.642639202	8.436699826	8.707997021
188	1810011O10Rik	4.145560869	2.125631887	2.298803958	2488	Capn1	5.080204735	5.235477655	5.266187952	4788	Eogt	3.53678445	3.945890684	3.439691603	7088	Iffo1	6.060320776	6.626246414	6.406615099	9388	Nfkbie	4.521298646	3.756542731	3.301144491	11688	Rhoa	9.57256902	9.720673701	9.744892497	13988	Tgtp1	3.232007175	2.661958271	2.566691459
189	1810013L24Rik	7.875505945	7.892284348	8.204238342	2489	Capn10	6.28438403	5.814962976	5.887090798	4789	Eomes	3.399318824	4.978075216	2.295959792	7089	Iffo2	6.271428481	6.328911754	6.722322709	9389	Nfkbil1	5.935134411	5.909730959	5.76162061	11689	Rhob	9.807029719	9.830371231	9.989818887	13989	Tgtp2	1.686407663	3.881487622	3.443319418
190	1810014B01Rik	5.396439672	4.52069452	5.505384325	2490	Capn12	8.720978986	9.014042687	8.825510859	4790	Ep300	8.807845906	8.555141036	8.896081982	7090	Ifi203	5.030325722	5.17234599	4.225251855	9390	Nfkbiz	4.25417525	4.48303015	4.982217144	11690	Rhobtb1	3.268786763	4.497645407	3.436036815	13990	Th	1.680191913	3.579663159	2.982892254
191	1810020O05Rik	2.651659068	1.402711871	2.303806727	2491	Capn15	6.008384643	6.053813077	5.594047015	4791	Ep400	8.023002896	7.841896545	8.002346268	7091	Ifi204	2.378875525	2.200741769	1.568146439	9391	Nfrkb	5.934010134	5.748723622	5.594689888	11691	Rhobtb2	7.767293035	7.849452283	8.035547034	13991	Thada	5.914256763	5.582367376	6.424171891
192	1810021B22Rik	4.406587298	5.284448526	4.564691832	2492	Capn2	8.224685084	8.178295495	8.208915738	4792	Epas1	8.752735424	8.49849329	8.561094137	7092	Ifi27	8.027798473	8.176648824	7.526487453	9392	Nfs1	6.937335075	6.60250504	6.910958693	11692	Rhobtb3	7.310736666	7.406361896	7.63120971	13992	Thap1	6.615221165	6.195409741	6.149785611
193	1810022K09Rik	8.514654856	8.377011923	8.208642328	2493	Capn3	5.513065707	6.402215227	5.533138813	4793	Epb4.1	7.982001141	8.151112109	8.297602344	7093	Ifi27l2a	3.243214583	3.563100527	2.786793184	9393	Nfu1	7.374681654	7.498519022	7.01287878	11693	Rhoc	7.41077653	7.725492886	7.159630702	13993	Thap11	8.172100928	7.87633816	8.078205296
194	1810024B03Rik	0.079049407	3.052386354	2.792060585	2494	Capn5	6.536903516	6.790732598	5.78591603	4794	Epb4.1l1	11.35611657	11.33763307	11.48084062	7094	Ifi27l2b	1.622199973	0.921264841	0.990437412	9394	Nfx1	8.993674336	9.141900292	9.206472546	11694	Rhod	4.066088046	3.659321034	3.149169197	13994	Thap2	6.36157261	6.028672514	6.00240666
195	1810026B05Rik	6.556536189	6.735435225	6.761762663	2495	Capn6	2.6587503	2.151747609	2.789547946	4795	Epb4.1l2	9.209583276	9.022322024	9.227985828	7095	Ifi30	4.664847228	4.748719425	4.735334501	9395	Nfxl1	4.778387126	4.935176293	5.069225298	11695	Rhof	6.6912893	6.455616258	6.283710295	13995	Thap3	6.692693638	6.437771359	6.565270036
196	1810026J23Rik	7.715431926	7.717650097	7.319648674	2496	Capn7	7.702773062	7.663654447	7.609171263	4796	Epb4.1l3	9.302442518	9.32924392	9.687456657	7096	Ifi35	2.661713994	2.851598037	3.153029076	9396	Nfya	4.933722301	4.413821475	4.623698736	11696	Rhog	7.649816471	7.863586812	7.380598466	13996	Thap4	6.690765692	6.194003739	6.283840624
197	1810030O07Rik	7.421174396	7.566050415	7.78769349	2497	Capn9	1.04803393	1.909311176	0.987882046	4797	Epb4.1l4a	5.718241913	4.868359036	5.959490252	7097	Ifi44	3.767685444	3.556626925	2.562036099	9397	Nfyb	9.543942232	9.510401194	9.538437688	11697	Rhoh	3.056753462	2.451374138	1.981756576	13997	Thap6	2.887239993	2.836263684	3.304061689
198	1810032O08Rik	3.406283915	3.139809872	3.788344274	2498	Capns1	9.831871002	9.768810373	9.680420098	4798	Epb4.1l4aos	7.594732432	7.468538193	7.045513035	7098	Ifi47	3.656958302	3.299877652	4.154009535	9398	Nfyc	6.587723642	6.5099475	6.319302907	11698	Rhoj	6.030882198	5.769522253	5.887378746	13998	Thap7	5.508018719	5.976839543	5.735364961

199	1810037I17Rik	8.978631492	9.06665955	9.179726833	2499	Capns2	0.011134149	1.512830812	0.991149167	4799	Epb4.1l4b	1.689343965	3.450689172	3.792185929	7099	Ifih1	4.087830849	4.984659459	3.883954468	9399	Ngb	4.927415257	3.616711261	4.566240945	11699	Rhoq	8.425373697	8.359970774	8.828813539	13999	Thbd	6.417005538	6.123398952	6.625171785
200	1810041H14Rik	1.073835162	0.842345959	1.984177659	2500	Caprin1	9.835574668	9.935910476	9.90924017	4800	Epb4.1l5	4.942054636	5.46162691	5.189600364	7100	Ifit1	5.424760597	4.799794238	4.064913265	9400	Ngdn	8.022941944	7.924637567	7.793518245	11700	Rhot1	8.556161496	8.534904282	8.546062486	14000	Thbs1	2.891646125	4.041880626	2.562052355
201	1810041L15Rik	8.051100711	8.220554266	7.637910096	2501	Caprin2	4.328620982	4.075378875	4.840098904	4801	Epc1	8.069298207	8.017180706	8.062113323	7101	Ifit1bl1	4.080569965	4.162601048	4.790336201	9401	Ngef	9.141366381	9.204229283	9.596748118	11701	Rhot2	8.004507443	7.77384064	8.067730525	14001	Thbs2	3.661680484	3.851902021	3.14809339
202	1810043G02Rik	7.111165947	6.827325816	6.838094475	2502	Capsl	6.11263621	5.945205597	4.562114119	4802	Epc2	8.885080537	9.074067085	9.211317439	7102	Ifit1bl2	3.077648004	3.010029414	2.29812927	9402	Ngf	2.897790488	3.546575484	3.148929711	11702	Rhou	8.438347415	8.652593854	8.471811612	14002	Thbs3	4.880730136	5.323336998	4.226742868
203	1810043H04Rik	8.832299872	9.084321319	8.980039129	2503	Capza1	6.385621168	6.436341004	6.371956428	4803	Epcam	2.376465932	1.876037667	0.98262943	7103	Ifit2	5.327920099	5.395384111	5.86456404	9403	Ngfr	3.773638693	4.395232891	3.789190353	11703	Rhov	5.359802152	4.633371153	4.679142877	14003	Thbs4	6.754805062	7.489074206	7.046241083
204	1810044D09Rik	3.250308593	3.76399949	3.5665212	2504	Capza2	9.230664898	9.355343705	9.357176366	4804	Epdr1	8.920006043	8.995109176	8.985594715	7104	Ifit3	6.057401022	5.762053469	6.002291777	9404	Ngfrap1	11.44796366	11.30326876	11.40152082	11704	Rhpn1	3.87400589	3.303655168	2.296797622	14004	Thegl	0.024640653	1.909343172	1.573372496
205	1810055G02Rik	4.604828869	5.283693628	4.73526739	2505	Capzb	10.45451557	10.43863861	10.4030875	4805	Epg5	6.38187234	6.004061708	6.149871935	7105	Ifit3b	4.466158285	4.084784626	4.150121271	9405	Ngly1	5.970397654	6.931032292	6.735188408	11705	Rhpn2	5.122232127	4.879522962	5.338996645	14005	Them4	8.322864174	8.286250941	8.040590533
206	1810058I24Rik	6.938671481	7.125832111	6.886736489	2506	Car10	8.848605431	8.806523165	9.308989828	4806	Epha10	6.248586155	5.240486023	6.247226041	7106	Ifitm1	5.072313956	4.41668518	4.371316027	9406	Ngp	0.019289323	2.243351873	0.989115077	11706	Rian	9.818459519	9.40286421	9.883938725	14006	Them6	7.159206762	7.601582305	7.534757259
207	1810062O18Rik	3.241273402	2.839717274	3.304420604	2507	Car11	8.749682087	8.874259451	9.075550017	4807	Epha2	1.05680104	0.880483576	0.985627801	7107	Ifitm10	2.081805282	2.663402562	0.973810425	9407	Ngrn	7.540030314	7.421291341	7.594767094	11707	Ribc2	2.066954511	3.485621698	0.97946215	14007	Thg1l	5.465891234	4.735193802	5.190143225
208	2010001A14Rik	5.608756675	6.321992706	6.247653172	2508	Car12	7.066381678	7.578073779	5.184338272	4808	Epha3	3.968742299	3.010682158	4.307639111	7108	Ifitm2	5.428156308	4.973059926	5.068587424	9408	Nhej1	3.248683724	3.000170957	2.978954496	11708	Ric1	4.936906338	5.558985369	5.068363889	14008	Thnsl1	8.112964502	8.048615298	8.008015607
209	2010012O05Rik	7.841638098	7.850023676	7.728141657	2509	Car13	1.649542165	0.850427713	0.98213898	4809	Epha4	11.2335274	11.31812242	11.12461542	7109	Ifitm3	8.016100335	7.803610067	7.510957391	9409	Nhlh1	3.873683294	3.554829732	2.785489787	11709	Ric3	8.676943537	8.651096285	8.822398519	14009	Thnsl2	4.784466687	5.008595929	5.30316005
210	2010107E04Rik	10.56827444	10.40284989	10.28029439	2510	Car14	7.456867282	8.104308734	7.447270503	4810	Epha5	9.485609884	9.392239673	9.365811022	7110	Ifitm7	2.417229836	3.437866715	3.984181095	9410	Nhlh2	4.731953542	5.80983853	5.265824546	11710	Ric8	7.814611256	7.638085826	7.714719087	14010	Thoc1	8.842232792	8.846055521	8.945811685
211	2010107G12Rik	1.65102889	0.846353569	2.30746008	2511	Car15	5.108205458	4.371739477	3.144319847	4811	Epha6	5.836715791	5.835361769	6.024656776	7111	Ifltd1	2.862321024	1.431398419	1.566702548	9411	Nhlrc1	6.401647293	6.664597	6.208483241	11711	Ric8b	7.861360374	7.643963422	7.893049454	14011	Thoc2	10.12891762	9.925010656	9.992596249
212	2010107G23Rik	6.794963545	6.86099636	6.980335335	2512	Car2	10.601273	10.63943177	10.68041986	4812	Epha7	9.373117515	9.490679936	9.441441637	7112	Ifnar1	6.924946943	6.905670007	7.150619058	9412	Nhlrc2	6.291879041	6.635502821	6.775137879	11712	Rictor	8.162234798	8.109733213	8.083300422	14012	Thoc3	7.867554477	7.618260901	7.714710603
213	2010109A12Rik	1.052545688	0.88982875	2.310793657	2513	Car3	3.9904425	1.675451213	4.840921717	4813	Epha8	3.093071712	3.936672686	2.976154708	7113	Ifnar2	7.328892139	7.680512754	7.463923081	9413	Nhlrc3	1.050317946	0.894696579	0.987295819	11713	Rif1	9.33562304	9.167986361	9.475902686	14013	Thoc5	6.912036273	6.804479017	6.813005785
214	2010111I01Rik	5.539553987	5.75109934	6.047791821	2514	Car4	7.892904707	7.862579889	7.217728897	4814	Ephb1	8.596990301	8.998601766	9.064953128	7114	Ifngr1	6.292632898	6.593780312	6.609517017	9414	Nhp2	7.602299895	7.353895875	7.149601207	11714	Riiad1	3.77222302	4.579440385	3.439981211	14014	Thoc6	5.635458134	5.446582896	5.88762334
215	2010204K13Rik	9.110583033	8.985613466	8.397466575	2515	Car5a	1.632792546	0.894663092	1.572862173	4815	Ephb2	7.110232782	6.794040263	6.911232249	7115	Ifngr2	7.82675168	7.444593298	7.43933569	9415	Nhp2l1	8.76637343	8.455513859	8.483744966	11715	Rilp	3.252402515	4.262026556	2.784089156	14015	Thoc7	9.451355035	9.414925695	9.55932591
216	2010300C02Rik	9.097391245	9.296058691	9.029786915	2516	Car5b	5.654165012	5.240962081	4.299073237	4816	Ephb3	6.265468408	5.665646877	6.229004156	7116	Ifrd1	8.645584051	8.275944563	8.256002339	9416	Nhs	2.394841668	2.438158134	2.567518094	11716	Rilpl1	7.868224256	7.649801792	7.754682091	14016	Thop1	5.568948773	5.261835302	5.108366429
217	2010315B03Rik	4.994647619	5.359407344	5.624540621	2517	Car7	6.708659631	6.920584763	6.761767166	4817	Ephb4	1.118656321	3.021772293	3.304979944	7117	Ifrd2	3.968291583	2.399621654	3.151174167	9417	Nhsl1	6.901664864	6.846412236	6.97992089	11717	Rilpl2	6.148663693	6.228094861	6.708856237	14017	Thpo	3.249125917	3.301467479	3.30248729
218	2010320M18Rik	6.166343414	5.965615088	6.247402088	2518	Car8	5.916114783	5.984188774	6.653769405	4818	Ephb6	8.214231637	8.406804435	8.355350829	7118	Ift122	6.653522967	7.318097183	7.256585458	9418	Nhsl2	8.619037733	8.587084548	8.538501269	11718	Rimbp2	6.2705483	6.598929893	6.283761202	14018	Thra	9.888970451	9.468649101	9.609359317
219	2200002D01Rik	5.782112484	6.185099842	6.002777659	2519	Car9	2.065738467	0.830973166	2.793399318	4819	Ephx1	6.604438574	6.993603854	6.60927608	7119	Ift140	5.627995752	4.96111952	5.536006358	9419	Nicn1	9.094343707	9.052389776	9.075427582	11719	Rimbp3	1.70042376	3.868116517	3.1505695	14019	Thrap3	9.099970896	9.025158741	9.051460946
220	2210011C24Rik	2.671468239	2.63094183	3.984559763	2520	Card10	4.882430112	5.386013774	3.675514564	4820	Ephx2	6.818537285	6.511265729	6.471870863	7120	Ift172	6.098288004	5.930167551	5.707816533	9420	Nid1	4.404921594	4.479639948	4.735754282	11720	Rimkla	5.434622448	5.333374807	5.338030999	14020	Thrb	8.965531582	8.683076372	8.780927852
221	2210013O21Rik	7.298276939	7.095073493	7.056709469	2521	Card14	2.370173185	1.891507377	2.309703697	4821	Ephx4	7.045334339	6.957295989	7.247391864	7121	Ift20	9.09992959	8.987443191	8.951487913	9421	Nid2	3.668924518	4.444261136	3.980709444	11721	Rimklb	6.457377059	6.591948981	6.813282604	14021	Thrsp	7.881715044	8.262797342	7.310027889
222	2210015D19Rik	5.908177111	5.480540874	5.594958969	2522	Card6	3.407380122	3.845829124	3.680657059	4822	Epm2aip1	9.426775692	9.108515504	9.3485233	7122	Ift22	7.058225815	7.04637551	7.337950043	9422	Nif3l1	6.72178668	6.595367473	6.638640339	11722	Rims1	8.907326097	8.943522922	9.211345208	14022	Thsd1	3.542335042	3.644508801	3.147036864
223	2210016F16Rik	6.588435411	6.747422168	6.708165036	2523	Carf	5.988456011	6.16183081	6.407179466	4823	Epn1	10.44996003	10.35073401	10.18453007	7123	Ift27	5.984275364	5.382277392	5.78749225	9423	Nifk	8.783515186	8.466930675	8.495677196	11723	Rims2	9.927620435	9.688883333	10.21609503	14023	Thsd4	6.541671742	6.928908336	6.666376743
224	2210016L21Rik	9.650187987	9.499508161	9.668298227	2524	Carhsp1	8.108477236	8.256884903	8.194394302	4824	Epn2	9.878179095	9.967444258	9.831727276	7124	Ift43	6.361949079	6.181432012	6.067602604	9424	Nim1k	7.7529922	7.644007277	7.741519286	11724	Rin1	7.663152463	7.431973253	7.337599303	14024	Thsd7a	8.823046868	8.926575812	9.557588294
225	2210018M11Rik	7.841412941	7.564084772	7.638088886	2525	Carkd	7.218926721	6.91227456	6.773802102	4825	Epn3	4.472993117	5.625706343	4.439222557	7125	Ift46	6.693984673	6.494730639	6.594772614	9425	Nin	7.103853519	7.33109147	7.149772006	11725	Rin2	7.589339796	7.65044661	7.687245573	14025	Thsd7b	4.535899347	4.945840215	4.736690457
226	2210404O09Rik	4.165847465	3.626860825	4.567200326	2526	Carlr	2.070395134	1.419320874	3.572522009	4826	Epor	4.177051962	4.8910271	5.111236768	7126	Ift52	6.725235578	6.927130587	7.03572307	9426	Ninj1	6.632940399	6.868444182	6.283411991	11726	Ring1	4.168668504	4.812354421	4.373087784	14026	Thtpa	6.398072286	6.048054621	6.150309425
227	2210406O10Rik	5.073794199	4.5897596	5.025839389	2527	Carm1	8.140805647	8.022516881	7.991151761	4827	Eppk1	3.374639219	3.053951931	1.981765808	7127	Ift57	8.02319829	8.105103114	8.088554031	9427	Ninj2	5.121312816	5.499112655	4.886808495	11727	Rinl	3.085096876	3.858666298	3.68230688	14027	Thumpd1	7.507247167	7.516629069	7.526807518
228	2210407C18Rik	0.008383876	0.933569984	1.576874431	2528	Carns1	5.781805506	5.883219425	6.046703256	4828	Eprs	9.932374095	9.93970766	9.970086871	7128	Ift74	8.567269333	8.301549769	8.218331513	9428	Ninl	4.995280463	5.800881568	5.708744007	11728	Rint1	6.078995896	5.763762359	6.668383786	14028	Thumpd2	3.256975414	2.802271173	4.305275151
229	2210408F21Rik	6.873328601	6.708464486	7.025086833	2529	Cars	6.29070458	6.296051036	6.595303336	4829	Eps15	10.28384877	10.24725251	10.24911632	7129	Ift80	6.606869091	6.999182738	7.013628038	9429	Nip7	7.260567601	7.32228029	7.246723537	11729	Riok1	7.471241674	7.447931868	7.109037364	14029	Thumpd3	6.652019446	6.803242955	6.934412007
230	2210408I21Rik	6.264878293	6.09242763	5.679980436	2530	Cars2	5.597164101	5.003634724	5.709953016	4830	Eps15l1	8.162726072	8.347053466	8.149892455	7130	Ift81	7.664635665	7.552455963	7.623996399	9430	Nipa1	6.400588536	6.176493083	6.283914898	11730	Riok2	6.507421451	6.205476458	6.708238355	14030	Thy1	11.76702149	11.67663876	11.58081602
231	2210416O15Rik	1.671507561	2.668704214	2.567537324	2531	Cartpt	5.132218732	5.797526457	6.210718547	4831	Eps8	7.693993387	7.155460194	7.666754509	7131	Ift88	6.833418582	6.564772351	6.638247473	9431	Nipa2	7.308813931	7.064268689	7.265448452	11731	Riok3	7.673724641	7.86639718	7.96318448	14031	Thyn1	8.322929209	8.286128611	8.134801739
232	2300009A05Rik	7.701987186	7.950065221	7.264863587	2532	Casc1	2.086131558	3.579643956	1.560513002	4832	Eps8l1	3.555485736	4.22963433	5.070808161	7132	Igbp1	7.300193185	7.31285676	7.47200753	9432	Nipal2	4.788378694	5.120501994	5.02363552	11732	Ripk1	4.779593273	4.746144878	4.838548947	14032	Tia1	7.572840893	7.501537359	7.673513968
233	2310001K24Rik	1.636484694	1.898331197	1.987797604	2533	Casc3	7.943841663	7.676006057	7.793793193	4833	Eps8l2	3.251638352	4.03380847	3.301868327	7133	Igbp1b	0.03248347	2.226239257	1.57192386	9433	Nipal3	7.619755206	7.530223556	7.754952824	11733	Ripk2	4.059996265	3.313248661	3.303923747	14033	Tial1	8.157152115	8.071135707	8.297598076
234	2310002D06Rik	2.415412598	3.167231611	3.150814787	2534	Casc4	8.993387343	9.000373796	8.991226106	4834	Eps8l3	4.349099715	5.483922496	5.736016649	7134	Igdcc3	5.071111073	5.253208134	3.294660611	9434	Nipal4	4.066834555	3.857309196	3.301751409	11734	Ripply2	5.079244441	5.00789613	5.228803998	14034	Tiam1	8.734635103	8.524046481	8.487711721
235	2310002F09Rik	5.290803373	5.731015979	5.26509221	2535	Casc5	1.659236677	2.18651248	2.569785861	4835	Epsti1	3.537264309	3.94502002	3.148481867	7135	Igdcc4	6.975714183	6.664884834	7.275086297	9435	Nipbl	10.2085142	10.13694783	10.1882233	11735	Ripply3	2.065824137	2.461773348	2.306321346	14035	Tiam2	7.124869861	7.291524969	7.218581355
236	2310009A05Rik	6.423547158	6.804759039	6.652214699	2536	Casd1	8.532036587	8.730364883	8.754888087	4836	Ept1	8.216622059	8.618907909	8.744919146	7136	Igf1	7.127874136	7.010591109	7.881308314	9436	Nipsnap1	9.143376587	8.922620079	8.951545636	11736	Rit1	7.730259362	7.777500428	7.169249478	14036	Ticam1	3.894695752	4.763055732	4.440429288
237	2310009B15Rik	6.724446927	7.110792099	7.014013567	2537	Cask	8.407006718	8.496638276	8.549964501	4837	Epx	2.635463543	1.454139986	1.569334612	7137	Igf1r	9.935394798	10.08265038	10.10028783	9437	Nipsnap3b	6.35717936	5.618663794	5.438702875	11737	Rit2	8.812560287	8.642976538	9.290732328	14037	Ticam2	3.059106584	1.407601163	1.980898162
238	2310010J17Rik	6.751553989	6.645984869	6.594677552	2538	Caskin1	8.379944815	8.471142041	8.649063983	4838	Epyc	5.497550477	4.340711034	4.934356404	7138	Igf2	9.019901461	9.369318996	8.47549926	9438	Nisch	9.632965495	9.57621801	9.483806235	11738	Rita1	6.831159774	6.20894341	6.624412622	14038	Ticrr	3.513753224	1.834630348	2.791239718
239	2310011J03Rik	7.248692979	7.326053642	6.979910396	2539	Caskin2	3.975287712	3.426160536	2.560272372	4839	Eqtn	0.042766755	1.885188961	1.570162355	7139	Igf2bp2	2.883034211	3.681209732	1.558337463	9439	Nit1	6.65178253	6.8153931	6.85046257	11739	Rlbp1	7.261173162	7.461133476	7.447730951	14039	Tie1	4.243113862	4.171250076	4.303958147
240	2310015A10Rik	5.476339475	5.589333601	6.15124889	2540	Casp1	4.064253762	3.302154408	1.553657411	4840	Eral1	5.912453712	5.970352942	5.862710546	7140	Igf2bp3	4.475304641	4.936133955	4.300454	9440	Nit2	6.369017251	7.265178012	6.471365308	11740	Rlf	8.584438607	8.537871379	8.583598259	14040	Tifa	5.363361594	5.460132696	5.149720822
241	2310022A10Rik	6.434566816	5.807519469	6.265800915	2541	Casp12	3.967368044	2.125941301	3.304179751	4841	Erap1	5.077460091	4.9695349	4.622628532	7141	Igf2os	1.058808413	2.488364395	1.571010898	9441	Nkain1	8.535867048	8.828369606	8.519134609	11741	Rlim	7.926419685	7.956360324	8.046126381	14041	Tifab	3.649372116	2.405586736	3.891718569
242	2310022B05Rik	8.978344597	8.925135612	9.024423939	2542	Casp2	3.78908375	4.493141528	4.149819271	4842	Eras	2.049750145	1.472284162	1.987392631	7142	Igf2r	7.11376955	7.104879383	7.139956591	9442	Nkain2	7.480935866	7.183633067	7.479950742	11742	Rln1	0.050304132	2.475586738	0.982632415	14042	Tigar	7.547841119	7.496031721	7.88742907
243	2310030G06Rik	3.071538077	3.180559538	3.443171582	2543	Casp3	6.250363703	6.347885328	6.337802057	4843	Erbb2	2.421798891	3.861657838	2.978715806	7143	Igfbp1	3.671488333	1.230075574	2.555643031	9443	Nkain3	6.268714432	6.41380583	5.979547761	11743	Rltpr	8.251064818	8.489540143	8.538534048	14043	Tigd2	7.998619195	8.014402073	7.793704143
244	2310033P09Rik	7.324132692	6.815612011	6.734759741	2544	Casp4	1.638272653	0.880511793	1.571450283	4844	Erbb2ip	8.938722827	9.05968283	9.137414121	7144	Igfbp2	7.787842902	8.268668889	7.012197656	9444	Nkain4	6.926021104	6.741693662	6.922498942	11744	Rmdn1	6.553704923	6.330104662	6.550457973	14044	Tigd3	0.08135123	2.875867038	2.985190552
245	2310034G01Rik	3.877332195	3.85733465	3.439968597	2545	Casp6	4.654907379	3.828255554	4.228705762	4845	Erbb4	3.256165362	3.418461471	3.681248027	7145	Igfbp3	5.657650529	5.003885107	4.500691691	9445	Nkap	7.546841455	7.329452549	7.301837211	11745	Rmdn2	4.98385825	4.692897531	4.934852464	14045	Tigd5	5.748695476	5.728055904	5.53489419
246	2310035C23Rik	7.89266635	7.472994824	7.73506702	2546	Casp7	2.659698116	2.149387457	2.565877897	4846	Erc1	9.413286001	9.301700329	9.189420972	7146	Igfbp4	9.055436706	9.600770302	9.41470171	9446	Nkapl	3.554835249	4.157536704	4.149605769	11746	Rmdn3	8.661074352	8.646371364	8.507497598	14046	Timeless	4.470968099	4.224445135	4.22721627
247	2310036O22Rik	9.402452056	9.512507359	9.38725416	2547	Casp8	3.775155594	4.326536016	3.43916553	4847	Erc2	11.15725839	11.05405608	10.92541733	7147	Igfbp5	10.17604961	10.30351248	10.43639072	9447	Nkd1	6.291053533	6.623790873	6.722270416	11747	Rmi1	6.291235355	6.32814032	6.56535566	14047	Timm10	8.248912172	7.977957051	7.774220781
248	2310039H08Rik	5.210940787	5.681765636	5.504364972	2548	Casp8ap2	8.442336055	8.353202815	8.587483787	4848	Ercc1	5.77567454	5.729399961	4.884739574	7148	Igfbp6	5.604587298	5.772050796	6.089922973	9448	Nkd2	5.982991073	5.44694487	5.564665866	11748	Rmi2	1.664360108	3.208614135	3.155890033	14048	Timm10b	8.091236017	8.241003303	7.85019593

249	2310040G24Rik	3.655800067	2.822339448	3.440794329	2549	Casp9	6.383806372	6.439856697	6.667310147	4849	Ercc2	6.308209767	6.60066349	6.189392055	7149	Igfbp7	8.194228153	8.277423887	7.957020412
250	2310045N01Rik	4.824538337	3.620417444	3.88527272	2550	Casq1	1.6703538	3.044725325	2.303569136	4850	Ercc3	7.488926548	7.237383099	7.50381719	7150	Igfbpl1	4.71689639	5.259822607	2.777266607
251	2310047M10Rik	8.130240601	8.169388453	8.149972095	2551	Casq2	3.424377497	4.601365633	4.148913934	4851	Ercc4	6.148011541	6.314594547	6.550577098	7151	Igflr1	3.779407502	3.530789484	4.151992463
252	2310057M21Rik	6.310725717	6.511758285	6.638696621	2552	Cass4	1.058794542	1.891583639	2.573367458	4852	Ercc5	6.988963372	7.433650007	7.311130844	7152	Igfn1	2.661014711	3.029177517	2.98252375
253	2310061I04Rik	7.573917413	7.849146969	8.07830662	2553	Cast	5.88907745	5.923922237	6.150414092	4853	Ercc6	5.915961519	6.350680774	6.150008757	7153	Ighmbp2	5.500039608	5.552535083	4.933673634
254	2310067B10Rik	5.810180589	6.009408354	6.473318245	2554	Casz1	4.419029858	4.510921154	5.440017133	4854	Ercc6l2	6.367463081	6.660835113	6.534636673	7154	Igip	9.122616657	9.117843677	9.463888354
255	2310068J16Rik	1.0899616	2.175439216	3.308199224	2555	Cat	6.914487954	7.096055816	6.760766304	4855	Ercc8	4.542002493	5.019631182	4.787930836	7155	Iglon5	6.739351898	6.668361584	6.910952042
256	2310069G16Rik	4.393766426	4.010839043	4.441730472	2556	Catip	4.071914778	4.511978018	3.300138045	4856	Erdr1	3.784659305	3.923637005	4.626732075	7156	Igsf1	5.342357778	5.778931309	6.695124731
257	2310075C17Rik	1.643297527	2.710132561	1.986253387	2557	Catsper2	3.08880426	3.289953659	4.070282226	4857	Erf	6.940826189	7.542250422	7.557950628	7157	Igsf10	6.207583389	6.067758813	6.338436084
258	2310081J21Rik	0.02465433	0.899687415	2.797859734	2558	Catsperd	1.64949003	2.203454314	1.568550597	4858	Erg	3.876729442	3.296943609	2.784395848	7158	Igsf11	6.886248724	6.447741212	6.455121472
259	2410002F23Rik	6.887829641	7.328321432	7.024677381	2559	Catsperg2	0.013869475	1.926110041	0.990439848	4859	Ergic1	8.428844924	8.471958531	8.310896634	7159	Igsf21	8.144752304	8.306263019	8.002644841
260	2410004B18Rik	6.383486498	6.777535559	6.001471024	2560	Cav1	5.5086938	5.910849728	5.371330621	4860	Ergic2	8.710191795	8.783185264	8.828629569	7160	Igsf3	6.941133771	7.176312829	7.572924292
261	2410004P03Rik	4.618424719	6.41634526	5.337298532	2561	Cav2	7.296959356	6.956988515	6.957294407	4861	Ergic3	8.683425581	8.559550517	8.251245781	7161	Igsf6	2.381157561	1.864417874	2.570759951
262	2410006H16Rik	8.44212608	8.574155032	8.423002041	2562	Cav3	2.046581104	0.889850076	1.572378969	4862	Erh	7.347096641	7.476552473	7.169560655	7162	Igsf8	9.82359411	9.840916935	9.769526069
263	2410015M20Rik	8.463616512	8.419926553	8.013107608	2563	Cbfa2t2	6.682223832	7.030114629	6.968864728	4863	Eri1	6.573040936	6.734381748	6.63819945	7163	Igsf9	2.875818791	2.155380293	2.30220401
264	2410016O06Rik	7.577820962	7.33144905	7.119387027	2564	Cbfa2t3	8.571319443	8.416213187	8.279184238	4864	Eri2	5.749205886	5.451654782	5.534764624	7164	Igsf9b	0.069685844	2.457647972	2.792936752
265	2410018L13Rik	2.064540015	2.890212649	2.793635783	2565	Cbfb	6.807844488	6.63030102	6.774480853	4865	Eri3	7.523456447	7.728979095	7.579994228	7165	Igtp	3.988190025	3.920914181	4.737906599
266	2410089E03Rik	6.964062116	6.727124276	7.10934361	2566	Cbl	8.283493074	8.028537617	7.928347079	4866	Erich1	6.231605334	6.96803692	6.355045719	7166	Iigp1	4.59648865	4.368219221	3.978985449
267	2410124H12Rik	0.052861586	1.873045359	2.987777722	2567	Cblb	3.244347095	3.311119839	2.298102694	4867	Erich2	3.255233853	5.174435081	2.559295255	7167	Ik	9.902332476	9.827727474	9.942896289
268	2410127L17Rik	6.142787752	5.814611672	5.957655564	2568	Cbll1	7.962622147	7.978614986	7.819149482	4868	Erich3	5.809393911	5.904536246	5.707340428	7168	Ikbip	7.145769444	7.124567298	6.502372733
269	2410131K14Rik	4.944657028	4.509947682	5.814615925	2569	Cbln1	6.001996212	5.117058788	5.22757612	4869	Erich5	3.405164388	4.025888708	3.788592698	7169	Ikbkap	7.497703534	7.468150619	7.447682806
270	2410137M14Rik	1.043259387	0.910025993	0.989105521	2570	Cbln2	7.440397014	7.506772708	8.062417146	4870	Erich6	2.8649417	2.684667388	1.565671118	7170	Ikbkb	7.044312827	6.838353128	6.934451289
271	2500002B13Rik	1.67038203	3.044689108	2.567742515	2571	Cbln4	4.883006129	4.592267627	5.375265242	4871	Erlec1	8.229829726	8.164617199	8.297582122	7171	Ikbke	1.05469937	0.885107058	1.987792859
272	2500004C02Rik	5.405588098	5.820475404	5.838712819	2572	Cbr1	9.45729677	9.122714596	9.286071632	4872	Erlin1	5.571714335	5.388465877	5.471646843	7172	Ikbkg	6.708999219	6.709922293	6.455328724
273	2510002D24Rik	7.722709356	7.430079455	7.638487819	2573	Cbr2	4.773765628	3.098275588	4.889076026	4873	Erlin2	7.271946641	7.729124867	7.363466176	7173	Ikzf1	4.395385488	3.395181117	4.567371027
274	2510003B16Rik	1.65108174	0.846208275	2.571294146	2574	Cbr3	6.648401934	6.482330071	6.264898444	4874	Ermap	1.639948699	1.468579695	1.987011922	7174	Ikzf2	6.207828301	5.661072034	5.622467769
275	2510009E07Rik	8.10836699	8.187406606	8.288538931	2575	Cbr4	6.223551526	5.319214692	5.564537399	4875	Ermard	6.229975362	6.023565594	6.5808813	7175	Ikzf4	6.566360551	6.446905503	5.957071327
276	2510039O18Rik	7.538173588	7.534678162	7.169631076	2576	Cbs	7.480278622	7.34610121	7.139556037	4876	Ermn	10.40918249	10.49720879	10.31982409	7176	Ikzf5	8.501947863	8.438559726	8.269948198
277	2610001J05Rik	8.151530322	7.795497418	8.098986951	2577	Cbwd1	6.367858847	6.736064839	6.694576339	4877	Ermp1	7.665585722	7.618550757	8.00277836	7177	Il10ra	2.879264206	2.421028161	3.569834256
278	2610002M06Rik	7.868359943	7.69422846	7.910800319	2578	Cbx1	7.539406451	7.51100972	7.274319019	4878	Ern1	1.080506999	1.856662958	2.570023048	7178	Il10rb	4.610375412	4.833881077	4.563193853
279	2610005L07Rik	4.396844581	4.371162094	4.567022017	2579	Cbx2	3.08158944	4.039682402	2.561617106	4879	Ero1l	7.089328087	6.872466401	6.80016577	7179	Il11	1.077136766	1.862024622	3.447564428
280	2610008E11Rik	7.199042847	6.922009243	7.415095416	2580	Cbx3	8.727650703	8.592568874	8.572406267	4880	Ero1lb	5.125203263	5.083883923	5.023899113	7180	Il11ra1	8.379560394	8.284590779	8.232556995
281	2610015P09Rik	6.571295669	6.496701343	6.595021104	2581	Cbx4	8.220397125	8.508319508	8.310923987	4881	Erp29	9.361126457	9.427189049	9.186954364	7181	Il11ra2	7.590982835	8.444197237	8.468201413
282	2610020C07Rik	3.540185684	4.182360582	3.681064667	2582	Cbx5	11.38374171	11.25030915	11.2391264	4882	Erp44	6.725685437	7.04025286	6.980220314	7182	Il12a	2.387271431	2.682841446	0.978217448
283	2610020H08Rik	3.388961011	3.178375667	2.981466852	2583	Cbx6	11.9662423	12.00561876	11.86223979	4883	Errfi1	7.238935063	6.557524664	6.438059944	7183	Il12rb2	3.255886372	3.850901135	4.152874767
284	2610027K06Rik	5.567819011	5.118800435	5.56605386	2584	Cbx7	7.329213842	7.37623061	7.715111975	4884	Esam	6.541493278	6.863078973	7.014089345	7184	Il13ra1	4.167743631	4.608360195	4.373306213
285	2610028H24Rik	2.3981962	3.693970664	2.302444524	2585	Cbx8	5.470921814	5.295831639	5.653506859	4885	Esco1	7.548521236	7.551442501	7.5421323	7185	Il13ra2	2.055593993	1.45754925	2.308699123
286	2610035D17Rik	6.014272888	6.246881802	5.836677728	2586	Cby1	7.874897652	8.058866252	7.862631601	4886	Esco2	2.048180462	0.88515978	1.987797476	7186	Il15	2.660990347	3.328656013	2.789009356
287	2610035F20Rik	2.381089631	2.19582407	2.306883465	2587	Cc2d1a	6.100512683	5.968581448	6.150636486	4887	Esd	8.950687596	8.941513103	8.815999312	7187	Il15ra	3.656644007	3.300602098	2.978715251
288	2610037D02Rik	0.013873104	1.507519555	1.575588029	2588	Cc2d1b	5.636780548	5.993460152	5.404999917	4888	Esf1	10.14776694	9.943636981	9.944312986	7188	Il16	5.888929558	6.029083559	5.679477952
289	2610042L04Rik	2.644692942	2.45753425	2.305781699	2589	Cc2d2a	7.251681993	7.399756878	7.735337384	4889	Esm1	2.394776567	3.199855054	2.303334277	7189	Il17d	6.510249336	7.127206235	6.549110141
290	2610044O15Rik8	5.574854316	5.769119261	5.887328929	2590	Ccar1	9.627233002	9.567658387	9.414568174	4890	Espl1	2.865822845	3.352609132	2.305264937	7190	Il17ra	5.958626333	5.796746515	5.300744104
291	2610100L16Rik	5.966462992	6.247507065	6.62484098	2591	Ccar2	6.382548295	6.380302629	6.423466577	4891	Espn	3.654543539	3.865720431	1.554447798	7191	Il17rb	3.378750034	1.39937185	3.307486821
292	2610301B20Rik	8.318727737	8.416427492	8.203928459	2592	Ccbe1	5.128415901	5.851315997	5.149152304	4892	Esr1	4.525042973	4.177680024	3.438098551	7192	Il17rc	2.688671766	4.626709959	3.788218943
293	2610305D13Rik	1.681453815	1.810617385	3.892990711	2593	Ccbl1	5.545059656	6.01026813	5.957428842	4893	Esrp1	1.047989106	2.236140853	0.987893545	7193	Il17rd	3.777976785	4.17952866	3.564591048
294	2610306M01Rik	5.247626861	5.580276802	5.067434442	2594	Ccbl2	4.325501304	4.493008913	3.979015483	4894	Esrra	8.490466692	8.632200524	8.79067407	7194	Il18	7.555801771	7.545990904	7.447542148
295	2610307P16Rik	0.016588015	0.91558156	1.990526866	2595	Ccdc101	5.332719577	5.84383089	5.887782502	4895	Esrrb	3.234441473	2.424363755	2.565923166	7195	Il18bp	3.057504268	2.877497356	2.304753497
296	2610316D01Rik	3.540159403	3.14139579	3.982022817	2596	Ccdc102a	3.081617264	3.954571817	2.979135639	4896	Esrrg	6.439019009	6.358023492	7.047220503	7196	Il1r1	5.290856377	5.48565523	4.620627128
297	2610318N02Rik	2.078745228	3.043125395	1.562781674	2597	Ccdc103	2.900286513	4.632253351	3.439454294	4897	Esyt1	5.131152581	4.104968295	6.090326128	7197	Il1rap	7.168604613	7.212387459	7.208735736
298	2610507B11Rik	9.93074964	9.855818787	9.965839002	2598	Ccdc106	6.939992413	6.995845837	7.381326991	4898	Esyt2	6.795483776	6.717849263	7.002620651	7198	Il1rapl1	5.724763361	5.064991584	6.33926024

9449	Nkiras1	10.20966896	10.04231861	10.08589699	11749	Rmnd1	4.985852155	5.438921475	5.229233983	14049	Timm13	8.544037634	8.463083478	8.61668208
9450	Nkiras2	5.395285485	5.341913289	4.29884809	11750	Rmnd5a	8.375497684	8.101932316	8.53847584	14050	Timm17a	8.717069522	8.592167694	8.75482889
9451	Nkpd1	0.024652393	1.909326158	0.987884356	11751	Rmnd5b	7.79330694	7.451481143	7.337216934	14051	Timm17b	7.618968764	7.573418032	7.6312353
9452	Nkrf	7.047673539	6.910031654	7.292994358	11752	Rmrp	0.07465678	1.848752551	1.56529375	14052	Timm21	4.671384716	5.127375493	5.19021513
9453	Nktr	8.841489768	8.382996379	8.583560837	11753	Rmst	4.255591058	3.492441838	5.627902486	14053	Timm22	12.61390619	12.47118423	12.3647495
9454	Nkx2-1	1.632770506	0.894719424	2.990503418	11754	Rn4.5s	9.714054088	9.975908929	10.2608216	14054	Timm23	6.886272878	6.862889038	6.80037613
9455	Nkx2-2	5.24697523	5.04779977	5.440958677	11755	Rn45s	19.34338635	19.38260603	19.46164815	14055	Timm44	7.454073324	7.121625671	7.34649867
9456	Nkx2-2os	2.384303896	1.428256651	3.57304122	11756	Rnase1	4.47008479	3.719149947	4.736662521	14056	Timm50	5.326796927	5.26541777	5.84008616
9457	Nkx2-9	1.663029449	3.210247028	2.792293778	11757	Rnase4	6.952063732	7.160745222	7.139945619	14057	Timm8a1	6.455402672	6.76273723	6.12888614
9458	Nkx3-1	2.048160024	1.898375138	1.571917046	11758	Rnase6	2.639765474	2.468469113	0.981223791	14058	Timm8b	10.33078828	10.21926146	10.2642292
9459	Nkx6-2	8.753110055	9.117987795	8.841106471	11759	Rnaseh1	3.409542909	3.529636695	4.568563552	14059	Timm9	8.294594571	8.025983416	8.16975042
9460	Nle1	3.789613248	4.949939714	4.440548592	11760	Rnaseh2a	5.986571639	6.109077522	5.934231389	14060	Timmdc1	7.419014073	7.379089991	7.52738845
9461	Nlgn1	9.650103116	9.557480534	9.534597204	11761	Rnaseh2b	6.207355348	6.068209651	6.088772416	14061	Timp1	1.06656302	0.858781257	1.98553687
9462	Nlgn2	8.735440174	8.83795742	9.078196161	11762	Rnaseh2c	7.497457057	7.345505376	7.179354567	14062	Timp2	12.05980186	11.42588069	11.7138565
9463	Nlgn3	4.257841962	4.414834606	5.267889293	11763	Rnasek	10.05630838	10.19591654	9.928332818	14063	Timp3	9.866975312	9.811820499	9.93997839
9464	Nlk	9.747775184	9.697018566	10.19807911	11764	Rnasel	5.287120884	4.780988903	5.473200417	14064	Timp4	7.815371213	7.767894934	7.62355733
9465	Nln	5.890412614	6.026214587	6.002097422	11765	Rnaset2a	7.695790121	7.855305456	7.564636768	14065	Tinagl1	5.480328622	6.303832331	5.43744843
9466	Nlrc4	1.092854801	1.837154801	1.980695065	11766	Rnaset2b	8.600224688	8.702600852	8.422770245	14066	Tinf2	4.988049671	5.126376816	5.40769690
9467	Nlrp1a	0.021980946	1.493087932	0.988488903	11767	Rnd1	5.256469483	4.987305827	6.47423643	14067	Tiparp	8.204472087	7.851930988	8.07793140
9468	Nlrp1b	0.013874466	0.9212747	0.990438581	11768	Rnd3	6.652225024	6.615107746	6.968920422	14068	Tipin	6.50843309	6.929696532	6.98065536
9469	Nlrp3	1.078841542	2.461880933	2.986637898	11769	Rnf10	8.370885609	8.583558112	8.523080494	14069	Tiprl	8.402318395	8.194124824	8.31533383
9470	Nlrp5-ps	3.530500997	3.163884742	3.567409582	11770	Rnf103	9.046143723	8.700559787	9.101601747	14070	Tirap	4.084027806	4.548460305	4.22777022
9471	Nlrp6	2.649240619	2.447408532	2.568590978	11771	Rnf11	8.954019006	8.862466018	9.228079767	14071	Tjap1	5.938648211	6.089057892	6.28473710
9472	Nlrx1	4.158935267	4.250477416	4.152087509	11772	Rnf111	7.056504392	7.029746686	6.799958322	14072	Tjp1	7.644153334	7.767339178	7.96306686
9473	Nmb	4.600055384	4.853577729	4.504111278	11773	Rnf112	9.550192201	9.785541344	9.372220873	14073	Tjp2	8.140979996	8.240040998	8.13486310
9474	Nmd3	6.403323893	6.436825408	6.638574831	11774	Rnf113a1	4.552500509	5.657262493	5.682047581	14074	Tjp3	5.475833249	5.770966223	5.47110218
9475	Nme1	10.39434869	10.29953121	10.16608883	11775	Rnf113a2	5.634306478	5.67330016	5.564907596	14075	Tk1	1.655305329	1.434334147	1.98354092
9476	Nme2	9.629240268	9.555518358	9.499597191	11776	Rnf114	7.402957006	7.352005055	7.61682549	14076	Tk2	7.009987625	6.814404912	7.22864194
9477	Nme3	8.442352891	8.471310042	8.251303204	11777	Rnf115	8.06855477	8.003438248	7.88682527	14077	Tkfc	5.35945649	5.239095067	5.02469805
9478	Nme4	4.82575091	4.29816093	3.884886825	11778	Rnf121	4.241608734	4.097718061	3.887433841	14078	Tkt	8.556626172	8.81029883	8.65594312
9479	Nme5	6.121440824	6.496030674	5.73452186	11779	Rnf122	5.365353622	5.486207037	5.912680133	14079	Tktl1	1.054672886	1.476218451	0.98617605
9480	Nme6	4.397144449	4.6097848	4.440896534	11780	Rnf123	7.455566519	7.525228712	7.447585908	14080	Tktl2	2.646565339	2.181776548	2.30525167
9481	Nme7	12.96321116	12.81968748	12.73548558	11781	Rnf125	3.401373203	3.150779728	2.784041858	14081	Tlcd1	6.011166619	6.107049385	6.0676466
9482	Nme8	0.02199961	1.493056487	1.989542865	11782	Rnf126	7.056778276	7.059016498	6.980089649	14082	Tlcd2	2.092307506	2.137618229	3.30511472
9483	Nmi	4.732922583	5.366013631	5.44027868	11783	Rnf128	8.608206031	8.754284933	8.728232388	14083	Tldc1	1.068481779	0.85446816	2.30806282
9484	Nmnat1	2.665833862	3.020372286	2.564281271	11784	Rnf13	9.637036553	9.531012861	9.683916018	14084	Tle1	8.270799597	8.453104779	8.37659215
9485	Nmnat2	9.537685613	9.441577102	9.344086521	11785	Rnf130	9.194219808	9.188901047	9.22319563	14085	Tle2	1.657924885	3.216530121	2.57002773
9486	Nmnat3	2.66769859	2.638818102	3.151756997	11786	Rnf133	0.037602402	3.731809716	0.985133275	14086	Tle3	7.04379779	6.816235534	6.7744552
9487	Nmral1	6.720741768	6.999118988	6.227558727	11787	Rnf135	5.119205239	4.692930823	4.838241637	14087	Tle4	9.556290308	9.666350426	9.67178055
9488	Nmrk1	4.253626733	4.942566452	4.148601832	11788	Rnf138	5.753687721	5.906396342	6.046818886	14088	Tle6	4.147165135	2.396658476	2.78642244
9489	Nmrk2	0.027248348	0.894825392	0.987310984	11789	Rnf138rt1	2.864975312	3.600726186	0.978605211	14089	Tlk1	9.069278874	9.162382672	9.29071942
9490	Nms	0.016595046	1.921615268	1.574995828	11790	Rnf139	7.572161682	7.852569147	7.463696105	14090	Tlk2	9.186529417	9.166304734	9.27234030
9491	Nmt1	9.535773323	9.420703885	9.382951452	11791	Rnf14	10.77559109	10.61023068	10.37118182	14091	Tln1	7.649953831	7.684562606	7.43921159
9492	Nmt2	8.925646903	8.857805442	8.825501733	11792	Rnf141	3.226741023	1.838897473	2.98496463	14092	Tln2	9.313527901	8.884138371	9.18463965
9493	Nnat	12.03940384	11.09152565	10.73311948	11793	Rnf144a	7.788014278	7.958264671	7.707775235	14093	Tlr12	2.645590902	1.851512893	2.30552758
9494	Nnmt	3.860412071	1.3887448	1.561840258	11794	Rnf144b	5.665646641	5.562649917	5.935017194	14094	Tlr13	2.655118883	3.039939598	1.56212696
9495	Nnt	7.780775072	7.584325134	7.5871007	11795	Rnf145	8.200083889	8.158340077	8.631297759	14095	Tlr2	3.219839661	0.834586189	1.56704966
9496	Noa1	5.000123296	6.150407042	6.003054211	11796	Rnf146	9.847198917	9.927967516	9.960139226	14096	Tlr3	5.939194436	6.18202799	5.78668750
9497	Nob1	5.837294185	5.945523012	6.356089052	11797	Rnf149	8.676445533	8.687086369	8.641808787	14097	Tlr4	1.088528074	2.877472814	1.98147898
9498	Noc2l	7.498244937	7.59415391	7.346104354	11798	Rnf150	8.547771764	8.492829393	8.515327219	14098	Tlr7	3.645906179	3.183056472	3.3053958

299	2610507I01Rik	7.447038838	7.452308285	7.825988508	2599	Ccdc107	6.752268043	7.121210612	6.438838821	4899	Esyt3	2.883707421	2.84367495	2.981447047	7199	Il1rapl2	5.027796306	4.415293424	5.069291489	9499	Noc3l	6.710339223	6.446792698	6.92285762	11799	Rnf152	7.934526173	8.481823472	8.72167118	14099	Tm2d1	7.481412741	7.496267059	7.7485676
300	2610524H06Rik	7.561523581	7.441618673	7.077876891	2600	Ccdc108	2.890438881	3.958635943	3.150616499	4900	Etaa1	6.104309109	6.298913095	6.519926354	7200	Il20rb	4.156730297	3.285290444	3.438785812	9500	Noc4l	6.032128912	5.692280421	6.209944212	11800	Rnf157	9.533298142	9.527049086	9.308674541	14100	Tm2d2	8.193660108	8.286865621	7.92820657
301	2610528A11Rik	3.228702506	3.04461962	2.303552654	2601	Ccdc109b	3.255026767	3.942991488	3.439278677	4901	Etaa1os	3.387972879	3.571903254	3.305105433	7201	Il21r	2.872137287	2.668674513	2.790852829	9501	Nod1	4.526409073	3.840479266	3.146345079	11801	Rnf165	10.03371141	9.668351568	9.308591767	14101	Tm2d3	9.689055621	9.156712246	9.27456829
302	2700029M09Rik	8.665327478	8.589261041	8.634643607	2602	Ccdc110	2.872926161	3.041669352	0.974621085	4902	Etf1	8.672744015	8.585987063	8.491653284	7202	Il25	0.029916464	2.229382505	0.986734408	9502	Nod2	0.027265151	1.484439217	0.987307314	11802	Rnf166	7.157859049	7.167979559	7.488225197	14102	Tm4sf1	5.861979882	6.129387939	5.83787196
303	2700038G22Rik	2.399124265	2.155369934	2.302202819	2603	Ccdc112	8.514295453	8.446347113	8.292874721	4903	Etfa	6.490413602	6.660314895	6.609519687	7203	Il27ra	1.646437631	2.705477047	0.983111461	9503	Nog	4.539335557	3.80865722	5.069742787	11803	Rnf167	6.950177189	6.95829319	7.120049834	14103	Tm4sf5	1.664426379	1.844038811	2.98538350
304	2700046A07Rik	2.046583647	0.889842647	1.988209198	2604	Ccdc113	5.854542469	6.217389016	5.066409311	4904	Etfb	8.851662615	9.073347546	8.526720908	7204	Il2rb	1.052483436	0.889964994	1.57239047	9504	Nol10	7.279663202	6.866191379	7.265499726	11804	Rnf168	8.511129946	8.47763246	8.659561126	14104	Tm6sf1	7.229455801	7.479324593	7.26565645
305	2700046G09Rik	3.558834651	5.257194291	4.681745621	2605	Ccdc114	4.158531291	3.935601205	3.887981807	4905	Etfdh	6.489907318	6.466797477	6.283233525	7205	Il2rg	1.650961105	1.870250124	1.568169148	9505	Nol11	7.76676753	7.792841764	7.645224185	11805	Rnf169	8.794295141	8.609151598	8.803482169	14105	Tm6sf2	1.070247927	1.873044687	1.56854237
306	2700049A03Rik	5.570714713	5.650481783	5.371991624	2606	Ccdc115	7.967327652	7.684112896	7.680522232	4906	Ethe1	6.88864204	6.824079485	7.275171367	7206	Il33	8.506709873	8.395695878	8.546101095	9506	Nol12	6.795684577	7.029766812	6.934262019	11806	Rnf17	2.655001604	2.161045804	0.974225258	14106	Tm7sf2	4.979241087	3.809007491	4.73590758
307	2700054A10Rik	3.87979724	3.145383695	4.30561643	2607	Ccdc117	6.348866611	5.799308638	6.565129199	4907	Etl4	8.587148916	8.501268484	8.427214806	7207	Il34	8.327711907	8.386618523	8.159908213	9507	Nol3	7.428428164	7.280462083	7.337594737	11807	Rnf170	7.80862582	7.755883511	8.067730794	14107	Tm7sf3	7.900602236	8.024116537	7.80644721
308	2700060E02Rik	8.932226269	8.959661797	9.054226733	2608	Ccdc12	7.482209924	7.677827864	7.616624891	4908	Etnk1	9.338314372	9.418659284	9.614839011	7208	Il4ra	5.246374278	4.577991516	5.22884178	9508	Nol4	9.057557446	9.062382725	9.174882311	11808	Rnf181	9.21968857	9.191639077	8.962923352	14108	Tm9sf1	4.791967542	5.89464179	5.76252391
309	2700062C07Rik	5.437118315	5.918131032	5.149032871	2609	Ccdc120	5.196553851	4.986830506	4.736422777	4909	Etnk2	2.110724312	3.29847242	3.983293323	7209	Il6ra	4.464172441	4.375037947	3.979916278	9509	Nol4l	7.335861126	7.258548872	6.968492646	11809	Rnf182	8.595578899	8.607361489	8.04570912	14109	Tm9sf2	9.217608728	9.326022469	9.33097243
310	2700069I18Rik	1.075556273	0.838420113	2.570772101	2610	Ccdc121	2.085392224	3.888308065	3.153651074	4910	Etnppl	6.772673898	6.83969021	7.701357367	7210	Il6st	6.618270694	6.581320609	6.372036555	9510	Nol6	7.887049195	7.631195109	7.659369764	11810	Rnf185	7.488545274	7.33030787	7.199006205	14110	Tm9sf3	10.08352548	10.11608157	10.2397398
311	2700070H01Rik	3.239509953	3.178413022	3.304854513	2611	Ccdc122	4.98850423	5.608520549	5.44087462	4911	Etohd2	7.767751304	7.998646565	8.088736231	7211	Il7r	0.074282876	3.481280842	2.56931304	9511	Nol7	8.90990963	8.774413218	8.892952556	11811	Rnf187	11.16660458	11.07689026	10.87855906	14111	Tm9sf4	8.472561781	8.467742268	8.51537470
312	2700081O15Rik	9.382067863	9.514269034	9.469874005	2612	Ccdc124	7.920235621	8.095922624	8.169982186	4912	Etohi1	3.407995255	4.178477005	3.981515532	7212	Ildr1	1.636474672	2.226170856	0.986178631	9512	Nol8	8.468048326	8.280383474	8.499777404	11812	Rnf19a	8.028489854	7.766880077	7.968704706	14112	Tma16	6.75180005	6.507996934	6.33680545
313	2700094K13Rik	7.43420015	6.613898639	6.957337967	2613	Ccdc125	5.360614495	5.200899613	5.373644206	4913	Ets1	5.604483788	5.591626646	5.652485888	7213	Ildr2	10.02849626	9.641614134	9.782513707	9513	Nol9	6.434846336	6.488893994	6.284160309	11813	Rnf19b	6.877031676	7.403826674	7.218554838	14113	Tma7	8.956672896	8.86043678	8.77436984
314	2700097O09Rik	5.391614202	5.281966014	4.623502974	2614	Ccdc126	5.12482817	4.961983861	5.625665114	4914	Ets2	8.709344859	8.569331529	8.659563167	7214	Ilf2	9.398049105	9.506983921	9.499652603	9514	Nolc1	8.998865463	8.774291798	8.751434881	11814	Rnf2	7.924853308	7.763252488	7.680390254	14114	Tmbim1	6.252740103	6.674797502	6.45571261
315	2700099C18Rik	4.163277916	3.521018498	4.567778097	2615	Ccdc127	7.658660339	7.762487705	8.030150488	4915	Etv1	8.804665856	8.602247289	8.662969599	7215	Ilf3	8.692073927	8.931007484	8.880871721	9515	Nom1	7.642604822	7.794019956	7.495514884	11815	Rnf20	9.085790114	8.870897291	9.035330688	14115	Tmbim4	8.26055942	8.358040243	8.18445698
316	2810001G20Rik	6.252722886	6.482181683	6.77515583	2616	Ccdc129	1.649478363	1.873128895	1.568553621	4916	Etv3	6.147719113	6.441922162	6.045512026	7216	Ilk	9.097089493	9.167355524	8.895801827	9516	Nomo1	8.485295787	8.254756138	8.418814349	11816	Rnf207	6.209932132	6.411724546	6.355397395	14116	Tmbim6	10.10457678	10.3013115	10.3593737
317	2810002D19Rik	4.60125963	4.291293966	4.503804339	2617	Ccdc13	6.0759807	6.170497913	5.405119869	4917	Etv4	5.463621736	5.053282017	5.151057282	7217	Ilkap	8.209366712	8.294728632	8.139897021	9517	Nono	9.037304828	8.917368717	8.91662948	11817	Rnf208	9.794458523	9.562228075	9.7011295	14117	Tmc2	0.008387076	0.933562442	0.99189694
318	2810004N23Rik	8.275127559	8.297790606	8.072574991	2618	Ccdc130	5.937245028	6.091778823	5.438005356	4918	Etv5	8.085490517	7.9621318	8.018994787	7218	Iltifb	1.052517309	0.889890861	1.988213453	9518	Nop10	9.222645106	9.201917675	9.159864052	11818	Rnf214	8.284678004	7.996144226	8.169800193	14118	Tmc3	4.829795987	4.355578898	4.93596518
319	2810006K23Rik	6.739105471	6.991098811	6.838102063	2619	Ccdc132	8.451301219	8.506998295	8.515304348	4919	Etv6	6.384391283	6.360792017	6.826513061	7219	Ilvbl	6.782996681	6.966354078	7.140096157	9519	Nop14	7.918255137	7.683719574	7.616566015	11819	Rnf215	7.144997372	6.946436483	7.160321042	14119	Tmc4	2.386321281	1.422371147	2.30552890
320	2810013P06Rik	7.644099685	7.993719637	8.019072437	2620	Ccdc134	4.332307874	5.180121028	4.438994367	4920	EU599041	1.038059223	0.921251013	1.575585262	7220	Immp1l	7.217667662	6.847991133	6.862522734	9520	Nop16	7.045897174	7.106349387	7.119785366	11820	Rnf216	7.634535745	7.789017702	7.422755039	14120	Tmc6	3.529462539	3.16629534	3.30344346
321	2810021J22Rik	4.541244275	4.891512395	4.888051696	2621	Ccdc136	8.285288812	8.529301723	8.380919157	4921	Eva1a	5.632298203	5.777650629	5.565407618	7221	Immp2l	4.335688851	4.9759198	4.887651345	9521	Nop2	6.189630403	6.36405245	6.023757492	11821	Rnf217	5.721525684	5.594811534	5.148476674	14121	Tmc7	4.995133167	5.258154194	5.3013286
322	2810025M15Rik	6.421157788	6.567168291	6.406029138	2622	Ccdc137	7.716110381	7.428979161	7.439264743	4922	Eva1b	3.977586965	3.28941999	3.439266015	7222	Immt	9.825307433	9.784777955	9.748160298	9522	Nop56	8.941260582	8.893578801	8.790543499	11822	Rnf219	6.899496454	6.534147859	7.046671309	14122	Tmcc1	9.736626291	9.418593471	9.5441136
323	2810029C07Rik	4.549065813	5.305432943	5.266290684	2623	Ccdc138	3.773766471	3.422920573	3.148464611	4923	Eva1c	3.641822992	1.383837703	3.306541698	7223	Imp3	8.777156179	8.748246655	8.55354489	9523	Nop58	9.170172974	8.749507703	8.880787384	11823	Rnf220	9.874672864	9.635311157	9.748192809	14123	Tmcc2	10.05319802	9.881019775	9.76786557
324	2810032G03Rik	4.098597072	4.785894894	4.98004735	2624	Ccdc14	4.239473184	3.412401646	4.442888671	4924	Evc	5.037086613	5.121431373	5.108994487	7224	Imp4	6.383193549	6.716657971	6.227769167	9524	Nop9	6.457733631	6.507202051	6.721569568	11824	Rnf223	1.649473358	1.873138609	2.307775572	14124	Tmcc3	9.311970084	9.363206181	9.34409123
325	2810047C21Rik1	0.016593623	1.921617564	0.989762129	2625	Ccdc141	6.442200005	7.191557053	6.910818093	4925	Evc2	5.986658075	5.527657631	6.30300153	7225	Impa1	8.922701935	8.710486998	8.8254576	9525	Nos1	8.475684996	8.248254507	7.892610298	11825	Rnf225	2.403130399	2.421152052	3.153191781	14125	Tmco1	9.154608798	9.068828243	9.19191766
326	2810049E08Rik	3.975588788	3.656755282	3.566008681	2626	Ccdc142	1.068431362	1.450677879	1.98518605	4926	Evi2a	7.402677003	7.772494278	7.447617036	7226	Impa2	2.676104893	3.664904693	3.3027514	9526	Nos1ap	9.695421482	9.991974743	9.968738101	11826	Rnf24	7.321403542	7.804581383	7.980332818	14126	Tmco2	1.077093209	3.817166402	1.56707904
327	2810403A07Rik	7.651381812	7.826533858	7.874867217	2627	Ccdc146	4.395929185	5.582620358	3.297607172	4927	Evi2b	1.653747121	1.864837846	2.570799963	7227	Impact	10.47606967	10.20364163	10.50649804	9527	Nos3	4.831490591	4.845728837	4.623911617	11827	Rnf25	2.398239612	3.03744732	2.7900925	14127	Tmco3	7.034839121	7.152285352	7.09902933
328	2810403D21Rik	2.669705053	3.963194316	1.974590092	2628	Ccdc148	8.317381661	7.961863767	7.880742076	4928	Evi5	9.138404393	9.009924048	9.139892989	7228	Impad1	9.510880946	9.317119311	9.551788743	9528	Nosip	7.554922491	7.533627917	7.088050341	11828	Rnf26	5.861599274	5.717327531	6.046716719	14128	Tmco4	2.084504659	3.333496927	3.89335838
329	2810408I11Rik	2.865796504	3.481219824	1.982064525	2629	Ccdc149	7.58908574	7.689315964	7.734901549	4929	Evi5l	5.129531664	5.22444041	5.653253636	7229	Impdh1	6.58792136	6.647074983	6.694584379	9529	Nostrin	5.2009324	4.844088179	4.564535513	11829	Rnf31	5.16070107	5.09785407	4.502563519	14129	Tmco5	4.163073591	5.084157982	3.7867042
330	2810410L24Rik	2.890129384	2.8301895	3.441571296	2630	Ccdc15	4.341099278	5.610596203	4.979972752	4930	Evl	7.951175725	7.971979977	8.169998554	7230	Impdh2	5.955215956	5.543164725	5.406390954	9530	Notch1	6.738832746	6.616874735	6.94619834	11830	Rnf32	6.166966799	6.414867435	6.265916385	14130	Tmco5b	0.011137435	0.927255185	0.99114829
331	2810417H13Rik	4.709376391	4.314106304	4.151357836	2631	Ccdc150	1.634660054	1.901870429	1.572381301	4931	Evpl	2.437196866	3.933241764	4.230250754	7231	Impg1	2.054114596	1.461296218	0.984104454	9531	Notch2	5.966675438	6.331347691	6.337769504	11831	Rnf34	8.388877497	8.349335446	8.422965563	14131	Tmco6	6.473129902	6.699553683	6.50356143
332	2810428I15Rik	7.962105537	7.861268723	7.623658733	2632	Ccdc151	3.989657261	3.729200628	5.154081043	4932	Ewsr1	7.717387121	7.756726631	7.910926787	7232	Impg2	1.05468533	0.88513789	2.310421538	9532	Notch3	4.245049139	2.332868752	4.984124662	11832	Rnf38	6.487661487	6.500591686	6.189196522	14132	Tmed1	5.96398799	6.217146815	5.78657685
333	2810429I04Rik	2.657267658	3.036018636	2.566509316	2633	Ccdc152	1.100091227	2.86484133	3.445024359	4933	Exd1	2.368767476	0.880625685	1.571461539	7233	Ina	10.02758782	10.09988203	10.1066861	9533	Notch4	4.649978972	3.840796102	3.299353401	11833	Rnf4	7.455739369	7.566809588	7.380710642	14133	Tmed10	8.910851145	9.18013479	9.12973237
334	2810433D01Rik	7.665901549	7.522007426	7.793849156	2634	Ccdc153	6.079012719	6.867265433	5.786799996	4934	Exd2	6.400243747	6.316048864	6.472563699	7234	Inadl	4.55885274	4.858829396	5.958476369	9534	Notum	1.08860995	1.41337823	2.985367249	11834	Rnf40	6.78011211	6.549567496	6.652334532	14134	Tmed2	8.225782634	8.475246369	8.53088365
335	2810442N19Rik	3.050947426	0.838470223	2.570775436	2635	Ccdc155	2.876541887	3.462833726	3.153804441	4935	Exo5	5.85387035	5.039678663	5.864613804	7235	Inafm1	6.755524302	6.845815321	7.228242125	9535	Nov	7.68021406	6.43300338	6.734991226	11835	Rnf41	8.11982535	7.990891351	8.451910739	14135	Tmed3	6.605958875	7.041369768	6.87496214
336	2810454H06Rik	4.667324983	5.13478369	4.787425375	2636	Ccdc157	6.721804025	6.207616375	6.748860938	4936	Exoc1	6.726192873	7.116883108	6.934199169	7236	Inafm2	7.385513762	7.473338077	7.687517165	9536	Nova1	8.147571468	7.844717237	8.797334907	11836	Rnf43	3.062569333	2.436458264	1.562494501	14136	Tmed4	7.532035388	7.606373943	7.54217912
337	2810459M11Rik	6.569159065	6.54391788	6.283821865	2637	Ccdc158	2.652455841	3.98656904	1.563124046	4937	Exoc2	6.491763469	6.6577093	6.991897593	7237	Inca1	4.398439697	4.367896637	4.228110196	9537	Nova2	8.811031764	8.727657319	8.648947482	11837	Rnf44	8.535927991	8.758015143	8.704627969	14137	Tmed5	7.481176322	7.452681174	7.34615340
338	2810468N07Rik	7.63328293	7.791434575	7.319790829	2638	Ccdc159	2.648237649	3.596706321	1.564689472	4938	Exoc3	9.911732269	9.840694294	9.901855116	7238	Incenp	6.167454102	6.178980405	6.625157921	9538	Nox1	8.709615666	8.516654281	8.697724813	11838	Rnf5	8.892446257	9.370790011	9.59843086	14138	Tmed7	7.732616165	8.053358465	7.83162149
339	2810474O19Rik	8.328165368	8.212572061	8.292910997	2639	Ccdc160	4.882490281	4.142981916	4.503231261	4939	Exoc3l	2.658031485	3.333555885	2.789720744	7239	Inf2	7.809348823	8.15224126	8.237662024	9539	Noxo1	4.527012174	4.315541659	3.299090755	11839	Rnf6	9.34732294	9.311437277	9.399978165	14139	Tmed8	7.011013128	7.068038904	7.08872875
340	2900005J15Rik	1.669246586	3.472925994	0.975780942	2640	Ccdc163	2.688910965	3.282470762	3.300174461	4940	Exoc3l4	2.662347732	3.326408127	2.982223676	7240	Ing1	9.489421386	9.157119544	9.324323873	9540	Noxred1	0.024646924	1.909334083	0.987885578	11840	Rnf7	8.713652279	8.744724256	8.590890385	14140	Tmed9	9.845221953	9.770209744	9.61111777
341	2900008C10Rik	4.792046407	5.359503262	5.503840073	2641	Ccdc166	4.708542288	3.274175196	4.442306771	4941	Exoc4	8.274657337	8.187083716	8.19437986	7241	Ing2	8.642499776	8.639274665	8.64183566	9541	Npas1	4.316822029	3.934588915	3.438081534	11841	Rnf8	7.687261112	7.465820047	7.430965762	14141	Tmeff1	9.078090833	9.24519042	9.23738054
342	2900009J06Rik	4.468705043	4.429247013	4.566370972	2642	Ccdc167	8.141595245	8.446888895	8.435524865	4942	Exoc5	8.86515472	8.829232181	9.019018161	7242	Ing3	7.564270694	7.620020586	7.714937203	9542	Npas2	6.050787736	5.622076717	5.863785294	11842	Rnf8-cmtr1	9.197087954	9.299403289	9.370203411	14142	Tmeff2	9.26993851	9.19146176	9.0106053
343	2900011O08Rik	9.982716923	9.883721092	9.901849637	2643	Ccdc169	2.048179094	2.226155046	1.987797829	4943	Exoc6	6.308539445	6.586353165	6.519888711	7243	Ing4	7.437205107	7.385768745	7.310911698	9543	Npas3	7.862364683	7.79635142	8.150141119	11843	Rnft1	5.08755942	5.823068587	5.652876757	14143	Tmem100	6.848187798	6.719302804	6.87499360
344	2900026A02Rik	8.748119801	8.308986106	8.203815407	2644	Ccdc17	2.433244706	3.646939345	4.152469925	4944	Exoc6b	8.68345587	8.467128779	8.414558555	7244	Ing5	6.618590192	6.451450851	6.923446136	9544	Npas4	5.11150352	4.077090256	5.305822047	11844	Rnft2	6.187593963	6.006399695	6.002168796	14144	Tmem101	6.795109957	6.948289306	6.95753339
345	2900052N01Rik	8.074054715	7.779148356	7.922526958	2645	Ccdc170	2.864069602	3.355729855	0.979047963	4945	Exoc7	7.766168114	7.715474695	7.761553589	7245	Inha	4.535689843	4.296824462	4.682073783	9545	Npat	7.665569457	7.644868238	7.774539453	11845	Rngtt	8.274509523	8.187360078	7.910512457	14145	Tmem104	5.568517684	5.486817925	5.22760696
346	2900055J20Rik	6.572003106	6.620853945	6.666808093	2646	Ccdc171	5.430782975	5.373780939	5.190355786	4946	Exoc8	7.374195258	7.167799057	7.565489599	7246	Inhba	5.542456803	5.695283695	5.6803101	9546	Npb	3.638982765	3.0430638	0.974983816	11846	Rnh1	7.135007595	7.170821248	6.957120575	14146	Tmem106a	4.060043125	3.012079012	1.55627799
347	2900060B14Rik	13.81978222	14.00804419	14.2452987	2647	Ccdc173	6.540159238	6.784433831	6.721838491	4947	Exog	6.455027416	6.328159755	6.503806776	7247	Inhbb	5.559585136	4.21073831	4.83849888	9547	Npbwr1	1.646451581	0.858816594	2.308389442	11847	Rnls	3.243361571	3.56282654	3.303906672	14147	Tmem106b	10.0274631	9.847183338	10.0753989
348	2900076A07Rik	4.472128909	4.541999112	4.226908597	2648	Ccdc174	8.397919786	8.345341087	8.427108196	4948	Exosc1	7.3354331	6.955990492	6.862334872	7248	Inhbe	1.038059223	0.921251013	1.575585262	9548	Npc1	8.760127955	8.885492761	8.916714408	11848	Rnmt	8.755865697	8.462883653	8.602070992	14148	Tmem106c	5.837770954	6.477013352	6.04616186

349	2900079G21Rik	5.828638737	5.653747378	5.265090422	2649	Ccdc175	7.760424486	7.882731377	7.86266842	4949	Exosc10	6.73809192	6.644783661	6.787613467	7249	Inip	6.058446714	6.085201774	6.456660592	9549	Npc1l1	0.040260363	1.888323394	1.570579893	11849	Rnmtl1	5.508460124	5.645826994	5.594603523	14149	Tmem107	6.248651449	6.561791165	6.024174409
350	2900092D14Rik	7.211480725	7.539372344	7.794274887	2650	Ccdc176	6.438743733	7.430123282	6.609609686	4950	Exosc2	6.01290555	6.300637057	6.372928311	7250	Inmt	3.90607312	5.671531155	3.674487157	9550	Npc2	9.722495522	9.825633062	9.545991689	11850	Rnpc3	6.988765453	6.974695833	7.265586087	14150	Tmem108	5.870429767	6.994388112	6.388858612
351	2900097C17Rik	11.43922997	11.28523778	11.50208002	2651	Ccdc177	8.804795879	8.978854886	8.825557434	4951	Exosc3	5.164400264	4.402770793	5.06794911	7251	Ino80	7.602218872	7.541113074	7.077593601	9551	Npcd	11.08921851	10.70538313	10.32200294	11851	Rnpep	6.622119897	6.802276114	7.057153629	14151	Tmem109	8.173303859	8.281667093	7.904498079
352	3000002C10Rik	1.675015707	2.432811098	2.983705286	2652	Ccdc178	0.024610907	2.92412767	1.57337821	4952	Exosc4	6.934153332	6.375080066	6.188682832	7252	Ino80b	5.959735569	6.328155585	5.707942133	9552	Npdc1	9.29241905	9.537554373	9.162314859	11852	Rnpepl1	5.327057186	5.547064298	4.368198846	14152	Tmem11	7.974443879	7.958363129	7.557082916
353	3010001F23Rik	2.080767502	3.1969061	2.790443885	2653	Ccdc18	5.205927011	4.736808856	4.886539633	4953	Exosc5	5.169952043	5.363508444	5.372611778	7253	Ino80c	8.028690814	8.042969864	8.002379301	9553	Npepl1	4.268207675	5.711214582	5.149894242	11853	Rnps1	7.679920529	7.374436402	7.602058234	14153	Tmem110	6.103866575	6.159213318	6.372819305
354	3010026O09Rik	5.807133056	6.03565655	5.533962274	2654	Ccdc180	2.882391264	3.572009965	2.300175967	4954	Exosc6	6.693514119	6.342137035	6.283159358	7254	Ino80d	7.612868729	7.803909124	7.934385683	9554	Npepps	10.09310452	10.05219733	10.07010955	11854	Rnu11	1.130967267	3.441271182	2.980131958	14154	Tmem114	4.5382428	5.757361566	3.783810262
355	3100003L05Rik	1.048024277	0.899692495	1.989085389	2655	Ccdc181	7.956180025	7.95846741	7.806560531	4955	Exosc7	6.822059458	6.948345659	7.067942437	7255	Ino80dos	3.982525708	3.744833582	4.069025829	9555	Nphp1	6.522623669	6.526034487	6.519347568	11855	Robo1	6.726162675	6.847843548	7.364517108	14155	Tmem115	6.538189136	6.650657311	6.472109239
356	3110001I22Rik	3.250080418	3.299537852	3.789958253	2656	Ccdc183	1.064586352	3.232785252	0.983615642	4956	Exosc8	7.488916348	7.431512285	7.265267981	7256	Ino80e	7.090744859	6.935422896	7.024502139	9556	Nphp3	3.541545343	3.749688435	4.443027426	11856	Robo2	8.695027075	8.645333088	8.862813652	14156	Tmem116	1.675060295	2.432741086	3.306855122
357	3110002H16Rik	6.913121643	6.742471173	7.150455349	2657	Ccdc184	6.6227937	6.544857699	7.247312274	4957	Exosc9	8.47150759	8.05137227	8.144894716	7257	Inpp1	6.288748267	6.473298904	6.423587772	9557	Nphp4	3.777609126	4.103655918	3.98173797	11857	Robo3	4.398698023	4.606786114	4.889757619	14157	Tmem117	4.336139156	4.587678563	5.068839288
358	3110009E18Rik	4.239750148	3.53241029	3.787934674	2658	Ccdc186	8.572552652	8.551682685	8.741638262	4958	Exph5	6.040562372	6.187874253	7.339158524	7258	Inpp4a	7.92004009	7.730264553	8.377026141	9558	Nphs1	2.380007908	1.440659575	0.98120694	11858	Robo4	3.537107527	3.543674768	4.305884871	14158	Tmem119	7.495654587	7.132768587	7.109265656
359	3110021A11Rik	1.123594477	3.016921333	3.791837546	2659	Ccdc22	6.225245004	6.341672285	6.170885835	4959	Ext1	7.969221294	7.987901791	8.284136062	7259	Inpp4b	6.037583591	6.471191156	6.087976751	9559	Npl	1.689565023	2.6426453	2.981432379	11859	Rock1	9.729855846	9.89217192	9.540228966	14159	Tmem120a	6.097843963	5.88692285	5.471022772
360	3110021N24Rik	4.398762296	3.253234982	5.269528867	2660	Ccdc23	6.102659996	5.877296036	6.209171115	4960	Ext2	6.328743211	6.77656302	6.535039329	7260	Inpp5a	8.086790639	8.362460244	8.381020352	9560	Nploc4	7.199631659	7.273218669	7.001949677	11860	Rock2	11.8362819	11.7211485	11.81563905	14160	Tmem120b	4.597898103	4.155691906	4.14937842
361	3110035E14Rik	11.01337179	11.0711946	11.12597126	2661	Ccdc24	7.08275272	7.361096311	7.414663209	4961	Extl1	7.12266712	6.926570086	6.850355198	7261	Inpp5b	6.147263961	6.332501383	6.283998655	9561	Npm1	10.02751481	10.09175216	10.03796891	11861	Rogdi	8.746034008	9.131944951	8.62010027	14161	Tmem121	8.30857093	8.24825266	7.892610072
362	3110039I08Rik	7.113709445	6.799185333	7.150043585	2662	Ccdc25	9.203742009	8.887848621	8.809674642	4962	Extl2	8.808649777	8.826299474	8.862625941	7262	Inpp5d	3.416353137	4.49832479	4.841386269	9562	Npm2	2.372757598	2.483086924	1.570159567	11862	Rom1	4.730697164	5.401875603	5.024146167	14162	Tmem123	6.925988673	7.12640642	6.623188519
363	3110039M20Rik	7.487186564	7.365020644	7.119568094	2663	Ccdc28a	7.230997431	7.609479019	6.886051735	4963	Extl3	8.630878961	8.444722396	8.568751042	7263	Inpp5e	7.581138925	7.872976813	7.748227712	9563	Npm3	5.601062439	5.392365386	5.108142467	11863	Romo1	9.796364132	9.734266122	9.782534188	14163	Tmem125	5.574797874	6.054861491	5.911263443
364	3110040N11Rik	5.826875262	5.518786558	5.227943845	2664	Ccdc28b	6.681429213	7.10025386	6.564439868	4964	Eya1	6.183401614	5.930294166	5.623478394	7264	Inpp5f	8.999736684	9.109233219	9.232714727	9564	Npm3-ps1	5.804172813	5.221946718	5.301185163	11864	Ropn1	1.130223941	5.149623523	2.786540927	14164	Tmem126a	7.437691021	7.369130843	7.463943994
365	3110043O21Rik	7.125300445	6.995106886	7.406658572	2665	Ccdc3	6.692313791	6.966952109	6.354907288	4965	Eya2	4.651490951	3.524102642	4.568059237	7265	Inpp5j	6.807465518	6.733264609	6.748283445	9565	Npnt	6.712358721	7.115851672	6.837652249	11865	Ropn1l	1.110994978	4.139622747	3.443857557	14165	Tmem126b	5.437882771	5.422726324	5.595303447
366	3110045C21Rik	4.156341405	3.649484383	1.968986157	2666	Ccdc30	6.095122906	5.291132614	5.595037052	4966	Eya3	6.507954084	6.783224792	6.721685743	7266	Inpp5k	6.604352883	6.684328584	6.422420491	9566	Nppa	1.652414171	1.440838575	2.307179411	11866	Ror1	2.382209754	2.463911841	2.570510787	14166	Tmem127	8.551842136	8.376507146	8.523086777
367	3110052M02Rik	5.546916257	5.785544552	6.695918427	2667	Ccdc32	8.934837394	8.849694134	8.84734443	4967	Eya4	0.083502269	2.173416236	3.155524992	7267	Inppl1	7.337332142	7.370794965	7.572967671	9567	Nppc	5.537818329	5.513658321	5.736058778	11867	Ror2	2.65181893	2.168896694	3.307660206	14167	Tmem128	6.653928786	6.92327074	7.364250384
368	3110056K07Rik	3.786425134	3.393866855	4.228781382	2668	Ccdc33	3.239547458	2.642914184	3.152174467	4968	Ezh1	7.723916357	7.715185827	7.761516566	7268	Ins2	0.064947609	3.487169238	0.979901309	9568	Npr1	3.87592097	4.116319485	2.978488706	11868	Rora	9.8226807	9.906962063	10.74992684	14168	Tmem129	5.213100181	5.624845131	5.264800395
369	3110057O12Rik	4.596681725	4.30129176	4.149718789	2669	Ccdc34	8.769623075	8.545518717	8.25122123	4969	Ezh2	7.642900247	7.739557652	7.875093473	7269	Insc	4.171921259	4.544415039	4.681798867	9569	Npr2	7.453861494	6.953024065	7.504045799	11869	Rorb	9.574860465	9.445749343	10.34647734	14169	Tmem130	9.868399616	9.651073159	9.9573129
370	3110062M04Rik	5.575661568	6.308764593	5.438116512	2670	Ccdc36	12.76357227	12.61252241	12.50279298	4970	Ezr	8.654128748	9.04967275	8.475710805	7270	Insig1	7.170342709	7.481372036	7.869241014	9570	Npr3	7.705538056	6.996543744	7.002215849	11870	Rorc	3.235635406	3.186586772	3.153186163	14170	Tmem131	6.035662899	6.215191749	6.355703878
371	3110070M22Rik	4.147120227	2.119231342	2.786439906	2671	Ccdc37	4.530309166	4.770452192	4.150630453	4971	F10	3.528456721	0.705050823	3.683872323	7271	Insig2	7.931662673	7.63692371	7.728070865	9571	Nprl2	7.102009733	6.924278661	7.150221807	11871	Rp2h	4.247780813	4.434297481	4.067233243	14171	Tmem132a	7.980436683	8.20803191	7.934363701
372	3110079O15Rik	2.381067356	1.437616785	2.306889227	2672	Ccdc38	7.189841769	7.211751498	7.3895366	4972	F11r	5.985068485	6.274996343	5.335848991	7272	Insl5	0.060154467	0.838594655	3.447752294	9572	Nprl3	6.754375727	7.260901823	6.799928424	11872	Rp9	8.748768978	8.569287551	8.656031529	14172	Tmem132b	8.650977431	8.904311009	8.996906649
373	3110082I17Rik	6.629207456	6.430246176	5.733765306	2673	Ccdc39	6.795946594	6.998838997	7.099268749	4973	F12	3.396919923	0.682234557	2.979173868	7273	Insl6	2.64277171	1.859213299	1.983240133	9573	Npsr1	3.068534493	3.328647948	3.153181916	11873	Rpa1	7.497190832	7.321712408	7.067000497	14173	Tmem132c	4.100956289	4.196076806	5.536249656
374	3110082J24Rik	4.168827646	4.491565434	3.885198948	2674	Ccdc40	3.234418125	3.331131418	2.789368775	4974	F13a1	5.197253448	4.292212748	4.065655381	7274	Insm1	4.883384126	4.845158327	4.438551173	9574	Nptn	11.31842427	11.38806708	11.41252647	11874	Rpa2	7.249551011	7.036174426	7.119525109	14174	Tmem132d	3.547074507	4.724278303	4.151224987
375	3300002I08Rik	2.870659777	1.402483901	2.79123525	2675	Ccdc42	2.064574536	1.861974065	1.9835601	4975	F13b	0.03768811	0.876102423	1.571015832	7275	Insm2	2.876617924	2.427455992	1.978341848	9575	Nptx1	10.76529741	10.97452115	10.71553376	11875	Rpa3	4.404876678	4.479728818	4.37155131	14175	Tmem132e	4.833258365	3.596004096	5.442182691
376	3300002P13Rik	7.571436321	7.614944582	7.319670366	2676	Ccdc42b	3.211811618	1.464665677	0.984574123	4976	F2	3.416918693	2.566824298	3.678470337	7276	Insr	6.681602943	7.174538653	7.002767764	9576	Nptx2	6.370461597	7.192260329	7.415736461	11876	Rpain	8.24535372	8.204034694	8.306503466	14176	Tmem134	8.224997886	8.530529491	8.056672955
377	3632451O06Rik	7.411969142	7.698233431	7.638423362	2677	Ccdc43	7.685966493	7.588515507	7.30177742	4977	F2r	5.545483809	6.029850622	5.934173382	7277	Ints1	6.454311485	6.329556116	6.337543925	9577	Nptxr	11.08500994	10.70074377	10.31531806	11877	Rpap1	5.627726616	5.521611795	5.302590845	14177	Tmem135	8.039001826	7.657100225	7.761370901
378	3632454L22Rik	3.662197143	3.287788103	4.152874798	2678	Ccdc47	9.782773499	9.735458755	9.919606394	4978	F2rl2	2.048155458	2.49400965	1.571918383	7278	Ints10	6.34663179	6.263671542	6.045391212	9578	Npw	2.044901579	1.905549536	1.572869542	11878	Rpap2	6.631764472	6.470501782	6.283703288	14178	Tmem136	6.288520154	6.103335665	6.169840035
379	3830403N18Rik	0.027304955	0.894720392	0.987298623	2679	Ccdc50	8.54433466	8.390920817	8.853612058	4979	F3	6.543530457	6.741679892	6.837785845	7279	Ints12	5.083162361	5.364994498	5.406867434	9579	Npy	9.617596051	9.688597388	9.678760568	11879	Rpap3	7.056786535	6.824796285	7.218947187	14179	Tmem138	5.125093286	5.335652131	5.373205428
380	3830406C13Rik	7.886858849	7.577999018	7.862851889	2680	Ccdc51	4.664627958	4.891262804	4.981496448	4980	F420014N23Rik	8.723550648	8.616849804	8.594798276	7280	Ints2	6.308880188	6.19381767	6.456222196	9580	Npy1r	5.96477181	5.985076423	6.247216015	11880	Rpe	6.366399114	6.913083184	6.108439697	14180	Tmem140	3.992178254	4.549673995	3.785037179
381	3930402G23Rik	1.068438838	0.854564866	2.988020284	2681	Ccdc53	7.308432423	7.16859099	7.415019931	4981	F5	7.221689846	8.517174753	6.437377218	7281	Ints3	6.833157696	6.551338769	6.623922156	9581	Npy2r	7.479948653	7.555781678	6.501961252	11881	Rpe65	3.649741589	2.637488195	3.568472194	14181	Tmem141	7.134625707	7.125372908	7.265919161
382	4430402I18Rik	4.723455182	5.247935187	5.025835506	2682	Ccdc54	2.064560436	3.360495087	1.983563755	4982	F630111L10Rik	2.383281801	2.69036196	1.566682381	7282	Ints4	7.702695954	7.798678295	7.84434029	9582	Npy5r	4.324694643	4.233949949	3.297102986	11882	Rpf1	6.170183379	6.155336375	6.227552497	14182	Tmem143	5.778854249	5.911200823	5.735247769
383	4632404H12Rik	4.932187144	4.980639372	4.839138898	2683	Ccdc55	8.871451161	8.624812874	8.748145345	4983	F730043M19Rik	5.210313119	4.869979628	5.440157255	7283	Ints5	6.69475808	6.591787933	6.825889621	9583	Nqo1	4.933331953	4.797545276	5.151992912	11883	Rpf2	7.63511877	7.578715909	7.74169277	14183	Tmem144	5.084115009	5.573397232	5.227629269
384	4632415L05Rik	7.780824149	7.792472552	7.963253524	2684	Ccdc57	3.650262327	3.014474874	3.568337904	4984	F8	5.20645351	4.922730825	5.190195714	7284	Ints6	6.509010271	6.669601669	6.748205493	9584	Nqo2	5.66467717	5.507487399	5.503181559	11884	Rpgr	6.189543583	6.176499118	6.406724786	14184	Tmem145	6.506479707	6.359075134	6.534726174
385	4632428N05Rik	6.348691871	6.78605406	6.487813383	2685	Ccdc58	6.122297922	6.128362341	5.812567292	4985	F8a	6.418238822	6.530602081	5.909785097	7285	Ints7	6.793458226	6.897367591	6.99195346	9585	Nr1d1	8.328750249	8.46034147	8.677198573	11885	Rpgrip1	7.788713333	8.357088658	8.093905714	14185	Tmem147	8.838169104	8.664497782	8.579844915
386	4732456N10Rik	0.029943	2.229349619	1.988212011	2686	Ccdc59	8.097259557	8.100152725	8.218547163	4986	F9	1.686357362	3.024360576	2.981930181	7286	Ints8	7.392612796	7.003887891	7.179490095	9586	Nr1d2	8.710545851	8.700792632	9.253987634	11886	Rpgrip1l	7.47241999	7.384482535	7.609661057	14186	Tmem14a	9.502374772	9.390541283	9.52502066
387	4732471J01Rik	5.319870883	4.792400136	4.934833813	2687	Ccdc6	7.736832316	7.553636038	7.389319437	4987	F930015N05Rik	1.054699106	0.885107638	2.574016159	7287	Ints9	6.18984288	6.754347693	6.610246343	9587	Nr1h2	7.42731909	7.340238349	7.077875988	11887	Rph3a	10.62563631	10.85689264	10.8921544	14187	Tmem14c	8.437719302	8.434731278	8.310876604
388	4732491K20Rik	4.083274494	4.080198108	4.682441599	2688	Ccdc60	1.048049236	0.899638215	1.573364882	4988	Fa2h	7.082961592	7.660908263	7.337408492	7288	Intu	4.715029886	4.661437535	4.789907259	9588	Nr1h3	3.665474418	3.532478672	4.50719875	11888	Rph3al	3.775325786	4.027240145	3.681347825	14188	Tmem150a	8.438101634	8.617135535	8.397803442
389	4831407H17Rik	1.650915691	1.870338313	2.307483027	2689	Ccdc61	4.663847152	4.593322083	4.438790262	4989	Faah	7.973917413	7.745162981	7.557205437	7289	Invs	6.382202062	5.896958696	6.302217785	9589	Nr1h4	2.043212689	0.899610783	0.987874885	11889	Rpia	5.335220876	5.693407808	5.980602587	14189	Tmem150b	3.064657088	3.037350327	2.56669878
390	4831440E17Rik	5.538460114	5.541191474	5.40614048	2690	Ccdc62	2.400651821	3.033427505	2.30180476	4990	Faap100	6.12245836	6.030236002	6.089015777	7290	Ip6k1	9.365751181	9.138760025	9.18206034	9590	Nr1i3	1.073699593	2.198441964	2.307188208	11890	Rpl10	10.10701565	10.16452832	9.954365734	14190	Tmem150c	8.588389311	8.462648076	8.50346736
391	4833411C07Rik	2.063414088	2.195806185	0.980764667	2691	Ccdc64	2.419718746	3.768022046	2.785384913	4991	Faap20	6.365919522	6.424030824	6.389447004	7291	Ip6k2	8.568403773	8.575965891	8.232345577	9591	Nr2c1	4.833036277	4.532254574	4.935176806	11891	Rpl10a	9.634580383	9.40981151	9.249057618	14191	Tmem150cos	7.268297149	6.7526662	6.693580131
392	4833412C05Rik	2.379943102	1.440845372	2.571047519	2692	Ccdc64b	3.663811724	4.104723756	4.443162664	4992	Faap24	5.931334796	5.542144646	5.788654242	7292	Ipcef1	7.963092469	8.148023447	8.377069299	9592	Nr2c2	10.11541396	10.11151701	10.33208	11892	Rpl11	8.472646294	8.449122543	8.149654364	14192	Tmem151a	8.630731008	8.616892074	8.439390131
393	4833415N18Rik	2.090489481	1.802989797	2.982093017	2693	Ccdc65	5.498885693	5.165504694	4.933989762	4993	Fabp1	4.259387474	1.979799297	5.068923361	7293	Ipmk	6.698419175	7.017066663	6.886525638	9593	Nr2c2ap	7.716500459	7.592624572	7.471644347	11893	Rpl12	7.731600257	7.768978186	7.714674421	14193	Tmem151b	9.736534134	9.554011142	9.546063775
394	4833417C18Rik	0.024651094	1.488656677	1.989085484	2694	Ccdc66	7.657579675	7.551638978	7.594702685	4994	Fabp3	9.074530431	8.842981211	8.991251287	7294	Ipo11	7.289386483	6.854931062	7.439819083	9594	Nr2e1	6.264944801	5.791321681	6.002929318	11894	Rpl13	11.70165292	11.59937346	11.38190327	14194	Tmem158	9.020024251	9.045184131	9.192007173
395	4833418N02Rik	1.068345853	3.496979935	2.30808573	2695	Ccdc67	1.636463177	2.493999491	1.987802488	4995	Fabp5	9.745731369	9.537891222	9.53644146	7295	Ipo13	7.687918213	7.969920466	7.819359835	9595	Nr2f1	9.841747334	9.643624126	9.716424168	11895	Rpl13a	11.57790112	11.51683748	11.45317119	14195	Tmem159	6.073537233	5.28998131	5.565047684
396	4833420G17Rik	7.496997195	7.220601093	7.447853993	2696	Ccdc69	2.071389575	3.351227939	2.305028297	4996	Fabp7	8.364458749	8.174503985	7.721075219	7296	Ipo4	7.228249666	7.018524513	7.228409241	9596	Nr2f2	9.718371794	8.826921083	7.939245948	11896	Rpl14	11.12764794	11.17558119	10.98137756	14196	Tmem160	9.121657365	9.003202211	9.075488591
397	4833422C13Rik	7.207340198	6.653837014	7.01364477	2697	Ccdc71	7.480385151	7.36979497	7.208602838	4997	Fabp9	2.935129474	6.731986481	4.935338838	7297	Ipo5	8.600378467	8.940143387	8.511248826	9597	Nr2f6	6.859245317	6.723169522	6.439079896	11897	Rpl14-ps1	10.0818325	9.8374296	9.995404111	14197	Tmem161a	6.633262157	6.452635826	6.51932486
398	4833423E24Rik	3.376036352	1.841544641	2.791839024	2698	Ccdc71l	6.999244358	7.097023439	7.078214161	4998	Fadd	4.327409655	4.155077949	4.227691061	7298	Ipo7	7.219599315	6.809818275	7.329027416	9598	Nr3c1	9.214977481	9.150447067	9.457875157	11898	Rpl15	9.611673647	9.51039634	9.404118493	14198	Tmem161b	7.919034843	7.643719959	8.046403868

399	4833427G06Rik	2.370137795	0.876096988	1.571015298	2699	Ccdc73	7.886148032	7.703416338	7.301478446	4999	Fads1	9.644697101	9.537933728	9.817687486	7299	Ipo8	7.579516084	7.209870535	7.609600767	9599	Nr3c2	4.478889798	4.972815191	5.11053789	11899	Rpl17	9.086196282	9.130338007	9.149945807
400	4833439L19Rik	8.365146021	8.118156542	8.251531443	2700	Ccdc74a	6.292601143	6.808558839	6.721895477	5000	Fads2	4.08753671	5.06767397	4.936294814	7300	Ipo9	8.162141271	8.03748591	8.098993633	9600	Nr4a1	6.771842895	7.042626053	7.595029136	11900	Rpl18	8.969116097	9.033261457	8.796895201
401	4921504A21Rik	5.90672746	5.422952235	5.1488648	2701	Ccdc77	5.327521425	5.22894964	5.440058715	5001	Fads3	5.883774208	5.934318515	5.813658235	7301	Ipp	4.720769092	4.218550718	5.111659313	9601	Nr4a2	9.825736754	9.056431578	8.727889131	11901	Rpl18a	11.1738495	11.03940764	10.77598489
402	4921507L20Rik	1.06079051	2.485696307	2.573053419	2702	Ccdc78	5.543969858	6.092144891	6.025002534	5002	Fads6	1.047998182	1.909370656	1.573376138	7302	Ippk	6.69723269	6.801733126	6.799918988	9602	Nr4a3	8.405228214	8.052549371	7.616110523	11902	Rpl19	9.588400932	9.719293831	9.639992634
403	4921507P07Rik	2.403102033	3.458296997	3.153197284	2703	Ccdc79	2.649215017	2.677153307	2.791838911	5003	Faf1	7.410140501	7.425486638	7.27469968	7303	Ipw	1.63277771	0.894701012	0.987296341	9603	Nr5a1	1.048018794	1.488666065	1.573371593	11903	Rpl21	9.97181888	9.713007416	9.589136443
404	4921511H03Rik	1.054632191	1.89840106	1.571920491	2704	Ccdc80	6.325579171	6.490456144	6.002135029	5004	Faf2	8.11337911	7.870161387	8.035324569	7304	Iqca	4.169079099	4.948895508	4.227927913	9604	Nr5a2	2.064545226	2.464107287	1.983567698	11904	Rpl22	11.64610355	11.54213767	11.40570467
405	4921511I17Rik	3.986168247	4.091921352	3.980253903	2705	Ccdc81	3.401332126	4.578778802	4.070729049	5005	Fah	6.361297901	5.967211589	5.734132547	7305	Iqcb1	6.401742274	6.210213531	6.722406576	9605	Nr6a1	5.934195635	5.287838989	5.911705884	11905	Rpl22l1	10.17337423	10.09611068	10.20277535
406	4921524J17Rik	7.240809549	7.288978214	6.990655855	2706	Ccdc82	7.628438264	7.617229739	8.088860862	5006	Fahd1	6.922983218	6.633020263	6.609386548	7306	Iqcc	4.656993451	4.000404648	4.682597343	9606	Nradd	3.397673429	3.159023588	4.306785868	11906	Rpl23	11.15063903	11.02757792	10.89657805
407	4921530L21Rik	2.059559693	3.36728734	1.984858783	2707	Ccdc83	1.639969728	2.488374379	1.571012505	5007	Fahd2a	5.859221578	5.476541362	6.02553396	7307	Iqcd	4.154113625	3.758135277	1.97019749	9607	Nrap	2.385267242	2.457693758	1.982656044	11907	Rpl23a	9.181001398	9.121057236	9.119553464
408	4921531C22Rik	3.673861739	4.086640148	4.303046777	2708	Ccdc84	7.913382484	8.092261305	7.793631231	5008	Faim	7.200625938	7.353463638	7.255935652	7308	Iqce	5.566921874	5.042232856	5.47284768	9608	Nrarp	5.891273712	6.004166718	6.456518181	11908	Rpl24	9.665454053	9.697599319	9.540279958
409	4921533I20Rik	1.64648621	1.878935353	1.569328579	2709	Ccdc85a	7.695921684	7.838075091	7.463307793	5009	Faim2	9.876746979	9.925155587	10.06880454	7309	Iqcf1	0.035117264	2.913718551	1.987400252	9609	Nras	8.56002358	8.569804728	8.523061485	11909	Rpl26	11.72529165	11.62692184	11.40411999
410	4921539E11Rik	0.011136083	1.512826965	0.991148654	2710	Ccdc85b	7.208468204	7.284395653	6.945597074	5010	Faiml	1.052493429	1.901923001	1.988218066	7310	Iqcf3	2.103074939	4.984128321	2.562387801	9610	Nrbf2	6.522321164	6.243737145	6.208258396	11910	Rpl27	8.874598848	8.885718298	8.590886019
411	4930402H24Rik	7.159317804	7.539531616	6.921578025	2711	Ccdc86	5.834600812	5.839512703	6.068363281	5011	Fam101a	1.070305573	2.203316082	0.982130401	7311	Iqcf4	2.087870472	4.907659681	1.559975082	9611	Nrbp1	8.627459155	8.609197714	8.690841152	11911	Rpl27a	11.41313689	11.36969169	11.20697515
412	4930405A21Rik	2.054188717	1.461108768	0.984078295	2712	Ccdc87	3.073799994	3.567912528	3.442668147	5012	Fam101b	6.87468551	7.19195144	6.77425403	7312	Iqcf5	1.11337718	4.811343291	1.559439276	9612	Nrbp2	7.853786741	7.558648612	7.292410623	11912	Rpl28	9.774145616	10.05640258	9.942920787
413	4930407I10Rik	0.040283643	2.712563232	1.570575983	2713	Ccdc88a	11.32493195	11.18805087	11.31816174	5013	Fam102a	8.727706794	8.833382429	8.598342878	7313	Iqcg	5.694473609	6.037507997	6.229707806	9613	Nrcam	10.69615847	10.71213019	10.64531767	11913	Rpl29	8.489656759	8.474901953	8.530839117
414	4930412C18Rik	4.167879142	4.368776275	4.682683919	2714	Ccdc88b	2.408511192	3.68005242	2.299752135	5014	Fam102b	8.798063609	8.866442706	8.777673346	7314	Iqch	0.027270362	2.725847938	1.98864709	9614	Nrd1	9.900509789	9.792134762	9.734863872	11914	Rpl3	10.93535736	10.67361425	10.77519939
415	4930413G21Rik	1.672813481	2.666808124	2.30307754	2715	Ccdc88c	5.023339451	4.900543795	4.736571315	5015	Fam103a1	8.319219092	8.337338064	8.393622563	7315	Iqck	7.836957188	8.239422362	8.139843548	9615	Nrde2	6.603655301	6.465403058	7.025407786	11915	Rpl30	9.141313205	9.279201282	9.093685541
416	4930419G24Rik	3.056803782	0.811704348	2.792246063	2716	Ccdc89	2.644642431	3.059032215	1.982649326	5016	Fam104a	9.64654639	9.706978911	9.572426266	7316	Iqgap1	7.366035963	7.460931018	7.389396536	9616	Nrep	9.219932874	9.054154144	9.28384477	11916	Rpl31	10.32961298	10.39746478	10.21238756
417	4930426L09Rik	1.630816316	0.899696491	1.989085748	2717	Ccdc9	6.975240327	6.869815344	7.199615501	5017	Fam105a	5.9659899	6.641215378	5.861618955	7317	Iqgap2	9.425156763	9.65506386	9.410358576	9617	Nrf1	6.266720248	6.127101295	5.980047337	11917	Rpl31-ps12	8.177632959	8.038082493	7.818998219
418	4930427A07Rik	2.643797423	1.856416938	2.569999732	2718	Ccdc90b	7.112157342	7.01976869	6.787117793	5018	Fam107a	10.36719762	10.48537673	10.1746702	7318	Iqgap3	0.060181868	2.195868516	2.570780771	9618	Nrg1	3.405960078	3.536667801	4.069783404	11918	Rpl32	11.18187952	11.2041811	11.04672556
419	4930429B21Rik	4.338061441	4.928592077	5.711068764	2719	Ccdc91	8.087023858	8.511944748	8.480020271	5019	Fam107b	4.784319751	5.467038844	5.06783635	7319	Iqsec1	9.252915298	9.264279757	9.467955853	9619	Nrg2	4.672013305	5.338956411	4.837147077	11919	Rpl34	11.34677611	11.35104343	11.31480921
420	4930430F08Rik	7.515721586	7.599347923	7.934632113	2720	Ccdc92	8.770876866	8.782749739	9.184754739	5020	Fam109a	5.02628859	4.701810057	4.681110267	7320	Iqsec2	9.164999641	9.182895856	9.149990027	9620	Nrg3	6.6347539	6.783711968	7.047054712	11920	Rpl34-ps1	11.24170227	11.26724961	11.20638979
421	4930432K21Rik	4.07098089	3.647953038	3.438706707	2721	Ccdc93	6.16879009	6.042922683	6.504252818	5021	Fam109b	2.375284083	0.858746746	1.985533985	7321	Iqsec3	8.406707098	8.419651068	8.550038325	9621	Nrg3os	0.040268823	0.871636693	2.795972347	11921	Rpl35	10.35410941	10.10567706	10.00922707
422	4930444P10Rik	1.077132044	3.064321685	0.980349174	2722	Ccdc94	6.167263266	6.232991941	6.595988902	5022	Fam110a	5.804467622	5.538877941	5.888105942	7322	Iqub	5.504705562	5.755253362	4.932402336	9622	Nrg4	1.08521349	0.816121769	1.565354193	11922	Rpl35a	8.623245613	8.477762933	8.583662047
423	4930447A16Rik	2.046570565	1.480233242	1.572382049	2723	Ccdc96	5.021796997	4.810826426	4.0665454	5023	Fam110b	8.12884458	7.774025528	7.714558168	7323	Irak1	8.029418811	8.406016681	8.109239025	9623	Nrgn	14.84350513	14.87212319	14.81656731	11923	Rpl36	9.239802459	9.07904743	8.790420187
424	4930447C04Rik	4.248791264	4.001199084	3.979055804	2724	Ccdc97	7.375786591	7.704758344	7.503603895	5024	Fam111a	5.772558241	5.121255303	5.189464303	7324	Irak1bp1	7.986885001	7.952304908	7.874801906	9624	Nrip1	7.328301439	7.157779335	7.519497931	11924	Rpl36a	8.919784511	9.057660452	8.865639733
425	4930447F24Rik	0.016600372	1.921606679	0.989760481	2725	Cchcr1	2.088018686	3.328766727	3.305818007	5025	Fam114a1	5.362875875	5.367320115	5.265772654	7325	Irak2	6.438321396	6.648781961	6.455711356	9625	Nrip2	6.68140455	6.694279495	7.275581752	11925	Rpl36al	9.442229481	9.329750715	9.182037537
426	4930447M23Rik	4.879487056	4.898869553	3.784099017	2726	Cck	11.98966332	12.00659071	11.66339556	5026	Fam114a2	8.215809781	8.452755517	8.549954413	7326	Irak3	3.865731847	3.453743618	1.976519442	9626	Nrip3	11.55461523	11.41304622	10.93339983	11926	Rpl37	11.46529815	11.4010477	11.21058188
427	4930447N08Rik	2.090831572	3.454669958	3.443412408	2727	Cckar	1.63463033	0.889949879	0.986737586	5027	Fam117a	5.362025849	5.336378848	5.150063545	7327	Irak4	3.086923654	2.990407206	4.569941035	9627	Nrl	2.069274609	2.455475287	2.569528531	11927	Rpl37a	9.644759744	9.758456232	9.636394612
428	4930451G09Rik	1.054679281	0.885151176	2.310422588	2728	Cckbr	6.678420734	6.762018117	6.826300008	5028	Fam117b	8.49345403	8.185623289	8.397926448	7328	Ireb2	8.146833982	8.195436578	8.381058026	9628	Nrm	3.768116045	3.435851708	2.561873748	11928	Rpl37rt	12.76201962	12.56800243	12.46277549
429	4930452B06Rik	4.546571524	4.524792327	5.408182805	2729	Ccl11	0.011140597	0.927248116	1.991605264	5029	Fam118a	6.633592443	6.567435788	6.503608217	7329	Irf1	5.962873035	6.089302354	6.046404596	9629	Nrn1	10.44273593	10.66035961	10.64708831	11929	Rpl38	10.36366172	10.45454036	10.23851387
430	4930453N24Rik	6.080858266	5.898959707	6.189678795	2730	Ccl12	1.653791375	2.694616461	1.567434397	5030	Fam118b	6.572159239	6.647032044	6.388975498	7330	Irf2	8.36114621	8.377453879	8.402133227	9630	Nrn1l	3.770388442	3.29994673	3.983438732	11930	Rpl39	9.725828168	9.534873875	9.499614804
431	4930455G09Rik	0.016590858	0.915575703	1.990526329	2731	Ccl17	3.072620172	3.020623524	2.787877334	5031	Fam120a	10.03324442	10.17256084	9.948636952	7331	Irf2bp1	7.649729447	7.665838179	7.631175362	9631	Nrp	3.414676452	4.310134184	3.980115374	11931	Rpl39l	0.076772713	3.351122975	2.305010311
432	4930455H04Rik	5.115369417	4.354475339	3.883447468	2732	Ccl19	1.071942968	2.200961173	2.571323698	5032	Fam120aos	6.951755582	6.986555374	7.237715579	7332	Irf2bp2	8.612061139	8.530574559	8.806656283	9632	Nrp1	9.971989075	9.907085407	9.218150486	11932	Rpl4	11.80423288	11.75626311	11.58867623
433	4930461G14Rik	0.005608334	0.940318714	0.992698372	2733	Ccl21b	0.016597084	0.915562877	1.574995394	5033	Fam120b	7.564263646	7.488193489	7.728440924	7333	Irf2bpl	9.267631182	9.102058752	9.260819846	9633	Nrp2	7.538736259	7.668223807	7.431330305	11933	Rpl41	11.78224868	11.68254895	11.48030702
434	4930465K10Rik	1.032340788	0.933566573	0.991897432	2734	Ccl24	0.01659603	0.91556505	2.312886547	5034	Fam120c	9.40450822	9.203760527	9.473931385	7334	Irf3	5.886555647	6.033687863	5.594042408	9634	Nrros	5.361281432	5.370501333	5.109451886	11934	Rpl5	10.50569584	10.37202092	10.4114784
435	4930469G21Rik	2.078769968	3.043088554	2.567514931	2735	Ccl25	5.432686605	5.16220124	4.678644668	5035	Fam122a	7.046362084	7.275707296	7.140029844	7335	Irf5	2.672063961	2.831493962	3.303566658	9635	Nrsn1	11.31953621	11.1328598	11.44863353	11935	Rpl6	10.89430956	10.80426224	10.79054445
436	4930473A02Rik	4.31680538	4.020176839	3.146849512	2736	Ccl27a	9.299892461	9.432374883	9.513382617	5036	Fam122b	6.538938701	6.78679321	6.283357146	7336	Irf6	3.061858486	2.867840851	2.984255293	9636	Nrsn2	8.735169578	8.605280372	8.662930586	11936	Rpl7	8.773999763	8.689172701	8.707867366
437	4930474G06Rik	2.046570624	0.889880687	1.572382032	2737	Ccl27b	6.478385411	7.599551533	7.346780714	5037	Fam124a	5.754570391	6.184925901	5.8869211	7337	Irf7	3.225923164	3.207029569	2.568606955	9637	Nrtn	4.1869234	4.962166733	5.189741303	11937	Rpl7a	8.62321254	8.5279143	8.549870676
438	4930474M22Rik	0.016588015	0.91558156	1.990526866	2738	Ccl28	2.657379201	0.769895443	2.302178127	5038	Fam124b	0.013889178	0.921243244	1.575584454	7338	Irf8	6.377473174	6.204823783	5.761215008	9638	Nrxn1	12.52892736	12.29459163	12.38432082	11938	Rpl7l1	7.760131026	7.838456508	7.616389472
439	4930478L05Rik	2.088192966	3.02908568	2.982512431	2739	Ccl3	1.639930644	0.876171221	1.571022591	5039	Fam126a	7.547005478	7.588680183	7.301797981	7339	Irf9	6.435016383	6.103370866	6.169844446	9639	Nrxn2	12.68622404	12.30125077	12.41747163	11939	Rpl8	11.02891213	11.06069794	10.92541453
440	4930480K15Rik	4.65735943	3.724532235	4.440494779	2740	Ccl6	3.990431826	4.233575484	4.228339722	5040	Fam126b	10.95712143	10.81482803	11.03798059	7340	Irgm1	4.235098881	3.423864488	3.439666585	9640	Nrxn3	12.25961518	12.37375219	12.52253244	11940	Rpl9	8.963344498	9.078928718	8.850240396
441	4930480K23Rik	4.713347435	4.371584661	4.890214426	2741	Ccl8	1.043247203	0.910052348	0.98910864	5041	Fam129a	3.228706514	2.869319597	0.975384641	7341	Irgm2	3.88072527	3.538811664	4.152808852	9641	Nsa2	9.81668912	9.680260525	9.63102621	11941	Rplp0	9.529767702	9.749855646	9.443455311
442	4930481A15Rik	10.18763955	10.28332474	9.99954894	2742	Ccl9	3.405165628	4.255750965	2.558871905	5042	Fam129b	7.464221818	7.567052039	7.71507758	7342	Irgq	8.614786627	8.475464178	8.471965416	9642	Nsd1	10.42034299	10.391528	10.41254134	11942	Rplp1	13.53552434	13.42155025	13.2197168
443	4930487H11Rik	2.382218952	1.434297665	2.570508609	2743	Ccm2	7.635225132	7.816902986	7.862919558	5043	Fam129c	2.077815753	2.16694097	1.980116914	7343	Irs1	6.234210002	6.842139681	6.946542387	9643	Nsdhl	6.848854847	7.030328706	6.87483079	11943	Rplp2	10.70254106	10.67000794	10.5724303
444	4930500L23Rik	1.058761757	1.468606226	1.571021032	2744	Ccm2l	2.877940895	2.149690345	2.7893493	5044	Fam131a	10.52141253	10.56671834	10.28142427	7344	Irs2	7.166903441	7.134069966	6.991145662	9644	Nsf	12.17992705	12.07014942	12.07932044	11944	Rplp2-ps1	11.70992817	11.72084574	11.60110659
445	4930503B20Rik	2.07134897	4.500132137	0.977821418	2745	Ccna1	1.659257022	3.602202126	1.982650481	5045	Fam131b	7.687659724	7.879169113	7.800373256	7345	Irs3	1.043241352	0.910065004	2.312432359	9645	Nsfl1c	9.304360292	9.120494995	9.196720092	11945	Rpn1	9.991668566	9.898795301	9.89589819
446	4930503E24Rik	4.716701775	4.227841316	4.227634272	2746	Ccna2	8.166250477	7.849437888	7.741444102	5046	Fam131c	2.65083604	1.405405198	1.980683789	7346	Irs4	3.864895553	2.417546694	3.569547685	9646	Nsg1	10.05507373	10.06890447	10.12973237	11946	Rpn2	8.485553622	8.610429484	8.389324653
447	4930503L19Rik	4.777806022	5.177858575	4.503103034	2747	Ccnb1	2.387392636	2.453365908	2.569283206	5047	Fam132a	5.723725028	5.953316667	5.680444147	7347	Irx1	2.156878596	4.220232487	5.507393349	9647	Nsg2	11.72567504	11.53262274	11.34190421	11947	Rpp14	8.194112008	8.061069034	8.242175894
448	4930505A04Rik	3.366157787	1.450627996	1.568926533	2748	Ccnb2	2.644726429	2.186263543	1.982622768	5048	Fam132b	3.240146619	3.177068766	1.97564937	7348	Irx2	2.127558053	2.064648404	4.627606557	9648	Nsl1	2.37993914	1.440856729	2.571048457	11948	Rpp21	8.166581091	7.952342382	7.761202193

14199	Tmem163	6.901947411	6.799658851	7.284201538
14200	Tmem164	2.865810547	2.181923284	1.56532759
14201	Tmem165	5.889588594	5.878760162	6.150291006
14202	Tmem167	8.812106311	8.890402065	8.982862953
14203	Tmem167b	7.842279906	7.931132194	7.898879034
14204	Tmem168	5.537358636	5.485191416	5.149077452
14205	Tmem169	5.810540462	6.008715211	6.284676878
14206	Tmem17	5.630962891	5.54385727	5.472305006
14207	Tmem170b	7.572053792	7.593634999	7.572529393
14208	Tmem173	5.389161636	4.893911549	3.559419429
14209	Tmem175	8.145511195	8.00360293	8.024515643
14210	Tmem176a	6.680131318	6.758706583	6.282845877
14211	Tmem176b	9.411032732	9.363989424	9.080571017
14212	Tmem177	5.396937537	5.403107608	5.150344182
14213	Tmem178	9.330463177	9.375973212	9.301993004
14214	Tmem178b	4.779081278	4.282411664	5.151829789
14215	Tmem179	9.272171718	9.057274354	9.182220673
14216	Tmem179b	6.66524086	6.852824345	6.721648597
14217	Tmem18	6.555741465	6.421187403	6.58008936
14218	Tmem180	5.213330936	5.871642561	5.681285879
14219	Tmem181a	7.480737725	7.547298066	7.301789999
14220	Tmem181b-ps	5.471261133	5.599213642	5.472230243
14221	Tmem181c-ps	6.188486762	6.382184705	6.169900122
14222	Tmem183a	8.705694002	8.690557971	8.426971707
14223	Tmem184a	1.636450507	3.088877514	0.986185972
14224	Tmem184b	6.974713614	7.10788056	7.140337854
14225	Tmem184c	9.483553564	9.259064649	9.56122662
14226	Tmem185b	6.268371125	6.123839037	6.42382314
14227	Tmem186	5.502285494	5.195089573	5.149699002
14228	Tmem189	6.007589444	5.79382837	5.78755356
14229	Tmem19	7.135857691	7.178250835	7.293146168
14230	Tmem191c	8.26973189	8.099744893	8.274768222
14231	Tmem192	5.606625657	6.03368259	6.151020342
14232	Tmem194	3.250812686	3.949961154	3.889761898
14233	Tmem194b	3.261816322	4.017384714	4.230084661
14234	Tmem196	6.837039828	7.105823512	7.674579014
14235	Tmem198	8.135282851	8.138106223	8.114523334
14236	Tmem198b	4.723648052	4.843143535	5.025790534
14237	Tmem199	7.738271324	7.763845006	7.951927511
14238	Tmem2	6.620576941	6.769596128	6.825731091
14239	Tmem200a	6.635621148	6.874498368	6.800422735
14240	Tmem200c	4.479157004	5.17101076	4.787239502
14241	Tmem201	7.009345909	6.990949371	6.518524037
14242	Tmem202	2.887929391	3.012101185	3.151192275
14243	Tmem203	5.242817397	5.096105868	5.025431749
14244	Tmem204	4.888668561	4.27212591	5.597114686
14245	Tmem205	6.877480633	7.604723143	7.024070699
14246	Tmem206	7.874970506	7.871225883	7.874804038
14247	Tmem208	7.346025825	7.265488842	7.256273303
14248	Tmem209	5.888943073	5.967044743	5.862292067

449	4930506C21Rik	4.321796734	3.734902666	4.229127706	2749	Ccnc	6.555045113	6.135210201	6.389258955	5049	Fam133b	8.828091829	8.615864756	8.602020148	7349	Irx3	3.572656056	4.127475825	5.339030733	9649	Nsmaf	6.36696861	6.077489116	6.246199599	11949	Rpp25	5.252379284	5.392954471	5.504022038	14249	Tmem212	4.40281429	5.483969437	4.226970094
450	4930507D05Rik	5.12259976	5.404972604	4.436874955	2750	Ccnd1	9.102252651	9.055604464	8.803313567	5050	Fam134a	8.749818268	8.95459489	8.922532329	7350	Irx5	3.420805357	4.157704173	5.269514627	9650	Nsmce1	6.519713088	6.266110586	5.403631595	11950	Rpp25l	6.73772426	6.619013683	6.609297284	14250	Tmem214	6.897617482	6.646724423	6.455450845
451	4930511M06Rik	2.636572469	0.854625753	2.308074615	2751	Ccnd2	10.09974834	9.995650057	9.493575507	5051	Fam134b	8.245477628	8.427902995	8.17466651	7351	Irx6	1.659288158	1.425324492	3.446999914	9651	Nsmce2	6.820904854	6.805450169	6.862988783	11951	Rpp30	6.489303771	6.726059101	6.406193611	14251	Tmem215	5.46858814	5.266418712	5.066959119
452	4930512B01Rik	2.867537958	1.843887159	2.792042839	2752	Ccnd3	7.069543406	7.274812219	7.045956567	5052	Fam134c	7.11236795	6.502971338	6.837802715	7352	Isca1	8.36612599	8.376762343	8.443541813	9652	Nsmce4a	7.375764578	7.503646633	7.363528629	11952	Rpp38	6.724083739	6.657430806	6.707948639	14252	Tmem216	5.667444621	5.906379251	5.887133284
453	4930513N10Rik	3.387452365	1.361894795	2.981900787	2753	Ccndbp1	7.572302901	7.697416428	7.579935787	5053	Fam135a	7.925603935	7.797403506	8.06772509	7353	Isca2	7.774193789	7.8495429	8.030118533	9653	Nsmf	10.78853308	10.6945612	10.23375797	11953	Rpp40	4.774207047	5.184555555	4.372209873	14253	Tmem218	6.290954912	6.31221878	6.227508502
454	4930519F09Rik	3.073397518	3.449290411	2.981268441	2754	Ccne1	6.20849804	5.685511228	6.228291637	5054	Fam135b	6.129149832	6.524736228	6.969719226	7354	Iscu	7.78827359	7.849002667	7.898904303	9654	Nsun2	8.871370606	8.756704045	8.728157851	11954	Rpph1	3.406722274	4.180542998	3.564739634	14254	Tmem219	6.122191979	5.925903582	5.957443753
455	4930520O04Rik	1.653933191	0.838321036	2.570765508	2755	Ccne2	7.664131267	7.383993238	7.487851327	5055	Fam136a	6.456141104	6.524629268	6.775025118	7355	Isg15	4.536318138	4.225688118	4.06608252	9655	Nsun3	1.661899921	1.419383862	2.792486251	11955	Rprd1a	9.352105236	9.491057559	9.39572949	14255	Tmem220	4.076479719	4.313112543	3.786905275
456	4930523C07Rik	3.09394827	4.101651381	3.680482069	2756	Ccnf	2.887158843	4.592469703	1.974649198	5056	Fam13a	6.647717077	6.46887578	6.208325787	7356	Isg20	3.775804618	3.753575236	3.681224422	9656	Nsun4	6.145756165	6.233736495	5.679153364	11956	Rprd1b	9.023169987	8.83387143	9.177272877	14256	Tmem222	7.821876659	7.894107118	7.616366676
457	4930525G20Rik	3.387475845	3.024067163	2.788330672	2757	Ccng1	8.463703904	8.427331193	8.455846371	5057	Fam13b	8.611613915	8.31211895	8.613060484	7357	Isg20l2	5.129302669	5.729408649	5.681413037	9657	Nsun5	6.875950423	7.207344098	7.109498125	11957	Rprd2	8.113796444	8.137588257	8.199291066	14257	Tmem223	7.384361353	7.641778404	7.189099392
458	4930526I15Rik	6.821328551	6.756893353	7.002898187	2758	Ccng2	7.298904149	7.195505029	7.129628041	5058	Fam13c	8.966182989	9.028104909	8.834782046	7358	Isl1	3.251055535	3.428371355	4.232085259	9658	Nsun6	5.367830139	5.291342611	5.958792048	11958	Rprm	8.27359094	8.486562314	8.315835055	14258	Tmem229a	9.248083448	9.070476163	9.326515857
459	4930528A17Rik	2.041410897	0.904770265	1.98954238	2759	Ccnh	8.375084044	8.445850907	8.359372034	5059	Fam149a	5.812234365	6.250460748	6.456676324	7359	Islr	5.509093501	5.476399559	5.81336458	9659	Nsun7	3.67973718	4.153194849	4.301823349	11959	Rprml	9.54747451	9.423603571	9.026992137	14259	Tmem229b	6.697020145	6.968351297	7.046389225
460	4930532I03Rik	1.675049405	3.038715602	0.97384828	2760	Ccni	10.39579802	10.48800253	10.59380235	5060	Fam149b	6.504413111	6.211362746	6.519764073	7360	Islr2	8.80347855	8.679238268	7.951104073	9660	Nt5c	8.188423783	8.027504354	7.892695392	11960	Rps10	9.380127792	9.640610042	9.640014777	14260	Tmem230	7.36415129	7.372714365	6.933821633
461	4930538K18Rik	2.407549984	3.451853535	2.788050174	2761	Ccnj	4.92952256	3.610671348	4.37214813	5061	Fam150b	4.816741017	4.24969031	3.146728999	7361	Ism1	3.673286982	3.116691724	4.44126131	9661	Nt5c1b	2.719148067	6.641927899	2.774963067	11961	Rps11	9.217622557	9.321955233	9.26308166	14261	Tmem231	5.986527216	5.764747897	5.622864042
462	4930539E08Rik	5.228437424	3.637181478	4.842163987	2762	Ccnjl	5.722267745	5.508502982	5.065458335	5062	Fam151a	1.064760554	1.881889529	1.985876438	7362	Isoc1	7.895022048	7.729753922	8.346643008	9662	Nt5c2	7.490449977	7.293874522	7.899302706	11962	Rps12	8.631246755	8.745531741	8.479718743	14262	Tmem232	2.676575717	3.664209893	3.682934256
463	4930539J05Rik	3.764852581	2.83481973	2.786756094	2763	Ccnk	6.50860157	6.695938111	6.734978583	5063	Fam151b	5.211061657	5.805925728	5.227678334	7363	Isoc2a	7.337449916	7.255518018	7.30197286	9663	Nt5c3	8.855289125	8.876976662	8.907813869	11963	Rps13	8.767002833	8.904798835	8.70104852	14263	Tmem234	8.015834638	7.738508563	7.701009111
464	4930539N22Rik	3.051857916	2.193281094	2.306587625	2764	Ccnl1	7.696119129	7.860286202	7.945887376	5064	Fam155a	9.715156318	9.585994664	9.695990906	7364	Isoc2b	3.774018389	4.186749621	3.889074798	9664	Nt5c3b	6.034613026	6.162919691	6.284508773	11964	Rps14	12.49764922	12.43067206	12.46756562	14264	Tmem237	9.046192081	9.290456031	8.901768692
465	4930544D05Rik	0.029912895	2.723021148	0.986735171	2765	Ccnl2	7.765449758	7.459409907	7.397678939	5065	Fam159b	2.383265199	2.190983162	2.793381662	7365	Ispd	4.843288642	5.54535314	5.406668599	9665	Nt5dc1	3.781728649	4.314993399	3.887208002	11965	Rps15	11.15197206	11.17501901	10.92393866	14265	Tmem238	4.772086399	4.298283739	4.372792082
466	4930544G11Rik	0.024604631	3.096348559	0.98789503	2766	Ccno	3.227335433	2.872535499	2.568162821	5066	Fam160a1	3.390134927	3.175772592	3.151860656	7366	Israa	2.382122637	1.86201705	2.570531417	9666	Nt5dc2	6.620246693	7.00318603	6.354227812	11966	Rps15a	11.0207766	10.98580518	10.70104668	14266	Tmem239	1.077088934	3.360552953	1.567079953
467	4930545L23Rik	3.233328989	1.381058037	3.893340751	2767	Ccnt1	5.362303869	5.160680259	4.979366878	5067	Fam160a2	8.260304253	8.314289582	8.246797762	7367	Ist1	9.384392213	9.39428139	9.317560815	9667	Nt5dc3	9.143440115	9.067243791	8.619994851	11967	Rps15a-ps4	5.288860763	5.365749103	5.302239129	14267	Tmem240	5.753611617	6.308896915	5.957761691
468	4930547E14Rik	1.073840684	2.198239939	2.307164684	2768	Ccnt2	6.820273656	6.564468918	6.875343614	5068	Fam160b1	7.115560023	7.454106308	7.5951147	7368	Isy1	6.648890398	6.259219208	6.838758868	9668	Nt5e	4.604545853	4.698107888	5.026010784	11968	Rps15a-ps6	8.259255525	7.892153803	7.7806208	14268	Tmem241	3.894054434	4.158465447	4.737234782
469	4930549G23Rik	1.647996737	2.206054552	1.568937506	2769	Ccny	8.947451645	8.957546219	8.831746743	5069	Fam160b2	7.230594773	7.255478434	7.189295312	7369	Isyna1	7.657465208	7.69092355	7.645429953	9669	Nt5m	7.461791304	7.046211919	7.274889127	11969	Rps16	10.26278457	10.08803366	10.0078528	14269	Tmem242	9.284860736	9.297821481	9.109233013
470	4930550C14Rik	2.665139521	3.969384793	3.152300834	2770	Ccnyl1	5.406979681	5.745632361	5.86312771	5070	Fam161a	4.835008693	5.243442721	4.679841574	7370	Itch	7.239835345	7.122688373	7.28398678	9670	Ntan1	8.702723078	8.714210003	8.940133494	11970	Rps17	9.892784327	9.985203387	9.876300513	14270	Tmem243	6.190400412	6.000871538	6.695198211
471	4930556M19Rik	3.377416004	2.170973996	2.568141845	2771	Ccp110	7.926789042	8.090583239	7.957123447	5071	Fam161b	6.007017413	5.841961014	6.003377888	7371	Itfg1	9.90694795	9.898803456	9.776013922	9671	Ntf3	4.604191231	5.284811525	4.438620927	11971	Rps18	9.531620258	9.448833609	9.395698041	14271	Tmem245	5.503812243	5.227782007	5.595737373
472	4930562F07Rik	0.101440376	2.86166239	2.790084625	2772	Ccpg1	8.842199292	8.967617129	8.974408186	5072	Fam162a	8.657553287	8.51366781	8.439336027	7372	Itfg2	4.734933321	5.486265733	6.090978739	9672	Nthl1	3.388591166	2.64394915	2.981573249	11972	Rps19	8.906126009	8.562110595	8.479730627	14272	Tmem246	9.034285533	8.843332801	8.977240566
473	4930563E22Rik	4.538308993	5.357174709	4.888726517	2773	Ccpg1os	5.083159651	5.428197975	5.108738706	5073	Fam163a	6.745611961	5.761348233	6.067496109	7373	Itfg3	5.691834476	5.937796231	5.735846515	9673	Ntm	11.15601701	10.91303705	11.23089297	11973	Rps19bp1	7.464265798	7.429858485	7.255765915	14273	Tmem248	7.963370752	8.119679956	8.088614211
474	4930570G19Rik	4.938971216	4.736833599	5.228948136	2774	Ccr2	0.081506476	3.050682732	2.985177314	5074	Fam163b	9.717343912	10.07337134	9.795361089	7374	Itga1	3.671090274	3.924795472	4.229300085	9674	Ntmt1	5.69885875	6.088634352	6.170414846	11974	Rps19-ps3	7.346045833	7.340029724	6.990876168	14274	Tmem25	6.860751461	6.630418221	6.887213178
475	4930577N17Rik	1.659370639	2.457474759	2.305774127	2775	Ccr5	4.329525819	4.485178173	4.065853213	5075	Fam166a	9.247723177	9.247697113	9.054074543	7375	Itga10	2.382169263	2.890106139	0.980327635	9675	Ntn1	2.893777801	3.302685617	2.785232809	11975	Rps2	9.063396594	9.209494849	8.641510723	14275	Tmem251	5.43856432	5.540212981	5.43930643
476	4930578M01Rik	3.757341243	3.189059513	2.789355894	2776	Ccr9	2.652638561	0.790390625	3.154959403	5076	Fam166b	8.559642526	8.430697782	8.542380572	7376	Itga2b	4.08120939	4.716142658	4.790203019	9676	Ntn3	4.668061388	5.243538127	5.339583185	11976	Rps20	11.75603127	11.70089385	11.36904381	14276	Tmem252	3.98872323	4.372828823	3.78595221
477	4930579G18Rik	6.568385922	6.364997971	6.355463488	2777	Ccrl2	2.639747903	2.468508047	1.567791529	5077	Fam167a	3.236174846	3.327594868	1.977218546	7377	Itga3	5.326754384	5.120149622	5.709634991	9677	Ntn4	0.008383876	0.933569984	1.576874431	11977	Rps21	10.29012072	10.45267677	10.33422101	14277	Tmem253	0.011137435	0.927255185	0.991148295
478	4930579G24Rik	2.876565179	3.034511876	2.789705916	2778	Ccrn4l	7.010131814	6.680012697	6.693837752	5078	Fam167b	3.757368475	2.855717286	1.977878441	7378	Itga4	7.084645829	6.192492524	6.001416334	9678	Ntn5	5.1933646	4.49298829	4.566701391	11978	Rps23	11.18176169	10.93575876	10.81204755	14278	Tmem254a	7.276442901	7.128245452	6.454882848
479	4930579K19Rik	2.88055216	2.850281422	2.982213468	2779	Ccs	6.211801504	6.209103303	6.550254508	5079	Fam168a	11.10845877	11.06781488	11.23089716	7379	Itga6	6.812259485	7.33836096	7.372448101	9679	Ntng1	8.509852237	7.728245056	8.018686191	11979	Rps24	11.66650891	11.72425217	11.63773585	14279	Tmem254b	7.314494776	7.021913164	6.405383473
480	4930581F22Rik	2.893333616	2.823439799	4.15434786	2780	Ccsap	7.375451633	7.848375248	7.863281531	5080	Fam168b	9.510813357	9.400028406	9.513364171	7380	Itga7	5.087836831	5.75037672	5.371694633	9680	Ntpcr	6.807336715	6.683296181	6.8873224	11980	Rps25	9.738771061	9.72195157	9.716378364	14280	Tmem254c	7.216115782	7.012246907	6.371406496
481	4930586N03Rik	3.981117134	3.935579464	3.564439454	2781	Ccser1	5.168679193	5.365960776	5.889175845	5081	Fam169a	7.310441874	7.46717088	7.844714073	7381	Itga8	4.240252806	4.019766243	4.152028453	9681	Ntrk2	11.38834844	11.33858836	11.47784462	11981	Rps26	10.61964166	10.6185137	10.45671129	14281	Tmem255a	7.985161313	7.544816556	7.542175847
482	4930587E11Rik	2.667286161	2.639678508	2.981112084	2782	Ccser2	9.034578351	8.825769484	9.147516376	5082	Fam169b	0.021984227	0.904805479	1.573890434	7382	Itga9	4.887672235	4.466772981	5.266917568	9682	Ntrk3	9.349468812	9.120344947	9.227899574	11982	Rps27	8.871816221	9.131292593	8.895858856	14282	Tmem255b	2.866691071	2.179605437	1.981759085
483	4930590J08Rik	0.005608334	0.940318714	0.992698372	2783	Cct2	9.37732004	9.194919346	9.310905128	5083	Fam171a1	8.773683646	8.578787365	8.784211623	7383	Itgad	2.383272314	1.859151931	2.570259151	9683	Nts	6.187932851	5.606780698	5.786379222	11983	Rps27a	10.56026051	10.5164994	10.31197834	14283	Tmem256	9.599762144	9.303080861	9.324251168
484	4930592A05Rik	3.653302178	3.868122979	3.303320166	2784	Cct3	9.621348226	9.490785492	9.32421319	5084	Fam171a2	9.439683288	9.217448116	8.962856743	7384	Itgam	6.078629948	6.183643122	6.150582567	9684	Ntsr1	4.235834413	4.883515822	2.559572212	11984	Rps27l	7.375048832	7.385095338	7.310826362	14284	Tmem258	8.519016743	8.372706175	8.414600588
485	4930592I03Rik	0.027292581	0.894743339	0.987301324	2785	Cct4	8.675961636	8.53535381	8.232441465	5085	Fam171b	11.12960469	10.96001486	10.91808722	7385	Itgav	6.770294702	6.921722919	7.381253535	9685	Ntsr2	8.832678948	8.927370201	9.062146376	11985	Rps27rt	9.592273061	9.677396403	9.544122779	14285	Tmem259	6.522941328	6.39084517	6.708479747
486	4930593A02Rik	2.646567369	1.848834806	2.792474572	2786	Cct5	9.684038447	9.656599139	9.433179108	5086	Fam172a	8.455280171	8.407945179	8.451734087	7386	Itgb1	8.756091826	8.52014691	8.634628301	9686	Nuak1	9.249619432	9.045492141	8.963008684	11986	Rps28	9.457652995	9.376900108	9.144756842	14286	Tmem260	5.722985637	5.591881191	5.405465374
487	4930594C11Rik	6.144078122	5.835322553	5.980123217	2787	Cct6a	10.02148053	9.913255888	9.885355094	5087	Fam173a	8.356611096	8.500776948	8.199015667	7387	Itgb1bp1	7.886585804	7.702596583	7.53440648	9687	Nuak2	3.059745927	2.443711401	3.307846773	11987	Rps29	12.10121087	12.00766275	12.00303391	14287	Tmem261	7.124236744	7.037551632	6.849975386
488	4930599N23Rik	3.663543848	3.848293159	3.300442669	2788	Cct7	10.79130825	10.81310647	10.62201508	5088	Fam173b	5.567654286	5.546713884	4.885353878	7388	Itgb2	4.153852316	3.149173196	3.681869192	9688	Nub1	9.500184095	9.257458888	9.372319953	11988	Rps3	11.12430361	11.20965224	11.12845478	14288	Tmem263	7.944678867	7.892745142	8.135040124
489	4931403E22Rik	4.831441622	5.140099956	4.788129417	2789	Cct8	10.44125394	10.28797675	10.22313624	5089	Fam174a	8.285609091	8.583882908	8.213418445	7389	Itgb3	3.404860288	3.753658384	4.15281024	9689	Nubp1	5.63298185	5.539803655	5.813987281	11989	Rps3a1	11.15467164	11.0761943	11.00510737	14289	Tmem265	5.661872495	5.423143069	6.13202466
490	4931406B18Rik	0.021974858	1.493098192	0.988490299	2790	Ccz1	7.634951325	7.923365719	7.728357013	5090	Fam174b	7.371275882	6.904427858	7.088944104	7390	Itgb3bp	4.783021157	5.309739612	5.024900481	9690	Nubp2	6.554185253	6.570005415	6.149343508	11990	Rps4l	7.328339431	7.476607739	6.945099008	14290	Tmem29	6.470170089	6.002699085	6.088121139
491	4931406C07Rik	7.068318482	7.038786215	7.302565332	2791	Cd109	3.981514948	4.568342508	3.680409505	5091	Fam175a	4.830329354	4.594528246	4.371506201	7391	Itgb4	5.725405834	5.791835656	6.407998392	9691	Nubpl	3.65192893	3.774709447	2.298162128	11991	Rps4x	9.49213965	9.508420101	9.499608627	14291	Tmem30a	10.98516993	10.93254276	10.96226781
492	4931406H21Rik	0.029938514	0.889882685	1.988212731	2792	Cd14	4.072575161	4.101915383	1.967781305	5092	Fam175b	7.490879356	7.708920366	7.51117388	7392	Itgb5	6.439950006	6.709800903	6.666593902	9692	Nucb1	7.589466445	7.732116292	7.541890573	11992	Rps5	10.99865523	10.95610842	10.87099177	14292	Tmem33	8.210162779	7.97481333	8.2883262
493	4931406P16Rik	6.753522882	7.060957832	6.968996974	2793	Cd151	7.126099446	7.491579722	7.067060997	5093	Fam177a	2.060915234	1.870138171	1.568154193	7393	Itgb8	3.781185966	3.930419743	4.4423011	9693	Nucb2	7.833953653	7.678175311	7.838331877	11993	Rps6	9.801427018	9.665972141	9.590978405	14293	Tmem35	9.649958348	9.384606202	9.281412921
494	4931408D14Rik	2.86240468	1.431113457	2.570247926	2794	Cd160	2.661651457	2.419566498	3.153042373	5094	Fam178a	7.788699333	7.842533306	7.774194436	7394	Itgbl1	5.660469011	5.781157709	5.406596835	9694	Nucks1	12.09491599	12.12616727	11.90886042	11994	Rps6ka1	5.434624669	4.915697642	5.149678299	14294	Tmem37	3.382455343	2.85994972	2.566474293
495	4931409K22Rik	0.021975677	2.506350504	0.988490111	2795	Cd163	3.974564727	2.612904724	3.983157411	5095	Fam178b	2.089645668	3.456197921	2.565215907	7395	Itih2	3.982562511	2.586829708	3.981156044	9695	Nudc	9.215360865	9.583398661	9.457778725	11995	Rps6ka2	6.946048513	6.622832554	6.354822355	14295	Tmem38a	8.24523334	8.432144599	8.056655713
496	4931414P19Rik	3.894707938	4.430289946	4.625631197	2796	Cd163l1	0.01658422	1.502503759	0.989764425	5096	Fam179a	1.655185127	2.890206402	1.983568027	7396	Itih3	6.350025389	5.749195762	6.813344993	9696	Nudcd1	7.794196607	7.598409874	8.035718251	11996	Rps6ka3	7.113599103	6.976018531	7.423565754	14296	Tmem38b	5.662550317	5.325809352	5.371988705
497	4931423N10Rik	1.628796024	0.904806417	2.312001482	2797	Cd164	7.881545272	7.870990755	7.767721361	5097	Fam179b	8.179111062	8.43499754	8.418888277	7397	Itih5	7.589814447	7.820531834	7.945889202	9697	Nudcd2	7.189753884	7.432238891	7.256065502	11997	Rps6ka4	6.399606816	6.317297439	6.109166783	14297	Tmem39a	3.269580804	4.162953884	3.885916353
498	4931428F04Rik	6.861286419	6.905826381	6.874984239	2798	Cd164l2	4.068166888	3.543298868	3.789166323	5098	Fam180a	3.382451009	2.15511093	3.893470963	7398	Itk	1.628807683	0.904777028	1.573887531	9698	Nudcd3	9.607699628	9.418971284	9.441467833	11998	Rps6ka5	6.473729198	6.698396436	6.863324586	14298	Tmem39b	3.414777043	4.309977109	3.886516431

499	4931429I11Rik	1.646469759	2.208714915	0.983101438	2799	Cd1d1	2.645642691	1.422190573	0.978588416	5099	Fam181a	3.988465543	4.006238423	5.071579353	7399	Itm2a	7.531412984	7.481759793	7.463782635	9699	Nudt1	5.631933573	5.80241303	5.066071678	11999	Rps6ka6	4.398301654	4.08185318	4.066894439	14299	Tmem40	2.064548131	3.360511797	1.56707089
500	4931440P22Rik	3.772727992	2.609416923	3.565993554	2800	Cd200	8.218939278	7.802018735	8.013461299	5100	Fam181b	7.250110059	7.140776461	7.550425292	7400	Itm2b	11.66370646	11.85885276	11.60795371	9700	Nudt10	6.768203128	6.936492761	7.099329832	12000	Rps6kb1	9.054713601	8.895502171	8.895823351	14300	Tmem41a	6.586582309	6.376193773	6.45582376
501	4932413F04Rik	0.047907126	2.477938168	2.308380422	2801	Cd22	0.019288004	0.910109368	1.574433438	5101	Fam183b	5.931913835	5.980814432	5.022684561	7401	Itm2c	12.66577208	12.59036122	12.56305857	9701	Nudt11	7.695980536	7.893793162	7.951688168	12001	Rps6kb2	2.654177056	3.041657689	1.562480393	14301	Tmem41b	7.919249606	7.71942544	7.83174755
502	4932416H05Rik	4.332536702	4.648831382	4.681038592	2802	Cd226	1.647967602	2.703212531	1.985197154	5102	Fam184a	7.651119682	7.549630286	7.910897078	7402	Itpa	8.177997797	8.123885222	7.934047751	9702	Nudt12	2.644676225	2.686506926	2.569772424	12002	Rps6kc1	6.821627432	6.533846815	6.991662186	14302	Tmem42	6.58733071	6.797694273	6.74878959
503	4932438A13Rik	9.902439317	9.955424671	10.15621871	2803	Cd247	3.879788827	3.652214135	3.982398804	5103	Fam184b	4.98340176	5.135443577	4.887468027	7403	Itpk1	7.249734162	7.38012763	6.956893996	9703	Nudt13	6.082605168	6.71717929	6.320010512	12003	Rps6kl1	7.113579899	7.179377374	7.265761163	14303	Tmem43	6.898679637	6.507156865	6.81318991
504	4932438H23Rik	4.591973585	3.834499117	3.886741394	2804	Cd248	2.400856926	1.814534317	3.306232528	5104	Fam185a	6.660842015	6.265146057	6.337870027	7404	Itpka	9.083496009	9.237045689	9.164879997	9704	Nudt14	5.91045003	5.794816727	5.787677209	12004	Rps7	9.083032885	9.023050099	8.948647893	14304	Tmem44	6.974438053	6.860113206	6.94593798
505	4932443I19Rik	2.652555955	3.044614185	2.791036513	2805	Cd24a	6.249721816	6.502607119	6.440103119	5105	Fam186b	2.644720747	2.457472446	3.156643548	7405	Itpkb	4.94463847	5.038862302	5.439919277	9705	Nudt15	1.062730513	0.867348196	2.988791993	12005	Rps8	11.55576641	11.45570053	11.42602334	14305	Tmem45a	3.64293936	1.814476208	1.56070112
506	4932702P03Rik	4.536898849	4.424586414	3.295541233	2806	Cd274	3.984262518	3.838051996	3.563546649	5106	Fam187a	2.060917371	2.200789511	1.984507442	7406	Itpkc	3.394457945	3.559212702	3.567654363	9706	Nudt16	7.208677647	7.08617003	7.284180714	12006	Rps9	11.57543023	11.66045322	11.40307646	14306	Tmem47	9.791320403	9.792116895	9.65589889
507	4933400F21Rik	2.412943088	2.123757481	2.786868349	2807	Cd276	4.707541069	3.132669039	4.069065533	5107	Fam188a	8.103288124	8.1412582	8.098834783	7407	Itpr1	10.26196098	10.56674215	10.73325817	9707	Nudt16l1	9.085948696	9.09333466	8.819070225	12007	Rpsa	9.726237059	9.9044226	9.564875688	14307	Tmem5	7.695814995	7.719707348	7.89300231
508	4933402C06Rik	0.008382678	0.933572806	0.991898172	2808	Cd2ap	7.581161919	7.751156407	7.564741569	5108	Fam188b	4.162496781	4.312828408	3.679379759	7408	Itpr2	7.880930668	7.792125049	7.812915292	9708	Nudt17	6.348333099	6.51164809	6.422884583	12008	Rptor	6.805147199	6.153208375	6.735524003	14308	Tmem50a	10.82441783	10.75085489	10.5393329
509	4933402J10Rik	1.071922713	3.720193643	1.984533489	2809	Cd2bp2	7.813774318	7.545031594	7.471706217	5109	Fam189a1	6.325875191	6.123763656	6.20911703	7409	Itpr3	2.415343759	2.395900727	4.154989078	9709	Nudt18	6.709019363	6.592268345	6.680575789	12009	Rpusd1	7.716499469	7.606075674	7.787602095	14309	Tmem50b	8.209098059	8.002348444	8.0188732
510	4933404O12Rik	4.418412205	5.119279229	5.709536068	2810	Cd300a	2.877831213	3.579539872	2.982874123	5110	Fam189a2	4.884641235	4.976129019	4.73503343	7410	Itpripl1	3.880546114	4.629871811	2.559043484	9710	Nudt19	8.378999179	8.150350816	8.051292672	12010	Rpusd2	4.777105853	4.537377145	4.788326466	14310	Tmem51	5.39006314	4.285346046	5.26800854
511	4933406C10Rik	0.032536758	1.898354998	1.98780039	2811	Cd300lg	1.666733992	3.475740447	2.79166409	5111	Fam189b	6.636540372	6.949058351	7.373441094	7411	Itpripl2	5.030225454	5.015868546	5.068692242	9711	Nudt2	6.211174684	6.227984721	6.439706814	12011	Rpusd3	4.088741029	4.98317709	4.148230662	14311	Tmem51os1	1.640018724	0.875939834	1.98699605
512	4933406I18Rik	3.393634085	2.630481227	2.980121371	2812	Cd302	5.295881587	5.746431525	6.210412951	5112	Fam192a	8.046706735	8.031780773	8.139872313	7412	Itsn1	9.546221494	9.538067407	9.598371273	9712	Nudt21	6.343873608	6.10601924	6.228441683	12012	Rpusd4	4.477245175	4.932607964	4.680299389	14312	Tmem52	1.664468783	1.843956018	2.79204883
513	4933407K13Rik	3.535750303	3.657538414	3.566106951	2813	Cd320	6.269990151	6.613551082	5.861495633	5113	Fam193a	9.0630182	8.846129983	8.996879434	7413	Itsn2	9.510800981	9.452719355	9.324239681	9713	Nudt22	5.172947785	5.701718473	5.405970933	12013	Rqcd1	6.348052636	6.596244989	6.471987541	14313	Tmem53	4.465545549	3.114633331	4.06742637
514	4933407L21Rik	3.987321867	3.83153814	3.786323355	2814	Cd33	2.881178125	3.325214653	2.300551334	5114	Fam193b	7.023550668	6.944226675	7.209037117	7414	Ivd	6.542019086	6.960225255	6.549368872	9714	Nudt3	12.09983814	12.06144357	12.04975822	12014	Rrad	1.632756346	0.894755615	1.988644515	14314	Tmem54	12.96037481	13.24843823	12.6640522
515	4933408B17Rik	3.050991054	1.437388127	1.983846675	2815	Cd34	8.510744553	8.556313278	8.718315862	5115	Fam195a	6.002826431	5.571248051	5.264971061	7415	Ivns1abp	8.572965654	8.667107608	8.73820536	9715	Nudt4	9.908811014	9.921133307	10.24802647	12015	Rraga	10.23769651	10.12103371	10.13482241	14315	Tmem55a	7.73172937	7.78063231	7.81295502
516	4933409K07Rik	5.538923544	5.676369822	5.81404391	2816	Cd36	3.85931295	3.041531606	1.562454485	5116	Fam195b	7.855165303	8.034629741	7.85654765	7416	Iws1	8.299373393	8.036009601	8.199137206	9716	Nudt5	5.688864056	5.488581109	4.732638209	12016	Rragb	7.122776113	6.742723372	7.150487621	14316	Tmem55b	8.910323833	9.035611715	8.97153827
517	4933411K16Rik	0.037669322	3.084278425	0.985119671	2817	Cd37	3.392513556	3.170494165	2.298485762	5117	Fam196a	3.996891446	5.106605399	4.3012037	7417	Iyd	5.288289325	5.784999969	4.785643208	9717	Nudt6	4.885157207	4.58764904	4.370610126	12017	Rragc	8.30923288	7.990309518	8.301941275	14317	Tmem56	7.88812437	7.796541783	8.27038945
518	4933412E12Rik	4.77149952	4.54935127	4.149504939	2818	Cd38	5.242483177	4.840855679	4.887463615	5118	Fam196b	2.908072826	2.587262713	4.89159349	7418	Izumo4	5.254258146	5.622636073	5.888174889	9718	Nudt7	3.427566997	4.802886765	4.681313872	12018	Rragd	8.683831586	8.410349599	8.83505517	14318	Tmem57	9.311701711	9.299408872	9.29287735
519	4933412O06Rik	3.39254411	2.633399973	3.568108959	2819	Cd3eap	8.450012362	8.132273127	7.962765272	5119	Fam198a	4.33974608	3.691767599	5.536877455	7419	Jade1	7.895139686	7.869521582	8.311172125	9719	Nudt8	5.207967847	5.401500159	5.338474227	12019	Rras	6.034609955	6.106518611	6.067579859	14319	Tmem59	10.16410177	10.18316652	10.1258909
520	4933413G19Rik	1.644936189	1.881942081	0.983578914	2820	Cd4	2.706080709	4.551029822	4.373168819	5120	Fam198b	3.985064732	4.503762588	3.886964131	7420	Jade2	8.162331067	8.205212086	8.154993981	9720	Nudt9	7.781525219	7.731149808	7.940060885	12020	Rras2	6.38187123	6.177994742	6.423632413	14320	Tmem59l	9.365909266	9.523282285	9.33533339
521	4933417D19Rik	3.784415854	3.631733669	4.938225546	2821	Cd40	1.639941215	1.891628368	1.571019863	5121	Fam199x	8.146254858	7.880581791	8.20896609	7421	Jade3	4.717397572	3.816698459	4.681984253	9721	Nuf2	5.033846646	5.3404638	5.067799803	12021	Rrbp1	9.014197437	9.174084972	8.977153731	14321	Tmem60	6.824109951	7.357522202	7.41522341
522	4933417O13Rik	0.016617067	0.915521721	1.57499114	2822	Cd44	5.793083087	4.347060776	4.838433768	5122	Fam19a1	7.998650698	7.84906989	7.844166805	7422	Jag1	5.471523081	5.392673329	5.814341644	9722	Nufip1	5.917540679	5.939169018	6.045483521	12022	Rreb1	4.931653716	3.98568872	4.681078845	14322	Tmem62	6.129600306	6.389296966	7.00336175
523	4933421O10Rik	4.777952269	4.592052496	4.56475032	2823	Cd46	2.072548812	3.208544529	1.564664991	5123	Fam19a2	7.137079037	7.272008974	7.439638807	7423	Jag2	3.654657542	2.110272048	3.790547763	9723	Nufip2	10.42596653	10.2690057	10.44146849	12023	Rrm1	6.421524009	6.435918861	6.694624827	14323	Tmem63a	8.040774713	8.421719759	8.03521014
524	4933424G05Rik	1.060779446	1.888348721	3.159351242	2824	Cd47	10.07522246	10.41164648	10.45372366	5124	Fam19a4	3.532893868	2.618777505	4.071412122	7424	Jagn1	7.418854519	7.177358173	7.265503474	9724	Numa1	7.801753571	7.937013542	8.062432644	12024	Rrm2	1.668106921	3.047611054	2.79143134	14324	Tmem63b	8.661756928	8.60567469	8.80664580
525	4933424G06Rik	2.376505162	2.205997403	2.571861458	2825	Cd52	4.321717388	4.30967338	3.298115839	5125	Fam19a5	9.451094456	9.413512197	9.246703638	7425	Jak1	11.01714892	10.97293101	10.85801904	9725	Numb	6.252699034	6.437398592	6.788129171	12025	Rrm2b	7.595930255	7.620077469	7.463679933	14325	Tmem63c	5.31530401	4.42024659	4.37176067
526	4933427D14Rik	6.753553578	6.849607689	7.150582693	2826	Cd53	5.166939295	5.004573217	4.836838115	5126	Fam204a	7.642493955	7.502128563	7.659563896	7426	Jak2	6.998803093	6.801577652	7.199685437	9726	Numbl	8.497353267	8.346501887	8.09358018	12026	Rrn3	7.993159533	8.08234619	7.928344433	14326	Tmem64	8.140565015	7.886676639	8.17494591
527	4933427G17Rik	2.393930339	3.342635464	2.303554255	2827	Cd55	4.66195061	4.983907253	4.148340304	5127	Fam205a2	2.685517422	3.288074377	4.685345038	7427	Jak3	2.885499283	3.875852262	3.684516131	9727	Nup107	6.575499595	7.059347314	7.160388454	12027	Rrnad1	8.351844915	8.246753856	8.194190425	14327	Tmem65	8.576814451	8.592072671	8.93726364
528	4933428G20Rik	2.873710014	2.665168827	2.983874293	2828	Cd59a	8.109008533	8.378102352	8.056612986	5128	Fam206a	5.985832633	5.990717119	5.910881789	7428	Jakmip1	8.29505683	8.309282945	8.663269837	9728	Nup133	6.05611459	6.009907967	5.957383506	12028	Rrp1	9.75814458	9.632095083	9.694237194	14328	Tmem67	4.261775017	5.094193771	4.5637509
529	4933430I17Rik	1.05028524	1.905573591	0.987304219	2829	Cd59b	2.066944895	2.688504479	1.98294531	5129	Fam207a	8.113337434	8.081935329	7.916549203	7429	Jakmip2	9.37484796	9.324072921	9.191854087	9729	Nup153	6.754895997	6.967010299	7.24729039	12029	Rrp12	6.287214288	6.216627049	6.302630752	14329	Tmem68	7.403524576	7.776796243	7.74843102
530	4933431E20Rik	8.416092579	8.48550818	8.631190006	2830	Cd6	0.042708797	0.86746124	2.795699182	5130	Fam208a	9.513316981	9.440800694	9.827124107	7430	Jakmip3	6.473212579	6.224330059	6.565092887	9730	Nup155	5.411188543	6.19950072	6.266100013	12030	Rrp15	5.216870138	5.865218497	6.191034482	14330	Tmem69	5.607721219	5.765996448	5.78708392
531	4933433G19Rik	2.661603799	3.457215063	3.569722735	2831	Cd63	10.27899559	10.54662369	9.892757411	5131	Fam208b	7.08012719	6.868760782	7.119715629	7431	Jam2	6.060348021	6.441218909	6.208628423	9731	Nup160	4.322552226	3.921546487	2.973620785	12031	Rrp1b	6.225738958	5.585618682	5.837920946	14331	Tmem70	8.135501625	8.07153607	7.89267621
532	4933434E20Rik	8.564095315	8.633217718	8.515286183	2832	Cd68	5.325208599	5.233628443	4.785909342	5132	Fam20a	5.166835032	4.73227513	4.979507278	7432	Jam3	7.716876702	7.793404105	7.687377525	9732	Nup188	4.882150383	5.103269604	4.839191645	12032	Rrp36	5.960819578	5.024289136	5.787368328	14332	Tmem71	1.643290697	2.21425871	2.30903297
533	4933439C10Rik	3.792935237	4.987993943	4.789249276	2833	Cd7	2.052730918	1.888289326	2.573049426	5133	Fam20b	8.972412236	9.066990113	9.021659764	7433	Jarid2	8.424805451	8.245996636	8.54615956	9733	Nup205	5.332012089	5.69944288	6.047616348	12033	Rrp7a	6.434796679	6.615707302	5.95671792	14333	Tmem72	5.602409578	6.505077159	3.42706939
534	5031414D18Rik	1.658055747	2.188562827	2.306039223	2834	Cd72	8.437783484	8.563828132	8.397879895	5134	Fam20c	6.127188318	6.470218878	6.65324301	7434	Jazf1	5.883699891	5.673376608	5.624016855	9734	Nup210	5.363950959	5.428375477	5.566142534	12034	Rrp8	6.538782403	6.422781712	6.227386994	14334	Tmem74	4.456654528	3.141806928	1.96891254
535	5031425E22Rik	5.718874079	5.261921633	5.653541472	2835	Cd74	3.535039133	3.949057142	3.889649842	5135	Fam21	8.984695138	9.059311065	9.333277273	7435	Jchain	1.085265641	3.708717777	3.308743844	9735	Nup214	6.724819926	6.780540376	6.748123735	12035	Rrp9	6.03154309	6.013386951	5.86288948	14335	Tmem74b	3.548997297	4.00954331	4.37419614
536	5031425F14Rik	2.070289917	2.881107937	1.565347157	2836	Cd81	10.83631788	10.97495894	10.60381077	5136	Fam210a	8.255551536	8.301588062	8.376692979	7436	Jdp2	7.63590739	7.832832605	7.787364493	9736	Nup35	5.572507962	6.018712892	5.405614345	12036	Rrs1	7.365551568	7.211579552	7.389514457	14336	Tmem79	3.398080823	3.552116385	3.98359298
537	5031426D15Rik	7.371070392	6.286646527	6.680049657	2837	Cd82	5.254396699	6.333166952	4.931839828	5137	Fam210b	6.763723793	6.810697108	6.283386533	7437	Jkamp	9.338000313	9.417362986	9.418805686	9737	Nup37	4.787524019	5.159802692	5.302472878	12037	Rs1	2.648341276	2.177433069	1.98147733	14337	Tmem8	5.540374022	5.845015313	5.30091736
538	5031434O11Rik	3.070539011	1.36355736	2.982009072	2838	Cd83	6.211806246	5.936187661	6.519481224	5138	Fam212a	4.251787442	4.808380043	4.14907265	7438	Jmjd1c	8.87853178	8.948755065	9.1773177	9738	Nup43	4.240235226	4.177247522	3.146789518	12038	Rsad1	6.469902633	5.851925381	6.423530104	14338	Tmem80	7.392550315	7.603623882	7.28379776
539	5031439G07Rik	7.695877436	7.871917387	8.002599576	2839	Cd84	4.067485505	4.1886225	2.783927009	5139	Fam212b	8.45939128	8.546816266	8.337554583	7439	Jmjd4	5.578967062	6.234101667	6.35585965	9739	Nup50	7.963295575	7.870202013	8.174960485	12039	Rsad2	4.766615598	3.924672817	3.145282162	14339	Tmem81	1.697888033	3.441394202	3.4417971
540	5033403F01Rik	1.048007918	1.488686483	1.573373991	2840	Cd86	2.883099367	2.411672234	2.981594771	5140	Fam213a	9.773971593	9.789304164	9.629200334	7440	Jmjd6	7.103048836	7.056069131	7.256359531	9740	Nup54	4.722310873	4.4768761	4.564755999	12040	Rsbn1	8.455389495	8.407740673	8.491821122	14340	Tmem86a	6.524451107	6.886865827	6.60931780
541	5033404E19Rik	2.38924022	2.449599884	2.568845495	2841	Cd8b1	2.359593757	0.910071826	0.989110946	5141	Fam213b	8.912674162	8.689741136	8.47568531	7441	Jmjd8	9.781043701	9.946755771	9.861119385	9741	Nup62	8.038798764	7.816247888	7.623724393	12041	Rsbn1l	8.4333648	8.349612666	8.333120028	14341	Tmem86b	2.877935349	2.149705246	1.97788415
542	5033406O09Rik	2.852362412	0.871489735	2.573041289	2842	Cd9	7.627563277	7.95476008	7.49546324	5142	Fam214a	6.937804747	7.099073558	6.968910367	7442	Jmy	8.650718297	8.561227655	8.940127644	9742	Nup62-il4i1	2.647403866	2.680888123	3.795356387	12042	Rsc1a1	4.775362253	4.850873745	4.624578083	14342	Tmem87a	7.962593067	7.797359433	8.00809261
543	5330411J11Rik	1.035280736	0.927235759	0.991145989	2843	Cd93	5.325207289	4.918704873	4.979412698	5143	Fam214b	7.135339233	7.179246221	7.002218488	7443	Josd1	7.665410863	7.645168854	7.741669097	9743	Nup85	6.98553577	6.938710832	6.721350759	12043	Rsf1	10.14256681	9.997416613	10.17476163	14343	Tmem87b	8.555903984	8.452563753	8.47185858
544	5330413P13Rik	2.07668747	3.202678533	2.791244246	2844	Cd99l2	8.929216273	8.825906198	9.117089273	5144	Fam216a	8.871838896	8.935986856	8.931282749	7444	Josd2	8.24506876	8.363050638	8.164872471	9744	Nup88	8.552467261	8.602592783	8.673515153	12044	Rsl1	2.870767418	1.402103522	2.56791628	14344	Tmem88	9.069276079	9.310536094	9.04874341
545	5330417C22Rik	5.533304887	5.085518681	4.145186643	2845	Cda	2.679909334	3.76250112	4.37697003	5145	Fam216b	3.790286853	5.033742154	4.566477175	7445	Jph1	8.361419998	8.584050113	8.119273182	9745	Nup93	6.824873368	7.113098963	7.674292356	12045	Rsl1d1	9.420238071	9.367375321	9.487854675	14345	Tmem88b	9.464265542	9.742682588	9.51333808
546	5330426P16Rik	3.990755479	4.001607944	4.149888124	2846	Cdadc1	8.78111226	8.759339767	8.951621964	5146	Fam217a	2.644676034	1.42527645	0.979027323	7446	Jph2	2.384259377	1.42838608	2.570025379	9746	Nup98	5.128377209	5.98373339	4.784972992	12046	Rsl24d1	7.219654128	7.204795889	7.098814381	14346	Tmem89	2.645632664	1.422222642	2.3055157
547	5330434G04Rik	5.9636544	5.987256562	6.109593898	2847	Cdan1	4.981732342	4.475338299	4.981369227	5147	Fam217b	7.867563555	7.61159738	7.92278617	7447	Jph3	9.461962095	9.562979635	9.487826365	9747	Nupl1	7.16619225	6.678932785	6.910664164	12047	Rslcan18	1.056804735	0.880475432	1.57144669	14347	Tmem8b	8.411017393	8.553937109	8.44773547
548	5430402O13Rik	2.877930056	3.689355806	3.444116678	2848	Cdc123	8.805090057	8.650686089	8.828621356	5148	Fam219a	5.201730663	4.888240311	4.935144946	7448	Jph4	11.54551442	11.3569732	11.2711434	9748	Nupl2	4.542928811	4.888374156	4.623492599	12048	Rsph1	5.365440437	6.085701909	4.885007404	14348	Tmem8c	2.037608356	0.915531863	0.98975760

549	5430405H02Rik	5.250350513	5.5468809	5.372791196	2849	Cdc14a	4.164323344	4.558395396	4.505899286	5149	Fam219b	6.724434969	6.669698175	6.707862875	7449	Jpx	4.659534331	4.226093434	4.066134652	9749	Nupr1	5.1668881	5.123975048	4.933493264	12049	Rsph14	0.032501355	2.49402393	1.571920675	14349	Tmem9	8.437700487	8.449708055	8.283718316
550	5430416N02Rik	5.368147241	5.675099775	5.503515944	2850	Cdc14b	6.551841901	6.141548761	6.228151908	5150	Fam220a	6.43879798	6.326068959	6.503543972	7450	Jrk	4.412525169	5.129386699	4.886682466	9750	Nupr1l	3.068535322	2.421045248	2.30114473	12050	Rsph3a	6.439290172	6.579782871	6.564975553	14350	Tmem91	5.780745291	5.064641313	6.003107158
551	5430416O09Rik	2.644672997	1.425286129	1.982639662	2851	Cdc16	7.488513549	7.297512251	7.381037798	5151	Fam221a	2.865006294	2.455430317	2.5695233	7451	Jrkl	6.986762445	6.870679613	6.9801375	9751	Nus1	9.14113633	9.004827181	9.016111342	12051	Rsph3b	6.150145827	6.539787896	6.595024763	14351	Tmem97	6.250256911	6.364190955	6.320078805
552	5430427O19Rik	1.060790009	0.871662345	0.984598156	2852	Cdc20	3.081517104	4.694707036	3.150004161	5152	Fam221b	2.046584078	0.889841388	1.572378099	7452	Jsrp1	4.166027048	4.00518113	4.228676681	9752	Nusap1	4.470795329	4.899955537	4.148864602	12052	Rsph4a	5.564099268	5.636858531	5.22876135	14352	Tmem98	6.121589752	6.129753523	5.43795729
553	5530601H04Rik	4.728275765	4.786634215	5.190415631	2853	Cdc23	6.665424829	6.68382011	6.800490936	5153	Fam222a	4.789392671	5.876399046	4.836148127	7453	Jtb	8.040109439	7.956854953	7.95153323	9753	Nutf2-ps1	7.101719222	7.058615506	6.707377611	12053	Rsph9	6.996828609	7.281509722	6.354161813	14353	Tmem9b	9.527422954	9.430337908	9.324234117
554	5730408K05Rik	6.307846752	6.458463361	6.149840192	2854	Cdc25a	5.885930855	5.972918632	5.502721615	5154	Fam222b	8.097457823	8.23541239	8.199242041	7454	Jun	9.191187615	8.956587273	8.840970261	9754	Nutm1	0.008386612	0.933563535	0.991897072	12054	Rspo1	5.629026778	4.87088221	5.736700273	14354	Tmf1	9.752954515	9.755269181	9.784136187
555	5730409E04Rik	10.42156272	10.42742147	10.45470414	2855	Cdc25b	5.630837174	5.190905509	5.504158065	5155	Fam227a	4.781961558	4.69065022	5.441696184	7455	Junb	8.244588656	7.667033494	8.372643783	9755	Nvl	6.912998921	6.459934334	6.886929809	12055	Rspo2	7.696933933	7.217458736	6.282256473	14355	Tmie	5.938984101	5.438031977	6.209489851
556	5730422E09Rik	1.060826797	2.217083731	0.984588648	2856	Cdc25c	1.048027667	1.488649406	2.575085427	5156	Fam227b	5.885226026	6.393172592	5.438532472	7456	Jund	9.86842497	10.04785071	9.972970679	9756	Nwd1	7.156773809	6.856716232	7.302318047	12056	Rspo3	7.493371626	7.010508305	7.130318469	14356	Tmod1	7.3481283	7.607694809	7.47184883
557	5730435O14Rik	0.011141278	1.512816629	0.991147276	2857	Cdc26	6.343196275	6.088068276	5.786696275	5157	Fam228a	4.169164802	3.38470645	3.978723573	7457	Jup	7.100808157	6.849078833	6.837945986	9757	Nwd2	8.800908719	8.881350044	9.062501169	12057	Rspry1	7.473893788	7.479857243	7.673521404	14357	Tmod2	12.19524294	11.96348197	11.95079784
558	5730455P16Rik	9.535879195	9.360763521	9.603899512	2858	Cdc27	8.32406375	8.415309266	8.587474304	5158	Fam228b	6.587849593	6.151704565	6.47167345	7458	Kalrn	10.75826892	10.87007246	10.84651205	9758	Nxf1	7.52272266	7.60847647	7.397828422	12058	Rsrc1	9.100498507	9.225757876	9.378799402	14358	Tmod3	7.013089782	7.354973253	7.302009789
559	5730480H06Rik	3.535374993	3.427068341	4.231960387	2859	Cdc34	7.702468184	7.867714047	7.92282477	5159	Fam229a	0.120050357	3.972375026	3.685375646	7459	Kank1	7.022344394	6.95711859	6.837763026	9759	Nxf2	0.01660042	1.502474425	0.98976047	12059	Rsrc2	10.73593766	10.48242532	10.58175837	14359	Tmpo	7.436897037	7.139905271	7.129493869
560	5730507C01Rik	1.08381603	1.422242466	2.985987581	2860	Cdc37	9.644471013	9.541823486	9.578065651	5160	Fam229b	6.810842352	8.256424149	6.773750755	7460	Kank2	6.471253058	6.137608459	6.189074891	9760	Nxn	6.348771274	6.76183524	6.246146608	12060	Rsrp1	8.35313188	8.413619008	8.803456888	14360	Tmppe	6.122094511	6.030955423	5.886858407
561	5730508B09Rik	4.541466005	4.476311247	5.304566584	2861	Cdc37l1	10.5573294	10.53471631	10.48775791	5161	Fam26e	5.286287325	5.905041679	5.266213912	7461	Kank3	4.981689904	4.934763407	4.300258215	9761	Nxnl1	2.372807886	0.867164245	0.984074894	12061	Rsu1	7.157602929	7.395200685	7.139768574	14361	Tmprss4	0.013865645	0.921293565	0.990440819
562	5730522E02Rik	4.393052268	4.442377122	3.886879978	2862	Cdc40	8.619723153	8.651805103	8.568656948	5162	Fam26f	2.060870071	1.870238727	2.57131545	7462	Kank4	4.174086759	4.703539128	4.065382944	9762	Nxpe2	1.066609579	0.858676791	2.308378902	12062	Rtca	7.695446235	7.738826537	7.951821019	14362	Tmprss5	1.094509054	3.04610714	2.567944126
563	5730559C18Rik	2.647462227	1.846375388	3.156060519	2863	Cdc42	11.40811015	11.39384973	11.29794868	5163	Fam32a	10.18737395	9.817731961	9.895862496	7463	Kansl1	8.103212664	8.01645902	8.381081919	9763	Nxpe3	6.952608167	7.015551992	7.199217722	12063	Rtcb	9.199160193	9.029967985	8.90469663	14363	Tmprss6	1.679055445	2.657770739	2.301833472
564	5830415F09Rik	0.067388417	1.428306436	2.986415794	2864	Cdc42bpa	10.35098227	10.33141301	10.55554557	5164	Fam35a	5.989265235	6.045159698	6.149948153	7464	Kansl1l	6.739403922	7.031228665	6.707680807	9764	Nxpe4	4.880345052	3.989408636	4.503813434	12064	Rtel1	2.673936739	3.867216777	1.973198961	14364	Tmprss7	3.524831304	3.568784964	2.787687625
565	5830416P10Rik	3.223794657	2.181703506	2.305243206	2865	Cdc42bpb	8.033537782	7.643563104	8.155344209	5165	Fam3a	5.773938259	5.07949036	5.472554416	7465	Kansl2	6.691791046	6.424281349	6.423118259	9765	Nxph1	5.508325527	5.385950343	6.003605217	12065	Rtf1	9.953716572	9.765594199	9.64882089	14365	Tmprss9	2.851228403	1.468417199	0.985095784
566	5830417I10Rik	6.988112524	7.300525413	7.098981495	2866	Cdc42bpg	4.717502624	4.855214543	3.88455947	5166	Fam3c	9.16540922	9.075752265	9.283822958	7466	Kansl3	8.255217904	8.00111351	8.256175079	9766	Nxph2	4.306466595	3.308173655	2.979744878	12066	Rtfdc1	8.797942748	8.628533214	8.72146536	14366	Tmsb10	9.592152333	9.504933079	9.555546561
567	5830418P13Rik	2.641790931	1.434418813	1.567050648	2867	Cdc42ep1	5.439903468	5.673741275	5.813721496	5167	Fam43a	5.837994928	6.106242931	6.864717156	7467	Kantr	8.355615592	8.141894245	8.072702806	9767	Nxph3	6.617376802	6.846244951	6.246385157	12067	Rtkn	7.472826166	7.546157449	7.526975506	14367	Tmsb15b1	5.36757872	5.937188689	5.76232678
568	5830432E09Rik	2.071452601	2.680884716	1.981774329	2868	Cdc42ep2	6.677756464	6.344284759	6.406242431	5168	Fam43b	6.946857582	6.699289308	6.207998166	7468	Kars	8.285864393	8.474442302	8.475839798	9768	Nxph4	3.083967765	4.192800213	4.071171037	12068	Rtkn2	3.666520436	2.793006594	3.437914236	14368	Tmsb15b2	6.035904739	6.284118255	6.302393024
569	5830444B04Rik	2.059619176	1.447291311	1.568539346	2869	Cdc42ep3	5.992716707	5.997976012	6.935313551	5169	Fam45a	6.93850225	6.914322462	7.170268783	7469	Kat2a	5.297660179	5.692884846	5.911063396	9769	Nxt1	5.25195119	5.15382517	5.56573801	12069	Rtl1	3.073203023	3.449646188	3.684760579	14369	Tmsb15l	6.768690675	6.9139912	7.160292613
570	5830454E08Rik	3.381781281	2.15723074	2.790078639	2870	Cdc42ep4	8.639107266	8.641743655	8.645295754	5170	Fam46a	6.754257459	6.666783291	6.734521211	7470	Kat2b	7.232591636	7.722090693	7.623910953	9770	Nxt2	8.224850215	8.051383376	8.213681865	12070	Rtn1	12.82350613	12.66251587	12.630801	14370	Tmsb4x	14.00325946	13.79956439	13.6113744
571	5930403L14Rik	6.996327833	7.013844803	6.487383952	2871	Cdc42ep5	2.870661606	2.169114112	2.303786409	5171	Fam46b	1.630799824	0.899738346	0.987889931	7471	Kat5	7.058079016	7.308530879	7.237647518	9771	Nyap1	4.481228165	5.051406875	5.150829453	12071	Rtn2	7.887670106	7.882631391	8.150279591	14371	Tmtc1	7.192791386	7.604130593	8.242632631
572	5930412G12Rik	4.24937085	3.913888172	4.440540361	2872	Cdc42se1	7.969644686	8.240405228	7.89868117	5172	Fam46c	4.085925247	5.392730465	4.840042463	7472	Kat6a	8.835910399	9.015314332	9.155005554	9772	Nyap2	4.59455494	4.005223418	3.785887534	12072	Rtn3	11.80723547	11.7655196	11.81128547	14372	Tmtc2	5.368127001	4.991382735	5.371788635
573	5930430L01Rik	5.32106996	4.690013679	5.567566343	2873	Cdc42se2	9.508752271	9.596683399	9.416692733	5173	Fam47e	2.069250695	2.684606187	0.97860816	7473	Kat6b	8.384039812	8.349892323	8.587571991	9773	Nynrin	5.748533338	5.624399604	5.439435794	12073	Rtn4	11.66839939	11.72422348	11.89138571	14373	Tmtc3	5.368767342	5.147421582	6.131538981
574	6030419C18Rik	7.604554328	7.980456351	7.70794764	2874	Cdc45	1.685398	3.455014679	1.976778936	5174	Fam49a	9.838661936	9.897574185	9.800178625	7474	Kat7	8.935154927	8.946926996	8.982898232	9774	Nyx	0.052713236	2.898154009	0.982173627	12074	Rtn4ip1	5.857332345	5.822941677	5.022377828	14374	Tmtc4	7.034130419	6.789253957	7.355761919
575	6030458C11Rik	6.9277265	7.141696572	7.169873176	2875	Cdc5l	9.069074416	9.012799863	9.035287506	5175	Fam49b	9.969377001	10.10365589	10.21964728	7475	Kat8	6.692716169	6.71335911	6.638688215	9775	Oaf	6.616368186	6.228681417	6.208561087	12075	Rtn4r	7.925682955	8.029305215	8.124966322	14375	Tmub1	7.043406538	6.769639038	6.862969233
576	6230400D17Rik	3.405367324	4.572873368	4.305314646	2876	Cdc7	4.723639402	4.889032975	4.735103674	5176	Fam50a	5.756434318	5.986833253	6.170256589	7476	Katna1	6.55421556	6.262095538	6.355021021	9776	Oard1	5.634840064	5.911475184	5.7618418	12076	Rtn4rl1	8.029258411	8.080837486	8.134841284	14376	Tmub2	9.022711557	8.936096143	9.096463597
577	6330403A02Rik	8.779708696	8.434167955	8.288205829	2877	Cdc73	7.229258767	7.123691466	7.056733256	5177	Fam53a	6.271424861	5.978906847	6.609953665	7477	Katnal1	9.727951759	9.561926686	9.823960778	9777	Oas1a	0.013875186	1.507515618	0.990438399	12077	Rtp4	5.154511146	4.22612044	4.789312579	14377	Tmx1	9.335071127	9.162221384	8.936975461
578	6330403K07Rik	9.871716439	9.670132573	9.848800983	2878	Cdca2	2.649988028	3.70351514	2.304281272	5178	Fam53b	7.347288751	7.547780118	7.511644905	7478	Katnal2	0.05031789	2.703196372	2.308079081	9778	Oas1c	1.066480282	1.879110645	2.572165719	12078	Rttn	4.313173586	3.289427148	3.681485549	14378	Tmx2	9.93383271	9.949078628	9.873273904
579	6330407A03Rik	0.019306983	0.910071504	2.575862524	2879	Cdca3	2.389295837	3.208536338	3.155880474	5179	Fam53c	8.947409775	8.747420345	8.734824039	7479	Katnb1	7.829047297	7.779295141	7.985732567	9779	Oasl2	4.056279901	3.18056996	2.981751628	12079	Rufy1	7.963197301	8.161526565	8.002400844	14379	Tmx3	9.242797191	9.279143827	9.116941156
580	6330408A02Rik	6.885725197	6.493126374	6.787657749	2880	Cdca4	5.634364027	5.219393177	5.652660256	5180	Fam57a	0.024692876	0.899614414	1.98907839	7480	Katnbl1	7.046975071	7.434446865	7.355429213	9780	Oat	8.157730075	8.190584211	8.333225219	12080	Rufy2	9.402400926	9.365326128	9.469887699	14380	Tmx4	10.89273437	10.76912942	10.98492581
581	6330409D20Rik	3.985201955	3.523120677	3.980495654	2881	Cdca7	4.976142285	4.657145831	3.884679802	5181	Fam57b	7.113715491	7.142523496	7.338002706	7481	Kazald1	3.262861079	3.526434159	4.068717657	9781	Oaz1	2.060898698	2.470761051	2.571309357	12081	Rufy3	11.26423906	11.2197853	11.30078071	14381	Tnc	4.718178379	4.757804368	4.227218643
582	6330415B21Rik	4.783357344	4.524100344	5.655070843	2882	Cdca7l	2.874359684	3.466571687	2.983716662	5182	Fam58b	6.249464892	6.141125629	6.372878546	7482	Kazn	6.783963779	7.307769695	7.077901777	9782	Oaz1-ps	9.60794827	9.569183002	9.640016641	12082	Rundc1	7.188934941	7.195751739	7.109156914	14382	Tnfaip1	8.244615657	7.918735864	8.333427652
583	6330415G19Rik	3.532550606	2.619744243	3.983687658	2883	Cdca8	3.255647905	4.027337228	4.443527892	5183	Fam60a	2.680984549	3.295564861	3.983006626	7483	Kbtbd11	9.088436402	8.862841344	8.874794576	9783	Oaz2	9.761387047	9.694805798	9.536492261	12083	Rundc3a	11.1131991	10.98440696	10.91513865	14383	Tnfaip2	3.077696502	3.311179641	3.683833631
584	6330416G13Rik	5.726236143	5.76613381	5.934413497	2884	Cdh1	5.317159501	3.896046547	4.935560229	5184	Fam63a	7.445686443	7.498933646	7.139618344	7484	Kbtbd13	1.056744682	1.894935168	0.98564233	9784	Oaz3	4.691329278	8.246694536	4.496171775	12084	Rundc3b	7.540564775	7.781925214	7.714807274	14384	Tnfaip3	2.663714193	2.647119841	2.981916082
585	6330418K02Rik	5.038007605	5.518371725	5.302175026	2885	Cdh10	6.95321249	7.6038849	7.346478544	5185	Fam63b	9.780886405	9.64335215	9.733210239	7485	Kbtbd2	7.855909947	7.992888413	8.134996577	9785	Obfc1	3.688886434	4.887905942	5.229944234	12085	Runx1	2.662302223	3.32648349	2.98223392	14385	Tnfaip6	6.927204017	7.292226604	6.760712473
586	6330549D23Rik	3.257804674	3.750899539	4.30512404	2886	Cdh11	9.010541581	8.970888801	8.648789942	5186	Fam65a	8.877556427	8.850143742	8.601986332	7486	Kbtbd3	5.754488983	5.765984399	6.024867511	9786	Obscn	2.080664237	2.161202112	3.154470158	12086	Runx1t1	9.683962308	9.515390857	9.764707866	14386	Tnfaip8	5.693385687	5.956373034	4.977636104
587	6430411K18Rik	2.382190374	1.434380182	2.793617878	2887	Cdh12	7.082338532	7.05324024	7.645829818	5187	Fam65b	8.671014061	8.094631271	8.270113665	7487	Kbtbd4	6.208474549	6.179602634	5.910161949	9787	Oca2	2.080652346	3.798283186	0.974260056	12087	Runx2	4.722332788	4.476829386	4.623910922	14387	Tnfaip8l1	5.281039686	5.017239108	4.299882498
588	6430503K07Rik	2.8743687	3.585253835	2.983714475	2888	Cdh13	7.737443577	7.390876063	7.806948289	5188	Fam69a	8.868254672	8.860544351	8.790551123	7488	Kbtbd6	2.072519483	3.208586133	2.30476581	9788	Ocel1	4.163826316	3.519666233	3.436579341	12088	Rusc1	9.15684359	9.040386698	8.985630286	14388	Tnfaip8l2	2.887206543	3.314439176	3.568241969
589	6430531B16Rik	2.38112515	0.838352856	1.983852928	2889	Cdh15	2.875141287	0.773498838	2.790074879	5189	Fam69b	9.562272814	9.522601501	9.50745797	7489	Kbtbd7	7.27006627	7.314752512	7.189372029	9789	Ociad1	10.69805543	10.79576585	10.69250449	12089	Rusc2	8.766576373	8.645354268	8.819345214	14389	Tnfaip8l3	7.872614621	7.660164667	6.909716908
590	6430548M08Rik	10.17179457	9.953996609	10.1373697	2890	Cdh16	1.035281373	0.927234387	0.991145826	5190	Fam69c	4.247716291	4.304390651	4.067250156	7490	Kbtbd8	4.34368508	5.302816844	5.228347173	9790	Ociad2	10.01839287	10.02323425	9.834853615	12090	Ruvbl1	7.308245169	7.299260563	6.88635112	14390	Tnfrsf10b	2.398975941	3.193206251	3.30657996
591	6430550D23Rik	1.050310275	2.232657102	1.572867232	2891	Cdh18	4.724028174	5.017856142	5.627205602	5191	Fam71d	0.016600372	1.921606679	0.989760481	7491	Kcmf1	8.819022862	8.884619198	9.088599435	9791	Ocln	3.552676801	4.553398273	4.44087812	12091	Ruvbl2	4.663307234	4.847969979	4.624201968	14391	Tnfrsf11a	3.529866202	3.438774424	3.567567071
592	6430571L13Rik	4.330403636	4.35860255	4.226925397	2892	Cdh19	3.656948348	3.861065206	2.97861984	5192	Fam71e1	3.41969693	4.860914289	5.027896656	7492	Kcna1	10.74154996	10.88293425	10.96085672	9792	Ocm	2.055540009	2.480537452	1.569747218	12092	Rwdd1	9.16513521	9.104773282	9.016124139	14392	Tnfrsf11b	2.651695913	1.402591078	3.445639411
593	6430573F11Rik	5.401105627	4.388999173	5.959410662	2893	Cdh2	9.335392758	9.276745002	9.308693132	5193	Fam71f1	1.064670836	3.372721869	0.983593748	7493	Kcna2	9.986280079	9.957380037	10.5697232	9793	Ocrl	8.337188247	8.150643837	8.342116699	12093	Rwdd2a	7.555114413	7.062357035	7.495878015	14393	Tnfrsf12a	2.672069546	2.118207289	3.7910845
594	6430584L05Rik	3.866234229	2.13728199	3.685088851	2894	Cdh20	6.30804	5.805833181	6.406832089	5194	Fam71f2	0.029917655	2.723016123	0.986734153	7494	Kcna3	0.090147181	2.670478261	1.563127004	9794	Odc1	6.987959844	7.161379472	7.002258175	12094	Rwdd2b	6.143829919	6.289206751	5.622861527	14394	Tnfrsf13b	1.058796697	1.891580121	0.985113014
595	6430706D22Rik	6.504296146	6.138710047	6.456209033	2895	Cdh22	6.382613962	6.298760531	6.389811271	5195	Fam72a	3.394738588	3.868376372	2.786094224	7495	Kcna4	7.680839633	7.738624957	7.549564567	9795	Odf1	2.71017091	6.521442999	2.971475113	12095	Rwdd3	4.837307545	5.406126101	4.733952774	14395	Tnfrsf13c	0.040218715	0.871722901	1.57058689
596	6530402F18Rik	3.863341207	3.57834956	1.560191313	2896	Cdh26	1.060794983	1.888323437	0.984596871	5196	Fam73a	9.611470919	9.33150933	9.455758246	7496	Kcna5	3.771494591	2.37784537	3.889685731	9796	Odf2	7.515199625	7.771414859	7.406076194	12096	Rwdd4a	8.107778551	7.919557609	7.951513073	14396	Tnfrsf19	8.701615467	8.411397801	8.656144965
597	6530403H02Rik	5.021866812	4.298839429	4.066523375	2897	Cdh4	6.27323721	6.036546655	7.003384096	5197	Fam73b	6.96219702	7.020527499	6.837949513	7497	Kcna6	9.263039232	9.284848932	9.276888659	9797	Odf2l	5.629422429	5.040913077	5.566125135	12097	Rxfp3	2.07878762	2.438098571	1.562768727	14397	Tnfrsf1a	5.745559751	5.575542271	4.732720537
598	6720468P15Rik	0.040270581	0.871633669	0.984594817	2898	Cdh5	4.547812194	5.16595872	5.407923271	5198	Fam76a	7.938172661	7.898837046	8.057030862	7498	Kcnab1	7.933160036	8.07109642	8.363950592	9798	Odf3b	2.887091742	3.777464795	3.304094031	12098	Rxra	6.455400363	6.422491966	6.838908999	14398	Tnfrsf1b	4.539716782	4.649370249	4.888402914

599	6720489N17Rik	4.978563714	4.984231869	4.681410749	2899	Cdh6	2.884258098	3.177192035	3.15203456	5199	Fam76b	6.192270412	6.67437279	6.519243531	7499	Kcnab2	10.77577511	10.88924081	10.72817157	9799	Odf3l2	1.066533916	1.879024083	0.983110574	12099	Rxrb	7.611912139	7.727899018	7.60934506	14399	Tnfrsf21	8.956741295	8.978118734	8.738134451
600	6820408C15Rik	5.65045915	5.477322105	4.564608705	2900	Cdh7	5.340185377	5.521916456	6.639448633	5200	Fam78a	3.081691352	3.160257324	3.440911415	7500	Kcnab3	7.613164371	7.433399679	8.351270196	9800	Ofd1	6.523758337	6.736048224	6.624020952	12100	Rxrg	6.246694939	5.788400188	6.109801665	14400	Tnfrsf23	1.643337511	1.461082226	1.570142852
601	6820431F20Rik	6.949074865	6.643445289	6.850590438	2901	Cdh8	7.572649662	7.67138236	7.587279975	5201	Fam78b	6.187849559	6.414743258	6.566226599	7501	Kcnb1	10.09045663	10.03355302	10.44665956	9801	Ogdh	9.642630552	9.625998165	9.634659214	12101	Rybp	7.289264806	7.230747632	7.256232297	14401	Tnfrsf25	2.659616271	2.655634819	1.560427102
602	7420461P10Rik	2.414193823	1.336400336	3.567972889	2902	Cdh9	5.72186832	5.750230882	5.565029865	5202	Fam81a	10.19538825	10.10948666	10.29748053	7502	Kcnb2	6.818973105	6.699479892	6.246097587	9802	Ogdhl	1.634655417	1.480237048	1.572382496	12102	Ryk	8.646547838	8.816249129	8.579859796	14402	Tnfrsf4	2.64075858	1.437666304	1.983874563
603	8030443G20Rik	2.060848048	2.698817885	2.307477749	2903	Cdhr1	4.405380993	4.751257951	4.147883843	5203	Fam83a	2.37408451	1.881905966	1.56972476	7503	Kcnc1	9.445346536	9.443948878	10.24222617	9803	Ogfod1	8.766773647	8.47680383	8.89901431	12103	Ryr1	4.086000779	4.65553489	4.68185856	14403	Tnfrsf9	2.869893124	3.345357381	1.980662565
604	8030462N17Rik	3.536028223	3.545884684	3.682060135	2904	Cdhr2	1.639990116	0.876014996	0.985105648	5204	Fam83d	3.651583794	3.442454839	2.298300596	7504	Kcnc2	8.443180392	8.670933243	8.670009889	9804	Ogfod2	7.472271134	7.547221384	7.527110879	12104	Ryr2	10.18512969	10.24742103	10.14990664	14404	Tnfsf10	3.646906148	3.022995084	3.152458277
605	8430408G22Rik	4.591170554	2.964869841	3.437079016	2905	Cdhr3	3.387924194	3.571998462	3.305118009	5205	Fam83e	1.043230318	0.910088871	1.574431333	7505	Kcnc3	8.71801919	8.884309226	9.159986108	9805	Ogfod3	4.7875496	4.826723089	5.535954678	12105	Ryr3	6.900056062	6.460584414	6.707650976	14405	Tnfsf12	4.895129112	5.486550643	5.595696424
606	8430419L09Rik	8.36132782	8.419458753	8.48392489	2906	Cdip1	10.94234387	10.89793421	10.6611868	5206	Fam83f	0.042826078	1.46116546	0.984086207	7506	Kcnc4	7.873472874	7.480187222	7.741723338	9806	Ogfr	6.65293741	7.269093593	6.825252093	12106	S100a1	10.07618382	9.958027576	10.0740968	14406	Tnfsf13	4.476103409	5.019745905	5.230211024
607	8430426J06Rik	1.650922899	2.20096056	1.568179059	2907	Cdipt	8.753091978	8.843770339	8.761325541	5207	Fam83h	1.652438618	2.198323896	1.984187526	7507	Kcnd1	4.172538715	4.653940113	5.11213921	9807	Ogfrl1	11.46097905	11.47726793	11.24733796	12107	S100a10	7.471610967	7.194113201	7.189266845	14407	Tnfsf13b	2.076632126	2.169322419	2.791255113
608	8430429K09Rik	7.086310772	6.773856844	5.860819382	2908	Cdk1	3.658398106	3.761823165	3.440096702	5208	Fam84a	7.548408055	7.502168062	7.687487529	7508	Kcnd2	10.13198607	10.10766886	10.53271234	9808	Ogg1	8.6764056	8.655325196	8.627487064	12108	S100a11	5.860225441	6.293726972	6.068485013	14408	Tnfsf9	0.048014222	1.45391887	1.98551482
609	8430431K14Rik	2.054157247	1.885135443	1.986255174	2909	Cdk10	7.744839211	7.451170619	7.534525883	5209	Fam84b	5.919688252	6.833654343	6.337261001	7509	Kcne1l	5.74938488	5.510687203	5.148565878	9809	Ogn	6.166792058	5.943449482	5.812081351	12109	S100a13	8.235416204	8.276876286	7.956953197	14409	Tnfsfm13	4.542656521	5.059196939	5.230056855
610	9030025P20Rik	5.864301108	5.785474265	6.472990714	2910	Cdk11b	9.287223766	9.224073165	9.127183024	5210	Fam89a	4.394684698	3.264893345	4.067880808	7510	Kcne2	4.482110464	6.13469675	3.974824033	9810	Ogt	8.742543924	8.86882471	8.800157244	12110	S100a16	9.314227024	9.618620345	9.201500312	14410	Tnik	9.782623145	9.659422438	9.801793229
611	9030612E09Rik	2.086494852	3.330816728	3.444093322	2911	Cdk12	8.270159054	8.065394801	8.288396869	5211	Fam89b	6.127340587	6.40921816	6.488178526	7511	Kcne4	2.886023709	3.17388549	2.563638769	9811	Oip5	1.1030095	3.336090876	1.978872113	12111	S100a4	5.540490544	5.673322161	6.069008543	14411	Tnip1	7.093335914	7.700658603	7.283734869
612	9030617O03Rik	4.331496648	4.895689596	4.439190715	2912	Cdk13	8.450989378	8.39248381	8.451723245	5212	Fam8a1	3.25894287	3.413070778	4.304916608	7512	Kcnf1	6.911920287	6.58998645	7.329875041	9812	Ola1	10.20059889	10.06065442	10.04876322	12112	S100a6	7.913795243	8.270755055	8.056886624	14412	Tnip2	5.568826611	5.515574861	5.108398861
613	9030624G23Rik	0.008386612	0.933563535	0.991897072	2913	Cdk14	10.90621317	10.68394728	10.67083295	5213	Fam92a	7.814202232	7.54423641	7.708018852	7513	Kcng1	3.50776647	2.459688805	0.979457788	9813	Olfm1	12.74124701	12.77204769	12.55669007	12113	S100a8	2.64828055	2.17758176	2.568839605	14413	Tnk1	4.988403149	5.04727426	5.302905198
614	9030624J02Rik	7.114625604	7.230578488	7.246856451	2914	Cdk15	1.641633265	2.485710086	2.309370979	5214	Fam92b	3.227337278	1.836891631	0.976165117	7514	Kcng2	6.247274067	6.145438374	5.146818921	9814	Olfm2	1.643350865	0.867126074	1.570139401	12114	S100a9	3.406802972	4.570770409	1.96829764	14414	Tnk2	9.483395893	9.298922956	9.616714627
615	9130008F23Rik	1.032340788	0.933566573	0.991897432	2915	Cdk16	9.314177858	9.388882536	9.55185744	5215	Fam96a	7.494988558	6.677547317	6.825059847	7515	Kcng4	2.442897004	3.835263786	4.841897672	9815	Olfm3	7.681117693	7.590522487	8.26594294	12115	S100b	11.45888459	11.20601983	11.4607687	14415	Tnks	7.565646878	7.577052702	8.030138498
616	9130011E15Rik	2.67316209	4.123785488	3.441488689	2916	Cdk17	9.621731892	9.48287499	9.931376317	5216	Fam96b	6.822774432	6.813542968	6.922529052	7516	Kcnh1	8.131004478	8.389481765	8.519380512	9816	Olfml1	6.822516292	7.098648844	6.922592126	12116	S100pbp	3.399683738	3.659974773	3.302077797	14416	Tnks1bp1	6.767332261	6.803713804	6.652082525
617	9130019O22Rik	4.531030496	3.829078475	4.303129589	2917	Cdk18	5.834352821	5.840000491	5.502666814	5217	Fam98a	6.832804685	6.537949674	6.63839778	7517	Kcnh2	5.087041317	5.892768533	5.534750408	9817	Olfml2b	7.538702551	7.385244286	6.886024154	12117	S1pr1	9.500615353	9.541705631	9.557417381	14417	Tnks2	9.12749814	9.237420417	9.114448452
618	9130019P16Rik	6.663063062	6.497559227	6.638733786	2918	Cdk19	9.879889125	10.04887445	10.12088702	5218	Fam98b	8.097977033	8.260077016	8.232646254	7518	Kcnh3	8.173489212	8.260269343	8.635019102	9818	Olfml3	5.908983697	5.673242961	5.65266726	12118	S1pr2	3.399223343	3.299084519	3.789910897	14418	Tnmd	3.650199951	3.964187308	2.563462989
619	9130023H24Rik	3.084962544	2.994564843	4.232060887	2919	Cdk2	3.528148548	3.672566602	3.441922073	5219	Fam98c	8.194606858	8.301626013	8.350755613	7519	Kcnh4	2.096891619	2.637963873	3.304365569	9819	Olfr239	0.011140597	0.927248116	1.991605264	12119	S1pr3	4.405865686	4.142527217	4.981637879	14419	Tnnc1	3.676870496	4.431224788	5.027771257
620	9130024F11Rik	4.184125631	5.2732016	5.865705228	2920	Cdk20	4.883710943	4.140045646	4.88790873	5220	Fan1	3.062701809	1.827432233	1.562437463	7520	Kcnh5	3.670034544	3.124644143	4.841940632	9820	Olfr287	4.241048542	3.529137338	2.975445781	12120	S1pr5	7.178737142	7.657421711	7.129606836	14420	Tnnc2	0.047865209	0.858766981	3.690283845
621	9130221H12Rik	4.728562163	4.737021834	5.338992414	2921	Cdk2ap1	8.760456002	8.646974132	9.172452173	5221	Fanca	2.40235027	2.8544603	3.443986611	7521	Kcnh7	5.993279662	6.496039536	7.171268891	9821	Olfr856-ps1	3.233883376	1.37878261	3.306238336	12121	Saa1	4.884063901	1.983926742	3.29361749	14421	Tnni1	5.072901382	4.74918278	4.735391855
622	9130401M01Rik	8.045628505	7.749205247	7.85028555	2922	Cdk2ap2	6.681733745	6.743430991	6.680116332	5222	Fancb	3.070365916	3.454834503	3.152733979	7522	Kcnh8	3.08027655	3.667241797	3.303070712	9822	Olfr920	1.65932293	2.457550294	2.305783741	12122	Saa2	4.327571695	0.488893894	2.287044639	14422	Tnni2	2.6474444	1.416407371	4.236983856
623	9230102K24Rik	0.024661595	0.899673654	1.573368474	2923	Cdk4	6.682532204	7.383870329	6.336178614	5223	Fancc	4.608127283	4.790754532	4.887133998	7523	Kcnip1	8.036357536	8.514436107	8.718332267	9823	Olig1	9.422610278	9.662111884	9.288261871	12123	Saal1	5.956316024	5.326094357	5.788499804	14423	Tnni3	2.0583185	0.854506933	2.571858716
624	9230110C19Rik	3.761343951	2.643806727	0.969177863	2924	Cdk5	8.999270478	8.870874985	9.101620564	5224	Fancd2	4.073719327	4.31839714	4.506964188	7524	Kcnip2	10.47381753	10.40494696	10.34411789	9824	Olig2	8.641955096	8.392299732	8.393598887	12124	Sac3d1	6.694109032	6.537548297	6.638347111	14424	Tnnt1	4.353584652	5.106701132	5.911563755
625	9230112J17Rik	2.043177133	1.909342923	1.573372463	2925	Cdk5r1	11.64608623	11.49867889	11.54699138	5225	Fancd2os	4.233199591	3.859517283	3.566407168	7525	Kcnip3	8.766946078	8.934374628	8.809767274	9825	Oma1	6.265197583	6.148918054	5.910954163	12125	Sacm1l	8.189426831	8.103273415	8.337603708	14425	Tnnt2	4.258716076	5.059643734	4.935279844
626	9230114K14Rik	6.36669805	6.482606554	6.708564621	2926	Cdk5r2	10.74947621	10.89802355	10.95801419	5226	Fance	6.033349058	6.050303279	6.209648073	7526	Kcnip4	8.314429399	8.102487922	8.519273188	9826	Omd	3.65824317	3.153296207	4.306294637	12126	Sacs	5.042376703	5.25665319	5.439172071	14426	Tnp1	5.981106585	10.33054418	8.587433809
627	9230116N13Rik	1.056756492	2.491138257	1.987398014	2927	Cdk5rap1	5.666785134	5.862824807	6.190744093	5227	Fancf	4.240163697	4.780363457	4.230381622	7527	Kcnj10	8.852962435	8.880672397	9.38734842	9827	Omg	8.627467208	8.6867972	8.673506045	12127	Sae1	9.890987404	9.822318978	9.909285191	14427	Tnp2	2.712978115	6.458803553	4.439604646
628	9230117E06Rik	2.848857084	1.476168034	1.571905698	2928	Cdk5rap2	5.892074487	6.742696964	5.759897214	5228	Fancg	4.876190317	4.367568315	3.88528567	7528	Kcnj11	7.155809923	6.824294376	7.209279337	9828	Onecut1	0.052726767	2.203541143	3.310532839	12128	Safb	8.699148481	8.75964922	9.03002065	14428	Tnpo1	4.997089919	6.039062552	5.838823732
629	9330020H09Rik	5.42734275	4.472006184	4.564166793	2929	Cdk5rap3	6.206617476	5.68934121	6.109774254	5229	Fanci	4.722688075	5.249325858	5.026014359	7529	Kcnj12	5.752602537	5.350713437	5.838334725	9829	Onecut2	7.34799032	7.48943654	7.274326574	12129	Safb2	8.015782867	7.637552635	7.680333217	14429	Tnpo2	9.242764619	9.049191526	9.281547959
630	9330102E08Rik	6.605232308	6.873847316	6.707875867	2930	Cdk6	1.062700442	2.48313578	0.984104051	5230	Fancl	5.911834441	6.24061934	5.812950546	7530	Kcnj13	6.665550743	7.653157428	4.72754368	9830	Onecut3	0.016616161	0.915523586	1.574991333	12130	Sag	4.924044624	4.163216026	3.297484458	14430	Tnpo3	9.910229219	9.791787781	9.928372013
631	9330117O12Rik	3.53198662	3.864273852	2.979139289	2931	Cdk7	8.388344316	8.177081544	8.527141253	5231	Fancm	6.30578147	5.609043799	6.424105016	7531	Kcnj14	1.66807017	2.171190233	2.304026255	9831	Ooep	1.083908637	2.684440039	0.978571442	12131	Sall1	5.75252637	5.616386008	6.44149357	14431	Tnr	6.210680463	6.061665646	6.372538772
632	9330133O14Rik	6.521485734	6.570466487	6.337552939	2932	Cdk8	5.755782229	5.880097707	5.707060629	5232	Fank1	4.398546942	5.263054459	4.066827589	7532	Kcnj16	8.559147488	7.964687545	8.427235824	9832	Opa1	9.100177027	8.971592408	9.459959616	12132	Sall2	7.650802777	7.54325846	7.89310534	14432	Tnrc18	7.986093447	7.744858476	7.862801394
633	9330136K24Rik	3.38294969	2.858728584	2.983194906	2933	Cdk9	8.023343381	8.066527549	8.14998668	5233	Fap	1.698454738	3.960151507	2.980062493	7533	Kcnj2	6.511990898	7.442645797	7.337994004	9833	Opa3	8.146042781	8.062239292	8.174919436	12133	Sall3	5.398055115	5.08479606	5.373287185	14433	Tnrc6a	9.31939383	9.43178908	9.591036376
634	9330151L19Rik	6.146202313	6.215208073	6.169984222	2934	Cdkal1	7.420740941	7.600908815	7.557546424	5234	Far1	7.481505161	7.429526737	7.624040014	7534	Kcnj3	10.0635238	10.07871765	10.0989451	9834	Opalin	8.086236828	8.481098094	7.996679309	12134	Samd1	6.794838364	6.838492224	6.874966511	14434	Tnrc6b	9.576527483	9.628963514	9.772831534
635	9330159F19Rik	9.287306871	8.867034737	9.253826743	2935	Cdkl1	6.938988488	6.823792654	7.390101505	5235	Far1os	0.037671274	1.891619716	1.571018712	7535	Kcnj4	9.539819545	9.645879215	9.574367245	9835	Opcml	10.12214726	10.08580403	10.09373576	12135	Samd10	6.52332698	6.510319147	6.863448951	14435	Tnrc6c	8.672476989	8.477812506	8.4837822
636	9330159M07Rik	3.542185155	3.136660922	4.627996214	2936	Cdkl2	8.397350865	8.238660639	8.515519443	5236	Far2	8.45121755	8.403605543	8.495731997	7536	Kcnj5	1.649545931	0.850417424	1.568536108	9836	Ophn1	6.456100113	6.580738297	6.76194998	12136	Samd11	6.962568801	6.989174912	6.812713933	14436	Tns1	7.540566155	7.895847569	7.159132879
637	9330162012Rik	4.072961604	4.31984796	3.680409989	2937	Cdkl3	5.545443033	6.009549591	6.424303836	5237	Farp1	6.487327549	6.441779614	6.488416058	7537	Kcnj6	7.101215822	6.545954923	6.623239895	9837	Oplah	6.104781547	6.571945611	6.066885892	12137	Samd12	5.808362193	6.073194454	6.190609737	14437	Tns2	4.18133497	4.527126918	4.887093535
638	9330175M20Rik	2.387318415	1.849022466	3.795512652	2938	Cdkl4	5.882272014	5.824401334	5.503665504	5238	Farp2	7.091457508	7.405013761	7.140027053	7538	Kcnj8	4.397707543	3.110544684	3.785455932	9838	Opn1mw	1.073726277	2.198403753	1.567797644	12138	Samd14	6.805248106	6.481862893	6.246171578	14438	Tns3	7.579770208	7.429678992	7.479831975
639	9330179D12Rik	4.234133623	3.760911874	3.566121422	2939	Cdkl5	7.773985574	7.894435148	7.881005675	5239	Fars2	5.985987492	6.323743821	5.837854911	7539	Kcnj9	7.932484278	7.951422003	8.213882759	9839	Opn3	4.474192173	4.066758016	5.152341328	12139	Samd15	4.474366915	5.022848325	5.069544665	14439	Tns4	2.649141177	3.348271784	2.791856641
640	9330182L06Rik	7.596501669	7.435400001	8.279993704	2940	Cdkn1a	7.374322922	6.674328693	6.720397122	5240	Farsa	8.480734189	8.412304559	8.341968934	7540	Kcnk1	9.556309228	9.587781827	9.429071087	9840	Opn4	2.073591885	2.447592	1.564349208	12140	Samd3	2.073642775	3.348174413	2.304504868	14440	Tob1	7.231352774	7.271034	7.471986182
641	9330188P03Rik	1.06466914	1.88203743	1.569742288	2941	Cdkn1b	8.303503254	8.104961316	8.179783517	5241	Farsb	9.982552229	9.74473545	9.611108785	7541	Kcnk10	6.413605422	5.717405865	5.404720453	9841	Oprd1	2.387270598	2.881101737	2.305282855	12141	Samd4	8.490206425	8.665035605	8.711425898	14441	Tob2	7.580384299	7.318501096	7.526804699
642	9430015G10Rik	5.12312321	4.965488759	5.596815001	2942	Cdkn1c	7.124254532	7.70459028	6.502369465	5242	Fas	3.383569316	2.85719745	1.978124793	7542	Kcnk12	3.402614652	2.809734346	3.681749664	9842	Oprk1	4.727079109	4.836423527	4.786953945	12142	Samd4b	8.880978116	8.838492556	8.734881378	14442	Toe1	6.03793179	6.280221977	6.535163977
643	9430016H08Rik	6.124999187	5.632622216	6.045828026	2943	Cdkn2aip	6.251878994	6.039832821	6.63884542	5243	Fasn	9.143702577	9.316830939	9.062145393	7543	Kcnk13	5.24830466	5.302933529	5.407355976	9843	Oprl1	6.86115075	6.642829806	6.922813903	12143	Samd5	5.091941554	5.767465755	6.521134877	14443	Tollip	8.627167939	8.636082987	8.451579747
644	9430018G01Rik	0.008383876	0.933569984	1.576874431	2944	Cdkn2aipnl	7.920049412	7.9140561	8.046154754	5244	Fastk	7.392911851	7.32171522	7.439620358	7544	Kcnk2	9.240373301	9.307867723	9.402150759	9844	Oprm1	4.98683814	5.273881898	5.229000245	12144	Samd8	9.593879871	9.392430758	9.322025304	14444	Tom1	5.659489551	5.33218088	5.337882741
645	9430020K01Rik	9.023273271	9.141801936	9.337672921	2945	Cdkn2b	1.65521201	1.861990154	2.570528859	5245	Fastkd1	4.484854013	5.432055344	5.067253271	7545	Kcnk3	7.628785102	8.09256848	8.209200785	9845	Optn	6.145371154	6.180851213	5.837329878	12145	Samd9l	4.830628121	4.700654381	5.111504137	14445	Tom1l1	5.837208925	6.068894616	6.067734677
646	9430021M05Rik	5.20359682	4.343475962	4.980646312	2946	Cdkn2c	4.078139217	4.820484884	4.505979505	5246	Fastkd2	6.363304085	6.02521342	5.95674338	7546	Kcnk4	3.555610693	4.991251603	4.840484852	9846	Orai1	5.63453481	5.417801744	5.534368595	12146	Samhd1	6.010456255	5.835092933	6.088943881	14446	Tom1l2	10.09459392	10.09046314	10.1870253
647	9430037G07Rik	1.660582465	2.882785389	3.156460346	2947	Cdkn2d	9.004743115	8.729277363	8.862682145	5247	Fastkd3	6.554254251	5.80206126	6.653137309	7547	Kcnk5	3.211810043	0.871462983	0.984574939	9847	Orai2	7.673336209	7.948560793	7.54958622	12147	Samm50	8.555974535	8.485408714	8.372215504	14447	Tomm20	9.955445204	9.799017711	9.991248296
648	9430038I01Rik	5.209242916	5.367102904	5.472947497	2948	Cdo1	7.815270895	7.662786821	7.812871768	5248	Fastkd5	5.282985687	5.05391762	4.501967374	7548	Kcnk6	4.228045348	2.398452593	3.567986181	9848	Orai3	6.780062835	6.874590748	6.455173848	12148	Samsn1	4.822273915	4.768464211	3.435939831	14448	Tomm20l	5.545401541	5.78843355	6.046426672

649	9430041J12Rik	1.671569007	0.787364867	3.154808189	2949	Cdon	4.884647892	4.473427607	4.62350502	5249	Fat1	6.4698438	6.412253337	6.189424742	7549	Kcnk7	1.6399659	1.468541711	1.571013493	9849	Oraov1	5.469233046	5.428531541	5.302187328	12149	Sap130	6.008656318	5.502006239	5.680009125	14449	Tomm22	8.646257832	8.649084204	8.380721249
650	9430060I03Rik	3.764830634	2.122653331	3.151199455	2950	Cdpf1	5.251614711	4.955222357	5.624910005	5250	Fat3	8.662786803	8.836893032	9.447837961	7550	Kcnma1	10.77559893	10.36394024	10.7340743	9850	Orc1	1.043246073	0.910054793	1.99002169	12150	Sap18	2.056965264	1.878915438	1.569325923	14450	Tomm34	7.981729777	8.429671537	7.892695047
651	9430083A17Rik	4.890009966	5.372117699	5.59682104	2951	Cdr1	10.46913071	10.45792508	11.76019201	5251	Fat4	5.471302989	5.328479662	5.504041708	7551	Kcnmb1	2.401500896	3.791785864	3.153507949	9851	Orc2	8.062858871	7.876313802	7.904776922	12151	Sap30	4.333393894	4.799164174	4.564851076	14451	Tomm40	8.410956922	8.317889682	8.389481079
652	9430091E24Rik	1.080528475	1.856628849	1.566347995	2952	Cdr2	6.668404338	6.902490987	7.046179911	5252	Fau	10.59323803	10.55727225	10.48379999	7552	Kcnmb2	6.750714853	6.342099228	6.761917826	9852	Orc3	9.323743556	9.489513882	9.431249191	12152	Sap30bp	5.909617361	5.416879599	5.405453917	14452	Tomm40l	7.707385663	7.139195841	7.246653633
653	9530027J09Rik	4.81522379	3.750263407	3.788249877	2953	Cdr2l	6.585754074	6.020574717	6.423153653	5253	Faxc	8.053117581	8.199091544	8.572708752	7553	Kcnmb4	8.10762899	8.23709348	7.934180426	9853	Orc4	9.121656774	8.941267441	9.099047569	12153	Sap30l	7.618733843	7.444944635	7.363530649	14453	Tomm5	7.802558752	7.982528349	8.08339481
654	9530036O11Rik	3.046336565	1.878875259	1.985525007	2954	Cds1	8.357062515	8.437614474	8.714913866	5254	Fbf1	7.238414634	7.216340304	6.734349511	7554	Kcnmb4os1	4.77199965	5.073129446	3.884931026	9854	Orc5	6.985278898	6.804743898	6.594360611	12154	Sapcd1	1.649507391	1.447349532	2.987779988	14454	Tomm6	9.845527903	9.925341376	9.9107234
655	9530052E02Rik	1.054701038	0.885103394	0.986168805	2955	Cds2	9.498737774	9.706747452	9.678690319	5255	Fbl	7.032360754	6.816124769	6.694090266	7555	Kcnmb4os2	4.890655025	5.163241027	5.109508148	9855	Orc6	7.35577499	7.289869315	7.088252721	12155	Sapcd2	2.063405229	2.466187556	1.983863148	14455	Tomm6os	3.649649572	3.174042785	2.787272892
656	9530059O14Rik	3.539631965	3.142627474	3.438955653	2956	Cdsn	1.643254773	2.483115798	0.984100186	5256	Fblim1	2.66293621	2.416664182	3.305410942	7556	Kcnn1	5.537873322	5.360308633	5.565554129	9856	Orm1	3.538534183	1.73808782	1.969568896	12156	Sar1a	8.498275246	8.4598752	8.385057672	14456	Tomm7	10.97224288	10.83499842	10.72563599
657	9530062K07Rik	2.399956971	2.42767443	3.306398731	2957	Cdt1	3.076707657	3.443233619	3.151182028	5257	Fbll1	8.294125031	8.051550472	8.242208549	7557	Kcnn2	6.190112566	5.894976741	5.956314753	9857	Ormdl1	5.505473329	5.541774471	5.372140025	12157	Sar1b	8.563981186	8.507070013	8.288168377	14457	Tomm70a	9.345153951	9.317220814	9.473868321
658	9530068E07Rik	7.572043439	7.886306112	7.264985745	2958	Cdv3	8.244702157	8.142193128	8.184677274	5258	Fbln1	3.980112474	3.535048365	2.976320113	7558	Kcnn3	5.985411514	5.742496092	6.266678006	9858	Ormdl2	7.610358844	7.453631674	7.088019186	12158	Saraf	10.68410267	10.71762795	10.3385742	14458	Tomt	1.622207617	0.921245945	0.99043517
659	9530077C05Rik	4.775829495	4.420281524	4.83947266	2959	Cdyl	8.09793433	8.203970976	8.274654684	5259	Fbln2	3.875848331	2.380371179	3.682530456	7559	Kcnq1	2.374063817	1.88195688	1.569731549	9859	Ormdl3	7.480526932	7.184396867	7.255957975	12159	Sardh	5.165745407	5.047867761	5.228700476	14459	Tonsl	4.594914343	4.610783482	4.150213758
660	9530080O11Rik	2.877188357	1.814843796	1.978143799	2960	Cdyl2	7.259315686	7.046383924	7.265676516	5260	Fbln5	3.558133444	4.545041336	4.625054683	7560	Kcnq1ot1	9.435864233	8.954821373	9.43326599	9860	Os9	9.413405216	9.317804744	9.30192983	12160	Sarm1	5.979148672	5.361205745	5.337428978	14460	Top1	11.30027901	11.05499251	11.13984545
661	9530082P21Rik	9.701016368	9.763881641	9.968714227	2961	Ceacam1	4.256503359	3.98446768	5.191945554	5261	Fbln7	2.687277661	4.453434411	3.981982338	7561	Kcnq2	9.833430401	9.645233422	9.579875741	9861	Osbp	6.951558611	7.037635816	7.247179523	12161	Sarnp	8.560301395	8.548633972	8.549841427	14461	Top1mt	5.958327551	5.79734747	5.813610386
662	9530091C08Rik	4.483175974	4.576793593	5.338739027	2962	Ceacam10	0.032500593	0.885257796	2.989902972	5262	Fbn1	3.694775807	5.12827107	5.303215559	7562	Kcnq3	4.978377072	4.7551866	4.624636106	9862	Osbp2	10.08051088	10.20125809	10.23979029	12162	Sars	10.12763216	10.10817665	10.05411575	14462	Top2a	5.831484858	5.868582398	5.439035474
663	9630001P10Rik	3.763871614	3.675484907	2.980739283	2963	Ceacam2	3.653226501	2.627357876	3.567573442	5263	Fbn2	4.155955323	4.256433724	3.147885955	7563	Kcnq5	7.98573698	7.638372819	7.70112675	9863	Osbpl10	2.391149682	3.475642597	2.30427655	12163	Sars2	3.662052007	4.107890364	3.439115212	14463	Top2b	9.941643425	9.85160932	10.00791524
664	9630013A20Rik	5.658395484	5.460885751	5.37305957	2964	Ceacam9	1.632762955	1.484391579	1.988643013	5264	Fbp1	1.661823714	3.055714588	2.985798977	7564	Kcnrg	3.766125364	3.008751533	1.973775106	9864	Osbpl11	6.014447653	6.04198426	6.406593356	12164	Sart1	7.801075768	7.676811658	7.542004809	14464	Top3a	2.871426766	3.201182031	2.303552086
665	9630013K17Rik	0.027309241	1.48436902	0.987297687	2965	Cebpa	5.474656712	5.56487058	5.405557483	5265	Fbrs	6.66528983	7.168337521	6.787671732	7565	Kcns1	4.728395864	5.128790429	4.563189597	9865	Osbpl1a	10.7162694	10.57061753	10.9479809	12165	Sart3	8.0462426	8.183494499	8.01889726	14465	Top3b	7.135373246	7.114523623	7.179852383
666	9630028B13Rik	2.388317065	2.451482062	2.985585533	2966	Cebpb	5.722140712	5.322716374	5.300940508	5266	Fbrsl1	7.547877889	7.801170788	7.495674601	7566	Kcns2	6.834942568	6.980679008	6.887107846	9866	Osbpl2	7.428537022	7.385583518	7.337568418	12166	Sash1	8.855119999	8.707779366	8.886953038	14466	Topbp1	6.210842373	6.080914451	6.535182561
667	9630028I04Rik	0.013879578	0.921263769	1.575586589	2967	Cebpd	6.556623082	6.819172976	6.08715695	5267	Fbxl12	6.327120575	6.265346566	6.610381099	7567	Kcns3	4.883061871	5.214310911	4.680756135	9867	Osbpl3	6.665590235	6.417927495	6.863049418	12167	Sash3	3.754823076	2.158754607	2.302592097	14467	Topors	8.415919204	8.384558905	8.279071016
668	9830132P13Rik	8.943952996	8.775001669	8.623792901	2968	Cebpg	8.17352261	8.251701991	8.447812949	5268	Fbxl12os	3.066684194	2.151455373	0.972361685	7568	Kcnt1	6.103494221	5.853042282	6.53558984	9868	Osbpl5	5.806913553	5.670092077	5.502877366	12168	Sass6	5.835931786	5.990397888	5.934373786	14468	Toporsos	6.631750344	6.36256662	6.087892069
669	9830147E19Rik	3.794129655	4.706336631	4.372181326	2969	Cebpz	9.439904159	9.120333967	9.208778654	5269	Fbxl13	0.035104479	2.223104137	0.985644935	7569	Kcnt2	6.166107878	6.106879581	6.504899688	9869	Osbpl6	7.690038755	8.036801587	8.503934695	12169	Sat1	7.169095402	7.120530819	7.527220186	14469	Tor1a	7.011142702	7.057992361	6.825128897
670	9830166K06Rik	2.38636023	2.45546873	3.308913948	2970	Cebpzos	8.337197423	8.016505305	8.213660359	5270	Fbxl14	6.779852429	6.961920695	6.694314998	7570	Kcnu1	4.971243694	3.73355989	4.374008094	9870	Osbpl7	6.660670099	6.299372907	6.088184957	12170	Sat2	5.276746734	4.704529917	3.67620598	14470	Tor1aip1	8.109313216	8.393050513	8.114213084
671	9930014A18Rik	3.780986074	4.316297647	4.229977127	2971	Cecr2	4.788606488	5.78386538	5.227944904	5271	Fbxl15	7.077466801	6.839870592	6.91113606	7571	Kcnv1	8.947163607	8.904950055	9.117251798	9871	Osbpl8	8.540403828	8.533990891	8.974569601	12171	Satb1	9.744036198	9.907255067	9.940082888	14471	Tor1aip2	5.039018718	5.601240937	5.653675361
672	9930021J03Rik	9.985669154	9.673478963	9.868752739	2972	Cecr5	2.89438065	3.301607028	3.566804414	5272	Fbxl16	11.42539864	11.36673664	11.455219	7572	Kcp	1.056760923	1.894908571	1.571456232	9872	Osbpl9	8.447059297	8.64941381	8.511380933	12172	Satb2	6.384322123	6.596872485	7.171554352	14472	Tor1b	8.595310595	8.404331883	8.208670865
673	9930104L06Rik	4.940533202	4.876145701	5.024241605	2973	Cecr6	6.589358638	6.950689399	7.014093386	5273	Fbxl17	8.784323564	8.801084077	8.99139263	7573	Kctd1	8.556357802	8.506266682	8.865909499	9873	Oscp1	7.058618656	7.478707747	6.968304763	12173	Sav1	5.913656887	6.146621454	6.170520774	14473	Tor2a	6.677541783	6.663263258	6.354935411
674	9930111J21Rik1	0.022019256	0.904737515	1.573883499	2974	Cela1	3.781130885	4.097094042	3.980908166	5274	Fbxl18	7.260672923	7.37027144	7.667083494	7574	Kctd10	6.92529546	6.860550837	6.898975466	9874	Oser1	4.784897213	5.088332479	4.886373328	12174	Saxo2	3.059789871	2.171039954	2.98479003	14474	Tor3a	4.984544677	5.471880558	4.370192812
675	a	1.058784982	2.488396002	0.985116038	2975	Celf1	11.34033018	11.42893074	11.3760534	5275	Fbxl19	5.530839765	4.339292864	5.505688546	7575	Kctd11	3.542824299	4.020473325	3.787883962	9875	Osgep	6.557131369	6.841299438	6.680513933	12175	Saysd1	4.618943883	5.651197433	5.337186329	14475	Tor4a	4.466806989	4.663006981	3.785477533
676	A130010J15Rik	6.031393023	5.443457804	5.862927025	2976	Celf2	12.23977177	12.07050055	12.01614747	5276	Fbxl2	7.881108916	7.779988005	8.035540174	7576	Kctd12	8.37948055	8.329851281	8.372365111	9876	Osgepl1	6.555629807	6.405986419	6.609630965	12176	Sbds	9.13569908	9.019944706	9.083275963	14476	Tox	6.682853101	6.892201842	7.265989302
677	A130077B15Rik	2.637650999	1.873090492	0.982152824	2977	Celf3	8.867588979	8.742614066	8.616626256	5277	Fbxl20	6.684061306	7.196768614	7.302278787	7577	Kctd12b	5.858127131	5.934276265	5.107422525	9877	Osgin1	1.10289683	2.662223822	1.978892218	12177	Sbf1	8.410216762	8.182695687	8.184631931	14477	Tox2	5.429486241	3.971009552	4.734257212
678	A230001M10Rik	3.550741803	4.55636304	3.297685168	2978	Celf4	13.31057125	13.04508512	12.9812061	5278	Fbxl21	2.071468762	3.053976728	1.981770126	7578	Kctd13	9.395569177	9.386881093	9.380891065	9878	Osgin2	6.689882831	6.381292406	5.95660403	12178	Sbf2	7.867350916	7.515581732	7.680441735	14478	Tox3	7.076330948	6.372506974	6.77479928
679	A230009B12Rik	5.152638514	4.080645779	4.066745379	2979	Celf5	10.22242815	10.24666256	10.08328685	5279	Fbxl22	7.732036012	7.617164272	8.088852073	7579	Kctd14	6.206282212	5.903646127	5.56375934	9879	Osmr	3.525809536	3.447384708	3.791825461	12179	Sbk1	7.801286445	7.604608712	7.922745143	14479	Tox4	7.900179311	7.8268348	7.645117444
680	A230020J21Rik	0.019293321	1.497692458	1.574432349	2980	Celf6	6.863056299	6.956127279	6.850047803	5280	Fbxl3	7.746229726	7.750135771	7.850405918	7580	Kctd15	4.99089278	5.120847059	5.440327483	9880	Osr1	4.593269748	4.166214782	4.1506748	12180	Sbk3	1.648020291	0.854572054	1.568931405	14480	Tpbg	4.545420113	4.883736229	5.626714939
681	A230046K03Rik	8.095837299	7.694761439	7.957470127	2981	Celrr	4.649051493	4.019597029	3.438002262	5281	Fbxl4	4.010262849	5.579982016	4.979568195	7581	Kctd16	2.407722275	2.84496184	3.792285739	9881	Ost4	8.256570223	8.79650927	8.645405989	12181	Sbno1	10.74053268	10.53623405	10.78170787	14481	Tpcn1	7.022394073	7.008814481	6.922462239
682	A230050P20Rik	7.081442036	7.221882583	7.431598209	2982	Celsr1	4.599536087	4.758504715	3.884458108	5282	Fbxl5	8.451408461	8.496048753	8.479817684	7582	Kctd17	8.519855357	8.778065749	8.797067748	9882	Ostc	9.083044589	9.072012962	8.796857808	12182	Sbno2	4.992396693	5.330940765	5.301969888	14482	Tpcn2	1.097233417	3.043307302	2.567546037
683	A230056J06Rik	4.515347311	3.308062125	1.555074667	2983	Celsr2	9.172395756	8.928437756	8.96021021	5283	Fbxl6	5.568481161	5.117388851	5.839787084	7583	Kctd18	5.563782918	4.632563409	5.109744078	9883	Ostf1	6.88737152	7.127968744	6.77428845	12183	Sbsn	5.692069325	5.702096325	4.784440139	14483	Tpd52	8.178338195	8.302826468	8.077969219
684	A230056P14Rik	5.282793843	4.407023957	5.229473828	2984	Celsr3	6.570236721	6.424039228	6.319722357	5284	Fbxl7	3.24476692	3.773681338	2.786337424	7584	Kctd2	9.060305322	9.22520907	8.907757817	9884	Ostm1	7.328952053	7.2876255	7.638569237	12184	Sbspon	3.071477813	3.682230216	3.443184994	14484	Tpd52l1	7.759394636	7.153651134	7.479582901
685	A230057D06Rik	3.763537927	2.838499219	1.97499683	2985	Cemip	4.085238829	4.42665034	3.978266349	5285	Fbxl8	1.681320473	3.33002725	2.982715634	7585	Kctd20	5.290547506	5.515629036	5.681673932	9885	Otc	0.052770713	0.850633999	1.984858481	12185	Sc5d	8.289891379	8.442821995	8.251439125	14485	Tpd52l2	7.679468257	7.653196615	7.631209615
686	A230065H16Rik	4.395496434	4.818348526	3.436150077	2986	Cend1	9.285364486	9.378868827	9.469870559	5286	Fbxo10	6.43460233	6.35054072	6.302417051	7586	Kctd21	5.630709373	5.804794958	5.535325495	9886	Otof	3.54378748	2.587782547	4.442594962	12186	Scaf1	6.83460353	6.906680903	6.748183296	14486	Tpgs1	7.957197626	8.133798706	7.916545069
687	A230070E04Rik	7.822615222	7.825445088	8.056943167	2987	Cenpa	3.667672452	3.743336829	4.506765187	5287	Fbxo11	9.481319797	9.353265666	9.422929224	7587	Kctd3	7.92593393	7.920140256	7.974425268	9887	Otop2	0.019306113	0.91007324	0.989111113	12187	Scaf11	9.311891654	9.267900051	9.50753389	14487	Tpgs2	8.342626649	8.587803111	8.499773983
688	A230072C01Rik	6.550817125	5.761240886	6.1898264	2988	Cenpb	8.941162669	8.975907083	8.809731676	5288	Fbxo16	4.880658155	4.75455711	4.148355744	7588	Kctd4	9.631053909	9.647321862	8.922190674	9888	OTTMUSG0000001660	6.228978695	5.807581916	6.24714177	12188	Scaf4	7.229294752	6.933366089	7.320238134	14488	Tph1	0.07247666	2.183779874	0.978541909
689	A230072E10Rik	1.080562082	2.459653218	2.570014756	2989	Cenpc1	7.672581177	7.310058722	7.549767245	5289	Fbxo17	3.768054322	3.865783412	3.302980799	7589	Kctd5	6.082879112	5.803822267	5.956315034	9889	Otub1	9.383879948	9.377823143	9.054051241	12189	Scaf8	6.999142315	7.234117004	7.099188093	14489	Tph2	1.659272239	2.686561704	1.982647054
690	A230077H06Rik	4.32850489	3.501499859	4.936700946	2990	Cenpe	5.399521883	5.51912725	5.302272002	5290	Fbxo18	6.250709368	6.263931656	6.68145411	7590	Kctd6	6.898408779	6.88640122	6.680508917	9890	Otub2	7.199433063	7.265121623	7.139763786	12190	Scai	8.903284373	8.622023014	8.748210726	14490	Tpi1	10.32598121	10.28092167	10.13351262
691	A230103J11Rik	1.142901736	3.765393218	4.07131402	2991	Cenpf	6.120873828	5.641041166	5.335865382	5291	Fbxo2	9.935136042	9.806666961	9.51321469	7591	Kctd7	5.290310841	5.456726865	5.37253607	9891	Otud1	6.012873758	6.333810011	6.149933396	12191	Scamp1	10.75642022	10.65001205	10.65417882	14491	Tpk1	4.460632558	3.930669056	3.679870315
692	A2m	3.106752755	4.813826004	4.302581618	2992	Cenph	3.767205789	3.005902507	2.562216854	5292	Fbxo21	8.468341997	8.535698576	8.463853637	7592	Kctd8	5.83978822	6.083506438	6.924297219	9892	Otud3	5.075257473	4.471121575	4.93491552	12192	Scamp2	7.16511477	6.533201349	6.874930651	14492	Tpm1	10.35304187	10.1505376	10.19793421
693	A330009N23Rik	5.289391468	4.957597804	5.440117975	2993	Cenpj	5.125471095	5.232367167	4.562252373	5293	Fbxo22	6.926015206	7.019256008	6.82527123	7593	Kctd9	5.864239603	5.900416295	6.170174013	9893	Otud4	8.046234698	7.841762448	8.13998785	12193	Scamp3	9.083182226	9.158215836	8.982783188	14493	Tpm2	5.214159271	4.667966322	6.026112723
694	A330015K06Rik	0.081347275	1.410840289	3.446128769	2994	Cenpk	5.80833276	5.614279789	5.707604126	5294	Fbxo25	8.556039721	8.517527308	8.367896043	7594	Kdelc1	3.887362194	2.964956269	3.786950183	9894	Otud5	8.851422741	8.638639616	8.834992416	12194	Scamp4	7.649756041	7.759113892	7.631168915	14494	Tpm3	9.685961557	9.87611553	9.570541438
695	A330023F24Rik	6.349102372	5.822180324	6.4226968	2995	Cenpl	3.785855818	4.007172817	4.150544316	5295	Fbxo27	3.792698484	4.295733554	5.192841713	7595	Kdelc2	5.330577778	5.186884018	5.863953181	9895	Otud6b	8.919310627	8.710946936	8.787354934	12195	Scamp5	9.494198494	9.606394952	9.620285396	14495	Tpm4	8.235392863	8.040765833	8.194258453
696	A330032B11Rik	2.634342654	1.881991054	1.569736105	2996	Cenpm	4.882736431	4.936762158	4.624008307	5296	Fbxo28	8.162571435	8.169057592	8.385386697	7596	Kdelr1	9.461809199	9.40993082	9.253744001	9896	Otud7a	6.819762967	6.579264037	6.471569522	12196	Scand1	8.442900016	8.813860373	8.475774085	14496	Tpmt	7.14655393	6.889370651	7.10922127
697	A330033J07Rik	2.867489338	2.449476101	1.981486202	2997	Cenpn	3.381173685	2.432808531	2.566898823	5297	Fbxo3	7.760211885	7.604019085	7.91678342	7597	Kdelr2	7.27079795	7.589230974	7.045564582	9897	Otud7b	8.739221341	8.962327239	9.007969763	12197	Scap	8.063239072	7.972022105	7.89872993	14497	Tpo	0.016590779	1.921622151	0.989762823
698	A330035P11Rik	1.054660891	2.226173793	0.986179141	2998	Cenpo	6.835211867	6.826805201	6.89903982	5298	Fbxo30	5.502272473	5.398144999	5.30229688	7598	Kdelr3	5.574418202	5.794032362	5.887432703	9898	Otulin	6.404343097	6.771942507	6.826027739	12198	Scaper	10.04040751	9.879342558	9.952972137	14498	Tpp1	8.781330023	8.806022132	8.764528614

699	A330041J22Rik	4.659142718	3.995232634	5.027321218	2999	Cenpp	4.071122432	3.284240387	2.976488933	5299	Fbxo31	8.719987063	8.629953586	8.451631115	7599	Kdm1a	7.900840279	7.946598475	8.03540447	9899	Otx1	5.474197885	5.537500621	6.30414559	12199	Scara3	6.874488184	6.743410408	6.968982208	14499	Tpp2	8.552359596	8.751731951	8.546004094
700	A330048O09Rik	3.798052743	4.93721448	4.888207195	3000	Cenpq	5.541408067	5.618586914	5.680564148	5300	Fbxo32	7.982195804	8.462324922	8.507819446	7600	Kdm1b	7.402252491	7.453617818	7.355003594	9900	Otx2	6.409604105	7.535660903	7.346561031	12200	Scara5	2.650043811	3.47558045	0.976562468	14500	Tppp	11.32187041	11.29069627	11.13542629
701	A330049N07Rik	1.071924564	2.200987604	1.984533144	3001	Cenpt	4.776699284	4.418372935	5.230651264	5301	Fbxo33	9.023226863	9.014928386	9.21368293	7601	Kdm2a	8.639394847	8.660557202	8.96311626	9901	Ovca2	7.504430329	7.356140065	6.78658569	12201	Scarb1	5.604056635	5.844113877	5.680700139	14501	Tppp3	8.379841805	8.610772819	8.194105351
702	A330050F15Rik	6.554808633	6.327944569	6.188831418	3002	Cenpu	3.773731178	3.757854185	4.070475154	5302	Fbxo34	8.780466476	8.612590778	8.758173748	7602	Kdm2b	7.158037968	7.492042744	7.431506827	9902	Ovgp1	3.242240926	3.014557347	4.072726064	12202	Scarb2	6.617996705	6.452605063	6.319496248	14502	Tpr	10.96471063	10.79399831	10.97787766
703	A330069E16Rik	5.807352367	5.382733513	5.533906559	3003	Cenpv	7.523632196	7.782988009	7.761656632	5303	Fbxo36	3.066612248	3.033248868	2.301776814	7603	Kdm3a	6.620533135	6.862885094	6.680367275	9903	Ovol1	3.788526545	4.907270074	4.228319291	12203	Scarf1	1.671613119	2.668550883	3.687087619	14503	Tpra1	6.4214894	6.258652802	6.721916751
704	A330070K13Rik	3.06974787	3.026663862	3.68547304	3004	Cenpw	5.7788757	5.535756875	5.911652064	5304	Fbxo38	7.259887919	7.379661389	7.302126492	7604	Kdm3b	5.918668719	6.244998513	6.851545879	9904	Ovol2	5.394579119	4.404862861	5.56743646	12204	Scarf2	3.071548397	2.413210406	2.788174172	14504	Tprgl	9.751210832	9.821048425	9.782515454
705	A330074K22Rik	2.656508157	2.157315828	3.306711294	3005	Cep104	5.632345024	5.728226139	5.624491933	5305	Fbxo39	1.088234543	4.231428366	1.564715153	7605	Kdm4a	7.67252687	7.728090057	7.414344115	9905	Ovol3	7.760066514	7.804015204	7.609126472	12205	Scarna10	1.659256417	2.457655604	2.569782223	14505	Tprkb	9.491919875	9.339366911	9.129580574
706	A330076C08Rik	1.676196361	1.820978048	2.566684792	3006	Cep112	6.403214262	6.421781779	6.694765524	5306	Fbxo4	6.076872276	5.928786714	5.88719635	7606	Kdm4b	7.3479822	7.647599161	7.602281847	9906	Oxa1l	7.48131148	7.352157937	7.708272603	12206	Scarna13	4.923926323	4.665839481	4.737770083	14506	Tprn	5.478442758	6.12942784	6.046629624
707	A330076H08Rik	5.865921716	5.758197582	6.504395378	3007	Cep120	7.531051205	7.546718326	7.380800409	5307	Fbxo41	9.306552332	9.204492654	9.290690329	7607	Kdm4c	5.753975576	5.692263752	6.321270915	9907	Oxct1	11.51462866	11.48893025	11.44095286	12207	Scarna17	3.771554419	3.762345856	3.889671249	14507	Tpsg1	5.981382945	6.119199084	5.371729054
708	A330093E20Rik	2.89122083	2.391686522	3.567437419	3008	Cep126	6.52539956	6.981742041	7.200133964	5308	Fbxo42	6.413907216	5.860198957	6.002798026	7608	Kdm4d	1.100291502	2.864624903	3.445002703	9908	Oxgr1	0.019284035	1.497708643	0.989116326	12208	Scarna2	2.081723041	0.776798485	4.074828272	14508	Tpst1	7.167518321	7.266413442	7.035138879
709	A330102I10Rik	0.045409514	0.862887096	2.988520652	3009	Cep128	5.330270077	5.4220526	5.301365733	5309	Fbxo44	7.981776838	8.114471623	8.204150662	7609	Kdm5a	9.199453629	9.243253086	9.027018523	9909	Oxld1	4.888883545	5.885631971	4.786651774	12209	Scarna3a	1.63279666	0.894652578	0.98729064	14509	Tpst2	6.507090342	6.634121382	6.680775372
710	A430005L14Rik	7.21047088	7.229225648	7.381005291	3010	Cep131	5.745369949	5.299743943	4.561995987	5310	Fbxo45	8.214572705	8.164621554	8.189511784	7610	Kdm5b	8.871978159	8.892749792	9.096414327	9910	Oxnad1	6.63301195	6.482854804	6.638725765	12210	Sccpdh	8.477512445	8.495517205	8.5873255	14510	Tpt1	11.56893904	11.66320543	11.50891178
711	A430033K04Rik	6.229322903	6.232036798	6.045780546	3011	Cep135	5.813632765	6.411685142	6.283942535	5311	Fbxo46	5.838539014	6.383550078	6.129881103	7611	Kdm5c	7.563895183	7.33577876	7.652656604	9911	Oxr1	9.855071316	9.575549907	9.900421764	12211	Scd1	9.152484364	9.454753852	9.623969796	14511	Tpx2	4.931856246	5.10351006	4.503357192
712	A430035B10Rik	4.893630496	5.19437141	5.337963295	3012	Cep152	2.686915919	3.752506802	4.507672017	5312	Fbxo5	3.080631323	3.555869914	2.561906508	7612	Kdm5d	6.680419357	6.092659732	6.549542547	9912	Oxsm	4.724248325	4.472749579	4.98108318	12212	Scd2	11.42439649	11.52205997	11.62789526	14512	Tra2a	7.981853177	8.081088707	8.046045834
713	A430046D13Rik	6.034436523	6.252667029	5.933949111	3013	Cep162	7.463110575	7.370196156	7.169589038	5313	Fbxo6	6.871558027	6.524573195	6.354621695	7613	Kdm6a	6.591094166	7.12694985	7.130216857	9913	Oxsr1	7.059087315	7.212735468	7.464209004	12213	Scd3	6.006354365	5.416710162	5.787864932	14513	Tra2b	8.156309338	8.0823534	7.850246035
714	A430078I02Rik	5.172980375	5.481583109	5.53475558	3014	Cep164	4.843943025	5.544197368	5.472347744	5314	Fbxo7	7.32861651	7.615895544	7.328682161	7614	Kdm6b	5.666630157	6.241715993	5.300272571	9914	Oxt	3.525752323	3.677300229	1.55731359	12214	Scd4	1.626690539	0.910095554	0.989113754	14514	Trabd	6.832752902	6.404845702	6.62402132
715	A430090L17Rik	2.38328137	1.431224557	1.983230408	3015	Cep170	8.151875693	7.885592275	8.389654343	5315	Fbxo8	6.522703421	6.452661103	6.354787243	7615	Kdm7a	8.932489674	9.015333136	8.939948815	9915	Oxtr	5.351873366	4.984152912	4.50374798	12215	Scel	1.674948999	2.432915843	2.790276425	14515	Trabd2b	4.82956377	4.702865837	4.300982689
716	A430105I19Rik	4.832244196	4.89007676	5.025917836	3016	Cep170b	9.785783441	9.597862121	9.704563738	5316	Fbxo9	8.944949144	9.049863992	9.16246705	7616	Kdm8	3.541468276	3.937391442	4.230717572	9916	P2rx3	2.64564893	0.819287145	2.985982526	12216	Scfd1	8.102530063	8.02274552	8.145034835	14516	Tradd	4.718781137	3.71537889	4.06574795
717	A4galt	0.040210971	2.217186425	0.984606761	3017	Cep19	8.763867499	8.794524634	9.010748171	5317	Fbxw10	1.105732277	2.858774551	1.561036716	7617	Kdr	6.05006066	5.870842387	5.264635965	9917	P2rx4	4.674237851	5.12217377	5.228200804	12217	Scfd2	5.887350233	6.072056449	6.209887451	14517	Traf1	1.073722572	1.867475507	2.571051504
718	A530016L24Rik	3.870601635	3.31203766	3.441900846	3018	Cep192	2.90934687	3.406588302	3.146278593	5318	Fbxw11	8.343108283	8.418730451	8.376512305	7618	Kdsr	8.580019274	8.61023754	8.431159181	9918	P2rx5	3.653684387	2.626022906	3.441362316	12218	Scg2	10.57873271	10.67344179	10.8980898	14518	Traf2	3.881286816	4.182496371	3.565025767
719	A530032D15Rik	0.008387076	0.933562442	0.991896942	3019	Cep250	6.347530057	6.408566353	6.319715278	5319	Fbxw15	2.069278247	2.882708921	1.982339477	7619	Keap1	8.969080599	8.949279628	8.910736924	9919	P2rx6	3.070975952	2.646933236	3.152584749	12219	Scg3	10.94634806	11.07810324	11.05947677	14519	Traf3	8.254381562	7.987345973	7.793498539
720	A530046M15Rik	0.076896351	1.846367712	1.981755456	3020	Cep290	9.074945017	8.956136637	9.274715513	5320	Fbxw17	5.162263267	4.972249583	4.622990117	7620	Khdrbs1	8.753061562	8.843828241	8.931379578	9920	P2rx7	6.284642597	6.324543799	6.068146394	12220	Scg5	10.80332765	10.6863424	11.04545743	14520	Traf3ip1	5.861568748	5.76677882	6.190497967
721	A530050N04Rik	0.032549648	1.476222481	1.987798369	3021	Cep295	7.611299621	7.613843746	7.487748346	5321	Fbxw2	8.855191622	8.947140268	8.883924812	7621	Khdrbs2	5.082793744	5.157819392	5.763236011	9921	P2ry1	4.941895249	5.044213477	4.733106015	12221	Scgn	5.112366279	4.151991032	3.884460045	14521	Traf3ip2	3.881387828	2.598010649	3.300536728
722	A530058N18Rik	5.368585941	5.071225858	5.33677554	3022	Cep350	8.654376066	8.595375781	8.748201553	5322	Fbxw4	7.101219359	7.163874666	6.850256452	7622	Khdrbs3	10.33545027	10.39280027	10.2711711	9922	P2ry12	7.091749994	7.036743426	6.979775818	12222	Schip1	2.405548366	2.416172219	3.443422402	14522	Traf3ip3	3.656347064	3.551909667	3.790140944
723	A530072M11Rik	1.638221972	0.880644029	1.987403428	3023	Cep41	5.248690584	5.736969424	4.562343558	5323	Fbxw5	7.166236526	6.801268265	7.088794922	7623	Khk	5.288653392	5.548053217	5.440296294	9923	P2ry13	7.03564078	7.178098274	7.389798345	12223	Scin	0.035171144	2.223025313	1.571450348	14523	Traf4	4.093125555	4.695538803	4.735021056
724	A630001G21Rik	0.02465994	1.909315221	1.573368792	3024	Cep44	3.895120485	4.711901244	4.682358791	5324	Fbxw7	11.8037948	11.75217565	11.81840284	7624	Khnyn	4.658867419	4.427609542	4.149214215	9924	P2ry14	4.167331559	4.002408678	3.677988606	12224	Sclt1	6.543884815	6.891919778	7.347013677	14524	Traf5	2.878558005	3.187858048	3.153342074
725	A630019I02Rik	3.990351462	4.433433249	4.440811813	3025	Cep55	1.652401526	2.198388403	2.794113436	5325	Fbxw8	6.77878437	6.660071707	6.422612856	7625	Khsrp	8.446709712	8.57031677	8.337477179	9925	P2ry2	1.632754222	1.484410736	0.987303408	12225	Scly	5.285007291	4.633240467	5.266532366	14525	Traf6	5.176249673	5.561952315	5.371105674
726	A630020A06	0.040229552	0.871704255	2.795976784	3026	Cep57	7.808337598	7.732127877	7.806565738	5326	Fbxw9	5.502592007	5.194448784	5.108811254	7626	Kidins220	9.754775809	9.673585789	9.853462143	9926	P2ry6	3.387552147	2.646713195	3.443276232	12226	Scmh1	7.473087387	7.759550844	7.631224064	14526	Traf7	6.54053609	6.291192369	6.62390964
727	A630023P12Rik	1.0502707	1.905597679	1.988648432	3027	Cep57l1	4.726900603	4.582593796	5.408307597	5327	Fcer1g	6.507239615	6.796489451	6.471647978	7627	Kif11	4.379831961	2.391684658	3.441343503	9927	P3h1	3.778894806	4.449491001	4.230436682	12227	Scml2	2.399164781	1.81889953	2.566493219	14527	Trafd1	7.447035578	7.474499547	7.594884126
728	A630033H20Rik	2.636601389	2.205978089	0.982610325	3028	Cep63	6.769561403	7.435427242	7.274995945	5328	Fcf1	8.271014504	8.563246165	8.389337476	7628	Kif13a	7.437796953	7.407898007	7.372272158	9928	P3h3	4.726198599	4.97178923	4.734469679	12228	Scml4	4.232827269	4.334260317	2.295993871	14528	Traip	4.674389903	5.334774995	4.93322592
729	A630066F11Rik	2.882490865	3.784584431	2.564632617	3029	Cep68	6.081139156	6.39813457	6.338114462	5329	Fcgbp	2.048171121	1.898350685	1.571913797	7629	Kif13b	5.124290339	5.234678134	5.067375534	9929	P3h4	6.984864574	6.982194673	6.887299366	12229	Scn10a	1.628786468	1.493104135	1.573892988	14529	Trak1	8.807505496	8.552304632	8.507453175
730	A630072M18Rik	5.699485395	6.199576859	5.910401993	3030	Cep70	7.297570895	6.690436825	7.046132603	5330	Fcgr1	3.873667847	3.304487834	1.554218962	7630	Kif14	1.632790428	0.894668505	1.572862718	9930	P4ha1	7.490435307	7.565726342	7.534524426	12230	Scn1a	10.0668515	9.98145563	10.79143751	14530	Trak2	9.450919417	9.359683879	9.258413538
731	A630075F10Rik	1.073681167	1.440970143	2.987319806	3031	Cep72	3.53930304	3.143395358	3.888690839	5331	Fcgr2b	2.656496552	2.431171724	2.983540864	7631	Kif15	3.651604315	3.871408436	3.303796749	9931	P4ha2	5.073098382	4.350970295	3.882954831	12231	Scn1b	10.80170194	10.84765181	10.90034709	14531	Tram1	7.993332198	8.161455226	7.825297866
732	A630089N07Rik	5.723498937	5.181162494	5.438627085	3032	Cep76	4.479485626	4.838098554	5.15120449	5332	Fcgr3	6.381967796	6.213785533	6.045705667	7632	Kif16b	6.145039756	6.217472361	6.407345435	9932	P4ha3	4.777958657	4.0641172	4.788114839	12232	Scn2a1	11.84333719	11.8251198	11.71933601	14532	Tram1l1	7.356939546	7.453270707	7.557670217
733	A730008H23Rik	4.247774547	3.624447305	4.89012253	3033	Cep78	4.895328373	5.227061993	5.023147775	5333	Fcgrt	6.602289615	6.343985388	6.471961202	7633	Kif17	4.473227762	4.596238785	5.069792872	9933	P4hb	7.957925454	8.006611668	8.324473129	12233	Scn2b	9.986136499	10.1609684	10.34637277	14533	Trank1	7.62878255	7.623194059	7.812803552
734	A730017C20Rik	8.384106605	8.238043429	8.324280301	3034	Cep83	8.332755508	8.159265454	8.315471703	5334	Fcho1	6.900914139	6.640379677	7.407150013	7634	Kif18a	2.899230203	3.945769057	3.56583362	9934	P4htm	7.642683488	7.592373342	7.572368671	12234	Scn3a	8.818959607	8.859850132	8.862585068	14534	Trap1	6.964868092	7.248234716	7.346607973
735	A730017L22Rik	3.886344211	4.825193428	3.563630858	3035	Cep83os	6.697163726	6.859409929	7.398753567	5335	Fcho2	8.103145706	8.302568828	8.315545232	7635	Kif19a	2.046567837	1.90188207	0.986730414	9935	Pa2g4	9.623335681	9.543662005	9.549869321	12235	Scn3b	8.244965044	8.200350083	7.916378761	14535	Trappc1	8.82107125	8.502956205	8.283538568
736	A730020E08Rik	4.980152712	4.478900222	5.191977663	3036	Cep85	5.694358994	5.385409489	6.089947891	5336	Fchsd1	8.063588772	8.095428566	8.359667667	7636	Kif1a	11.59074101	11.39334919	11.39096226	9936	Pabpc1	11.11199354	11.28446023	11.27690837	12236	Scn4b	6.31883674	7.279210578	8.046987654	14536	Trappc10	8.374992889	8.284587445	8.56140044
737	A730020M07Rik	1.65934272	1.425200655	2.569766321	3037	Cep85l	7.365394654	7.378211982	7.456002804	5337	Fchsd2	7.572217656	7.45890397	7.659639682	7637	Kif1b	13.08410977	12.97039582	13.06046119	9937	Pabpc4	5.571938636	5.509357898	5.62413408	12237	Scn5a	2.640824225	0.838364804	2.793852727	14537	Trappc11	7.531136025	7.407361663	7.503657842
738	A730046J19Rik	0.047970377	1.878840503	2.572139769	3038	Cep89	5.16685263	5.551291696	4.979502769	5338	Fcrls	6.70896466	7.024743225	6.800597543	7638	Kif1bp	9.260376506	9.251594988	9.147361974	9938	Pabpc4l	3.646971568	2.846214503	3.569186501	12238	Scn7a	4.395876608	4.087209998	4.626379068	14538	Trappc12	7.093147244	7.596749294	7.42317708
739	A730056A06Rik	3.263506195	4.725702913	3.980720653	3039	Cep95	5.439144335	5.032401753	5.065693158	5339	Fdft1	8.383644013	8.277111435	8.072590148	7639	Kif1c	9.049254904	9.258702697	8.985562926	9939	Pabpc5	4.53351507	4.000404298	4.566654217	12239	Scn8a	10.58540566	10.67398781	10.79620885	14539	Trappc13	8.954042184	8.992775653	9.018937416
740	A730090N16Rik	2.070389873	1.84892635	3.308729465	3040	Cep97	7.070110214	7.290525873	7.119420647	5340	Fdps	7.353831504	7.038381949	7.013473072	7640	Kif20a	6.539996322	6.698711479	6.666785737	9940	Pabpc6	1.09288345	4.228106767	2.568182621	12240	Scn9a	4.409672269	5.015217586	4.93481831	14540	Trappc2	4.149534783	3.304524283	3.302859854
741	A830009L08Rik	3.396562915	2.824914716	3.983910819	3041	Cept1	8.612006899	8.5023377	8.758161841	5341	Fdx1	6.753897708	6.93718456	6.774225366	7641	Kif20b	4.162421018	4.09384145	3.437016357	9941	Pabpn1	9.652219646	9.734606212	9.564931986	12241	Scnm1	6.212135339	6.662677351	6.372184792	14541	Trappc2l	8.794744904	8.863467927	8.793780395
742	A830010M20Rik	10.79220312	10.75219907	10.97438	3042	Cercam	3.072692631	3.450579759	2.787857261	5342	Fdx1l	7.847168683	7.551809201	7.572438831	7642	Kif21a	10.87421772	10.7631211	11.07869914	9942	Pacrg	7.122366554	6.990432704	7.150587662	12242	Scnn1a	3.399176269	4.036040695	3.889876186	14542	Trappc3	8.124717365	8.123665083	8.08328418
743	A830011K09Rik	4.471063134	4.48549177	4.789110888	3043	Cerk	8.074469013	7.830748989	8.035329871	5343	Fdxacb1	3.881913068	2.59634414	3.888353827	7643	Kif21b	6.707599831	6.342964494	6.624207533	9943	Pacrgl	3.785710296	4.308000327	3.979829061	12243	Scnn1g	1.043232387	0.910084397	1.574430874	14543	Trappc3l	1.062715895	2.710195376	1.986262967
744	A830018L16Rik	8.480479328	8.094019547	8.40220825	3044	Cerkl	1.082086957	3.813016765	1.982654731	5344	Fdxr	5.036323446	4.827651038	5.596377318	7644	Kif22	5.288631754	5.459982148	4.885545472	9944	Pacs1	7.228045015	7.135368454	7.160316436	12244	Sco1	5.886742193	5.95006883	5.707601812	14544	Trappc4	8.631430024	8.726925988	8.741593135
745	A830019L24Rik	3.062601505	3.041658945	1.562480651	3045	Cers1	8.903289495	8.651340965	8.71809448	5345	Fech	7.702382746	7.884532836	7.806730146	7645	Kif23	1.672589495	3.587793321	2.303122274	9945	Pacs2	8.384559713	8.613775506	8.515325849	12245	Sco2	6.329868855	6.798267366	6.503661737	14545	Trappc5	7.657264198	7.747051346	7.397618152
746	A830052D11Rik	3.068017845	2.147634525	2.982661886	3046	Cers2	8.825599392	9.218803052	8.711171788	5346	Fem1a	7.99244269	7.76825007	7.787324532	7646	Kif24	3.233812401	3.190428671	0.972414385	9946	Pacsin1	11.01940725	11.0697154	11.08065752	12246	Scoc	10.87170293	10.79558255	10.88913656	14546	Trappc6a	5.688712886	5.547045686	5.302160478
747	A830080D01Rik	5.888616113	5.967680963	6.150525163	3047	Cers3	2.05126168	1.468489388	2.30970656	5347	Fem1b	9.352019272	9.311381834	9.465872786	7647	Kif26a	5.720895879	6.08184056	4.931965062	9947	Pacsin2	6.586866574	6.540173839	6.455753951	12247	Scp2	8.70320101	8.993127257	8.895861146	14547	Trappc6b	7.309003287	7.314331989	7.265402506
748	A830082K12Rik	5.571223283	5.539600263	5.813962764	3048	Cers4	8.428390491	8.382196466	8.035079344	5348	Fem1c	5.07738951	5.439059011	4.837746701	7648	Kif26b	5.162399076	5.054715004	4.88715422	9948	Pacsin3	7.932192959	7.993435209	7.868677857	12248	Scp2d1	0.016598358	0.915560254	2.576277843	14548	Trappc8	7.068502424	7.068025992	7.013479565

749	A830082N09Rik	8.204168731	8.037038853	8.213738373	3049	Cers5	7.547082308	7.52610573	7.431276366
750	A930001C03Rik	4.400495591	3.248270169	5.23157888	3050	Cers6	5.036730761	4.679346974	5.654188439
751	A930002C04Rik	2.068136842	1.853992727	3.572865853	3051	Ces1c	2.89201973	0.687911416	2.7856909
752	A930003A15Rik	0.088208605	1.834563957	3.987782339	3052	Ces1d	0.052664322	2.473304086	0.9821829
753	A930004D18Rik	4.659917281	4.900306962	3.978087106	3053	Ces2a	1.630805991	1.48868184	0.987888085
754	A930005H10Rik	6.949366394	6.46144689	6.911019299	3054	Ces2e	0.021978644	0.904816313	0.988489431
755	A930006K02Rik	3.664438574	3.646170764	4.069576926	3055	Ces5a	2.857635965	2.203314618	1.568529797
756	A930009A15Rik	2.05696392	1.878918418	1.56932632	3056	Cetn1	1.688162497	5.116703587	3.152352355
757	A930011G23Rik	0.021991714	0.90479095	1.573888951	3057	Cetn2	9.491587058	9.449595424	9.299675248
758	A930012L18Rik	3.651155424	3.443400171	3.791390838	3058	Cetn3	11.30631333	11.17429101	11.25902213
759	A930013F10Rik	5.863077804	5.468645507	6.407526221	3059	Cetn4	6.32862617	6.377719604	6.406420072
760	A930015D03Rik	4.071200775	2.979841446	4.152522917	3060	Cfap126	5.399493985	6.147006346	4.621232041
761	A930016O22Rik	0.037656995	1.89163807	2.989334111	3061	Cfap20	7.752164426	7.572089494	7.319381852
762	A930017M01Rik	4.410450012	4.276457709	5.33952495	3062	Cfap36	9.763669545	9.766887538	9.864166625
763	A930024E05Rik	4.832677449	5.13775766	4.502747086	3063	Cfap43	5.541042769	5.911473465	5.336512357
764	AA387883	3.393917765	3.310341482	3.791087597	3064	Cfap44	4.332685932	5.418435473	4.565017273
765	AA388235	5.169650317	5.876238786	4.932780027	3065	Cfap45	5.199097264	5.386888578	4.565058454
766	AA414768	5.749269717	5.291491213	4.931914793	3066	Cfap46	2.417892203	4.041861136	3.441252121
767	AA415398	5.529069633	4.639019062	4.980681683	3067	Cfap53	3.783341463	5.16238994	3.563156978
768	AA465934	5.467916096	4.917838353	5.338277653	3068	Cfap57	3.074320986	3.780040119	2.299234296
769	AA467197	2.0796802	3.041733362	3.154640941	3069	Cfap58	3.227415192	2.443503276	0.976120884
770	AA792892	0.005608334	0.940318714	0.992698372	3070	Cfap69	4.887300838	5.242096138	5.267003296
771	AA986860	3.66074422	3.147239435	3.681670569	3071	Cfap74	2.655656962	2.863327471	2.566927464
772	Aaas	6.229839778	6.731108166	6.208856876	3072	Cfap97	8.220355385	8.199151348	8.503760589
773	Aacs	6.187908807	6.4300422	5.679099952	3073	Cfb	3.883912669	2.589982112	3.680385422
774	Aadat	1.639947329	2.220097101	0.985118768	3074	Cfdp1	10.09314251	10.01138114	10.02299986
775	Aaed1	2.881301276	1.363901801	3.685327819	3075	Cfh	6.508290961	6.69653732	6.579718177
776	Aagab	8.46813999	8.296829546	8.333111348	3076	Cfhr1	0.021976578	0.904820322	2.312002617
777	Aak1	9.736881844	9.684915785	9.647084341	3077	Cfl1	11.31355921	11.33324935	11.1163112
778	Aamdc	8.134852038	7.967465	7.874735076	3078	Cfl2	9.292839129	9.394008045	9.487782554
779	Aamp	10.63895592	10.58681888	10.4926979	3079	Cflar	6.913278569	7.319871455	6.957396396
780	Aar2	6.603967967	6.274466453	6.735212793	3080	Cfp	3.08029819	3.77016925	2.297245437
781	Aard	3.261618587	4.098587271	4.068959276	3081	Cftr	3.066604636	2.657377443	3.306252376
782	Aars	8.481825258	8.572420045	8.666547016	3082	Cga	0.088577419	2.671672108	1.980354112
783	Aars2	5.544236617	6.110941171	6.130599689	3083	Cggbp1	9.330891206	9.21232062	9.530823848
784	Aarsd1	8.135377152	8.042463132	8.072784783	3084	Cgn	3.686955056	4.534679298	4.735321893
785	Aasdh	4.40205856	4.853523902	3.67659316	3085	Cgnl1	6.808665963	7.201113326	6.564341471
786	Aasdhppt	7.772485061	7.17333399	7.701362287	3086	Cgref1	6.760886415	6.122225116	5.979428774
787	Aass	4.466305233	4.434166437	4.440901625	3087	Cgrrf1	6.269822613	6.265026902	6.265510138
788	Aatf	5.081395874	5.400274034	5.407281346	3088	Ch25h	1.660656315	1.422215819	1.982335402
789	Aatk	10.65509236	10.71533307	10.60020747	3089	Chac1	4.08145943	4.86203879	4.68282949
790	Abat	9.255625861	8.986118813	9.495774779	3090	Chac2	4.613262735	4.731784192	5.302657038
791	Abca1	7.366738714	7.375647106	7.715035639	3091	Chadl	5.471677208	5.89534862	5.472127453
792	Abca2	9.515287394	9.663115441	9.629268807	3092	Chaf1a	4.169623983	4.659163906	4.440277844
793	Abca3	6.419848246	6.498634102	6.801202583	3093	Champ1	7.093919472	7.430822663	7.673910633
794	Abca4	2.663017405	4.212730903	1.559127887	3094	Chat	1.749938893	4.905877039	5.112982712
795	Abca5	7.687778538	7.564586992	7.819394125	3095	Chchd1	9.014151315	8.881733889	9.192007181
796	Abca6	3.652892237	3.309243545	3.150691541	3096	Chchd10	11.18523499	11.32115744	11.28433306
797	Abca7	3.992457502	3.99816727	4.62555464	3097	Chchd2	13.35131189	13.22348919	13.10603062
798	Abca8a	6.343006017	6.304663211	5.533082858	3098	Chchd3	8.481143379	8.449184821	8.414624414

5349	Fen1	5.5073556	5.1477178	5.471576414	7649	Kif27	3.98204729	4.100073473	4.304600693
5350	Fer	7.688321081	7.756608206	7.806589055	7650	Kif2a	9.502544196	9.572944566	9.483821599
5351	Fer1l5	2.642767788	2.461810605	2.306326089	7651	Kif2b	1.045672168	0.904800435	2.798213038
5352	Fermt2	8.635024371	8.677531035	8.781029565	7652	Kif2c	3.070473393	3.683940074	2.788471768
5353	Fermt3	5.693082439	5.566459866	5.594889152	7653	Kif3a	13.07741035	12.93823885	12.8369777
5354	Fes	3.869248037	3.172644236	3.442289553	7654	Kif3b	9.968923313	9.787835329	9.731529736
5355	Fez1	7.80878762	7.848882145	8.008064021	7655	Kif3c	8.960150456	8.933076985	9.005152539
5356	Fez2	7.515528588	7.746673533	7.447403435	7656	Kif4	2.659511759	3.031922064	2.789364881
5357	Fezf2	8.76555029	8.369365261	8.406304446	7657	Kif5a	14.11426947	14.14498625	13.99277498
5358	Fga	1.097297098	0.787539745	2.567536755	7658	Kif5b	12.21341444	12.14093731	12.32566821
5359	Fgb	5.873678844	4.40391337	7.21890439	7659	Kif5c	11.98522134	11.94595367	12.05546434
5360	Fgd1	8.514343242	8.238251031	8.310915159	7660	Kif6	3.769362906	3.302394401	3.890201671
5361	Fgd2	3.892457	4.161470408	4.682897919	7661	Kif7	3.64491216	2.651449685	2.788855104
5362	Fgd3	4.596420165	4.551459352	4.566695385	7662	Kif9	4.884937801	4.842002764	4.838491852
5363	Fgd4	8.396242794	8.047649199	7.700701745	7663	Kifap3	10.91421057	10.81717055	10.963708
5364	Fgd5	3.982310485	3.409064123	3.787650708	7664	Kifc2	9.711143897	9.568059182	9.382986411
5365	Fgd6	5.53659934	5.117198129	5.265214899	7665	Kifc3	7.882037882	7.9192541	8.078109834
5366	Fgf1	8.058865627	8.325898716	8.44773555	7666	Kifc5b	2.382169844	1.434439454	2.306603966
5367	Fgf10	5.80404292	5.675851883	5.912128778	7667	Kin	7.189600311	7.272627155	7.001875557
5368	Fgf11	6.763269974	6.278577043	6.406310224	7668	Kirrel	2.869935444	0.797520173	2.791422679
5369	Fgf12	9.547865328	9.16780803	9.634733379	7669	Kirrel2	2.652557874	0.790733049	2.567731365
5370	Fgf13	9.669094329	9.70246337	9.620252457	7670	Kirrel3	5.754296753	5.52957796	6.285031121
5371	Fgf14	7.572200299	7.488290444	7.794131615	7671	Kiss1	1.056712435	2.717698576	1.571466792
5372	Fgf16	3.070910955	3.453837989	1.97653872	7672	Kiss1r	4.977052426	4.074239784	4.839982887
5373	Fgf18	4.602489474	4.702162624	4.371624614	7673	Kit	7.87127703	7.280738235	6.74712313
5374	Fgf5	3.5498036	4.375002502	3.88632853	7674	Kitl	7.701655154	7.154800888	7.934722407
5375	Fgf8	0.013867802	2.251278153	0.990440272	7675	Kiz	6.926264535	7.13522816	6.898739308
5376	Fgf9	7.702614334	7.50089812	7.80029549	7676	Kl	7.024654897	8.086053329	6.021513349
5377	Fgfbp3	5.69187685	5.390512584	5.406069278	7677	Klc1	11.51985529	11.56028218	11.48629262
5378	Fgfr1	7.887987324	8.143807648	7.579468497	7678	Klc2	8.836065149	9.056967136	9.194382721
5379	Fgfr1op	6.058037263	5.711363951	6.170086052	7679	Klc3	3.545499419	3.526476156	4.304206504
5380	Fgfr1op2	9.419962444	9.173020256	9.423037988	7680	Klc4	6.752092321	6.92993242	6.735049495
5381	Fgfr2	7.78709495	7.928252484	7.714795026	7681	Klf10	6.681887866	6.849273856	6.77426707
5382	Fgfr3	8.397962496	8.153403775	8.333073413	7682	Klf11	3.77586295	2.982984188	3.565155929
5383	Fgfrl1	6.693988826	6.404576297	6.863310321	7683	Klf12	8.214293813	8.061833935	8.306676875
5384	Fgg	3.545284895	2.06110386	4.506683949	7684	Klf13	10.53161562	10.55326633	10.28715975
5385	Fggy	4.268056087	5.491657576	5.149925225	7685	Klf15	6.48603016	6.065092992	5.650856317
5386	Fgl1	3.881305872	2.07957224	3.300561358	7686	Klf16	7.723835742	7.793846799	7.631046059
5387	Fgl2	3.409311143	3.641734488	4.739128649	7687	Klf2	6.570305566	5.679250291	5.732934618
5388	Fh1	8.544411854	8.67090984	8.634616694	7688	Klf3	8.564422776	8.602863074	8.666558236
5389	Fhad1	6.231723408	6.957226845	6.337482891	7689	Klf4	6.209026668	5.168015506	5.861773521
5390	Fhdc1	4.176877113	4.798137099	5.375214858	7690	Klf5	7.389030634	6.96937352	6.774280277
5391	Fhit	4.529334114	4.376879176	4.150899682	7691	Klf6	8.938217562	8.960081888	9.135009472
5392	Fhl1	7.41989202	7.423807073	7.519425941	7692	Klf7	7.765885428	7.912992505	7.694415039
5393	Fhl2	6.849484369	7.20824201	7.067711334	7693	Klf8	4.826073606	4.227667714	4.566184201
5394	Fhl4	1.679058752	4.86334855	1.560817194	7694	Klf9	11.90396188	11.86978936	11.97398338
5395	Fhl5	1.070160742	3.612897496	1.568561077	7695	Klhdc1	3.883515539	3.844537477	3.887953425
5396	Fhod1	2.388323329	3.479746702	1.981770128	7696	Klhdc10	9.242391562	9.105029697	9.075417272
5397	Fhod3	6.328987463	6.01976981	7.100558109	7697	Klhdc2	9.489790381	9.396197506	9.418781235
5398	Fibcd1	8.047305513	7.176513569	7.319937017	7698	Klhdc3	8.102773355	8.27416308	8.29310746

9949	Padi1	0.008386612	0.933563535	0.991897072	12249	Scpep1	7.198505285	7.170206402	6.957043154
9950	Padi2	7.498308604	7.660070676	7.255738773	12250	Scrg1	8.579383856	8.325773982	8.203878628
9951	Paf1	7.41209909	7.722913771	7.659690536	12251	Scrib	5.884766243	5.722317721	5.534161393
9952	Pafah1b1	11.39574068	11.34502157	11.46933381	12252	Scrn1	7.367037838	7.685058529	7.741839893
9953	Pafah1b2	9.876667177	9.820540908	9.95870897	12253	Scrn2	2.90188803	4.392339816	3.788771957
9954	Pafah1b3	6.507100784	6.888489918	6.594841367	12254	Scrn3	6.939381076	7.105569036	7.440127589
9955	Pafah2	4.840436793	5.160778238	5.109199667	12255	Scrt1	8.271102042	8.531839215	8.725046798
9956	Pag1	5.889643091	6.181218236	5.957057742	12256	Scrt2	3.910587073	4.78637688	4.622368684
9957	Pagr1a	7.680358681	7.745631143	7.89318629	12257	Scube1	5.693150888	5.774622853	6.304209865
9958	Pah	0.027291824	0.894744743	1.572870397	12258	Scube2	2.882490264	2.84622486	2.788168973
9959	Paics	10.71092186	10.87270643	10.77520377	12259	Scx	3.252474247	3.425620921	3.789452617
9960	Paip1	9.133557964	9.396551371	9.286028064	12260	Scyl1	7.759961828	7.780703996	8.030193683
9961	Paip2	10.41041211	10.52882887	10.3808438	12261	Scyl2	5.367827778	4.949878623	5.735710615
9962	Paip2b	7.229993919	7.159348785	7.293053791	12262	Scyl3	5.722632137	5.564859632	5.761903232
9963	Pak1	10.9716909	11.04779767	11.23915817	12263	Sdad1	7.463428814	7.41623181	7.169513087
9964	Pak1ip1	8.887163309	8.544533585	8.605659689	12264	Sdc1	2.654968639	2.665265641	2.302865265
9965	Pak2	8.926092306	9.020628867	8.999614494	12265	Sdc2	7.828508772	7.656670268	7.800186004
9966	Pak3	10.73866282	10.48774943	10.62474405	12266	Sdc3	7.695898389	7.931791734	7.761281886
9967	Pak4	5.908360327	5.750471506	5.70850672	12267	Sdc4	8.721473044	8.856928877	8.877781294
9968	Pak6	6.504765309	6.362418104	6.488235734	12268	Sdcbp	10.37049336	10.29006162	10.21477974
9969	Pak7	8.173936438	8.550266875	8.605952657	12269	Sdcbp2	2.653340474	2.66863336	2.567528437
9970	Pakap	5.164138799	4.787167125	4.886698616	12270	Sdccag3	6.664545668	6.698238132	6.721818484
9971	Palb2	2.060932742	0.84632624	1.984503463	12271	Sdccag8	8.047140904	8.277021748	8.159825088
9972	Pald1	3.684680673	4.479587458	4.788433378	12272	Sde2	7.938574348	7.914354572	7.963000164
9973	Palld	5.890838613	6.232569425	6.189631006	12273	Sdf2	8.650300276	8.742488767	8.641784312
9974	Palm	8.950449772	9.041309225	9.027274907	12274	Sdf2l1	6.64911922	6.60767801	6.148914708
9975	Palm2	9.275092165	9.243254927	9.213624722	12275	Sdf4	9.886415538	9.947624169	10.02985711
9976	Palm3	4.461530847	4.824300574	3.437219987	12276	Sdha	10.47711581	10.31839545	10.37336099
9977	Palmd	7.570229985	7.520807736	6.837173175	12277	Sdhaf1	6.83293272	6.819608114	6.503390543
9978	Pam	9.504873183	9.412764379	9.74160473	12278	Sdhaf2	7.912146304	7.745463084	7.754880886
9979	Pam16	8.713532039	8.891459927	8.645273553	12279	Sdhaf3	2.414693622	2.397251318	2.5628083
9980	Pamr1	5.078598885	4.633610392	5.567163414	12280	Sdhaf4	8.518949144	8.493040415	8.443539252
9981	Pan2	4.88530439	4.64176906	4.935011483	12281	Sdhb	10.92601299	10.86917541	10.81365357
9982	Pan3	7.327197016	7.332162628	7.406694026	12282	Sdhc	8.907188078	8.972447849	9.018959118
9983	Panct2	3.97266503	2.999676296	3.790206569	12283	Sdhd	8.013823983	8.670693034	8.704521889
9984	Pank1	8.415502451	8.301343233	8.350719101	12284	Sdk1	2.416824168	3.668872024	3.683498701
9985	Pank2	7.711134808	7.870581177	8.135028033	12285	Sdk2	1.056774123	0.880542894	1.987394637
9986	Pank3	9.097437317	9.056681053	9.1348092	12286	Sdpr	7.489604205	7.474921997	7.503652335
9987	Pank4	3.816420008	5.454202834	5.912442949	12287	Sdr39u1	7.651320685	7.89391335	7.980194645
9988	Pantr1	9.930609142	9.837823679	9.8144464	12288	Sdr42e1	5.07165304	4.066984514	4.300769141
9989	Pantr2	3.536114803	3.656817427	3.301646342	12289	Sds	4.335023252	4.843578857	5.506670565
9990	Panx1	5.807272864	5.863852046	5.623513842	12290	Sdsl	3.66422537	3.646609411	3.788243326
9991	Panx2	7.657736509	7.537375867	7.714835616	12291	Sebox	3.775396435	4.455429123	3.43909853
9992	Paox	5.125816443	4.678514082	5.50474222	12292	Sec1	2.058285815	1.875984833	0.982620901
9993	Papd4	2.676683242	4.195454766	2.978997549	12293	Sec11a	7.710347013	7.756370682	7.825524781
9994	Papd5	8.119342566	7.986670032	8.051404775	12294	Sec11c	9.398046728	9.415189411	9.208687959
9995	Papd7	6.45918532	6.871964089	6.774282492	12295	Sec13	7.402770001	7.58771916	7.17920925
9996	Papola	9.292156765	9.10902908	9.132249495	12296	Sec14l1	10.37874753	10.51734931	10.56025897
9997	Papolb	2.664968176	4.539814279	2.300006441	12297	Sec14l2	7.950397756	7.947308527	7.868754104
9998	Papolg	6.42315096	6.695812972	6.862998045	12298	Sec14l3	5.516376388	4.16804594	3.298257924

14549	Trappc9	7.738412443	7.714764801	7.838185575
14550	Trdmt1	2.901880813	4.184392239	4.231223879
14551	Trdn	1.651073049	2.470644487	2.307451128
14552	Treh	1.657910542	3.060785917	0.979477795
14553	Trem2	5.541040519	5.619304822	5.735282982
14554	Treml1	1.628786468	1.493104135	1.573892988
14555	Treml2	2.634342062	1.881992642	1.985888465
14556	Trerf1	5.401619608	5.705606111	5.624829777
14557	Trex1	5.567581919	5.602782836	4.785375303
14558	Trf	10.38804121	10.70902011	10.50154596
14559	Trh	3.747652583	1.851490743	2.305525293
14560	Trhde	6.437135396	6.439611877	6.503950235
14561	Trhr	6.287397917	4.063712956	3.782846634
14562	Trhr2	0.008382678	0.933572806	0.991898172
14563	Triap1	7.79452096	7.707819798	7.831851592
14564	Trib1	7.200435651	7.157403441	7.337549038
14565	Trib2	8.519289168	8.332497429	8.6666243
14566	Trib3	0.074434839	2.181909534	3.446642333
14567	Tril	7.540953483	7.543420166	8.145447033
14568	Trim11	6.188901878	6.333085344	6.50442307
14569	Trim12a	3.538971566	3.419416087	3.888765402
14570	Trim12c	4.832766489	5.01859626	5.2676805
14571	Trim13	6.209215867	6.024607891	5.979457984
14572	Trim14	2.671944533	3.96015893	2.786373271
14573	Trim16	2.062137235	2.198355347	1.567790418
14574	Trim17	1.657985351	3.060688797	1.982936924
14575	Trim2	11.51152613	11.55127432	11.32259313
14576	Trim21	3.390571242	3.317431506	2.563776973
14577	Trim23	6.754502423	6.967770326	7.035487495
14578	Trim24	7.7869482	7.578841319	7.422631924
14579	Trim25	5.079157536	5.048904977	5.150525203
14580	Trim26	6.383119092	6.001584983	6.472360722
14581	Trim27	6.490230972	6.552733573	6.580049935
14582	Trim28	9.130078155	8.974682836	8.865590837
14583	Trim29	1.08049485	1.856682255	2.986421002
14584	Trim3	6.329895815	6.687576538	6.580193186
14585	Trim30a	5.42740169	5.016650412	4.502454476
14586	Trim30d	4.078990294	4.238758428	4.441397
14587	Trim32	9.863505776	9.858896701	9.926927591
14588	Trim33	8.544000901	8.513742651	8.697808654
14589	Trim34a	3.38364036	1.814420017	3.306224362
14590	Trim35	9.580351437	9.683773594	9.439364886
14591	Trim36	4.889755337	4.922792425	5.505431211
14592	Trim37	11.22211312	11.24390582	11.43634951
14593	Trim39	5.807369122	5.951957986	5.707843874
14594	Trim41	7.854346505	7.862144773	7.60924762
14595	Trim43a	1.054643554	0.885229632	0.986183604
14596	Trim43b	1.058761783	3.084288524	0.985122026
14597	Trim43c	1.094494181	0.79423959	3.687383396
14598	Trim44	9.639033877	9.744965456	9.675216203

799	Abca8b	5.079122535	4.922885421	5.373747743	3099	Chchd4	8.239217745	7.847827641	7.951497371	5399	Fibin	4.327049474	4.29877612	4.736955851	7699	Klhdc4	5.836995632	6.164559028	5.93411455 9999		Pappa	3.983841005	4.246090387	2.97502733	12299	Sec14l4	1.664567477	1.413288628	1.564625605	14599	Trim45	3.527555786	3.443679253	2.980473287
800	Abca9	5.325983173	4.678922159	4.621403554	3100	Chchd5	5.985605871	6.451178357	5.862691445	5400	Fibp	7.612095647	7.799561252	7.601985131	7700	Klhdc7a	4.601277233	4.851241319	4.56547510210000		Pappa2	3.751342559	3.047599797	1.563696573	12300	Sec16a	6.424320252	7.227747223	7.013801952	14600	Trim46	6.67986459	6.806962194	6.264481824
801	Abcb10	7.157098157	7.141905968	7.199297241	3101	Chchd6	9.039958155	8.904340653	8.968768135	5401	Ficd	5.508479189	5.866449763	5.564746229	7701	Klhdc8a	5.297838026	5.861295577	6.28514096510001		Papss1	9.404629792	9.35551447	9.451710634	12301	Sec22a	6.766642868	6.562843937	7.068173618	14601	Trim47	3.548262077	4.09188187	3.29835786
802	Abcb1a	7.338671122	7.270205072	7.721863358	3102	Chchd7	7.887342522	7.563408139	7.974584377	5402	Fig4	6.170062965	6.393318348	6.169313468	7702	Klhdc8b	6.721110184	6.857614328	6.2274671110002		Papss2	6.22775021	5.923084996	6.266109626	12302	Sec22b	7.780228878	7.592114082	7.838228618	14602	Trim56	5.6629796	5.149471954	5.471785294
803	Abcb1b	4.600365768	4.654015825	4.839847682	3103	Chd1	7.888234115	7.748743003	8.256452767	5403	Figf	3.506328827	2.692418864	0.980319165	7703	Klhdc9	5.333602009	6.377623507	5.53409239510003		Paqr3	3.39453773	1.774164221	3.56763541	12303	Sec22c	7.248992515	6.787764466	7.046057742	14603	Trim59	7.20179673	7.593318232	7.875270612
804	Abcb4	5.807536927	5.840397578	5.263625158	3104	Chd1l	5.331102469	5.726468223	5.439188717	5404	Fign	5.809105173	5.790322312	5.707412147	7704	Klhl1	4.686958166	4.303437285	6.59653110610004		Paqr4	8.643233681	8.806652269	8.787345979	12304	Sec23a	9.217229803	9.09762692	9.164907511	14604	Trim62	6.052694618	5.795614549	5.503003405
805	Abcb6	5.121769432	4.128276089	5.303356417	3105	Chd2	9.130097798	8.964308296	9.142458837	5405	Fignl1	1.078821141	3.062523201	0.979906969	7705	Klhl10	0.076734705	3.479772258	1.56499738110005		Paqr5	4.728568495	5.839088916	4.501435704	12305	Sec23b	7.088831903	6.949757583	6.549383629	14605	Trim65	0.052847917	2.473127823	3.158143669
806	Abcb7	7.135537095	6.810619284	7.045977645	3106	Chd3	12.23941981	11.9907097	11.81995512	5406	Fignl2	3.242788149	2.401357051	2.980573108	7706	Klhl11	6.86194445	6.937022581	6.48690744110006		Paqr6	4.329121611	4.424898391	4.372341152	12306	Sec23ip	7.9932125	7.802230315	7.996854812	14606	Trim66	5.405187287	5.32200617	5.788019341
807	Abcb8	6.64835281	6.609165584	6.519332194	3107	Chd3os	10.24435786	10.06235268	10.56311618	5407	Filip1	5.750487611	6.018917077	5.735426394	7707	Klhl12	7.356344436	7.476602261	7.19896375210007		Paqr7	8.035447502	7.912254614	8.297509641	12307	Sec24a	5.727496513	5.988300326	5.734148336	14607	Trim67	4.246665084	3.91991238	4.303084701
808	Abcb9	6.973887083	7.041338427	7.024840413	3108	Chd4	10.14933594	10.19108237	10.31430819	5408	Filip1l	5.54153236	5.671299037	5.30062238	7708	Klhl13	8.628011351	8.533174628	9.13502588810008		Paqr8	8.691876743	8.494275068	8.841112637	12308	Sec24b	5.782916654	5.689681082	6.13033838	14608	Trim68	4.066088636	3.428185311	3.566330476
809	Abcc1	6.034426731	6.400944331	6.189967067	3109	Chd5	9.794426072	9.616033708	9.801853512	5409	Fip1l1	8.356443067	8.301390919	8.306424307	7709	Klhl14	3.079613645	2.828197975	3.68343809110009		Paqr9	6.059984794	6.229915807	6.265457882	12309	Sec24c	7.148009053	7.540829716	7.274502666	14609	Trim7	0.037742369	1.468482332	1.987001929
810	Abcc10	4.612346023	5.270505384	5.625887321	3110	Chd6	9.393608238	9.411240008	9.664820457	5410	Firre	4.941530365	4.680701977	5.625669627	7710	Klhl15	2.100359238	2.398566826	3.30380072210010		Pard3	8.040078401	7.876125137	7.928336267	12310	Sec24d	5.396643951	5.271646514	4.369206566	14610	Trim71	2.068156749	2.186325031	1.982630745
811	Abcc12	1.628797906	1.913250977	1.573890046	3111	Chd7	7.531642688	7.7042098	7.363455207	5411	Fis1	9.871654599	9.922913602	9.638121602	7711	Klhl17	6.267911671	6.198534544	6.10941989910011		Pard3b	6.270098266	6.426538681	5.733278805	12311	Sec31a	8.599966086	8.727802382	8.631118053	14611	Trim72	1.043228749	1.497686787	0.989113365
812	Abcc3	1.656533735	1.431561558	0.979919612	3112	Chd8	8.757097805	9.062348764	9.177339174	5412	Fitm2	4.467609053	4.231685717	3.677696187	7712	Klhl18	6.778837232	6.523749022	6.74862432110012		Pard6a	8.766045998	8.395714323	8.507493034	12312	Sec61a1	8.769826311	8.784726304	8.393474448	14612	Trim8	8.604095075	8.693305988	8.652456414
813	Abcc4	4.476172567	5.138591874	4.735238611	3113	Chd9	9.119580176	9.031991911	9.32437285	5413	Fiz1	5.804277536	5.389120789	5.863830568	7713	Klhl2	9.738915206	9.99057726	9.72309598810013		Pard6b	3.88800011	3.926334826	4.068281707	12313	Sec61a2	7.056677661	6.81340493	7.067749531	14613	Trim80	0.021952694	2.239682214	1.573896679
814	Abcc5	8.370604805	8.292530154	8.587504131	3114	Chdh	6.365866895	6.702403509	6.439630003	5414	Fjx1	7.290443014	7.21977237	7.397913485	7714	Klhl20	6.422115722	6.324455138	7.04707164410014		Pard6g	5.698663661	6.146142966	6.373499973	12314	Sec61b	7.730880017	7.86293413	7.631072609	14614	Trim9	10.05484374	9.988919685	9.922485821
815	Abcc8	5.780118815	5.908738827	6.025349554	3115	Chek1	3.077164794	2.632063192	3.151070105	5415	Fkbp10	2.701812764	4.497373931	4.228753009	7715	Klhl21	6.403460052	6.844103198	6.38910288910015		Parg	7.57226504	7.509799421	7.652563234	12315	Sec61g	9.279740569	9.164665025	9.024346274	14615	Trio	8.576524309	8.462586119	8.77778026
816	Abcc9	3.679396019	4.227044729	4.936806642	3116	Chek2	5.028588636	5.248157345	3.558665206	5416	Fkbp11	1.671640888	2.165001677	3.154797446	7716	Klhl22	7.924925663	7.856060915	7.47148127310016		Park2	4.071245796	3.283918295	3.564827059	12316	Sec62	11.81737905	11.72384556	11.66296498	14616	Triobp	5.603102961	5.845947563	5.405782227
817	Abcd1	2.648260015	1.844178509	2.568844922	3117	Cherp	7.687814615	7.862151765	7.787494188	5417	Fkbp14	6.099630986	5.883334645	5.651785667	7717	Klhl23	8.245169474	8.199966291	8.33770174410017		Park7	9.400420562	9.519159911	9.517214366	12317	Sec63	9.014308905	9.010130991	9.007873262	14617	Trip10	4.783474302	4.635001194	5.068034343
818	Abcd2	7.975973998	7.99677771	8.414935355	3118	Chfr	6.986537217	6.667074175	6.799994289	5418	Fkbp15	6.054861889	6.130544026	5.335781384	7718	Klhl24	8.485914617	8.46298796	8.65603404710018		Parl	7.642998228	7.664244229	7.838184763	12318	Secisbp2	6.455939923	6.943904901	6.624170095	14618	Trip11	8.996334058	8.887315685	9.021686174
819	Abcd3	9.051976101	8.953926318	8.979970037	3119	Chga	9.678663922	9.537709501	9.871833613	5419	Fkbp1a	12.54741109	12.5488369	12.35392306	7719	Klhl25	4.470238463	3.816437253	4.14901540310019		Parm1	7.89885234	7.302163183	7.447404476	12319	Secisbp2l	10.27897981	10.37627739	10.29740927	14619	Trip12	10.05060793	9.902290696	10.12085197
820	Abcd4	4.162833981	4.441945006	4.068267923	3120	Chgb	11.83463756	11.95681859	11.5592661	5420	Fkbp1b	7.375128846	7.490410647	7.519386189	7720	Klhl26	7.625913013	7.368987958	7.36365221910020		Parn	6.682193124	6.91536276	7.08894825	12320	Seh1l	6.78222418	7.252149585	6.886804011	14620	Trip13	2.079765919	3.339890647	2.56730407
821	Abce1	8.249439717	7.84178734	7.95719094	3121	Chia1	3.530269198	3.668375864	3.150471061	5421	Fkbp2	8.424812869	8.430408064	8.414614527	7721	Klhl28	7.06765792	6.904342023	7.04641571610021		Parp1	8.450480411	8.280942662	8.159791878	12321	Sel1l	9.768843479	9.792037535	9.853454134	14621	Trip4	7.849274757	8.043224017	8.062262307
822	Abcf1	9.034048385	8.829583253	8.7937702	3122	Chic1	9.060480086	8.870700504	9.142480012	5422	Fkbp3	10.57443631	10.48418812	10.67087325	7722	Klhl29	6.405575034	6.816917791	6.83824537510022		Parp10	1.65658945	2.191109439	2.570274264	12322	Sel1l3	7.409903412	7.273612727	7.311115865	14622	Trip6	5.328228637	5.544807953	5.472424459
823	Abcf2	7.981047065	8.023805519	8.051621868	3123	Chic2	6.149036267	6.66391536	6.535025976	5423	Fkbp4	9.713558716	9.636861416	9.618380716	7723	Klhl31	0.02731583	1.484358533	2.31118325210023		Parp11	6.790482521	6.244844063	6.609976174	12323	Selenbp1	5.835099008	5.558990001	5.8380578	14623	Triqk	6.080910564	6.04577491	6.129788842
824	Abcf3	6.961566514	6.505327315	6.454958756	3124	Chid1	7.806132548	7.475102186	7.319666606	5424	Fkbp5	7.427259561	7.045814378	6.679465874	7724	Klhl32	6.384526116	6.311683577	6.53490900610024		Parp12	4.596213938	4.302315251	4.228226598	12324	Selenbp2	4.070223256	3.142455555	3.565125075	14624	Trit1	5.720816285	5.222767357	5.471853384
825	Abcg1	7.787527373	7.798676945	7.844340117	3125	Chil1	5.859458729	5.746494271	5.735065243	5425	Fkbp6	0.021987115	2.239635621	0.988487488	7725	Klhl34	1.649467485	1.873150011	1.9848582910025		Parp14	4.717443197	3.908153851	3.784462485	12325	Selk	10.46427418	10.44510773	10.50942473	14625	Trmt1	6.541234507	6.90802885	6.761558401
826	Abcg2	6.82165682	6.756260998	6.721319181	3126	Chil3	0.008381654	1.518469423	0.991898458	5426	Fkbp7	5.127410359	5.545814751	5.066595563	7726	Klhl35	2.063468758	1.864444661	1.56739333210026		Parp16	3.245350894	3.439422982	3.150672294	12326	Selm	9.063726271	9.310288736	9.1092544	14626	Trmt10a	6.628720334	6.086839389	6.129998734
827	Abcg3	1.657873311	3.060834254	1.982962165	3127	Chit1	0.035126673	1.472344196	3.575666607	5427	Fkbp8	9.782662088	9.829627168	9.714684752	7727	Klhl36	3.980284364	4.180107382	4.06957276310027		Parp2	6.847870481	6.780843098	7.013960682	12327	Selo	5.632596326	5.752610514	5.503715745	14627	Trmt10b	6.521601776	6.345680772	6.535065857
828	Abcg4	7.249587651	7.046035489	7.109228611	3128	Chka	6.348199369	6.497411803	6.389277382	5428	Fkbp9	7.42981575	7.753444694	7.519155866	7728	Klhl4	5.782021958	5.969800035	6.00279947810028		Parp3	3.998780302	3.985393908	5.069606697	12328	Selplg	6.541160789	6.886116442	6.388706251	14628	Trmt10c	6.807604896	6.873995258	6.787506958
829	Abcg5	0.019276875	1.497721125	1.574435719	3129	Chkb	5.936883304	5.861450368	6.046804016	5429	Fkbpl	5.745865587	5.546686776	4.732630842	7729	Klhl40	5.597134357	5.268302577	4.93337010910029		Parp4	4.257563112	5.318719905	5.152063157	12329	Selt	9.691431521	9.632338027	9.709650588	14629	Trmt11	5.716914085	5.042267691	5.149716516
830	Abhd1	6.186907168	6.235283323	6.109573241	3130	ChkbCpt1b	4.984152721	5.278988952	4.623083271	5430	Fkrp	7.515998248	7.75182518	7.793965768	7730	Klhl41	2.063383869	1.437607652	0.9807757110030		Parp6	7.962800971	8.211468991	7.874791526	12330	Sema3a	6.556760607	6.73500164	7.219674343	14630	Trmt112	8.643037865	8.723575018	8.8930053
831	Abhd10	6.032844939	5.765566926	6.321100488	3131	Chl1	10.95371121	10.54419695	10.51329076	5431	Fktn	7.87479816	7.877102471	7.721294651	7731	Klhl42	5.833042798	5.722167205	5.81339303110031		Parp8	3.565164204	4.844525647	5.069185976	12331	Sema3b	4.253270216	5.028097543	3.884213008	14631	Trmt12	5.859164339	5.384675766	5.652397891
832	Abhd11	6.8228797	7.088342046	6.8868053	3132	Chm	8.978266019	8.873720562	8.980020018	5432	Flad1	5.718017466	5.156073954	5.14941868	7732	Klhl5	8.162477678	8.164709857	8.25159767410032		Parp9	4.934151987	5.018633869	4.225691323	12332	Sema3c	7.445825057	7.338279885	7.346399184	14632	Trmt13	4.665831519	4.795721409	4.62356859
833	Abhd11os	6.681372342	6.927769914	6.707748245	3133	Chml	5.98911751	5.807390764	6.789013266	5433	Flcn	6.875345605	6.847902223	6.945825733	7733	Klhl6	3.869785218	2.835873519	2.5632437310033		Parpbp	4.156962945	3.648004789	4.152582194	12333	Sema3d	5.833761482	5.908914807	5.623545658	14633	Trmt1l	6.808592763	6.706202622	6.761213196
834	Abhd12	10.00546093	10.21539584	10.05946675	3134	Chmp1a	9.214616468	9.258674936	9.043366567	5434	Fli1	5.035078426	4.575688675	5.067496489	7734	Klhl7	8.446400835	8.263638814	8.27914969610034		Pars2	5.038105761	5.682957591	5.108745701	12334	Sema3e	8.687315853	8.814079868	8.372169939	14634	Trmt2a	7.145214105	6.858452784	7.237888089
835	Abhd13	7.620404799	7.631081742	7.83809714	3135	Chmp1b	8.096951145	8.057430984	8.119662274	5435	Flii	7.597046674	7.889275703	7.844319827	7735	Klhl8	7.497659484	7.39252396	7.51156957210035		Particl	6.472044954	6.376624127	6.503826764	12335	Sema3f	2.693432377	4.016787113	4.627495552	14635	Trmt2b	6.269950668	6.15810346	6.227900104
836	Abhd14a	6.766950609	7.052098294	6.838147294	3136	Chmp2a	8.891162372	8.990576455	8.850314362	5436	Flna	6.062300115	6.635778042	6.550090439	7736	Klhl9	8.507405413	8.582066834	8.89602706810036		Parva	7.80203243	8.058741369	7.761201567	12336	Sema4a	6.964994532	7.307001179	7.431606136	14636	Trmt44	3.778325758	4.178891991	4.230561704
837	Abhd14b	5.36407655	5.70857526	5.189224635	3137	Chmp2b	8.6037283	8.506809573	8.372193791	5437	Flnb	6.012395062	6.489830495	6.002054276	7737	Klk10	2.059619717	1.87302078	0.9821415310037		Parvb	6.520313392	6.176583947	6.045550935	12337	Sema4b	5.504706929	5.115496061	5.595518974	14637	Trmt5	4.933359932	5.139159514	4.147497803
838	Abhd15	3.392036667	3.56395276	2.298661714	3138	Chmp3	9.902302862	9.902634222	9.944348848	5438	Flnc	3.064684286	2.157164475	3.444696356	7738	Klk11	1.622202045	0.921259719	0.99043680410038		Parvg	4.733683698	5.708323238	5.302102987	12338	Sema4c	5.696866603	5.692634963	6.190568003	14638	Trmt6	8.484981654	8.393103929	8.154762011
839	Abhd16a	8.588440722	8.690054683	8.645346884	3139	Chmp4b	10.70538407	10.69652343	10.82392403	5439	Flot1	8.847730991	8.767024388	8.319636527	7739	Klk6	4.550679539	5.003895867	5.30260681510039		Pask	2.647384053	2.179775461	2.305019738	12339	Sema4d	5.437362198	5.570808201	5.653339104	14639	Trmt61a	5.862762098	6.050168918	6.473353202
840	Abhd17a	8.485822347	8.538398723	8.591071128	3140	Chmp5	10.0959991	10.07734078	10.14364426	5440	Flot2	8.057240678	8.120272889	7.88078529	7740	Klk7	2.394906849	2.164902869	1.97985274510040		Patl1	7.208608509	6.857168344	7.078072199	12340	Sema4f	6.553965931	6.279629196	6.389524556	14640	Trmt61b	6.454930945	6.61022412	6.624416118
841	Abhd17b	9.38890039	9.461991295	9.523082393	3141	Chmp6	6.325990162	6.160901483	5.979595184	5441	Flrt1	8.485528513	8.514306863	8.806840821	7741	Klk8	5.207763883	5.303967872	4.56260117910041		Patz1	6.997604809	6.815567896	6.734754159	12341	Sema4g	5.078299666	4.967817632	5.536759806	14641	Trmu	4.472370286	4.357969319	4.301216168
842	Abhd17c	8.184046571	8.370289673	8.077855726	3142	Chmp7	8.356471482	8.361829425	8.499805795	5442	Flrt2	9.008542609	9.137270356	9.286162526	7742	Klrg1	3.511238552	2.177395101	2.79204498210042		Pawr	2.69903678	4.615131537	2.555563833	12342	Sema5a	9.28561118	9.72880327	9.218239588	14642	Trnau1ap	5.66943884	5.857716581	6.209540654
843	Abhd2	3.771485982	4.263886292	3.566325373	3143	Chn1	12.77973011	12.79161206	12.65680982	5443	Flrt3	8.81735761	8.47102203	8.324170979	7743	Kmt2a	10.3577199	10.29839774	10.317626810043		Pax3	1.685610274	1.802396358	3.569438133	12343	Sema5b	6.117354385	5.891501381	5.471622794	14643	Trnp1	9.678480663	9.688296402	9.414526392
844	Abhd3	7.376319719	7.260468412	7.519093843	3144	Chn1os3	11.93501762	11.87159195	11.64751631	5444	Flt1	7.221162035	7.329156546	7.645749413	7744	Kmt2b	5.964241611	5.922275108	5.93383357210044		Pax6	6.487562265	6.882810479	5.785866148	12344	Sema6a	8.023239769	8.066724327	8.194454048	14644	Trnt1	7.808924336	7.814335239	8.145188141
845	Abhd4	8.811892213	8.840782891	8.69069865	3145	Chn2	8.420345176	8.51456496	8.561326893	5445	Flt3	4.476306337	4.474936313	4.6805217	7745	Kmt2c	9.680255205	9.599516456	9.52879259210045		Pax6os1	2.051291688	1.468413941	2.309699581	12345	Sema6b	7.328120332	7.229506115	7.319909575	14645	Tro	10.26264349	9.881841232	9.916585678
846	Abhd5	6.650406309	6.782683101	6.354286867	3146	Chodl	3.533253982	3.55156299	4.154106559	5446	Flt3l	1.705312925	3.433396753	4.071517853	7746	Kmt2d	7.980866847	8.003995583	8.17509971910046		Pax7	3.110950443	2.756654252	5.231522417	12346	Sema6c	4.474863864	5.178455273	4.680863287	14646	Trove2	9.568436357	9.565676151	9.616622583
847	Abhd6	8.814829103	8.720746006	8.669978814	3147	Chordc1	9.252484813	8.956313338	8.974290956	5447	Flt4	1.052475859	0.889981576	0.98674131	7747	Kmt2e	10.73875641	10.65630712	10.8160552810047		Pax9	0.027275646	1.484422505	0.987305022	12347	Sema6d	6.987863865	7.366003376	6.933972713	14647	Trp53	4.894794421	5.297274104	5.372592384
848	Abhd8	9.646580577	9.710007037	9.853522152	3148	Chp1	9.270327455	9.299540365	9.101436919	5448	Flywch1	10.1572881	10.09523209	10.16364375	7748	Kndc1	8.298637891	8.165211291	8.09897654310048		Paxbp1	6.990005268	7.439157623	7.25598603	12348	Sema7a	8.103682338	8.354101924	8.499831069	14648	Trp53bp1	8.926207113	8.851167066	9.029863684

849	Abi1	9.219997289	9.146054843	9.139837984	3149	Chp2	2.390281292	2.175303857	2.791840043	5449	Flywch2	6.681906517	6.399609186	7.247574327	7749	Kng1	3.651836896	1.778868033	2.5628692110049	Paxip1	7.056613043	6.801835566	6.910725646	12349	Senp1	6.170850511	6.482886493	6.5653061	14649	Trp53bp2	6.58988733	6.99155	6.388499932
850	Abi2	10.15342161	10.26529235	10.36049237	3150	Chpf	8.288358919	7.736758189	7.93418219	5450	Fmn1	7.963104433	7.748815141	8.484391608	7750	Kng2	2.643685882	0.827132111	2.30606654210050	Pbdc1	7.632937527	7.288923135	7.40643184	12350	Senp2	8.7806194	8.654770869	8.834985485	14650	Trp53cor1	2.892629387	4.040475955	3.302886171
851	Abi3	5.72446046	5.994117148	5.980784199	3151	Chpf2	5.889965767	6.384202786	5.933822925	5451	Fmn2	9.97516894	9.757948103	10.05687957	7751	Knop1	8.932365748	9.153967027	8.86867177410051	Pbk	2.65179647	2.441701367	2.303767683	12351	Senp3	6.195573446	6.79149977	6.69394561	14651	Trp53i11	7.839625188	7.647766083	6.933291988
852	Abi3bp	4.077981812	3.398441101	4.441625515	3152	Chpt1	7.430936011	7.833565474	7.526629215	5452	Fmnl1	7.656997923	7.94599629	7.471799665	7752	Knstrn	1.638245658	1.472336882	0.98563934210052	Pbld1	2.658810274	2.857224187	2.983020002	12352	Senp5	7.732555541	7.903862384	8.035418072	14652	Trp53i13	4.324451773	2.950646621	3.144106722
853	Abl1	6.753206962	6.861604477	7.046633112	3153	Chrac1	6.832808298	6.842957742	6.69456104	5453	Fmnl2	8.498058345	8.493092914	8.59486432	7753	Kntc1	3.066493263	3.461911728	0.97246844910053	Pbld2	1.630828919	0.899664501	0.987881221	12353	Senp6	9.356679406	9.409803081	9.308636536	14653	Trp53inp1	7.033266883	6.926408231	6.651953275
854	Abl2	8.804718559	8.913240634	8.579808647	3154	Chrd	5.74557473	5.081038685	5.406913314	5454	Fmnl3	4.251052289	4.60387934	4.982871218	7754	Kpna1	9.363537491	9.205892631	9.50560387410054	Pbrm1	9.915061424	9.75917156	10.05148771	12354	Senp7	8.379724665	8.373139303	8.534725272	14654	Trp53inp2	9.170748067	9.364035292	9.11948874
855	Ablim1	9.164904052	9.064632904	9.152517038	3155	Chrdl1	5.164715691	4.833380736	5.3032248	5455	Fmo1	4.658531723	4.364659444	4.504598544	7755	Kpna2	4.831286064	4.53602134	4.37124119210055	Pbx1	11.43152176	11.57433243	11.48430923	12355	Senp8	6.327369195	6.120816443	6.246822126	14655	Trp53rka	7.392680946	7.322154311	7.406455996
856	Ablim2	8.150154891	7.372383751	8.035585644	3156	Chrm1	9.393030382	9.314009618	9.201567816	5456	Fmo2	1.674807902	2.663915977	1.979154446	7756	Kpna3	9.37911562	9.044572191	9.18215629210056	Pbx2	7.822119426	7.968356373	7.793707209	12356	Sep15	9.525851794	9.513231927	9.659457035	14656	Trp53rkb	4.982241783	4.532973357	4.935258107
857	Ablim3	6.231862095	6.07936944	6.488080878	3157	Chrm2	5.380099599	6.018992576	6.911883101	5457	Fmo5	4.082291368	4.368493066	4.373239469	7757	Kpna4	8.515316786	8.524151563	8.67357281910057	Pbx3	7.127962421	7.350894179	7.917102524	12357	Sephs1	6.438469618	6.567562115	6.168988934	14657	Trp63	2.078787295	2.668496439	2.790832137
858	Abr	9.55020651	9.685341184	9.546036394	3158	Chrm3	8.080046275	8.028152253	8.194554397	5458	Fmod	5.431562666	4.336202255	4.886357024	7758	Kpna6	7.29946072	7.499082183	6.96812169310058	Pbx4	4.888253384	4.925774825	5.266783402	12358	Sephs2	6.998799123	6.936038743	7.035490213	14658	Trp73	3.248977281	4.525661847	2.7850991
859	Abra	1.630810687	0.899710776	1.573372238	3159	Chrm4	6.949160975	6.669285567	6.774455361	5459	Fmr1	8.255434818	8.136174005	8.423104324	7759	Kpnb1	8.684641273	8.904309437	8.84417955810059	Pbxip1	9.054578174	9.151922184	8.841024697	12359	Sepn1	4.267187359	5.269139482	5.33844169	14659	Trpa1	2.63320571	1.461079005	2.309023135
860	Abracl	6.211834262	6.541251272	6.694977468	3160	Chrm5	5.448759962	3.836978094	3.145808258	5460	Fn1	6.94875989	6.072773275	7.089310599	7760	Kptn	5.324606202	5.00529803	5.02415141210060	Pcbd1	5.210724617	5.079845701	4.978804859	12360	Sepp1	10.26429488	10.23096608	10.28146249	14660	Trpc1	5.075847529	4.885133335	4.734605953
861	Abt1	7.799779322	7.459549764	7.557522009	3161	Chrna1	4.583802786	3.7585153	3.982726974	5461	Fn3k	6.290099459	6.47068487	6.4722924	7761	Kras	10.32705922	10.1710801	10.1722904710061	Pcbd2	6.695316737	6.720823135	7.068222256	12361	Sepsecs	2.37407349	0.862878394	2.79540174	14661	Trpc3	5.24985555	5.120336559	5.440267237
862	Abtb1	5.938261865	6.050429954	6.356276015	3162	Chrna3	2.688097011	4.389198355	3.981849199	5462	Fn3krp	2.039538349	0.910090253	0.989113126	7762	Krba1	5.087359887	5.510347621	5.59500907810062	Pcbp1	9.684094352	9.672436523	9.530687426	12362	Sept1	1.647995485	2.703171284	2.571867797	14662	Trpc4	7.996287145	7.607802471	7.045362061
863	Abtb2	5.364525098	4.726130692	4.561741303	3163	Chrna4	6.742011621	6.900485502	7.542939118	5463	Fnbp1	8.304805587	8.325371059	8.393639232	7763	Krcc1	8.599755909	8.499766104	8.49964167610063	Pcbp2	10.02306147	9.950104569	10.07408309	12363	Sept10	5.607632422	6.273554971	5.812724557	14663	Trpc4ap	8.926173309	8.943601329	9.08334493
864	Acaa1a	6.508217516	6.940854672	6.594568722	3164	Chrna5	2.88606787	3.015778606	2.563626331	5464	Fnbp1l	8.838731764	8.923193209	9.137543953	7764	Kremen1	4.478124501	4.744258268	5.22978321810064	Pcbp3	7.034812767	7.037428324	7.31139571	12364	Sept11	7.499272464	7.769969687	7.721567107	14664	Trpc5	6.780361015	6.708123572	6.800361568
865	Acaa1b	3.664096144	3.646875276	4.375739665	3165	Chrna6	2.112431718	2.814864979	4.306231659	5465	Fnbp4	7.229300949	7.142259555	7.237630264	7765	Kremen2	2.659463388	2.42450013	2.56593890310065	Pcbp4	10.92056433	11.22191378	10.93636127	12365	Sept2	8.808879956	8.925172254	8.999609328	14665	Trpc6	7.207817643	6.924836313	6.707377972
866	Acaa2	7.37487814	7.505352567	6.956578272	3166	Chrna7	2.38329368	2.190919728	0.979866769	5466	Fndc1	2.39303846	1.402472897	1.563393888	7766	Kri1	6.949172982	6.850738853	6.72138500310066	Pcca	6.604155567	6.941604269	6.28293377	12366	Sept3	11.6148682	11.56697542	11.53264346	14666	Trpc7	5.466057164	5.007193875	5.473517929
867	Acaca	7.571136903	7.561198652	7.572752169	3167	Chrnb1	2.660255506	3.459357633	3.569973325	5467	Fndc3a	8.034749733	7.880819533	8.315679984	7767	Krit1	7.523899448	7.579195664	7.70807757210067	Pccb	8.418615344	8.09596127	7.928253445	12367	Sept4	10.55536436	10.69978847	10.63461916	14667	Trpm2	6.291799675	6.581707785	6.372085631
868	Acacb	4.319751958	4.379997115	2.974689983	3168	Chrnb2	6.403521648	6.51040853	6.301390695	5468	Fndc3b	6.509093346	6.839500833	6.694124165	7768	Krr1	8.442118094	8.130797075	8.28827102210068	Pcdh1	6.519361112	6.084424351	6.208993567	12368	Sept5	11.92801127	11.96907213	11.9576253	14668	Trpm3	7.117333171	7.949871395	7.602020784
869	Acad10	0.052814502	2.70095729	0.982154434	3169	Chrnb3	3.08350586	3.156621367	4.154028986	5469	Fndc4	7.987075039	7.881932334	8.083475597	7769	Krt1	4.169225396	4.366184629	4.50477961310069	Pcdh10	9.508803648	9.296669317	9.699488332	12369	Sept6	9.918298182	9.925109632	10.14745379	14669	Trpm4	4.465885402	4.76741331	4.505406164
870	Acad11	5.299568742	6.089204597	5.98010359	3170	Chrnb4	0.07193582	3.710090194	3.572708702	5470	Fndc5	9.160543513	9.212166307	9.495739112	7770	Krt10	6.781869083	7.069620474	6.87479252110070	Pcdh11x	7.982645231	8.212532574	8.673857569	12370	Sept7	12.42292681	12.26424748	12.41095632	14670	Trpm6	2.665175322	1.796532644	2.564452821
871	Acad12	1.05878661	1.891596586	1.987009903	3171	Chrne	2.384258506	3.485647321	0.979469438	5471	Fndc9	2.891235655	2.827860802	4.071943433	7771	Krt12	4.417719008	5.194950747	5.68210315610071	Pcdh15	5.56603517	5.368109627	5.596341309	12371	Sept8	9.952282174	9.946681456	9.897381502	14671	Trpm7	7.980887181	7.636277316	7.844132417
872	Acad8	6.710572183	7.071297669	6.666225142	3172	Chst1	11.81174968	11.60566245	11.51720069	5472	Fnip1	8.959902088	8.831952427	8.948702601	7772	Krt15	3.658829819	3.546627536	3.56612803210072	Pcdh17	10.22364937	10.01277981	9.916592944	12372	Sept9	5.401716604	5.827742906	5.565713052	14672	Trps1	8.880657825	8.689961717	8.673508515
873	Acad9	7.091573056	7.006874169	7.2282159	3173	Chst10	8.203776939	8.007748508	8.07809713	5473	Fnip2	5.570289275	5.151305915	5.406174315	7773	Krt17	1.636488481	1.898323853	0.98617443610073	Pcdh18	4.891466929	4.874797813	5.710072399	12373	Sepw1	10.63220693	10.83263611	10.46880597	14673	Trpt1	6.053735602	5.615977148	5.734872417
874	Acadl	8.26523484	8.159283378	8.460002178	3174	Chst11	7.709954326	7.856074802	7.673412369	5474	Fnta	8.572769331	8.571873035	8.701101406	7774	Krt18	5.505017389	4.995352399	3.9726538610074	Pcdh19	9.419729638	8.932298634	8.974245394	12374	Serac1	5.862520502	6.147146438	5.934260522	14674	Trpv2	6.221987648	5.508677439	5.652717375
875	Acadm	7.650614514	7.816493133	7.652365051	3175	Chst12	5.03581889	5.123917807	5.302692141	5475	Fntb	6.835714339	6.893553432	7.140402482	7775	Krt19	0.013866364	1.507532306	0.99044063710075	Pcdh20	9.240159111	9.25626094	8.601684368	12375	Serbp1	12.31721917	12.30941685	12.40860234	14675	Trpv4	3.077068558	4.472736386	2.298322082
876	Acads	5.777848799	6.09925159	5.065527558	3176	Chst14	3.875127738	4.117844532	4.377283163	5476	Focad	6.058708011	6.474388127	6.001969685	7776	Krt2	3.972454213	4.119060189	2.97896647310076	Pcdh7	9.240799649	9.209884764	9.907983692	12376	Serf1	7.114190459	7.471607714	7.320153705	14676	Trpv6	2.658044741	2.658894362	3.444410821
877	Acadsb	8.684298976	8.494643298	8.83493979	3177	Chst15	6.899597597	6.938624872	7.238304722	5477	Folh1	4.978417379	3.09358937	5.192391783	7777	Krt222	6.962341765	6.999896273	6.99147849310077	Pcdh8	7.176794861	6.741217838	6.707379463	12377	Serf2	11.75568148	11.82755257	11.69978495	14677	Trrap	7.696114085	7.904446881	7.95739827
878	Acadvl	7.391675211	7.055642378	6.933766361	3178	Chst2	10.05920443	10.18478002	9.912165878	5478	Folr1	6.171995189	7.857145271	5.43592127	7778	Krt26	1.045688182	0.904765707	1.57388637610078	Pcdh9	10.99267952	10.9139101	11.0264384	12378	Sergef	5.661552363	5.513794878	5.337348254	14678	Trub1	6.755288904	6.823344646	6.91049868
879	Acan	4.319408921	3.837683369	4.151395085	3179	Chst3	2.091549248	2.646968236	3.569317032	5479	Folr2	2.639787302	0.842376884	1.984180182	7779	Krt73	4.072862187	4.510251097	3.56435601310079	Pcdha11	3.403420094	3.14621128	4.443691998	12379	Serhl	5.437973749	5.959976006	5.301428312	14679	Trub2	6.871889198	6.593635508	6.283088046
880	Acap2	8.14649308	8.021924285	8.184634819	3180	Chst5	1.652474474	1.440703202	2.794100882	5480	Fopnl	9.124608261	8.956594364	9.043413215	7780	Krt8	5.566938231	4.871315546	3.77957809910080	Pcdha4	0.005608334	0.940318714	0.992698372	12380	Serinc1	11.29615591	11.31549555	11.41461476	14680	Tsacc	3.402058478	4.329842405	4.15336948
881	Acap3	8.130338503	8.351488893	8.218500879	3181	Chst7	3.400239945	3.762140508	4.153732301	5481	Fos	6.329820083	4.788540641	6.455724597	7781	Krt85	0.029957615	0.889847921	1.57237875310081	Pcdha5	1.669322361	2.671881152	2.791225568	12381	Serinc2	5.63687025	6.375360009	5.40497721	14681	Tsc1	7.709888223	7.775206705	7.59453886
882	Acat1	9.808412033	9.648033212	9.772778188	3182	Chst8	6.122484504	5.689786297	5.980160765	5482	Fosb	5.83292004	5.106823755	5.62375792	7782	Krt9	5.4047862	5.845273001	5.76203454110082	Pcdha9	1.092985802	2.872570994	2.568167584	12382	Serinc3	10.77668125	10.81010381	10.71806009	14682	Tsc2	8.010755837	7.952039297	7.98571047
883	Acat2	9.245506086	9.329964997	9.223137586	3183	Chst9	3.650898445	2.123618672	3.684120335	5483	Fosl1	3.073231846	2.641524901	3.152032794	7783	Krtcap2	8.871105482	8.757193292	8.65592827310083	Pcdhac1	10.30792449	10.24116005	10.20280957	12383	Serinc4	6.404950821	6.875217065	6.5496106	14683	Tsc22d1	11.49310033	11.4100305	11.38192401
884	Acat3	9.491987272	9.435831741	9.439411832	3184	Chsy1	6.489830305	6.539435443	6.4718212	5484	Fosl2	7.846704036	7.051028039	7.0667077	7784	Krtcap3	1.101659475	2.86314867	3.1542976410084	Pcdhb10	3.235155906	1.810226817	3.892967622	12384	Serinc5	7.847528418	7.658283474	7.455452961	14684	Tsc22d2	7.63531265	7.341839766	7.899164506
885	Acbd3	8.937787934	8.720647309	8.724844666	3185	Chsy3	4.842793084	5.364283266	5.763070317	5485	Foxb1	2.698859825	3.120880695	4.567636926	7785	Ksr1	2.8867444	3.564819156	2.98070903410085	Pcdhb11	3.650002141	0.717823574	2.980794378	12385	Serp1	8.518802175	8.55333508	8.251329818	14685	Tsc22d3	8.839279938	8.776146891	8.880761138
886	Acbd4	7.268168722	7.077661438	7.209233128	3186	Chtf18	7.780647708	7.816129784	7.774477567	5486	Foxc1	5.695797615	5.104283153	6.339311421	7786	Ksr2	5.934938264	5.721960591	5.88760180210086	Pcdhb12	4.978684084	3.902253863	4.503725416	12386	Serp2	9.50454109	9.339383273	9.641871308	14686	Tsc22d4	9.290295986	9.315166042	9.299594457
887	Acbd5	9.196918919	9.253792973	9.299710925	3187	Chtf8	9.566126287	9.492691133	9.427065652	5487	Foxc2	3.969803425	3.007707445	4.233151253	7787	Kti12	5.983371339	5.771084067	6.00339921410087	Pcdhb13	2.401669075	3.188944328	2.982838716	12387	Serpina1a	4.837938955	2.49943203	4.224203631	14687	Tsen15	6.679969461	6.578364627	6.623831238
888	Acbd6	7.881974931	8.332632787	8.194540837	3188	Chtop	9.272946538	9.434495874	9.465891509	5488	Foxd1	3.892875888	4.552937945	4.790135007	7788	Ktn1	10.22284055	10.34258618	10.5326950510088	Pcdhb14	5.96066846	5.885164115	5.76132514	12388	Serpina1b	5.802609932	3.449091138	4.060728054	14688	Tsen2	5.857135591	5.914335718	5.148508645
889	Acbd7	2.860465168	2.694584851	1.983877495	3189	Chuk	6.249718522	6.003587237	6.284043774	5489	Foxd2	3.059021611	1.839192037	3.687681164	7789	Kxd1	6.311340821	6.580953457	6.24611738810089	Pcdhb15	3.069099189	2.851732387	2.300959131	12389	Serpina1c	6.362960229	3.290678864	4.219122988	14689	Tsen34	9.377959143	9.927542614	9.538364911
890	Accs	2.083469298	3.464174469	3.686398929	3190	Churc1	6.168965992	6.544198131	6.320024411	5490	Foxf1	0.050360071	2.47553227	0.982621713	7790	Ky	4.16400596	4.615146987	4.22917260610090	Pcdhb16	5.250049755	5.194430015	5.763218025	12390	Serpina1d	4.605484861	0.396495236	2.774178712	14690	Tsen54	3.256184016	3.753731666	4.376073688
891	Acd	8.486480826	8.795380409	8.922648952	3191	Ciao1	7.665463514	7.677271016	7.54968727	5491	Foxf2	1.670336794	2.670405667	2.98444436	7791	Kynu	0.027273188	0.89477931	2.31118960410091	Pcdhb17	5.574728828	5.588678617	6.089597106	12391	Serpina1e	5.575597937	2.180990032	3.668557145	14691	Tsfm	5.436583098	5.425309303	5.337538733
892	Ace	5.174821896	6.628603094	4.560313485	3192	Ciapin1	7.628711167	7.681544202	8.145237715	5492	Foxg1	9.526791173	9.256168002	9.258498583	7792	L1cam	8.356126512	8.094171228	8.5038200110092	Pcdhb18	5.693217989	5.593913403	5.405725461	12392	Serpina3c	1.713964774	1.294441967	4.98621259	14692	Tsg101	7.98743291	8.192802883	7.996808795
893	Ace2	2.656446835	2.431283354	3.306723703	3193	Ciart	5.086360344	5.42218526	5.814132797	5493	Foxj1	6.737799421	7.257168519	6.35431952	7793	L2hgdh	6.264720035	5.472677872	6.1307363310093	Pcdhb19	4.890939938	5.337784129	5.302824065	12393	Serpina3f	1.628784125	1.493109263	0.988491805	14693	Tsga10	6.490355687	6.465928861	6.594852061
894	Acer2	3.407336127	3.533967833	3.788110788	3194	Cib1	6.555439825	6.510994325	6.503625072	5494	Foxj2	7.069923867	7.282505857	7.228040298	7794	L3hypdh	2.889470111	3.440667559	3.79109280410094	Pcdhb2	0.107960009	3.033215358	3.306246892	12394	Serpina3g	2.049719566	1.894937247	1.571460045	14694	Tshb	3.417968492	5.230503749	0.948416653
895	Acer3	6.329644029	6.437754678	6.624302553	3195	Cib2	5.669855372	6.107327309	6.067681616	5495	Foxj3	7.01093159	7.251407945	7.228410363	7795	L3mbtl1	0.060243404	2.466171554	1.56740967710095	Pcdhb20	6.03546693	6.005678108	6.228296122	12395	Serpina3k	5.543291624	0.936241913	4.559621123	14695	Tshr	2.05963167	2.203353836	2.307760476
896	Ache	6.635219857	6.632039485	6.652432909	3196	Cic	8.539350693	8.604331052	8.447663179	5496	Foxk1	8.141073505	8.319391494	7.922294257	7796	L3mbtl2	8.308677608	8.281872522	8.24218132610096	Pcdhb21	4.600219723	4.223741912	4.982436555	12396	Serpina3n	7.366042575	7.641904224	6.622359722	14696	Tshz1	8.506710941	8.50692982	8.800432122
897	Acin1	9.993124359	9.735531244	9.754761067	3197	Cidea	5.960698476	6.597251499	5.106724985	5497	Foxk2	7.867807049	7.750752465	7.741528391	7797	L3mbtl3	6.588526115	6.734959343	6.56484834810097	Pcdhb22	4.782689853	4.970016652	4.934413199	12397	Serpina6	0.019270911	1.497731521	0.989119425	14697	Tshz2	8.753115966	8.774348631	8.758034725
898	Ackr1	7.401864943	7.254919577	7.301896445	3198	Cideb	5.940256308	5.877395726	6.247256474	5498	Foxl1	0.008382678	0.933572806	0.991898172	7798	l7Rn6	7.580986526	7.856491828	7.92282318610098	Pcdhb3	4.325754776	3.914268441	4.682587741	12398	Serpina9	2.884741013	3.318847149	2.563999868	14698	Tshz3	7.489975551	7.716891623	7.905378596

899	Ackr2	1.670384548	2.440090947	2.984436797
900	Ackr3	5.911131566	5.588700839	5.470897135
901	Ackr4	1.052524405	0.889875332	0.986728828
902	Acly	8.643104758	8.902158069	8.797013638
903	Acmsd	2.66570152	1.355224187	3.152181386
904	Acnat2	3.075557044	3.675283113	3.304169382
905	Aco1	6.451821225	6.123418984	6.265814046
906	Aco2	10.86274017	10.90330689	10.74151244
907	Acot1	6.691875343	6.858311674	6.149330632
908	Acot10	3.88710348	4.443618527	4.151331153
909	Acot11	6.586504435	6.526666891	6.108439174
910	Acot12	1.052497057	1.901917015	1.572387487
911	Acot13	10.81936585	10.68255868	10.53262339
912	Acot2	7.545669416	7.40298141	7.109126684
913	Acot3	2.108193729	4.038147298	2.78510722
914	Acot4	2.652576509	2.439957017	3.445469016
915	Acot5	4.268755771	5.334181528	5.149782195
916	Acot6	1.63997322	1.468525547	1.987006364
917	Acot7	10.39441106	10.32583493	10.23854762
918	Acot8	6.569028472	6.10125358	6.372627953
919	Acot9	5.662230462	5.651094508	5.188486261
920	Acox1	8.419584447	8.443167898	8.19427959
921	Acox3	5.941872233	6.348501564	6.40677244
922	Acp1	8.535830747	8.674881195	8.341768877
923	Acp2	9.010873955	9.062882074	8.8191597
924	Acp5	1.064652196	0.86306045	0.983598579
925	Acp6	1.038065181	0.921238173	0.990434248
926	Acr	2.434570589	4.625778198	4.50730794
927	Acrbp	3.236194697	3.028007591	0.970995552
928	Acsbg1	7.680879156	7.756735952	7.748163635
929	Acsf2	2.884500216	1.792261226	3.792022549
930	Acsf3	4.400372509	4.227614045	4.06633022
931	Acsl1	7.793762836	7.752231932	7.735044287
932	Acsl3	11.2679829	11.28221464	11.27056365
933	Acsl4	9.151594829	9.03775556	9.048803059
934	Acsl5	7.880496379	7.895055462	8.08375285
935	Acsl6	8.802242927	8.740288145	9.199268742
936	Acsm3	5.324795898	5.234465026	4.933520852
937	Acss1	6.948203558	6.507613193	6.487390862
938	Acss2	6.823317325	6.967011077	7.218857765
939	Acta1	5.804018201	5.800369711	5.958816136
940	Acta2	6.886596332	6.447072123	6.77447741
941	Actb	13.81372627	13.97060042	13.71171522
942	Actg1	10.95085357	10.8441348	10.84029257
943	Actl6a	6.487706038	6.61259516	6.189185461
944	Actl6b	9.646378196	9.687029738	9.52301839
945	Actl7a	0.040181771	3.376640109	2.309378887
946	Actl9	2.625525211	1.905537156	1.5728679
947	Actn1	8.972095913	9.043223937	8.907779528
948	Actn2	4.595449175	4.553456158	4.737531088

3199	Ciita	1.0701825	1.447438457	1.56855641
3200	Cilp2	4.531443818	4.236377867	4.683095934
3201	Cinp	6.767783207	6.958549636	7.120082447
3202	Cipc	7.580556517	7.585798942	7.652478366
3203	Cir1	8.580110243	8.623295958	8.451620154
3204	Cirbp	8.318807814	8.435171958	8.402167801
3205	Cirh1a	7.447746608	7.480463403	7.328312835
3206	Cisd1	9.655704974	9.345222935	9.505532362
3207	Cisd2	8.406977858	8.407713785	8.714963385
3208	Cisd3	8.701654803	8.463756561	8.59851598
3209	Cish	5.075300648	5.347368373	4.225287035
3210	Cit	6.796259188	6.317920292	7.588417676
3211	Cited1	4.662072392	4.596960091	4.439256022
3212	Cited2	7.393973705	7.647197641	7.594878016
3213	Cited4	5.12382938	5.402627081	5.76382366
3214	Ciz1	6.780671708	7.211486279	6.813024466
3215	CK137956	2.07455113	2.173505009	2.304293861
3216	Ckap2	2.875157893	3.194213694	2.302407211
3217	Ckap2l	3.400210669	3.762194245	3.566287459
3218	Ckap4	7.166644065	6.880428796	6.991208484
3219	Ckap5	9.366156975	9.327166372	9.699482684
3220	Ckb	12.34831642	12.5588652	12.63528838
3221	Cklf	3.767357643	3.005501906	3.150426589
3222	Ckm	0.04282195	0.867268628	3.797800788
3223	Ckmt1	9.887999896	9.91590326	9.965834518
3224	Cks1b	5.430268814	4.68750126	4.786740201
3225	Cks1brt	0.013868036	0.921288451	0.990440213
3226	Cks2	3.394452891	2.830183508	3.683620421
3227	Clasp1	8.087041958	8.055132699	8.451819193
3228	Clasp2	10.08767816	10.09388807	10.26657562
3229	Clasrp	5.405717687	6.227296996	5.471299378
3230	Clca3a2	1.056802887	0.880479506	1.571447093
3231	Clcc1	7.880310476	7.775550369	7.29228388
3232	Clcf1	1.043251762	0.910042488	1.574426569
3233	Clcf1-pold4	4.935557392	5.05662487	4.980885691
3234	Clcn1	3.065334116	3.334740703	2.983356398
3235	Clcn2	9.918122928	9.913419426	9.731474747
3236	Clcn3	10.81615553	10.82076023	10.8065225
3237	Clcn4-2	10.0986855	9.970786302	10.0950458
3238	Clcn5	4.08106117	4.161625237	4.067244262
3239	Clcn6	1.674828532	2.863369626	2.566933119
3240	Clcn7	5.864063773	5.87850823	6.3384021
3241	Cldn1	4.339949054	5.498469737	4.298904349
3242	Cldn10	8.848702758	8.695018605	8.694127488
3243	Cldn11	10.21759264	10.47311181	9.992530688
3244	Cldn12	6.574488553	6.850017041	7.190236503
3245	Cldn14	2.403886645	3.788665381	2.565373362
3246	Cldn2	5.401394774	6.304375377	3.97292852
3247	Cldn22	3.66626678	4.62366565	3.299676509
3248	Cldn3	2.872145031	2.668657262	0.975020371

5499	Foxl2	1.674996582	2.159187127	3.444854351
5500	Foxm1	2.071474665	2.87923361	1.564989662
5501	Foxn2	6.058150234	5.831674575	6.209162661
5502	Foxn3	7.808316516	7.916016716	7.887016314
5503	Foxo1	7.695591759	7.937713412	7.463385772
5504	Foxo3	1.665709177	2.67714585	2.985179154
5505	Foxo4	6.635575062	6.829092406	6.991883084
5506	Foxo6	7.46633242	7.063731939	6.168536791
5507	Foxp1	10.85789422	10.93714794	11.2249887
5508	Foxp2	7.551607013	8.230045302	8.850891262
5509	Foxp3	6.225240166	6.357951884	6.110174499
5510	Foxp4	6.572691905	6.96298092	6.708155098
5511	Foxq1	5.832829081	5.385096911	5.564679567
5512	Foxr2	1.060822229	0.871590837	1.986622254
5513	Foxred1	6.312290431	6.537306166	6.623927371
5514	Foxred2	5.834598728	5.531717544	5.7076904
5515	Foxs1	0.040332334	2.217048578	1.570567807
5516	Fpgs	4.601953643	5.143983986	4.565305289
5517	Fpgt	5.776078584	5.326702758	5.337201557
5518	Fpr2	1.638203935	0.880691086	0.985652076
5519	Fra10ac1	7.309948012	7.353021055	7.43125863
5520	Fras1	6.209717365	6.332511952	6.406808653
5521	Frat1	6.649007155	6.820757051	6.638511628
5522	Frat2	6.288489102	6.546275615	5.650783424
5523	Frem1	2.417069148	4.123167708	3.790812634
5524	Frem2	3.52235631	2.416109989	2.982036031
5525	Frg1	8.510640584	8.399992991	8.467876788
5526	Frk	2.871443199	2.167040816	2.567727526
5527	Frmd3	3.985848799	3.521603859	5.270610887
5528	Frmd4a	7.472002124	7.219897778	7.397929657
5529	Frmd4b	6.421025836	6.635177333	6.188656687
5530	Frmd5	5.729002754	5.760742303	6.20918534
5531	Frmd6	6.695049107	6.604774612	6.887350112
5532	Frmd7	5.531734326	4.878530268	4.837323002
5533	Frmd8	7.102100674	7.334486241	6.799749349
5534	Frmpd1	4.672671118	4.733296895	4.979628097
5535	Frmpd1os	4.314169616	4.57330205	2.976816082
5536	Frmpd3	2.872189956	3.199757215	2.303318214
5537	Frmpd4	7.148028776	7.36169517	7.355052993
5538	Frrs1	2.103942135	3.867424376	3.441368016
5539	Frrs1l	12.39121967	12.33332084	12.45495175
5540	Frs2	6.570785462	6.344192653	6.70862061
5541	Frs3	4.016304636	4.619134627	5.624736432
5542	Fry	8.433673056	8.372734341	8.652608472
5543	Fryl	7.962349946	7.780144806	8.002607014
5544	Frzb	6.520116956	6.411941627	6.08816288
5545	Fsbp	4.181799865	4.63736289	5.655288321
5546	Fscb	1.075350458	3.717219343	1.983896513
5547	Fscn1	9.700564681	9.672611507	9.587318373
5548	Fscn2	1.04323123	1.497682098	1.574431131

7799	Lacc1	3.660436202	3.147985892	2.78365336510099
7800	Lace1	5.938779983	5.857703103	5.88665176210100
7801	Lactb	7.136369311	7.379305466	7.2747268110101
7802	Lactb2	4.475138093	4.936436634	4.88811034310102
7803	Lad1	2.412630508	2.634839524	3.30406334110103
7804	Lage3	7.462990582	7.246795729	7.31095861410104
7805	Lair1	6.145294841	5.922597261	6.4405347810105
7806	Lama1	3.975585216	3.29450376	1.97064604810106
7807	Lama2	5.538363091	5.597348614	5.37209017110107
7808	Lama3	3.231397177	3.038650895	1.56186133910108
7809	Lama4	4.600380468	3.498222803	5.15292638410109
7810	Lama5	3.523789776	3.021724008	3.15231044110110
7811	Lamb1	6.875230954	7.009398672	6.57916935510111
7812	Lamb2	6.872539953	6.356410331	6.77477767910112
7813	Lamb3	2.065699463	1.431605574	1.56672640710113
7814	Lamc1	6.084984326	6.134423985	6.53472726110114
7815	Lamc2	4.323196598	4.164016416	4.3738007210115
7816	Lamc3	3.077588158	3.671822751	3.79119689510116
7817	Lamp1	10.8758144	10.88287126	10.834839610117
7818	Lamp2	9.32611867	9.473208676	9.34631667810118
7819	Lamp3	2.39301592	3.591395515	1.98040475510119
7820	Lamp5	8.494275758	8.043742438	9.14787717910120
7821	Lamtor1	9.568470253	9.662979627	9.69941397310121
7822	Lamtor2	6.723272366	6.842005255	6.81335029610122
7823	Lamtor3	7.339205508	7.639983266	7.33723090310123
7824	Lamtor4	8.428667344	8.346176205	8.38523526110124
7825	Lamtor5	8.166781801	8.096931133	7.78072146110125
7826	Lancl1	9.279961208	9.28958837	9.2160289910126
7827	Lancl2	8.437016434	8.104042144	8.30655201610127
7828	Lancl3	5.532083615	4.461683363	5.15046672810128
7829	Lap3	8.642109144	8.352847959	8.60948156110129
7830	Laptm4a	11.25858703	11.29013843	11.0871796610130
7831	Laptm4b	9.354488502	9.465545436	9.35499313410131
7832	Laptm5	7.419388622	7.401757782	7.51954910710132
7833	Large	9.301351286	9.259086671	8.92831519610133
7834	Larp1	9.537759936	9.420819188	9.46783580110134
7835	Larp1b	6.508193649	7.093155109	6.77469477410135
7836	Larp4	8.858331746	8.6542026	8.80019567110136
7837	Larp4b	8.735064587	8.667393726	8.56859975710137
7838	Larp6	7.375694036	7.790036144	7.3106703510138
7839	Larp7	7.987514145	7.992575604	7.92240669710139
7840	Lars	8.588109148	8.655703745	8.5574473410140
7841	Lars2	5.579596983	5.710593222	6.37303711510141
7842	Las1l	8.298668789	8.037296817	8.13994459710142
7843	Lasp1	9.217326251	9.071203485	9.30426859510143
7844	Lat	1.040690241	1.921623065	0.98976296210144
7845	Lat2	4.471749326	4.852420879	4.37210617610145
7846	Lats1	6.998407845	6.915228187	6.98019333210146
7847	Lats2	8.167390091	8.165423724	7.80626201410147
7848	Layn	2.893220856	3.954562664	3.44092660510148

Pcdhb4	2.097225786	2.637400756	3.304311066
Pcdhb5	5.268608844	4.441734475	3.436853416
Pcdhb6	1.050275273	1.905590104	1.572874923
Pcdhb7	6.382060106	5.962039584	5.933442593
Pcdhb9	5.569711862	5.189323304	5.788710115
Pcdhga1	1.048014733	1.488673688	0.987886971
Pcdhga10	2.676171742	2.107702186	4.377515414
Pcdhga11	6.12552084	5.683647456	6.488617287
Pcdhga12	3.248148366	3.434005023	3.302728068
Pcdhga2	2.362882812	1.48864157	0.987882582
Pcdhga3	6.03306825	5.43996547	5.78697405
Pcdhga4	2.058281471	0.854621959	0.982622459
Pcdhga5	3.398159234	2.104308462	3.790146954
Pcdhga6	1.636478161	2.226164749	1.987799087
Pcdhga8	1.077178227	2.692501461	1.567060924
Pcdhga9	11.68003238	11.76896885	11.76870747
Pcdhgb5	2.900395828	3.653501789	4.305748352
Pcdhgb6	3.386418006	1.804327189	2.300725397
Pcdhgb8	3.747716742	0.819115383	2.985972926
Pcdhgc3	1.045667504	0.904810549	0.98848875
Pcdhgc4	1.052479734	1.480311985	1.988220712
Pcdhgc5	2.058296202	2.206016187	1.568931801
Pced1a	5.887033637	6.052851155	5.980484672
Pced1b	4.084328747	3.819067135	4.372769583
Pcf11	9.15752191	9.006790391	9.208798116
Pcgf1	3.53844858	4.3932121	3.148144641
Pcgf2	6.145003335	6.549639827	6.266120881
Pcgf3	6.36651582	6.775205365	6.519399402
Pcgf5	4.61329054	4.918950242	5.473183086
Pcgf6	5.831433939	5.798796082	5.534553253
Pcid2	4.095322701	4.528192507	5.506152637
Pcif1	5.750808767	6.173736205	5.958437859
Pck1	1.669107518	0.794708099	3.155176604
Pck2	6.308039928	6.282741449	6.169749197
Pclo	11.47229193	11.28532617	11.42241568
Pcm1	10.49534606	10.41586866	10.7448894
Pcmt1	9.017228389	9.025166678	9.286200592
Pcmtd1	10.73343218	10.64901889	10.93857234
Pcmtd2	8.455900721	8.696817161	8.591053794
Pcna	6.560908294	7.151795721	7.088530865
Pcnp	9.533874152	9.645491141	9.636445573
Pcnt	8.342157779	8.238837784	8.359500093
Pcnx	8.74187578	8.724283295	8.612989371
Pcnxl2	7.408605985	6.891012589	7.109431898
Pcnxl3	7.880622748	7.707986436	8.002705767
Pcnxl4	6.329699371	6.46768672	6.422880007
Pcolce	6.03157626	6.467715187	5.335908444
Pcolce2	6.212870607	6.496202599	6.319398008
Pcp2	7.829129175	8.233106956	8.199499217
Pcp4	11.51538732	11.82675687	12.17936796

12399	Serpinb1a	7.168133523	7.150244776	7.364004947
12400	Serpinb1b	3.546070247	4.380990646	2.974878285
12401	Serpinb6a	7.44694211	7.748069123	7.292600538
12402	Serpinb6b	2.903621239	3.751907424	3.681016155
12403	Serpinb6c	0.019295954	1.917346298	1.990025215
12404	Serpinb8	2.419793877	3.303616894	4.071595587
12405	Serpinb9	5.936817692	6.111900133	5.623222375
12406	Serpinc1	4.949596683	3.85282599	6.090007359
12407	Serpind1	3.784631802	4.3098944	4.229175695
12408	Serpine1	3.534363795	1.755108427	3.149307209
12409	Serpine2	10.54318729	10.36670378	10.76054297
12410	Serpine3	3.639566924	0.78365087	1.562465568
12411	Serpinf1	6.338441265	5.218430292	5.863452061
12412	Serpinf2	2.399799592	1.817263851	2.302026873
12413	Serping1	6.8594944	6.11312631	6.422488084
12414	Serpinh1	5.402680807	5.57000975	5.066052057
12415	Serpini1	9.793004233	9.64487713	9.891434994
12416	Sertad1	5.080234218	5.086478714	5.150261833
12417	Sertad2	8.124349401	8.188227114	7.945709201
12418	Sertad3	4.789102046	5.546714354	5.936483343
12419	Sertad4	6.542095038	6.562171661	7.07867779
12420	Sertm1	7.879381506	7.573120054	7.118861268
12421	Sesn1	7.779515995	7.573036149	7.354747753
12422	Sesn2	3.388453538	3.179500571	3.152317443
12423	Sesn3	7.31909031	7.321437132	7.602456559
12424	Sestd1	7.931920891	7.931305282	8.051684001
12425	Set	10.52655296	10.49519675	10.56120788
12426	Setbp1	8.406819852	8.419438284	8.418829078
12427	Setd1a	7.102078793	7.125155965	6.934055035
12428	Setd1b	8.356113921	8.230238843	8.451888673
12429	Setd2	9.189320478	9.337862775	9.352882121
12430	Setd3	9.255621069	9.251190406	9.348508586
12431	Setd4	3.889837332	4.089563026	4.229056603
12432	Setd5	8.797417115	8.527672107	8.538394845
12433	Setd6	6.287137795	6.067207663	6.10946617
12434	Setd7	9.083314096	9.004985651	9.062135994
12435	Setd8	9.02549253	8.806286381	8.757999833
12436	Setdb1	8.53212947	8.910900005	8.583568386
12437	Setdb2	3.26981097	3.113978016	4.937700886
12438	Setmar	4.66954433	4.688726968	4.886857118
12439	Setx	8.498895288	8.658129896	8.816172543
12440	Sez6	9.944254154	9.807004672	9.600184538
12441	Sez6l	8.906659432	8.774569647	8.948789256
12442	Sez6l2	10.33317643	10.21596271	10.29286891
12443	Sf1	10.10846667	10.24648812	10.0687608
12444	Sf3a1	7.930401815	7.599457052	7.850707209
12445	Sf3a2	5.082328219	5.36657796	5.682166033
12446	Sf3a3	7.080458788	7.066625045	6.991062311
12447	Sf3b1	9.594454931	9.723638766	9.771181686
12448	Sf3b2	11.0119423	10.88767915	10.83640081

14699	Tsix	2.649255843	1.841430553	2.304489036
14700	Tsks	1.660560454	3.710039358	1.565672211
14701	Tsku	2.064668546	1.861763877	3.157257201
14702	Tsn	10.52242638	10.56334995	10.48480484
14703	Tsnax	9.696934891	9.629094417	9.63999878
14704	Tsnaxip1	3.24806114	3.864189234	3.983801047
14705	Tspan1	1.043218422	2.243357404	0.989116008
14706	Tspan11	3.070968852	2.139139081	4.308730019
14707	Tspan12	5.864072614	5.965522226	6.18989832
14708	Tspan13	10.64838003	10.55387703	10.42704818
14709	Tspan14	7.759381609	7.658149964	7.463531124
14710	Tspan15	5.935933488	5.694623792	5.533859444
14711	Tspan17	7.610367178	7.595490595	6.967946163
14712	Tspan18	6.551831478	6.655514728	5.979497446
14713	Tspan2	9.049345531	9.378233274	8.988347186
14714	Tspan3	10.04922418	10.00136447	10.01889496
14715	Tspan31	6.571209581	6.581743764	6.001075992
14716	Tspan33	6.568179127	6.473357062	5.760043349
14717	Tspan4	5.936043615	5.769112401	5.787473403
14718	Tspan5	9.832298535	9.993999446	10.0767392
14719	Tspan6	6.523448715	6.724254168	6.422686696
14720	Tspan7	11.54660761	11.48348374	11.3225935
14721	Tspan8	1.056832533	0.880414171	1.571440636
14722	Tspan9	7.238862956	6.879765209	7.265813256
14723	Tspo	4.888309045	5.639867037	4.679394034
14724	Tspyl1	9.644703769	9.622404108	9.611149354
14725	Tspyl2	7.841368604	7.763228399	7.868942829
14726	Tspyl3	6.292922195	7.065463132	6.354487848
14727	Tspyl4	9.988113239	10.42060737	10.82002747
14728	Tspyl5	8.822387244	8.87912629	9.150037883
14729	Tsr1	7.340050007	7.970129174	7.579762673
14730	Tsr2	9.054353718	8.800540191	8.800139733
14731	Tsr3	7.801204896	7.833357506	7.899084134
14732	Tssc1	8.091388945	8.165637846	7.880699967
14733	Tssc4	7.829012182	8.120834074	7.780760155
14734	Tssk2	0.119773083	4.905540316	2.982112616
14735	Tssk3	1.636460806	2.72030283	0.986182844
14736	Tssk4	0.027312983	1.484363065	1.572866445
14737	Tssk6	2.415024195	4.849413791	0.965872767
14738	Tst	5.399518496	5.489770221	5.472814931
14739	Tsta3	6.169088376	6.599452021	6.066889367
14740	Tstd1	2.874413358	2.159135867	2.983703639
14741	Tstd2	7.338863429	7.640650303	7.455686768
14742	Tstd3	7.736835821	7.4151344	7.616904604
14743	Ttbk1	7.078751497	7.11792535	7.013717378
14744	Ttbk2	9.377579687	9.112671684	9.553692737
14745	Ttc1	8.897453282	8.834841067	8.841054609
14746	Ttc12	4.403455975	5.626451135	4.226802971
14747	Ttc13	6.709993954	6.873891747	6.51867064
14748	Ttc14	8.300738746	8.287063149	8.751617556

949	Actn3	1.050326958	0.894676918	0.987293505	3249	Cldn5	5.569324019	5.485196552	5.624775215	5549	Fsd1	6.782445826	6.716615047	7.228449615	7849	Lbh	7.908653959	8.259992452	8.55770768310149	Pcp4l1	8.700052335	8.989581362	9.361676517	12449	Sf3b3	7.280732026	7.49786367	7.169493054	14749	Ttc16	5.958885134	5.866131814	5.623806283
950	Actn4	9.644578041	9.789832384	9.623864127	3250	Cldn9	2.65164911	3.802979573	1.563424738	5550	Fsd1l	6.540596287	6.818956967	6.638284106	7850	Lbhd1	7.124438598	7.056960311	6.99107904510150	Pcsk1	5.665489885	5.617917449	6.089837203	12450	Sf3b4	6.874806872	7.040428142	6.666064792	14750	Ttc17	6.506685557	6.451706435	6.50357208
951	Actr10	9.625499695	9.717764821	9.636420909	3251	Cldnd1	8.910267254	8.898494712	8.865654769	5551	Fsip1	2.076765175	3.046075186	2.303779561	7851	Lbp	5.435129255	5.943717928	4.67794996510151	Pcsk1n	10.58349068	10.68801475	10.83254444	12451	Sf3b5	8.204819649	8.314162188	8.288375927	14751	Ttc19	8.864451151	8.635521586	8.648884699
952	Actr1a	8.971651479	8.858097266	8.659403757	3252	Clec10a	3.655531655	2.10714465	3.682967702	5552	Fst	3.279768488	5.218773441	4.503716465	7852	Lbr	6.10579727	6.439472383	6.2464810610152	Pcsk2	10.15757973	10.3741726	10.60485154	12452	Sf3b6	9.275288451	9.232165382	9.288338277	14752	Ttc21a	2.063380831	1.437615503	2.793864594
953	Actr1b	8.798463122	8.860116798	9.083411035	3253	Clec11a	4.081701873	4.715290977	4.505185075	5553	Fstl1	8.041056105	8.290179563	7.957049704	7853	Lca5	5.666820862	5.719369793	5.93473604210153	Pcsk2os1	5.326489715	5.266035502	4.933071273	12453	Sfi1	6.542460798	6.815356883	6.693993144	14753	Ttc21b	4.680369699	5.450856561	5.888129961
954	Actr2	10.60231577	10.77003435	10.77683585	3254	Clec12a	2.068027012	2.457752709	1.566028234	5554	Fstl3	4.770979633	4.157948999	3.978863489	7854	Lca5l	2.906610069	4.250723331	4.15211837110154	Pcsk2os2	0.064891518	2.461929999	2.306341291	12454	Sfmbt1	7.412791656	7.494571536	7.850663484	14754	Ttc22	1.043235034	0.910078671	0.989111756
955	Actr3	9.361467828	9.465509509	9.35063436	3255	Clec14a	4.590447776	4.505415547	3.787167668	5555	Fstl4	6.05797132	6.006259489	6.456774626	7855	Lcat	6.148493267	6.346408711	6.43976182610155	Pcsk4	4.152308335	3.297112772	3.78970496	12455	Sfmbt2	3.671212139	4.669976866	3.145265259	14755	Ttc23	3.246681457	3.55663647	3.303089324
956	Actr3b	4.089901402	4.800593849	4.788388901	3256	Clec16a	7.080436322	7.241238381	7.024300199	5556	Fstl5	8.27464481	8.026839164	8.002219555	7856	Lck	2.66237	3.685205814	3.79276499510156	Pcsk5	6.205992738	6.128800702	5.502205653	12456	Sfn	3.229395135	3.043086015	2.56751457	14756	Ttc25	2.666938006	3.876958573	2.563993538
957	Actr5	4.660280873	4.360849381	4.504145999	3257	Clec18a	1.058806472	1.468523105	0.98511049	5557	Fth1	13.31737683	13.47575561	13.29398803	7857	Lclat1	6.3481404	6.422801217	6.68105596310157	Pcsk6	6.874813949	7.03041388	6.94595558	12457	Sfpq	9.791432837	9.678141204	9.831733871	14757	Ttc26	5.861546715	5.860844897	6.024970107
958	Actr6	7.1378545	7.45293458	7.715137637	3258	Clec1a	3.971377094	3.555505177	2.561844053	5558	Ftl1	9.689354421	9.536723726	9.385078367	7858	Lcmt1	8.887160507	8.582255519	8.66299215810158	Pcsk7	4.941027903	5.401685907	5.109356796	12458	Sfr1	10.89748532	10.74384559	10.71551923	14758	Ttc27	5.634219422	5.773910004	5.471483352
959	Actr8	7.642449088	7.516526117	7.673601567	3259	Clec2d	1.077058676	2.890281991	1.983580393	5559	Fto	8.981406674	9.045200896	9.101599926	7859	Lcmt2	5.291024098	5.153484406	5.26503746210159	Pcsk9	1.688446809	3.02172629	3.792293014	12459	Sfrp1	6.706885358	7.058775213	6.422974119	14759	Ttc28	7.867384669	7.810814891	7.974733118
960	Actrt2	1.649469763	3.367282053	1.984857776	3260	Clec2l	7.067637844	6.754651005	7.002612904	5560	Ftsj1	6.70772028	6.749578636	6.148998586	7860	Lcn2	3.65059995	2.12467163	1.9746479710160	Pctp	3.243165462	3.313504386	2.563158148	12460	Sfrp2	4.245270742	3.630539068	4.374119027	14760	Ttc29	1.060753747	2.485745692	0.984607529
961	Actrt3	3.076503035	4.047438542	2.298510114	3261	Clec3b	2.893247394	3.767939727	2.785371046	5561	Ftsj2	4.893762476	5.708873824	5.653902701	7861	Lcor	6.417352398	6.196508218	6.08834419610161	Pcx	6.013778415	6.21271792	6.488490772	12461	Sfrp4	1.660536894	2.184255648	2.569546406	14761	Ttc3	13.86869707	13.60197048	13.74970952
962	Acvr1	7.269594665	7.348147732	7.337844838	3262	Clec4a2	3.072599912	4.05338534	3.442935259	5562	Ftsj3	8.285146959	8.225353332	8.292872079	7862	Lcorl	8.308474746	8.118743443	8.17482112710162	Pcyox1	8.285424679	8.357459652	8.475949001	12462	Sfrp5	4.014526132	3.674614625	5.472527813	14762	Ttc30a1	4.828704548	4.421817446	4.788816957
963	Acvr1b	3.081083052	4.338401913	3.441046836	3263	Clec4a3	2.088800984	3.576324782	3.15306213	5563	Ftx	4.730475359	4.196516354	5.505160446	7863	Lcp1	6.003012836	4.81904454	4.36750698610163	Pcyox1l	7.035268221	7.308358461	7.045971352	12463	Sfswap	8.219586875	8.298872354	8.051390946	14763	Ttc30b	5.939638815	6.46184825	6.284499666
964	Acvr1c	7.86202651	7.904127255	7.991345265	3264	Clec4a4	0.019311678	0.910062138	0.989109799	5564	Fubp1	9.32854181	9.311368057	9.292836541	7864	Lcp2	2.66593022	3.020196152	2.98141727510164	Pcyt1a	7.658272837	7.763230425	7.52662015	12464	Sft2d1	5.755552182	6.0091928	5.957293458	14764	Ttc32	5.857140832	5.450837386	6.026038633
965	Acvr2a	8.345942195	8.01762333	8.209001055	3265	Clec5a	5.274144177	4.364382944	4.566229613	5565	Fubp3	6.059420155	6.815679431	6.320190157	7865	Lct	5.37638075	6.19746452	6.20910541510165	Pcyt1b	8.057115667	7.743059267	8.056975025	12465	Sft2d2	7.230376817	7.417593438	7.023979341	14765	Ttc33	8.987366397	8.946304296	8.883818354
966	Acvr2b	4.934651138	4.695201492	5.229962222	3266	Clec7a	2.660188057	3.886360033	0.971716969	5566	Fuca1	7.893925406	7.915631069	7.518731835	7866	Lctl	1.068347541	2.206140839	1.56895033610166	Pcyt2	6.860331153	6.840475014	6.471318254	12466	Sft2d3	7.113360413	7.389697934	7.150128723	14766	Ttc34	3.867550626	3.67910955	2.29956895
967	Acvrl1	4.40252821	4.292883438	4.301412278	3267	Clec9a	1.650933922	1.444157701	2.571321586	5567	Fuca2	6.888858276	6.945484039	7.046163267	7867	Ldah	7.723773182	7.599209784	7.70120792610167	Pdap1	11.09728062	11.09891706	10.94720648	12467	Sfxn1	9.716851074	9.576069738	9.551745748	14767	Ttc37	5.431276241	4.96625648	4.886435411
968	Acy1	5.663011499	5.539660483	5.863877778	3268	Clgn	6.170064942	6.542090523	6.550352293	5568	Fuk	4.993118037	5.654075013	5.406529404	7868	Ldb1	7.829931497	8.137630819	8.32455317710168	Pdcd10	8.123661519	7.725182154	7.87481821	12468	Sfxn2	5.075810935	4.929719572	4.838157736	14768	Ttc38	5.396784952	5.006900166	5.024349559
969	Acy3	3.539356896	4.183765376	2.976913966	3269	Clhc1	1.064593102	0.863192536	1.98590804	5569	Fundc1	7.875549748	7.736246164	8.056878444	7869	Ldb2	8.769779735	8.582954177	8.66308302810169	Pdcd11	6.456039473	6.749323851	6.389096124	12469	Sfxn3	7.841605196	8.045297011	7.825576306	14769	Ttc39a	3.087235649	4.260614857	2.559906439
970	Acyp1	7.249427323	7.427119154	6.693368966	3270	Clic1	5.776336956	5.728076803	5.372031993	5570	Fundc2	9.494033639	9.313156946	9.443522421	7870	Ldb3	4.669792436	4.925913417	5.19057085810170	Pdcd2	5.293286079	5.38904478	5.188218415	12470	Sfxn4	5.660991099	5.226395713	5.265065666	14770	Ttc39aos1	3.995152121	4.361052973	4.839975369
971	Acyp2	5.790171645	6.373495676	6.748633856	3271	Clic3	1.64797805	2.206087099	2.794876263	5571	Fuom	6.796781463	7.15255497	7.002302667	7871	Ldha	9.40443913	9.408263285	9.34416121510171	Pdcd2l	6.344144675	5.964670666	6.302576895	12471	Sfxn5	10.26290913	10.11486361	10.28378298	14771	Ttc39b	8.081093043	8.136511856	8.527196166
972	Ada	1.083820114	1.851408991	2.305516847	3272	Clic4	9.220371088	9.363458414	9.391524283	5572	Furin	7.315594715	7.079201174	6.623251028	7872	Ldhal6b	1.064593435	3.372798632	0.98361380710172	Pdcd4	9.73698488	9.773109224	9.862677616	12472	Sgcb	7.76728857	7.768149505	7.767833551	14772	Ttc39c	6.382763141	6.042783078	5.909731541
973	Adad1	0.02988116	2.723054655	0.986741959	3273	Clic5	4.733928217	5.364231057	5.595875792	5573	Fus	11.76034813	11.73021408	11.50305826	7873	Ldhb	10.12383929	10.26394474	10.3495865810173	Pdcd5	7.874873804	7.920610543	7.880884121	12473	Sgcd	0.01660218	1.921603763	0.98976004	14773	Ttc39d	1.058798132	1.891577779	1.987007705
974	Adal	6.267996617	6.251352401	6.170114755	3274	Clic6	6.975925278	7.941812009	5.809646732	5574	Fut10	5.323345781	5.127189417	4.933906557	7874	Ldhd	4.460708164	3.4059918	4.37492835810174	Pdcd6	7.938505045	8.003404883	7.910697272	12474	Sgce	6.555224826	6.49691697	6.066377777	14774	Ttc4	7.900488036	8.125768472	7.874831259
975	Adam10	8.544015694	8.56259543	8.534582618	3275	Clint1	9.007924957	8.811974993	8.84725405	5575	Fut11	5.694853215	5.91008163	6.068709077	7875	Ldlr	6.740146453	6.825379838	7.15041638110175	Pdcd6ip	8.502611725	8.291688448	8.656074846	12475	Sgcg	1.040703565	0.915548276	1.574993885	14775	Ttc41	1.653960792	1.43736267	0.980741068
976	Adam11	8.401014111	8.379578288	7.874581832	3276	Clip1	10.31637885	10.15976604	10.16981367	5576	Fut2	3.226625709	2.445643297	2.984996138	7876	Ldlrad3	5.833412042	5.974331464	5.37116416110176	Pdcd7	7.752123588	7.544496188	7.623868949	12476	Sgcz	4.249759634	3.724069004	4.625651678	14776	Ttc5	6.270348212	6.175657457	6.302006933
977	Adam12	3.525347662	2.841001919	2.563931936	3277	Clip2	6.554632947	6.438599301	6.472034507	5577	Fut4	1.038051171	1.507511375	1.575587067	7877	Ldlrad4	6.166508432	5.965287732	6.067526210177	Pdcl	6.751912881	6.46385147	6.991978955	12477	Sgip1	9.086161659	9.071424753	9.213686181	14777	Ttc7	5.028968671	4.795605328	4.147225755
978	Adam15	8.299639024	8.536684286	8.324326942	3278	Clip3	11.14597066	11.25951834	11.09827124	5578	Fut8	7.540785959	7.805008122	7.652381347	7878	Ldlrap1	5.032004933	5.529424655	3.4308997710178	Pdcl2	1.117140654	4.904073638	2.788225633	12478	Sgk1	9.151529501	8.404446712	8.363234989	14778	Ttc7b	8.254696109	8.095367984	8.159984384
979	Adam17	7.04603549	7.047882306	7.07815334	3279	Clip4	4.597042489	3.999316092	4.302348671	5579	Fut9	10.87345351	10.88409878	11.29291443	7879	Ldoc1	3.397733662	3.000044886	3.79024135410179	Pdcl3	6.062824141	6.374474141	6.246101762	12479	Sgk2	4.607254452	5.532250461	4.502278253	14779	Ttc8	5.69001991	5.394351199	5.265151012
980	Adam18	0.027287919	1.905561485	0.987302342	3280	Clk1	7.104503103	6.875786843	7.355086731	5580	Fuz	7.28106787	7.854216956	7.587604421	7880	Ldoc1l	7.504973972	7.34709757	7.11932357210180	Pddc1	6.010872255	5.987798522	6.046281551	12480	Sgk3	6.16912275	6.837376818	5.909666508	14780	Ttc9	5.541284802	5.888784809	5.761783483
981	Adam19	5.935754222	6.170340307	5.863075914	3281	Clk2	6.271008232	6.210355753	6.735874915	5581	Fxn	5.165350136	5.496019772	5.067709325	7881	Leap2	0.045262968	1.457736305	2.98853561910181	Pde10a	7.817630152	8.430943932	8.778027905	12481	Sgms1	7.752898095	7.855850192	7.707865399	14781	Ttc9b	9.063048527	9.17469983	9.32899493
982	Adam1a	3.077694652	3.441427049	3.683834013	3282	Clk3	7.700753106	7.40771276	7.310745588	5582	Fxr1	9.531501901	9.400081876	9.467859666	7882	Lect1	3.078629175	3.670048969	3.4415931310182	Pde11a	3.06787116	3.886298262	3.153344129	12482	Sgms2	5.435395546	5.758289469	4.435591988	14782	Ttc9c	8.102185286	7.705799143	8.013485251
983	Adam22	9.537894296	9.413001807	9.731625913	3283	Clk4	7.251868783	7.137446363	7.616858226	5583	Fxr2	9.45772518	9.56110211	9.443513745	7883	Lect2	1.634629488	0.889952045	2.31080347810183	Pde12	5.779519195	5.644403348	5.438577513	12483	Sgol1	1.080618395	2.688367892	1.566328887	14783	Ttf1	7.410637664	7.255214144	7.119231854
984	Adam23	8.125695766	8.255339856	8.816489181	3284	Clmn	9.876282549	9.999908388	9.3849929	5584	Fxyd1	9.035096104	9.504547355	9.101477418	7884	Lef1	5.546982938	5.466103685	6.50477105510184	Pde1a	11.04962465	10.74861347	10.71216291	12484	Sgol2a	4.254759509	4.896208697	4.624517415	14784	Ttf2	3.253698316	4.187469253	3.148508318
985	Adam24	0.005608334	0.940318714	0.992698372	3285	Clmp	7.588512877	7.592464544	7.638230549	5585	Fxyd2	3.902175276	5.215566178	4.064920667	7885	Lefty1	5.211546063	5.227326696	5.88879102310185	Pde1b	9.20043295	9.617348333	9.926989466	12485	Sgol2b	0.027277083	1.905576749	0.987304708	14785	Tti1	7.308674683	7.433213227	6.956781942
986	Adam32	2.385371218	1.853906506	0.979009046	3286	Cln3	5.695550432	5.745449277	5.734934434	5586	Fxyd3	5.887205309	6.29121853	5.734544615	7886	Lefty2	4.217971997	2.666733422	1.97959065910186	Pde1c	7.088621409	6.769381455	6.388562608	12486	Sgpl1	6.794123704	6.928790857	6.922937729	14786	Tti2	7.112073674	6.871079022	7.078413512
987	Adam33	2.650006082	1.408111785	3.155519411	3287	Cln5	6.189174638	6.248313896	5.909856908	5587	Fxyd5	5.68930315	5.363890699	5.565987098	7887	Lekr1	5.93867618	5.924659177	5.62275107710187	Pde2a	10.16901455	10.08617157	10.17112541	12487	Sgpp1	7.635058872	7.530288932	7.701199481	14787	Ttk	1.66690033	2.17321821	2.304260694
988	Adam4	1.050290916	0.894755545	2.311187695	3288	Cln6	3.785465185	4.720090969	4.441411584	5588	Fxyd6	9.458823844	8.953953614	8.904736385	7888	Lelp1	1.102740328	5.001171281	1.56166330610188	Pde3a	1.083800616	1.422270955	2.305520037	12488	Sgpp2	5.758027288	5.550438425	6.709341607	14788	Ttl	8.983927794	8.803599755	9.040842479
989	Adam5	2.381061807	2.466244253	1.983871199	3289	Cln8	7.219968936	7.323305649	7.328936967	5589	Fxyd7	8.108136985	8.303577941	8.279455393	7889	Lemd2	6.553506411	6.529106889	6.31991261710189	Pde3b	6.437396311	6.498572328	6.472098172	12489	Sgsh	4.972942476	4.084637497	4.440922106	14789	Ttll1	5.695025632	5.644242416	6.003383216
990	Adam8	2.390268957	2.447501323	1.981204785	3290	Clns1a	8.897518982	8.884934848	8.790487631	5590	Fyb	5.037013093	5.334464832	5.65412519	7890	Lemd3	8.314013822	8.264020614	8.50768412610190	Pde4a	9.191239023	9.005215678	9.005120365	12490	Sgsm1	8.307679146	7.79056817	8.093906352	14790	Ttll11	6.807076493	6.605058192	6.66650836
991	Adam9	5.048182162	6.051057992	6.247813245	3291	Clock	9.28517614	9.231988693	9.322068824	5591	Fyco1	7.96397713	8.070954589	8.242199964	7891	Lenep	4.933871892	4.533492125	4.88782125910191	Pde4b	10.06059365	10.06565824	10.0976661	12491	Sgsm2	8.161465873	7.870926052	7.974423301	14791	Ttll12	6.161465433	5.286813587	5.371302456
992	Adamts1	6.193114102	6.735803219	6.336962846	3292	Clp1	4.896615324	5.778522391	5.227226417	5592	Fyn	9.562152605	9.552427633	9.422912505	7892	Leng1	6.145819022	6.28534308	5.97969025910192	Pde4c	3.225207946	2.177507789	1.56466248	12492	Sgsm3	7.080954072	7.299598865	7.098864863	14792	Ttll13	1.652447577	3.067895362	2.307172646
993	Adamts10	3.421760508	4.079103569	4.682326191	3293	Clpb	7.539657975	7.830343637	7.659722628	5593	Fyttd1	9.277706505	9.177381815	9.423022134	7893	Leng8	4.605850366	4.795140167	4.50263540210193	Pde4d	8.745821356	8.628428857	9.008096134	12493	Sgta	9.450931651	9.379235617	9.12963642	14793	Ttll3	7.586665492	7.431574359	7.237212478
994	Adamts12	1.656680427	1.431230101	3.573215109	3294	Clpp	8.183264311	7.929445982	7.850105695	5594	Fzd1	4.838865235	5.087369266	4.837123282	7894	Leng9	4.236608026	3.755068802	3.98233358110194	Pde4dip	9.282492625	9.097113685	9.253811129	12494	Sgtb	9.702416537	9.606059585	9.789032176	14794	Ttll4	3.0551669	1.851377452	1.982333649
995	Adamts13	3.512576584	2.873988067	1.563985127	3295	Clptm1	8.4292849	8.507262983	8.427067289	5595	Fzd10	1.691328267	3.678245809	3.684633801	7895	Leo1	7.000477368	7.132434204	7.24697782310195	Pde5a	5.864325877	6.216550787	6.390313924	12495	Sh2b1	7.579998831	7.830000325	7.813302031	14795	Ttll5	7.716110261	7.572946976	7.54223438
996	Adamts15	3.425810384	3.904650483	4.736292253	3296	Clptm1l	8.27032969	8.486995325	8.260759632	5596	Fzd2	6.874646809	6.546895777	6.707618853	7896	Lepr	4.080940336	4.234958862	4.50535493310196	Pde6d	7.524065729	7.840228918	7.594664396	12496	Sh2b2	5.237210332	4.422977882	4.439530082	14796	Ttll6	3.057521074	2.177429702	1.981476894
997	Adamts16	2.098052147	1.343461375	3.791608325	3297	Clpx	6.523701967	6.45072402	6.721871504	5597	Fzd3	10.20325632	9.980912208	10.27924769	7897	Leprot	7.057781629	7.258669011	6.99109544710197	Pde6g	0.057884884	2.893898816	1.567767481	12497	Sh2b3	5.631665257	5.678504613	5.066143893	14797	Ttll7	10.7441825	10.75659633	10.91000069
998	Adamts17	3.766928693	3.438567062	2.786035957	3298	Clrn1	3.387453944	3.572918031	3.68522338	5598	Fzd4	3.550696727	4.769326023	4.303165869	7898	Leprotl1	8.688104979	8.698581927	8.87488961910198	Pde7a	7.229834864	7.273940699	7.189479754	12498	Sh2d3c	5.834303231	5.694321767	6.046999109	14798	Ttll8	1.641712831	1.464680377	2.573040145

999	Adamts2	2.388354723	2.451405302	2.985577777
1000	Adamts20	5.83100728	5.751121995	5.735643719
1001	Adamts3	5.397006878	5.434197261	5.505246052
1002	Adamts4	5.02421016	5.028035302	4.736340051
1003	Adamts5	3.905209613	4.281124749	5.374867312
1004	Adamts6	2.66107978	3.186405504	2.78898785
1005	Adamts8	3.255291634	4.027912219	2.977135763
1006	Adamts9	3.657849294	3.297823438	3.789779178
1007	Adamtsl1	6.451494328	6.367453717	5.679109296
1008	Adamtsl4	5.830192619	5.482517244	5.681221138
1009	Adamtsl5	3.761743879	1.354597563	3.985472862
1010	Adap1	8.766860127	9.044291571	8.73823511
1011	Adap2	4.824388463	4.492404941	3.4351633
1012	Adar	7.673437467	7.732491059	7.754782271
1013	Adarb1	8.24198764	8.480468513	8.890041781
1014	Adarb2	4.408275251	4.531808244	4.502595434
1015	Adat1	3.886278303	4.315393156	3.563649039
1016	Adat2	6.268358077	5.760285244	5.811989042
1017	Adat3	2.054203653	1.461070993	1.570141543
1018	Adck1	6.346047927	6.395863215	6.108919344
1019	Adck2	5.915914134	6.045736382	6.228243194
1020	Adck3	8.727418094	8.645800731	8.38498145
1021	Adck4	5.403412117	5.650213043	5.762358172
1022	Adck5	6.211063088	6.570743225	6.439733902
1023	Adcy1	11.37812144	11.48481496	11.60342084
1024	Adcy2	8.47362998	8.625308	8.87796696
1025	Adcy3	6.848343548	6.815298379	6.922752081
1026	Adcy4	3.757375543	2.4241959	2.565903798
1027	Adcy5	7.439199858	7.645547838	7.84449031
1028	Adcy6	5.981580688	5.355990695	5.188080599
1029	Adcy7	2.907072249	3.278891892	4.50708646
1030	Adcy8	4.099709599	4.831491335	5.566951216
1031	Adcy9	8.944559972	8.967580655	8.931305866
1032	Adcyap1	4.338206989	4.638256003	5.068403256
1033	Adcyap1r1	9.772057309	9.504512275	9.680446405
1034	Add1	10.00686848	10.08474965	10.04338968
1035	Add2	10.65701021	10.57669261	10.63462462
1036	Add3	10.43588927	10.48812967	10.38192277
1037	Adgb	3.063989585	3.195609053	2.790264048
1038	Adgra1	8.704533604	8.060287099	8.154813523
1039	Adgra2	6.247324844	6.235852751	5.837506438
1040	Adgra3	3.791555312	4.547583142	5.027424878
1041	Adgrb1	8.162883901	8.186425227	8.324404232
1042	Adgrb2	7.750673676	7.581728426	7.406289773
1043	Adgrb3	8.280752885	8.434172606	8.487847837
1044	Adgrd1	3.105767753	4.161033572	5.306465971
1045	Adgre1	4.663760713	4.750867491	4.735600343
1046	Adgre4	0.008387076	0.933562442	0.991896942
1047	Adgre5	5.437163379	5.18968378	5.439652272
1048	Adgrf4	2.872142599	2.438279163	2.984254538

3299	Clspn	2.686798936	4.324629493	3.788541135
3300	Clstn1	11.18771288	11.07262508	11.29686483
3301	Clstn2	8.489312073	8.553710011	8.164699705
3302	Clstn3	8.872264027	9.081037362	9.109289573
3303	Clta	10.8370395	10.79791647	10.64529982
3304	Cltb	9.455634869	9.372728234	9.613045109
3305	Cltc	11.38664912	11.2909513	11.52785569
3306	Clu	12.25396001	12.31960437	12.06111245
3307	Cluap1	7.841670574	7.726209467	7.856627463
3308	Cluh	9.068840399	8.949118189	8.960151704
3309	Clvs1	7.489863765	7.429713265	7.735253858
3310	Clvs2	7.301464793	7.83510659	7.579918357
3311	Clybl	8.084722168	7.88306868	7.741461007
3312	Cma1	0.005608334	0.940318714	0.992698372
3313	Cmas	10.02450465	9.976042971	10.00512688
3314	Cmbl	6.360379272	5.716039582	5.910795062
3315	Cmc1	7.166386884	7.018609328	7.170211253
3316	Cmc2	6.998871472	6.9143386	7.099254313
3317	Cmc4	5.160594703	4.745870047	4.787675699
3318	Cmip	11.74980775	11.60645313	11.53312877
3319	Cmklr1	3.980109617	4.103907738	3.147327731
3320	Cml1	6.057760969	6.368259244	6.002203023
3321	Cml2	2.119435621	3.850230794	4.305401651
3322	Cml3	2.39659059	1.391752517	2.790454481
3323	Cml5	3.227366113	1.405096408	2.79142693
3324	Cmpk1	7.508689509	8.034509059	7.767802982
3325	Cmpk2	6.540924561	6.290440113	6.957972057
3326	Cmss1	5.633450361	5.914139012	5.813874114
3327	Cmtm2a	0.052753389	2.898120495	2.307779054
3328	Cmtm2b	2.063355629	3.490308421	0.980786029
3329	Cmtm3	4.250466138	4.229163805	4.06652951
3330	Cmtm4	7.986142571	7.563823457	7.63088526
3331	Cmtm5	8.672108721	8.415227516	8.265240822
3332	Cmtm6	6.635218463	6.550347735	6.934823181
3333	Cmtm7	1.644897543	2.480490207	0.983590921
3334	Cmtm8	5.325269987	5.268490767	4.562413451
3335	Cmtr2	4.937167967	5.310979628	4.886994433
3336	Cmya5	5.252482938	5.260478077	5.788736418
3337	Cnbd2	4.938523483	5.341795662	4.733976997
3338	Cnbp	9.846951662	9.79648985	9.748164162
3339	Cndp1	1.647976871	2.475575978	2.308079417
3340	Cndp2	6.269190612	6.517500665	6.067158704
3341	Cnep1r1	7.817208217	8.074954051	8.511636881
3342	Cnfn	0.013880299	1.507505947	1.575586429
3343	Cnga4	2.382154551	2.692485605	0.98033365
3344	Cngb3	0.016596698	0.915563672	0.989761378
3345	Cnih1	8.717293073	8.806813993	8.774418075
3346	Cnih2	11.59972317	11.85691359	11.31701007
3347	Cnih3	7.195726929	6.184269072	6.813022731
3348	Cnih4	8.716844458	8.635627989	8.663019297

5599	Fzd5	2.080704054	2.161121813	2.983884323
5600	Fzd6	4.17118874	4.545762197	4.227410458
5601	Fzd7	5.249218712	5.196099552	5.625470022
5602	Fzd8	4.527742214	2.785386004	4.938612618
5603	Fzd9	2.674971466	3.666577706	4.071777638
5604	Fzr1	8.323457471	8.259922205	8.431366039
5605	G0s2	5.601959307	5.358563856	5.107903951
5606	G2e3	7.169177807	7.446661589	7.464022238
5607	G3bp1	8.877597226	8.763154568	8.623798883
5608	G3bp2	10.91898258	10.78086476	10.8861167
5609	G530011O06Rik	1.094527553	2.441810617	3.15514894
5610	G630025P09Rik	3.049151742	0.846415279	2.571308124
5611	G630093K05Rik	0.027248348	0.894825392	0.987310984
5612	G6pc3	7.479407026	7.256595194	7.169772352
5613	G6pdx	6.166576161	5.986122199	6.320605942
5614	Gaa	8.47745944	8.509926685	8.731668537
5615	Gab1	7.907548066	8.316276559	8.124790236
5616	Gab2	7.356043413	7.417238379	7.179504911
5617	Gab3	5.170502867	5.29636071	5.681675559
5618	Gabarap	9.496189293	9.5848751	9.337486246
5619	Gabarapl1	11.70124277	11.66791469	11.63237343
5620	Gabarapl2	9.311591118	9.247305818	9.127132706
5621	Gabbr1	10.9055761	11.02462446	11.01616507
5622	Gabbr2	9.091755877	9.184833498	9.33548449
5623	Gabpa	5.69458143	5.645123096	6.151467927
5624	Gabpb1	4.902298564	6.074116937	5.813034446
5625	Gabpb2	6.189658096	6.022715863	6.639213473
5626	Gabra1	11.56096895	11.60954827	12.02883121
5627	Gabra2	9.11581517	8.83809323	8.542034513
5628	Gabra3	9.354137837	8.923775216	9.64551672
5629	Gabra4	9.576667726	9.749313	9.831778352
5630	Gabra5	9.922598533	9.856419234	9.509322347
5631	Gabrb1	9.595713069	9.40418522	9.075241725
5632	Gabrb2	10.91275546	10.90642328	11.13671587
5633	Gabrb3	9.957015424	9.771110261	9.859548344
5634	Gabrd	6.19156615	6.327935069	6.694917395
5635	Gabrg1	7.455345111	7.183480653	7.319675054
5636	Gabrg2	10.13040317	9.958946738	10.26427295
5637	Gabrg3	3.079117225	3.868264001	2.786074768
5638	Gabrq	2.641772622	1.861931476	0.980332646
5639	Gabrr1	0.083359035	2.445879332	3.155536449
5640	Gabrr2	2.419843772	2.823512455	3.790376652
5641	Gad1	10.20996942	10.09117306	10.47187962
5642	Gad2	9.502985795	9.322336329	9.906360746
5643	Gadd45a	4.943083418	5.547865883	5.108863828
5644	Gadd45b	5.40110362	5.154876665	5.337634028
5645	Gadd45g	7.011184141	7.028117557	6.578982401
5646	Gadd45gip1	8.643528143	8.794455203	8.898860089
5647	Gak	8.269612958	8.187332299	8.179749371
5648	Gal	2.07574562	3.047594993	1.563695593

7899	Letm1	8.805161665	8.817879173	8.96306786610199
7900	Letm2	5.83384925	5.863927593	5.30038006610200
7901	Letmd1	7.693354694	7.601931471	7.36364490510201
7902	Lfng	6.420750504	6.153395588	6.24622016110202
7903	Lgals1	6.231545891	6.925340378	6.28360931810203
7904	Lgals2	5.077292962	5.092352671	4.78692381410204
7905	Lgals3	3.783650504	5.123538576	3.43680079910205
7906	Lgals3bp	5.889285382	6.591771258	6.08878587110206
7907	Lgals8	7.869506464	8.155854212	7.89265172610207
7908	Lgals9	5.367990568	5.622467929	5.7356723610208
7909	Lgalsl	8.735355268	8.789129254	8.86573853210209
7910	Lgi1	8.634566278	8.643102053	8.3850488910210
7911	Lgi2	6.331122054	6.52322046	6.89956295810211
7912	Lgi3	7.829736398	8.179000861	7.97993150910212
7913	Lgi4	6.147596684	6.558743531	6.33783381810213
7914	Lgmn	7.651850417	8.129779829	7.91071502910214
7915	Lgr4	6.419271586	6.17462534	6.40649109410215
7916	Lgr5	3.661327443	4.327446848	2.78335289510216
7917	Lhb	2.069248981	2.684609085	2.305525610217
7918	Lhfp	5.300793445	6.106289109	6.35592892510218
7919	Lhfpl1	2.07252951	3.208571908	1.98149420210219
7920	Lhfpl2	6.573814096	6.873888773	6.80034567210220
7921	Lhfpl3	6.235768877	6.629896652	7.05741628510221
7922	Lhfpl4	9.344317848	9.054546503	9.09894963110222
7923	Lhfpl5	4.772188772	3.249568287	4.30204740610223
7924	Lhpp	5.393163052	5.055464374	5.06854715410224
7925	Lhx1	3.098314692	3.837472376	4.56811232310225
7926	Lhx1os	2.907551749	3.530475	3.78771588110226
7927	Lhx2	9.172519695	9.025955186	8.77107256710227
7928	Lhx3	2.046561885	0.889906211	0.98673245610228
7929	Lhx5	2.693562833	3.527495564	4.37498117810229
7930	Lhx6	5.109979421	4.49204367	4.44052222110230
7931	Lhx8	1.636438944	0.88527979	0.98618948510231
7932	Lhx9	6.738860314	6.804565343	6.72139073710232
7933	Lias	8.627406586	8.592588149	8.82877581810233
7934	Lifr	8.351292661	8.103999149	8.18945038610234
7935	Lig1	7.260654008	7.447230981	7.38102388110235
7936	Lig3	6.910620733	6.818906916	6.51893748110236
7937	Lig4	7.044251819	6.430929936	6.82553058510237
7938	Lilra5	0.016591713	1.502490189	1.99052616710238
7939	Lilrb4a	1.638251033	1.894905765	2.31006192210239
7940	Lima1	7.034447172	7.334528023	7.08868855510240
7941	Limch1	9.799768373	9.703601149	9.74487837710241
7942	Limd1	6.807470468	6.851685788	6.80039196910242
7943	Limd2	8.735155603	8.896814725	8.54589911710243
7944	Lime1	4.9348557	4.975307261	3.97612864110244
7945	Limk1	7.759171656	7.389806605	7.91703938810245
7946	Limk2	7.99876061	7.785370123	7.87481102410246
7947	Lims1	6.681774876	6.894283096	6.89900958810247
7948	Lims2	4.784250503	5.467161203	4.67914635110248

	Pde7b	3.426385326	4.54207379	5.231058169
	Pde8a	5.629983664	5.36387307	5.337762213
	Pde8b	8.941958828	9.139704962	9.194385428
	Pde9a	6.737647839	7.024558079	6.503263122
	Pdf	8.19897422	7.981597015	8.067431365
	Pdgfa	7.821826041	7.910542475	7.518770676
	Pdgfb	6.722118652	6.64817375	6.708429175
	Pdgfc	4.876919127	4.429545932	3.14327382
	Pdgfra	7.289785504	7.322501814	7.488161397
	Pdgfrb	4.669198071	5.012230859	5.190703575
	Pdgfrl	2.402367415	1.810623602	1.977664017
	Pdha1	10.17037728	9.981280248	9.916608986
	Pdhb	9.545716203	9.369304987	9.227849885
	Pdhx	8.056385125	7.744398918	7.579626461
	Pdia3	9.740370179	9.757093547	9.664747066
	Pdia4	8.383700541	8.103599024	7.962689414
	Pdia5	3.066649489	1.378842945	1.978123579
	Pdia6	9.483438948	9.452674706	9.385110574
	Pdik1l	7.7026897	7.456955746	7.985957864
	Pdk1	7.17745945	7.17983142	7.293347923
	Pdk2	8.591933168	8.715838992	8.641896168
	Pdk3	7.595768641	7.538522332	7.659676372
	Pdk4	3.90307172	4.7494896	4.564752727
	Pdlim1	6.010379963	5.924942372	6.20959877
	Pdlim2	8.684074849	8.92690127	8.447421455
	Pdlim4	6.251816145	6.924724374	6.227522104
	Pdlim5	7.000053287	7.151780818	7.179847035
	Pdlim7	7.649794334	7.538154375	7.414406838
	Pdp1	9.600665748	9.724569495	10.12599914
	Pdp2	5.507389126	5.822297067	5.708459975
	Pdpk1	7.308585835	7.064698946	7.26550382
	Pdpn	7.306333619	7.327649259	6.623189432
	Pdpr	1.676288481	2.15694877	1.561548192
	Pdrg1	8.245085823	8.036472358	8.320002674
	Pds5a	6.492375618	6.590015694	6.922894974
	Pds5b	10.56425504	10.32643557	10.40938261
	Pdss1	6.054573853	5.950062248	5.564139401
	Pdss2	2.875168541	2.861527071	2.790067818
	Pdxdc1	8.250125444	7.944425733	8.199187548
	Pdxk	9.892719363	9.793229171	9.967280448
	Pdxk-ps	0.016612017	0.91553212	0.989757639
	Pdxp	9.894416751	9.895687855	10.02030099
	Pdyn	5.580757413	6.158802765	6.355444787
	Pdzd11	7.082148187	7.432053705	7.431423075
	Pdzd2	6.607013331	6.836422898	7.228502458
	Pdzd3	3.058256401	2.447528565	1.981208668
	Pdzd4	8.531639874	8.770323451	8.503554478
	Pdzd7	7.067518877	6.848833144	6.680023044
	Pdzd8	8.828788456	8.688716474	9.083436304
	Pdzd9	8.960199844	9.095783256	9.15501996

12499	Sh2d4a	2.04970068	1.8949789	0.985649213
12500	Sh2d4b	2.393063901	2.441793584	3.445627936
12501	Sh2d5	8.642715165	8.655054366	8.816190919
12502	Sh2d7	5.324717219	5.234624573	5.407457508
12503	Sh3bgr	3.673446891	4.30689074	3.979680172
12504	Sh3bgrl	9.564041618	9.510684755	9.425013877
12505	Sh3bgrl2	5.473222349	5.324597807	5.788324183
12506	Sh3bgrl3	10.15619381	10.48300431	10.07267998
12507	Sh3bp1	5.081855108	5.399405963	5.504811334
12508	Sh3bp2	3.415952238	4.438051701	4.150595148
12509	Sh3bp4	5.320627048	4.928566374	4.622926202
12510	Sh3bp5	9.713258712	9.594541905	9.306297616
12511	Sh3bp5l	8.897345748	8.965263435	9.008013748
12512	Sh3d19	6.105925248	6.637512432	6.265117313
12513	Sh3d21	3.889002293	5.527732518	3.786528
12514	Sh3gl1	7.628643288	7.978698101	7.65922192
12515	Sh3gl2	11.22393513	11.27096614	11.22256581
12516	Sh3gl3	7.780529849	7.938316384	7.503266204
12517	Sh3glb1	9.848753662	9.68437962	9.992659789
12518	Sh3glb2	9.105819016	9.283413043	9.199179743
12519	Sh3kbp1	7.914000383	8.165467458	7.979931558
12520	Sh3pxd2a	7.686865409	7.607036464	7.673700357
12521	Sh3pxd2b	4.844243639	5.328980882	5.472281014
12522	Sh3rf1	8.327437183	8.278196206	8.174837109
12523	Sh3rf2	4.670636195	4.402487813	5.654941262
12524	Sh3rf3	6.912262744	6.83884401	7.078636274
12525	Sh3tc2	3.395179941	3.668501369	3.1504891
12526	Sh3yl1	4.886074629	4.640138459	4.787363355
12527	Shank1	5.600170334	5.226918946	5.839687768
12528	Shank2	5.933549595	6.137277899	5.438968778
12529	Shank3	7.173100524	8.062003986	8.083319277
12530	Sharpin	6.985477617	6.981010455	6.934553934
12531	Shb	5.434651319	5.429157519	5.535761281
12532	Shc1	2.915843951	3.921545158	3.678665778
12533	Shc2	6.104063164	6.230807221	6.109006765
12534	Shc3	3.065313906	3.035972849	3.153969349
12535	Shc4	4.087244634	4.542682336	4.372097058
12536	Shcbp1	1.660553435	2.68468381	2.305535871
12537	Shcbp1l	3.769673285	3.766227103	3.44069025
12538	Shd	6.526143996	6.826947268	6.957595116
12539	She	4.938825328	4.737137489	5.109885164
12540	Shf	4.535419259	5.031583665	4.936870164
12541	Shfm1	9.374954833	9.284967993	9.154845578
12542	Shh	3.248537618	3.00049789	4.306811173
12543	Shisa2	3.997993796	5.024594955	5.152513403
12544	Shisa3	2.059614392	2.473104255	2.987776661
12545	Shisa4	7.699570995	7.158627666	7.179550489
12546	Shisa5	8.61998111	8.968630638	8.673451619
12547	Shisa6	7.596190507	7.71579811	7.118772329
12548	Shisa7	7.427831868	7.394722641	7.227924439

14799	Ttll9	1.083667385	2.184278352	1.98236523
14800	Ttn	4.944961778	4.821149966	6.61309048
14801	Ttpa	4.716697046	4.364172774	5.02741027
14802	Ttpal	6.470156711	6.002725757	6.51992440
14803	Ttr	13.97591954	15.47680447	12.5772804
14804	Ttyh1	11.06647524	11.03733226	11.0976159
14805	Ttyh2	6.311889335	6.854511107	6.59480913
14806	Ttyh3	9.391131396	9.369204956	9.35935753
14807	Tub	8.611698352	8.488526752	8.61304008
14808	Tuba1a	12.46691712	12.29391759	12.0500855
14809	Tuba1b	11.94395484	11.78793793	11.6993538
14810	Tuba1c	7.355058121	7.752546841	6.47039934
14811	Tuba3a	1.115996507	4.858044905	0.97001681
14812	Tuba3b	1.109700379	4.603041666	2.30137394
14813	Tuba4a	11.71810702	11.72044739	11.7382068
14814	Tuba8	4.721555266	4.700433713	4.83914008
14815	Tubb2a	11.40856173	11.22369597	10.8040787
14816	Tubb2b	11.68217965	11.42188299	11.1303293
14817	Tubb3	9.891208301	9.972338785	10.076746
14818	Tubb4a	9.222876914	9.464123314	9.39576816
14819	Tubb4b	9.46402452	9.454791764	9.26996242
14820	Tubb5	11.60705696	11.57927879	11.6404314
14821	Tubb6	2.400743587	1.814827216	1.56074816
14822	Tubd1	3.532556506	2.385023586	1.97224667
14823	Tube1	3.080790473	2.623301066	3.79055754
14824	Tubg1	6.863685244	7.12393349	7.02427930
14825	Tubg2	7.702637455	7.577873585	7.95759150
14826	Tubgcp2	6.666978946	7.089955127	6.81285000
14827	Tubgcp3	6.386655477	6.795300018	6.6238728
14828	Tubgcp4	8.527313412	8.340960996	8.46382434
14829	Tubgcp5	7.507301124	7.208190296	7.85072031
14830	Tubgcp6	6.65071196	6.874554976	6.68042150
14831	Tufm	5.962174533	5.882182738	6.06801181
14832	Tuft1	3.673034866	4.66714932	4.37391417
14833	Tug1	8.194187912	8.432338511	8.1448673
14834	Tulp2	4.476665751	6.322300721	4.30010069
14835	Tulp3	5.291340816	5.360877559	5.50400781
14836	Tulp4	9.416393869	9.493712259	9.89147306
14837	Tunar	5.479523512	5.81173634	6.06781544
14838	Tusc1	6.345125571	6.365989969	6.33805459
14839	Tusc2	8.781113723	8.835101498	8.73481119
14840	Tusc3	3.652087413	3.168166109	3.30366115
14841	Tusc5	2.386213211	3.600907086	0.97865912
14842	Tut1	5.406885698	5.589623236	6.04715178
14843	Tvp23a	6.083979907	6.454036705	6.68116670
14844	Tvp23b	7.581008974	7.799058934	7.60192215
14845	Twf1	7.61946718	7.530766619	7.42269079
14846	Twf2	7.430032325	7.665733604	7.52685032
14847	Twist1	3.249485312	3.157309641	3.44058391
14848	Twist2	3.964797977	3.451672306	2.56447448

1049	Adgrf5	7.279811803	7.131163593	7.208779927	3349	Cnksr2	11.33497786	11.15847029	11.36855048	5649	Gal3st1	3.991842612	4.300589394	4.440482412	7949	Lin28b	4.598183069	3.382401209	4.14929871410249	Pdzk1	5.206032013	4.966864298	5.150620968	12549	Shisa8	2.679309938	3.660177065	2.978406696	14849	Twistnb	7.913894602	8.012838235	7.922403061
1050	Adgrg1	7.854338035	7.964920103	7.707952912	3350	Cnksr3	4.07710907	4.822224897	3.980353351	5650	Gal3st3	7.685677952	7.921288031	6.886028697	7950	Lin37	4.937861972	5.240607476	4.73414811110250	Pdzk1ip1	3.233801092	3.580630712	1.978150047	12550	Shisa9	6.781918026	6.730018622	6.93428278	14850	Twsg1	6.572526403	6.783914004	6.594720177
1051	Adgrg2	3.985205658	4.012832622	4.229559636	3351	Cnn1	3.059734082	0.797792055	0.976170814	5651	Gal3st4	5.506017256	5.540701616	5.534868649	7951	Lin52	5.75422752	5.948841222	6.15082502210251	Pdzph1	3.976634452	3.291839499	4.153254923	12551	Shkbp1	5.112873731	4.600273737	3.56036216	14851	Txlna	7.580748526	7.764004014	7.787547415
1052	Adgrg6	2.909752019	4.016059199	4.568309665	3352	Cnn2	5.119300164	4.692722717	5.339696515	5652	Galc	5.253158558	5.453349141	5.912342622	7952	Lin54	6.058099813	6.285284778	6.20917491810252	Pdzrn3	7.278807668	6.944120924	7.406810642	12552	Shmt1	3.389962085	3.448657766	3.304591511	14852	Txlnb	3.986760635	4.009686833	4.441604745
1053	Adgrl1	11.75783278	11.76191833	11.77602847	3353	Cnn3	8.774192217	9.041113709	8.60553064	5653	Gale	6.537215046	6.543612158	6.580673557	7953	Lin7a	9.301558275	9.064262964	9.20407318910253	Pdzrn4	7.578672471	7.47647286	7.534925255	12553	Shmt2	5.605606716	5.504552614	5.652210821	14853	Txlng	6.847923513	6.970631084	6.774439038
1054	Adgrl2	7.223489739	7.624283033	8.189962593	3354	Cnnm1	8.34039037	7.743532908	7.844149447	5654	Galk1	5.783325533	6.522290732	6.266273955	7954	Lin7b	9.029021528	9.195989875	9.05946645910254	Pea15a	11.78265179	11.62778986	11.36743423	12554	Shoc2	9.655847344	9.495954014	9.459736897	14854	Txn1	9.425081134	9.701017166	9.459728092
1055	Adgrl3	5.963102165	5.924511193	6.150486742	3355	Cnnm2	6.270312715	6.441377705	6.519807475	5655	Galk2	6.288928496	6.641179652	6.023878367	7955	Lin7c	9.149229534	9.312458916	8.96858505810255	Peak1	8.245053216	8.331128169	7.806133917	12555	Shox2	6.245245091	7.032832824	9.03292499	14855	Txn2	9.393146674	9.313795004	9.179613571
1056	Adgrl4	6.766278495	6.696036374	6.564668226	3356	Cnnm3	6.738575348	6.708012832	6.774527759	5656	Galm	4.31700533	4.177178339	2.781732761	7956	Lin9	5.037845468	5.265303004	5.37286447210256	Pear1	2.65946504	3.331181963	2.982872603	12556	Shpk	2.098696681	4.048238402	2.786946351	14856	Txndc11	8.119939302	8.412911491	8.306496828
1057	Adgrv1	2.421032657	3.663229421	3.682815559	3357	Cnnm4	4.33819523	4.971562631	4.980596315	5657	Galns	4.593949116	4.498095297	3.436111768	7957	Lincpint	5.16691139	4.195266055	4.9794877110257	Pebp1	11.24976448	11.1799983	10.89433404	12557	Shprh	7.498016308	7.100115679	7.580118264	14857	Txndc12	5.864280255	6.216637785	6.266007602
1058	Adh1	4.163264875	3.122691182	3.436753944	3358	Cnot1	8.058373205	8.011352127	8.29752236	5658	Galnt1	9.103078222	8.93213003	9.251497883	7958	Lincppara	5.400349102	5.156435452	5.40678848110258	Pecam1	4.998967095	5.745831126	5.863152262	12558	Shq1	3.77587936	3.418080313	3.438964178	14858	Txndc15	9.096792944	8.970126152	8.645145101
1059	Adh5	8.172183962	8.228433374	7.831605519	3359	Cnot10	7.198542924	7.179196412	6.707140673	5659	Galnt10	4.833311438	5.097952677	4.887612322	7959	Lingo1	10.07612747	10.03531432	10.0674933110259	Pecr	5.394420933	4.882242368	5.19067272	12559	Shroom1	3.987019332	5.082394779	4.067929317	14859	Txndc16	7.531896265	7.32692815	7.680612162
1060	Adhfe1	6.080095215	5.98621992	6.423979187	3360	Cnot11	6.557875628	6.562860864	6.761435669	5660	Galnt11	5.728506305	6.027420316	6.228464679	7960	Lingo2	6.950301923	6.80235067	7.12001928810260	Pef1	8.777130622	8.757317782	8.616580035	12560	Shroom2	8.352575852	8.455917665	8.701252766	14860	Txndc17	8.124271291	8.124508541	7.868592367
1061	Adi1	7.042873118	6.645059797	6.534293361	3361	Cnot2	7.867664085	7.720452429	7.916877737	5661	Galnt12	3.236832192	2.142561886	3.98603677	7961	Lingo3	7.650718517	7.959365674	7.83814731810261	Peg10	4.983232503	4.744897244	4.935009456	12561	Shroom3	3.988945735	4.717883842	4.505519488	14861	Txndc5	5.865679781	6.024442777	6.372874733
1062	Adipor1	7.507201064	7.619784558	7.714906852	3362	Cnot3	8.156357908	8.106083297	7.767666487	5662	Galnt13	6.710812819	6.883464474	7.256993254	7962	Lingo4	2.696679437	3.634667166	4.68384528610262	Peg12	1.653872575	1.437561153	1.567413299	12562	Shtn1	8.051051128	8.102348232	7.800108375	14862	Txndc9	8.922744474	9.005989515	8.774336502
1063	Adipor2	8.560817231	8.581912317	8.459565581	3363	Cnot4	8.38899485	8.337120887	8.684122813	5663	Galnt14	5.042583307	5.891825813	5.301093665	7963	Lins	4.717378276	4.226293627	4.06616040110263	Peg13	9.333145062	9.353764981	9.324295932	12563	Siae	6.726158122	6.957335659	7.266045731	14863	Txnip	7.410499547	7.34662999	7.159697036
1064	Adk	8.752481831	8.491761094	8.670039901	3364	Cnot6	8.646872675	8.76881272	8.868833099	5664	Galnt16	8.250216017	8.382379381	8.007694274	7964	Lipa	6.346274948	6.544188124	6.51984442810264	Peg3	10.21650263	10.0005548	10.69343699	12564	Siah1a	7.437804862	7.407882956	7.715185358	14864	Txnl1	9.072017894	9.047285759	9.177280903
1065	Adm	3.238560633	1.359292005	1.558545022	3365	Cnot6l	9.253003053	9.313791123	9.308686694	5665	Galnt18	7.020019835	6.829177969	6.518769363	7965	Lipe	6.187552793	6.234022787	6.33831642710265	Peg3os	9.481694476	9.211370518	9.922623469	12565	Siah1b	5.985020559	5.971354504	6.190617071	14865	Txnl4a	8.977962338	8.806399656	8.676843584
1066	Adnp	9.248121759	9.299257823	9.437430449	3366	Cnot7	8.052514561	8.136993448	8.447898647	5666	Galnt2	7.801011091	7.550563508	7.680416953	7966	Lipg	3.236834178	2.850204204	3.44356183610266	Peli1	9.049454023	9.23055301	9.139856471	12566	Siah2	6.104168954	6.282339094	6.406781786	14866	Txnl4b	5.75334284	5.928968479	5.680088308
1067	Adnp2	4.405505392	4.06597486	4.68093952	3367	Cnot8	7.998484388	7.871797136	7.541847695	5667	Galnt3	4.238901678	3.749656481	3.147257346	7967	Liph	2.398258263	1.821231311	3.15413758110267	Peli2	7.035258116	6.954581658	7.274926838	12567	Sidt1	7.966825765	7.359269744	7.638352637	14867	Txnrd1	7.856281111	7.875189149	8.124732702
1068	Ado	7.809277975	8.105884435	8.109435776	3368	Cnp	11.10922151	11.46062343	11.0547715	5668	Galnt4	4.536864043	4.899679241	4.301586392	7968	Lipo1	2.655044801	2.160938979	0.97420463510268	Peli3	3.238027666	2.414518982	2.981877716	12568	Sidt2	6.900026654	6.937797279	6.680114402	14868	Txnrd2	5.396362896	4.965794486	4.501232851
1069	Adora1	10.62334228	10.42004811	10.33311868	3369	Cnppd1	7.892999028	7.816851516	7.856778924	5669	Galnt5	2.367412806	0.885066935	0.986164531	7969	Lipt1	5.778369436	5.724027031	5.56486192110269	Pelo	7.155649951	6.753570905	7.078287351	12569	Sigirr	2.054141923	2.214291073	2.309036752	14869	Txnrd3	5.504988857	5.625877815	5.188682904
1070	Adora2a	4.903932905	5.926579127	6.303160175	3370	Cnpy2	8.496743457	8.085636024	7.91050416	5670	Galnt6	6.20738865	6.49211949	6.00234988	7970	Lipt2	3.785541489	3.629333479	4.50578851210270	Pelp1	7.218289997	7.125313071	6.786922307	12570	Siglecf	2.855608832	1.878828742	1.569314357	14870	Tyk2	4.25484301	4.850259009	4.788682608
1071	Adora2b	4.8806682	4.940893286	3.559872471	3371	Cnpy3	8.887475153	8.970955289	8.806581369	5671	Galnt7	7.347014424	7.237872443	7.129429522	7971	Litaf	6.509365308	7.138280283	6.72134128510271	Pemt	3.889337691	4.090523323	4.374186357	12571	Siglech	6.420487015	6.700950569	6.337299012	14871	Tymp	4.238885608	4.022743764	4.304998353
1072	Adora3	2.070317026	2.181999009	2.985796798	3372	Cnpy4	7.069287117	7.142599521	6.910395929	5672	Galnt9	8.915525013	8.516912839	8.718190865	7972	Lix1	8.913230061	9.018698254	8.75471232310272	Penk	8.248086712	8.948245441	9.319963459	12572	Sigmar1	6.681019495	6.804730976	6.609040776	14872	Tyms	2.663197627	3.025189971	3.30535808
1073	Adpgk	5.440065934	5.322407481	5.761987435	3373	Cnr1	9.028526338	8.873949352	8.957314783	5673	Galntl6	6.03494133	6.006706906	6.320595331	7973	Lix1l	5.575209187	5.667938362	5.86292029910273	Peo1	4.334490869	4.844530047	5.069186495	12573	Sik1	4.475650975	4.748930475	5.2679107	14873	Tyro3	8.818661813	9.031235363	8.982911924
1074	Adprh	8.993603984	9.132832781	9.032540916	3374	Cnrip1	9.059854029	8.846706881	8.583469067	5674	Galr1	0.062655297	1.434391193	3.157264553	7974	Llgl1	8.360990311	8.404842391	8.27460338910274	Pepd	6.418906906	6.26368858	5.933151338	12574	Sik2	2.891794979	3.556476028	3.30306814	14874	Tyrobp	6.603162757	6.900072032	6.246080859
1075	Adprhl1	0.005608334	0.940318714	0.992698372	3375	Cnst	6.29110404	6.498174086	6.389373789	5675	Galt	6.621045553	6.980997333	7.057417396	7975	Llgl2	2.883668813	3.178295333	4.23421515110275	Per1	7.124305352	6.625311125	6.812403822	12575	Sik3	7.261636652	7.460237319	7.594947227	14875	Tyrp1	0.024624946	2.728757807	1.573375513
1076	Adprhl2	2.38631364	3.057326745	1.98235299	3376	Cntd1	8.932045958	8.923082474	8.994110753	5676	Gamt	6.539956067	6.831794017	6.624051296	7976	Llph	7.715887688	7.503167383	7.36346818510276	Per2	7.101198893	6.705016748	7.002578003	12576	Sike1	7.79246637	7.100422067	7.565185946	14876	Tysnd1	5.635649644	5.590483659	5.471123496
1077	Adprm	5.694696764	5.618175863	5.594486272	3377	Cntf	3.649333801	3.016833673	2.563776792	5677	Gan	4.877171202	3.505231431	4.372849799	7977	Lman1	8.250381948	8.113032364	8.35515346110277	Per3	6.189395043	5.896395703	6.66763134	12577	Sil1	6.739068982	7.119496088	6.88712459	14877	Tyw1	5.323454939	4.965721639	4.786389679
1078	Adra1a	3.881340915	3.537410631	4.230983409	3378	Cntfr	5.034426919	4.735045635	5.22874644	5678	Ganab	8.564011363	8.376375651	8.43116728	7978	Lman2	8.446638977	8.682210687	8.44356538110278	Perm1	2.64078835	1.864604106	2.570777693	12578	Simc1	7.649454966	7.517584498	7.580027794	14878	Tyw3	6.097251939	5.910347466	5.863307136
1079	Adra1b	6.523930586	6.80792741	7.07912826	3379	Cntln	8.506493613	8.518006598	8.553753355	5679	Ganc	5.890021512	5.922081718	6.488955858	7979	Lman2l	5.697363488	5.990965356	5.88699122410279	Perp	3.996034773	4.898361719	4.839797768	12579	Sin3a	7.547179985	7.497286978	7.572718253	14879	Tyw5	6.34780093	6.555311255	6.423014532
1080	Adra1d	3.539537983	3.28696023	3.788645624	3380	Cntn1	11.77841767	11.80756282	11.83134434	5680	Gap43	11.70619524	11.23357772	11.61207857	7980	Lmbr1	7.671608954	7.155939443	7.64562295210280	Pes1	7.325330215	6.914530435	6.850200924	12580	Sin3b	8.419930354	8.621476567	8.22308285	14880	U2af1	7.814796826	7.529102228	8.046476513
1081	Adra2a	6.466947581	5.924682968	6.088930402	3381	Cntn2	9.370757173	9.65069738	9.45368907	5681	Gapdh	8.091597733	8.007366309	8.204155001	7981	Lmbr1l	3.402077352	4.111705773	3.68187284710281	Pet100	8.304377271	8.450279288	8.439590741	12581	Sipa1	6.099832562	5.927081241	5.812743148	14881	U2af1l4	7.992320836	7.953312169	7.85044537
1082	Adra2b	2.656620428	0.773226219	3.154112356	3382	Cntn3	5.368946087	5.74955309	6.21078526	5682	Gapvd1	8.488761384	8.281449663	8.297501153	7982	Lmbrd1	9.805216891	9.958237318	9.96585157110282	Pet117	6.228291563	5.855238	6.504752483	12582	Sipa1l1	11.32310498	11.37176503	11.35503746	14882	U2af2	9.832102409	9.827927565	9.87026438
1083	Adra2c	6.886797989	6.656126821	7.109983524	3383	Cntn4	6.952695249	7.204554147	7.337821921	5683	Gar1	7.949576322	7.651065748	7.957599835	7983	Lmbrd2	9.561692725	9.10162522	9.48194396310283	Pex1	6.523827128	6.56592681	6.721840971	12583	Sipa1l2	7.31994683	7.001465299	7.721831315	14883	U2surp	10.1262733	10.09759191	9.991179934
1084	Adrb1	7.686255133	7.35260439	7.755263242	3384	Cntn5	4.772111358	3.617096053	4.682230695	5684	Garem	7.239753885	7.299335099	7.07788296	7984	Lmcd1	4.922217944	4.167716297	3.98012564610284	Pex10	6.205714827	5.969547483	6.08917943	12584	Sipa1l3	7.124665529	7.487091795	6.874376697	14884	Uaca	5.837249425	5.497493606	6.504996629
1085	Adrb3	2.661724009	3.327441037	3.153026946	3385	Cntn6	5.36763743	4.906901213	5.981622944	5685	Gareml	4.989052403	5.234696096	5.266091534	7985	Lmf1	6.247615676	5.923258653	5.86217524610285	Pex11a	6.402645775	6.540664614	6.735637927	12585	Sirpa	9.756213175	9.551962756	9.594699361	14885	Uap1	8.112897001	7.74323685	8.056998561
1086	Adrbk1	9.219848028	9.273182928	9.270095871	3386	Cntnap1	7.680820245	7.707833955	7.974637397	5686	Garnl3	7.673620373	7.88875914	8.150360869	7986	Lmf2	4.788045201	5.631642302	5.37300517310286	Pex11b	7.758716163	7.716367436	7.471738437	12586	Sirt1	6.587355949	6.374686211	6.838777081	14886	Uap1l1	5.436977151	5.755276912	5.188759525
1087	Adrbk2	8.450256107	8.055734507	8.431417337	3387	Cntnap2	8.592361708	8.509627956	8.937338985	5687	Gars	9.181034374	9.106969388	9.070076731	7987	Lmln	4.725804711	5.014495685	4.37024657910287	Pex11g	3.239638583	2.843583612	2.981436458	12587	Sirt2	9.402329238	9.408005436	9.189363797	14887	Uba1	11.18050741	11.23889004	10.92832414
1088	Adrm1	7.907215758	8.215414805	7.934188674	3388	Cntnap3	3.055941287	2.45344992	2.569292975	5688	Gart	6.489877351	6.867102392	6.487791413	7988	Lmna	7.692753141	7.498491971	7.30190772110288	Pex12	5.20483951	5.275596431	4.786857508	12588	Sirt3	7.374170045	6.983966164	7.363915904	14888	Uba1y	0.021977226	1.493094202	0.988489756
1089	Adsl	5.887907522	6.070978707	5.91079431	3389	Cntnap4	6.308816186	6.456584503	6.066894768	5689	Gas1	6.104230424	6.212820604	6.709151816	7989	Lmnb1	6.146285379	6.250156563	6.0673035310289	Pex13	8.959939919	8.904006957	8.907776957	12589	Sirt4	2.673724527	3.437964857	3.984191196	14889	Uba2	7.659214525	8.008936063	7.880848671
1090	Adss	8.588362364	8.612759819	8.634657418	3390	Cntnap5a	3.385918489	1.806150183	3.986136031	5690	Gas2	5.913246413	5.715770789	6.407690495	7990	Lmnb2	5.539699917	5.256223904	5.83902843410290	Pex14	7.633404283	7.110509445	7.572772576	12590	Sirt5	4.548558417	5.237193517	5.596841723	14890	Uba3	7.650683227	7.631533382	7.714719606
1091	Adssl1	5.781034569	5.443078449	5.787350009	3391	Cntnap5b	3.98888258	3.730898912	4.937787451	5691	Gas2l1	7.548347849	7.764695846	7.65251892	7991	Lmntd2	3.066602608	3.332326392	2.98302817810291	Pex16	5.887080389	5.883678778	5.502426603	12591	Sirt6	4.724661713	4.410593316	5.11076699	14891	Uba5	8.622939875	8.441451684	8.260584843
1092	Adtrp	1.07200857	1.870219851	2.571313646	3392	Cntnap5c	2.387450666	2.453247679	2.569269459	5692	Gas2l2	3.54730353	4.243225352	4.567938214	7992	Lmo1	6.834010806	6.563627794	6.51876229610292	Pex19	8.298555974	8.27024607	7.910573118	12592	Sirt7	6.062619545	6.661409778	6.389142436	14892	Uba52	11.61964413	11.48079284	11.23793041
1093	Aebp1	6.293327311	6.493889985	6.549703537	3393	Cntrl	6.680216763	7.034001521	6.405638028	5693	Gas2l3	3.996937754	4.421563538	4.503781787	7993	Lmo2	6.72462189	6.816289056	7.13052038910293	Pex2	5.840246682	6.212669123	6.53539428	12593	Siva1	6.402620227	6.713817896	6.667101101	14893	Uba6	6.99807976	6.718361818	6.837958831
1094	Aebp2	8.332629508	8.031239297	8.385321356	3394	Cntrob	4.779394662	5.282226417	4.838597671	5694	Gas5	10.74144515	10.7507411	10.60475196	7994	Lmo3	7.829054977	7.973076002	7.84411782910294	Pex26	6.222960132	5.320503329	5.652462659	12594	Six1	1.674985362	2.159206567	2.983710067	14894	Uba7	3.393544299	3.168205089	2.562784121
1095	Aen	5.663930276	5.449577367	5.336734344	3395	Coa3	8.990247623	9.119622321	8.711158837	5695	Gas6	8.397891667	8.532410137	8.319780752	7995	Lmo4	11.20262764	11.18564327	11.0727484610295	Pex3	7.6041644	7.619278377	7.511218131	12595	Six2	1.655159648	1.434662599	1.567078765	14895	Ubac1	8.074781649	7.929524584	8.078052714
1096	Aes	11.88549032	11.91401546	11.68861985	3396	Coa4	4.325709632	3.725495476	4.504997539	5696	Gas7	10.61766105	10.59623516	10.49469831	7996	Lmo7	8.868534341	9.048079326	8.87781998910296	Pex5	6.144053779	5.946654701	5.98012928	12596	Six3	7.149856351	7.702804604	7.694320691	14896	Ubac2	7.595706818	7.765547619	7.337266164
1097	AF251705	3.972822494	3.552120615	3.440674092	3397	Coa5	8.890801733	8.832478636	8.819217448	5697	Gas8	5.75231689	6.152326463	5.887468368	7997	Lmod1	4.602622243	5.064617815	5.02644710610297	Pex5l	9.485876838	9.536391237	9.79064727	12597	Six5	3.524240661	2.642993076	3.44293079	14897	Ubald1	9.627182877	9.611421938	9.404112711
1098	AF357355	2.411393717	2.838467732	2.298997285	3398	Coa6	6.926356338	6.847187267	6.922417244	5698	Gata2	2.917466985	2.565411613	3.885946758	7998	Lmtk2	5.946832401	6.794046934	7.04685864610298	Pex6	6.535913911	5.962440542	5.909959256	12598	Ska1	1.630820342	1.488650398	1.989084831	14898	Ubap1	7.587497546	7.368151838	7.389274368

1099	AF357359	1.103102313	2.157206809	3.154126883	3399	Coa7	6.769757516	7.196951742	7.398389156	5699	Gata3	3.702223171	4.258327755	5.864577189	7999	Lmtk3	8.919225905	9.024261619	8.793805988	10299	Pex7	6.105956196	6.59718289	6.580431958	12599	Ska2	4.602881883	4.938429934	4.503391056	14899	Ubap2	7.603947432	7.728268292	7.519059546
1100	AF357425	3.894832649	3.252105377	4.149539855	3400	Coasy	7.154554336	6.755659615	6.579357867	5700	Gatad1	9.468404757	9.355386782	9.540394081	8000	Lmx1a	3.527993068	4.275292978	0.966427204	10300	Pf4	5.426208222	3.362357272	4.735189573	12600	Ska3	2.394888032	2.164945292	2.567507104	14900	Ubap2l	9.103064101	9.38376133	9.037904844
1101	AF529169	4.653665709	3.734188707	4.683423242	3401	Cobl	7.868058656	7.91586511	8.073088511	5701	Gatad2a	7.775139079	8.105666491	8.204230095	8001	Lnp	8.080837152	8.061145678	8.427357544	10301	Pfas	5.208391533	5.550584293	5.654320905	12601	Skap1	3.769237121	3.66406618	2.978999828	14901	Ubash3b	8.459616009	8.300801511	8.451730901
1102	Afap1	6.75059172	6.326057651	6.56509421	3402	Cobll1	4.99157726	5.332477357	5.189250797	5702	Gatad2b	8.893754506	8.902366086	8.655905485	8002	Lnpep	6.213125527	6.3583072	6.487718942	10302	Pfdn1	9.468276084	9.270146651	9.215940611	12602	Skap2	6.418233159	6.002724476	6.149705031	14902	Ubb	2.86833079	2.447455247	2.568596402
1103	Afap1l1	5.753898138	5.927895072	6.303320148	3403	Coch	5.00329901	5.833739049	6.067653663	5703	Gatb	6.245088881	5.992148527	5.623226633	8003	Lnx1	5.443080098	5.667659144	5.957817696	10303	Pfdn2	9.260509685	9.147883602	9.363734803	12603	Ski	9.451028777	9.313430762	9.435357363	14903	Ubc	11.75390864	11.79416789	11.87858992
1104	Afap1l2	4.166376115	4.235618985	4.441031846	3404	Cog1	5.940083075	6.251402875	6.002170391	5704	Gatc	7.506934242	7.429138056	7.741851648	8004	Lnx2	4.469806372	3.995201435	4.889316102	10304	Pfdn4	7.717900616	7.79731233	7.819150384	12604	Skida1	5.665791667	5.794825525	6.151339387	14904	Ube2a	7.250340979	7.462913108	6.990763546
1105	Aff1	5.781113981	5.906804178	5.564168876	3405	Cog2	6.928216484	7.306871719	7.189417081	5705	Gatm	8.923056438	9.178659436	8.922464351	8005	LOC100048884	4.933050733	0.4125665	4.438592719	10305	Pfdn5	10.49204837	10.4653575	10.55554551	12605	Skil	8.894212653	8.834928994	8.960200804	14905	Ube2b	10.19161352	10.06891077	10.10797358
1106	Aff2	5.333928188	5.589878647	6.267103633	3406	Cog3	5.698933117	6.145634519	6.228662203	5706	Gatsl2	8.259275042	8.062398118	7.892747062	8006	LOC100503496	6.52029809	6.331957348	6.189340015	10306	Pfdn6	8.20360989	8.115205338	7.928296563	12606	Skiv2l	7.309363269	7.483930381	7.48807558	14906	Ube2c	2.879221947	2.146243858	3.305810994
1107	Aff3	7.861334028	7.478635311	7.916834306	3407	Cog4	7.586719997	7.280000896	7.406362179	5707	Gatsl3	3.970932977	3.163147045	3.303077269	8007	LOC100504039	2.640796154	2.694488476	0.980767321	10307	Pfkfb2	6.169131025	6.347342094	6.337733659	12607	Skiv2l2	9.602375383	9.541131605	9.820800855	14907	Ube2cbp	3.250789414	3.859556602	2.978375492
1108	Aff4	9.440414072	9.483455919	9.613035559	3408	Cog5	4.720819355	4.649533784	4.226476325	5708	Gba	6.102490211	6.335533803	5.403959546	8008	LOC100504703	5.688710259	5.630150509	4.932919059	10308	Pfkfb3	6.875857384	6.777034441	6.665809932	12608	Skor1	1.064588248	2.211591743	1.56975974	14908	Ube2d1	7.419999203	7.288103529	7.414703694
1109	Afg3l1	7.1587949	7.377262115	7.246585836	3409	Cog6	7.056109157	6.667545406	6.874853329	5709	Gba2	7.548288329	7.465979656	7.579915421	8009	LOC100505025	3.0605321	2.441694177	1.563368661	10309	Pfkfb4	4.541833357	4.590817869	4.981393785	12609	Skp1a	11.06644212	11.09045446	11.06942672	14909	Ube2d2a	9.89411173	9.822466694	9.766260907
1110	Afg3l2	7.61959097	7.764455265	7.301448667	3410	Cog7	7.237832234	7.039487069	7.002535989	5710	Gbas	8.54040933	8.850153411	8.590951506	8010	LOC100861615	2.069267857	1.422284268	2.985990522	10310	Pfkl	9.20140337	9.227251349	8.757936956	12610	Skp2	3.99236526	4.079670873	4.302239695	14910	Ube2d2b	0.024631466	2.503105002	0.987889032
1111	Afm	1.647925699	0.85482878	1.568955904	3411	Cog8	5.401931727	5.82733892	5.406397568	5711	Gbe1	6.765698483	6.32380191	6.863168099	8011	LOC100862268	8.467831149	8.216716758	8.306441214	10311	Pfkm	9.578496212	9.650093999	9.662996335	12611	Sla	4.665552075	5.248028398	5.191516838	14911	Ube2d3	9.684097926	9.65488488	9.491674713
1112	Afmid	5.317703928	4.213665314	4.887919624	3412	Coil	7.101113217	6.730070247	7.18024326	5712	Gbf1	8.25521812	8.199830845	8.297504908	8012	LOC105245869	2.646435887	3.2117781	1.982081717	10312	Pfkp	9.8182072	9.669489426	9.538393384	12612	Sla2	8.446855673	8.500026005	8.346220983	14912	Ube2e1	9.201683861	9.128275998	9.142440122
1113	Aftph	9.656094765	9.651607482	9.728209413	3413	Col11a1	3.561049493	4.481904314	4.982093226	5713	Gbp11	0.032496098	1.898409489	2.310431649	8013	LOC105246506	6.103263562	6.413664551	6.189604065	10313	Pfn1	6.770444907	7.604860003	6.837294519	12613	Slain1	6.557458053	6.840668398	6.503131412	14913	Ube2e2	7.709417108	8.00666766	7.487576477
1114	Aga	6.101063895	6.029499599	6.209560448	3414	Col11a2	2.678211851	3.951672464	1.553173051	5714	Gbp2	4.827537828	4.986909844	4.98248724	8014	LOC106740	6.664940545	6.564984172	6.800609251	10314	Pfn2	11.37055442	11.3366909	11.3647742	12614	Slain1os	0.052914607	1.447246843	1.568534189	14914	Ube2e3	8.861447165	8.84423978	8.65236676
1115	Agap1	10.15083763	10.32878873	10.40942761	3415	Col12a1	2.673831694	3.557549757	2.979639093	5715	Gbp3	3.786360268	3.829075279	3.9796759	8015	LOC432842	5.124188674	5.370025029	5.109390156	10315	Pfn4	5.122857154	5.00804408	4.622162465	12615	Slain2	7.665829211	7.85040937	7.86281864	14915	Ube2f	6.861492152	7.021076056	6.623410957
1116	Agap2	10.49083166	10.43517682	10.3064091	3416	Col13a1	3.248594546	3.433140605	2.296602377	5716	Gbp4	2.864924774	2.455625577	1.565677803	8016	Loh12cr1	7.634073438	7.546185745	7.439379896	10316	Pgam1	10.25158662	10.11391221	10.17229045	12616	Slamf8	1.062774549	0.867250116	1.57015144	14916	Ube2g1	8.250998607	8.58747233	8.463879424
1117	Agap3	10.38757977	10.2984336	10.23383308	3417	Col14a1	3.397897623	4.583796745	3.302539918	5717	Gbp5	3.778891355	5.00891447	3.564346619	8017	Lonp1	7.840621578	7.465811856	7.728385792	10317	Pgam2	4.324341588	4.766780123	2.972936308	12617	Slbp	5.9084652	5.44993245	5.471588978	14917	Ube2g2	7.506192658	7.532814316	7.034606928
1118	Agbl2	2.898814452	3.855837072	3.148694775	3418	Col15a1	2.086283777	3.460535924	3.444126516	5718	Gbp6	1.673928988	3.040146989	3.154455945	8018	Lonp2	8.831845032	8.759744396	8.673415724	10318	Pgam5	7.900447359	7.909927393	7.968823945	12618	Slc10a1	1.070176618	3.227007132	1.984859362	14918	Ube2h	9.726188356	9.653038046	9.774427881
1119	Agbl3	3.532852015	3.66327004	3.790194813	3419	Col16a1	4.890340626	5.371511665	5.150384729	5719	Gbp7	6.451268611	6.251294871	6.046010577	8019	Lonrf1	7.943908121	7.768390918	7.910883449	10319	Pgap1	8.268795181	8.072755322	8.007984609	12619	Slc10a3	5.205244397	5.406775099	5.110019632	14919	Ube2i	8.319014807	8.39287287	8.184409637
1120	Agbl4	4.832375763	5.317311003	4.735203442	3420	Col17a1	1.050299644	1.484389676	0.98730052	5720	Gbp9	3.755321816	2.156976937	2.302387585	8020	Lonrf2	4.836224014	4.689064174	4.679529772	10320	Pgap2	6.961830748	7.070900441	6.707690345	12620	Slc10a4	2.083376246	4.064654255	1.56136234	14920	Ube2j1	9.230517212	9.268052307	9.435369166
1121	Agbl5	8.246519331	8.773106163	8.605764906	3421	Col18a1	4.166688178	4.495558841	4.440959406	5721	Gbx2	0.024667824	0.899661856	1.989082643	8021	Lonrf3	7.456990567	7.522505632	7.687347539	10321	Pgap3	4.594184721	4.373178801	3.979662507	12621	Slc10a7	4.932055089	4.287003513	4.680973293	14921	Ube2j2	1.653870384	2.19583441	1.983864515
1122	Ager	2.390329927	2.677071242	1.564313525	3422	Col19a1	6.285605017	5.925478175	6.002616303	5722	Gc	5.86725396	4.22414788	6.863984954	8022	Lor	5.474394188	6.22846195	5.788046261	10322	Pgbd1	3.655829107	3.302480032	2.561416737	12622	Slc11a1	3.644993552	1.368911139	2.300930498	14922	Ube2k	10.66173596	10.66919849	10.57709367
1123	Agfg1	7.658062473	7.878915976	7.518923225	3423	Col1a1	6.825937386	7.19363278	5.99926638	5723	Gca	6.887590447	6.979448084	7.160510685	8023	Lox	2.082507982	1.821401517	2.302445612	10323	Pgbd5	9.615827189	9.713149864	9.47177559	12623	Slc11a2	6.54174703	6.884983749	6.875137571	14923	Ube2l3	9.765146561	9.747973003	9.612956071
1124	Agfg2	8.783740058	8.599598916	8.499564888	3424	Col1a2	7.492607038	7.924836619	8.355441199	5724	Gcat	5.573911566	5.974463053	5.594401952	8024	Loxl1	3.098253124	4.444006877	3.980674172	10324	Pgd	6.913073911	7.098787729	6.85030832	12624	Slc12a1	0.013894425	0.921232025	0.990433519	14924	Ube2l6	2.902485115	4.183567456	3.681235169
1125	Aggf1	9.500332722	9.517724767	9.467854838	3425	Col20a1	0.037724887	1.891550821	1.571009543	5725	Gcc1	6.949429988	6.744102337	6.7480999	8025	Loxl2	3.778675249	4.386543734	3.146958988	10325	Pgf	5.3215002	5.011875803	4.837774611	12625	Slc12a2	9.157637729	9.541073097	9.184508788	14925	Ube2m	7.070188591	7.463453254	7.045799761
1126	Agk	4.942193092	4.779252873	5.535960157	3426	Col22a1	2.878532536	3.187905675	3.986398799	5726	Gcc2	10.77665374	10.69023193	10.80574618	8026	Loxl3	7.23003974	7.347701192	7.056544702	10326	Pggt1b	4.985812595	4.404812624	5.505833012	12626	Slc12a4	5.127841866	5.706768295	5.50427074	14926	Ube2n	9.162466605	9.0545233	9.013375497
1127	Agl	8.239571723	7.789594226	8.237577927	3427	Col23a1	6.423218433	4.914936963	5.653911653	5727	Gcdh	5.937545376	5.83719647	6.13054328	8027	Lpar1	9.472872252	9.766635714	9.429114168	10327	Pgk1	9.019681952	8.936285297	8.901857184	12627	Slc12a5	9.983253249	9.921894285	10.57533608	14927	Ube2o	8.51064476	8.288131961	8.65262022
1128	Agmat	2.382127786	1.434560872	3.573410532	3428	Col25a1	7.596158151	7.891009402	7.534506834	5728	Gcfc2	5.725583384	5.640651769	5.533612337	8028	Lpar3	0.045235959	3.077862751	0.983612398	10328	Pgls	7.893691421	7.861089814	7.714616558	12628	Slc12a6	8.845332672	8.759374301	8.971618341	14928	Ube2q1	9.173353299	9.115955976	9.235040251
1129	Agmo	1.096195995	1.399328505	1.563037059	3429	Col26a1	2.40083339	2.85714162	2.301757428	5729	Gch1	1.13490098	3.557731273	2.785947343	8029	Lpar4	6.009715101	5.638696783	5.470391727	10329	Pglyrp1	3.774748428	3.420671459	3.681496618	12629	Slc12a7	4.657911287	4.299469236	4.302221711	14929	Ube2q2	7.751965458	7.495058747	7.787634145
1130	Ago1	7.788064326	7.725656422	7.856555796	3430	Col27a1	4.238273648	3.536132473	4.152536178	5730	Gck	3.782177765	4.725304844	3.887099253	8030	Lpar6	6.539105715	6.622400895	6.455723849	10330	Pgm1	4.259546868	5.381430875	4.981089445	12630	Slc12a8	0.05766432	1.440916936	2.794117268	14930	Ube2ql1	9.016517608	8.849435825	8.948770152
1131	Ago2	8.139182346	7.611261407	7.862794689	3431	Col2a1	2.068040285	3.059099336	2.305806422	5731	Gclc	8.275300048	8.354161272	8.149785381	8031	Lpcat1	2.883678359	3.782747034	3.568949291	10331	Pgm2	7.787947447	7.838284469	7.673310339	12631	Slc12a9	6.080751377	6.197617451	6.265864559	14931	Ube2r2	9.240454317	9.29540234	9.218372333
1132	Ago3	5.209186491	5.301173778	5.407141203	3432	Col3a1	5.401529661	4.44958628	5.372423807	5732	Gclm	7.739375208	7.826271327	7.874833468	8032	Lpcat2	5.602892521	5.978772026	5.594962631	10332	Pgm2l1	12.22548786	12.06964488	11.9757431	12632	Slc13a3	6.286490466	5.833988366	6.473170367	14932	Ube2s	6.694549272	6.722305675	6.518899116
1133	Ago4	5.29158215	5.598405068	5.372225935	3433	Col4a1	5.808036064	5.971926602	5.734736194	5733	Gcn1l1	6.327781546	6.543877684	6.088003069	8033	Lpcat3	8.057580557	8.19261843	7.916519625	10333	Pgm3	7.932241129	7.898329707	8.17995938	12633	Slc13a4	5.606091255	5.719763764	5.83826723	14933	Ube2t	2.387350937	1.419351552	2.305262028
1134	Agpat1	8.498531931	8.76111812	8.507441338	3434	Col4a2	5.832361417	5.698207425	5.471351039	5734	Gcnt1	5.031116591	5.410105484	4.299422574	8034	Lpcat4	1.052500867	2.229385246	2.31080147	10334	Pgm5	5.034946142	5.608649689	5.15031965	12634	Slc13a5	4.470512403	4.601563295	4.681893797	14934	Ube2v1	9.040351006	9.037884627	9.002379518
1135	Agpat2	4.07553306	4.381205453	3.980758094	3435	Col4a3bp	8.375286161	8.044499313	8.538528143	5735	Gcnt2	7.045274777	7.009202042	7.024596466	8035	Lpgat1	10.29118774	10.31369604	10.44458349	10335	Pgp	9.043175567	9.040464146	8.931264328	12635	Slc14a1	6.127292467	6.528292651	6.087826787	14935	Ube2v2	8.851545537	8.605496215	8.634577537
1136	Agpat3	8.468360359	8.411482103	8.310807803	3436	Col4a4	3.23078992	0.779895493	1.56213869	5736	Gcnt4	4.33827483	5.013388945	5.441696534	8036	Lpin1	6.938587437	6.626835589	7.02453996	10336	Pgpep1	6.66434587	6.435593146	6.57998219	12636	Slc14a2	2.637675377	2.898015513	1.568539713	14936	Ube2w	8.464276212	8.445005898	8.306265849
1137	Agpat4	8.622972242	8.524839257	8.418748149	3437	Col4a5	1.655328214	1.861764197	2.793610446	5737	Gcsh	7.724838434	7.780128549	7.831773914	8037	Lpin2	9.124320645	8.912084345	8.58709126	10337	Pgr	8.47727519	8.418419621	9.075818524	12637	Slc15a1	0.008383876	0.933569984	1.576874431	14937	Ube2z	8.147076793	8.512228699	8.333218758
1138	Agpat5	8.393806627	8.364421439	8.54608691	3438	Col5a1	3.090156402	3.941225855	4.152835241	5738	Gda	11.64016846	11.24354164	11.27461536	8038	Lpin3	1.066618477	1.87888762	2.308377397	10338	Pgrmc1	9.597844881	9.424750838	9.370137173	12638	Slc15a2	7.505668271	7.40864879	7.328705477	14938	Ube3a	10.86600941	10.79119471	10.88085577
1139	Agpat6	8.424536592	8.392785958	8.355036283	3439	Col5a2	3.255782418	3.75443349	2.783090159	5739	Gdap1	10.28018927	10.03824926	10.36156142	8039	Lpl	8.317825088	8.295105947	7.760982042	10339	Pgrmc2	9.545947989	9.46540418	9.547985255	12639	Slc15a3	7.21916405	7.558238177	7.274932872	14939	Ube3b	8.150760225	8.003788689	7.741342668
1140	Agpat9	4.874643903	3.729207741	4.15015076	3440	Col5a3	1.660628003	1.851447803	3.156453458	5740	Gdap10	2.091644822	2.414564585	3.685208988	8040	Lpo	1.056729647	1.894959793	2.31006762	10340	Pgs1	7.787977101	7.921189955	7.761295591	12640	Slc15a4	7.373088188	7.30850238	6.968432	14940	Ube3c	8.299234152	8.247739883	8.056663649
1141	Agps	7.327828193	7.140264309	7.208755306	3441	Col6a1	4.773700234	4.424960771	4.301624753	5741	Gdap1l1	8.355047495	8.067326076	7.985602591	8041	Lpp	6.836212773	6.627154808	7.119921843	10341	Phactr1	11.9574635	12.00370943	11.99226324	12641	Slc16a1	8.205072691	8.33797354	8.251401244	14941	Ube4a	6.865153614	7.477055006	7.414848663
1142	Agrn	7.906518453	7.669398596	7.774373155	3442	Col6a2	4.311907786	3.544231659	3.78927188	5742	Gdap2	7.7875363	7.757091374	7.687300397	8042	Lppos	4.190378574	5.263327376	5.535474103	10342	Phactr2	7.861165082	8.010604292	7.694189871	12642	Slc16a10	2.890572797	3.308419032	3.150585773	14942	Ube4b	8.978439747	8.979882815	9.114489261
1143	Agt	6.578691647	6.438155116	7.587722517	3443	Col6a3	3.883913269	4.020122017	4.230400269	5743	Gde1	9.865178403	9.874062331	9.8793515	8043	Lppr1	5.54098811	5.107946238	5.935234532	10343	Phactr3	9.797852031	9.557141779	9.557404011	12643	Slc16a11	5.288831014	4.728113989	4.836359128	14943	Ubfd1	6.312445884	6.606051053	6.57984116
1144	Agtpbp1	11.01795239	10.91981387	11.09568806	3444	Col6a4	1.050318425	0.894695535	2.311182876	5744	Gdf10	4.249179783	4.812996129	4.066866579	8044	Lppr2	7.472839504	7.692183218	7.431139489	10344	Phactr4	5.894277744	6.277755618	6.609803922	12644	Slc16a12	2.12821031	4.017178844	3.563888628	14944	Ubiad1	4.984090287	4.639898768	5.303914393
1145	Agtrap	5.514356043	5.94369245	6.129953187	3445	Col8a1	5.378117426	7.203544886	5.403421131	5745	Gdf11	6.20640549	5.88115264	5.404423287	8045	Lppr3	6.664676911	6.222910995	6.694502878	10345	Phax	9.250044264	8.916556671	9.124676325	12645	Slc16a13	3.652407182	3.869854937	3.150834762	14945	Ubl3	10.30113397	10.48529482	10.53937612
1146	Ahctf1	7.105249372	7.236193787	7.36358151	3446	Col8a2	4.941187015	6.205646545	4.368855465	5746	Gdf9	3.062582986	2.436423132	0.974628485	8046	Lppr4	10.76417099	10.69929305	10.23376528	10346	Phb	8.406555326	8.238249617	8.189329104	12646	Slc16a14	1.088403546	2.679113149	2.56884368	14946	Ubl4a	8.468394255	8.556447944	8.410429235
1147	Ahcy	5.505579022	5.326816184	5.227160375	3447	Col9a1	1.644837617	2.480585938	0.983609538	5747	Gdi1	10.42573158	10.19200624	10.13730602	8047	Lppr5	7.900573451	7.843203221	7.714493567	10347	Phb2	8.506794354	8.414682838	8.341787192	12647	Slc16a2	8.517079156	8.236524657	7.217294461	14947	Ubl5	10.80416143	10.75224609	10.70534544
1148	Ahcyl1	10.84054652	10.83764639	10.92615298	3448	Col9a2	5.744703195	4.960272248	5.06703239	5748	Gdi2	10.19298045	10.08672934	10.13605574	8048	Lpxn	2.38927033	2.449538479	2.792062132	10348	Phc1	7.010403386	6.903906855	7.046360343	12648	Slc16a3	4.157754331	4.32213054	3.564674425	14948	Ubl7	4.662256235	4.481484178	4.839480442

1149	Ahcyl2	9.199763052	9.454930258	9.352869693	3449	Col9a3	5.212650228	6.064329303	3.972629958
1150	Ahdc1	8.645284347	8.543533616	8.306426252	3450	Colec11	2.8714645	2.67017299	0.975366821
1151	Ahi1	11.1962944	10.86870005	11.27460812	3451	Colec12	4.661015376	4.072222168	5.070113873
1152	Ahnak	7.003016784	7.531639004	7.666726464	3452	Colgalt1	8.198535736	8.115049467	8.018936252
1153	Ahr	4.718084725	4.854069454	4.789157888	3453	Colgalt2	5.074127007	4.281695081	5.068988649
1154	Ahsa1	9.491676482	9.311563235	9.399994659	3454	Colq	0.029892849	2.229411779	1.988220069
1155	Ahsa2	9.914760068	9.786323961	9.769528445	3455	Commd1	7.011313383	6.913066469	6.665743347
1156	Ahsg	6.456309535	2.80895723	3.967643777	3456	Commd10	6.94972064	6.872252935	6.887036263
1157	AI115009	4.772342788	4.297699959	4.625356959	3457	Commd2	7.437646987	7.193037271	7.337485199
1158	AI118078	2.883130726	2.844883339	2.981587153	3458	Commd3	8.694668885	8.596554344	8.422842709
1159	AI197445	1.646463984	1.87897847	2.30838692	3459	Commd4	7.421112692	7.678723635	7.602144436
1160	AI314180	8.763657463	8.768405157	8.925519995	3460	Commd5	6.898913968	7.111342646	6.862992766
1161	AI317395	1.071991417	2.470820342	1.984520687	3461	Commd6	7.289432359	7.195173129	7.19913441
1162	AI413582	8.762564456	8.514046937	8.467818043	3462	Commd7	7.602122157	7.280502526	7.439647197
1163	AI414108	7.50657779	7.566920934	7.265053915	3463	Commd8	8.146365919	8.032130603	7.934008097
1164	AI429214	4.88646591	5.207879076	5.02528588	3464	Commd9	6.398390412	6.006936968	6.209290122
1165	AI450353	5.164064203	4.787327873	5.024793122	3465	Comt	9.46415631	9.456389506	9.352821053
1166	AI463170	0.024646119	1.90933525	1.573371446	3466	Comtd1	6.274950379	6.928902005	6.99180475
1167	AI464131	5.961229159	5.928198862	6.068242371	3467	Copa	9.333317115	9.365266158	9.315344364
1168	AI467606	2.078882333	1.82968823	0.974946793	3468	Copb1	8.832352149	8.857095542	9.155062651
1169	AI504432	7.41940154	7.053876287	7.363841112	3469	Copb2	9.269988362	9.141778887	9.157435573
1170	AI593442	9.403256509	9.790407071	10.05286149	3470	Cope	8.607950876	8.763761542	8.499551017
1171	AI597479	7.260582227	7.289209244	7.328802911	3471	Copg1	9.541974605	9.512038298	9.572471902
1172	AI607873	2.384402884	1.427968799	1.566307861	3472	Copg2	8.568610264	8.775346861	8.838126457
1173	AI662270	1.674019634	2.160879839	1.979331404	3473	Coprs	9.479056869	9.369249655	9.387266906
1174	AI837181	9.856669668	9.987289194	9.66473412	3474	Cops2	9.031225495	8.707584702	8.942971309
1175	AI839979	2.389241202	2.177635257	2.304771829	3475	Cops3	8.057416771	8.096349743	7.945740327
1176	AI846148	5.608495724	6.009780693	6.228803324	3476	Cops4	8.006515581	8.27671686	8.41892686
1177	AI854517	6.146522571	5.759305899	6.69566316	3477	Cops5	9.219740678	9.004935673	9.024351029
1178	AI854703	5.543155926	5.768647134	6.210141646	3478	Cops6	8.61191962	8.733438179	8.545989925
1179	AI987944	7.814646696	7.792966819	7.780990551	3479	Cops7a	8.442620299	8.376304421	8.495704315
1180	Aida	7.366113189	7.21930466	7.519351754	3480	Cops7b	6.910644809	6.605996922	6.761701958
1181	Aif1	6.678321158	6.635507767	6.283291398	3481	Cops8	9.184286154	9.55443713	9.308621844
1182	Aif1l	6.329734982	6.810313183	6.246240782	3482	Copz1	8.804330825	8.861914519	8.288065552
1183	Aifm1	7.446772894	7.628078942	7.431197695	3483	Copz2	3.786472299	4.910392338	2.973377039
1184	Aifm2	4.881381057	4.650096234	2.970046565	3484	Coq10a	6.419146057	6.514490205	6.472277703
1185	Aifm3	6.541902913	6.895751694	7.130538779	3485	Coq10b	5.916317029	6.024855029	6.440212486
1186	Aig1	7.412261583	7.488339689	7.76822432	3486	Coq2	7.723702435	7.721784866	7.708055843
1187	Aim1	4.150265014	3.663957775	1.553758324	3487	Coq3	6.961761533	6.894735515	6.748061968
1188	Aim1l	2.405344745	3.025609192	2.565027451	3488	Coq4	6.119657886	5.973810954	6.068585235
1189	Aim2	4.162159974	4.244035277	3.563359167	3489	Coq5	8.313846873	8.381995842	8.328808209
1190	Aimp1	9.631110077	9.5879191	9.363611882	3490	Coq6	5.751688371	5.865464119	5.708072649
1191	Aimp2	7.840003033	7.452126915	7.50357434	3491	Coq7	8.488761933	8.322638996	8.013208546
1192	Aip	9.738905567	9.979109615	9.904828924	3492	Coq9	7.259734446	7.177914359	7.26557453
1193	Aipl1	1.060796208	2.485688652	0.984596554	3493	Coro1a	7.219826395	7.644630889	7.237472819
1194	Airn	2.062096572	3.491948233	1.984202138	3494	Coro1b	8.085552186	8.053041767	8.165101262
1195	Ajap1	8.773885821	8.872109366	8.904931488	3495	Coro1c	8.683781125	8.63594956	8.568703373
1196	Ajuba	3.518541353	2.658793798	1.561005038	3496	Coro2a	7.078182435	6.668437017	6.899062766
1197	AK010878	6.081787571	6.248601734	6.20887235	3497	Coro2b	9.541567935	9.386940687	9.431255907
1198	Ak1	8.510897731	8.613509187	8.439381184	3498	Coro7	6.328852753	6.377280125	6.550316752

5749	Gdnf	2.379924313	1.867480367	2.307184883	8049	Lrba	7.012958114	7.238854304	7.49613303310349	Phc2	7.565878714	8.083391089	7.734746851
5750	Gdpd1	9.074434381	9.023345511	8.683733747	8050	Lrch1	4.677206734	5.262199857	5.33758920810350	Phc3	9.08596124	8.799681148	9.139943355
5751	Gdpd2	5.881365053	5.754048315	5.148902617	8051	Lrch2	7.0806415	6.90098681	7.21862515710351	Phex	1.680295564	3.977099019	1.977923091
5752	Gdpd3	2.409782334	4.051615054	3.568718086	8052	Lrch3	6.541426653	6.817353327	6.87521585610352	Phf1	6.778426649	6.855189242	6.565075812
5753	Gdpd5	5.425148821	4.846234953	4.564817541	8053	Lrch4	4.990822455	5.855628623	5.33809536310353	Phf10	6.25180699	6.727241752	6.503888226
5754	Gdpgp1	4.842189197	5.683267808	5.936551788	8054	Lrfn1	3.388136613	0.7369414	3.44313182910354	Phf11b	2.64462743	2.457679867	1.982654068
5755	Gem	2.081675268	2.15916246	2.790266028	8055	Lrfn2	7.174745969	6.928776506	7.01402899610355	Phf11c	1.091630462	2.445478704	2.568366946
5756	Gemin2	3.659227021	4.032419239	3.301656223	8056	Lrfn3	2.421061733	2.999452952	4.07141246710356	Phf11d	0.045318343	2.211485891	1.569744043
5757	Gemin4	4.594704279	4.305620676	4.373676877	8057	Lrfn4	5.539365973	5.324583165	5.59503785810357	Phf12	7.774203724	7.675706325	8.002602644
5758	Gemin5	6.145733777	6.233780048	6.265942805	8058	Lrfn5	4.606103031	4.210697153	5.2299443910358	Phf13	6.383216654	6.021779848	6.775611965
5759	Gemin6	4.338431218	5.500874956	4.787087701	8059	Lrg1	4.59137177	3.523072389	2.97454736910359	Phf14	8.85844001	8.756488917	8.800169783
5760	Gemin7	6.938160877	6.825376893	7.119985287	8060	Lrif1	7.808885357	8.05371541	7.83166605210360	Phf19	2.059569536	3.071775318	1.568554084
5761	Gemin8	4.17179248	4.151526229	3.978033057	8061	Lrig1	6.442073987	6.705706806	6.89895016210361	Phf2	7.665368611	7.53710823	7.887211174
5762	Gen1	2.865894811	2.45330121	1.565294231	8062	Lrig2	6.808812584	6.69317323	7.02474550310362	Phf20	9.29636898	8.890179206	8.922493215
5763	Get4	6.681792927	7.080277424	6.594170408	8063	Lrig3	4.991738443	5.943557223	5.22783840210363	Phf20l1	8.702685678	8.756876606	8.676915152
5764	Gfap	10.03611755	10.18378015	9.56667161	8064	Lrmp	2.076649697	3.202732026	1.56342058310364	Phf21a	8.419995753	8.216446259	8.467969113
5765	Gfer	6.588764721	6.671450642	6.638214193	8065	Lrp1	9.14935967	9.027048482	9.1698166910365	Phf21b	4.169989408	3.819126249	5.112647486
5766	Gfm1	6.823780083	7.124633276	7.311418967	8066	Lrp10	5.987396636	6.564330035	6.00240988710366	Phf23	8.630904411	8.324330366	8.523023113
5767	Gfm2	7.375783376	7.587452655	7.652690458	8067	Lrp11	8.539691177	8.507056147	8.5911262610367	Phf24	6.766761873	6.884755931	7.046729107
5768	Gfod1	9.162602969	9.11393115	9.260837868	8068	Lrp12	7.893517612	7.76884871	8.06253819610368	Phf3	9.991754855	9.712374113	9.883875857
5769	Gfod2	4.88709926	4.837620257	4.887067424	8069	Lrp1b	7.589756255	7.936817194	8.02460811610369	Phf5a	8.294125575	8.355409218	7.812513378
5770	Gfpt1	9.170851326	9.19073419	9.299685843	8070	Lrp3	7.9694777	8.114922627	7.99119611610370	Phf6	7.239434104	7.223172408	7.002148795
5771	Gfpt2	4.525579304	2.793009355	4.151937738	8071	Lrp4	6.589845104	6.792835424	6.68027704610371	Phf7	3.801912286	5.314620937	4.934980742
5772	Gfra1	8.831929015	8.624850066	8.503334088	8072	Lrp5	3.081670662	3.664866053	3.30274612110372	Phf8	6.987430937	6.891621107	7.448427439
5773	Gfra2	8.179257829	8.284496616	8.576390524	8073	Lrp6	7.61253728	7.964465567	7.7874382410373	Phgdh	7.766683521	8.015916093	7.422519642
5774	Gfra4	8.219059764	8.192721627	7.837777977	8074	Lrp8	7.604160635	7.840288087	7.87516516910374	Phip	9.603761027	9.178933344	9.493748459
5775	Gga1	6.938319373	6.892786871	6.922520247	8075	Lrpap1	9.424407766	9.326108361	9.09365306710375	Phka1	6.557003296	6.323678261	6.78770141
5776	Gga2	7.134348014	7.058674453	6.812587563	8076	Lrpprc	9.177577666	8.826600437	8.81919462510376	Phka2	5.532350515	4.877146849	5.067606726
5777	Gga3	7.5646987	7.523244243	7.72833387	8077	Lrrc1	6.050529298	5.726615525	4.93187289710377	Phkb	7.384500751	7.475279975	7.542438004
5778	Ggact	7.751942329	7.863300081	7.526807038	8078	Lrrc10b	8.142324269	8.70714101	8.27907452810378	Phkg1	4.992288621	5.363806095	5.265328992
5779	Ggct	7.587599344	7.510764159	7.487810194	8079	Lrrc14	7.610288541	7.575224842	7.25593875710379	Phkg2	7.034075296	7.078169195	6.72095791
5780	Ggcx	5.466768698	5.124930157	5.024195468	8080	Lrrc14b	5.397800112	4.874815339	5.62574426910380	Phlda1	7.446925879	7.614516977	7.572625071
5781	Ggh	7.099325362	6.950837272	6.721535592	8081	Lrrc16a	5.813152774	5.897247266	6.50440772510381	Phlda3	6.102541516	5.660292154	6.407174517
5782	Ggn	0.055329985	2.895985802	1.568159356	8082	Lrrc16b	4.465077129	4.306730571	4.22877957510382	Phldb1	8.419792406	8.598451514	8.129590756
5783	Ggnbp1	3.527155515	4.048205281	2.563302018	8083	Lrrc17	18.14535866	17.87793492	17.852990310383	Phldb2	6.361138891	5.714470966	6.088929242
5784	Ggnbp2	10.75115956	10.69271039	10.63907884	8084	Lrrc18	3.973337262	4.401023918	3.56664793810384	Phlpp1	8.37043826	8.43849811	8.31087875
5785	Ggnbp2os	4.468319683	4.491026017	3.67747501	8085	Lrrc2	4.98364108	4.345788615	4.93490694410385	Phlpp2	7.556442965	7.243044599	7.787631577
5786	Ggps1	8.351532603	8.145461939	8.398000394	8086	Lrrc20	6.574049703	7.022291224	6.95772092510386	Phospho1	4.175536563	5.141933003	4.681006565
5787	Ggt1	0.083436947	3.475720987	2.568405856	8087	Lrrc23	5.713213027	5.050513456	5.10992143610387	Phospho2	8.25477101	7.913026606	8.174830164
5788	Ggt5	3.073821377	2.407275258	2.299399815	8088	Lrrc24	7.277751416	6.801010351	7.2188837110388	Phox2a	6.384622707	6.763264763	6.624367732
5789	Ggt6	1.040696725	1.502480606	0.989761303	8089	Lrrc26	7.533950101	8.129684297	8.15517586110389	Phpt1	9.113616215	8.881747972	8.999592436
5790	Ggt7	5.244643624	5.310189619	4.734218524	8090	Lrrc27	4.460163954	3.640099814	4.06892217510390	Phrf1	8.2698221	8.182472263	8.346519621
5791	Ggta1	4.244948521	3.923736322	3.786454004	8091	Lrrc28	6.168809164	6.158008352	6.00166459110391	Phtf1	7.401763268	7.330111813	7.283742485
5792	Gh	7.28834519	11.09826878	6.797490507	8092	Lrrc3	4.257287099	4.845977015	4.98156422810392	Phtf1os	6.901690002	6.987136802	7.150167877
5793	Ghdc	5.07341509	4.350220699	4.300246504	8093	Lrrc32	2.890090105	3.868948959	3.15069652310393	Phtf2	6.099560226	5.640102979	6.04673498
5794	Ghitm	10.68241518	10.66021072	10.80335736	8094	Lrrc34	3.228678296	3.59015308	2.98443523410394	Phxr4	1.097564493	1.829759877	2.567497795
5795	Ghr	4.897389216	5.482485775	5.50373009	8095	Lrrc38	3.06905175	2.419704947	2.98239421810395	Phyh	8.544224208	8.425488033	8.634662632
5796	Ghsr	0.060363003	2.195685624	2.306867354	8096	Lrrc39	4.988723412	5.125065222	4.67871972510396	Phyhd1	4.794636885	6.314547404	4.931544747
5797	Gid4	8.285089484	8.301559184	8.406377329	8097	Lrrc3b	5.807939818	5.839607116	5.93470996510397	Phyhip	9.653704496	9.663759868	9.38724766
5798	Gid8	9.077084994	8.809187709	9.010766882	8098	Lrrc4	8.990514866	8.997624141	9.17730263510398	Phyhipl	9.817025865	9.826426303	9.861100537

12649	Slc16a4	3.090255056	4.026466266	4.569406721	14949	Ublcp1	7.657776363	7.366174487	7.687437265
12650	Slc16a6	7.462189391	7.323752038	7.056588542	14950	Ubn1	9.1886109	9.078990907	8.901759331
12651	Slc16a7	4.728467596	5.405807062	5.228990609	14951	Ubn2	9.539976025	9.594608863	9.509405587
12652	Slc16a9	4.541408044	5.38444264	4.935507647	14952	Ubox5	5.081288815	5.197524819	4.785874715
12653	Slc17a2	1.052462744	0.890010278	1.572395003	14953	Ubp1	8.497924962	8.171705629	8.587511133
12654	Slc17a5	7.17655478	6.881530408	7.160355783	14954	Ubqln1	9.858777847	9.918555629	10.14244013
12655	Slc17a6	8.187377359	8.502144404	9.8566603	14955	Ubqln2	9.598188846	9.541615712	9.687392901
12656	Slc17a7	10.59496021	10.62310259	10.19062393	14956	Ubqln3	0.040178806	3.504215888	0.984613207
12657	Slc17a8	4.083779743	4.36569518	3.677370573	14957	Ubqln4	8.275234969	8.427841451	8.488002652
12658	Slc18a2	5.509128895	5.910029587	5.958195426	14958	Ubr1	8.264928849	7.981041176	8.184594888
12659	Slc18a3	0.078767727	2.877766247	3.308395686	14959	Ubr2	8.825603494	8.808894024	8.980040572
12660	Slc18b1	5.53785348	5.678454108	5.066137006	14960	Ubr3	8.97558627	8.88410336	9.184634901
12661	Slc19a1	4.946260453	5.188834125	5.535048392	14961	Ubr4	9.629299504	9.697747241	9.652440534
12662	Slc19a2	5.664507198	5.288304164	6.047512669	14962	Ubr5	8.904160409	8.992668497	9.167448431
12663	Slc19a3	3.375363645	1.413463722	0.977344575	14963	Ubr7	7.411419634	7.423041137	7.503706758
12664	Slc1a1	8.255409576	8.221408333	8.3332278	14964	Ubtd1	5.244560522	5.376295976	4.98040728
12665	Slc1a2	14.29553498	14.26884456	14.37851847	14965	Ubtd2	7.499942748	7.637428861	7.748154624
12666	Slc1a3	11.6260378	11.61666638	11.57102646	14966	Ubtf	8.838550565	8.880153552	8.844231499
12667	Slc1a4	7.586922145	7.377110099	7.455861795	14967	Ubxn1	9.772481309	9.946524918	9.880850187
12668	Slc1a5	1.670510474	2.869228027	2.984416856	14968	Ubxn10	3.883350163	4.781405159	3.14701911
12669	Slc1a6	5.751551763	5.591118472	5.471204067	14969	Ubxn11	3.662330403	4.256521138	2.976929948
12670	Slc20a1	9.309637631	9.183345508	9.652512883	14970	Ubxn2a	7.816955908	7.874153952	8.46409422
12671	Slc20a2	7.513259875	7.347142563	6.990758669	14971	Ubxn2b	7.873747139	7.557840673	7.55727301
12672	Slc22a1	0.055121064	1.444290726	0.981719169	14972	Ubxn4	10.08460384	9.968427611	9.695849811
12673	Slc22a12	1.647988104	2.900154404	1.568939742	14973	Ubxn6	8.151094077	7.992984626	7.862513174
12674	Slc22a15	4.472347271	4.851312897	4.736133396	14974	Ubxn7	8.086901666	8.112837137	8.488007407
12675	Slc22a17	11.18518354	11.28808671	11.11567786	14975	Ubxn8	10.37152868	10.26213524	10.25146294
12676	Slc22a18	1.657897514	2.459808057	0.979481983	14976	Uchl1	11.79347087	11.82449637	11.56868601
12677	Slc22a2	4.52989025	2.958683018	4.150746106	14977	Uchl1os	2.674372534	3.306500701	4.071872959
12678	Slc22a21	2.869990195	2.170892935	1.563663541	14978	Uchl3	7.319667791	7.467736728	7.587570109
12679	Slc22a23	7.671440863	7.278938348	7.389324309	14979	Uchl4	6.060619091	6.228692929	6.389625507
12680	Slc22a3	3.247654319	2.824572416	4.232716767	14980	Uchl5	8.60387824	8.303828643	8.718228604
12681	Slc22a30	0.016610929	1.5024554	0.989757904	14981	Uck1	7.346624199	7.556035448	7.098659597
12682	Slc22a4	4.07467285	3.743202152	3.887425566	14982	Uck2	5.159266229	5.020414452	4.838868305
12683	Slc22a5	3.888440643	3.12290971	4.229373458	14983	Uckl1	6.104863315	6.486087328	6.087990548
12684	Slc22a6	5.688651865	4.727489239	5.566151106	14984	Uckl1os	5.860282089	6.113614748	5.911276297
12685	Slc22a8	6.554461476	6.117549377	6.188917441	14985	Ucma	4.068655341	4.25891274	2.559544823
12686	Slc23a1	1.071963032	2.470857625	1.568174854	14986	Ucp2	5.174188031	5.97757498	5.438967546
12687	Slc23a2	7.524983888	7.916010971	7.922684138	14987	Ucp3	2.892220628	3.43594972	4.154534859
12688	Slc24a1	2.632030435	0.871464538	1.986611439	14988	Uevld	4.665880353	4.589160105	5.110981182
12689	Slc24a2	11.52251004	11.53124777	11.69037064	14989	Ufc1	8.442345032	8.489507282	8.503640419
12690	Slc24a3	10.24166857	10.14713544	10.27462125	14990	Ufd1l	8.916511839	8.938794543	8.963033968
12691	Slc24a4	1.634635111	1.901918817	2.310802325	14991	Ufl1	8.433457658	8.535974746	8.491850749
12692	Slc24a5	5.367911646	6.100749288	5.439208325	14992	Ufm1	8.676392414	8.470157089	8.648934193
12693	Slc25a1	6.588859564	6.522101423	6.51885066	14993	Ufsp1	5.661704869	5.327564455	5.681413155
12694	Slc25a10	4.728925256	5.127812375	4.622237049	14994	Ufsp2	8.680545185	8.483914034	8.711310811
12695	Slc25a11	8.88457497	8.928393839	8.8988864	14995	Ugcg	7.920112405	7.919321278	7.996801139
12696	Slc25a12	9.450838001	9.323916489	9.098833686	14996	Ugdh	6.455586502	6.422130727	6.838863764
12697	Slc25a13	5.690133661	5.731233437	5.301788518	14997	Uggt1	8.28891233	7.789646916	7.968582809
12698	Slc25a14	8.551671653	8.361108999	8.455769448	14998	Uggt2	5.541884792	5.590437055	6.003399346

1199	Ak2	5.837210353	5.857343081	6.26623367	3499	Cort	4.920987753	4.503677771	3.145695357	5799	Gigyf1	7.580459499	7.665227698	7.714874144	8099	Lrrc40	7.51391623	7.111014528	7.283696332	10399	Phykpl	4.159164388	4.019779771	3.981353689	12699	Slc25a15	6.012139884	5.899558921	5.95688945	14999	Ugp2	8.91983902	8.903697188	8.999610722
1200	Ak3	8.759959682	8.829902529	8.655881877	3500	Cotl1	9.170516119	9.275297189	8.880708709	5800	Gigyf2	9.330490314	9.266607226	9.109231743	8100	Lrrc41	5.755812295	6.218546322	6.302870202	10400	Pi15	2.394909989	1.829804062	2.5675019	12700	Slc25a16	6.606338218	7.118250353	7.150523422	15000	Ugt1a6b	4.539654823	3.986081745	4.371587486
1201	Ak4	8.558595057	8.08055926	7.850022707	3501	Cox10	8.11874672	8.082051059	7.939956112	5801	Gimap1	3.070482133	0.743521434	3.443406575	8101	Lrrc42	7.64268454	7.585599771	7.714824606	10401	Pi16	1.045658334	1.493104076	1.989547817	12701	Slc25a17	7.79545111	8.125103631	8.007998314	15001	Ugt2b34	1.072039177	1.444005311	2.307465539
1202	Ak5	7.867006207	7.619279407	7.53446076	3502	Cox11	6.822733937	7.145311802	6.679935982	5802	Gimap5	1.05673002	1.472385753	1.571462962	8102	Lrrc43	1.632745375	2.725847411	1.572874316	10402	Pi4k2a	8.279955968	8.293780335	8.242108293	12702	Slc25a18	4.827199194	4.655464587	3.978060509	15002	Ugt2b35	0.019283077	0.910119198	0.989116552
1203	Ak6	7.595179375	7.511288586	7.380790839	3503	Cox14	9.742100736	9.659780423	9.559279854	5803	Gimap6	3.400236897	3.427763366	3.30193465	8103	Lrrc45	5.171466955	5.513827313	5.264922961	10403	Pi4k2b	3.656511921	2.820404656	2.784973579	12703	Slc25a19	7.058579443	7.204873965	7.337862855	15003	Ugt8a	8.657598023	8.649077455	8.274361905
1204	Ak7	5.284662787	5.341415214	5.02468162	3504	Cox15	7.279164709	7.299531863	7.293149659	5804	Gimap8	3.38353884	3.332307795	1.978137324	8104	Lrrc46	2.890540886	4.123763933	3.567573671	10404	Pi4ka	9.494053632	9.331315313	9.519188359	12704	Slc25a2	0.021965291	1.913282241	1.573894184	15004	Uhmk1	7.176859232	7.078036107	7.002437702
1205	Ak8	3.885200497	4.383585868	4.068945622	3505	Cox16	8.662125763	8.753571068	8.80973131	5805	Gimap9	2.393956458	1.832234253	2.567727904	8105	Lrrc47	8.35195941	8.263546628	8.376628826	10405	Pi4kb	6.309774916	6.570165241	6.389490768	12705	Slc25a20	6.057622149	6.216820225	6.088652233	15005	Uhrf1	2.856605361	1.450650085	1.568929098
1206	Akap1	6.539679349	6.566835928	6.721955586	3506	Cox17	9.536063538	9.500940412	9.578014226	5806	Gin1	5.600312243	5.425169493	5.06634526	8106	Lrrc48	5.661145575	6.161550013	5.653444473	10406	Pianp	9.523356453	9.506930985	9.711424204	12706	Slc25a21	2.073571308	2.677304943	1.981223066	15006	Uhrf1bp1	5.472150082	5.326764732	5.337209272
1207	Akap10	5.253109879	5.188299092	5.814207809	3507	Cox18	6.265912437	6.202473488	5.73434777	5807	Ginm1	6.589586232	6.793335096	6.911142974	8107	Lrrc49	8.785018182	8.755501275	9.192004768	10407	Pias1	8.028930971	8.124055861	8.28860543	12707	Slc25a22	9.013903223	9.098640696	8.787268376	15007	Uhrf1bp1l	9.146704249	9.210881021	9.218418108
1208	Akap11	11.00094453	10.93307875	11.09373575	3508	Cox19	6.884700756	6.633938468	6.594820807	5808	Gins1	3.231384975	2.159044488	2.98369533	8108	Lrrc4b	8.831761495	8.921111141	8.767923191	10408	Pias2	8.519270884	8.37223827	8.704672716	12708	Slc25a23	10.8528463	10.90876023	10.86721558	15008	Uhrf2	7.382124746	6.51474528	7.209038161
1209	Akap12	8.205559466	8.166808431	8.666694564	3509	Cox20	9.313146866	9.047970861	8.728046923	5809	Gins2	7.317778995	7.340189705	6.956880933	8109	Lrrc4c	9.970697378	9.775623244	10.04882114	10409	Pias3	6.913164199	6.848527506	7.013680179	12709	Slc25a24	5.158239021	4.478498152	4.935738027	15009	Uimc1	7.375177323	7.287907126	7.246554586
1210	Akap13	7.765020897	7.430244857	7.283518458	3510	Cox4i1	11.56841474	11.60395295	11.52735347	5810	Gins3	3.904031031	4.697306691	4.887875201	8110	Lrrc51	3.691837472	5.794973475	4.501987685	10410	Pias4	4.884293222	5.137726998	4.981244549	12710	Slc25a25	7.327628195	6.994569137	7.265487394	15010	Ulk1	8.193884861	8.173833678	8.218516365
1211	Akap14	5.20692275	5.495262409	4.979785544	3511	Cox4i2	3.077601048	3.671800789	3.150963289	5811	Gins4	6.536346202	6.380797528	6.109006524	8111	Lrrc55	6.151406771	7.381815651	7.321347188	10411	Pibf1	6.873316574	6.535377428	6.680399289	12711	Slc25a26	6.667373451	6.692783471	6.886912187	15011	Ulk2	8.818607114	8.729190413	8.960234747
1212	Akap17b	7.912449572	7.637754605	7.800260829	3512	Cox5a	10.06074162	10.09640947	9.941385235	5812	Gipc1	6.456457906	7.07576079	6.565008912	8112	Lrrc56	3.654471254	3.956138824	3.683231533	10412	Picalm	9.527507565	9.454365119	9.433243658	12712	Slc25a27	5.543918878	5.717796633	6.110151058	15012	Ulk3	5.399071957	5.121368878	5.53587557
1213	Akap2	9.358920325	9.436354787	9.344133771	3513	Cox5b	11.09738382	10.98931493	11.12081525	5813	Gipc2	3.073828779	2.640091567	4.15576372	8113	Lrrc57	6.587584748	6.389993501	6.609579418	10413	Pick1	6.589553746	6.928908036	6.60909834	12713	Slc25a28	7.446069426	7.416405677	7.380954762	15013	Ulk4	3.088669835	3.541692824	4.376340697
1214	Akap3	2.120495567	5.877641814	3.300554677	3514	Cox6a1	12.23762121	12.25299962	12.16917974	5814	Git1	9.722417112	9.782363683	9.68737659	8114	Lrrc58	11.23410114	11.18153207	11.22196932	10414	Pid1	7.418568633	7.26552909	7.045855432	12714	Slc25a29	5.253012211	5.91696694	5.372145878	15014	Umad1	5.726856136	5.764923307	6.130398834
1215	Akap4	0.230585515	6.003196334	3.886933241	3515	Cox6a2	6.365753623	6.262291036	6.610001215	5815	Git2	6.506854561	6.374271718	6.666861455	8115	Lrrc59	7.71707226	7.72072017	7.659412101	10415	Piezo1	5.780947096	6.187312487	5.564210934	12715	Slc25a3	11.03765269	10.90577659	11.0030429	15015	Umodl1	1.048003657	1.488694483	1.98908941
1216	Akap5	18.13333255	17.86158795	17.84039301	3516	Cox6b1	11.4130803	11.18145574	11.11117313	5816	Gja1	9.946447843	10.04481589	9.901773694	8116	Lrrc6	6.452980093	6.516412881	6.169681015	10416	Piezo2	3.528103981	3.562096348	2.29831166	12716	Slc25a32	4.097622029	5.375063965	4.934249984	15016	Umps	5.469182282	5.041339681	4.93296072
1217	Akap6	10.08069774	10.0591117	10.48488769	3517	Cox6b2	5.701923112	7.072717774	6.488509783	5817	Gja3	1.639929427	3.238882336	1.571022905	8117	Lrrc61	7.781452245	8.29699282	7.825423381	10417	Pifo	3.249370214	4.843107341	2.784983362	12717	Slc25a33	7.262490597	7.703529631	7.572448701	15017	Unc119	7.0331438	6.871713467	6.623346797
1218	Akap7	9.209673831	8.955900514	9.235069007	3518	Cox6c	11.91386217	11.86428867	11.84261187	5818	Gja4	2.892603464	3.002855744	3.302891824	8118	Lrrc7	8.387762648	8.127900731	8.359609721	10418	Piga	5.746521124	5.22808455	5.023279628	12718	Slc25a34	5.468527526	5.082503856	5.338123107	15018	Unc119b	5.724524065	5.908299117	5.78751969
1219	Akap8	7.187425496	6.902711868	7.056955353	3519	Cox7a1	4.715114018	4.081306986	3.785206942	5819	Gjb1	6.683799661	7.114666222	7.129852925	8119	Lrrc71	5.838783315	6.267803812	6.088479135	10419	Pigb	8.068876	8.129059398	8.046137215	12719	Slc25a35	4.176097555	4.980220963	4.839085793	15019	Unc13a	8.32368115	8.438685852	8.534782533
1220	Akap8l	7.828519941	7.872120353	7.862728242	3520	Cox7a2	11.77009617	11.60633758	11.58822605	5820	Gjb2	4.552661596	4.727550788	5.189272713	8120	Lrrc73	8.151324033	8.316214062	7.837766336	10420	Pigc	7.020877645	6.400814269	6.694064939	12720	Slc25a36	3.267935368	3.395598935	4.441368507	15020	Unc13b	6.650068906	6.449361624	6.549684082
1221	Akap9	10.84639194	10.91115375	11.06280679	3521	Cox7a2l	10.17986337	10.08085156	10.18456863	5821	Gjb6	8.309810936	8.097459915	8.45575076	8121	Lrrc74b	2.671865802	4.471510845	2.980081442	10421	Pigf	6.208993548	5.898224266	6.372949201	12721	Slc25a37	6.959348714	6.223607183	6.439157612	15021	Unc13c	8.465695517	8.264268135	9.333384128
1222	Akip1	4.715268972	3.822166077	4.62570942	3522	Cox7b	10.50785431	10.2709813	10.34298769	5822	Gjc1	4.728303301	4.879987099	5.30329846	8122	Lrrc75a	5.568582586	5.573097854	6.132361383	10422	Pigg	2.422430554	1.309121351	4.686287073	12722	Slc25a38	6.363115995	6.317987046	6.088434807	15022	Unc13d	1.068399732	2.20606542	0.982626032
1223	Akirin1	7.411608554	7.518364312	7.624136142	3523	Cox7b2	2.407859843	2.64390098	2.787982247	5823	Gjc2	7.299739384	7.803808281	7.237261861	8123	Lrrc75b	6.724108665	6.757846752	6.838291198	10423	Pigh	7.135957347	7.395735659	7.045875766	12723	Slc25a39	8.790851092	8.733269729	8.418596734	15023	Unc45a	5.082588886	5.459867183	4.885529716
1224	Akirin2	9.173289585	9.41444486	9.167340614	3524	Cox7c	10.84707093	10.71494515	10.71974474	5824	Gjc3	7.532035433	7.758721117	7.572438874	8124	Lrrc8a	7.604254358	7.181050239	7.813133155	10424	Pigk	8.244842274	8.036920261	8.204094716	12724	Slc25a4	12.38751772	12.31724619	12.33807373	15024	Unc45b	2.083506973	3.464125724	2.789907389
1225	Akna	2.872152356	3.199827418	2.984252171	3525	Cox8a	12.27932935	12.26011728	12.1694886	5825	Gjd2	4.843063989	4.868638074	5.839869299	8125	Lrrc8b	2.384360138	2.459539478	1.566322157	10425	Pigl	1.085321086	2.181948669	2.305271631	12725	Slc25a40	4.886392067	5.095083391	4.623046356	15025	Unc50	7.794384581	7.883910212	7.806651206
1226	Aknad1	0.013867802	2.251278153	0.990440272	3526	Cox8b	4.161074573	4.56437565	3.146129983	5826	Gk5	1.655245786	2.193443909	2.306606561	8126	Lrrc8c	3.78847369	3.824837195	4.566842901	10426	Pigm	5.982693131	5.287448903	6.068850024	12726	Slc25a42	5.433610158	4.331112204	5.302529875	15026	Unc5a	7.391966282	7.177553682	7.159846177
1227	Akr1a1	9.610042867	9.728386834	9.61660028	3527	Cp	7.318448243	6.687130773	7.088221346	5827	Gkap1	8.056971827	7.936371559	7.837909127	8127	Lrrc8d	7.363393894	7.342212677	7.046119212	10427	Pign	5.365116	5.486670821	5.681724784	12727	Slc25a44	9.031804517	9.027711527	8.94283363	15027	Unc5b	8.140516801	8.262285849	8.002374144
1228	Akr1b10	6.187662493	5.854988327	5.91023482	3528	Cpa2	3.656702983	2.819885075	2.784909401	5828	Gkn3	4.322798048	2.955835495	2.555036932	8128	Lrrc9	0.143905895	4.046687821	3.151111552	10428	Pigo	5.248298517	5.161203159	5.654345717	12728	Slc25a45	3.779334173	3.410158857	3.88778779	15028	Unc5c	8.687186774	8.448597794	8.410369937
1229	Akr1b3	6.60231176	6.775050026	6.652966343	3529	Cpa4	1.04321999	1.917360053	1.574433628	5829	Gla	6.565839161	5.923992582	6.109663934	8129	Lrrcc1	7.809694936	7.875118547	8.274845448	10429	Pigp	6.782480987	7.008632743	6.874643069	12729	Slc25a46	8.893688516	8.689619236	8.687347648	15029	Unc5cl	1.032340788	0.933566573	0.991897432
1230	Akr1b8	3.218192074	1.867302971	2.794100196	3530	Cpb2	3.97475392	2.376878984	3.566245701	5830	Glb1	5.83758058	6.068180429	5.651257512	8130	Lrrd1	3.392053252	2.836161788	2.980576777	10430	Pigq	8.446419526	8.511574921	8.194170703	12730	Slc25a47	3.528626157	3.441407268	3.303669282	15030	Unc5d	7.317067047	6.73947357	7.488521262
1231	Akr1c14	1.068381188	1.450770719	1.568943106	3531	Cpd	9.085554232	8.938923452	8.850344321	5831	Glb1l	5.465275894	4.463141451	5.266535396	8131	Lrrfip1	8.182550468	7.761147707	7.844085685	10431	Pigr	3.093892679	2.070868308	4.568822715	12731	Slc25a5	9.475162116	9.632541484	9.561192122	15031	Unc79	7.58034832	7.436140874	8.052089639
1232	Akr1c18	5.326445905	5.195262894	5.936663522	3532	Cpe	13.40236701	13.31502439	13.11664069	5832	Glb1l2	3.771203942	3.548876554	2.978361285	8132	Lrrfip2	7.900465765	7.871480681	7.916709053	10432	Pigs	7.237920684	7.059113886	6.922499346	12732	Slc25a51	9.784051483	9.525984433	9.507441093	15032	Unc80	10.15200651	9.948490582	10.34746157
1233	Akr1e1	7.421056863	7.390830789	7.623994476	3533	Cpeb1	8.591654592	8.426645783	8.367961109	5833	Glcci1	8.005068943	7.831101516	8.046195363	8133	Lrriq1	7.066969405	7.583799386	6.47106493	10433	Pigt	7.219814765	7.418445898	7.346598591	12733	Slc25a53	3.776006113	3.849839657	4.56935169	15033	Unc93b1	5.915591049	6.335108689	6.109338464
1234	Akr7a5	7.693619973	7.587914145	7.406211897	3534	Cpeb2	8.275634877	8.267630117	8.561373985	5834	Glce	6.350785394	6.56349666	6.969282106	8134	Lrriq3	2.869074614	2.874220292	3.155516257	10434	Pigu	5.209927166	4.569905311	5.625303753	12734	Slc26a1	3.064592675	3.037485393	1.978883871	15034	Uncx	2.897491926	3.296039806	4.153649186
1235	Akt1	10.09454459	10.08103979	10.18703738	3535	Cpeb3	9.194476075	9.157017508	9.453786776	5835	Gldc	5.574209942	5.794432466	5.787629144	8135	Lrrk1	4.310412234	3.297143522	2.978244818	10435	Pigv	4.244235754	4.670601041	3.298396841	12735	Slc26a10	1.085545487	2.45323866	1.565283899	15035	Ung	3.790581914	4.711969278	4.504756596
1236	Akt1s1	4.257153933	4.646938916	4.788177245	3536	Cpeb4	9.389070375	9.376625484	9.697763397	5836	Gldn	2.877194904	3.190406631	0.972408201	8136	Lrrk2	6.289646356	5.895468635	6.535293848	10436	Pigw	4.077575314	4.616208339	3.786607423	12736	Slc26a11	3.264475239	3.125402188	4.683902693	15036	Unk	5.660459884	5.33015575	5.535380095
1237	Akt2	8.113650983	8.037457393	8.119623098	3537	Cped1	4.257419123	4.592076277	4.83895437	5837	Gle1	7.249301667	7.258014964	7.07798751	8137	Lrrn1	6.914685146	7.027084925	6.886519241	10437	Pigx	6.585148979	6.21133138	6.246674977	12737	Slc26a2	4.465365824	4.954202428	3.297524514	15037	Unkl	7.464596538	7.566332747	7.557476037
1238	Akt3	10.40240813	10.28481761	10.45372375	3538	Cplx1	11.47183961	11.38215839	11.62204679	5838	Glg1	9.615939055	9.697441206	9.70109562	8138	Lrrn2	9.059810217	9.006633493	8.545875893	10438	Pigyl	8.016945804	8.119981237	7.886780219	12738	Slc26a3	1.647958336	2.475605528	0.982636106	15038	Uox	3.647879904	3.450626328	1.558075104
1239	Aktip	9.815151501	9.698780507	9.711291512	3539	Cplx2	12.11878958	12.12324458	11.89397778	5839	Gli1	3.534118055	3.155477986	3.789901523	8139	Lrrn3	9.9477416	9.824930019	9.909253313	10439	Pigz	4.886868033	5.277839367	4.679767654	12739	Slc26a4	6.054754476	6.168101423	4.930665009	15039	Upb1	1.666778338	3.049320416	1.980958164
1240	Alad	6.677373717	6.278789145	6.354976958	3540	Cplx3	6.329471313	7.135578419	6.51939411	5840	Gli2	2.673804059	3.867401327	3.567457749	8140	Lrrn4	2.885676588	3.447174026	3.442500232	10440	Pih1d1	6.326960358	6.177321065	6.001789362	12740	Slc26a6	1.040697542	1.921610727	0.989761094	15040	Upf1	8.687493182	8.555410791	8.613015876
1241	Alas1	7.807942557	7.577825647	7.940285782	3541	Cpm	6.93770404	7.000247286	6.761169905	5841	Gli3	6.122738089	6.00930564	6.067823399	8141	Lrrn4cl	1.646480337	0.858739035	1.569330099	10441	Pih1d2	3.888732882	5.122982921	4.506096937	12741	Slc26a7	4.066765012	2.992689029	4.444163783	15041	Upf2	9.580255442	9.386225332	9.503548191
1242	Alas2	4.339383755	4.926274312	4.369816106	3542	Cpn1	1.659224396	2.884649355	2.569788124	5842	Glipr1	4.660489666	4.360394942	4.372245598	8142	Lrrtm1	8.906524348	8.747917008	8.557288573	10442	Pik3ap1	2.37764408	2.203473278	2.794617061	12742	Slc26a8	3.646443941	3.324073817	3.443305684	15042	Upf3a	7.523312535	7.640435223	7.715020892
1243	Alb	9.286207414	7.580773842	10.28046255	3543	Cpne1	6.016920789	6.832413889	6.47175466	5843	Glipr2	4.721447947	4.847460257	4.735646936	8143	Lrrtm2	8.968625861	8.783518686	8.910845676	10443	Pik3c2a	7.605478895	7.694238167	8.124975571	12743	Slc27a1	4.608468411	5.277309943	5.26703766	15043	Upf3b	8.944398891	8.719879296	8.825475894
1244	Alcam	10.78957649	10.54749781	10.9144545	3544	Cpne2	7.528976372	7.500939089	6.874291796	5844	Glis2	4.326622726	4.65988097	4.504779945	8144	Lrrtm3	7.506815775	7.421773753	7.781283593	10444	Pik3c2b	5.719560327	5.328104087	5.565611357	12744	Slc27a2	4.004591117	2.90820555	5.02525183	15044	Upk1a	2.361284106	0.904749535	0.98848154
1245	Aldh16a1	3.879931597	4.027953715	3.88884893	3545	Cpne3	7.365550867	7.254987362	7.389514627	5845	Glis3	4.600982741	4.358568212	3.884022392	8145	Lrrtm4	6.832804352	6.223381783	6.564973818	10445	Pik3c3	7.024791046	7.289586186	7.550335513	12745	Slc27a3	2.689766217	4.321037052	3.564508008	15045	Upk1b	2.649165897	2.875871078	2.568610331
1246	Aldh18a1	4.660934539	4.756461294	4.98235364	3546	Cpne4	9.02864378	9.276237239	8.522719571	5846	Glmn	6.65204815	6.602024347	7.078527428	8146	Lrsam1	7.16727428	7.142653884	7.398421388	10446	Pik3ca	9.361371236	9.311263172	9.598475935	12746	Slc27a4	7.808256327	7.958665264	7.819257285	15046	Upp1	2.68723148	4.572338932	2.976625498
1247	Aldh1a1	7.607000729	7.607263725	7.979762866	3547	Cpne5	8.178449128	8.195790959	8.511729402	5847	Glmp	6.845869443	6.357708074	6.422626802	8147	Lrtm1	3.059706445	2.673775558	2.304036093	10447	Pik3cb	6.938730335	7.028691012	7.06765778	12747	Slc29a1	5.334182758	6.224969368	5.470985194	15047	Upp2	5.717797084	5.33180757	4.978836747
1248	Aldh1a2	4.543537898	4.587430132	5.408815983	3548	Cpne6	10.41211812	10.2143987	9.49548212	5848	Glo1	8.759586207	8.585802179	8.690847656	8148	Lrtm2	7.843249997	8.076245185	8.452049247	10448	Pik3cd	6.971874966	6.565307934	6.518974893	12748	Slc29a2	5.540262864	5.565889097	6.047626831	15048	Uqcc1	7.999767835	8.090653519	8.15999071

1249	Aldh1a7	0.060069548	2.892136463	0.980796839	3549	Cpne7	8.382360189	7.61735954	5.809293293	5849	Glod4	9.346776608	9.254238927	9.211268063	8149	Lrwd1	4.667176186	5.056776588	4.29970320810449	Pik3cg	3.880075496	4.455932243	3.439181024	12749	Slc29a3	6.950251667	6.882385824	6.980207741	15049	Uqcc2	9.250299444	9.219586886	9.05673279
1250	Aldh1b1	5.981219056	5.538896099	5.336873187	3550	Cpne8	6.973903082	6.990599539	6.77433531	5850	Glp2r	3.511887183	3.206955255	1.981197526	8150	Lsamp	6.20890337	5.853549354	6.24710350110450	Pik3ip1	7.420615951	7.407128788	7.565122117	12750	Slc29a4	5.782950914	6.201604079	5.186687449	15050	Uqcc3	7.337815875	7.220202517	7.337568375
1251	Aldh1l1	6.168370948	6.158862229	6.519989277	3551	Cpne9	5.972475451	6.990341035	7.238199295	5851	Glra1	4.803918084	4.155865894	6.900542733	8151	Lsg1	6.349036037	6.421069213	6.54994426810451	Pik3r1	9.838818285	9.84621773	10.05954057	12751	Slc2a1	7.939077397	7.929498286	7.60147152	15051	Uqcr10	10.5792119	10.19607054	10.30188959
1252	Aldh1l2	5.402476488	5.423349533	5.535050154	3552	Cpox	8.555537042	8.349538992	8.237257421	5852	Glra2	5.980317849	5.801320611	5.188426659	8152	Lsm1	6.71125046	6.692974625	7.02472015510452	Pik3r2	8.129576276	8.003171289	8.099092612	12752	Slc2a10	2.642791945	2.190972905	3.157053635	15052	Uqcr11	9.632815224	9.526474191	9.365820041
1253	Aldh2	9.034908343	9.182533658	9.062114709	3553	Cpped1	8.074289061	8.033387507	7.985657116	5853	Glra3	5.035952746	4.332393305	5.654362639	8153	Lsm10	6.283533054	5.862951166	5.95791447810453	Pik3r3	8.420600218	8.28772473	8.670152096	12753	Slc2a12	6.473811299	6.920850947	6.594765855	15053	Uqcrb	10.1193061	10.00024065	9.985607085
1254	Aldh3a1	3.525321764	3.567782819	3.304561198	3554	Cpq	6.330841646	6.685756122	6.519060479	5854	Glrb	9.342864111	9.339267054	9.46582526	8154	Lsm11	7.73026237	7.777494675	7.28340394510454	Pik3r4	7.539608198	7.503451105	7.46377631	12754	Slc2a13	7.814801779	7.529093098	7.980306775	15054	Uqcrc1	9.765267885	9.97294327	9.827076719
1255	Aldh3a2	6.710193638	7.062232791	6.405440242	3555	Cps1	2.918536653	1.210081496	4.84105646	5855	Glrx	7.480351634	7.175720914	7.363763069	8155	Lsm12	6.508639598	6.604406388	6.87520515610455	Pik3r5	1.659291665	2.686533216	3.446999431	12755	Slc2a2	1.058754629	1.468619477	0.985123872	15055	Uqcrc2	11.02452906	10.85651514	10.98279571
1256	Aldh3b1	3.776592513	4.105550118	2.976608398	3556	Cpsf1	7.635653667	7.916610362	7.707885013	5856	Glrx2	9.224411952	8.980261578	8.940002214	8156	Lsm14a	7.926060313	7.935935719	8.20912567910456	Pik3r6	3.400069579	3.153686423	3.30197795	12756	Slc2a3	8.39237649	8.061153907	8.260882147	15056	Uqcrfs1	9.406892232	9.257373205	9.152300811
1257	Aldh3b2	3.063292754	3.040210437	2.790456425	3557	Cpsf2	9.097123995	9.027775785	9.101580326	5857	Glrx3	0.016601276	1.921605221	0.989760261	8157	Lsm14b	8.194542508	8.389632008	8.39375321810457	Pikfyve	8.102165408	7.87609698	8.145123564	12757	Slc2a4	3.220635191	1.431360237	2.570268097	15057	Uqcrh	11.61636394	11.56435154	11.69465879
1258	Aldh4a1	6.077815724	5.948490215	6.046646045	3558	Cpsf3	7.986641598	7.882744317	7.898935896	5858	Glrx5	8.996120196	8.909438596	8.883868243	8158	Lsm2	6.382338337	6.140178637	6.28398764510458	Pilra	4.661535201	4.22158323	4.50382168	12758	Slc2a4rg-ps	2.66830597	3.446567145	2.563636871	15058	Uqcrq	11.20462016	11.08275349	11.03999372
1259	Aldh5a1	7.835595334	7.87132804	7.97447531	3559	Cpsf3l	6.253407015	6.566576463	6.371917665	5859	Gls	10.16135753	10.22171516	10.16981734	8159	Lsm3	8.669343106	8.751081829	8.72147634410459	Pim1	4.831217676	4.892120503	4.788185644	12759	Slc2a5	4.250320285	4.42937929	4.840488458	15059	Urah	3.67393041	4.665759832	4.228696945
1260	Aldh6a1	7.429321926	7.415014041	7.511487279	3560	Cpsf4	5.287776331	5.267680039	5.189602378	5860	Gls2	6.587749141	6.275644208	6.946710945	8160	Lsm4	8.09223356	8.382339699	8.11945889910460	Pim2	6.505701415	6.468559264	6.565364277	12760	Slc2a6	6.164483907	6.290129894	5.734401909	15060	Urb1	4.319119781	3.404657813	4.738741678
1261	Aldh7a1	7.219439731	7.13246993	6.956928307	3561	Cpsf6	8.342604095	8.333714356	8.333135204	5861	Glt1d1	5.882059149	5.358525478	5.735834265	8161	Lsm5	4.930422232	4.482424322	4.56542657310461	Pim3	9.335022441	9.0372169	8.957177382	12761	Slc2a8	5.665681366	5.590318441	5.935008881	15061	Urb2	3.994459185	4.295715325	4.625131875
1262	Aldh9a1	7.429068454	7.540024614	7.406265984	3562	Cpsf7	7.376817908	7.537141854	7.893222024	5862	Glt28d2	1.128172844	2.633674813	3.151233806	8162	Lsm6	9.15444332	8.969281249	9.10154909510462	Pin1	8.446625015	8.57389233	8.269898528	12762	Slc2a9	3.977890357	3.419827841	3.148050771	15062	Urgcp	7.147677571	7.322103232	7.580370576
1263	Aldoa	12.75850018	12.75594899	12.42239062	3563	Cpt1a	6.54123706	7.073343134	6.422327835	5863	Glt8d1	6.492014288	7.120768375	6.887284996	8163	Lsm7	8.563825396	8.467558487	8.36795903210463	Pin4	6.723633697	6.721900033	6.549620246	12763	Slc30a1	7.787917919	7.932014718	7.934368569	15063	Uri1	8.848527197	8.968163389	8.907857352
1264	Aldoart1	5.470120242	5.573742271	5.472512226	3564	Cpt1b	2.068085297	3.602196409	1.982649297	5864	Glt8d2	5.605027455	5.505706625	6.025504379	8164	Lsm8	8.005686849	8.081082614	8.06212310810464	Pink1	10.44247344	10.43125806	10.40414909	12764	Slc30a10	6.719792486	6.175601734	6.389695299	15064	Urm1	8.294247673	8.173430266	8.144888691
1265	Aldoart2	2.07369775	2.44739864	2.304492097	3565	Cpt1c	7.400245393	7.196845293	6.837448834	5865	Gltp	7.759886278	8.103529163	7.471461825	8165	Lsmem1	1.062794356	0.867205996	2.57273952310465	Pinx1	7.539145787	7.594949889	7.354935633	12765	Slc30a2	5.283994215	5.55699199	4.563379661	15065	Urod	7.602284203	7.166614536	7.397961661
1266	Aldob	5.024916434	3.240541607	3.295087255	3566	Cpt2	4.671299307	5.741356473	4.43683881	5866	Gltscr1	5.119620321	4.528479842	5.537307782	8166	Lsp1	4.478230857	5.245065586	4.93490971410466	Pip4k2a	9.199604409	9.29954534	9.274622814	12766	Slc30a3	8.576305804	8.552122664	8.680536639	15066	Uros	5.542093268	5.534013377	5.40522824
1267	Aldoc	11.90241123	11.78816227	11.95617784	3567	Cptp	5.254349227	5.389135909	5.762229946	5867	Gltscr1l	8.119118944	8.066868337	8.088562406	8167	Lsr	5.986111078	5.55707171	5.78703183910467	Pip4k2b	8.874286747	8.689790635	8.777741472	12767	Slc30a4	7.881578757	7.946753432	8.297843038	15067	Usb1	5.129310882	5.678338037	5.681411168
1268	Alg1	7.429806601	7.729054463	7.549901491	3568	Cpxm1	2.411234052	3.965054208	3.684408553	5868	Gltscr2	7.497304006	7.148202134	7.609780234	8168	Lss	6.210353183	6.062306823	6.51983300510468	Pip4k2c	8.912809532	8.809995062	8.86580233	12768	Slc30a5	6.364576245	6.26459106	6.372657747	15068	Use1	9.621327707	9.33723973	9.387221548
1269	Alg10b	3.39533912	2.113600528	3.984167	3569	Cpxm2	3.085742675	3.948123129	3.889534067	5869	Glud1	10.24061445	10.16600386	10.47585904	8169	Lst1	5.287890323	5.196606745	5.47301541210469	Pip5k1a	10.15595685	10.06779025	10.10798442	12769	Slc30a6	6.077847196	6.221134175	5.934406416	15069	Usf1	4.935857013	4.585694874	4.98081195
1270	Alg11	8.8589086	8.927802396	8.931253989	3570	Cpz	0.016589069	0.915579389	0.989763241	5870	Glul	13.48642408	13.45392217	13.52131126	8170	Lta4h	6.885307331	6.578813129	6.74850236210470	Pip5k1b	6.82203957	6.849480638	6.637778318	12770	Slc30a7	6.555369208	6.661700538	6.188692734	15070	Usf2	10.43240066	10.34084937	10.25610984
1271	Alg12	4.592964315	3.923179802	3.980021545	3571	Cr1l	6.452308881	5.941291525	6.129655036	5871	Glycam1	1.073752234	3.067939065	1.984193065	8171	Ltb	0.019285051	1.917363198	0.98911608610471	Pip5k1c	9.489844202	9.77121985	9.654278833	12771	Slc30a9	9.263008125	9.331872673	9.391551231	15071	Ush1g	1.070272067	1.447272808	2.3077617
1272	Alg13	4.528669088	3.633780578	3.886789799	3572	Cr2	2.082695413	3.335936104	1.978842097	5872	Glyctk	4.080057673	4.306265346	4.373754581	8172	Ltb4r1	1.040717696	0.915517774	0.98975593810472	Pip5kl1	2.893313705	2.823481786	4.154351206	12772	Slc31a1	7.187990461	7.552002909	6.086167246	15072	Ushbp1	4.163554181	3.39911886	4.22928347
1273	Alg14	5.293010687	5.870168321	5.226919311	3573	Crabp1	3.407208834	3.749346222	3.981680361	5873	Glyr1	9.191727737	9.245273383	9.242100841	8173	Ltbp1	5.25421482	5.032892824	5.93563305210473	Pipox	2.665829447	1.794645076	2.787845324	12773	Slc31a2	6.005338061	5.418860147	5.107516315	15073	Usmg5	10.36929957	10.26200241	10.20517679
1274	Alg2	7.842781231	8.086763696	8.232940175	3574	Crabp2	5.239253443	4.594361215	4.6810238	5874	Gm10012	10.41485663	10.26794014	10.38511354	8174	Ltbp3	7.687165177	7.572608254	7.81954466310474	Pir	4.338496888	5.311300798	5.151120342	12774	Slc32a1	8.397591396	8.135723992	8.557651167	15074	Uso1	8.631375392	8.683514911	8.666497383
1275	Alg3	4.835618259	5.277246271	4.787081667	3575	Cracr2b	3.656187467	3.952903487	2.561286028	5875	Gm10033	6.006200762	5.477699528	5.838691485	8175	Ltbp4	6.121603283	6.071839717	6.02490786110475	Pisd	7.82110361	7.412406031	7.371870132	12775	Slc33a1	6.55815702	6.780843794	6.887169487	15075	Usp1	8.935261991	8.962404022	8.806484176
1276	Alg5	6.910453517	6.952665255	6.62392956	3576	Cradd	5.287061925	5.336656019	4.678554375	5876	Gm10052	3.250405553	3.763830055	2.97848108	8176	Ltbr	6.165130758	5.968025819	5.98014183310476	Pisd-ps1	7.658160031	7.611782683	7.526647153	12776	Slc35a1	6.573334537	7.02367559	6.694291978	15076	Usp10	7.765405117	7.429530709	7.372112862
1277	Alg6	6.18845655	5.75932066	6.109190826	3577	Cramp1l	6.653242353	7.028582649	7.08874612	5877	Gm10069	4.609345859	4.739386673	5.505825739	8177	Ltc4s	5.075419938	5.722625726	4.78741687810477	Pisd-ps2	4.827045629	3.907326725	4.565926667	12777	Slc35a2	6.421528716	6.538439416	6.40593841	15077	Usp11	8.762900304	8.352066434	8.522979907
1278	Alg8	5.469987098	5.853073035	5.189081738	3578	Crat	6.367087446	6.725158204	6.43933191	5878	Gm10094	7.745471949	7.81663918	7.52666925	8178	Ltk	5.285496749	4.965822825	5.30307025610478	Pithd1	8.630814026	8.542074775	8.620340111	12778	Slc35a3	4.83832618	4.965827715	5.266411144	15078	Usp12	7.588446154	7.727939795	7.55734593
1279	Alg9	6.099275881	5.861460849	5.957729644	3579	Crb2	7.365056154	7.34708335	7.077724362	5879	Gm10125	1.062775418	1.885105633	0.984084647	8179	Ltn1	7.932922502	8.017364211	8.15001024310479	Pitpna	10.15471785	10.16762919	10.16857452	12779	Slc35a4	6.973951314	6.826795244	6.862742015	15079	Usp13	7.237461709	6.692784577	7.002625872
1280	Alk	3.540737152	4.452814903	4.230866788	3580	Crb3	2.401560242	3.977009016	2.7893638	5880	Gm10190	2.658099625	3.191572555	2.983179982	8180	Ltv1	6.721854435	6.539741793	6.20811949810480	Pitpnb	7.703359147	7.893642194	7.951668749	12780	Slc35a5	7.739387268	7.920634409	7.945885043	15080	Usp14	9.944676533	9.901236677	10.02988033
1281	Alkbh1	4.605930057	4.13751426	4.887646987	3581	Crbn	9.572276477	9.382355256	9.572475323	5881	Gm10220	2.863276518	1.428137847	2.306040933	8181	Luc7l	9.512681014	9.156845767	9.30641162610481	Pitpnc1	9.78468633	9.87797696	10.04207019	12781	Slc35b1	8.928860951	8.985934575	8.714618351	15081	Usp15	9.15475651	9.031919111	9.18700507
1282	Alkbh2	3.995183733	4.294366151	4.982520806	3582	Crcp	6.556648607	6.71032649	6.405783528	5882	Gm10334	0.019303244	0.910078962	2.312433507	8182	Luc7l2	8.360958745	8.158877853	8.40638274810482	Pitpnm1	8.527737795	8.668280195	8.620285054	12782	Slc35b2	6.572285161	6.465350732	6.319217943	15082	Usp16	8.981339864	8.85693704	8.945767074
1283	Alkbh3	5.46470337	5.09065454	4.563261895	3583	Creb1	9.733043317	9.336944373	9.47980566	5883	Gm10336	5.36380672	5.230218785	5.189293311	8183	Luc7l3	11.16935087	10.98288795	11.0911079510483	Pitpnm2	9.361219764	9.502142887	9.473924315	12783	Slc35b3	4.986226943	4.687849119	5.44139561	15083	Usp18	3.639595078	2.162889303	2.56729106
1284	Alkbh4	5.604610209	5.843050156	5.471236873	3584	Creb3	8.526940734	8.349630929	8.18928576	5884	Gm10364	1.05674707	0.880602507	1.571459248	8184	Lum	3.093670722	4.45022401	3.6805392510484	Pitpnm3	6.810242342	7.161911185	7.496536438	12784	Slc35b4	6.988564197	7.196348893	7.218554809	15084	Usp19	8.290533303	8.636935801	8.515343185
1285	Alkbh5	8.477548901	8.562190473	8.605710257	3585	Creb3l1	5.235718944	4.709126904	3.676952987	5885	Gm10389	1.062773158	1.461158475	1.986252123	8185	Lurap1	4.991410319	4.915074847	5.53569791210485	Pitrm1	7.239741591	7.265737421	7.4150783	12785	Slc35c1	5.728050139	5.923411261	6.390465723	15085	Usp2	9.163022487	9.496819405	9.350699434
1286	Alkbh6	6.724185155	6.670179934	6.534181234	3586	Creb3l2	4.775129815	4.704653208	4.839644456	5886	Gm10390	2.866752335	0.811703086	3.156048325	8186	Lurap1l	7.701385924	7.573391009	7.17912435810486	Pitx2	1.113585149	1.366942179	3.443583985	12786	Slc35c2	7.498102642	7.539337081	7.397753366	15086	Usp20	8.264644522	8.090528304	8.208936305
1287	Alkbh7	6.652399798	7.136603563	6.812756235	3587	Creb3l3	3.384718649	2.422635178	2.565723758	5887	Gm10406	2.404758192	2.649783669	3.443561743	8187	Luzp1	9.954157601	9.89809398	10.0647938310487	Piwil1	0.069771144	3.48409694	1.565998582	12787	Slc35d1	6.17995173	5.870619102	5.14865611	15087	Usp21	7.299170464	7.247563949	7.557968352
1288	Alkbh8	6.794637157	6.861625776	6.694045693	3588	Creb5	5.038251212	5.629815242	5.864720077	5888	Gm10408	2.653424803	1.829854297	2.303304706	8188	Luzp2	9.710100385	9.634916451	10.0326295710488	Pja1	9.766932416	9.676990067	9.60748379	12788	Slc35d2	2.064560238	2.464079892	1.567067273	15088	Usp22	10.34497439	10.41261874	10.24909218
1289	Alms1	5.20767784	4.682185096	5.407502746	3589	Crebbp	9.703866513	9.456405159	9.455742679	5889	Gm10409	3.070457756	1.802189156	3.305356065	8189	Lxn	7.455242408	7.320603887	7.35494804910489	Pja2	11.96361184	11.88246079	11.95868898	12789	Slc35d3	3.906867066	4.47031372	5.53737574	15089	Usp24	7.794674723	7.815565271	7.793796695
1290	Alms1-ps2	2.072587796	2.678966555	3.155874686	3590	Crebl2	6.452863029	6.043601398	6.762736693	5890	Gm10421	1.67049544	2.670175022	3.154968744	8190	Ly6a	7.069629983	6.922299028	7.09888865810490	Pkd1	7.45514058	7.392491557	7.602373346	12790	Slc35e1	7.289204845	7.339859152	7.109138494	15090	Usp25	7.673019665	7.696053831	7.659445485
1291	Alox12	3.050063605	1.440725708	1.567779017	3591	Crebrf	11.30161048	11.27230394	11.43222817	5891	Gm10432	8.102066929	7.95182028	8.056956712	8191	Ly6c1	7.571625149	6.981187867	7.15940561910491	Pkd1l3	5.162506677	4.928538895	4.437595041	12791	Slc35e2	6.402800674	6.208145699	6.595097486	15091	Usp27x	6.59475709	5.929122579	5.404928309
1292	Alox12b	4.82143298	4.374536074	3.678691497	3592	Crebzf	5.98574946	4.756672195	6.22902059	5892	Gm10433	0.04019553	0.871762794	2.573060604	8192	Ly6c2	3.885220507	3.841009088	3.14642627210492	Pkd2	6.012728567	6.084714163	6.504548094	12792	Slc35e3	7.308667663	7.425636436	7.431572272	15092	Usp28	5.463939743	5.092290114	4.786915588
1293	Alox5	3.215568232	2.205972052	0.982609239	3593	Creg1	8.361368533	8.489727492	8.44357703	5893	Gm10471	2.638747616	0.846369315	1.984507	8193	Ly6d	0.032535892	1.476243392	2.31042601810493	Pkd2l1	2.386334171	1.422333412	1.56566069	12793	Slc35e4	6.503926175	6.331683972	6.439956775	15093	Usp29	9.682704188	9.606740074	10.14622526
1294	Alox5ap	5.367822209	5.892630197	5.371863769	3594	Creg2	9.180487479	8.898492933	8.94001088	5894	Gm10509	0.060214121	2.892020533	2.306884846	8194	Ly6e	9.578169443	9.650712095	9.47984359410494	Pkd2l2	4.730319008	5.005968989	5.109468371	12794	Slc35f1	9.569658267	9.367874549	9.263096581	15094	Usp3	6.573947604	6.781169363	6.680304734
1295	Alox8	3.783276599	4.093096459	4.506251798	3595	Creld1	6.914339598	7.284566822	7.056870375	5895	Gm10516	5.50034005	5.551938159	5.024171236	8195	Ly6f	2.065821725	2.190995656	1.98323670110495	Pkdcc	4.599161792	4.075699924	4.227419171	12795	Slc35f2	5.119849992	4.884524893	5.441789145	15095	Usp30	5.633845426	5.479849272	5.708469145
1296	Aloxe3	5.972368591	5.130825221	4.837749403	3596	Creld2	5.599097366	5.458249776	4.93282735	5896	Gm10536	0.011133568	0.927263826	0.991149321	8196	Ly6g6d	2.386332871	1.851496634	2.5695342510496	Pkdrej	2.074738822	2.173127989	3.308008584	12796	Slc35f3	8.398013036	8.388433887	8.479895965	15096	Usp31	8.16276169	8.021404914	8.179662852
1297	Alpk1	3.235639068	2.421082412	1.559907717	3597	Crem	6.708233766	6.341728676	6.666796488	5897	Gm10548	3.653341593	3.165140331	3.303309101	8197	Ly6g6e	2.877216138	2.151636284	2.30177395610497	Pkia	10.55731893	10.462429	10.54888651	12797	Slc35f4	5.860586264	5.641987207	5.93473324	15097	Usp32	8.572900311	8.591933002	9.016370168
1298	Alpl	4.544912886	5.615862795	4.563836182	3598	Crh	5.664675737	6.117097472	5.735310985	5898	Gm10560	3.632773682	0.822762403	2.305756255	8198	Ly6g6f	3.051806483	2.692459253	2.98685225810498	Pkib	6.708427476	7.025783441	6.666749084	12798	Slc35f5	5.508299038	5.866790912	4.883899315	15098	Usp33	9.017028632	8.922474767	8.937075724

1299	Als2	7.091158907	7.106006553	6.760817918	3599	Crhbp	7.216779734	6.861014952	7.002750393	5899	Gm10584	1.64163477	1.464853082	3.159350906	8199	Ly6h	9.633861103	9.202600368	8.67320694610499	Pkig	8.515474804	8.889424052	8.744974329	12799	Slc35f6	5.637142935	5.614758536	5.680088616	15099	Usp34	10.94774805	10.79560217	10.80573369
1300	Als2cl	3.669660147	4.170939497	4.151256545	3600	Crhr1	4.075446895	3.741460397	4.374817559	5900	Gm10635	2.41576302	3.166678954	4.378058008	8200	Ly6k	1.040699442	1.502475452	0.98976060810500	Pklr	1.094466388	1.834621927	3.155156294	12800	Slc35g1	3.547568952	3.926321338	4.683797392	15100	Usp35	6.377689484	5.88385142	5.679963828
1301	Alx3	1.068326452	2.703268599	1.985205719	3601	Crhr2	2.387270587	3.211744614	1.982075587	5901	Gm10636	1.622208275	0.921244318	0.990434977	8201	Ly75	1.638273663	0.880509157	2.31005729410501	Pkm	11.79136267	11.77151455	11.65636972	12801	Slc35g2	5.61449971	6.31133572	6.911865043	15101	Usp36	6.899898861	6.838384614	7.099527047
1302	Alx4	3.067393545	2.149424511	3.444093775	3602	Crim1	9.640482114	9.284346721	9.359328737	5902	Gm10638	3.395281876	3.867407923	3.303216443	8202	Ly86	5.296498153	5.669279047	5.59425254110502	Pkmyt1	7.963949281	8.146368048	8.169860657	12802	Slc36a1	7.881277753	7.662479183	8.324830309	15102	Usp37	6.807094129	6.448546002	6.652396066
1303	Alyref	7.375238007	7.705829562	7.389385321	3603	Crip1	5.206774721	5.200969868	4.786336961	5903	Gm10653	1.674008853	1.825277239	1.979333825	8203	Ly96	2.411317622	3.446403093	3.79171838510503	Pkn1	5.327855039	5.732567903	5.59579557	12803	Slc36a4	5.932900073	5.388735549	5.226811157	15103	Usp38	6.105737233	6.677054744	6.535050646
1304	Alyref2	5.252662284	5.75446957	5.188650667	3604	Crip2	9.30402916	9.354736701	9.164909335	5904	Gm10658	1.639969734	0.876068541	0.985111898	8204	Lyar	7.570884573	7.431457565	7.21818118910504	Pkn2	8.493934221	8.237318527	8.427069458	12804	Slc37a1	2.892752868	3.955247815	3.983889842	15104	Usp39	7.230331705	7.272987986	7.067141969
1305	Amacr	4.480786935	4.835692959	4.980329213	3605	Crip3	0.035169743	0.880514917	1.571450592	5905	Gm10677	4.149297699	3.305157803	2.979334801	8205	Lyg2	0.016605645	0.915545244	1.57499357110505	Pkn3	5.329866144	5.802171916	5.624563016	12805	Slc37a2	3.978110634	4.32619356	3.982270033	15105	Usp4	8.02250791	7.957227045	8.135068232
1306	Ambp	5.082346098	2.884441705	5.654057726	3606	Cript	8.591963036	8.649422593	8.609440619	5906	Gm10684	2.386373258	2.455442203	2.985985928	8206	Lyl1	6.267122751	6.447559873	6.15037476810506	Pknox1	6.490555749	6.899257726	6.207901879	12806	Slc37a3	7.905744497	7.727296159	7.542056017	15106	Usp40	5.813849907	6.31518296	6.149658747
1307	Ambra1	6.252540924	6.54022999	6.65293231	3607	Crisp2	1.730265598	5.800687405	3.787434141	5907	Gm10714	2.381072325	1.437602488	1.983868165	8207	Lyn	6.534612528	5.986037016	6.00220754710507	Pknox2	7.528788699	7.257776455	7.389871917	12807	Slc37a4	6.102142801	6.067300709	6.150237178	15107	Usp42	7.166945953	7.133988465	7.311419032
1308	Amd1	9.547889991	9.367095411	9.354996965	3608	Crispld1	5.666593506	5.9080128	5.934790487	5908	Gm10731	1.052492033	2.22939841	0.986737152	8208	Lynx1	9.71715022	9.816915678	9.77930035110508	Pkp2	6.22593946	6.129372292	5.064364716	12808	Slc38a1	10.13473099	10.2169425	10.47587576	15108	Usp43	2.374052973	1.457582159	2.308702139
1309	Amd2	10.13582354	10.0749293	10.07800376	3609	Crispld2	4.786178676	5.55181781	5.473304119	5909	Gm10754	2.381098316	1.437527711	0.980764406	8209	Lypd1	8.810584317	8.267040684	8.11409584310509	Pkp4	11.11868855	11.02649397	10.91808469	12809	Slc38a10	8.319148865	8.337470626	8.51539786	15109	Usp44	1.040698405	1.92160927	0.989760873
1310	Amdhd1	1.652381642	2.198422985	1.984200383	3610	Crk	8.455171454	8.25500257	8.475962139	5910	Gm10767	4.398200216	4.082077133	4.682639645	8210	Lypd4	1.058792514	2.715088028	2.57336777810510	Pla1a	1.094463087	2.441892061	1.980404814	12810	Slc38a2	9.398319099	9.312595956	9.738277846	15110	Usp45	7.491534552	7.726225553	7.825562556
1311	Amdhd2	5.657723747	4.827768916	5.228306377	3611	Crkl	8.04060832	7.875142736	8.284018107	5911	Gm10778	7.134993135	6.811648275	7.34688943	8211	Lypd6	6.648410372	6.482313643	6.762087310511	Pla2g12a	8.30310738	7.94599709	7.951489268	12811	Slc38a3	6.822215587	6.779251455	6.874869178	15111	Usp46	8.424537391	8.377241151	8.499766542
1312	Amer1	5.721666717	5.323703125	5.594931988	3612	Crlf1	5.07642359	5.132723675	3.97555419	5912	Gm10785	3.870590991	2.398323773	3.791267758	8212	Lypd6b	6.168011492	6.024346512	6.38998083110512	Pla2g15	6.453932295	6.584935991	6.129250678	12812	Slc38a4	3.985351341	3.401650363	4.229525633	15112	Usp47	8.900714042	8.865596875	8.985672986
1313	Amer2	9.034426034	8.935978026	8.837913698	3613	Crlf2	6.420083963	6.58305021	6.488029964	5913	Gm10789	0.008381654	1.518469423	0.991898458	8213	Lypd8	0.013875062	1.507515853	1.99105106710513	Pla2g16	9.338005236	9.514361086	9.274559031	12813	Slc38a5	4.067171578	3.294548378	3.789437216	15113	Usp48	8.749162462	8.648321191	8.731583882
1314	Amer3	6.011218217	6.007789516	5.837436637	3614	Crlf3	6.016052224	6.540563511	6.128923677	5914	Gm10790	1.097347731	2.43825553	2.567529376	8214	Lypla1	6.556672059	6.994397072	6.63829944910514	Pla2g2d	2.062175551	1.440725577	0.981216259	12814	Slc38a6	4.075882297	3.837521833	3.787067702	15114	Usp49	4.403710774	4.147171577	4.936131625
1315	Amfr	8.728096335	8.871926571	8.704462458	3615	Crls1	7.873302989	7.684467402	7.430966225	5915	Gm10814	1.064657738	2.211490648	1.569744748	8215	Lypla2	8.234782681	8.359555656	8.10927732310515	Pla2g3	2.86142428	1.434446987	1.567053898	12815	Slc38a7	5.931643741	5.597464898	4.731889765	15115	Usp5	8.5475034	8.296705596	8.471930729
1316	Amhr2	1.085441913	2.181778662	1.982040974	3616	Crmp1	7.963132201	8.152529738	7.916583914	5916	Gm10865	3.39002431	3.876775723	3.151890712	8216	Lyplal1	4.834797728	5.17136972	5.22960828210516	Pla2g4a	4.59898065	4.759589965	4.22746875	12816	Slc38a9	7.124668577	6.856586501	7.09894639	15116	Usp50	1.078851516	3.48716112	2.306333397
1317	Amica1	3.236286355	2.651169319	3.153017867	3617	Crnde	2.408164375	3.020913385	3.792215934	5917	Gm10941	2.636632849	0.854398292	2.308057595	8217	Lyrm1	6.709878622	6.590608961	6.9805733610517	Pla2g4c	0.027253816	2.499996574	0.987309789	12817	Slc39a1	8.209661638	8.294165852	7.962750138	15117	Usp51	4.531928455	4.162226922	4.30289181
1318	Amigo1	7.731876349	7.792154836	7.980239344	3618	Crnkl1	7.695103155	7.612013448	7.83195892	5918	Gm11110	1.644914073	0.862950105	1.569735295	8218	Lyrm2	7.5156576	7.788341239	7.47165767910518	Pla2g4e	3.397570992	2.82239332	4.306805427	12818	Slc39a10	10.11927581	9.919837554	10.26779301	15118	Usp53	7.032367719	6.643154735	6.787407116
1319	Amigo2	7.069047128	7.389552197	6.812399849	3619	Crocc	5.540931824	5.97640341	5.264102135	5919	Gm11149	3.24805061	3.434194403	3.567032083	8219	Lyrm4	9.08844895	8.933450941	8.8656639210519	Pla2g5	4.087799519	5.145375301	4.681473653	12819	Slc39a11	7.122223862	7.210555082	6.454908422	15119	Usp54	7.034295902	6.891729035	6.608534559
1320	Amigo3	2.068072994	1.854136087	1.982652491	3620	Crot	6.83521247	6.803511028	6.693973838	5920	Gm11346	0.005608334	0.940318714	0.992698372	8220	Lyrm5	6.888232164	7.311106048	7.13013482710520	Pla2g6	6.145935633	6.53364602	6.20915375	12820	Slc39a12	7.320525681	7.579530877	7.430999469	15120	Usp6nl	4.792021779	5.512803696	5.935879637
1321	Ammecr1	4.080727085	4.90894064	4.56705383	3621	Crp	3.297175161	3.781172235	5.913430408	5921	Gm11423	2.402364614	2.422727338	2.565734387	8221	Lyrm7	6.0140399	6.315113014	6.08806897610521	Pla2g7	9.818751379	9.844751423	9.661138381	12821	Slc39a13	6.252362521	6.726176294	6.372172175	15121	Usp7	10.23410298	10.1539473	10.34086504
1322	Ammecr1l	7.716517493	7.413008917	7.564890957	3622	Crtac1	8.869555641	8.536712271	7.837483594	5922	Gm11517	3.648430061	3.781675081	2.981285794	8222	Lyrm9	7.505131937	7.212234428	7.31099294810522	Pla2r1	0.042848341	1.885087815	1.986249615	12822	Slc39a14	5.126982518	4.568633681	5.337862569	15122	Usp8	9.191618071	9.078251938	9.172285686
1323	Amn	6.34469604	5.7104247	6.189656335	3623	Crtap	4.167549231	4.302926933	4.682756111	5923	Gm11549	5.443939632	5.991105562	6.682679886	8223	Lysmd1	6.602594285	6.452521364	6.31948564810523	Plaa	8.108211178	7.886150355	8.306686322	12823	Slc39a2	2.648281081	2.449548002	3.155886347	15123	Usp9x	9.494495649	9.518584384	9.856580641
1324	Amn1	6.079388254	6.145282015	5.734060144	3624	Crtc1	8.784132336	8.807248129	8.711328692	5924	Gm11627	4.24716927	3.625919958	3.885897205	8224	Lysmd2	7.998572589	7.610280227	7.82546530210524	Plac9a	4.983842413	5.208881731	4.563995651	12824	Slc39a3	7.135047433	7.373987692	7.024358993	15124	Uspl1	7.564410064	7.358839822	7.714902951
1325	Amot	7.209514577	7.299095593	7.329000999	3625	Crtc2	4.425490148	5.645377763	6.110742845	5925	Gm11681	4.719579968	4.597770716	3.559949995	8225	Lysmd3	5.606300826	5.56029843	5.91120297610525	Plac9b	4.988401648	5.371063593	4.786221219	12825	Slc39a4	4.471027042	4.806507511	2.552068746	15125	Ust	7.462238872	7.550056837	6.824781722
1326	Amotl1	8.013373948	8.166127184	8.731695478	3626	Crtc3	6.505761221	6.822827155	6.422975516	5926	Gm11696	2.396655338	2.665122791	3.307008854	8226	Lysmd4	6.806220818	6.373129108	6.81343623410526	Plag1	3.651649781	3.312102956	2.298274128	12826	Slc39a5	5.327068259	5.630142531	5.595982559	15126	Utp11l	7.723870397	7.645348899	7.542098132
1327	Amotl2	6.188341973	6.267175737	6.390098998	3627	Cry1	6.250438784	6.298345366	6.283867442	5927	Gm11715	7.008708123	6.85139051	6.70789006	8227	Lyst	7.413367626	7.708025166	7.98031591610527	Plagl1	6.958071484	6.554740341	6.188816702	12827	Slc39a6	8.194480935	8.322314582	8.169740939	15127	Utp14a	6.664503251	6.187696594	6.227037845
1328	Ampd1	0.008381654	1.518469423	0.991898458	3628	Cry2	8.198943215	8.056833823	8.213754842	5928	Gm11744	4.164284531	4.439194577	4.505908148	8228	Lyve1	4.591593961	3.269319022	3.78681007110528	Plagl2	5.075803622	4.929734696	5.110551482	12828	Slc39a7	8.058532818	8.431951024	8.154848857	15128	Utp14b	7.230032465	6.910416554	7.056546457
1329	Ampd2	7.114145767	7.178289871	7.41510193	3629	Cryab	9.75840072	10.03684413	9.636332061	5929	Gm11747	1.6552617	1.43443252	1.567052229	8229	Lyz2	3.965724922	3.019133433	2.29956312210529	Plat	6.211221417	6.624889931	5.978960042	12829	Slc39a8	3.770484276	3.550462078	3.149469831	15129	Utp15	6.422618254	6.709447435	6.923125204
1330	Ampd3	5.599964783	4.912067598	5.337610359	3630	Cryba4	2.071380767	3.210201798	1.565018019	5930	Gm11762	2.052729799	1.464792965	2.573049664	8230	Lyzl4	1.643289595	2.483060114	1.57015523410530	Platr17	3.518550145	1.38128415	0.972732223	12830	Slc39a9	7.963355226	8.356573333	7.812652968	15130	Utp18	6.780336754	6.805272977	6.594427498
1331	Amph	9.955209706	9.729563604	9.657668724	3631	Crybb1	3.893731544	4.492961077	4.682639997	5931	Gm11780	0.035073532	2.913759812	2.31007105	8231	Lzic	5.039148165	5.681080872	5.2652387710531	Platr22	1.632777315	1.484360077	1.988639748	12831	Slc3a1	3.890690006	3.7324182	4.567330856	15131	Utp20	6.383925347	6.361697697	6.503951987
1332	Amt	1.062669337	1.461351022	0.984112102	3632	Crybb3	3.059003978	2.445613535	1.564020764	5932	Gm11837	7.318691083	7.454789017	7.456010437	8232	Lztfl1	7.880797623	7.597648317	7.80659749510532	Plau	1.670429971	3.471602917	2.567733771	12832	Slc3a2	9.483527449	9.347173062	9.453687537	15132	Utp23	7.012012413	7.03652792	7.237622159
1333	Amy1	4.257559209	4.476587	5.230341699	3633	Crybg3	6.52198487	6.392707424	6.455919706	5933	Gm11944	5.750258671	5.890824824	5.624078507	8233	Lztr1	8.547384607	8.377247193	8.40210662110533	Plb1	2.859523321	0.842577911	1.567796147	12833	Slc40a1	5.400175411	4.999668122	5.50447398	15133	Utp3	9.335409215	9.223559374	9.337581615
1334	Amz1	5.425819532	4.534498109	4.98141329	3634	Cryga	2.070450645	2.18173127	1.565298695	5934	Gm11974	4.323448543	4.436108128	4.441139432	8234	Lzts1	3.094252885	3.411089712	4.44276994210534	Plbd2	7.300107294	7.699943854	7.431321873	12834	Slc41a1	7.759736495	7.611583742	7.659400249	15134	Utp6	7.67857973	7.4756276	7.227651856
1335	Amz2	7.580103454	7.495613812	7.594789486	3635	Crygn	2.654159235	2.666940207	1.562487072	5935	Gm11992	1.104518677	2.429281665	1.561290771	8235	Lzts2	7.249256256	7.026734493	7.00218692510535	Plcb1	10.75381058	10.7620248	10.78977504	12835	Slc41a2	2.399148853	2.660406653	3.444552014	15135	Utrn	8.420346406	8.325097904	8.503637604
1336	Anapc1	6.913884557	6.902918769	7.13005254	3636	Cryl1	5.753015039	6.113172087	6.068415809	5936	Gm12060	1.07023244	0.850518501	2.571586299	8236	Lzts3	7.221775881	7.452402197	7.77485751810536	Plcb2	2.650909768	2.171132466	2.79143192	12836	Slc41a3	3.903760583	4.590916668	4.564609184	15136	Uty	6.491315679	6.78310386	6.694386918
1337	Anapc10	5.987793607	6.007543303	6.373303188	3637	Crym	10.17343257	9.709043072	8.84382575	5937	Gm12070	6.722270711	6.58075049	6.594916855	8237	M6pr	8.058065266	8.331506867	8.23751281410537	Plcb3	5.356984378	4.743064898	4.437777629	12837	Slc43a2	7.860410777	7.703192706	7.701233456	15137	Uvrag	7.269105502	6.878808092	7.488454488
1338	Anapc11	9.472802711	9.430268897	9.477857585	3638	Cryz	6.077505894	5.883375328	5.147309805	5938	Gm12191	7.894833367	8.291582065	7.957222232	8238	Maats1	3.879701507	4.393378662	3.30104342810538	Plcb4	7.811045179	7.926880048	8.731826717	12838	Slc43a3	2.389241422	2.877621756	2.985395161	15138	Uvssa	6.846965432	6.758523752	6.708028168
1339	Anapc13	8.679234894	8.482682349	8.174573032	3639	Cryzl1	8.498447784	8.52091145	8.507461688	5939	Gm12216	0.067391729	0.82697819	2.306055573	8239	Mab21l1	4.84618543	4.3833284	5.91218703610539	Plcd1	4.98847284	5.608577135	5.109507515	12839	Slc44a1	7.95629473	8.221032661	7.812882934	15139	Uxs1	5.96069662	5.839586127	6.110312754
1340	Anapc15	5.286973287	5.234305569	5.373350876	3640	Cs	9.388821844	9.115284539	9.31312024	5940	Gm12338	10.89192821	10.64884474	10.82781501	8240	Mab21l2	3.794990519	2.296565655	5.19243824710540	Plcd3	4.934372868	4.746436957	4.623525701	12840	Slc44a2	6.12242161	6.305977553	6.024704936	15140	Uxt	5.162413377	5.013897357	5.267127053
1341	Anapc16	8.971950475	8.815000689	8.700986034	3641	Csad	7.429492159	8.023824999	7.397737391	5941	Gm12359	5.204799693	3.970108358	5.110131934	8241	Macf1	10.50587837	10.47057266	10.7793036710541	Plcd4	6.350306698	6.493346379	6.787685561	12841	Slc44a3	1.0502769	2.499978229	1.988647229	15141	Vac14	5.543532875	5.9924688	5.934634322
1342	Anapc2	8.167817437	8.085446053	8.194382024	3642	Csdc2	6.489392791	6.190179488	6.667046904	5942	Gm12504	5.807242657	5.952203713	6.025348065	8242	Macrod1	6.168907881	6.729897075	5.97920273610542	Plce1	6.312368269	6.672110412	6.245865921	12842	Slc44a5	6.324762467	5.944851942	5.437450855	15142	Vamp1	9.126231664	9.113862554	10.05555591
1343	Anapc4	8.596135066	8.592930803	8.397695773	3643	Csde1	11.59627466	11.57079755	11.58360669	5943	Gm12522	3.960676835	3.190393911	2.789540931	8243	Macrod2	8.040544812	8.160828153	8.28403391710543	Plcg1	3.395716272	3.306527904	2.979521105	12843	Slc45a1	6.286767909	6.028018869	6.02441762	15143	Vamp2	11.93384561	11.98639215	11.83094922
1344	Anapc5	8.903812941	9.043335027	8.942935478	3644	Cse1l	8.163057055	8.172650924	8.288369021	5944	Gm12530	1.647917483	3.824549111	1.98520848	8244	Mad1l1	6.187737341	5.760767207	5.86196658910544	Plcg2	3.658158048	2.097690524	3.682314178	12844	Slc45a3	2.390234477	3.476971589	2.568609922	15144	Vamp3	7.766401621	7.868005871	7.616559426
1345	Anapc7	6.231755027	6.136747831	6.283558222	3645	Csf1	6.190664234	6.5425044	6.208471391	5945	Gm12596	7.220353778	7.024369193	7.246759674	8245	Mad2l1	4.400916079	4.75956062	3.88462095410545	Plch1	4.253113838	4.806032014	4.301499275	12845	Slc45a4	6.794240682	6.960553418	6.838197184	15145	Vamp4	8.36504299	8.256557662	8.124617261
1346	Ang	3.988989693	4.435888823	3.29748927	3646	Csf1r	9.311527137	9.247424167	9.186991634	5946	Gm12657	4.541000622	4.749877966	4.98158161	8246	Mad2l1bp	5.505400897	5.677985719	5.3721582610546	Plch2	7.462505276	7.637635381	7.129582586	12846	Slc46a1	5.860680576	5.816302708	5.652015887	15146	Vamp5	4.82854918	4.291720395	4.62467847
1347	Angel1	3.878056579	3.946375456	3.68194305	3647	Csf2ra	6.341091174	5.83830373	5.812856645	5947	Gm12669	5.809764872	5.858979664	5.886811517	8247	Mad2l2	4.984478154	4.691574823	5.02525745810547	Plcl1	8.280049045	8.310078958	8.363815959	12847	Slc46a3	3.994200625	4.759527266	4.439961413	15147	Vamp8	6.616937548	6.556169769	6.455996895
1348	Angel2	7.573908335	7.963157803	7.837965822	3648	Csf2rb	2.864959699	3.057309003	2.79270942	5948	Gm12709	6.470242747	6.380142612	6.28391569	8248	Madd	8.64959759	8.583761783	8.54995353410548	Plcl2	7.731740752	7.732412539	8.10454103	12848	Slc47a1	4.772999874	4.296204523	4.840167572	15148	Vangl1	6.0547443	5.501788708	5.812877509

1349	Angpt1	7.408198208	7.198702701	6.837681481	3649	Csf3r	1.056773941	1.89488725	1.987394672	5949	Gm128	1.104441134	3.464062155	3.306552082	8249	Maea	8.51910125	8.463627595	8.70129735110549		Plcxd1	7.059163137	7.492249271	7.573000907	12849	Slc48a1	10.84874191	10.88842526	10.77276126	15149	Vangl2	4.773132924	4.545856202	4.504342678
1350	Angpt2	2.684724515	3.942873366	3.148144989	3650	Csgalnact1	7.135085982	7.017397615	7.228265115	5950	Gm12942	3.255956437	3.754129378	3.888707367	8250	Mael	2.412383784	4.898711395	2.29873775410550		Plcxd2	9.025827244	8.518330009	9.548282089	12850	Slc4a10	9.642615307	9.492291744	9.603894779	15150	Vapa	9.665730242	9.792033881	9.906325286
1351	Angptl1	2.656585349	2.861531246	2.790068337	3651	Csgalnact2	5.91702003	6.140154328	6.37275778	5951	Gm12992	7.596382467	7.758231221	7.673553301	8251	Maf	7.539117028	7.279142742	7.6808450110551		Plcxd3	7.799106947	7.345085742	7.527101681	12851	Slc4a11	1.102686714	1.38727469	0.973593267	15151	Vapb	9.730002085	9.900033893	10.01759431
1352	Angptl2	3.661745278	3.755152742	3.788840784	3652	Csk	6.083015802	6.297365303	6.389636294	5952	Gm13031	0.083821736	2.173121658	2.791626427	8252	Maf1	8.103177581	8.252216003	8.24216755610552		Plcz1	1.652443205	2.893989534	1.984186491	12852	Slc4a1ap	7.861161468	7.570867919	7.666407058	15152	Vars	6.307629158	6.365874892	6.08831401
1353	Angptl3	3.243596553	2.121780922	3.891240685	3653	Csl	0.03251111	2.494013103	1.987804412	5953	Gm13034	3.081641201	4.039603453	3.567032851	8253	Mafb	7.678086485	7.305356529	7.38950707310553		Pld1	4.827701879	4.223850149	3.97790517	12853	Slc4a2	6.212523639	7.096854527	6.108317616	15153	Vars2	5.932052227	5.650520362	5.681212359
1354	Angptl4	3.979362257	3.284908094	3.147571285	3654	Csmd1	6.540143847	6.49486575	6.851023824	5954	Gm13102	1.056722096	1.89497216	1.571464688	8254	Maff	2.882470423	3.022878028	2.56463836110554		Pld2	4.601684095	3.901975568	4.503696181	12854	Slc4a3	8.03407959	7.814284743	8.051630889	15154	Vash1	7.716289099	7.934505597	7.38057657
1355	Angptl6	6.10336503	6.214497448	6.550924584	3655	Csmd2	7.209479355	7.103782589	7.504130096	5955	Gm13139	4.890462892	5.200337665	5.710288997	8255	Mafg	8.615766355	8.527387935	8.70458690710555		Pld3	10.04152616	9.70502282	9.796994352	12855	Slc4a4	10.3651305	10.34101001	10.97297818	15155	Vash2	3.264938593	4.01235065	4.229504034
1356	Angptl7	3.372480544	0.823181197	1.98263804	3656	Csmd2os	2.382202032	0.834507082	2.793615315	5956	Gm13152	0.019302225	0.910080995	1.574430524	8256	Mafk	5.664977171	5.772341432	5.40544458610556		Pld4	4.602167565	4.596088429	4.56525159	12856	Slc4a5	4.733565419	6.356573269	3.973368676	15156	Vasn	5.632462946	5.150656179	5.301360963
1357	Ank	9.765431885	9.765381844	10.0079512	3657	Csmd3	7.603760225	7.208629263	7.687551835	5957	Gm13157	3.546099333	4.014673887	4.229772028	8257	Mag	10.43157243	10.7571496	10.21712110557		Pld5	5.087298036	5.622463175	6.322317434	12857	Slc4a7	7.766697506	7.850591401	7.541997668	15157	Vasp	5.839188962	6.655748876	5.786257533
1358	Ank1	6.712637067	6.891014631	7.73607018	3658	Csnk1a1	10.14249671	10.01013207	10.07274653	5958	Gm13238	3.648984714	2.840787732	3.151844966	8258	Maged1	10.08617446	10.1023341	10.0674446110558		Pld6	1.673894558	2.665239899	3.307020306	12858	Slc4a8	8.107891995	7.961715045	8.213858615	15158	Vat1	5.034451182	5.236833826	4.837196172
1359	Ank2	10.60900944	10.52067767	10.70281495	3659	Csnk1d	8.748929708	8.668022689	8.623866405	5959	Gm13293	4.485899419	5.196074533	5.535903252	8259	Maged2	7.619261342	7.53115519	7.58737891210559		Plec	8.26573381	8.445937109	8.656226313	12859	Slc50a1	6.183502348	5.840434099	5.911281162	15159	Vat1l	7.506481647	7.717395825	7.328510254
1360	Ank3	11.97656579	12.01081257	12.11601481	3660	Csnk1e	8.756394085	8.786602571	8.982991992	5960	Gm13305	6.151779406	6.806870914	7.03616164	8260	Magee1	9.127636235	8.958645328	9.1796998210560		Plek	6.061902928	6.526559826	6.638753246	12860	Slc52a2	4.252087164	4.988271209	4.681941632	15160	Vaultrc5	3.080816889	3.435710577	3.441106091
1361	Ankar	1.683244253	4.543807647	2.982415096	3661	Csnk1g1	7.547548372	7.496587126	7.708289047	5961	Gm13306	6.592427439	7.224075835	7.573349798	8261	Magee2	6.02498694	5.192030954	4.83600411810561		Plek2	2.393045285	2.870903366	2.303784242	12861	Slc52a3	2.650033722	1.408021799	1.980939272	15161	Vav1	3.52673132	3.315355417	2.298840576
1362	Ankdd1b	5.251683416	5.456186377	5.188894911	3662	Csnk1g2	9.077471063	9.156116793	9.018931869	5962	Gm13308	1.048021955	1.488660129	0.987885118	8262	Magef1	4.401584862	4.361464706	4.98257084110562		Plekha1	8.877603105	8.81014644	8.557315917	12862	Slc5a10	2.651745156	1.402429612	2.567942049	15162	Vav3	5.815832914	6.596230252	6.550181804
1363	Ankef1	2.412625615	3.674513603	2.563299386	3663	Csnk1g3	8.406426166	8.26407312	8.25143267	5963	Gm13315	2.866673142	2.879213048	0.977769201	8263	Mageh1	8.370075776	8.203807082	8.14478565310563		Plekha2	7.627806925	8.005938181	7.511110285	12863	Slc5a11	2.05415099	1.461204182	0.98409161	15163	Vax1	1.056737568	0.880623446	2.310066252
1364	Ankfn1	0.06740346	2.188692104	2.793155707	3664	Csnk2a1	10.37167703	10.15600517	10.45879084	5964	Gm13363	7.025456584	7.58902234	7.488011469	8264	Magel2	3.409988701	3.640450282	4.230122110564		Plekha3	8.209672824	8.021437157	8.154894095	12864	Slc5a12	2.390231233	2.175416125	2.791851039	15164	Vbp1	8.555807725	8.388322697	8.471881571
1365	Ankfy1	7.580725171	7.537170162	7.564847314	3665	Csnk2a2	7.943955735	7.979189996	7.868824954	5965	Gm13375	4.543824881	4.974383026	4.980944406	8265	Magi1	8.419831249	7.959324724	8.58389880810565		Plekha4	1.669154385	2.672125582	2.98463175	12865	Slc5a2	1.040705933	0.915543165	0.989758948	15165	Vcam1	7.745536288	7.702225891	7.609237576
1366	Ankhd1	9.766760073	9.543281189	9.572431699	3666	Csnk2b	8.304029389	8.243490054	8.223205208	5966	Gm13446	4.607090769	4.208512505	4.7347218	8266	Magi2	9.698644142	9.529557401	9.78422455310566		Plekha5	7.22315652	7.564276934	7.666442718	12866	Slc5a3	8.687505336	8.440569955	8.337328771	15166	Vcan	7.280394387	7.255032912	7.423127809
1367	Ankib1	7.627782771	7.584927676	7.881115553	3667	Csnka2ip	0.029928077	2.229368113	0.986731924	5967	Gm13483	3.254690383	0.638528627	4.23140978	8267	Magi3	8.446995089	8.348675473	8.45974984110567		Plekha6	6.976853801	7.213574494	7.667312974	12867	Slc5a5	6.186137005	5.788088822	6.190161917	15167	Vcl	6.231464889	6.060578831	6.246532703
1368	Ankle2	7.179087257	7.238602923	7.109019666	3668	Cspg4	5.749314772	5.62282601	5.534736996	5968	Gm13498	5.474699835	5.889660846	5.594680986	8268	Magoh	8.119005994	8.04261707	7.95719327210568		Plekha7	5.666914153	5.929300683	5.862936767	12868	Slc5a6	6.056405979	6.183207146	5.734195396	15168	Vcp	10.87812241	10.82020881	10.67954109
1369	Ankmy2	7.836017152	8.236977922	7.980024854	3669	Cspg5	10.14794468	10.02869032	10.07537143	5969	Gm13629	4.417406935	4.916281241	5.535833211	8269	Magohb	6.329195955	6.295190694	6.40628092510569		Plekha8	6.602089001	6.097964073	6.609850787	12869	Slc5a7	3.414862933	4.61512222	4.505968637	15169	Vcpip1	9.202113854	9.053869795	9.372390625
1370	Ankra2	9.392990343	9.175374764	9.083208821	3670	Cspp1	7.587586993	7.227070412	7.511436484	5970	Gm13807	0.024610413	2.72877403	0.987893738	8270	Magt1	8.665123493	8.748980521	8.46775519410570		Plekhb1	11.03406103	11.21412578	10.91290942	12870	Slc5a8	0.037659585	1.891634741	1.987014363	15170	Vcpkmt	4.549155264	4.783323443	5.067564217
1371	Ankrd10	7.409704851	7.282419651	6.910231269	3671	Csrnp1	5.205093105	5.240145897	4.93426521	5971	Gm13830	3.540518334	3.9391153	3.888417452	8271	Mak	3.78333653	3.738027707	4.62699460910571		Plekhb2	10.41600253	10.31934041	10.59196305	12871	Slc6a1	8.742712781	8.716568435	8.910752166	15171	Vdac1	10.95020447	10.98626503	11.0413604
1372	Ankrd11	11.51611173	11.19745754	11.1997431	3672	Csrnp2	6.147436181	5.961234773	6.169681957	5972	Gm13889	7.794240135	7.733064296	7.471513597	8272	Mak16	8.249358976	7.892073172	8.04612302410572		Plekhd1	4.17489656	4.35527755	4.681146862	12872	Slc6a11	8.819975411	8.65147147	9.602150472	15172	Vdac2	9.798288136	9.769866571	9.851898563
1373	Ankrd12	12.2514041	11.90204938	12.11761107	3673	Csrnp3	9.42968832	9.502687377	9.75975306	5973	Gm13986	2.375329851	0.858589918	2.308372353	8273	Mal	11.81654187	11.90667539	11.4622493410573		Plekhf1	4.937798873	4.465985671	4.563437556	12873	Slc6a12	2.064622688	1.434401068	2.98684925	15173	Vdac3	9.277252171	9.088249164	9.067450737
1374	Ankrd13a	7.316835036	7.086500834	7.189727147	3674	Csrp1	10.24065947	10.56683165	10.38511095	5974	Gm14005	2.380000273	2.468400093	0.981210031	8274	Mal2	10.13567758	9.891429598	10.1348428210574		Plekhf2	6.379934494	6.163501403	6.109595405	12874	Slc6a13	5.7147186	4.46024021	4.562740754	15174	Vegfa	7.848872257	8.144096353	7.455139947
1375	Ankrd13b	7.317203502	7.214767444	7.056751224	3675	Csrp2	6.400833598	6.717264923	6.169579139	5975	Gm14057	5.362657745	5.159950323	5.50484518	8275	Malat1	13.95283718	13.99274044	14.4048619110575		Plekhg1	5.333755061	5.562469996	5.405251174	12875	Slc6a15	7.634614749	7.639784044	7.255641253	15175	Vegfb	8.845136553	8.863622939	8.784142818
1376	Ankrd13c	6.229496986	6.283430974	6.535563611	3676	Csrp2bp	7.309229094	7.167080221	7.519521803	5976	Gm14164	8.304556668	8.22089687	8.17461204	8276	Malsu1	6.421029027	6.436876829	6.47181735310576		Plekhg2	3.233205922	2.658940485	2.789722905	12876	Slc6a17	8.25011013	8.476866481	8.324446272	15176	Vegfc	3.08990494	3.850951295	4.569462903
1377	Ankrd13d	6.228373202	6.124565773	5.861996195	3677	Cst12	0.016583365	0.915591139	0.989764633	5977	Gm14169	4.782624207	4.40541611	5.151013337	8277	Malt1	2.654227296	3.041566049	3.15462142810577		Plekhg3	6.00527879	6.156360291	5.43900917	12877	Slc6a20b	1.048027375	1.488649954	1.573369701	15177	Vezf1	8.702998095	8.888001674	8.940064596
1378	Ankrd16	4.668637505	5.34489401	4.837954157	3678	Cst13	0.011134731	1.930843894	0.991149012	5978	Gm14204	4.546799695	4.787785293	5.536885907	8278	Mamdc2	2.070388766	2.181855263	1.56531735410578		Plekhg4	2.646580622	2.453335478	1.565299891	12878	Slc6a6	9.202059229	9.245352261	9.387362362	15178	Vezt	8.347091882	8.216680044	8.572710648
1379	Ankrd17	10.5098894	10.45165337	10.40519342	3679	Cst3	13.64357336	13.74792647	13.57020614	5979	Gm14288	3.081678858	3.664852087	3.440914196	8279	Mamdc4	1.062785661	2.214218789	0.98408199610579		Plekhg5	8.433524791	8.560141771	8.527007303	12879	Slc6a7	7.653343324	6.869138707	6.850038132	15179	Vgf	9.034588117	9.03783326	8.883808351
1380	Ankrd2	0.076709959	1.416572597	2.305017173	3680	Cst6	3.985631605	4.312167358	4.841885746	5980	Gm14295	3.684528626	4.75229285	4.681098093	8280	Maml1	3.904548878	4.533084939	5.11102929910580		Plekhh1	7.375493098	7.686426748	7.363594497	12880	Slc6a8	7.687694701	7.816833829	7.74165795	15180	Vgll3	4.698801181	1.319659712	3.683099581
1381	Ankrd23	2.887814417	2.12288304	3.151218654	3681	Cst8	0.07414405	4.305287783	1.565367182	5981	Gm14296	6.60648539	6.692935424	7.002645125	8281	Maml2	6.924675229	6.744274788	6.53402438810581		Plekhh2	5.42062589	6.518082126	7.464906865	12881	Slc6a9	7.550151704	7.962473019	7.819078182	15181	Vgll4	6.489927639	6.832750914	6.666916466
1382	Ankrd24	6.987157461	7.068431195	6.799843756	3682	Cstad	4.245460635	4.23937105	4.067841559	5982	Gm14305	2.869147013	2.173124031	1.564000805	8282	Maml3	5.85974039	5.994901777	6.35695809110582		Plekhh3	4.984973863	4.637992434	5.025135369	12882	Slc7a1	7.53162968	7.59370019	7.71499591	15182	Vhl	8.234380126	7.977021247	7.874632876
1383	Ankrd26	8.157281962	7.806391628	8.49209369	3683	Cstb	8.108430477	8.061493706	7.974194913	5983	Gm14308	6.170688934	6.662897854	6.472005446	8283	Mamld1	2.058281135	1.875995227	1.98518948910583		Plekhj1	6.618949466	6.711294981	6.245996711	12883	Slc7a10	7.907302756	8.013378657	7.922471514	15183	Vil1	3.64013963	2.665177683	3.154454682
1384	Ankrd27	6.933771448	6.391580468	6.50372677	3684	Cstf1	5.694211042	5.772546376	5.107275215	5984	Gm14322	4.780741398	4.930589487	4.980869469	8284	Mamstr	2.395747523	2.163006546	2.30308177810584		Plekhm1	5.254296503	5.958085447	5.595035841	12884	Slc7a11	8.472667798	8.014603985	8.609511128	15184	Vill	0.081373598	1.410807927	3.308194898
1385	Ankrd28	6.287048682	6.747004912	5.979817629	3685	Cstf2	9.3020634	9.065774144	9.330960039	5985	Gm14325	6.209833606	6.282328118	6.488516962	8285	Man1a	5.699873852	6.047326649	6.15021866610585		Plekhm2	8.900853879	8.954239675	9.220951558	12885	Slc7a14	9.267593802	9.264676151	8.971407232	15185	Vim	8.28951859	8.322826469	8.088417241
1386	Ankrd29	7.077301109	7.062905318	6.638047067	3686	Cstf2t	8.560302258	8.527685729	8.572461723	5986	Gm14326	4.474065318	4.354449517	4.503385147	8286	Man1a2	10.45666157	10.35632987	10.5565001210586		Plekhm3	6.913356622	6.936396491	7.266084962	12886	Slc7a2	7.390945638	6.976167722	6.97983696	15186	Vimp	9.944439431	9.898968234	9.749822535
1387	Ankrd32	7.281044149	7.518786677	7.67389334	3687	Cstf3	7.288860176	6.964565335	7.056654263	5987	Gm14391	4.156124473	2.803848834	3.888632995	8287	Man1b1	6.847888832	6.81617445	6.65217954610587		Plekhn1	0.067381836	2.688473669	0.979458964	12887	Slc7a3	5.601311922	4.90908658	5.33726454	15187	Vip	6.288682665	6.159434035	6.749619703
1388	Ankrd33b	6.508005016	6.356117972	6.652472757	3688	Ctage5	7.868971804	7.870315979	8.150116603	5988	Gm14393	0.042852185	1.46112847	2.309027683	8288	Man1c1	7.033415218	6.627970639	7.01369256310588		Plekho1	7.339630483	7.788227385	7.893274028	12888	Slc7a4	7.27939942	7.514834347	7.109161225	15188	Vipas39	7.505173086	7.338666196	7.129490889
1389	Ankrd34a	7.78011951	7.585546869	7.963427281	3689	Ctbp1	9.667502115	9.755291718	9.767904216	5989	Gm14403	5.210669299	5.156413563	5.066657652	8289	Man2a1	6.274715406	6.631187857	6.9463268210589		Plekho2	4.267898972	5.122698622	5.37319274	12889	Slc7a5	4.600208062	4.423969733	4.565743582	15189	Vipr1	5.633523813	5.775263403	6.151881109
1390	Ankrd34b	6.695962913	6.476269297	7.057395984	3690	Ctbp2	7.962304046	7.984374406	7.479390361	5990	Gm14405	1.640008096	1.891498683	1.571002605	8290	Man2a2	8.419627753	8.301932166	8.12962908510590		Plekhs1	1.050306703	1.484376458	1.572868016	12890	Slc7a6	4.256676676	5.141249318	5.111455471	15190	Vipr2	1.095774004	1.400111014	1.563125805
1391	Ankrd34c	1.096012926	0.790614658	3.894278699	3691	Ctbs	4.769062369	4.496099553	3.562094003	5991	Gm14420	4.606463763	5.280826598	4.623342303	8291	Man2b1	6.488696209	6.541634382	6.4230646310591		Plg	4.85662787	3.530528616	6.550866614	12891	Slc7a6os	6.435972333	6.331576391	6.283882031	15191	Vit	5.932758691	5.957895652	5.439176157
1392	Ankrd35	5.799597307	5.300982698	5.566364879	3692	Ctc1	6.583397587	5.963257854	6.625138846	5992	Gm14430	4.477603917	4.841579157	4.787598689	8292	Man2b2	2.890529497	3.772144831	3.15059570810592		Plgrkt	5.69546096	6.277325909	5.594295793	12892	Slc7a7	3.408778696	4.319346515	4.152027797	15192	Vkorc1	7.335946341	7.365531722	6.760709803
1393	Ankrd37	4.004178418	4.63971484	5.110548478	3693	Ctcf	9.529644732	9.508531654	9.49962299	5993	Gm14431	5.399949985	4.870119772	5.372816101	8293	Man2c1	5.865156727	6.334313151	6.30242327310593		Plin2	5.722494155	5.748997063	5.503251127	12893	Slc7a8	7.917356866	7.698026878	7.503546446	15193	Vkorc1l1	6.740432622	6.935990526	7.180148358
1394	Ankrd39	4.985635933	5.408118778	5.150991983	3694	Ctdnep1	8.040173126	7.95148786	8.129927492	5994	Gm14434	4.477603917	4.841579157	4.787598689	8294	Man2c1os	0.035154742	2.223044702	0.98563456310594		Plin3	7.13629856	7.363641006	7.001986434	12894	Slc8a1	11.23086021	11.06659962	11.11825896	15194	Vldlr	9.270379758	9.245002036	9.308682063
1395	Ankrd40	9.61181301	9.632557618	9.622072451	3695	Ctdp1	5.574073949	6.207640606	5.735021589	5995	Gm14440	4.24497072	3.923686859	4.374191463	8295	Manba	4.003393364	4.279098683	5.30403920910595		Plin4	7.393011134	7.393225632	7.274510658	12895	Slc8a2	10.19545928	10.28124278	10.0661156	15195	Vma21	8.438366974	8.589918111	8.439356505
1396	Ankrd42	4.396653999	4.235496472	4.737654041	3696	Ctdsp1	6.117942131	5.724425466	5.471466511	5996	Gm14634	1.066608903	1.87890307	0.983091114	8296	Manbal	7.169078424	7.4983689	7.37240002110596		Plin5	3.064538266	3.694035351	2.98356228	12896	Slc8a3	6.387208352	6.758283186	7.089400162	15196	Vmac	5.368418203	5.450229543	5.838994929
1397	Ankrd44	5.324377277	5.370452445	5.067456793	3697	Ctdsp2	7.587540995	7.383782655	7.534690534	5997	Gm14827	0.069774039	1.854014619	1.565998161	8297	Manea	6.939924587	7.283195013	7.1092653410597		Plk1	3.07661636	3.012205443	2.563116611	12897	Slc8b1	3.242770486	3.444417741	2.786923494	15197	Vmn1r32	0.035134546	1.894912299	1.571456727
1398	Ankrd45	7.438658839	7.593527378	7.708174985	3698	Ctdspl	7.346652354	7.121436237	7.355206103	5998	Gm15217	4.063371936	3.5548322	3.98388325	8298	Maneal	6.997203636	6.906643007	6.60903008810598		Plk2	8.514312516	8.313742511	8.223091701	12898	Slc9a1	6.937793737	6.882728151	6.850347013	15198	Vmn2r29	3.402573379	3.148101739	4.153266727

1399	Ankrd46	9.766977875	9.728129687	9.564883371
1400	Ankrd49	8.039467444	7.942257688	7.748068071
1401	Ankrd50	7.498567211	7.495715366	7.694481869
1402	Ankrd52	3.07441198	3.447434523	2.563779284
1403	Ankrd54	6.033383656	5.968132323	5.862429697
1404	Ankrd55	2.100329449	3.312279865	3.683952568
1405	Ankrd6	7.931264821	7.584356153	7.838099498
1406	Ankrd63	5.606072422	5.907999055	6.441416089
1407	Ankrd66	3.765648338	3.960760525	3.441809382
1408	Ankrd9	5.297430642	5.862044714	5.838129319
1409	Anks1	6.634392035	6.685656581	6.28305403
1410	Anks1b	12.55681247	12.41469619	12.45114281
1411	Anks3	5.91711654	6.04341415	6.535416458
1412	Anks6	3.250053105	2.818854679	2.296084814
1413	Ankub1	4.154490923	3.291278932	3.681685136
1414	Ankzf1	6.304626131	5.812744667	6.209697859
1415	Anln	8.097665258	8.195643736	8.265493375
1416	Ano1	3.782469628	3.836877626	5.233216811
1417	Ano10	5.961595956	6.032321347	5.862706945
1418	Ano2	4.388247324	3.646750963	4.23077186
1419	Ano3	10.21773148	10.37310406	10.49571637
1420	Ano4	7.288874821	7.104149244	7.139863645
1421	Ano5	2.099654202	3.562892896	2.980425593
1422	Ano6	6.67829369	6.452365265	6.246201332
1423	Ano8	7.919342497	7.968363588	8.040928782
1424	Anp32a	11.00165276	11.05405067	10.83326527
1425	Anp32b	8.871544552	8.899008277	8.948748384
1426	Anp32e	10.02604739	10.11991745	10.1486866
1427	Anpep	3.082246825	3.159144512	3.682894824
1428	Antxr1	5.664374016	5.912278251	5.594731272
1429	Antxr2	2.407761675	3.451547098	3.304959114
1430	Anxa1	3.245219245	3.959209623	2.297795438
1431	Anxa11	4.334260262	4.5912266	4.935457604
1432	Anxa2	6.833413821	6.605813002	6.680575272
1433	Anxa3	5.983059312	5.028083501	5.623749781
1434	Anxa4	4.542025324	5.019589742	4.623712789
1435	Anxa5	7.925973878	7.936099503	8.046235808
1436	Anxa6	9.384486667	9.614837132	9.330862463
1437	Anxa7	7.455384646	7.504156429	7.346182053
1438	Anxa8	2.071457833	1.416518797	0.977780434
1439	Anxa9	6.619520957	6.994305378	6.455362566
1440	Aoc1	2.05268402	1.464908392	0.984607914
1441	Aoc2	2.07035151	2.453509238	1.982060594
1442	Aoc3	2.385301153	2.457624744	1.982646228
1443	Aox1	3.258098956	3.750385298	3.438554927
1444	Aox3	1.683511727	3.027957334	3.569708058
1445	Ap1ar	6.837029004	6.891025351	7.464586795
1446	Ap1b1	9.157208	9.109386368	9.169857635
1447	Ap1g1	9.072063181	8.897699152	9.201654458
1448	Ap1g2	6.031259356	5.816521496	6.068401803

3699	Ctdspl2	7.88246896	7.981523848	8.406576959
3700	Ctf1	3.07219747	2.844816764	3.792272786
3701	Ctgf	8.937711991	9.002110171	8.673475585
3702	Cth	3.417147186	4.086735296	3.297550927
3703	Cthrc1	4.081154138	4.234542366	3.435618308
3704	Ctif	8.022989108	7.997631876	7.968626782
3705	Ctla2a	6.203261401	5.47603348	5.623652898
3706	Ctla2b	2.089843535	2.417866108	2.788669291
3707	Ctnna1	8.081142099	8.168390922	8.480092252
3708	Ctnna2	9.347103827	8.978756677	9.40633926
3709	Ctnna3	3.076582993	3.872330952	1.97440771
3710	Ctnnal1	5.643791586	6.765442892	6.189112524
3711	Ctnnb1	11.39117563	11.34557419	11.24440679
3712	Ctnnbip1	10.42690356	10.3427923	10.24559132
3713	Ctnnbl1	7.057490196	7.143527931	7.078140979
3714	Ctnnd1	7.79628973	8.160290112	8.077995759
3715	Ctnnd2	10.23016445	10.13593798	10.26774315
3716	Ctns	4.839626664	5.401941422	5.109390078
3717	Ctps	6.404086135	6.494823129	6.761819187
3718	Ctps2	6.79148336	6.406774342	6.283320954
3719	Ctr9	9.146168179	9.071992761	8.937070147
3720	Ctsa	8.284948668	8.450429166	8.13469211
3721	Ctsb	10.42150433	10.3798477	10.39043308
3722	Ctsc	6.073585532	5.79879848	5.107574623
3723	Ctsd	10.54197506	10.6374077	10.20391451
3724	Ctsf	7.290208736	7.385598014	7.227756759
3725	Ctsh	6.455662906	6.867522386	6.208122555
3726	Ctsk	4.771195103	4.904895519	4.682464139
3727	Ctsl	8.669158428	8.686778662	8.475708009
3728	Ctso	6.36489201	6.001674708	5.979136518
3729	Ctss	9.657798958	9.506547346	9.42079205
3730	Ctsz	8.462582201	8.492237772	7.892667301
3731	Cttn	8.323778663	8.36946844	8.328707462
3732	Cttnbp2	9.270264865	9.075597605	9.070011716
3733	Cttnbp2nl	6.794470196	6.768744565	6.946170739
3734	Ctu1	5.694446192	5.954334877	5.564695929
3735	Ctu2	5.98736561	6.401592694	5.862254093
3736	Ctxn1	10.20847423	10.37751745	10.19917034
3737	Ctxn2	7.470552628	6.701142446	7.077891825
3738	Ctxn3	4.174497492	4.539678929	4.439146311
3739	Cubn	0.027284202	2.232688372	2.574721191
3740	Cuedc1	6.489716875	6.608697505	6.595008028
3741	Cuedc2	9.088360138	9.040890145	8.774338863
3742	Cul1	9.489890848	9.501544955	9.473847617
3743	Cul2	7.841943506	7.80935916	7.800150389
3744	Cul3	9.217447804	9.15288746	9.397960754
3745	Cul4a	7.326979744	7.316242386	6.707049501
3746	Cul4b	8.168311715	8.443026736	8.274645841
3747	Cul5	10.00679079	9.950242409	10.03935653
3748	Cul7	5.327138103	5.264732065	5.566118729

5999	Gm15328	2.367396208	0.885121138	1.571907219
6000	Gm15401	1.648032777	1.875934572	2.30806806
6001	Gm15417	5.292810179	5.776607652	5.565274263
6002	Gm15421	8.511539876	8.595614874	8.64174378
6003	Gm15446	3.084679746	3.154267796	4.232110538
6004	Gm15455	5.720650537	5.623771568	5.371990403
6005	Gm15612	1.657922329	2.459768737	0.979474006
6006	Gm1564	3.395236234	4.271536745	3.890724272
6007	Gm15645	2.070368468	1.848974554	2.305265517
6008	Gm15663	4.775551994	4.983806672	4.226733931
6009	Gm15694	5.077094281	5.276469155	5.374225128
6010	Gm15706	7.121203211	6.75792555	6.609158892
6011	Gm15708	0.040195714	2.217203763	0.984609819
6012	Gm15760	2.911354953	3.403149065	4.229669041
6013	Gm15787	2.655814872	1.388966264	3.154283711
6014	Gm15800	9.116339891	8.940985837	9.106778583
6015	Gm15816	5.029071205	4.842752297	5.711554332
6016	Gm15867	2.65883679	3.58049126	1.978120698
6017	Gm15880	1.043232938	0.910083205	0.989112292
6018	Gm15910	0.02993653	1.901880163	0.986730117
6019	Gm15972	2.082489097	3.194473817	2.566727857
6020	Gm16023	2.876498062	3.034643967	3.306405227
6021	Gm1604b	4.070956657	2.597014733	4.507579018
6022	Gm16062	7.7309742	7.65841213	7.609323502
6023	Gm16223	2.054149449	2.214276091	0.984092154
6024	Gm16287	2.424582858	3.761712835	4.231980934
6025	Gm16325	1.630820269	2.236083757	0.98788381
6026	Gm16381	3.266192236	4.615874676	4.567704524
6027	Gm16386	2.886771559	3.315216946	3.442271772
6028	Gm16523	2.089865409	3.883248486	2.98220693
6029	Gm1653	2.869943537	2.443577352	2.568146324
6030	Gm16532	3.264159055	3.126077989	3.980590507
6031	Gm16596	5.728501551	6.384546495	6.228465802
6032	Gm166	7.239045913	7.241333457	6.760729463
6033	Gm16617	2.647482321	0.811855096	3.308537643
6034	Gm16677	2.398330436	2.861565294	3.89361026
6035	Gm16701	2.878583893	2.147961566	1.560178309
6036	Gm16702	3.975900268	3.946402312	3.565920255
6037	Gm16712	2.399898437	3.191746181	3.153819028
6038	Gm1673	7.809214805	7.772732582	8.056924639
6039	Gm16740	4.734418812	5.805726322	4.978690002
6040	Gm16845	2.663815682	2.646908667	3.892392152
6041	Gm16853	6.492207909	6.81674901	7.002951826
6042	Gm16861	1.638221903	1.894962251	0.985646592
6043	Gm16894	5.164946092	5.307191481	5.024568615
6044	Gm16897	0.057635317	1.867528818	2.571056592
6045	Gm16938	3.984803562	3.739840483	4.229653484
6046	Gm16973	7.694632019	7.523587973	7.495546827
6047	Gm17066	7.774157394	7.6173128	7.831783003
6048	Gm17296	1.656689726	1.431209086	0.979869634

8299	Manf	8.361388133	8.48244419	8.17454378210599
8300	Mansc1	4.169862359	4.604513739	3.43470112910600
8301	Mansc4	4.229182537	2.628080929	3.68360294110601
8302	Maoa	8.214657835	8.17349727	8.35088968610602
8303	Maob	7.319905046	7.42233297	7.57261585410603
8304	Map10	4.467696304	4.905643779	4.22808411710604
8305	Map1a	12.93313092	12.86148429	13.0889969110605
8306	Map1b	13.94206067	13.76219088	13.6602214510606
8307	Map1lc3a	10.31177646	10.43521988	10.3723204810607
8308	Map1lc3b	10.0607847	10.15758261	10.058114910608
8309	Map1s	7.453252344	7.257514701	7.11952232110609
8310	Map2	12.91367412	12.70613991	12.9695536210610
8311	Map2k1	9.420142745	9.329822308	9.18693975810611
8312	Map2k2	8.245006392	8.204686777	8.30658813210612
8313	Map2k3	5.834215451	5.444200743	5.8873397910613
8314	Map2k3os	1.077137758	1.862023042	2.79363329310614
8315	Map2k4	9.941415597	9.843606071	9.74815194810615
8316	Map2k5	5.911217509	5.863212102	5.53384377810616
8317	Map2k6	5.079733569	4.734510147	5.33871431910617
8318	Map2k7	8.074876998	8.192037532	8.12980200310618
8319	Map3k1	5.636624595	6.014414843	6.00317381510619
8320	Map3k10	7.814523266	8.016119344	7.94032788810620
8321	Map3k11	4.669202931	5.34389972	5.06824609110621
8322	Map3k12	9.827039561	9.727862603	9.850367310622
8323	Map3k13	5.256133791	5.477655197	5.95835238510623
8324	Map3k14	3.073976191	3.175690802	2.29934853710624
8325	Map3k15	7.866731848	7.652578824	7.38910090510625
8326	Map3k19	3.893140235	4.60874271	4.50516626210626
8327	Map3k2	7.913338376	7.767334242	8.19465677710627
8328	Map3k3	6.911287143	6.289734377	6.94636372610628
8329	Map3k4	8.235063599	8.235021407	8.34211252410629
8330	Map3k5	7.794055077	7.775678315	7.99723273210630
8331	Map3k6	4.239413954	4.021592965	4.23056983410631
8332	Map3k7	8.091964642	8.051469165	8.32891900310632
8333	Map3k8	0.047869952	0.858759056	1.5693320210633
8334	Map3k9	6.750846753	6.538408295	6.82615659910634
8335	Map4	11.13588806	11.14951324	11.2177946210635
8336	Map4k1	1.657933763	1.856695127	1.56635686310636
8337	Map4k2	5.987820394	6.217359487	6.13006309310637
8338	Map4k3	7.420212534	7.574772766	7.51934752310638
8339	Map4k4	10.75822711	10.83946702	10.6129618710639
8340	Map4k5	7.427934059	7.455417018	7.04572496510640
8341	Map6	8.034359717	8.077357138	8.15514256910641
8342	Map6d1	7.251649109	7.445476915	7.45577602810642
8343	Map7	8.304952521	8.422926262	8.52312406510643
8344	Map7d1	9.375041631	9.357657888	9.29512654510644
8345	Map7d2	11.23532856	10.98035161	11.07536910645
8346	Map9	10.65022326	10.40091627	10.4648099110646
8347	Mapk1	10.69987775	10.68248244	10.6506274610647
8348	Mapk10	11.77267009	11.65404333	11.6395371910648

Plk3	5.747251295	4.911191376	5.406478134
Plk4	4.9375903	4.58194736	4.886888579
Plk5	5.580360769	6.159542173	6.302283976
Pllp	9.402506068	9.842151225	9.308608193
Pln	3.664535672	3.5344788	3.981708258
Plod1	5.573028288	5.417113668	5.503144255
Plod2	4.940828565	5.493812807	4.678673722
Plod3	6.272141681	6.774921018	6.487921089
Plp1	14.42267763	14.49038302	14.18706145
Plp2	2.064588798	2.692489075	0.980334403
Plrg1	8.817567949	8.43358278	8.301734433
Pls1	5.670839338	6.285574455	6.088572007
Pls3	9.809787892	9.702407159	9.517172683
Plscr2	3.768065779	3.435966671	3.89051558
Plscr3	5.035496684	5.085861646	5.302768256
Plscr4	4.327589045	4.658074493	4.936898484
Plscr5	0.011144827	0.927238664	1.576210015
Pltp	8.080292317	8.278778317	7.85014147
Plvap	5.438338701	5.893631591	5.762396292
Plxdc1	5.292524176	5.452443633	5.888317446
Plxdc2	7.841890376	7.603924216	7.934351005
Plxna1	7.452871269	7.095459953	6.886492129
Plxna2	9.196624763	9.173638025	9.172331104
Plxna3	5.201792548	4.975760803	4.147117657
Plxna4	9.083098028	9.114622266	8.819081365
Plxnb1	7.391230516	7.133061919	6.991018363
Plxnb2	7.022546566	7.25153718	6.564072767
Plxnb3	4.731672758	5.368229041	5.536034826
Plxnc1	7.424575712	6.615524426	7.035704298
Plxnd1	5.81027281	5.788029663	6.209573929
Pm20d1	2.083556197	2.155363931	2.983361103
Pm20d2	5.940221113	6.30226087	5.760348774
Pmaip1	3.378650848	1.399788255	2.791038128
Pmch	3.975282461	3.657448319	3.98297779
Pmel	6.099132348	5.838808607	5.564138988
Pmepa1	9.069601125	9.320068445	9.447713837
Pmf1	4.839580912	5.551897978	5.625821005
Pmfbp1	1.094594811	2.671846657	3.571547578
Pml	5.086252388	5.677291415	5.762463837
Pmm1	9.180841969	8.822608133	9.056817164
Pmm2	6.145293269	6.319852356	6.088672224
Pmp22	6.768063436	6.813999135	7.140371869
Pmpca	8.091807672	8.213852812	8.139904461
Pmpcb	8.131515967	8.441741564	8.459818608
Pms1	5.530694549	5.010634762	4.934242537
Pms2	6.144338318	6.127188769	6.228706147
Pmvk	7.555247836	7.368878403	7.227629921
Pnck	9.387531808	8.734973884	8.731435813
Pnisr	10.39339901	10.2868401	10.35719682
Pnkd	9.162750908	9.009471468	9.201584715

12899	Slc9a2	2.873725103	3.196890375	2.302847309
12900	Slc9a3r1	7.145141335	6.977683918	6.774012535
12901	Slc9a3r2	7.318665833	7.362519565	7.218287439
12902	Slc9a4	5.797387493	5.553480632	3.429926246
12903	Slc9a5	4.415789114	4.396718419	5.440673416
12904	Slc9a6	9.691280598	9.519043495	9.566843469
12905	Slc9a8	4.678495799	5.259908506	5.681411539
12906	Slc9a9	6.189965789	6.193780653	6.610216727
12907	Slc9b1	1.643311125	1.461140719	1.986250309
12908	Slc9b2	1.663156716	1.416515692	2.305012167
12909	Slco1a1	2.396482125	3.040376463	2.790478293
12910	Slco1a4	7.572215463	7.63330798	7.572490142
12911	Slco1a5	2.680847232	4.190196489	3.30179583
12912	Slco1b2	2.376468553	0.854673318	1.985193678
12913	Slco1c1	8.402091143	8.57789313	7.973990656
12914	Slco2a1	2.666501099	3.449433967	2.787683701
12915	Slco2b1	5.807798665	5.907642447	5.934744292
12916	Slco3a1	8.510221696	8.22521686	8.199045507
12917	Slco4a1	2.411362106	2.404160424	3.891613163
12918	Slco4c1	1.082214671	2.884487662	2.569766427
12919	Slco5a1	3.76866848	0.682470967	3.15002555
12920	SlcUbl4a	4.72857799	5.128453931	5.150668012
12921	Slfn2	4.078427159	2.958779001	3.786375854
12922	Slfn5	5.725651079	5.906122043	5.623185863
12923	Slfn8	0.067521245	2.188576912	2.793144352
12924	Slfn9	2.065796507	2.461823774	2.570267525
12925	Slfnl1	0.013871431	1.92610674	1.575588401
12926	Slirp	9.085909843	8.925004662	8.764471503
12927	Slit1	6.693198198	6.844327957	6.389131434
12928	Slit2	7.487449382	7.150918259	7.246972091
12929	Slit3	4.166249222	3.730155111	4.302959165
12930	Slitrk1	8.214485968	8.200594051	8.320063867
12931	Slitrk2	7.540779957	7.664292752	7.572298967
12932	Slitrk3	8.787208455	8.680865085	8.51909876
12933	Slitrk4	8.167637047	7.852540586	8.297626348
12934	Slitrk5	8.868490457	9.053054057	8.784049693
12935	Slitrk6	2.696305601	2.33958019	4.791190097
12936	Slk	10.03459773	9.897099799	9.958707745
12937	Slmap	9.141364808	9.031880126	9.32879231
12938	Slmo1	6.975880577	7.133876242	7.440135344
12939	Slmo2	8.016503871	7.941718552	7.892891967
12940	Sln	4.313137482	4.631228851	3.565453528
12941	Sltm	10.32006813	10.2007755	10.12588251
12942	Slu7	9.609989864	9.574113751	9.559279068
12943	Slx1b	6.246430261	5.90372821	5.67966113
12944	Slx4	5.573265555	5.618769557	5.438716397
12945	Slx4ip	3.983523864	4.016235601	3.563756323
12946	Smad1	7.012885419	7.298350752	7.346529975
12947	Smad2	7.046231062	6.976516954	6.812423397
12948	Smad3	7.848554586	7.737925238	7.886970173

15199	Vmn2r46	0.029919447	1.48026443	2.990197552
15200	Vmn2r87	2.054182998	1.461123232	2.572739866
15201	Vmn2r-ps129	2.051259459	0.87602301	1.571008032
15202	Vmp1	9.323805182	9.28462418	9.465869326
15203	Vnn1	2.044897263	0.894748756	1.988643905
15204	Vopp1	8.506484936	8.449092112	8.594853271
15205	Vprbp	7.473841359	7.776044781	7.844465403
15206	Vps11	6.792217908	6.566557193	6.487690371
15207	Vps13a	8.759788392	8.598875636	8.960330331
15208	Vps13b	7.957324402	7.881046008	8.165037311
15209	Vps13c	7.908322377	8.051168578	8.337711667
15210	Vps13d	7.738843899	7.821538467	7.734849814
15211	Vps16	7.165332192	6.718391697	6.862677358
15212	Vps18	6.507725575	6.578944997	6.934930849
15213	Vps25	8.600589828	8.863788631	8.922647038
15214	Vps26a	8.051065974	7.91531932	8.002545788
15215	Vps26b	9.363507533	9.333829578	9.361561147
15216	Vps28	10.12502136	10.09856404	10.13605572
15217	Vps29	8.763185743	8.541050865	8.754854751
15218	Vps33a	7.822256269	7.837553503	8.265849978
15219	Vps33b	5.906780112	5.802171395	5.337227353
15220	Vps35	10.33781667	10.19420926	10.18820818
15221	Vps36	7.455077185	7.468322956	7.346256184
15222	Vps37a	9.571978377	9.286691674	9.469889085
15223	Vps37b	7.023838164	7.324481245	7.218569854
15224	Vps37c	3.780496561	3.640087738	4.375066472
15225	Vps37d	6.309308547	6.409864484	6.227704204
15226	Vps39	7.879073193	7.406831832	7.455686647
15227	Vps41	9.596253495	9.646979605	9.536466225
15228	Vps45	7.177926779	7.160753883	6.968428912
15229	Vps4a	8.669746553	8.863196524	8.957359317
15230	Vps4b	8.046532986	8.118874462	8.124685138
15231	Vps51	8.184191044	8.39339042	8.279207573
15232	Vps52	6.291132734	6.526964269	6.283473403
15233	Vps53	8.249587426	8.222993724	8.046068976
15234	Vps54	5.913991896	5.811472598	6.302905577
15235	Vps72	8.919798187	8.773617556	8.988451308
15236	Vps8	6.364146169	6.177093367	6.088178115
15237	Vps9d1	6.590537728	7.030997706	6.887013542
15238	Vrk1	6.387496519	6.939931036	6.487274059
15239	Vrk2	5.401109762	5.731874405	5.681721527
15240	Vrk3	6.148364258	6.598691079	6.565635595
15241	Vsig10	2.876563052	2.658798722	2.983181952
15242	Vsig10l	3.646927128	3.180599182	3.152452087
15243	Vsig2	3.982055823	2.350704432	4.684529208
15244	Vsig8	0.069787646	3.058959721	2.569770864
15245	Vsnl1	11.35088689	11.31549486	11.4409577
15246	Vstm2a	9.99644006	9.807418838	9.931247434
15247	Vstm2b	6.170705083	6.554936081	6.354885077
15248	Vstm2l	7.549956838	7.755566969	8.209202872

1449	Ap1m1	7.240153118	7.556645404	7.414977256	3749	Cul9	5.93540771	5.721013894	5.934937289	6049	Gm17750	4.663536104	4.353773136	4.735655306	8349	Mapk11	5.471622095	5.598509454	5.86422399410649	Pnkp	5.630862219	4.820674152	5.565765686	12949	Smad4	7.968427431	7.725795597	7.774365894	15249	Vstm4	4.178888988	4.411549831	4.88760668
1450	Ap1m2	0.035108834	1.894946012	2.79656716	3750	Cuta	7.842559855	7.98812279	7.980046046	6050	Gm17757	1.056762482	0.880568547	2.310061949	8350	Mapk12	2.101979485	3.167102289	3.56775347910650	Pnky	0.024632389	1.488687267	1.573374083	12950	Smad5	8.219754194	8.127757097	8.134799095	15250	Vstm5	5.206701862	4.124862092	5.441018188
1451	Ap1s1	9.529185436	9.329880737	9.139777294	3751	Cutal	1.649433618	2.473254522	0.982173116	6051	Gm17762	4.394990821	3.396258775	3.886322539	8351	Mapk14	7.309671058	7.361515563	7.50379186810651	Pnlip	1.05249822	2.496933753	1.572387232	12951	Smad6	1.697549307	3.870839699	4.072356852	15251	Vsx1	0.024622223	2.236126501	2.311590298
1452	Ap1s2	8.885075454	8.945955513	9.078049689	3752	Cutc	5.696038565	5.668481944	5.734814763	6052	Gm17769	2.068173438	2.457485431	1.565984233	8352	Mapk15	3.068450358	2.853178783	3.15320249110652	Pnma1	3.772295322	3.546471787	4.153588473	12952	Smad7	6.976509161	7.283091986	7.129754845	15252	Vta1	7.401453518	7.297844894	7.077695668
1453	Ap1s3	3.975572522	4.189237835	3.682036339	3753	Cux1	8.109403563	8.246575611	8.666786447	6053	Gm1821	12.41512485	12.47423894	12.25783992	8353	Mapk1ip1	6.861250096	6.719298854	7.09956743110653	Pnma2	9.212002251	8.878649124	9.03797178	12953	Smad9	5.539628599	5.750955476	5.301107435	15253	Vti1a	8.069143299	8.090873656	8.228110162
1454	Ap2a1	7.319711281	7.83680632	7.572663802	3754	Cux2	5.602082391	5.511593988	5.981675052	6054	Gm1821	10.59143232	10.47858027	10.4985913	8354	Mapk1ip1l	6.740849986	7.233919457	6.87459095610654	Pnma3	6.327686651	6.138998073	6.389731978	12954	Smagp	3.898736426	4.985870735	5.268750678	15254	Vti1b	10.55115507	10.53488852	10.555541
1455	Ap2a2	9.723843251	9.552550375	9.164724914	3755	Cwc15	9.889376134	9.75058566	9.741515365	6055	Gm18853	1.056762482	0.880568547	2.310061949	8355	Mapk3	10.29787789	9.930833105	9.76131295510655	Pnmal1	8.741429256	8.426177869	8.354911575	12955	Smap1	8.984129745	9.043282733	8.892888066	15255	Vtn	7.54020699	6.827615693	7.768161524
1456	Ap2b1	9.848751856	9.975165115	9.800144152	3756	Cwc22	7.225492994	6.492899391	6.623757007	6056	Gm19345	3.652429782	3.56023943	2.980035064	8356	Mapk4	7.126203333	7.401447966	7.34639378110656	Pnmal2	11.32545558	11.0885609	11.14362276	12956	Smap2	9.543974691	9.503225756	9.592902517	15256	Vwa1	7.357615641	7.209169831	7.638405499
1457	Ap2m1	9.51911466	9.454582306	9.463831632	3757	Cwc25	7.2699617	7.131301596	7.218400768	6057	Gm1943	5.122354398	5.128274088	4.786563283	8357	Mapk6	7.413895503	7.681851617	8.14527898710657	Pnn	9.014625111	8.886351023	9.244473545	12957	Smarca1	9.567964178	9.331596574	9.383011988	15257	Vwa3a	4.097724217	5.308952467	4.934229226
1458	Ap2s1	9.233488181	8.994962346	8.292597906	3758	Cwc27	7.626495741	7.391402476	7.463785709	6058	Gm19522	4.256685429	4.286455996	4.888218812	8358	Mapk7	5.471170844	5.9403936	4.78489182410658	Pno1	6.925074398	6.815123169	6.825500236	12958	Smarca2	11.53588603	11.43765259	11.62159623	15258	Vwa5a	5.729410572	6.50429698	6.18966898
1459	Ap3b1	8.531445488	8.308536575	8.367933568	3759	Cwf19l1	5.294777523	5.698275985	5.935252876	6059	Gm1966	1.657885487	2.188858482	1.982959422	8359	Mapk8	9.58609505	9.441445915	9.65604021810659	Pnoc	5.080248002	5.464251348	5.150258462	12959	Smarca4	9.446759623	9.41157297	9.469874063	15259	Vwa5b2	5.404903664	5.322572018	5.958536631
1460	Ap3b2	10.2149514	10.15063107	10.19430674	3760	Cwf19l2	8.531293925	8.41914276	8.455766421	6060	Gm19705	3.236892734	2.142377498	2.788645303	8360	Mapk8ip1	9.882796706	9.897739985	9.64351495410660	Pnp	6.348840545	6.621697452	6.108228754	12960	Smarca5	9.165170624	8.836588624	9.162425625	15260	Vwa7	1.660648603	1.422233362	3.572690673
1461	Ap3d1	9.866618543	9.835311531	9.754779587	3761	Cwh43	3.65734384	3.950723174	3.440379816	6061	Gm19710	4.775987193	5.14368428	4.300986167	8361	Mapk8ip2	8.877966081	8.957674687	8.97729458710661	Pnpla1	0.013875906	1.926099189	0.990438216	12961	Smarca5-ps	7.269803397	7.282021905	7.346578906	15261	Vwa8	6.605603799	6.21955331	7.002861316
1462	Ap3m1	7.587974545	7.375136344	7.526879606	3762	Cx3cl1	10.50473923	10.50669755	10.63196094	6062	Gm1976	8.062204088	7.478179186	8.125040626	8362	Mapk8ip3	9.297248709	9.385190504	9.237354410662	Pnpla2	6.128909306	6.510845207	6.455657159	12962	Smarcad1	7.035126198	6.51476065	7.20904017	15262	Vwa9	5.727938906	5.966487145	6.424129813
1463	Ap3m2	9.964329691	9.795183156	9.701078852	3763	Cx3cr1	9.45523316	9.518232458	9.242056806	6063	Gm19897	0.081461064	2.175305569	2.304502154	8363	Mapk9	10.16282482	9.965134207	10.5250220210663	Pnpla3	3.670959965	4.241611594	4.683669099	12963	Smarcal1	7.489779477	7.090264716	7.602248407	15263	Vwc2	4.938268239	4.635002808	5.068034526
1464	Ap3s1	6.647911053	6.468497164	6.319616612	3764	Cxadr	2.886753782	3.675271227	2.787061274	6064	Gm2002	6.623827087	7.266562986	7.588159743	8364	Mapkap1	8.081044943	8.204272176	8.19916385710664	Pnpla6	6.490514058	6.509548912	6.264655043	12964	Smarcb1	7.573009594	7.793299353	7.838189531	15264	Vwc2l	5.16061004	4.932528885	4.887623528
1465	Ap3s2	8.172768597	8.123843321	8.119610762	3765	Cxcl12	7.147077345	7.371458432	6.968223359	6065	Gm20063	3.246161191	4.042645918	3.150462413	8365	Mapkapk2	7.056624372	6.825106054	6.78707714210665	Pnpla7	4.473586374	4.93925702	3.675836753	12965	Smarcc1	9.435459191	9.257907137	9.306456053	15265	Vwf	4.470433791	3.500966006	3.784385785
1466	Ap4b1	6.249324051	6.266629882	6.228133556	3766	Cxcl14	8.871690386	8.811980429	8.925474069	6066	Gm2011	1.64645828	1.878989536	2.572154085	8366	Mapkapk3	1.700990722	3.668491761	4.07198395410666	Pnpla8	9.105790665	9.05416739	9.189465948	12966	Smarcc2	10.74511228	10.79481713	11.09506933	15266	Wac	9.393274385	9.161363616	9.27228418
1467	Ap4e1	4.264877212	5.049309307	4.786486599	3767	Cxcl16	3.075440484	3.874164093	1.556834646	6067	Gm20172	2.386398857	2.183993621	2.569518619	8367	Mapkapk5	7.19012079	7.20237919	7.38946792710667	Pnpo	7.348140686	7.367976905	7.526972321	12967	Smarcd1	7.745683282	7.775171351	7.969008799	15267	Wapal	9.330926928	9.263696062	9.467877895
1468	Ap4m1	5.168149209	5.710531865	4.562167512	3768	Cxcl5	4.707382285	4.249264657	4.069112631	6068	Gm20187	2.641837067	1.434273393	2.306589082	8368	Mapkbp1	7.779359413	7.552624882	7.80696624210668	Pnpt1	5.606027244	5.840329549	6.089585635	12968	Smarcd2	6.252186936	6.497854515	6.58033376	15268	Wars	7.419413652	7.289212167	7.565417172
1469	Ap4s1	8.011508892	8.114461408	8.23757068	3769	Cxcr2	2.058279015	1.875999934	1.568936895	6069	Gm20199	2.071511444	1.41637722	2.792261785	8369	Mapre1	10.22507525	10.25496438	9.99396720110669	Pnrc1	7.94484446	7.859199138	7.974350291	12969	Smarcd3	7.670311974	7.054058321	7.439716268	15269	Wars2	5.531357148	5.43675811	5.266570014
1470	Ap5b1	2.054170108	2.214234962	0.984084863	3770	Cxcr4	4.823795679	4.994252446	3.677888729	6070	Gm2022	0.019324886	0.910035794	0.989106681	8370	Mapre2	10.44671407	10.28281645	10.4031301810670	Pnrc2	8.188578257	8.196390894	7.934016171	12970	Smarce1	7.932251419	7.837018984	8.129976525	15270	Was	3.63957204	2.866099413	2.303077365
1471	Ap5m1	6.20827971	6.318841113	6.488893001	3771	Cxcr5	3.682425033	4.291675621	5.070019474	6071	Gm20257	2.082702219	2.661889043	1.978840317	8371	Mapre3	9.444670715	9.693631968	9.5441879910671	Poc1a	5.428526614	5.054993932	5.110469861	12971	Smc1a	10.85367994	10.77806674	10.73067241	15271	Wasf1	9.648019704	9.419121423	9.350649714
1472	Ap5s1	6.753624642	6.602089346	7.256958624	3772	Cxx1a	9.907310371	9.975912975	10.24917457	6072	Gm2027	4.87905458	4.757895249	4.736469277	8372	Mapt	12.06247264	12.04302309	11.9356266310672	Poc1b	5.93946022	5.737807483	6.109556257	12972	Smc1b	3.547804368	5.271648876	3.436841794	15272	Wasf2	7.034643438	7.405471064	7.013393319
1473	Ap5z1	5.571978121	5.725313753	5.813781898	3773	Cxx1b	10.12102786	10.27664774	10.44560093	6073	Gm20300	7.999346604	8.037993425	8.18967094	8373	Marc2	8.709407846	8.671435361	8.43520500210673	Poc5	6.369261822	6.657370055	7.057572891	12973	Smc2	8.167073175	8.018210826	7.734581059	15273	Wasf3	6.435917335	6.212322864	6.63922126
1474	Apaf1	5.978449854	5.330044915	5.023192319	3774	Cxx1c	9.844025276	9.869480354	10.27813027	6074	Gm20319	3.776135619	3.538006612	3.565083069	8374	March1	8.129417402	7.925296974	8.0676169110674	Podn	3.532886712	2.821370236	3.790186776	12974	Smc2os	3.527796263	3.011938305	1.974347205	15274	Wash1	6.67841929	6.608593676	6.624210605
1475	Apba1	9.681843833	9.49270492	9.625735923	3775	Cxxc1	7.463686284	7.674266616	7.519370066	6075	Gm20594	7.902593369	8.074287371	8.627724126	8375	March10	1.69101671	4.131927076	3.56874597210675	Podxl	6.081089487	6.350464526	6.423739939	12975	Smc3	10.39676801	10.33869056	10.26305484	15275	Wasl	9.247983092	9.3097577	9.249111478
1476	Apba2	8.575813441	8.675649227	8.439441392	3776	Cxxc4	8.280175904	8.055277144	8.691179513	6076	Gm20604	7.986897177	7.826003252	8.002504827	8376	March11	4.898458172	6.020356681	5.59496173110676	Podxl2	5.779361401	5.670957019	5.680491337	12976	Smc4	7.628340461	8.049847814	7.680284334	15276	Wbp1	7.494558227	7.11607368	6.608539885
1477	Apba3	5.98936627	6.178402968	5.83701484	3777	Cxxc5	9.020225542	9.23527777	9.239839909	6077	Gm20605	2.672096999	3.67082295	3.303559989	8377	March2	8.235924528	8.434477628	8.4598476510677	Pof1b	1.075526842	2.466192373	3.157478573	12977	Smc5	7.644128228	7.820175806	8.040867678	15277	Wbp11	8.899719994	8.6325614	8.605712768
1478	Apbb1	10.5120289	10.54011135	10.56402775	3778	Cyb561	7.046036494	7.189224705	6.837615377	6078	Gm2061	3.661309331	2.807319981	4.153067191	8378	March4	6.055966541	5.98953382	6.33877263910678	Pofut1	5.636391067	5.817448757	6.110455032	12978	Smc6	9.823825537	9.831998065	9.867194695	15278	Wbp1l	7.374884044	7.446616012	7.439593343
1479	Apbb1ip	6.674204274	6.179700226	6.067383572	3779	Cyb561a3	6.348128345	6.596098962	6.722179777	6079	Gm20735	4.602902649	4.848134793	5.069610941	8379	March5	8.173661834	8.229957108	8.21356346510679	Pofut2	6.538116696	6.329024669	6.826589472	12979	Smchd1	7.849166914	7.693444161	8.035391413	15279	Wbp2	10.03466411	10.02657168	9.925413722
1480	Apbb2	8.720618304	8.657978892	8.711342066	3780	Cyb561d1	5.438612283	5.596093268	5.681147334	6080	Gm20751	1.083769148	2.882752411	2.792706925	8380	March6	9.043239093	8.977459039	9.12983852410680	Pogk	8.720750645	8.648066421	8.741565491	12980	Smco3	5.988052085	6.144050548	6.024342354	15280	Wbp4	9.386462042	9.357650782	9.501681548
1481	Apbb3	7.219206831	6.911744748	7.247039169	3781	Cyb561d2	6.599260847	6.42869863	6.338060313	6081	Gm20754	4.605930759	5.05857008	4.935144042	8381	March7	6.873000463	6.841003235	6.77466509710681	Poglut1	8.075029487	7.9659289	7.98547975	12981	Smco4	5.077663291	5.375446883	5.024868779	15281	Wbp5	10.87652925	10.75962444	10.79697019
1482	Apc	11.10299118	11.02744475	11.34140201	3782	Cyb5a	8.379382072	8.25546389	8.324321024	6082	Gm2083	7.554667364	3.249646517	6.064582209	8382	March8	8.183520718	8.261133447	7.96281091510682	Pogz	7.759245695	7.677641761	7.714825254	12982	Smcp	2.916631306	5.665486393	3.144608161	15282	Wbscr16	5.403431362	5.676397348	5.503681301
1483	Apc2	6.861349365	7.11904049	7.046588901	3783	Cyb5b	9.611845673	9.60285141	9.66478006	6083	Gm20878	5.079563976	5.23672316	5.150425773	8383	March9	4.667014107	5.209639926	4.5024070810683	Pola1	6.269151232	6.365708897	6.265674861	12983	Smcr8	5.786054328	6.213600473	6.38994669	15283	Wbscr17	8.415306352	8.591811478	7.979688001
1484	Apeh	6.054319166	5.839322969	5.787366019	3784	Cyb5d1	0.081367071	2.447557141	2.304512256	6084	Gm20939	2.40079545	3.190326648	3.444261139	8384	Marcks	10.41038661	10.4066755	10.4301634410684	Pola2	4.659322687	4.988864307	4.566060321	12984	Smdt1	9.691408397	9.555378482	9.522985638	15284	Wbscr22	6.740806276	6.716222959	7.150253834
1485	Apela	4.368484061	1.394131236	1.56244853	3785	Cyb5d2	6.807838309	6.917656055	6.862900968	6085	Gm2109	0.021986974	1.913249801	1.98954508	8385	Marcksl1	7.419123782	7.014538059	7.28384897610685	Polb	8.723718446	8.470491176	8.426986422	12985	Smek1	7.62013719	7.769391048	7.934501408	15285	Wbscr25	1.073770166	2.696624192	0.981227564
1486	Apex1	7.821053642	7.72741405	7.572330276	3786	Cyb5r1	7.098301027	6.565491103	6.336921819	6086	Gm21119	0.052844291	1.447341515	3.310522554	8386	Marf1	9.62559165	9.688099317	9.77931462710686	Pold1	4.393256449	3.926240792	4.068270639	12986	Smek2	9.306779833	9.001734927	9.265411322	15286	Wbscr27	6.075332116	5.887734128	6.131155855
1487	Apex2	4.23638438	3.540896044	4.153020996	3787	Cyb5r3	8.485125111	8.377293366	8.324269666	6087	Gm2115	6.402967707	6.648986865	6.595056796	8387	Mark1	8.393404604	8.430626078	8.55757083810687	Pold2	5.2553627	5.418474484	5.863601538	12987	Smg1	8.619468348	8.387489209	8.714831876	15287	Wdfy1	8.05250103	8.222215826	8.062049364
1488	Aph1a	8.189662678	8.532776275	8.297367081	3788	Cyb5r4	6.711921912	6.946502767	7.18992824	6088	Gm21586	4.252258007	4.854788307	4.889165695	8388	Mark2	8.08495004	8.203104484	8.13515759310688	Pold3	6.70738039	5.997757788	6.503675166	12988	Smg5	6.213231624	6.495508779	6.680802225	15288	Wdfy2	2.378805271	2.470825899	3.448150311
1489	Aph1b	8.396791047	7.93422366	8.213705266	3789	Cyb5rl	3.38352177	3.033288366	2.301782993	6089	Gm21949	10.76849756	10.36709956	10.39465659	8389	Mark3	7.956754395	7.925600619	7.9913315510689	Poldip2	8.063214078	8.156683815	8.013432648	12989	Smg6	7.651010518	7.57053416	7.991513208	15289	Wdfy3	8.990455376	9.0766558	9.204118251
1490	Aph1c	5.079057466	5.30681444	5.109749001	3790	Cyba	5.073663884	4.696903817	3.88276667	6090	Gm21975	1.653747121	1.864837846	2.570799963	8390	Mark4	3.401390937	4.189194471	3.78942999210690	Poldip3	8.572314683	8.727734046	8.568720598	12990	Smg7	10.11391922	10.18685563	10.20884895	15290	Wdfy4	2.084420036	2.658990792	3.570392901
1491	Api5	9.202406135	9.297079316	9.263031658	3791	Cybb	3.074870925	3.446594813	3.442429766	6091	Gm21992	7.165850912	6.802002287	6.665973985	8391	Mars	8.748969928	8.727329982	8.61298701910691	Pole	2.391163999	3.205576962	1.564030893	12991	Smg8	4.940433383	5.552637823	5.682673715	15291	Wdhd1	4.467304666	3.255012884	4.56671189
1492	Apip	5.253958743	5.824253736	5.68113551	3792	Cybrd1	2.672506305	3.773090041	3.791008897	6092	Gm22109	2.656470704	2.662155981	2.56671624	8392	Mars2	4.244890977	3.266046021	4.22919526810692	Pole2	2.058356494	0.854388884	0.982595562	12992	Smg9	5.537296089	5.514698481	5.265035491	15292	Wdpcp	4.541300253	4.979003222	4.981514033
1493	Apitd1	5.317046857	4.592387217	5.069364345	3793	Cyc1	9.386557371	9.417387964	9.441507784	6093	Gm22291	2.066950888	1.856653725	2.306059538	8393	Marveld1	4.786022805	5.523323325	4.78611647410693	Pole3	6.949483622	6.883863297	6.761291627	12993	Smim1	7.88082404	7.726185951	7.81294097	15293	Wdr1	9.019877323	9.074603482	8.892853228
1494	Aplf	4.833897467	4.530403725	4.980954461	3794	Cycs	10.0562267	9.9385181	9.873266457	6094	Gm2518	4.929270327	5.108563669	4.736377311	8394	Marveld2	2.068138297	2.686486703	0.97902278810694	Pole4	7.271467052	7.518396651	7.479911678	12994	Smim10l1	5.839322692	6.064836086	6.302355724	15294	Wdr11	5.760245851	6.484674806	6.624565252
1495	Apln	7.126345286	7.202620116	7.4880609	3795	Cyct	1.624501433	0.915558246	0.989760735	6095	Gm26391	1.641618934	1.888375198	0.984605092	8395	Marveld3	1.056720812	2.71768804	1.98740484910695	Polg	6.19184929	6.45280011	6.826385554	12995	Smim10l2a	7.269523616	7.387899677	6.979651553	15295	Wdr12	7.731637806	7.774868995	7.780945022
1496	Aplnr	0.021973375	2.239654217	0.988490639	3796	Cyfip1	9.032064557	8.986952117	8.910592308	6096	Gm266	5.594242388	4.580041786	4.73399002	8396	Mas1	6.076044788	5.952015654	5.4709414910696	Polg2	2.875147869	2.661960213	3.444697128	12996	Smim11	7.723434296	7.443788226	7.735125376	15296	Wdr13	9.745588552	9.589429918	9.568697646
1497	Aplp1	11.6046318	11.68688271	11.46780422	3797	Cyfip2	12.156059	12.16169881	12.2846272	6097	Gm2694	5.884912125	6.171979333	4.931229574	8397	Masp1	2.377702299	2.203344195	2.79460426510697	Poli	5.907825063	5.51067293	5.534717434	12997	Smim12	8.101948653	7.920146408	7.996907684	15297	Wdr17	7.270447373	7.330356968	7.355153417
1498	Aplp2	10.70916213	10.83350071	10.93127572	3798	Cygb	9.519602223	9.539610123	9.759747253	6098	Gm28042	4.334955181	4.843700472	5.340136559	8398	Masp2	3.792119537	3.615298327	4.84022638110698	Polk	6.400132281	6.412356466	6.440087698	12998	Smim13	9.879511876	9.664326855	9.834915776	15298	Wdr18	7.146589154	7.075300654	7.364086643

1499	Apmap	7.808707308	7.952548719	7.940097967	3799	Cyhr1	9.31654	9.373400831	9.144806119	6099	Gm2897	2.385278374	2.186495204	2.79293925	8399	Mast1	6.94888644	6.769415665	6.60908392110699	Poll	5.131687804	5.798484432	5.62409379	12999	Smim14	10.15201668	10.1438857	10.15366439	15299	Wdr19	6.572334543	6.593240529	6.748597187
1500	Apoa1	6.419449283	3.632133599	5.369076402	3800	Cylc1	0.011140696	0.927247897	0.99114743	6100	Gm29766	5.282349097	4.691806378	4.437702157	8400	Mast2	7.834065967	7.64576034	7.78761118910700	Polm	3.978355335	4.325737234	4.305444778	13000	Smim15	9.406556253	9.16178612	9.142336102	15300	Wdr20	5.172372803	5.569186595	5.762430264
1501	Apoa1bp	9.077736092	9.19151115	9.05143232	3801	Cylc2	1.647996138	2.47554526	2.308075503	6101	Gm29811	3.056767425	2.179546889	2.985571021	8401	Mast3	8.145706431	8.028384335	7.96869376610701	Poln	1.085350165	0.815802803	3.895296133	13001	Smim18	6.208755484	6.284423998	6.045863901	15301	Wdr24	5.601488814	5.569843549	5.439480859
1502	Apoa2	5.686595899	2.704191973	5.473246742	3802	Cyld	9.422862023	9.647169398	9.568713429	6102	Gm2a	8.437683502	8.614630386	8.279145085	8402	Mast4	8.486552163	8.815532522	8.84114518110702	Polq	3.244643971	2.118528873	2.980063974	13002	Smim19	7.86133581	7.878003463	7.761354655	15302	Wdr25	4.263569357	5.010690042	4.679369255
1503	Apoa4	3.884639187	0.595183765	2.781541599	3803	Cym	1.035275339	0.927247378	0.991147368	6103	Gm3002	3.964952797	2.643757616	2.787965687	8403	Mastl	2.104815565	3.306307576	3.44123263610703	Polr1a	6.553551909	6.585043003	6.023595119	13003	Smim20	6.249120532	6.413644242	6.302386636	15303	Wdr26	10.09592955	10.07267671	10.18703928
1504	Apob	2.168014449	3.700142668	5.537554206	3804	Cyp11a1	3.391986238	3.963365318	3.151357531	6104	Gm30505	0.024621235	1.48870551	1.573376226	8404	Mat1a	3.275009171	2.758384726	3.78469813310704	Polr1b	4.397124468	3.825952626	4.150081241	13004	Smim22	5.197347394	5.357796633	2.551585496	15304	Wdr3	7.447986909	7.683964234	7.714821017
1505	Apobec1	3.772329728	2.813796174	3.88948358	3805	Cyp1a2	2.403102777	1.808714825	2.565559302	6105	Gm3086	3.778848978	4.020525223	3.88790527	8405	Mat2a	10.16803827	10.13808612	10.0338656110705	Polr1c	7.309231464	7.289043546	7.464091136	13005	Smim23	0.032485722	2.72033809	1.987808395	15305	Wdr31	4.601664061	5.323285283	4.226734747
1506	Apobec3	3.081073568	2.824914598	3.89043875	3806	Cyp1b1	4.07922996	4.007285932	4.737956366	6106	Gm31763	0.032555024	1.476214309	1.987797526	8406	Mat2b	8.815453853	8.887730645	8.98848845910706	Polr1d	8.33305284	8.305550149	8.337583199	13006	Smim3	6.588893392	6.782787929	6.518842393	15306	Wdr33	7.738873289	7.69515692	7.687249183
1507	Apobr	2.66516205	3.021600228	2.788005909	3807	Cyp20a1	3.653320806	3.669029345	2.979756207	6107	Gm3219	6.381659916	6.350019115	6.390045311	8407	Matk	7.779797866	7.846388653	7.17901550210707	Polr1e	5.955328237	5.542929665	5.066208796	13007	Smim4	6.851609503	7.644266231	7.024037792	15307	Wdr34	5.755423625	6.108587222	6.390652606
1508	Apoc1	5.283420489	3.068935661	4.146288295	3808	Cyp26b1	6.795335475	6.518318805	6.666066602	6108	Gm3230	4.723563743	4.643909175	5.474832349	8408	Matn2	6.726746696	7.326450283	6.87456164710708	Polr2a	7.868073203	7.797855293	7.940170985	13008	Smim5	2.875856291	3.334719283	1.561294194	15308	Wdr35	8.0807464	8.066175715	8.232769158
1509	Apoc3	5.611082586	4.004817164	6.189617503	3809	Cyp27a1	4.834803555	4.344312365	5.068543192	6109	Gm3258	2.394865156	2.438110076	2.567512526	8409	Matn4	6.693780116	6.808398533	6.63842759610709	Polr2b	9.102832337	9.102450603	9.134845707	13009	Smim7	8.600032462	8.745932735	8.659553446	15309	Wdr36	6.55577183	6.785450836	6.74874438
1510	Apoc4	3.667022058	2.065088136	3.680107873	3810	Cyp2a5	2.871423873	2.167092117	1.563088216	6110	Gm3317	1.673881562	2.434592389	2.302864316	8410	Matr3	11.60268452	11.55669028	11.6752139210710	Polr2c	9.546052029	9.356907028	9.596574992	13010	Smim8	7.157402674	7.441496785	7.246926085	15310	Wdr37	8.305005331	8.388363343	8.609509835
1511	Apod	11.04141588	11.02306386	11.19004023	3811	Cyp2b10	1.659305453	2.186393087	2.792929194	6111	Gm3383	3.247705547	2.387636138	3.98387194	8411	Mau2	7.687567403	7.799620839	7.68049270310711	Polr2d	5.253781109	5.800914968	5.708748212	13011	Smkr-ps	2.870558326	3.701105323	1.563435838	15311	Wdr38	8.531117792	8.445786514	8.422988563
1512	Apoe	14.17920426	14.0314978	13.87857148	3812	Cyp2c29	2.403943748	0.750595223	3.685598514	6112	Gm3414	6.28485579	5.74173843	5.934424827	8412	Mavs	4.836883694	4.96863784	5.02480982110712	Polr2e	9.411416301	9.581728856	9.410451996	13012	Smn1	7.679595532	7.600129675	7.43103499	15312	Wdr4	6.470711615	6.100817387	6.439861594
1513	Apol10b	0.052790655	1.447413751	2.307774496	3813	Cyp2c44	1.066518363	0.858881444	1.569343764	6113	Gm3417	1.072075233	2.200771233	2.571303432	8413	Max	8.572753661	8.598915094	8.79385781210713	Polr2f	8.057066492	7.724812211	7.898845515	13013	Smndc1	8.172699743	7.94022335	7.968588191	15313	Wdr41	6.937799151	6.88271774	7.024732596
1514	Apol8	1.670498086	2.869244868	2.303540277	3814	Cyp2c66	0.045256158	0.863145585	2.572455658	6114	Gm3435	6.1461888	6.065650716	6.373032379	8414	Maz	6.753785221	6.979588032	7.04649137510714	Polr2g	8.580235434	8.790737586	8.384996662	13014	Smo	5.12890593	5.260690269	5.472200997	15314	Wdr43	8.790910468	8.625588282	8.680449888
1515	Apol9b	1.045661301	0.904824001	1.573892324	3815	Cyp2d22	5.609375858	6.068421085	6.109618035	6115	Gm3448	3.533039207	3.432033906	3.302477485	8415	Mb21d1	3.408302271	3.279768087	3.98145118410715	Polr2h	6.616241556	6.330633684	6.301959876	13015	Smoc1	5.208360042	5.302796491	4.979414381	15315	Wdr44	6.649270996	6.698841785	6.666802193
1516	Apold1	5.032746825	4.202649062	4.679382472	3816	Cyp2d26	1.080506434	0.827048563	1.982944927	6116	Gm3488	2.640813477	1.437493918	1.98385728	8416	Mb21d2	6.923638594	6.577803105	6.98068368910716	Polr2i	8.220139241	8.247385726	8.154835997	13016	Smoc2	6.366291022	6.392310977	6.023320328	15316	Wdr45	6.420051093	6.328461826	6.406300064
1517	Apom	0.050209122	0.854865758	1.985209703	3817	Cyp2d34	0.005608334	0.940318714	0.992698372	6117	Gm3500	2.665073137	2.84508202	3.152314944	8417	Mbd1	6.12337522	5.96641052	6.1096089310717	Polr2j	7.686072533	7.344935988	7.422967915	13017	Smok4a	0.016596698	0.915563672	0.989761378	15317	Wdr45b	8.76697432	8.891258338	8.803400256
1518	Apon	1.032340788	0.933566573	0.991897432	3818	Cyp2d9	1.659248663	0.823362227	2.305798708	6118	Gm3558	3.662091985	3.754461708	3.788757266	8418	Mbd2	6.937353244	6.980281879	6.66618051410718	Polr2k	7.626275255	7.525361776	7.534721448	13018	Smox	7.403321031	7.53834138	7.292484956	15318	Wdr46	3.989612607	4.863054528	4.567026335
1519	Apoo	7.656071922	7.304730857	7.319770246	3819	Cyp2e1	3.984767811	2.056193049	3.980604272	6119	Gm35612	4.774016318	4.073601984	4.148771801	8419	Mbd3	8.06366991	8.160366166	7.93400334910719	Polr2l	9.349128805	9.17349811	9.043337876	13019	Smpd1	8.903952659	9.033203912	8.907759794	15319	Wdr47	8.82530375	8.797847562	8.828632333
1520	Apool	4.393751123	3.833982142	4.150980588	3820	Cyp2f2	3.078041314	1.776824443	3.441724	6120	Gm3604	3.235694538	1.808363023	3.153170876	8420	Mbd4	6.48806868	6.192776421	6.45609240110720	Polr2m	9.028967646	8.870379132	9.184641491	13020	Smpd2	5.172096498	5.728588744	5.227145484	15320	Wdr48	8.360952388	8.338211821	8.256097535
1521	Apoo-ps	2.068126556	0.823188072	3.156652977	3821	Cyp2j12	5.83666974	6.355063958	5.862413618	6121	Gm3636	3.081672475	3.303587752	4.071592784	8421	Mbd5	6.252859527	6.798037649	6.73550626510721	Polr3a	5.782794404	6.070233572	5.734273187	13021	Smpd3	8.50203443	8.353570232	8.491870883	15321	Wdr5	7.14467275	6.859484835	6.957377217
1522	Apopt1	7.221640586	7.452664389	7.70147289	3822	Cyp2j6	6.213512524	6.48032779	6.992191621	6122	Gm3696	1.101747708	2.663547859	1.979097415	8422	Mbd6	6.306658789	6.124386939	6.26593463710722	Polr3b	6.210022117	6.101824714	6.595646713	13022	Smpd4	5.540261916	5.821936177	5.83889471	15322	Wdr53	5.933199956	5.913566138	4.619813317
1523	App	12.50109682	12.50994849	12.67150899	3823	Cyp2j9	6.987088988	6.741351831	7.046277994	6123	Gm37013	0.005608334	0.940318714	0.992698372	8423	Mbip	4.614223049	5.196363153	5.53593722610723	Polr3c	3.519669042	3.188976697	2.565904203	13023	Smpd5	6.148238822	6.61247839	6.108767356	15323	Wdr54	5.509682778	5.616734973	5.83839594
1524	Appbp2	7.522294149	7.321386226	7.617044427	3824	Cyp2s1	2.886082372	3.173775607	1.55706176	6124	Gm3704	3.059039315	2.874095279	1.980922745	8424	Mbl2	1.644856495	0.863104391	1.98590059710724	Polr3d	6.984850448	6.521145228	6.95783804	13024	Smpdl3a	7.249189769	7.349397658	7.024272671	15324	Wdr55	5.60227205	5.325232525	5.53478468
1525	Appl1	10.24659613	10.06283942	10.31765839	3825	Cyp2u1	2.3765536	1.45051187	0.982594496	6125	Gm38426	2.895284177	3.66165439	1.553134549	8425	Mblac1	5.03023399	5.347318862	4.787441510725	Polr3e	6.126255103	6.062705329	5.885819012	13025	Smpdl3b	3.772776499	2.991175423	3.889375431	15325	Wdr59	6.123826117	6.162728141	6.284484918
1526	Appl2	7.900754901	7.92549859	7.991318403	3826	Cyp39a1	1.671475461	3.043287166	2.790861699	6126	Gm38431	7.867015972	7.59922697	7.616615397	8426	Mblac2	7.846081102	7.601699258	7.31968836110726	Polr3f	6.212056487	6.483127147	6.246332166	13026	Sms	8.900501463	8.820832183	8.889928172	15326	Wdr5b	4.244779973	4.7217553	3.980134028
1527	Aprt	8.056498934	7.832554632	7.601771064	3827	Cyp3a11	1.050284147	0.894770312	0.987304499	6127	Gm38437	0.013876736	0.921269847	1.991050736	8427	Mbnl1	9.265556269	9.156815567	9.47391894710727	Polr3g	5.50579439	5.871536553	5.188474232	13027	Smtn	4.076325357	3.6355381	2.974636435	15327	Wdr6	9.670708876	9.506954854	9.447593191
1528	Aptx	7.157147342	7.123163667	7.013189263	3828	Cyp3a13	1.078773362	2.191205532	1.983263931	6128	Gm38666	1.639941203	1.468596247	1.571019866	8428	Mbnl2	11.27219815	11.07726839	11.1869959410728	Polr3gl	6.573211134	6.758524979	6.666513066	13028	Smtnl2	3.53080702	3.163174121	4.071856881	15328	Wdr60	7.269484501	7.315868715	7.265597401
1529	Aqp1	5.574058887	6.961874413	5.021745636	3829	Cyp46a1	8.279279451	8.169633042	8.184686832	6129	Gm3893	3.233911341	2.657163019	2.789516846	8429	Mbnl3	3.516190861	3.338642144	1.97936634710729	Polr3h	10.43696819	10.45357653	10.26071953	13029	Smu1	8.646902229	8.701812706	8.741540297	15329	Wdr61	8.141363243	8.104104025	8.33330404
1530	Aqp11	6.488329101	6.42457098	6.18903093	3830	Cyp4a10	1.040695909	0.915564801	0.989761512	6130	Gm4013	2.062189491	1.440689661	0.981211188	8430	Mboat1	4.399636365	4.711470186	3.43478034910730	Polr3k	7.931836501	7.899093275	7.910810358	13030	Smug1	5.865051438	6.429546926	5.979589918	15330	Wdr62	1.073720376	2.198412203	1.984198984
1531	Aqp4	9.648810959	9.762450427	9.850358581	3831	Cyp4a12b	1.050292068	1.905562281	2.311187493	6131	Gm4070	5.722296906	5.593262452	5.652688727	8431	Mboat2	7.83084314	8.431360086	8.67382407610731	Polrmt	5.367473198	5.752325341	5.735793931	13031	Smurf1	6.949970476	6.574599489	6.761173149	15331	Wdr63	2.399969875	4.143287286	1.561015853
1532	Aqp8	2.376480737	1.450718598	1.568937054	3832	Cyp4a14	1.0838151	1.422244184	3.156451061	6132	Gm4262	1.656686226	2.461728159	1.566681654	8432	Mboat7	9.797941625	9.75711588	9.74656430710732	Pom121	7.573293175	7.844705273	7.734888485	13032	Smurf2	7.279759093	7.140557022	7.13971977	15332	Wdr64	0.013875062	1.507515853	1.991051067
1533	Aqp9	2.869935185	1.836746208	3.155319047	3833	Cyp4b1	0.024641479	1.4886724	0.987886795	6133	Gm4285	5.632026661	5.778179981	5.503858892	8433	Mbp	14.53574573	14.74277233	14.488014710733	Pomc	4.755185688	7.322380497	5.184307705	13033	Smyd1	4.157574515	2.357771007	3.14733083	15333	Wdr7	9.049188087	9.044897792	9.124699324
1534	Aqr	7.058626351	7.159753705	7.067274683	3834	Cyp4f13	3.679515921	5.223701775	3.78455922	6134	Gm4349	3.516317565	1.391253377	2.790418425	8434	Mbtd1	7.868425471	7.831891815	7.88690792610734	Pomgnt1	6.875418012	7.181523846	7.189948626	13034	Smyd2	7.547564291	7.546295953	7.447580745	15334	Wdr70	6.456768328	6.659637399	6.838576225
1535	Ar	8.411179566	8.26354936	8.718416286	3835	Cyp4f14	4.888317791	4.524259385	4.563361193	6135	Gm4432	1.058888434	0.875875216	0.985089334	8435	Mbtps1	8.225788765	8.369234056	8.36368710810735	Pomgnt2	7.31814895	7.290191395	7.423300346	13035	Smyd3	8.140599991	7.813333072	8.213841945	15335	Wdr72	2.077765004	3.590079396	1.563080792
1536	Araf	7.926311954	8.380363026	8.067550945	3836	Cyp4f15	6.006774753	5.352514057	5.88761065	6136	Gm4532	2.092220019	3.880590489	2.788205999	8436	Mbtps2	5.780613662	5.694241442	6.00313887710736	Pomk	6.147912769	6.441549918	6.337757059	13036	Smyd4	6.262831205	5.416032688	5.405347828	15336	Wdr73	7.495442761	7.316837635	6.991031816
1537	Arap1	5.438217068	5.825092834	5.439392128	3837	Cyp4f16	2.667016518	3.176109481	2.563973073	6137	Gm4724	5.605755394	5.695086099	5.68028534	8437	Mc3r	0.027290482	2.232680379	1.9886437710737	Pomp	11.16395003	10.99954695	10.8464578	13037	Smyd5	5.539643501	5.999771869	5.762177451	15337	Wdr74	6.873525605	6.928908797	6.579584013
1538	Arap2	10.66742989	10.6651159	10.85730183	3838	Cyp4f17	0.019308208	0.910069061	1.990022989	6138	Gm4737	4.255355031	4.419639372	4.735887085	8438	Mc4r	6.008628031	5.472635256	5.53364479110738	Pomt1	6.4188567	6.678552205	6.337699492	13038	Snai1	2.646529238	2.682690568	1.56531878	15338	Wdr75	7.437297575	7.193693123	7.372393016
1539	Arap3	4.535496314	4.603440836	4.149200351	3839	Cyp4v3	5.573719373	5.771305066	5.787747054	6139	Gm4787	1.064665638	1.457650342	2.988529334	8439	Mcam	5.96796884	6.610751483	6.28354157310739	Pomt2	4.892315589	5.196846837	5.709889082	13039	Snai2	2.079910964	1.393926289	2.98402997	15339	Wdr76	4.66321184	4.06707192	4.788420011
1540	Arc	7.958916586	7.727075763	8.530861454	3840	Cyp4x1	0.035103537	0.880631705	2.310066899	6140	Gm4788	1.653771922	0.838769644	2.793877741	8440	Mcat	5.538529202	5.677137639	5.65315416410740	Pon1	2.071434005	1.84657333	2.792277026	13040	Snap23	5.930245105	5.600362811	5.472375735	15340	Wdr77	7.045743565	6.800816319	6.862400853
1541	Arcn1	7.745409743	7.805143403	7.652398379	3841	Cyp4x1os	2.059556299	1.87316179	1.984859661	6141	Gm4792	0.021985921	0.904802191	1.989545265	8441	Mcc	5.214630121	5.621988947	5.56511260810741	Pon2	7.963250652	8.124582945	7.825293941	13041	Snap25	12.90020693	13.03189664	13.16035621	15341	Wdr78	6.058442308	6.459957298	5.760245292
1542	Areg	1.052515981	1.480244262	0.986730994	3842	Cyp51	8.723958224	8.60613868	8.609360438	6142	Gm4832	2.37404978	1.457591157	2.572442664	8442	Mccc1	6.434883813	6.232022256	5.91000558410742	Pon3	5.288925589	6.146603518	4.223028324	13042	Snap29	7.298062712	7.232367881	7.024276509	15342	Wdr8	6.63443106	6.223227711	6.748603698
1543	Arel1	8.275589471	8.594485515	8.503695959	3843	Cyp7b1	6.568347235	6.516945805	6.001794344	6143	Gm4890	2.376463464	2.475576551	2.30807949	8443	Mccc1os	3.068627565	2.145883936	2.30111437610743	Pop1	6.327581985	5.850834476	6.001634218	13043	Snap47	11.77180657	11.53383406	11.5663474	15343	Wdr81	6.080217683	6.027031564	6.247327509
1544	Arf1	10.89436699	10.92263402	10.93272755	3844	Cyr61	5.072507131	4.64700626	3.140839729	6144	Gm4925	3.059036699	2.173163925	2.568372862	8444	Mccc2	5.719894494	5.188827224	5.1886049110744	Pop4	7.428583515	7.393288639	7.503836055	13044	Snap91	10.10271641	9.946340062	10.03665193	15344	Wdr82	8.337704225	8.297411069	8.372394698
1545	Arf2	7.688216328	7.78662053	7.910937374	3845	Cys1	5.172201326	5.151091382	5.534937699	6145	Gm4944	5.605542642	5.886774514	5.887462849	8445	Mcee	8.455755056	8.583230332	8.51527560110745	Pop5	8.274924748	8.080142158	8.013221955	13045	Snapc1	7.980185346	7.617802502	7.831847016	15345	Wdr83	7.146435882	7.26616447	7.129752444
1546	Arf3	11.13517207	11.26161162	11.14740363	3846	Cysltr1	3.237961813	3.68308364	2.564809768	6146	Gm4951	1.06651048	3.075774844	0.983116656	8446	Mcf2	4.725179421	3.356769941	5.22972897410746	Pop7	6.873377372	6.73326177	6.666412977	13046	Snapc2	6.680851089	6.896067765	7.002951895	15346	Wdr83os	7.114280166	7.315806586	7.129689104
1547	Arf4	8.531989177	8.551800922	8.522957028	3847	Cysltr2	1.050286491	1.484414306	0.987303898	6147	Gm4961	1.043233299	1.497678189	1.574430671	8447	Mcf2l	8.716531	8.687493112	8.57994419210747	Popdc2	2.630764145	1.468532031	1.571012362	13047	Snapc3	5.635599018	5.818978437	5.887589575	15347	Wdr86	3.770278652	4.934874643	2.561057135
1548	Arf5	3.778103407	3.845633811	3.564557208	3848	Cystm1	4.329500544	4.424150436	4.839905054	6148	Gm4980	3.877053585	3.857911297	3.149011629	8448	Mcfd2	8.044974597	7.726005359	7.61638427410748	Popdc3	5.019476486	4.434633622	3.297273302	13048	Snapc4	5.400674434	5.118031648	5.736446887	15348	Wdr89	11.7149293	11.72960664	11.60613573

1549	Arf6	8.887651336	8.907206088	8.901899585	3849	Cyth1	7.241563595	7.6284176	7.227615338	6149	Gm5	2.394833905	2.668565571	3.307345668	8449	Mchr1	5.807496725	6.07485733	5.83830097410749	Por	8.162688273	8.141473918	8.38109151	13049	Snapc5	7.208420978	7.292868008	7.024375555	15349	Wdr90	4.155522676	3.144485079	4.070144562
1550	Arfgap1	7.938263854	8.215855043	7.856467296	3850	Cyth2	8.555853892	8.482006334	8.591145931	6150	Gm5065	1.628806248	0.904780645	0.988485216	8450	Mcidas	1.632749728	0.894772526	1.9886460210750	Porcn	7.650024825	7.537720146	7.397549475	13050	Snapin	8.944871656	9.128518124	9.0298588	15350	Wdr91	4.601353069	4.896893051	4.565456061
1551	Arfgap2	7.10294225	7.240634845	7.035133848	3851	Cyth3	6.794413306	7.081555425	6.734834638	6151	Gm5069	6.060424155	6.379071561	5.811455803	8451	Mcl1	8.309455143	8.162974269	8.40212019510751	Postn	3.387430369	3.324079254	1.558844913	13051	Snca	11.02062761	10.92428485	10.38505007	15351	Wdr92	6.520233366	5.896348746	6.423476722
1552	Arfgap3	6.033248022	6.272199621	5.734284685	3852	Cyth4	5.806254548	6.172442075	5.371304825	6152	Gm5083	2.890631515	3.771986971	3.303321891	8452	Mcm10	1.071976413	0.846569972	1.56817198910752	Pot1a	5.126742603	5.299075618	5.302162144	13052	Sncaip	4.930446506	3.988763356	4.624571358	15352	Wdr93	1.062723212	2.906717905	0.984098158
1553	Arfgef1	8.811864393	8.640996362	9.005262365	3853	Cytl1	2.650072841	1.839171225	1.980926822	6153	Gm5086	0.047782154	2.208804218	1.569346098	8453	Mcm2	5.359146426	4.787215029	5.15080393610753	Pot1b	2.872195354	3.698498441	2.303316559	13053	Sncb	11.04052103	10.95803439	11.1474314	15353	Wdr95	2.634308879	1.457694538	0.983601276
1554	Arfgef2	7.723712454	7.690534397	7.911140308	3854	Cyyr1	2.873608246	3.19710864	2.567122406	6154	Gm5088	5.887115969	6.092061477	5.502417484	8454	Mcm3	4.720080533	4.420504426	5.02662213410754	Pou2f1	7.767337046	7.832324792	7.98581908	13054	Sncg	4.995082747	5.752716099	5.981707879	15354	Wdsub1	5.287477938	5.26827721	5.338335915
1555	Arfgef3	8.22935121	7.89738856	8.129855347	3855	D030028A08Rik	5.323284282	5.372623227	5.33885315	6155	Gm5089	3.987019483	4.912915357	3.786403441	8455	Mcm3ap	7.43539839	7.027063145	7.00222864610755	Pou2f2	5.803247634	5.624537872	5.762483858	13055	Snd1	7.974237556	7.899942695	7.881010864	15355	Wdtc1	7.634128292	7.679447355	7.511429108
1556	Arfip1	7.739224514	7.926314	7.800135728	3856	D030045P18Rik	0.024682584	1.488605194	1.573364445	6156	Gm5113	6.301614267	5.818929211	5.708034337	8456	Mcm4	5.572108268	5.479615812	5.73549679910756	Pou3f1	9.008828165	9.217250317	9.408453628	13056	Sned1	0.011143244	0.927242201	0.991146754	15356	Wdyhv1	6.014085696	6.082082429	6.108877911
1557	Arfip2	8.501773784	8.397090499	8.350736789	3857	D030047H15Rik	5.718361523	5.117595767	4.885151704	6157	Gm5124	7.642648774	7.619213295	7.503378491	8457	Mcm5	3.408138757	3.747638746	4.15215559910757	Pou3f2	7.558361956	7.831190769	8.464374294	13057	Snf8	8.303826137	8.191452487	8.184610492	15357	Wee1	6.93985134	6.922557647	7.247038297
1558	Arfrp1	7.081021994	7.299467423	7.189528358	3858	D030056L22Rik	7.337718264	7.175915507	7.169571376	6158	Gm5126	3.525900286	2.405382545	3.44250244	8458	Mcm6	5.473139637	6.000460466	5.14857519110758	Pou3f3	9.178596641	9.152858435	9.232680415	13058	Snhg1	6.537033648	6.042340941	6.337744328	15358	Wee2	0.008383876	0.933569984	1.576874431
1559	Arg1	2.885974429	1.787641559	2.563652647	3859	D030068K23Rik	5.635397774	6.077000681	5.78778632	6159	Gm5127	0.008382678	0.933572806	0.991898172	8459	Mcm7	6.652474068	7.173289061	6.85029344710759	Pou3f4	5.407762103	5.531910272	5.787413873	13059	Snhg10	5.028298305	4.748089418	4.838784398	15359	Wfdc1	3.399457019	2.379635739	1.552794683
1560	Arg2	10.13841782	10.15213137	10.07407458	3860	D10Jhu81e	8.947400858	8.84934533	8.841103336	6160	Gm5136	2.640794055	2.69449279	1.567412578	8460	Mcm8	4.669702725	5.343020757	4.50171412610760	Pou4f1	3.711916404	5.840622947	6.597109076	13060	Snhg11	11.20862338	10.84778126	11.48333621	15360	Wfdc17	1.080597751	2.188617375	2.570009388
1561	Arglu1	10.21859723	9.956553743	9.944293539	3861	D10Wsu102e	7.400556419	7.356577391	7.274858345	6161	Gm5141	3.89643055	3.503007993	4.982796576	8461	Mcm9	4.479395654	4.468731421	5.33952650310761	Pou5f1	0.013879255	0.92126446	0.990437367	13061	Snhg12	7.383190848	7.212741261	7.056495611	15361	Wfdc18	4.163023493	4.378129843	4.151062385
1562	Arhgap1	8.00317582	7.709331244	7.564886979	3862	D11Wsu47e	4.538724202	4.754252192	4.503688595	6162	Gm5148	11.13024023	10.81139487	10.75144547	8462	Mcmbp	7.873697372	7.50151254	7.54970026310762	Pou5f2	2.060928119	2.200768068	0.981668971	13062	Snhg14	8.006108633	8.06094012	8.260854034	15362	Wfdc2	4.334093248	5.318489053	3.675202643
1563	Arhgap10	5.918635498	6.689921114	6.708795906	3863	D130017N08Rik	6.977398843	7.024587399	7.527359205	6163	Gm5176	3.879872182	2.806552462	3.681460119	8463	Mcmdc2	1.073825144	1.440704042	1.9841795210763	Pou6f1	7.731711994	7.943051705	8.041048883	13063	Snhg17	3.983700945	4.246356432	4.151570267	15363	Wfdc21	3.667128706	1.254914518	0.954538448
1564	Arhgap11a	4.770554926	4.232104605	4.228158802	3864	D130020L05Rik	5.630800959	5.600127472	5.472345943	6164	Gm5177	0.024680579	0.899637699	0.98787806	8464	Mcoln1	5.085384698	5.114991368	5.50399589410764	Pou6f2	3.794788227	5.255720111	5.19247389	13064	Snhg18	5.744066493	5.368547496	5.109198649	15364	Wfdc3	2.375302987	2.477929306	1.569324625
1565	Arhgap12	9.055136138	8.974131207	9.157427506	3865	D130040H23Rik	4.246876957	3.626631156	4.790421794	6165	Gm527	3.646989911	3.322818986	2.788161564	8465	Mcph1	6.695327918	6.695694953	7.35632110510765	Pp2d1	7.538960092	7.533175758	7.397863722	13065	Snhg20	6.060434214	6.330750977	6.08796385	15365	Wfikkn1	1.060830335	1.888265914	1.986620714
1566	Arhgap15	5.636001786	5.617007755	5.958098555	3866	D130043K22Rik	7.175579592	6.615713899	6.991585758	6166	Gm5294	0.005608334	0.940318714	0.992698372	8466	Mcrs1	7.694968226	7.834205459	7.75492729110766	Ppa1	8.795199943	8.992961547	8.951541849	13066	Snhg3	6.454749145	6.423757218	6.595244916	15366	Wfikkn2	3.542676023	4.92212028	3.981471893
1567	Arhgap17	4.335637086	4.211228443	4.56432451	3867	D15Ertd621e	7.814912939	7.68260317	7.754778449	6167	Gm5415	4.976601544	3.718497799	4.840098756	8467	Mctp1	8.151282498	8.110323145	7.97996854510767	Ppa2	8.717395349	8.732345132	8.831768149	13067	Snhg4	6.492389231	6.576313188	6.707831154	15367	Wfs1	7.433565901	6.445197329	6.747949133
1568	Arhgap18	6.124933615	6.382517831	6.776047781	3868	D16Ertd472e	7.974859682	7.76751107	7.825414396	6168	Gm5424	5.542751899	5.474452617	5.564354942	8468	Mctp2	2.663000782	0.744602218	3.44345199810768	Ppan	5.121873091	4.834320968	5.229061021	13068	Snhg5	6.170398866	6.583266715	6.265079168	15368	Whamm	6.415625184	6.1999305	5.83745623
1569	Arhgap19	2.646480459	2.45355842	1.982067595	3869	D17H6S53E	5.629357301	4.824089136	5.959677617	6169	Gm5464	2.384308531	1.856564718	1.566339414	8469	Mcts1	8.38918208	8.418714641	8.43528657310769	Ppap2a	8.052414738	8.10448583	8.213705233	13069	Snhg6	7.719133039	8.218117659	8.018803281	15369	Whrn	6.267323812	6.269911249	6.189965245
1570	Arhgap20	8.868819545	8.725127941	9.315566149	3870	D17H6S56E-5	4.660310062	4.60057713	4.065909619	6170	Gm5468	4.824550924	3.999778762	4.625722957	8470	Mcts2	6.03288724	5.836570563	6.00271347410770	Ppap2b	10.75037257	10.70959573	10.80255679	13070	Snhg7	5.175077977	6.244943794	5.226416851	15370	Whsc1	9.189200764	9.011733086	9.49581939
1571	Arhgap20os	4.463440705	3.83289477	4.626772503	3871	D17Wsu92e	10.70808041	10.77460395	10.76708739	6171	Gm5475	1.04565561	1.493109204	1.573893583	8471	Mcu	9.033926716	8.701730437	8.54968302110771	Ppap2c	4.670649061	5.405863855	4.369537691	13071	Snhg8	9.418094031	9.501870412	9.324254661	15371	Whsc1l1	9.124879127	9.076148615	9.106696937
1572	Arhgap21	9.636716942	9.405599976	9.378691296	3872	D19Bwg1357e	8.406712099	8.305203247	8.389426772	6172	Gm5512	5.249159439	5.577369375	5.407149715	8472	Mcur1	7.752579953	7.92245314	7.61651752510772	Ppapdc1a	2.894256986	3.953045861	3.566829181	13072	Snhg9	5.728682241	6.162279482	6.35587713	15372	Wibg	1.086903234	1.416534994	3.156072737
1573	Arhgap22	3.885820439	4.173960888	3.787347051	3873	D1Ertd622e	7.347108847	7.229012799	7.580325529	6173	Gm5512	6.269415304	6.023932729	6.423568827	8473	Mdc1	5.936166146	6.113163961	6.17106675610773	Ppapdc1b	5.285841503	5.236587357	4.979796673	13073	Snip1	5.865047538	6.36689346	6.228246484	15373	Wif1	2.093336155	1.796363634	4.234294471
1574	Arhgap23	8.932569944	9.155843714	9.117030525	3874	D1Pas1	1.064648953	2.707799634	1.985897499	6174	Gm5523	2.885457345	3.44755003	3.442545978	8474	Mdfi	1.064627705	2.211534339	0.98360492610774	Ppapdc2	7.821181516	7.564557341	7.623773854	13074	Snn	9.15999427	9.304122993	9.159906539	15374	Wipf1	5.701270174	6.546249876	6.228111317
1575	Arhgap24	5.132893448	5.477367853	5.981106347	3875	D230025D16Rik	6.603565744	6.450634244	6.851041759	6175	Gm5532	1.040693543	0.915569906	1.574996121	8475	Mdfic	4.784970161	5.95035358	4.6789640210775	Ppapdc3	6.101946205	6.40061851	6.473071545	13075	Snora16a	0.062621506	2.692453878	2.793622049	15375	Wipf2	9.832022298	9.809461056	9.876354203
1576	Arhgap25	5.083209033	6.145955737	5.108726554	3876	D2hgdh	3.770808633	4.265096836	2.295970666	6176	Gm5577	2.403275777	2.852824165	2.788987452	8476	Mdga1	7.662824339	7.167519831	7.05643594110776	Ppara	5.9353944	5.864403139	5.680363813	13076	Snora19	0.01660226	0.915552217	1.990524174	15376	Wipf3	11.89834167	11.98064118	11.58218777
1577	Arhgap26	8.384937108	8.690489002	8.803557632	3877	D2Wsu81e	7.533042796	7.889089954	7.946022628	6177	Gm5595	5.210951451	4.775010922	5.372646353	8477	Mdga2	8.548441979	8.428978456	8.88406169510777	Ppard	4.612624648	5.162462086	5.067421579	13077	Snora21	4.472617162	4.147524369	4.839579867	15377	Wipi1	6.271630378	6.555428673	6.565403315
1578	Arhgap27	5.292768227	5.596154687	5.471842747	3878	D330023K18Rik	5.601808784	5.358872147	5.534900096	6178	Gm5607	6.787761642	6.065567362	6.18966164	8478	Mdh1	11.58342256	11.56653141	11.5654079410778	Pparg	3.662333147	4.107384473	3.439039674	13078	Snora26	2.872954087	3.041614458	2.984057686	15378	Wipi2	8.167553632	8.071506232	7.850095454
1579	Arhgap28	0.060212899	2.892021513	0.980770338	3879	D330041H03Rik	6.308375331	6.427089989	6.624877353	6179	Gm561	8.887342526	8.754020319	8.673450278	8479	Mdh1b	2.876524094	3.793811766	3.30639954810779	Ppargc1a	8.832413215	8.694466841	9.124761214	13079	Snora28	1.650991577	1.870190949	2.794356249	15379	Wisp1	3.881054627	3.752944095	4.305337235
1580	Arhgap29	6.924695521	6.476248528	6.454966099	3880	D330050G23Rik	3.392032687	3.444451233	3.304056421	6180	Gm5617	5.759878649	7.418735363	5.811386054	8480	Mdh2	11.11053542	11.0767954	11.0865448610780	Ppargc1b	7.540437957	7.721659977	7.721605247	13080	Snora30	7.176975057	6.428076145	6.623077169	15380	Wiz	6.947860014	6.734843541	6.282923159
1581	Arhgap30	3.895851658	4.710703397	4.066419763	3881	D330050I16Rik	3.527668823	3.313281233	1.974403076	6181	Gm5640	1.040716832	0.91551964	0.98975616	8481	Mdk	7.969289481	7.9249045	8.00794016110781	Ppat	6.169375014	6.410224861	6.565662304	13081	Snora35	3.386894272	3.97219219	1.559108001	15381	Wls	7.349473494	7.769408049	8.114949817
1582	Arhgap31	6.246461383	5.903665483	5.786932925	3882	D3Ertd254e	8.119651137	8.104165323	8.279319345	6182	Gm5643	2.075797569	2.17100045	2.791419019	8482	Mdm1	3.655686854	3.76722487	1.97231694510782	Ppbp	2.637699852	1.44721845	2.794602066	13082	Snora47	2.382275866	1.434133307	2.570495131	15382	Wnk1	10.38785916	10.53577214	10.56965798
1583	Arhgap32	9.682433906	9.66572442	10.28167331	3883	D3Ertd751e	5.839197956	6.160355092	6.26576041	6183	Gm5643	8.275260612	8.427792126	8.251437891	8483	Mdm2	8.152180473	8.113336442	8.10918390510783	Ppcdc	4.946765357	5.074544028	5.595265045	13083	Snora61	1.649567375	1.872956106	2.794601835	15383	Wnk2	7.707158036	7.194156329	7.456004555
1584	Arhgap33	7.81250533	7.6354839	7.346272295	3884	D430019H16Rik	9.72015012	9.367195984	9.350655648	6184	Gm5662	0.011140597	0.927248116	1.991605264	8484	Mdm4	7.862052145	8.03395688	7.85030317810784	Ppcs	6.184757637	5.90565702	5.564020094	13084	Snora64	0.035072717	1.472424622	1.571467509	15384	Wnk3	8.146720188	8.141434959	8.129696954
1585	Arhgap35	8.852111366	8.773922473	9.062260348	3885	D430020J02Rik	2.870694302	2.441769933	2.567936699	6185	Gm5741	0.241066565	6.02189129	5.444912294	8485	Mdn1	8.203753486	7.919199656	8.0622016710785	Ppdpf	7.545960158	7.402428796	7.129559813	13085	Snora75	0.027312087	1.484364489	1.988640206	15385	Wnk4	3.404347472	4.257052722	3.888762567
1586	Arhgap36	1.078859759	2.461857309	2.793391494	3886	D430036J16Rik	3.758373161	2.420962	1.977411943	6186	Gm5766	2.869129284	2.44553603	2.304255224	8486	Mdp1	8.664335197	8.313333594	8.20867001110786	Ppef1	1.066610082	1.454048885	2.988266494	13086	Snora7a	2.871495835	1.399524427	2.791018871	15386	Wnt10a	4.599749771	4.486002747	3.88439373
1587	Arhgap39	7.522180666	7.461618252	7.23722215	3887	D430041D05Rik	9.617372343	9.447147063	9.374429533	6187	Gm5796	2.659470879	3.031996704	3.306111853	8487	Me1	8.332111902	8.164994485	7.97421914310787	Ppfia1	7.767161092	7.968914994	7.72136637	13087	Snora81	6.906189872	7.019201694	7.701100046	15387	Wnt16	2.381037418	1.864716489	1.567429671
1588	Arhgap42	4.478754487	5.208388848	4.623097896	3888	D430042O09Rik	5.829254732	5.67870856	5.736081747	6188	Gm5801	3.774040123	4.029690418	3.565642983	8488	Me2	5.809266447	6.1293471	5.91085517710788	Ppfia2	10.86182616	10.71275314	10.47577979	13088	Snord100	0.027299956	1.905544534	0.987299714	15388	Wnt2	5.394885628	4.635520954	4.886779594
1589	Arhgap44	7.945416032	8.100049889	8.16990202	3889	D4Ertd617e	2.375298399	0.858697695	2.308380478	6189	Gm5801	7.158243075	7.280834976	7.319910406	8489	Me3	6.538399534	6.650249229	6.3549413410789	Ppfia3	8.984253457	9.140445575	9.16502005	13089	Snord104	4.722976725	4.350338182	4.564584378	15389	Wnt2b	2.886594493	3.445599764	3.791630759
1590	Arhgap5	11.23288244	11.17028655	11.35449007	3890	D5Ertd579e	9.438339268	9.518941042	9.744938977	6190	Gm5803	0.029913572	0.889928087	0.986735026	8490	Mea1	9.143490361	8.925082634	8.89282548310790	Ppfia4	8.28907645	8.311429222	8.199286035	13090	Snord110	0.040237865	1.464861393	2.309371439	15390	Wnt3	5.363494991	4.959076504	5.265616968
1591	Arhgap6	5.743149844	4.782447315	5.338587129	3891	D630003M21Rik	1.67481903	1.82368573	2.302677285	6191	Gm5820	2.63770408	1.447205314	1.568529374	8491	Meaf6	7.767320569	7.785901142	7.65219114710791	Ppfibp1	7.30571702	6.53265049	6.454801484	13091	Snord116	2.649229147	1.410636998	2.791835518	15391	Wnt4	6.180405568	5.510401332	5.405889977
1592	Arhgap9	3.398017562	2.999377576	3.79017838	3892	D630013N20Rik	1.045668541	1.493084854	0.988488484	6192	Gm5936	0.024644798	1.909337165	1.5733717	8492	Mecom	3.780304723	4.017750745	4.3044342210792	Ppfibp2	6.10243055	6.383939932	6.189807427	13092	Snord116l1	4.873761324	4.164106607	3.886069412	15392	Wnt5a	5.834046308	6.014711212	5.680253858
1593	Arhgdia	8.752589361	8.824831244	8.718146289	3893	D630023F18Rik	5.946071927	4.841640542	4.623381793	6193	Gm608	7.680515202	7.751394557	7.714731987	8493	Mecp2	9.964570705	9.878177643	10.0964131310793	Pphln1	7.822679038	7.972471027	8.184825463	13093	Snord116l2	2.88552001	3.016860401	2.787381979	15393	Wnt5b	3.391099718	2.838533535	2.787215927
1594	Arhgdib	5.290482293	5.680573998	5.265171862	3894	D630024D03Rik	2.654968825	3.33862697	2.983884782	6194	Gm6182	5.838893373	6.250353032	6.265833011	8494	Mecr	5.533815294	5.123119726	4.62159304310794	Ppia	11.02598243	11.02274357	10.95367514	13094	Snord15a	0.086242519	2.443470608	2.984777124	15394	Wnt6	2.878650638	3.187684822	1.977635212
1595	Arhgdig	8.318267631	8.213341967	8.328891727	3895	D630033O11Rik	3.235127098	2.422485928	3.443963359	6195	Gm6225	2.051263697	1.468484318	0.985105104	8495	Med1	9.028907369	8.94598029	9.10674297610795	Ppib	8.600224296	8.801901094	8.534512663	13095	Snord15b	0.037703325	0.876075935	1.987007792	15395	Wnt7a	5.164245997	5.050932147	4.934177176
1596	Arhgef1	5.633330676	5.032427371	5.336899969	3896	D630039A03Rik	1.64166657	0.871595919	2.309364184	6196	Gm6251	8.419423761	8.298186951	8.002155694	8496	Med10	7.022662185	7.077833406	7.26593989610796	Ppic	3.887255581	4.171259214	4.151295905	13096	Snord16a	2.094857526	2.842380603	2.787692504	15396	Wnt7b	6.845238589	6.259542965	6.337165572
1597	Arhgef10	7.665846879	7.927368613	7.82561315	3897	D630041G03Rik	3.890114524	4.613823201	3.886304615	6197	Gm6297	2.405377309	3.325331497	3.305398476	8497	Med11	6.266216735	6.070205209	5.98017384410797	Ppid	8.493544711	8.255131787	8.324278006	13097	Snord17	0.024644047	1.909338253	1.98908668	15397	Wnt8b	2.869037638	2.445756382	1.564044495
1598	Arhgef10l	6.608334237	7.169958463	7.129814471	3898	D630045J12Rik	7.099770255	6.744929831	6.81305318	6198	Gm6402	6.651823788	6.791832022	6.991606786	8498	Med12	4.942603007	5.398785871	5.6821986710798	Ppie	4.781376445	4.639336281	5.190975635	13098	Snord22	5.259849034	6.031292405	6.150724058	15398	Wnt9a	4.72899686	5.466657435	5.815769193

1599	Arhgef11	6.082532128	6.395461698	6.20869112	3899	D6Wsu163e	6.724960766	6.681515694	6.934500751
1600	Arhgef12	10.03207154	10.20304864	10.28834756	3900	D730005E14Rik	0.024650652	0.899694383	1.573370576
1601	Arhgef15	5.438392461	5.482342139	5.565329856	3901	D7Ertd443e	5.317066156	4.477126532	4.564786283
1602	Arhgef16	2.054175433	1.885095207	1.986250478	3902	D830030K20Rik	3.070333804	2.648470366	3.152741834
1603	Arhgef17	9.328196749	9.340259807	9.251476473	3903	D830031N03Rik	5.887799162	6.051368077	6.046585569
1604	Arhgef18	7.229814283	7.411009938	7.265569034	3904	D8Ertd738e	8.498069339	8.460260663	8.333062884
1605	Arhgef19	5.885876295	5.744989219	5.502735636	3905	D8Ertd82e	7.226762284	6.849950207	6.438568548
1606	Arhgef2	8.665255527	8.739648704	8.75490689	3906	D930015E06Rik	4.976463255	4.295896385	4.301782663
1607	Arhgef25	9.850401191	10.15265985	9.759669304	3907	D930016D06Rik	5.834555492	5.249026347	5.734759398
1608	Arhgef26	7.899956656	7.713914759	7.701011086	3908	D930020B18Rik	1.636456127	1.476277932	1.987804088
1609	Arhgef28	7.784723246	7.338371389	6.968082014	3909	D930028M14Rik	2.364433145	0.894716499	1.988641036
1610	Arhgef3	8.162099877	7.950847438	7.957179154	3910	D930048N14Rik	2.642739148	2.690493168	2.306334184
1611	Arhgef33	3.058977307	2.87423275	2.791646937	3911	Daam1	8.855111357	8.936744697	8.515055369
1612	Arhgef39	3.050911529	1.864692207	0.980785205	3912	Daam2	7.596654688	7.890040836	7.781047247
1613	Arhgef4	8.935216482	9.033487408	8.831713494	3913	Dab1	9.573891761	9.30946236	9.288307299
1614	Arhgef40	7.035929965	7.248062711	6.933695513	3914	Dab2	6.270285794	6.639703389	6.439887132
1615	Arhgef5	4.073098147	4.020021125	3.787824778	3915	Dab2ip	9.644675456	9.592608763	9.805018627
1616	Arhgef6	6.486614043	6.248638552	6.550804336	3916	Dach1	6.388796288	6.915855604	7.424027056
1617	Arhgef7	7.986868438	7.936476375	8.035465221	3917	Dach2	3.648348757	3.568912449	3.304710392
1618	Arhgef9	11.38431721	11.2460552	11.38193684	3918	Dact1	5.805579876	5.448356545	5.652607262
1619	Arid1a	8.738224569	8.53453649	8.663052127	3919	Dact2	6.902417643	7.266332074	7.729200728
1620	Arid1b	9.274923305	9.125610595	9.02432622	3920	Dact3	8.91331605	9.040861206	9.075521576
1621	Arid2	7.696300037	7.904086603	8.099179121	3921	Dad1	9.70448334	9.919219763	9.834895528
1622	Arid3a	5.854778943	5.851309815	5.188841086	3922	Dag1	9.072168401	9.080886614	9.315495754
1623	Arid3b	4.073703194	3.933179014	3.981228102	3923	Dagla	8.304014917	8.354939536	8.232709937
1624	Arid4a	10.02895475	9.981040856	9.996776314	3924	Daglb	6.650232703	6.591973928	6.735110526
1625	Arid4b	10.8528476	10.66094844	10.72732053	3925	Dalir	3.083101765	3.765538395	3.302412897
1626	Arid5a	4.16566419	4.163987021	3.678468332	3926	Dalrd3	9.214448103	9.020311458	9.010657297
1627	Arid5b	8.570282762	7.992022889	7.979890342	3927	Dancr	4.17035485	4.710147387	4.06633929
1628	Arih1	8.767258749	8.777098459	8.960213049	3928	Dand5	5.960280617	5.817110558	6.025279222
1629	Arih2	7.841073626	7.501392612	7.862906494	3929	Dao	0.027306411	0.89471769	0.987298305
1630	Arl1	1.100423086	0.780016535	2.983864325	3930	Dap	5.468404648	4.960069509	5.596209902
1631	Arl11	1.077277634	1.434324237	2.570510788	3931	Dap3	8.572678921	8.818966928	8.583556725
1632	Arl13b	6.507468518	6.735624102	6.761786516	3932	Dapk1	7.588042472	7.473691615	7.813266578
1633	Arl14ep	7.664061477	7.278807287	7.447615737	3933	Dapk2	3.770389411	2.819269358	3.302301699
1634	Arl15	7.011753595	7.266924515	7.067445925	3934	Dapk3	4.472654223	5.182284483	4.68138657
1635	Arl16	8.136202706	8.428143199	8.119431925	3935	Dapl1	4.307209344	2.111694431	3.984045482
1636	Arl2	8.646571878	8.830526788	8.774510909	3936	Dapp1	5.285127934	5.202208935	4.837355671
1637	Arl2bp	8.777341453	8.66405636	8.877948825	3937	Dars	7.679819143	7.678236452	7.623918833
1638	Arl3	10.38201957	10.30432467	10.27458565	3938	Dars2	6.059829724	6.315448675	5.75989768
1639	Arl4a	8.563700044	8.514716102	8.406271774	3939	Daw1	2.07363199	2.875822221	2.304507373
1640	Arl4c	8.485839344	8.210627388	8.787554169	3940	Daxx	7.694314133	7.659409668	7.447453495
1641	Arl4d	4.936207167	3.887422482	4.563865694	3941	Dazap1	9.211928268	9.035128127	9.085935791
1642	Arl5a	8.818342247	8.683109487	8.754799782	3942	Dazap2	9.275266461	9.309745023	9.215979028
1643	Arl5b	6.899547934	6.091027767	6.991687933	3943	Dazl	4.888315424	5.167681654	4.67939238
1644	Arl5c	2.377679272	1.447312501	1.568541803	3944	Dbf4	3.972072265	2.823366542	3.567007172
1645	Arl6	7.012145585	7.223010283	7.431743049	3945	Dbi	12.2573509	12.18264908	11.98313092
1646	Arl6ip1	12.29091607	12.23763376	12.07437856	3946	Dbil5	2.452110761	5.861982274	3.979066955
1647	Arl6ip4	8.527560134	8.444753501	8.564999796	3947	Dbn1	8.603152967	8.594467232	8.310868938
1648	Arl6ip5	6.619953852	6.875245397	6.666537271	3948	Dbndd1	5.807726594	5.668467468	5.980704835

6199	Gm6498	1.65658652	3.062505892	1.566707602	8499	Med12l	7.463944176	7.31223844	7.58758202910799
6200	Gm6524	1.038047567	1.926102747	0.990438751	8500	Med13	8.881742417	8.959241382	9.16497368610800
6201	Gm6525	5.567168902	5.041697471	5.265578046	8501	Med13l	10.06470622	10.00559987	10.0488236610801
6202	Gm6537	3.248982377	3.552344525	3.983617365	8502	Med14	7.290469995	7.749377007	7.47202630310802
6203	Gm6548	8.265271062	8.476336228	8.154836227	8503	Med15	8.074504527	8.086626613	7.92241983510803
6204	Gm6568	1.655170672	3.064347376	2.793637486	8504	Med16	7.805345462	7.321799137	7.29283895710804
6205	Gm6578	2.383309772	0.83052236	1.9832222	8505	Med17	7.27905195	7.364739395	7.19929661810805
6206	Gm6623	4.163342059	4.440981571	4.374345683	8506	Med18	4.827901284	4.986197398	4.06578601110806
6207	Gm6642	1.638269068	2.223031144	0.985632196	8507	Med19	8.50220446	8.471396441	8.35063475310807
6208	Gm6644	6.602338384	6.774998443	6.65295983	8508	Med20	6.765250595	6.659530692	6.65259092710808
6209	Gm6654	10.61173528	10.36717304	10.21833772	8509	Med21	7.376034374	7.383216615	7.7285029810809
6210	Gm6682	3.646003503	2.416189641	2.98204396	8510	Med22	7.05654554	7.098264263	7.08888395510810
6211	Gm6710	3.890906229	4.087508814	4.441242865	8511	Med23	6.230104689	6.04341542	6.14973000910811
6212	Gm6756	0.027289111	1.905559808	1.988643996	8512	Med24	5.864287686	6.067052391	6.08862735210812
6213	Gm6760	0.045231007	3.617958048	1.569758254	8513	Med25	6.588689983	6.919756302	6.80056747710813
6214	Gm6787	2.645568622	3.213278859	2.305533888	8514	Med26	4.787353441	5.605669405	5.40722823910814
6215	Gm6793	4.475183596	4.215377592	4.735469956	8515	Med27	7.391286688	7.046512093	6.84985300410815
6216	Gm684	2.664489659	3.322861088	3.152438968	8516	Med28	9.761795158	9.882592977	9.71974995110816
6217	Gm694	3.401762957	3.544899431	3.148696745	8517	Med29	6.055997391	6.23762562	5.98020240410817
6218	Gm6981	1.638291489	1.472235859	0.985625352	8518	Med30	5.444979296	6.272017115	6.28481561410818
6219	Gm6994	1.65385592	2.195859554	2.570779816	8519	Med31	7.490210114	7.646505613	7.45560223710819
6220	Gm7102	2.082596131	3.194322669	2.983537126	8520	Med4	7.781692664	8.111094833	7.87475502710820
6221	Gm7120	2.865806423	2.6827491	1.982061141	8521	Med6	6.232130737	6.468634011	6.76222648310821
6222	Gm715	6.168890028	6.082122749	6.900428531	8522	Med7	6.873732877	6.462084512	6.89920147910822
6223	Gm7173	3.377337113	3.047681338	1.980671813	8523	Med8	7.146052376	7.325242132	7.17986336610823
6224	Gm7244	0.032524305	1.898371685	0.986181929	8524	Med9	7.060295401	7.628506089	7.19881639910824
6225	Gm7334	5.54055228	5.912402208	5.371537327	8525	Med9os	3.390085132	3.448407847	2.78752824410825
6226	Gm7367	6.650772078	6.885593135	6.850709554	8526	Medag	6.103613776	5.919582478	6.30230424610826
6227	Gm7457	3.652074566	3.168197093	3.791169599	8527	Mef2a	10.17862124	10.26948304	10.2526300410827
6228	Gm7694	3.874465523	3.953614173	2.561423519	8528	Mef2c	12.49843406	12.35169248	13.0115384110828
6229	Gm7854	2.397410208	3.038829623	1.979120279	8529	Mef2d	2.881174306	3.182964121	3.56945132610829
6230	Gm8096	1.040698481	0.91555925	1.990524819	8530	Meg3	13.15593136	12.75010327	13.4029648610830
6231	Gm8179	1.05027804	1.48443013	1.572874315	8531	Megf10	6.226066097	5.969315866	6.08915069710831
6232	Gm8234	6.074097471	5.508275572	5.624000906	8532	Megf11	8.145721492	7.887066541	8.13501485910832
6233	Gm8580	2.395730169	2.436339685	2.567307488	8533	Megf6	2.858627544	0.846281345	1.98449977610833
6234	Gm8615	5.96108289	5.971332672	5.934612012	8534	Megf8	6.887988665	7.154660996	7.07838102910834
6235	Gm867	10.30235925	10.62759742	10.50158582	8535	Megf9	8.402441266	8.228940091	8.67372036110835
6236	Gm8787	0.045326501	1.457647523	2.572447399	8536	Mei1	2.646498216	2.682754485	2.79249117210836
6237	Gm8801	4.658696958	4.547571609	4.982872614	8537	Mei4	3.755773638	1.818998047	3.15396893110837
6238	Gm884	0.011144214	0.927240035	1.576210158	8538	Meig1	4.886858824	5.642417356	4.8871270910838
6239	Gm8979	1.070182441	2.203493436	1.568556422	8539	Meis1	3.679124881	4.42752798	4.73678890910839
6240	Gm8989	2.076704528	3.046165573	2.791240896	8540	Meis2	9.616220008	9.72162051	10.1748552210840
6241	Gm8994	3.527636421	3.673578439	3.303936467	8541	Meis3	6.587878395	6.698712559	6.88766333210841
6242	Gm9079	2.403005131	3.186830729	3.443871028	8542	Melk	1.05465283	1.476255848	1.57191598310842
6243	Gm9159	1.062782949	1.461140317	2.309028772	8543	Memo1	5.886340741	6.112850126	5.56424252210843
6244	Gm9199	2.080715018	2.864637265	2.790454171	8544	Men1	7.771541283	7.091401338	7.29273649110844
6245	Gm960	1.054687176	1.898310688	1.987795204	8545	Meox1	1.089870171	2.67738455	3.57204591110845
6246	Gm973	6.505801786	6.623314813	6.826463482	8546	Mepce	6.766665103	6.720397044	6.80033300610846
6247	Gm9767	2.65331435	1.830162553	2.984257226	8547	Mertk	3.077679895	2.119592434	3.89109623410847
6248	Gm9776	4.314839927	3.536678729	3.680983924	8548	Mesdc1	7.429207733	7.344701478	7.57268383110848

Ppif	6.458503925	6.862040176	6.946182428	13099	Snord33	1.641678245	1.464756899	1.570571476
Ppig	11.63125304	11.39995274	11.47283353	13100	Snord35a	1.087068989	3.053828891	0.977739032
Ppih	5.631354475	4.865648569	5.709078478	13101	Snord35b	1.054645657	0.885225013	1.57191755
Ppil1	6.186356677	5.967218944	5.957250587	13102	Snord42b	1.63827429	1.472273771	1.987391562
Ppil2	7.472149783	7.415919996	7.372316935	13103	Snord45c	1.674935347	3.466580753	1.979125837
Ppil3	6.191102902	6.295710601	6.681191621	13104	Snord47	1.045645239	0.904858833	0.988494455
Ppil4	9.26010047	9.080881141	9.139875938	13105	Snord49a	0.032528807	0.885207277	2.310427021
Ppil6	4.773859746	4.757784234	4.301580177	13106	Snord49b	7.345104019	6.71506917	6.862253894
Ppip5k1	7.281018244	7.445799087	7.572721141	13107	Snord55	2.094033926	3.020436942	2.981446925
Ppip5k2	7.221566385	7.368423756	7.594947739	13108	Snord65	1.050303524	1.48438241	0.987299523
Ppm1a	9.791584212	10.05002909	10.0514881	13109	Snord68	3.657111608	2.615999075	4.30652938
Ppm1b	9.572420277	9.579310106	9.748243579	13110	Snord72	1.628802828	0.904789265	1.57388878
Ppm1d	6.587754796	6.673403313	6.638460956	13111	Snord83b	1.66936799	1.402246305	3.571544709
Ppm1e	9.316247904	9.340376417	8.931121001	13112	Snord87	6.172199635	6.566009068	7.089676472
Ppm1f	6.620993723	6.630005204	6.774443344	13113	Snord89	8.106255093	8.088922164	9.824228978
Ppm1g	8.319362469	8.559881621	8.435325161	13114	Snph	10.11233279	10.13186022	9.848781765
Ppm1h	6.893145473	7.638831119	7.905170261	13115	Snrk	7.970953938	8.233536768	8.511513507
Ppm1k	9.65049176	9.661093109	9.918182851	13116	Snrnp200	8.732012888	9.084511244	9.093879001
Ppm1l	9.604064716	9.558882477	9.736612214	13117	Snrnp25	7.587138719	7.475398547	7.283573373
Ppm1m	6.172420191	6.723182527	6.168737132	13118	Snrnp27	9.808057387	9.582713694	9.524929816
Ppme1	10.54411871	10.55975118	10.68218137	13119	Snrnp35	5.888298818	5.90353194	5.910698299
Ppox	3.394871614	3.006764183	2.97976467	13120	Snrnp40	7.167809868	7.403651346	7.179730335
Ppp1ca	9.431497231	9.609975286	9.42292513	13121	Snrnp48	8.250019693	8.123060606	8.067364544
Ppp1cb	9.678766978	9.749157867	9.88842303	13122	Snrnp70	8.996262791	8.8230944	8.904779429
Ppp1cc	9.002198356	8.951694021	9.070190015	13123	Snrpa	6.665552735	6.535788302	6.748349943
Ppp1r10	8.112674857	7.899000396	7.787258674	13124	Snrpa1	6.310740649	6.359833487	6.439425424
Ppp1r11	10.04628687	10.06560461	9.977155341	13125	Snrpb	8.423744544	8.146069265	8.194342731
Ppp1r12a	10.18090261	9.929210889	9.970096269	13126	Snrpb2	9.871384998	9.723703268	9.634606642
Ppp1r12b	7.010624774	6.957191505	6.812627173	13127	Snrpc	5.958408307	5.478277097	5.405546218
Ppp1r12c	8.208416213	8.1064089	7.831635798	13128	Snrpd1	9.113403193	8.903943245	8.837923393
Ppp1r13b	7.680444492	7.76956657	7.673469145	13129	Snrpd2	8.791090422	8.789621762	8.515108886
Ppp1r13l	4.511495904	3.176975882	2.564103263	13130	Snrpd3	10.22644588	10.11494558	10.21361238
Ppp1r14a	7.598079308	8.300469974	8.024490125	13131	Snrpe	8.285188367	8.163192137	8.350722868
Ppp1r14b	6.585509348	6.528605479	6.246585499	13132	Snrpf	7.251407574	7.279419469	7.414664477
Ppp1r14c	6.452632281	5.982896071	6.284041803	13133	Snrpg	7.919997019	7.951431448	7.951510737
Ppp1r15a	6.553783901	6.440255854	6.087861803	13134	Snrpn	11.99121892	11.97798439	11.95259886
Ppp1r15b	9.309357901	9.108471826	9.251422259	13135	Snta1	7.861120719	7.995440021	7.439011992
Ppp1r16a	6.901707631	7.017736695	7.311444119	13136	Sntb2	7.045527512	6.84744366	6.910648337
Ppp1r16b	9.724750601	9.898410216	10.07542822	13137	Sntg1	7.589415623	7.701255321	8.204507175
Ppp1r18	5.471581827	5.114797517	5.503973129	13138	Sntg2	2.662957947	3.972343073	3.792663084
Ppp1r1a	9.040580343	9.005042697	9.013386798	13139	Sntn	4.764387561	4.918362797	3.887331181
Ppp1r1b	9.559369164	10.53869928	10.5966037	13140	Snupn	6.437411776	6.098637665	6.354978259
Ppp1r1c	0.029961991	0.889839957	0.986724672	13141	Snurf	12.01131583	12.00521262	11.9768044
Ppp1r2	8.477793992	8.723909537	8.751545227	13142	Snw1	10.11392544	10.03742085	10.10023272
Ppp1r21	7.680238626	7.702695217	7.345857177	13143	Snx1	8.858279997	8.578310519	8.819246803
Ppp1r26	5.86536825	6.025042956	6.504526177	13144	Snx10	9.607434044	9.255441258	9.313140058
Ppp1r2-ps3	5.692447044	5.539479057	5.652960609	13145	Snx11	6.524049297	6.759958097	6.838558084
Ppp1r2-ps9	0.060059582	3.818570884	1.567437433	13146	Snx12	7.900188986	8.034882904	7.844232825
Ppp1r32	1.655241196	2.46402061	1.983555386	13147	Snx13	8.080391875	8.066844204	8.199324205
Ppp1r35	4.168297561	4.431588439	3.978951518	13148	Snx14	6.849852134	7.077727082	7.140167214

15399	Wrap53	5.034788477	5.338678464	4.886235357
15400	Wrb	8.033091161	7.856049249	7.541975408
15401	Wrn	6.752018409	6.478437981	7.003116054
15402	Wrnip1	7.438633535	7.710356325	7.728481971
15403	Wsb1	7.404335881	7.427115168	7.694215216
15404	Wsb2	9.754482957	9.643716677	9.748231829
15405	Wscd1	9.048738422	9.152126329	8.734773423
15406	Wscd2	7.219274359	7.085399222	6.862135956
15407	Wtap	7.710741736	8.073422379	7.754632487
15408	Wtip	5.571923602	5.9569675	5.708485617
15409	Wwc1	4.09016221	4.593898357	5.304816693
15410	Wwc2	8.501805261	8.333999483	8.431329199
15411	Wwox	6.584025466	6.282953727	6.355499799
15412	Wwp1	8.189607051	8.083920456	8.591263445
15413	Wwp2	6.553716883	6.542906734	6.472259523
15414	Wwtr1	7.375813309	7.496342052	7.439366557
15415	Xab2	5.443571341	6.111576021	5.734595675
15416	Xaf1	5.28760083	5.160440857	5.440550623
15417	Xbp1	8.388601574	8.064656193	8.306407356
15418	Xcr1	1.062772444	1.885110458	1.570151895
15419	Xdh	3.865730024	2.847580218	3.305239158
15420	Xiap	9.558471804	9.632212655	9.830248998
15421	Xirp2	0.047919898	1.454048849	3.158604047
15422	Xist	3.07780514	3.009697864	3.791153577
15423	Xk	6.848411699	6.838274743	7.078563892
15424	Xkr4	6.125895567	6.411995055	6.22797444
15425	Xkr6	5.694011895	5.592325782	6.025715747
15426	Xkr8	4.601595221	4.803349435	4.301126858
15427	Xkrx	0.045279353	0.863106479	2.795416485
15428	Xlr	2.665811029	3.680115642	2.56428722
15429	Xlr3a	3.966513962	2.405820436	2.787390395
15430	Xlr4a	3.225899426	1.410893401	0.976977548
15431	Xndc1	3.225217101	2.177480578	1.981483492
15432	Xntrpc	3.235025792	3.030602975	2.789191188
15433	Xpa	7.481832336	7.806507044	7.58738026
15434	Xpc	6.861352918	6.803703089	6.874968006
15435	Xpnpep1	6.231821198	6.66371757	6.108546347
15436	Xpnpep2	1.045662243	1.913266615	1.573892116
15437	Xpnpep3	5.437386511	5.678571996	5.472136153
15438	Xpo1	8.328505875	8.185418807	8.439517145
15439	Xpo4	2.659505583	2.655864505	2.982863476
15440	Xpo5	5.567151499	5.157943351	5.566219136
15441	Xpo6	6.887435112	6.574715227	6.898987846
15442	Xpo7	8.996188787	8.785546303	9.00241747
15443	Xpot	8.214522594	8.239778714	8.046002559
15444	Xpr1	9.250548994	9.175010694	9.380918648
15445	Xrcc1	5.540923454	5.772930814	5.761870223
15446	Xrcc2	5.073384414	4.4141897	4.371004329
15447	Xrcc3	4.889186555	5.405525781	5.373866127
15448	Xrcc4	6.189759484	6.229726376	6.439924369

1649	Arl6ip6	6.368818404	6.683999355	6.694342779
1650	Arl8a	8.029975355	8.439020114	8.443678723
1651	Arl8b	9.26540454	9.344027242	9.433351673
1652	Armc1	10.22391968	10.13343549	10.21480578
1653	Armc10	6.520854148	5.780236474	6.456197425
1654	Armc12	0.035065372	2.491210381	1.987408212
1655	Armc2	4.073098957	4.319584964	3.564287017
1656	Armc3	4.080916544	4.908626909	4.373556525
1657	Armc4	2.399933866	3.034623867	1.978379895
1658	Armc5	5.634770604	5.866805317	5.761858673
1659	Armc6	6.400937994	6.08174408	6.08801519
1660	Armc7	7.521572262	7.255890198	7.129531948
1661	Armc8	8.519162936	8.559552036	8.491759409
1662	Armc9	4.884186371	4.696592961	4.680463852
1663	Armcx1	7.290922976	7.53263915	7.602312352
1664	Armcx2	8.922235051	8.81865375	8.744890076
1665	Armcx3	8.723943187	8.551877888	8.542193821
1666	Armcx4	7.787277877	7.804993682	7.793929653
1667	Armcx5	7.919110876	7.75628536	7.934357588
1668	Armcx6	5.076494394	5.170219018	5.025161811
1669	Armt1	6.147698962	6.331661457	6.302089418
1670	Arnt	5.632660556	5.623619049	5.735813856
1671	Arnt2	9.292522221	9.231905211	9.650799836
1672	Arntl	6.418041078	6.348665273	6.208814527
1673	Arntl2	3.537758636	3.290914301	3.789057979
1674	Arpc1a	9.737005089	9.783381472	9.66294486
1675	Arpc1b	8.13433915	8.111904676	7.594370944
1676	Arpc2	11.60413686	11.64640504	11.46075473
1677	Arpc3	9.253007107	9.295354638	9.232634879
1678	Arpc4	9.498495995	9.865418209	9.555530781
1679	Arpc5	9.541764235	9.62490907	9.281418285
1680	Arpc5l	9.708156983	9.737163219	9.72481082
1681	Arpin	5.291267769	5.779470784	5.301645284
1682	Arpp19	10.29755223	10.61606296	10.95377477
1683	Arpp21	11.03687199	10.81491726	11.17908365
1684	Arr3	1.060802347	1.464815392	2.309367196
1685	Arrb1	9.342447344	9.219244434	9.352905788
1686	Arrb2	4.943454987	5.000278631	5.372831441
1687	Arrdc1	5.030916194	5.378608917	4.934725165
1688	Arrdc2	4.326759349	4.490559977	4.625546108
1689	Arrdc3	7.986662159	7.675398275	7.666262183
1690	Arrdc4	5.293040479	5.567918526	5.534738851
1691	Arrdc5	1.628785846	2.239658077	0.988491293
1692	Arsa	6.666625223	6.83873251	6.934499412
1693	Arsb	7.080790222	7.529926572	7.056698757
1694	Arsg	6.557469705	7.03356332	6.54953675
1695	Arsj	4.332796324	5.141731749	4.624148623
1696	Arsk	6.269329504	6.231294922	6.023925237
1697	Art3	3.10110189	4.500982758	4.790882266
1698	Artn	3.632707468	1.853976507	1.56599306

3949	Dbndd2	10.28646508	10.52898553	10.34957304
3950	Dbnl	7.808312471	7.994774962	7.800193947
3951	Dbp	8.361960595	8.484974779	8.598426881
3952	Dbpht2	8.273968673	8.018075481	8.199399713
3953	Dbr1	7.102525036	7.086265227	7.247133486
3954	Dbt	7.056680988	6.836505193	6.837799312
3955	Dbx2	7.218396916	6.934839832	6.957179683
3956	Dcaf10	7.751966074	7.45102694	7.579899093
3957	Dcaf11	7.499340697	7.851161694	7.728285336
3958	Dcaf12	7.998651862	8.004228278	7.825446431
3959	Dcaf12l1	8.992668885	8.789457331	8.641694444
3960	Dcaf12l2	4.39587136	4.666367959	4.228780181
3961	Dcaf13	8.079643865	7.636091279	7.666187006
3962	Dcaf15	5.080092018	5.235696709	5.338629435
3963	Dcaf17	2.091339565	3.683360797	2.788377575
3964	Dcaf4	7.454302701	7.314191764	7.535013307
3965	Dcaf5	9.09189468	9.234882945	9.182136664
3966	Dcaf6	9.355999899	9.217886776	9.124676187
3967	Dcaf7	8.99330587	8.911822032	8.803257758
3968	Dcaf8	9.747735505	9.768514787	9.787340516
3969	Dcakd	7.09103807	7.259487135	6.874550102
3970	Dcbld1	5.040149274	5.485047	5.439687913
3971	Dcbld2	7.136274614	6.953212875	7.768803813
3972	Dcc	7.410674662	7.469417876	7.337609524
3973	Dcdc2a	6.22969644	6.397045649	6.722830491
3974	Dcdc2b	4.082355736	4.431983424	4.066920128
3975	Dchs1	5.833220917	5.887740319	5.652350263
3976	Dck	5.781370501	6.168188597	6.110184858
3977	Dclk1	11.21757088	11.13403979	11.28205791
3978	Dclk2	8.085694909	8.174898848	8.035328069
3979	Dclk3	7.428262956	7.024007949	7.667098972
3980	Dclre1a	5.3636302	5.26558109	5.338004912
3981	Dclre1b	4.826160454	3.818061302	4.44008245
3982	Dclre1c	6.118163555	5.592723475	5.371495581
3983	Dcn	10.02377082	7.983173701	8.113981121
3984	Dcp1a	4.779213133	4.282113552	5.151799419
3985	Dcp1b	4.726597477	5.093478434	5.110322851
3986	Dcp2	8.397804139	8.176310432	8.56889426
3987	Dcps	6.708108696	6.634305005	6.721919586
3988	Dct	3.067860406	3.688386534	0.971701295
3989	Dctd	4.240036942	3.934687751	2.781825546
3990	Dctn1	10.00519609	9.81347217	9.895868315
3991	Dctn2	9.806670261	9.761222185	9.842662569
3992	Dctn3	9.474825395	9.30514327	9.359353645
3993	Dctn4	8.891072191	8.980518101	8.980059865
3994	Dctn5	8.112327499	7.932019221	7.637993071
3995	Dctn6	8.694848436	8.582745496	8.591027337
3996	Dctpp1	6.750087277	6.511322889	6.519302139
3997	Dcun1d1	9.633067651	9.59288275	9.65068809
3998	Dcun1d2	6.620065408	6.658036554	6.652402039

6249	Gm9833	7.657370917	7.599888509	7.557416748
6250	Gm9866	6.637278615	6.802511657	6.81275647
6251	Gm9899	4.988110464	5.126258439	5.596760069
6252	Gm9920	2.06339091	1.437589456	1.567416568
6253	Gm9926	2.04493061	1.484317249	0.987290586
6254	Gm9958	2.073595658	2.175423611	2.985192023
6255	Gm996	6.886381991	6.757391281	7.200139011
6256	Gm9962	4.473775323	4.355051464	4.839315102
6257	Gm9999	1.686229498	4.95001031	2.788390548
6258	Gmcl1	7.773843646	7.701635737	7.934436989
6259	Gmds	5.327121009	5.332358594	5.440156045
6260	Gmeb1	7.36596809	7.438645275	7.565261125
6261	Gmeb2	6.011850241	6.251541593	6.150182288
6262	Gmfb	10.15827893	9.942664409	9.495537821
6263	Gmfg	1.109637398	3.33003072	2.789208966
6264	Gmip	2.901576006	3.288730442	4.231273957
6265	Gmnc	4.230874623	3.434938011	1.554201766
6266	Gmnn	3.779191394	3.746584956	3.43804251
6267	Gmppa	5.724110129	6.05589993	5.438471215
6268	Gmppb	7.515152815	7.60714712	7.439307267
6269	Gmpr	8.524000442	8.835982764	8.764692699
6270	Gmpr2	5.858826743	5.21789108	5.935163626
6271	Gmps	6.901917554	6.955405007	7.170048295
6272	Gna11	9.566263622	9.490640449	9.441436592
6273	Gna13	8.852376116	9.002910018	8.913639274
6274	Gna14	2.383218745	1.859285556	1.983248504
6275	Gna15	2.650793062	3.204234183	2.304052722
6276	Gnai1	10.10694413	9.939633265	10.21127294
6277	Gnai2	11.00307378	10.94444478	10.81680699
6278	Gnai3	7.113730329	7.197044115	7.078010158
6279	Gnal	9.930840207	9.890116347	10.26547403
6280	Gnao1	12.83315926	12.79798406	12.70128892
6281	Gnaq	11.03828767	10.95814502	10.87479727
6282	Gnas	13.3286584	13.20339349	13.10699845
6283	Gnat2	0.021978371	1.493092271	1.989546589
6284	Gnaz	9.158858294	8.821269524	8.916748648
6285	Gnb1	12.0816554	12.08004034	12.19004732
6286	Gnb1l	2.690232564	4.251149032	3.887963441
6287	Gnb2	9.069526532	9.34631774	9.270052187
6288	Gnb2l1	10.33553005	10.32676707	10.20154854
6289	Gnb4	6.25522929	6.939737026	7.002979478
6290	Gnb5	8.947767182	8.972518612	9.007948162
6291	Gne	7.045126384	6.825286241	7.024632639
6292	Gng10	8.728012118	8.815629492	8.623761809
6293	Gng11	8.275086356	8.212807914	8.342050825
6294	Gng12	8.900048982	8.775047624	8.616561274
6295	Gng13	8.463321318	8.230149021	8.319925533
6296	Gng2	10.21600805	10.04857689	9.865628106
6297	Gng3	10.70335108	10.54869072	10.45570287
6298	Gng4	7.418710975	7.195584124	7.256244043

8549	Mesdc2	8.618892439	8.36115339	8.43123057310849
8550	Mesp1	1.630821256	2.236082024	0.98788351510850
8551	Mest	8.398209457	8.630121441	8.64903144610851
8552	Met	5.494646514	4.643022084	5.02570913710852
8553	Metap1	7.985597619	7.834159066	7.65948219910853
8554	Metap1d	5.206410206	4.577846681	5.33885172910854
8555	Metap2	10.05784278	10.0337537	10.0216319310855
8556	Metrn	8.235923708	8.577364389	8.30635306410856
8557	Metrnl	5.832862218	5.932572424	5.37130745510857
8558	Mettl1	4.244331769	4.440981948	4.06813762710858
8559	Mettl10	6.490330255	6.785006115	6.43919471110859
8560	Mettl13	5.031010104	5.055071934	3.88195893610860
8561	Mettl14	7.001001325	7.230518422	7.25620310610861
8562	Mettl15	4.407450454	4.413403985	5.19152658210862
8563	Mettl16	8.356381024	8.093708162	8.20873020210863
8564	Mettl17	5.604525325	5.447276692	5.59455953610864
8565	Mettl18	5.597790931	5.159313013	4.97919139210865
8566	Mettl2	6.07747281	6.239516355	6.00287916510866
8567	Mettl20	3.393427314	3.774640597	1.97402629110867
8568	Mettl21a	5.667992984	5.860497954	5.93445528810868
8569	Mettl22	6.229092972	6.366545814	6.28420912510869
8570	Mettl23	6.552704608	6.364447223	6.28394431210870
8571	Mettl24	3.23073991	3.040107319	3.15445131910871
8572	Mettl25	4.325290071	4.714612188	4.30257797510872
8573	Mettl3	6.16769682	5.684424672	5.88609812210873
8574	Mettl4	4.930540044	4.220682187	3.88383733910874
8575	Mettl5	8.074333811	7.897989144	7.83782186610875
8576	Mettl6	6.522131978	6.039524116	6.73570513810876
8577	Mettl7a1	6.951027327	6.812654797	6.99126823710877
8578	Mettl7a2	4.782988604	4.524881189	5.15092931810878
8579	Mettl7b	4.078133319	4.439798057	3.1451365910879
8580	Mettl8	6.079379654	6.028677523	6.19003929710880
8581	Mettl9	7.329765039	7.626925502	7.68065594110881
8582	Mex3a	5.721631257	5.869106813	5.56508951810882
8583	Mex3b	6.796173206	6.967328929	7.10957467210883
8584	Mex3c	7.53976118	7.524579679	7.49567307210884
8585	Mex3d	5.171199668	5.730230986	5.37231335810885
8586	Mfap1a	9.455561638	9.345274264	9.43325423410886
8587	Mfap1b	8.749261379	8.526799087	8.81612975110887
8588	Mfap2	4.871666815	4.722998383	2.78019629810888
8589	Mfap3	7.717255953	7.650760213	7.70104489410889
8590	Mfap3l	7.860961889	7.638176904	7.71473080910890
8591	Mfap5	2.055581565	1.45758078	0.98358536510891
8592	Mff	9.578457333	9.515247934	9.66651328610892
8593	Mfge8	10.32803505	10.18928676	9.8332408610893
8594	Mfhas1	9.085717308	8.95438779	9.09904708810894
8595	Mfi2	1.653865252	2.466205332	2.793861710895
8596	Mfn1	7.597801996	7.947041441	7.87480239210896
8597	Mfn2	8.944294603	8.9366638	8.85961819110897
8598	Mfng	2.691224232	3.531045443	3.14676974410898

Ppp1r36	3.537589073	4.328543474	2.977448557	13149
Ppp1r37	2.660275976	3.688303789	3.153334864	13150
Ppp1r3b	2.873567696	3.467927896	2.302895623	13151
Ppp1r3c	8.763156822	8.578910208	8.471697576	13152
Ppp1r3d	5.03660827	4.730429732	5.189601098	13153
Ppp1r3e	3.39164711	2.125644125	2.787047349	13154
Ppp1r3f	6.973664919	6.629610665	7.002824599	13155
Ppp1r3fos	3.067877235	3.329899613	2.789189929	13156
Ppp1r3g	5.599973634	5.456470388	5.108432592	13157
Ppp1r42	3.080764581	3.555634929	3.790562711	13158
Ppp1r7	9.539720547	9.400056002	9.467856289	13159
Ppp1r8	7.962343483	7.750222982	7.65927707	13160
Ppp1r9a	10.98525787	10.98810148	11.24855539	13161
Ppp1r9b	10.57343026	10.67876619	10.58361034	13162
Ppp2ca	10.45445873	10.38372134	10.44147447	13163
Ppp2cb	10.34365614	10.28303622	10.21600446	13164
Ppp2r1a	11.16997235	11.11162673	11.14615383	13165
Ppp2r1b	5.635446983	5.618102932	5.371259142	13166
Ppp2r2a	7.279098178	7.214096158	7.293165869	13167
Ppp2r2b	10.54690308	10.18404173	10.48980444	13168
Ppp2r2c	12.56463509	12.49956565	12.45621664	13169
Ppp2r2d	9.06351061	9.022589479	9.201665838	13170
Ppp2r3a	8.646667516	8.760256575	8.623810341	13171
Ppp2r3c	9.313722658	9.083207605	9.302016876	13172
Ppp2r3d	6.692798492	6.391310651	6.595063296	13173
Ppp2r4	9.592514518	9.868993368	9.721458023	13174
Ppp2r5a	8.265299926	8.203919387	8.381010956	13175
Ppp2r5b	8.583971623	8.606936498	8.507496189	13176
Ppp2r5c	10.06338521	9.942423688	9.934177769	13177
Ppp2r5d	8.962861376	8.843454768	8.774351228	13178
Ppp2r5e	9.35872981	9.085097474	9.400034224	13179
Ppp3ca	14.72593617	14.89116319	14.78349856	13180
Ppp3cb	10.34373956	10.28288277	10.34414168	13181
Ppp3cc	4.845411992	5.849063095	5.439482	13182
Ppp3r1	13.01906502	12.95247591	12.98612789	13183
Ppp3r2	1.647972071	3.073743492	2.571872155	13184
Ppp4c	4.549408481	5.00619838	5.710236814	13185
Ppp4r1	5.810140182	6.049959625	5.886719922	13186
Ppp4r1l-ps	3.244264487	3.561143337	3.891103869	13187
Ppp4r2	7.694871127	7.658353373	7.439134423	13188
Ppp4r4	7.479874277	7.42503826	7.034865759	13189
Ppp5c	8.760001083	8.711062419	8.806577655	13190
Ppp6c	5.986853253	5.880733797	5.934161853	13191
Ppp6r1	8.906970656	9.122267636	8.771085108	13192
Ppp6r2	4.009561871	4.876506163	5.710256758	13193
Ppp6r3	8.514814225	8.434158943	8.483873632	13194
Pprc1	5.162754672	4.27568042	5.11034656	13195
Ppt1	9.65994119	9.740343025	9.733216731	13196
Ppt2	5.506667963	5.481204207	5.565189524	13197
Pptc7	9.830532636	9.730647585	9.981423331	13198

Snx15	6.421848082	6.710932417	6.735280259	15449
Snx16	9.297011959	8.905348203	9.249156262	15450
Snx17	7.611606292	7.626525009	7.5724653	15451
Snx18	7.289939267	7.203103575	7.319903997	15452
Snx19	7.651322598	7.675602029	8.057100923	15453
Snx2	7.981261742	8.023397213	7.89280716	15454
Snx20	2.638756378	2.200764892	2.307458769	15455
Snx21	5.829333251	5.189200396	5.788696035	15456
Snx22	8.611987304	8.836271295	8.542158932	15457
Snx24	6.540914612	6.671388914	6.652453578	15458
Snx25	6.349198019	6.275725153	6.851101605	15459
Snx27	9.832090834	9.85053566	9.907794738	15460
Snx29	2.85955163	1.867375854	2.571041993	15461
Snx3	9.832110834	9.751966817	9.988469472	15462
Snx30	8.607166716	8.241988306	8.410472859	15463
Snx31	0.027321476	1.90551423	0.987295016	15464
Snx32	7.199862594	7.203520922	7.496095255	15465
Snx33	5.40424834	5.935885056	5.813856043	15466
Snx4	6.928355779	7.230278608	7.283877133	15467
Snx5	9.060616456	8.997550027	9.106738229	15468
Snx6	8.694667748	8.678053608	8.319654215	15469
Snx7	6.161648234	5.590838984	5.838557283	15470
Snx8	6.709151154	6.658527466	6.721664686	15471
Snx9	3.539480148	3.753648865	3.438994897	15472
Soat1	7.269387753	7.005814686	7.088420039	15473
Sobp	10.6557612	10.48017871	10.3561163	15474
Socs2	7.750751599	7.312522489	7.487931023	15475
Socs3	4.767428268	4.239379268	4.150694272	15476
Socs4	4.159713207	4.248922942	3.14659723	15477
Socs5	8.28081123	8.332997722	8.587455442	15478
Socs6	6.18447369	5.717997934	5.67998134	15479
Socs7	10.05335001	9.929288567	10.00653848	15480
Sod1	11.8765873	11.70306491	11.65768529	15481
Sod2	9.083272469	8.873148434	9.139915721	15482
Sod3	4.991954913	5.943183029	5.023418202	15483
Soga1	7.197695678	7.116160663	6.850101276	15484
Soga3	10.22129653	10.13126586	10.09501269	15485
Sohlh1	0.005608334	0.940318714	0.992698372	15486
Son	11.52036775	11.3504453	11.35880664	15487
Sorbs1	10.18901199	10.02492013	10.18334907	15488
Sorbs2	8.410891499	8.264081593	8.414757989	15489
Sorbs2os	1.083782771	2.184115625	2.305522956	15490
Sorbs3	5.074860932	5.210114163	4.623338061	15491
Sorcs1	6.084009072	6.392629556	7.079591418	15492
Sorcs2	6.189959219	5.850415732	6.30201097	15493
Sorcs3	8.641979184	8.325016436	8.439439043	15494
Sord	6.926265712	7.068436278	6.799844395	15495
Sorl1	9.272867693	9.09914345	9.397900099	15496
Sort1	10.16124067	10.06908118	9.974310861	15497
Sos1	8.108376111	8.066455141	8.098937523	15498

Xrcc5	6.737813003	6.591869849	6.735097368
Xrcc6	5.406786043	6.056075927	6.003324857
Xrcc6bp1	5.632332043	5.292907889	5.471959179
Xrn1	7.767766699	7.808466376	8.093956014
Xrn2	8.506825086	8.495969082	8.561232559
Xrra1	3.065260093	3.464164004	1.978637483
Xxylt1	3.789073432	4.159288208	4.840802886
Xylb	3.879292651	4.45730289	3.982519435
Xylt2	5.508363702	5.385873865	5.680652466
Yae1d1	6.231804184	6.361296097	6.595271617
Yaf2	9.804961466	9.7326373	9.723131225
Yap1	6.900723584	6.957700832	6.980150792
Yars	7.482164013	7.658680364	7.721627193
Yars2	6.8881131	7.269425291	6.825293613
Ybey	5.121128503	5.499459488	5.190625553
Ybx1	10.38893636	10.49439621	10.37657797
Ybx2	3.911346228	6.723426732	3.291474273
Ybx3	7.849901012	8.572922383	7.974210235
Ydjc	6.617805243	6.526196141	6.638707496
Yeats2	6.647111893	6.584358026	6.423179424
Yeats4	9.00206958	9.040766824	8.942921722
Yes1	5.443117998	5.412374615	6.046883925
Yif1a	10.61986753	11.07665658	10.56865304
Yif1b	6.926048153	7.088255024	7.099269003
Yipf1	8.365145944	8.330946436	8.104033782
Yipf2	8.14076562	7.982008376	7.974267624
Yipf3	7.761073925	8.038217948	7.97435613
Yipf4	7.88134091	7.915193745	8.01910037
Yipf5	8.72057581	8.751330237	8.57237903
Yipf6	7.103564355	7.035292427	7.077814831
Ykt6	8.854879648	8.8243722	8.63097951
Ylpm1	9.805083677	9.662334852	9.798562688
Yme1l1	9.245458361	9.367786686	9.211189553
Yod1	6.331109115	6.619924417	6.148764626
Ypel1	7.327006265	7.291335991	7.119522747
Ypel2	8.337520538	8.310093923	8.31545681
Ypel3	10.79745985	10.90393088	10.94216666
Ypel4	2.404722064	2.417875016	3.152883121
Ypel5	9.330748513	9.436510157	9.199108809
Yrdc	6.915497095	7.195730563	7.423364682
Ythdc1	9.851871473	9.667318007	9.692492042
Ythdc2	7.308196054	7.12286275	7.337872837
Ythdf1	8.151353224	8.232067439	8.169991137
Ythdf2	8.239980221	8.104339212	8.223250464
Ythdf3	8.897374071	8.904273517	8.954479713
Ywhab	12.70969609	12.59515862	12.53982879
Ywhae	12.85288892	12.67081969	12.6339349
Ywhag	12.23821417	12.16788252	12.04875038
Ywhah	11.96090517	12.00340496	11.84106173
Ywhaq	10.57832116	10.54789044	10.26879465

1699	Arv1	3.787406243	4.371471341	3.78575602	3999	Dcun1d3	6.470299667	6.298616698	6.456305919	6299	Gng5	8.501783778	8.412410064	8.114232987	8599	Mfsd1	8.005500712	7.95083453	7.98555563210899	Ppwd1	6.210047251	6.082470966	6.06698214	13199	Sos2	7.788473863	7.925600235	8.247178296	15499	Ywhaz	12.10917632	12.02911219	12.10151358
1700	Arvcf	7.277660513	7.107014961	6.7344132	4000	Dcun1d4	8.25034551	8.238879597	8.223165002	6300	Gng7	8.696355647	9.102343565	9.253878425	8600	Mfsd10	5.288268594	5.855976377	4.50043385610900	Pqbp1	8.993159606	8.752960199	8.940021575	13200	Sost	0.008383876	0.933569984	1.576874431	15500	Yy1	8.559857939	8.471122672	8.483860644
1701	Arx	6.398947066	5.80826375	6.33823523	4001	Dcun1d5	7.355902862	7.238603138	7.139664897	6301	Gng8	4.256779371	6.183386172	5.711986446	8601	Mfsd11	6.402560334	6.278025782	6.43949342710901	Pqlc1	7.454795574	7.263122375	7.208583248	13201	Sostdc1	6.987272319	8.151556739	5.704453759	15501	Yy2	2.874432204	1.823221644	3.306855845
1702	Arxes1	7.010323613	6.936523811	6.934262701	4002	Dcx	6.998302816	6.802535023	7.311755706	6302	Gngt2	5.025962952	4.59579953	3.977278473	8602	Mfsd2a	6.584498924	5.896238633	6.18934249910902	Pqlc2	4.322659305	4.437669245	3.78614311	13202	Sowaha	9.873074886	9.646606252	9.614758227	15502	Zadh2	6.402542938	6.376331492	6.534893596
1703	Arxes2	8.280631989	8.292504164	8.46790539	4003	Dcxr	5.172487008	5.596689477	5.471910515	6303	Gnl1	9.393135609	9.26413941	9.333174746	8603	Mfsd2b	1.043237876	1.497669542	0.98911102810903	Pqlc3	3.240073272	3.679282779	1.557833778	13203	Sowahb	5.668319389	5.585090006	6.441020417	15503	Zak	6.519602534	5.806479882	6.228095664
1704	As3mt	5.250187528	5.15738268	5.066780133	4004	Dda1	8.555991196	8.559516791	8.684068002	6304	Gnl2	9.080157264	9.057639784	8.856451365	8604	Mfsd3	5.282103474	4.692361174	4.8872887310904	Pradc1	5.573279997	6.077517259	5.761772863	13204	Sowahc	6.571288606	6.922882247	6.149059817	15504	Zan	1.040697588	1.502478969	0.989761082
1705	Asah1	6.016084099	6.437976766	6.609796538	4005	Ddah1	9.407059313	9.454430904	9.511400449	6305	Gnl3	8.219794499	8.001478825	8.104059192	8605	Mfsd4	7.993177691	8.067828204	7.96300827610905	Praf2	8.08575706	7.754371571	8.114546666	13205	Sox1	6.888117943	7.252305633	7.002536886	15505	Zap70	3.061246961	1.832096682	0.975355039
1706	Asah2	6.056049773	5.637972593	6.024717922	4006	Ddah2	6.983652256	6.854108015	6.207856492	6306	Gnl3l	10.26293065	9.954787037	10.34961281	8606	Mfsd5	7.695422293	7.911213023	7.84433378810906	Pramef8	5.601534059	5.293318002	5.472009003	13206	Sox10	8.429022204	8.688697297	8.292802952	15506	Zar1	0.035123819	1.472348449	1.987399246
1707	Asap1	9.194367129	9.127432901	9.469964554	4007	Ddb1	9.660028064	9.743199694	9.79057319	6307	Gnmt	3.975386895	3.546115866	1.970747797	8607	Mfsd6	9.054940949	9.144378862	9.17235748810907	Pramel4	0.027278537	1.905574701	0.987304391	13207	Sox11	9.270083539	9.056598015	9.054073178	15507	Zar1l	1.624497429	1.502485148	0.989761915
1708	Asap2	7.428564989	7.353929528	7.511672268	4008	Ddb2	5.630954502	5.572115964	5.14914332	6308	Gnpat	7.208712423	7.27549548	7.140003103	8608	Mfsd7b	5.295685382	5.644565142	5.76166274110908	Prc1	3.892258566	4.610223217	3.561998773	13208	Sox12	5.604367256	5.619071581	5.56474589	15508	Zbbx	1.638229767	1.894947001	0.985644192
1709	Asap3	4.070343356	3.538064397	3.14774636	4009	Ddc	6.188472212	6.31745619	6.189588298	6309	Gnpda1	6.125265329	6.317121249	6.247039441	8609	Mfsd7c	2.892720783	3.86499366	3.3028662410909	Prcc	5.601650236	5.391165606	5.406150523	13209	Sox13	4.981930897	4.697016688	4.680516378	15509	Zbed3	5.248162367	5.36923263	5.024050635
1710	Asb1	7.664919856	7.665509504	7.594738707	4010	Ddhd1	7.759883058	7.768953522	7.838107137	6310	Gnpda2	7.678437165	7.337370811	7.447732084	8610	Mfsd8	6.012286039	6.398976422	6.20913697310910	Prcp	4.333396007	4.536392481	4.624009779	13210	Sox14	2.127124285	2.971924927	4.375168452	15510	Zbed4	6.489943705	6.539215864	6.549989904
1711	Asb10	2.052685427	0.871741888	0.98460742	4011	Ddhd2	8.94421666	8.641225036	8.803380154	6311	Gnpnat1	6.124411476	5.94308454	6.228336383	8611	Mfsd9	4.330497024	4.755540181	4.6246809510911	Prdm1	3.230060675	3.041612024	2.567304959	13211	Sox15	8.531585953	8.633256628	8.431152237	15511	Zbed5	5.959275784	5.415794016	5.47115469
1712	Asb11	3.244224067	3.168451434	3.150964101	4012	Ddi2	5.072897426	4.935755389	4.788082219	6312	Gnptab	7.635526599	7.6314942	7.963321769	8612	Mga	9.368297894	9.272026323	9.39784669310912	Prdm10	3.070963389	3.572909982	2.564813699	13212	Sox17	3.67620797	3.509577141	4.937376968	15512	Zbed6	7.081042779	6.985219224	7.056637357
1713	Asb13	7.301122088	7.35953814	7.735200771	4013	Ddias	1.646473733	1.878959557	1.569331808	6313	Gnptg	7.974911518	8.068370014	7.844122044	8613	Mgat1	7.318973243	7.377730782	7.55779520510913	Prdm11	3.059041584	1.407844232	2.791629215	13213	Sox18	2.696134643	3.635463819	4.229607842	15513	Zbtb1	5.444121117	5.585448172	6.303206128
1714	Asb14	1.072045319	0.846413672	0.981677341	4014	Ddit3	8.81843317	8.912902249	8.771166116	6314	Gns	8.476649536	8.594481715	8.40630948	8614	Mgat2	7.11477597	7.264706839	7.50402252310914	Prdm12	0.011141891	1.512815409	0.991147113	13214	Sox2	8.845337146	8.993615687	8.910791277	15514	Zbtb10	5.939485829	6.106734895	6.024409493
1715	Asb17	0.02727081	1.905585586	1.988647016	4015	Ddit4	9.005486935	8.764142725	8.711076439	6315	Golga1	7.539423424	7.399428181	7.708289568	8615	Mgat3	7.987989622	8.109351641	8.25627355510915	Prdm15	5.074900998	4.530750612	4.299806722	13215	Sox21	7.259679741	7.222496129	7.43991093	15515	Zbtb11	9.250246461	9.017120938	9.142372156
1716	Asb17os	1.050271401	1.905596518	1.572875774	4016	Ddit4l	7.299957815	7.385401428	7.857288563	6316	Golga2	9.040151535	8.86002931	8.982861681	8616	Mgat4a	7.29990528	7.193603851	7.23722169510916	Prdm16	6.885547523	6.658538535	6.534346184	13216	Sox2ot	7.701332933	7.552786014	7.587459083	15516	Zbtb11os1	4.830169187	4.894209535	3.883484243
1717	Asb2	2.886040635	2.838781724	2.980880083	4017	Ddn	11.56281032	11.4288552	11.19487501	6317	Golga3	7.428819833	7.461155162	7.503778326	8617	Mgat4b	7.875031949	7.957452509	7.63786921210917	Prdm2	9.39987086	9.159420503	9.503686378	13217	Sox3	4.310106529	4.115587311	2.97836103	15517	Zbtb12	5.470325866	5.681114628	5.53541696
1718	Asb3	6.524178912	6.807447806	6.188379556	4018	Ddo	4.255894469	4.418596788	4.565104134	6318	Golga4	9.891138053	9.79464553	9.876310832	8618	Mgat4c	7.840876596	7.960259432	7.34587971910918	Prdm4	5.478802248	5.881947728	5.934313398	13218	Sox30	2.052696956	3.082069436	0.984603374	15518	Zbtb14	5.663798837	5.184559026	5.981399286
1719	Asb4	3.973516804	3.764584565	2.296112995	4019	Ddost	7.657758157	7.817235984	7.673549988	6319	Golga5	6.810442198	7.206556627	7.265802857	8619	Mgat5	5.472844678	5.596126477	5.9357063110919	Prdm5	3.551305292	4.555499646	3.678401471	13219	Sox4	7.911342544	7.678542895	7.587378338	15519	Zbtb16	8.176795963	7.915628021	7.700931486
1720	Asb5	2.383278946	1.859135388	1.566683334	4020	Ddr1	8.604047382	8.932674929	8.426934711	6320	Golga7	8.097753393	8.476691969	8.002153497	8620	Mgat5b	6.038032271	6.041170705	6.66736536110920	Prdm6	2.652519321	2.440084946	3.154984769	13220	Sox5	9.135215688	9.112685777	8.963019427	15520	Zbtb17	7.481160287	7.503959647	7.414575031
1721	Asb6	5.753344375	5.502647842	6.003050214	4021	Ddr2	6.471058255	6.156559018	6.439776511	6321	Golga7b	8.880999646	8.801137669	8.874909434	8621	Mgea5	10.15866531	10.09278662	10.2088302810921	Prdm8	8.400666047	8.223536927	7.4630979	13221	Sox6	7.687076812	7.639724949	7.55742168	15521	Zbtb18	11.72347091	11.78633335	11.39569846
1722	Asb7	6.710757111	6.826924277	6.899054923	4022	Ddrgk1	8.356450431	8.229614895	8.283751448	6322	Golgb1	10.18768913	9.97574478	10.1845767	8622	Mgl2	3.228765411	1.832072346	1.56305929210922	Prdm9	2.87506201	2.861749284	2.983546241	13222	Sox7	1.660649877	2.455450193	1.982336852	15522	Zbtb2	4.996877725	5.774003783	5.762004285
1723	Asb8	8.230013943	8.150612648	8.270115742	4023	Ddt	7.894676145	8.018262371	8.029914847	6323	Golim4	7.68909559	7.802531353	7.996894213	8623	Mgll	10.84617284	10.90619272	10.7711541610923	Prdx1	10.49321423	10.6315295	10.52203658	13223	Sox8	7.32898415	7.400049545	7.283569184	15523	Zbtb20	9.008253211	9.151563502	8.65218243
1724	Ascc1	6.46986337	6.457978042	5.88593695	4024	Ddx1	8.919955013	8.831363193	8.977156601	6324	Golm1	8.023145019	8.261785351	7.556798197	8624	Mgme1	5.85766492	5.387810392	5.68088547410924	Prdx2	9.886185079	9.807930796	9.666428733	13224	Sox9	10.15215442	10.57149036	10.22314866	15524	Zbtb21	6.538239991	6.209409003	6.519520551
1725	Ascc2	3.520659936	3.458304259	2.789026663	4025	Ddx10	8.906479769	8.676886066	8.721467492	6325	Golph3	7.279814779	7.347296816	7.077751108	8625	Mgmt	1.676063429	2.431166379	2.56670891210925	Prdx3	7.555629506	7.407126689	7.624102958	13225	Sp1	8.250968764	8.45683154	8.346284055	15525	Zbtb22	6.951786465	7.284008843	7.150085993
1726	Ascc3	7.355845232	7.130941364	7.218354697	4026	Ddx11	0.050380577	1.875965563	2.308071304	6326	Golph3l	6.58877805	6.734472596	6.875345439	8626	Mgp	8.618905051	8.034669975	8.47591196810926	Prdx4	5.166709609	5.198767008	5.109355849	13226	Sp110	2.640812584	1.864538364	2.306878431	15526	Zbtb24	4.984518644	5.014170661	5.151260634
1727	Ascl1	5.539731093	5.822951237	5.188150615	4027	Ddx17	9.868335144	9.681424227	9.704465954	6327	Golt1a	2.039547645	0.910063943	0.989110013	8627	Mgrn1	9.506915215	9.612821566	9.59839060710927	Prdx5	9.424935088	9.543754682	9.449620158	13227	Sp140	1.661909906	2.181873117	3.572524335	15527	Zbtb25	5.281441607	4.586930427	4.437971436
1728	Ascl4	1.058836439	2.220005841	1.57100481	4028	Ddx18	7.992345775	7.826973257	7.616382807	6328	Golt1b	6.459689884	7.098505552	7.024659398	8628	Mgst1	5.864346415	5.275651536	6.35586831810928	Prdx6	10.45782555	10.71701769	10.25720737	13228	Sp2	5.986227059	6.494803498	5.862536938	15528	Zbtb26	5.472539454	5.452549094	5.595205424
1729	Asf1a	7.673206187	7.798802456	7.774493991	4029	Ddx19a	6.309312492	6.409856838	6.24661495	6329	Gon4l	7.88085694	7.969088471	7.541909028	8629	Mgst3	7.430085847	7.208516213	7.86305871710929	Prdx6b	1.661818743	2.45360161	2.569310613	13229	Sp3	8.269719973	7.991542939	8.134821271	15529	Zbtb3	3.526752047	3.564859863	2.298832598
1730	Asf1b	2.401549065	3.689442825	2.789366252	4030	Ddx19b	6.836309348	6.789615714	7.302605823	6330	Gopc	7.788712776	8.018450394	8.05693718	8630	Mhrt	3.063945594	1.389281338	3.30687068810930	Preb	7.538711419	7.313133072	7.337523227	13230	Sp3os	7.113232921	7.067368546	7.024389012	15530	Zbtb33	7.327604877	7.418078285	7.045776844
1731	Asgr1	5.931417912	4.253799781	5.503890417	4031	Ddx20	4.723327889	4.413474706	4.564493887	6331	Gorab	6.793256487	6.771087973	6.825919786	8631	Mia	4.987400377	5.993778226	4.78647949710931	Prelid1	8.611472705	8.590110048	8.359253904	13231	Sp4	8.225414605	8.190332268	8.49983788	15531	Zbtb34	4.173007539	4.755989309	4.065659463
1732	Ash1l	11.1426445	11.06528191	11.28206516	4032	Ddx21	8.591872597	8.620194203	8.380784184	6332	Gorasp1	7.886594267	7.769845713	7.631008872	8632	Mia3	9.546209445	9.52936304	9.72487674210932	Prelid2	2.393032741	2.671968283	2.7912357	13232	Sp5	1.665705162	3.206965746	1.981199452	15532	Zbtb37	3.538269967	3.852570053	3.439307701
1733	Ash2l	7.679612537	7.495538071	7.463686964	4033	Ddx23	4.67326898	5.303365734	5.654395087	6333	Gorasp2	8.005878161	8.123309796	8.06207649	8633	Miat	6.726587954	6.284649814	7.2092545410933	Prelp	5.605754756	6.310892322	5.707859784	13233	Sp6	1.064694889	2.480472689	2.572443083	15533	Zbtb38	8.893866227	8.71421192	8.803401693
1734	Asic1	6.384752758	6.453118659	6.319561291	4034	Ddx24	11.51669158	11.35199569	11.41461456	6334	Gosr1	5.410599134	6.182477218	6.338576147	8634	Mib1	3.419773089	4.432283383	5.07109833310934	Prep	4.547033942	4.925283625	5.024794712	13234	Sp8	2.39823335	3.037457755	1.978879009	15534	Zbtb39	4.074643958	3.275039029	4.684283516
1735	Asic2	8.812077527	8.964984203	8.865671843	4035	Ddx25	8.463989611	8.202729133	8.495779672	6335	Gosr2	7.780810782	8.005288761	7.780930715	8635	Mib2	9.088361129	8.854600796	8.85342351310935	Prepl	9.442846608	9.406920408	9.904911499	13235	Sp9	4.714940341	4.431673492	4.066878189	15535	Zbtb4	9.81529904	9.748971786	9.950134081
1736	Asic3	1.641595232	0.871784569	0.984612392	4036	Ddx26b	5.087867807	5.891303393	5.680915566	6336	Got1	10.38550571	10.26484981	10.54987679	8636	Mical1	4.328452392	4.545866337	3.56068068810936	Prex1	8.011394723	7.748025075	8.179822234	13236	Spa17	6.149756841	7.303701086	6.471959903	15536	Zbtb40	4.397307205	3.11175144	4.790168378
1737	Asic4	4.26691984	4.629103928	5.266078517	4037	Ddx27	7.715781189	7.311368583	7.673750896	6337	Got1l1	2.65725649	3.334839196	2.566512216	8637	Mical2	6.080503675	5.943170152	6.3205191410937	Prex2	9.409114592	9.272135719	9.372313071	13237	Spaca3	0.040152166	3.730041443	0.984618548	15537	Zbtb41	7.464072609	7.496793408	7.66683675
1738	Asl	4.331212621	4.703674788	3.676066981	4038	Ddx28	6.571399134	6.496500406	6.406080016	6338	Got2	10.26786403	10.28918012	10.10536198	8638	Mical3	7.198057621	6.955860599	7.44018174510938	Prg4	7.09200273	6.975205209	7.550574609	13238	Spaca6	5.206114704	4.048655741	5.190279969	15538	Zbtb43	7.024948764	7.401778588	7.389576107
1739	Asna1	7.944883221	8.08198832	8.046160545	4039	Ddx31	4.161373208	4.245606671	3.787177846	6339	Gp1ba	2.640802759	1.864565019	0.980764388	8639	Micalcl	3.400604372	2.376231446	3.6822105310939	Prickle1	8.861736544	8.901785246	8.889942298	13239	Spag1	4.40305544	4.541989274	4.681509803	15539	Zbtb44	8.450966551	8.411722902	8.443570496
1740	Asns	8.265322682	8.070067956	8.301977095	4040	Ddx39	5.985142554	5.315707955	5.787274579	6340	Gp1bb	11.95092665	11.98601315	11.9729253	8640	Micall1	7.84895237	8.224546255	7.98009664510940	Prickle2	10.20064234	10.18681688	9.901766093	13240	Spag16	3.078615931	4.407673006	2.562514465	15540	Zbtb45	5.880911927	5.152930485	5.108215145
1741	Asnsd1	7.488008952	7.418021106	7.129586139	4041	Ddx39b	9.094675085	9.289737309	9.032492699	6341	Gp5	2.898640045	4.031892498	2.783964273	8641	Micall2	1.06078272	1.46485183	1.98662976410941	Prickle3	3.661104538	3.653028898	3.301128561	13241	Spag17	4.170848187	5.361352185	3.97828118	15541	Zbtb46	3.517945552	3.334768977	2.566501204
1742	Aspa	6.953508599	7.623337494	7.372443489	4042	Ddx3x	10.722696	10.7883357	10.96015124	6342	Gp9	0.074637841	1.419155189	1.56529646	8642	Micu1	9.022660414	8.89865655	8.96588943410942	Prim1	5.165113853	4.736010335	5.303128423	13242	Spag4	1.073727204	2.198402424	1.984197716	15542	Zbtb48	3.784922983	4.309383009	2.779740664
1743	Aspdh	3.387394063	3.453872023	1.558862746	4043	Ddx3y	9.477168202	9.370755206	9.622102973	6343	Gpaa1	7.887531454	7.774048642	7.70776269	8643	Micu2	7.16579034	7.059618553	7.09934007710943	Prim2	5.466757744	4.733214675	5.840630746	13243	Spag5	4.265238472	5.272283172	5.190181097	15543	Zbtb49	1.088323332	3.208691793	1.564696392
1744	Aspg	3.065199518	3.980200047	2.302240295	4044	Ddx4	4.310262381	4.636677847	2.295727737	6344	Gpalpp1	7.564988706	7.745557488	7.687372221	8644	Micu3	9.985963217	9.957966223	10.162433810944	Prima1	2.882411853	3.970388078	2.564654819	13244	Spag6	4.832764996	5.673414799	4.370831921	15544	Zbtb5	5.439715849	5.647872661	5.534527297
1745	Asph	9.604111207	9.65821298	9.579890109	4045	Ddx41	6.900288006	7.146227877	6.825424897	6345	Gpam	2.399185014	1.818847433	2.302186994	8645	Mid1	2.09399895	3.569878472	3.44291648610945	Primpol	4.243133141	3.402807329	3.887012389	13245	Spag6l	0.045275444	1.882102937	1.98590133	15545	Zbtb6	7.61803439	7.438751807	7.595066915
1746	Asphd1	7.208221657	6.966559365	7.140122385	4046	Ddx42	9.422538613	9.349478295	9.485851017	6346	Gpank1	7.239648986	7.299536641	7.542919037	8646	Mid1ip1	9.52151533	9.632472079	9.63465127610946	Prkaa1	6.727214289	7.170494925	7.189699062	13246	Spag7	8.222962137	7.762548127	7.968921015	15546	Zbtb7a	10.28734053	10.08214544	10.10669105
1747	Asphd2	8.90693739	8.957356847	8.738128978	4047	Ddx46	8.928870389	8.765292495	8.812868997	6347	Gpatch1	7.114006783	7.22303551	7.139898769	8647	Mid2	6.900997963	6.957172662	7.00249633510947	Prkaa2	7.523472027	7.44426402	7.819618581	13247	Spag8	3.654097412	3.667433593	3.567348815	15547	Zbtb7b	5.08431291	5.39476361	5.301870317
1748	Aspm	3.660129421	3.543861657	3.148552482	4048	Ddx47	7.849394814	8.143074528	8.083395312	6348	Gpatch11	8.650031135	8.510065136	8.48376341	8648	Midn	9.551926036	9.400082055	9.38512024210948	Prkab1	4.726193313	5.27790224	5.303766204	13248	Spag9	10.43495819	10.41872647	10.76711228	15548	Zbtb7c	3.541095314	3.847305745	4.569098063

1749	Aspn	3.979593758	3.415681307	3.888370846	4049	Ddx49	7.112853821	6.9136969	7.275173877	6349	Gpatch2	6.47434957	6.256826786	6.708109896	8649	Mief1	6.60168718	6.059882697	6.53500105510949
1750	Asprv1	2.378788254	2.200935429	0.981699341	4050	Ddx5	11.12093155	11.31667889	11.17103278	6350	Gpatch2l	5.604375052	5.820204823	5.53426134	8650	Mief2	5.430347282	4.580235862	4.62246101210950
1751	Aspscr1	4.249208529	5.115090037	3.978943691	4051	Ddx50	8.724110484	8.440213128	8.455571336	6351	Gpatch3	4.523343242	2.359705784	4.375827897	8651	Mien1	9.552269358	9.602815404	9.5460283910951
1752	Asrgl1	9.838809222	9.750412064	9.891390643	4052	Ddx51	5.250583753	5.602364643	5.227795835	6352	Gpatch4	8.519032619	8.471092957	8.47587901	8652	Mier1	9.127626331	9.088053684	9.02159359110952
1753	Ass1	1.048038268	1.488629505	1.5733673	4053	Ddx52	7.881618569	7.802539929	8.124892531	6353	Gpatch8	8.375115024	8.549988534	8.315334312	8653	Mier2	6.70728052	6.452718717	6.48789297410953
1754	Aste1	2.701170004	4.307289857	4.228849606	4054	Ddx54	6.998423996	7.303560719	7.302721472	6354	Gpbp1	9.722573415	9.618928824	9.741553519	8654	Mier3	7.964098216	8.182336446	8.31106144710954
1755	Astn1	8.51063311	8.296436031	8.568841817	4055	Ddx55	7.279704452	7.239141083	7.159807631	6355	Gpbp1l1	7.861882236	7.909827115	8.024586931	8655	Mif	10.07337683	9.964478825	9.84412118910955
1756	Astn2	5.914940455	6.319886853	6.046115416	4056	Ddx56	6.92372673	6.644761933	6.721575622	6356	Gpc1	8.428397472	8.537233265	8.189415923	8656	Mif4gd	6.364903053	6.557847601	6.04542690310956
1757	Asun	6.059060222	6.103309868	6.456513329	4057	Ddx58	5.032601292	4.78794755	4.563391502	6357	Gpc2	4.325510324	4.551511489	2.972488629	8657	Miip	1.691488567	3.175816499	1.97541391510957
1758	Asxl1	7.522486111	7.32102478	7.46395471	4058	Ddx59	5.501688478	5.043406839	5.40716414	6358	Gpc3	4.977147581	4.29426831	4.372307479	8658	Milr1	1.650885317	2.470943227	1.9845381210958
1759	Asxl2	7.54903294	7.508160384	7.707923879	4059	Ddx6	10.46976363	10.50035429	10.68650528	6359	Gpc4	4.53145693	4.005216904	4.067453199	8659	Mina	6.122828297	5.902723725	5.98007528210959
1760	Asxl3	5.988310986	6.143549701	6.002185578	4060	Ddx60	2.069327155	2.183981868	1.565637203	6360	Gpc5	7.327330286	7.045474546	7.109157049	8660	Mink1	8.318201925	8.239514089	8.1347964610960
1761	Atad1	9.676953192	9.826596798	9.853449956	4061	Deaf1	6.924039606	7.062940267	6.787534309	6361	Gpc6	4.670596609	4.579530986	5.303280648	8661	Minos1	9.483386397	9.40201802	9.32646532610961
1762	Atad2	6.034295876	5.879309924	6.424111384	4062	Dear1	4.328740507	4.152257479	4.736574496	6362	Gpcpd1	7.875127141	7.876479321	8.062322491	8662	Minpp1	6.08142512	6.196311353	6.28412970910962
1763	Atad2b	7.724215048	7.515358206	7.609228568	4063	Deb1	8.31370191	8.285587464	8.154862796	6363	Gpd1	8.199491115	8.330732623	8.109174493	8663	Mios	7.268989408	7.016707907	7.3291537710963
1764	Atad3a	6.454299589	6.469924071	6.087814622	4064	Decr1	6.385502312	6.421314863	6.694706854	6364	Gpd1l	8.579919641	8.388166973	8.447592031	8664	Mip	2.871463447	2.670175335	2.5677218710964
1765	Atad3aos	3.265765117	4.441096182	3.679153901	4065	Decr2	8.641448789	8.330018432	8.184453334	6365	Gpd2	10.7607066	10.61523251	10.39251996	8665	Mipep	6.586297428	6.541280479	6.12901529110965
1766	Atad5	5.541835801	5.865162029	5.887583408	4066	Dedd	7.69529838	7.822094067	7.601897863	6366	Gper1	3.403825569	1.738292941	3.565428249	8666	Mipol1	4.729378407	4.632034036	5.47358085810966
1767	Atat1	7.078735586	7.164437781	7.002644071	4067	Dedd2	5.287222852	5.268787894	5.265981307	6367	Gpha2	7.992981908	8.220069637	7.940036057	8667	Mis12	6.860531652	6.590568943	6.76146423210967
1768	Atcay	8.429616206	8.513752449	8.67708446	4068	Def6	2.668459913	3.015570985	3.568449159	6368	Gphn	8.871520149	8.621400893	8.889940085	8668	Mis18a	6.100239014	5.470399116	5.70731818210968
1769	Atcayos	1.062773603	0.867252223	1.986252038	4069	Def8	7.419930903	7.622513128	7.346301822	6369	Gpi1	10.48017694	10.42021599	10.22551562	8669	Mis18bp1	3.763101909	2.638533778	3.56856520310969
1770	Ate1	10.17971383	9.991272537	10.04203397	4070	Defb1	2.887279986	1.783530858	3.304052965	6370	Gpkow	7.9131289	7.737542889	8.035492273	8670	Mitd1	6.291425466	6.116692322	6.22739307410970
1771	Atf1	5.211977873	4.866468846	5.535255553	4071	Defb11	4.536218137	6.113601865	2.286534761	6371	Gpld1	7.19047142	6.971596514	7.471976152	8671	Mitf	6.708801986	7.01491711	6.35442004210971
1772	Atf2	9.506606369	9.454593228	9.561264144	4072	Defb30	1.050283708	2.232696876	0.987304612	6372	Gpm6a	13.26963988	13.22039138	13.155056	8672	Mki67	4.658771858	4.657970371	3.88480403710972
1773	Atf4	9.955488135	9.772578148	9.932731362	4073	Degs1	8.890759595	9.009075855	8.662900786	6373	Gpm6b	11.52718016	11.5771381	11.53935105	8673	Mkks	8.527950268	8.51666631	8.78747393710973
1774	Atf5	6.99960601	7.366261605	6.786852737	4074	Degs2	7.932569697	7.935402268	8.184781711	6374	Gpn1	6.421690316	6.68591103	6.863353929	8674	Mkl1	7.497303177	7.547869612	6.96800382110974
1775	Atf6	8.411552957	8.532010102	8.376539385	4075	Dek	10.30079877	10.12771379	10.18943968	6375	Gpn2	5.207182041	5.662562625	4.786227554	8675	Mkl2	9.472932348	9.555705107	9.6436082410975
1776	Atf6b	8.022026886	7.947616366	7.645087639	4076	Dennd1a	7.547927238	7.640230152	7.406086621	6376	Gpn3	5.214075966	5.32504429	5.958821865	8676	Mkln1	8.016611538	7.854197766	7.86868994310976
1777	Atf7	7.080944902	7.205212721	7.256337586	4077	Dennd1b	6.525638952	6.630185593	6.721398782	6377	Gpnmb	2.901603061	3.755023346	3.888812239	8677	Mkln1os	3.404332947	4.392376032	4.07011593510977
1778	Atf7ip	7.403653662	7.612911678	7.768143894	4078	Dennd1c	2.043186456	0.899686199	1.573369746	6378	Gpr1	3.380661351	1.39130275	3.444978023	8678	Mknk1	6.61485588	6.024489497	5.95665188310978
1779	Atg10	6.555147457	6.375589794	6.652919034	4079	Dennd2a	6.400094354	6.063850267	5.811719561	6379	Gpr101	6.215656296	4.195265676	4.979487671	8679	Mknk2	6.489932763	6.481538188	6.58012275910979
1780	Atg101	8.091694562	8.137603032	8.155002697	4080	Dennd2c	1.056741451	1.89494046	0.985643163	6380	Gpr107	7.00869685	6.885135676	6.438753204	8680	Mkrn1	8.662025724	8.780551245	8.8065794610980
1781	Atg12	9.328294971	9.283017138	9.080554683	4081	Dennd2d	7.385772393	7.45066107	7.602023283	6381	Gpr108	6.522579102	6.595825353	6.264937056	8681	Mkrn2	7.603970875	7.523684253	7.60207053610981
1782	Atg13	7.794534281	7.549934934	7.928589409	4082	Dennd3	2.872181158	3.199773643	1.56278336	6382	Gpr12	6.973901073	6.731389064	6.812894289	8682	Mkrn2os	3.534495824	3.66001953	3.78981400310982
1783	Atg14	7.199700248	7.314749669	7.319997246	4083	Dennd4a	7.556802587	7.276381513	7.850635561	6383	Gpr135	3.782870341	3.836074018	4.441951086	8683	Mkrn3	3.524906951	3.319390109	2.78766331710983
1784	Atg16l1	7.035612845	6.878153182	7.159922202	4084	Dennd4b	6.510134655	6.667436082	7.190094919	6384	Gpr137	8.152408836	8.362316492	8.179593275	8684	Mkx	4.783061518	4.340411317	5.62647612210984
1785	Atg16l2	5.20872474	5.461865356	5.440535956	4085	Dennd4c	4.66920178	5.205700135	5.151026082	6385	Gpr137b	4.258707567	4.843488871	4.935281649	8685	Mlc1	9.525514357	9.538430871	9.27679394910985
1786	Atg2a	6.144551098	6.20055087	6.17038918	4086	Dennd5a	9.490022989	9.508421476	9.600238273	6386	Gpr137b-ps	4.260920061	4.930027397	4.838196518	8686	Mlec	8.763365973	8.537237902	8.87189399310986
1787	Atg2b	7.628126485	7.827360822	7.922730329	4087	Dennd5b	9.017170838	8.984927436	9.072784016	6387	Gpr137c	4.336188229	4.931876387	5.025594757	8687	Mlf1	4.330914342	5.680068664	3.67615647410987
1788	Atg3	7.489943284	7.343820847	7.609523842	4088	Dennd6a	6.077885858	5.790081143	5.679805964	6388	Gpr146	6.457696256	6.806051533	6.748356218	8688	Mlf2	11.45394556	11.42796901	11.2595749810988
1789	Atg4a	2.865063017	1.422006494	2.569507495	4089	Dennd6b	5.441315383	5.384618359	5.981015935	6389	Gpr149	1.641666077	0.871597223	1.986622801	8689	Mlh1	4.476430567	5.350433745	5.02586272210989
1790	Atg4b	8.091563849	7.918841425	8.189599488	4090	Denr	9.727942835	9.737359623	9.675210034	6390	Gpr150	5.073619265	5.175901378	4.838729293	8690	Mlh3	7.635092968	7.77609096	7.70802192110990
1791	Atg4c	6.925368775	6.802864407	7.120084877	4091	Depdc5	6.473283568	6.566768366	6.519159514	6391	Gpr151	2.078679621	3.985401911	3.571262677	8691	Mlip	6.617434563	6.39234887	6.30166446110991
1792	Atg4d	6.833530091	6.746833357	6.680546837	4092	Depdc7	4.661107698	4.149207786	4.439509356	6392	Gpr153	4.951740164	5.103786671	6.266918481	8692	Mllt1	6.721258602	6.623366833	6.45584608810992
1793	Atg5	8.046300017	8.032547551	7.904641505	4093	Deptor	8.086124148	8.142202382	8.402393501	6393	Gpr155	7.603505879	7.672646759	7.580011654	8693	Mllt10	9.052128963	9.083487474	9.08328628910993
1794	Atg7	7.39090265	7.047238149	7.346846159	4094	Dera	3.661504105	3.942877318	1.550543591	6394	Gpr156	1.668118786	3.047595799	2.984796076	8694	Mllt11	11.14249799	11.01466223	10.8687065310994
1795	Atg9a	7.298103939	7.27518933	7.119606466	4095	Derl1	8.328097813	8.168215796	8.134699145	6395	Gpr157	4.080656397	4.55451837	3.678248073	8695	Mllt3	9.265047731	9.109316657	9.08849999410995
1796	Atg9b	4.883196679	4.891389296	4.503039778	4096	Derl2	6.960677153	6.918715933	6.471336197	6396	Gpr158	8.018142189	8.117692952	8.569022959	8696	Mllt4	9.096706404	8.750121953	8.86873627610996
1797	Athl1	3.531648659	2.82460495	2.561731398	4097	Derl3	1.664499835	2.449366031	1.981470519	6397	Gpr162	9.599846744	9.649225254	9.585542335	8697	Mllt6	9.657758273	9.609586926	9.74494945410997
1798	Atic	7.010366149	7.163785576	6.812689925	4098	Des	0.085897563	3.899314904	1.563712207	6398	Gpr165	2.056902337	0.858889523	1.985545955	8698	Mlph	2.411832767	3.014806129	3.79163716110998

Prkab2	6.439586593	5.995161748	6.826028465	13249	Spanxn4	1.123407552	4.805547177	3.891752385	15549	Zbtb8a	4.834868653	4.639355814	5.151303148
Prkaca	9.580411472	9.609410879	9.583611805	13250	Sparc	10.79170428	10.79721665	11.31426207	15550	Zbtb8b	4.155683438	3.419292629	4.152903153
Prkacb	11.68098084	11.60255692	11.92361383	13251	Sparcl1	14.88680199	14.57052077	14.72643441	15551	Zbtb8os	7.021943702	7.059687319	6.837860289
Prkag1	8.519029633	8.275480217	8.389336938	13252	Spast	7.627779925	7.822281824	7.645267599	15552	Zbtb9	4.78405071	5.166249633	5.228981944
Prkag2	8.865030556	8.988540931	8.901891572	13253	Spata1	3.978419171	4.107253242	2.976906149	15553	Zc2hc1a	9.146659813	9.155264212	9.326597872
Prkag3	1.048014798	0.89971311	0.987886954	13254	Spata13	5.91690107	6.472945941	6.228005936	15554	Zc2hc1c	4.072433515	4.02140226	3.78800554
Prkar1a	11.9071174	11.82252723	11.91035131	13255	Spata17	2.869854182	3.204117024	2.791443704	15555	Zc3h10	6.402422185	6.423317111	6.354914765
Prkar1b	10.90866652	10.83354467	10.96228315	13256	Spata18	3.259857304	5.206212209	2.975875932	15556	Zc3h11a	9.029074494	8.883957173	9.134832661
Prkar2a	8.20836832	7.972933879	7.91073443	13257	Spata19	2.648328771	2.678981263	2.568827119	15557	Zc3h12a	2.375298972	1.454065446	1.985529709
Prkar2b	8.944794724	8.906320957	9.387507875	13258	Spata2	7.166401635	6.935405446	7.067652646	15558	Zc3h12b	6.587049394	6.581636604	6.624244313
Prkca	10.68311645	10.67175807	10.46579788	13259	Spata20	2.07964949	4.068663902	1.562505333	15559	Zc3h12c	4.987258113	4.685656281	5.937445903
Prkcb	13.34151386	13.40847342	13.44580226	13260	Spata24	5.438120467	6.367603061	5.565395837	15560	Zc3h13	10.31994009	10.11505227	10.19432008
Prkcd	6.273227629	6.495222433	6.405918336	13261	Spata2l	6.58754511	6.621323279	6.439243932	15561	Zc3h14	8.289691103	8.306164147	8.159838213
Prkcdbp	7.620247832	7.810408757	7.741578099	13262	Spata3	1.677035996	4.292036635	1.978654377	15562	Zc3h15	10.3225496	10.27755544	10.426061
Prkce	12.03762059	11.9983839	11.74234891	13263	Spata32	1.630821448	1.909320327	0.987883457	15563	Zc3h18	8.437180698	8.293820356	8.169767957
Prkcg	9.136866159	8.903129538	8.495609232	13264	Spata33	4.259568696	5.246510223	5.304198371	15564	Zc3h3	3.671646259	3.120706396	3.886511994
Prkch	3.989721922	4.161828642	4.937622759	13265	Spata4	3.229337779	3.199849088	0.975022554	15565	Zc3h4	8.079670994	7.988411457	8.093957704
Prkci	7.688526161	7.855163455	8.008147787	13266	Spata5	5.665970179	6.035922771	5.564480957	15566	Zc3h6	7.73756088	7.61351184	8.019561899
Prkcq	5.541007651	6.209702553	5.652551455	13267	Spata6	4.555730759	7.187032084	5.022845157	15567	Zc3h7a	6.886935247	6.668807613	6.707750043
Prkcsh	7.840996199	7.645194636	7.345851974	13268	Spata7	6.106723658	6.375689117	6.319520174	15568	Zc3h7b	8.996138341	8.776800104	9.114559759
Prkcz	9.537662809	9.3132248	9.497702628	13269	Spata9	3.887519911	4.379454057	4.068395587	15569	Zc3h8	5.932609401	5.510675823	5.336949702
Prkd1	5.693485149	5.219757198	5.40565706	13270	Spatc1	1.634628301	1.48029662	1.57238948	15570	Zc3hav1	3.648802079	3.78093461	1.975464316
Prkd2	3.400940185	3.760855156	4.07080911	13271	Spats1	3.383647468	1.814394325	2.98299444	15571	Zc3hav1l	2.673917822	3.164138163	4.37784431
Prkd3	6.292711483	6.698635868	6.62403191	13272	Spats2	6.522681339	6.225578808	6.534801544	15572	Zc3hc1	7.672963662	7.810929941	7.80672887
Prkdc	6.765150806	6.537715151	6.86330199	13273	Spats2l	7.104866423	7.431033491	7.645746047	15573	Zc4h2	6.663700335	6.635142493	6.624188563
Prkg1	6.345043679	6.397818955	6.440218462	13274	Spc24	3.892413617	4.304275128	4.067235034	15574	Zcchc10	7.898628634	7.466375792	7.65258485
Prkg2	6.337648681	4.904536302	6.111022249	13275	Spc25	3.528979915	3.869871327	2.297973523	15575	Zcchc11	8.623559355	8.537782304	8.754883713
Prkra	7.208649316	6.98653368	7.067472765	13276	Spcs1	9.23761786	9.01726462	9.077993235	15576	Zcchc12	8.433705963	8.304502522	8.638374665
Prkrip1	7.471134388	7.203904058	7.179582183	13277	Spcs2	9.772289303	9.872957868	9.712980426	15577	Zcchc14	7.374541583	7.338399627	7.456004915
Prkrir	8.906817212	8.729166144	8.819197241	13278	Spcs3	6.891328624	7.353793255	7.62429997	15578	Zcchc16	4.533142019	4.49336293	3.677835262
Prkx	6.506653609	6.648145553	6.455630781	13279	Spdl1	1.090059669	2.677161537	1.981200967	15579	Zcchc17	9.68576028	9.538285966	9.431147986
Prl	2.703915119	4.084318556	0.947918399	13280	Spdya	6.208880721	6.267140023	6.407011886	15580	Zcchc18	11.44246397	11.32964109	11.39678634
Prlr	8.045896719	8.875541708	6.621624362	13281	Specc1	8.699169793	8.76259762	8.940108106	15581	Zcchc2	7.188076879	7.170378084	7.265769181
Prm1	6.265241292	10.68521582	7.792988619	13282	Specc1l	7.932235932	7.778982915	7.991301041	15582	Zcchc24	8.926482893	9.125704776	9.106699109
Prm3	0.098992682	4.362436174	3.306885375	13283	Spef1	5.431988344	4.683543959	5.407650169	15583	Zcchc3	7.515330621	7.863749395	7.602132421
Prmt1	8.600489858	8.714499814	8.627394856	13284	Spef2	5.635337539	6.572544279	4.73097639	15584	Zcchc4	6.310120605	6.408291721	6.565419951
Prmt10	5.910895294	6.075285591	5.734925302	13285	Speg	7.968967006	7.952041241	7.886856385	15585	Zcchc6	9.046431506	9.146479177	9.164911396
Prmt2	8.683895506	8.520326883	8.557380964	13286	Spem1	2.096137467	4.704039899	1.975311607	15586	Zcchc7	8.498153967	8.148606544	8.538503522
Prmt3	6.60471092	6.591347905	6.318965722	13287	Spen	9.318527793	9.171158479	9.2861343	15587	Zcchc8	6.975140843	6.836021914	7.035443943
Prmt5	8.55116353	8.35014477	8.119374454	13288	Spert	1.086810594	3.707406951	3.446479781	15588	Zcchc9	6.308384196	5.682543291	6.456343059
Prmt6	6.719409904	6.176363285	5.95643871	13289	Spesp1	1.086774581	4.232476395	1.56502381	15589	Zcrb1	10.03465177	9.972288566	10.01892417
Prmt7	6.488468245	6.6510176	6.63891747	13290	Spg11	5.726679974	6.580397217	5.980251372	15590	Zcwpw1	4.994824571	5.292957015	5.33716286
Prmt8	8.299384092	8.362514588	8.406436885	13291	Spg20	8.848673769	8.783041605	8.877868327	15591	Zdbf2	8.373505584	7.972008103	8.067526833
Prnp	12.4345093	12.174796	12.20789155	13292	Spg21	7.745615236	7.95943933	7.714720191	15592	Zdhhc1	5.70066755	5.942413528	6.535711589
Prob1	4.837233836	5.527287588	5.067967614	13293	Spg7	8.313187744	8.227013319	8.098938936	15593	Zdhhc12	5.54214579	6.075994706	5.761576794
Proca1	4.836506808	4.739206154	5.150921167	13294	Sphk1	3.524837844	2.641331375	3.568814276	15594	Zdhhc13	5.812979017	6.231599807	5.933485317
Prodh	5.40775116	5.744175027	5.652030566	13295	Sphk2	8.878273618	8.898539123	8.901829967	15595	Zdhhc14	7.390232684	7.097171886	6.707347183
Prok2	1.639986938	2.220027882	1.987003255	13296	Sphkap	8.932026245	8.719826193	8.683777753	15596	Zdhhc15	5.66273738	5.186802267	5.226983747
Prokr2	4.460420275	4.016804396	4.068850846	13297	Spi1	4.883027763	5.214375392	4.935571453	15597	Zdhhc16	6.006372955	5.645519699	5.503089518
Prom1	6.008884247	5.970747011	5.812846732	13298	Spice1	5.915296803	6.445948704	6.320563472	15598	Zdhhc17	8.329317291	8.459253735	8.591041672

1799	Atl1	9.031838556	9.093167424	9.18220236	4099	Desi1	9.160048526	9.226135632	9.218422713	6399	Gpr17	6.830896138	6.513110254	6.550299571	8699	Mlst8	7.012889682	7.17709656	7.29299850210999	Prorsd1	6.085288207	6.634730894	6.455601029	13299	Spidr	4.532876153	4.233233163	3.979142228	15599	Zdhhc18	4.783745359	4.83423717	5.150754808
1800	Atl2	7.987978358	8.137355306	8.154970896	4100	Desi2	7.104078291	7.306084204	7.149717215	6400	Gpr171	3.404287635	2.805518201	3.788787249	8700	Mlx	7.619502094	7.834897245	7.47166881411000	Pros1	6.635450059	6.829333928	6.800464528	13300	Spin1	8.433667388	8.449037477	8.495750339	15600	Zdhhc2	7.319545369	7.407725074	7.694671674
1801	Atl3	5.889699248	6.269431735	5.862105946	4101	Det1	3.083567578	3.299966846	2.784836625	6401	Gpr173	4.069420634	3.755023414	3.788825154	8701	Mlxip	5.030462868	4.470444812	4.06382708811001	Prosc	7.5803822	7.665375768	7.666616196	13301	Spin2c	5.169004415	5.365333947	5.406909349	15601	Zdhhc20	8.864941033	8.621527298	8.93137749
1802	Atm	8.0334632	7.803813438	7.721368027	4102	Dexi	8.397092976	8.08491577	8.256182278	6402	Gpr176	5.198600702	4.355590445	4.624354589	8702	Mlxipl	3.386334973	2.649946871	1.97698426411002	Proser1	6.383623721	6.279941795	6.749223711	13302	Spin4	3.085023529	2.994435629	4.070977023	15602	Zdhhc21	8.720966601	8.707818688	8.980131801
1803	Atmin	8.0805531	7.869347529	8.208945014	4103	Dffa	7.453098629	7.449806838	7.320217373	6403	Gpr179	2.401448604	1.813004041	3.153518098	8703	Mlycd	6.633232982	6.52591125	6.43940397811003	Proser2	4.241301323	3.931867041	3.563931376	13303	Spink10	6.599775619	6.531240729	6.067080409	15603	Zdhhc22	2.063427346	0.838408242	0.980759822
1804	Atn1	7.97556231	8.179116594	8.199360787	4104	Dffb	3.539158986	3.850913659	3.888723243	6404	Gpr18	1.050342801	0.894642356	0.987289436	8704	Mmaa	6.661093543	6.21228839	6.16961613911004	Proser3	1.65242774	1.440808115	2.571044442	13304	Spink2	1.759421301	6.412435964	3.884340806	15604	Zdhhc23	4.52938976	4.31040322	4.229239392
1805	Atoh8	4.239876444	4.449622753	2.781889868	4105	Dfna5	5.54033785	5.956268208	5.708396046	6405	Gpr180	5.604151861	5.478043448	5.503177322	8705	Mmab	6.037573501	6.456269928	6.2653641711005	Prox1	6.965396475	7.221165432	7.098655412	13305	Spink8	8.160161304	7.805004647	7.495424966	15605	Zdhhc24	5.122909693	5.201560756	5.067721132
1806	Atox1	8.370452903	8.624463188	8.256028706	4106	Dfnb59	0.011134731	1.930843894	0.991149012	6406	Gpr183	3.538320566	4.109091957	3.888911837	8706	Mmachc	5.776501804	5.62376086	5.43935421511006	Prox2	2.075613831	2.171369771	2.304047004	13306	Spint1	3.06791532	2.422653002	2.565725814	15606	Zdhhc3	8.103501946	8.140852948	8.328807602
1807	Atp10a	3.772278612	3.151741108	3.566113613	4107	Dgat1	6.632242724	6.424273774	6.355035631	6407	Gpr19	5.509552394	5.745951142	5.438488615	8707	Mmadhc	8.167629595	8.081002265	7.94561331411007	Proz	5.887137357	6.373642645	5.86273705	13307	Spint2	4.841979107	5.96595987	4.500264258	15607	Zdhhc4	7.290421289	7.416115456	7.227705313
1808	Atp10b	2.884329979	3.568807631	2.981295785	4108	Dgat2	7.657848377	7.612371945	7.54209283	6408	Gpr21	2.077717047	2.670303319	3.571408781	8708	Mmd	9.632781094	9.530042802	9.30186066411008	Prpf18	7.944826058	8.008189133	8.114589244	13308	Spire1	9.008751475	9.05402544	9.410566921	15608	Zdhhc5	7.627568143	7.763901838	7.687401657
1809	Atp10d	2.077763502	3.044587625	2.791033014	4109	Dgcr14	5.507162286	5.290154447	5.76212853	6409	Gpr22	10.0443492	9.673806312	9.594651653	8709	Mmd2	9.360893126	9.197700681	8.93112365111009	Prpf19	8.680090314	8.606264885	8.538381386	13309	Spire2	5.509587861	6.225255128	5.813247045	15609	Zdhhc6	6.182687857	5.617612868	5.911487084
1810	Atp11a	6.14821281	6.22896781	6.456172928	4110	Dgcr2	7.795088237	7.915301535	7.904869447	6410	Gpr25	4.984772474	5.377848216	5.229489911	8710	Mme	6.346738361	6.346748387	6.58063328611010	Prpf3	7.230033402	7.273559844	7.208831783	13310	Spn	2.647491784	2.45130665	0.977742775	15610	Zdhhc7	6.321075547	5.994531457	6.131017906
1811	Atp11b	7.682402901	8.081200861	8.17487021	4111	Dgcr6	7.641346687	7.392160241	7.389338528	6411	Gpr26	7.765464445	7.869804904	7.673728659	8711	Mmel1	4.252282366	4.22546425	4.68189793411011	Prpf31	6.794501878	6.8276331	6.579476906	13311	Spns1	6.5368001	6.229908374	6.108892913	15611	Zdhhc8	7.279817201	7.112385885	7.749003379
1812	Atp11c	6.975513635	7.077527913	7.189815542	4112	Dgcr8	6.619282847	6.565552647	6.748571277	6412	Gpr3	3.674549587	3.729622427	4.302903552	8712	Mmgt1	7.7735153	7.62512838	7.68735454311012	Prpf38a	6.587398772	6.539140664	6.666903074	13312	Spns2	7.247733642	6.881871746	6.761039775	15612	Zdhhc9	7.382602627	7.55750839	7.159924066
1813	Atp13a1	5.967353557	6.22834875	6.519679579	4113	Dgka	6.206568268	6.029849701	5.812477464	6413	Gpr34	5.4057217	5.889026626	6.089975068	8713	Mmgt2	6.473854605	6.710761981	6.83857920711013	Prpf38b	9.060207838	8.504510887	8.806480395	13313	Spock1	9.806556549	9.824139532	9.871847718	15613	Zeb1	9.277991856	9.227392082	9.523088548
1814	Atp13a2	8.489280214	8.305348195	8.341953183	4114	Dgkb	9.946076393	9.789065648	9.780875393	6414	Gpr37	8.612713609	8.985683885	8.922541016	8714	Mmp11	3.648794438	3.318670602	3.44267483811014	Prpf39	9.446650621	9.24685333	9.425045111	13314	Spock2	6.522450629	6.688360533	6.595124727	15614	Zeb2	10.58444572	10.79008077	10.59099728
1815	Atp13a3	7.375026749	7.1932439	7.503790178	4115	Dgkd	8.433198842	8.337925594	8.38093799	6415	Gpr37l1	10.38337328	10.41310702	10.47181873	8715	Mmp14	6.538234702	6.751905793	6.16925386211015	Prpf4	7.134893098	7.267743191	6.968576107	13315	Spock3	8.991338247	9.248836282	9.629363933	15615	Zeb2os	8.527636756	8.81115835	8.431113211
1816	Atp13a4	2.692396025	3.932544951	4.304478797	4116	Dgke	8.034041734	7.71255181	7.940047168	6416	Gpr39	8.340363288	7.623314554	7.623529763	8716	Mmp15	5.326583094	5.631067019	4.88552928511016	Prpf40a	9.354314989	9.323613874	9.431245278	13316	Spon1	8.563400091	8.389171337	8.218299052	15616	Zer1	8.848346758	9.009364591	8.748191331
1817	Atp13a5	3.976605402	3.75781214	3.681753521	4117	Dgkg	11.08577126	10.84034247	10.07524621	6417	Gpr4	4.476550184	4.747231427	5.151835392	8717	Mmp16	8.052547721	8.260794973	8.39808988411017	Prpf40b	7.751682747	7.639943494	7.534574974	13317	Spop	10.58935571	10.5758816	10.7053751	15617	Zfand1	5.50806985	5.672816254	5.838778328
1818	Atp1a1	11.88634306	11.94469273	12.15367713	4118	Dgkh	5.786071811	6.024036349	6.067115957	6418	Gpr45	4.882766103	5.140653868	4.438721316	8718	Mmp17	8.270225374	8.428029593	8.39377239811018	Prpf4b	10.01867189	9.884594065	10.02847492	13318	Spopl	7.023683875	6.996131159	7.320231119	15618	Zfand2a	7.238364224	6.987766202	6.760892866
1819	Atp1a2	12.85127922	12.68531037	12.9882321	4119	Dgki	5.133659289	5.670382506	6.210406681	6419	Gpr61	6.079235023	6.236094157	6.373430552	8719	Mmp19	5.121814386	4.92495119	5.3739837211019	Prpf6	8.749334691	8.780733794	8.557289185	13319	Spp1	5.288768746	4.328487501	5.566233128	15619	Zfand2b	6.305938592	5.901190023	6.130077387
1820	Atp1a3	10.83968049	10.93060238	10.9743484	4120	Dgkk	3.678197227	3.250650902	5.443901532	6420	Gpr62	10.8025422	11.15399418	10.8395206	8720	Mmp2	3.082654064	4.038058533	2.78509101911020	Prpf8	9.199635262	9.469865132	9.29741946	13320	Sppl2a	8.374984628	8.39291638	8.333139271	15620	Zfand3	7.703504653	8.11155067	7.837928364
1821	Atp1b1	14.18110029	14.04100388	14.08530894	4121	Dgkq	7.437167291	7.297222717	7.572881547	6421	Gpr63	1.050281856	1.484422984	1.572873476	8721	Mmp24	8.595862901	8.590077131	8.5612521211021	Prph	4.814849673	3.751290176	2.782557477	13321	Sppl2b	5.95605475	5.825594871	5.653197883	15621	Zfand4	3.680263855	5.327211702	4.736575655
1822	Atp1b2	10.08783849	10.07574293	10.42192709	4122	Dgkz	10.72269862	11.03904028	10.75558154	6422	Gpr65	1.630829795	1.90930412	0.987880959	8722	Mmp9	3.650732075	1.783365781	3.30404155411022	Prps1	9.831666642	9.674193796	9.509383124	13322	Sppl3	8.627917428	8.826818687	8.90191864	15622	Zfand5	9.238083584	9.240526752	9.557534193
1823	Atp1b3	9.170834711	9.257867799	9.201550906	4123	Dguok	6.875998187	7.18940489	7.078175867	6423	Gpr68	6.399047485	6.575649039	5.861903626	8723	Mmrn2	4.084700791	4.154398092	5.11256984911023	Prps1l3	6.751870334	6.463933756	6.813278976	13323	Spr	7.860952953	7.906172509	7.638127585	15623	Zfand6	8.713838259	9.159339856	8.871816288
1824	Atp2a1	3.275176514	4.5471999	5.685443344	4124	Dhcr24	7.649085159	7.518282274	7.602289101	6424	Gpr75	5.536496304	5.038887417	5.814622721	8724	Mms19	7.226821355	6.872405619	6.65207051811024	Prps2	7.299519772	7.636802365	7.119264981	13324	Spred1	9.796607659	9.595272694	10.09901421	15624	Zfa-ps	3.895220206	4.8109892	4.682338597
1825	Atp2a2	11.47618526	11.59224141	11.7869515	4125	Dhcr7	6.362619519	6.302311889	6.228360288	6425	Gpr82	1.077084019	2.69261505	2.570540133	8725	Mms22l	1.660621918	2.684583485	2.30552208211025	Prpsap1	7.393053096	7.575492284	7.542557967	13325	Spred2	4.83612528	4.882364083	5.408269313	15625	Zfat	2.876474382	2.858802266	2.983203474
1826	Atp2a3	3.252474983	3.9472119	3.789452461	4126	Dhdds	7.77276387	7.558963817	7.768190658	6426	Gpr83	6.108346438	6.522906932	6.825993994	8726	Mn1	5.863941627	5.833199325	6.3732873411026	Prpsap2	7.33586947	7.017091968	7.08856521	13326	Spred3	5.245526358	4.834249023	5.024730903	15626	Zfc3h1	7.962845281	7.898584333	7.806424614
1827	Atp2b1	13.47936697	13.67035289	13.62664583	4127	Dhdh	4.607918921	4.343734527	4.679828115	6427	Gpr84	1.639983503	1.468502841	2.309707805	8727	Mnat1	8.136469	8.093696828	8.55388561211027	Prr11	1.641667517	1.464780634	0.984590128	13327	Sprn	8.661444317	8.945991995	8.25582793	15627	Zfhx2	8.101496936	7.947616255	7.934327999
1828	Atp2b2	11.03414935	11.09566422	11.54893849	4128	Dhfr	5.752493896	6.035428536	5.911346261	6428	Gpr85	7.873408484	7.523460799	7.42266087	8728	Mnd1	2.866665574	2.680842265	1.98176779611028	Prr12	8.224235447	8.312045433	8.01891358	13328	Sprr2a2	1.040715968	0.915521505	0.989756381	15628	Zfhx3	7.510337271	8.031181729	8.758579391
1829	Atp2b3	9.686051335	9.796710106	9.652393793	4129	Dhodh	6.400758491	6.331504786	6.169597753	6429	Gpr87	2.070438018	2.181756571	3.308721477	8729	Mnd1-ps	0.045410166	1.881938584	1.9858821611029	Prr13	7.907472161	7.886803339	7.780637957	13329	Sprtn	2.072499173	3.596667139	1.564680006	15629	Zfhx4	7.130177993	7.621957796	8.406679692
1830	Atp2b4	10.34995126	9.878559275	9.817646495	4130	Dhps	7.55671998	7.817143856	7.687430069	6430	Gpr88	7.615445587	8.158209762	8.963481478	8730	Mnda	1.656606077	1.859278761	1.56670251211030	Prr14	5.511710645	6.221357555	6.303220234	13330	Spry1	3.557216194	4.85586305	3.560774735	15630	Zfp1	6.861840409	6.802765493	6.651962593
1831	Atp2c1	8.717524155	8.750279181	8.910780235	4131	Dhrs1	9.330742698	9.335920421	9.182055899	6431	Gpr89	6.938059007	6.766624968	6.837872199	8731	Mndal	5.798067542	5.552067522	4.50092847711031	Prr14l	8.323579176	8.289067856	8.189340958	13331	Spry2	8.662418755	9.057919558	9.062255916	15631	Zfp101	6.229682637	6.044246327	6.129597573
1832	Atp5a1	11.61346098	11.59718311	11.65063189	4132	Dhrs11	3.240050223	3.781916294	3.152044397	6432	Gprasp1	11.98033096	11.75048723	12.10862663	8732	Mns1	5.208009369	5.551307256	5.2284311611032	Prr15	2.397416131	3.585242636	2.302647607	13332	Spry4	4.417888478	5.365768144	4.732772183	15632	Zfp105	4.402743757	3.990563152	4.372086216
1833	Atp5b	11.36018982	11.22782175	11.31145084	4133	Dhrs13	4.661851802	3.988725392	4.43931394	6433	Gprasp2	10.39118624	10.15698011	10.52883902	8733	Mnt	7.30951039	7.483646289	7.49595700811033	Prr16	3.097676599	2.787254068	4.738754649	13333	Spryd3	9.138617897	9.25396132	9.11438148	15633	Zfp106	10.87737407	10.79705706	10.82154769
1834	Atp5c1	9.84365508	9.793551106	9.827064551	4134	Dhrs3	6.345619997	6.545455852	5.979350877	6434	Gprc5b	7.863515674	8.306235441	8.164818703	8734	Moap1	7.986974999	7.825858453	8.04625860911034	Prr18	9.891161754	10.22558767	9.876305146	13334	Spryd4	6.121417962	5.790770777	6.130613395	15634	Zfp108	4.166547414	3.625194944	3.562049565
1835	Atp5d	10.37866532	10.56347954	10.40624708	4135	Dhrs4	6.778474123	6.647612519	6.455563292	6435	Gprc5c	3.076085832	3.17147478	2.563276205	8735	Mob1b	4.723938951	5.211296474	4.98115596911035	Prr22	0.042815788	3.079877224	0.984088241	13335	Spryd7	6.807602053	6.757472892	6.825725982	15635	Zfp11	4.081706848	4.494126531	3.78548439
1836	Atp5e	10.27044028	10.33472107	10.26768361	4136	Dhrs7	7.375806469	7.789815127	7.198701702	6436	Gprin1	8.818014562	8.77244472	8.526801929	8736	Mob2	6.603995827	6.710344347	6.4390385911036	Prr3	6.45043181	6.337177016	5.563490795	13336	Spsb1	6.99971767	7.397480655	6.849971396	15636	Zfp110	6.128077906	6.569011039	6.503807168
1837	Atp5f1	10.33451819	10.371032	10.39148481	4137	Dhrs7b	7.258627306	7.161848035	6.991154571	6437	Gprin3	4.254797652	3.901816887	5.268493138	8737	Mob3a	5.397712438	5.045773057	5.47325712911037	Prr32	3.788505979	5.366580714	2.972708399	13337	Spsb2	5.328910371	5.850886479	5.653453691	15637	Zfp111	5.163029282	5.012625086	5.441609073
1838	Atp5g1	9.665392815	9.70548349	9.467768199	4138	Dhx15	8.974817411	8.777375874	8.734836607	6438	Gps1	8.468311583	8.400086175	8.587483866	8738	Mob3b	6.379308219	5.880547389	5.95729401811038	Prr36	6.185583965	6.323070139	6.002669563	13338	Spsb3	6.524658104	6.852742165	6.623801283	15638	Zfp112	4.171799034	4.486021873	4.148863285
1839	Atp5g2	8.842283676	8.982909946	8.960164088	4139	Dhx16	6.081464389	6.103386454	6.045747419	6439	Gps2	7.546034836	7.617449219	6.921906006	8739	Mob3c	2.677924966	4.117635931	3.44057027711039	Prr5	4.534909649	3.721954201	4.14936192	13339	Spsb4	3.407511176	3.281454165	4.152280929	15639	Zfp113	6.572380297	6.698159075	6.405839321
1840	Atp5g3	12.20538646	12.16412892	12.32038622	4140	Dhx29	6.900695646	6.95775463	6.991407415	6440	Gpsm1	7.034229774	7.116158443	7.119827478	8740	Mob4	9.976689019	9.809278285	9.81444571611040	Prr5l	7.271114242	7.648462763	7.479998654	13340	Sptan1	11.07775955	11.00260485	11.0521089	15640	Zfp114	3.062619771	2.163033349	3.686941277
1841	Atp5h	11.35138499	11.2724739	11.07006835	4141	Dhx30	8.284918071	8.230115929	8.355145038	6441	Gpsm2	6.602445353	6.497183595	6.149119554	8741	Mobp	10.12087769	10.39970094	9.9180216511041	Prr7	5.74847302	5.151201642	5.188502896	13341	Sptb	8.764125186	9.027981608	9.016193401	15641	Zfp119a	4.233884831	3.857943889	3.301843503
1842	Atp5j	9.596307406	9.544927315	9.563042021	4142	Dhx32	7.516954507	7.77990123	7.741428212	6442	Gpsm3	3.085936013	3.760857154	4.376802102	8742	Mocos	2.885491665	3.447491172	2.98101349811042	Prrc1	5.855731618	5.188646838	5.653265948	13342	Sptbn1	12.51705348	12.45909177	12.59238765	15642	Zfp119b	3.537621592	3.853778036	4.070403746
1843	Atp5j2	11.35785528	11.34361464	11.26998998	4143	Dhx33	6.975859187	7.12456286	6.933980262	6443	Gpt	5.498660419	5.165982291	4.563123807	8743	Mocs1	4.072058171	3.749086359	3.30009249611043	Prrc2a	8.95062199	9.126993607	8.859578634	13343	Sptbn2	10.66261928	10.6708905	10.56588459	15643	Zfp12	6.959904701	6.419307707	6.666645051
1844	Atp5k	9.919773594	9.952949099	9.780831718	4144	Dhx34	3.988080517	4.238039032	4.228888597	6444	Gpt2	7.230926563	7.393398423	7.274532491	8744	Mocs2	8.406704335	8.28869784	8.23255030211044	Prrc2b	10.20587549	10.1977452	10.14615376	13344	Sptbn4	7.057571506	7.077021196	7.302425938	15644	Zfp120	7.58682169	7.237172117	7.149555851
1845	Atp5l	9.823296096	9.587707286	9.435243283	4145	Dhx35	4.475422508	4.845616812	4.981521047	6445	Gpx1	9.527906232	9.578986376	9.716405089	8745	Mocs3	5.532788333	5.739341626	5.37350393511045	Prrc2c	11.00751537	10.71943873	10.88311084	13345	Sptlc1	8.068736528	8.067586704	7.898769562	15645	Zfp128	3.260650858	3.745926403	4.442649103
1846	Atp5o	11.15725464	10.89298418	10.90181097	4146	Dhx36	9.09192326	9.175583409	8.999486632	6446	Gpx2-ps1	2.671519929	2.832552785	3.567881308	8746	Mog	8.695418321	8.920983283	8.56856369511046	Prrg1	5.608832263	6.088998825	5.862351323	13346	Sptlc2	6.419405111	6.424772905	6.519637962	15646	Zfp13	5.498125241	4.925061698	5.441340727
1847	Atp5s	6.231680827	6.469500674	6.149342329	4147	Dhx37	3.983490557	3.839695802	3.679868752	6447	Gpx3	5.087138414	5.481360647	5.912108234	8747	Mogat1	2.065833084	1.859158239	2.9866253411047	Prrg2	5.65803116	5.122371532	5.149916902	13347	Sptssa	6.821492454	6.548180031	6.887144071	15647	Zfp131	8.864413673	8.520183145	8.549782816
1848	Atp5sl	7.66514293	7.639149551	7.781136729	4148	Dhx38	6.986902614	6.957326773	7.150357476	6448	Gpx4	8.852205276	9.156491962	8.784030552	8748	Mogs	7.218984855	6.714689993	7.06743808811048	Prrg3	7.012387435	7.015742323	7.480396197	13348	Sptssb	3.384708164	1.81055269	3.685851731	15648	Zfp133-ps	4.075629955	4.444432442	3.887172959

1849	Atp6ap1	10.71716151	10.87154394	10.63012625
1850	Atp6ap1l	5.6295101	5.080501444	5.406849964
1851	Atp6ap2	11.06338333	10.91006488	10.79775961
1852	Atp6v0a1	10.32365594	10.26306145	10.38835161
1853	Atp6v0a2	7.861720786	7.877272162	7.700920469
1854	Atp6v0b	11.08273357	11.28193753	11.16361888
1855	Atp6v0c	11.23020816	11.067233	10.98839805
1856	Atp6v0c-ps2	11.23147766	11.0690352	10.98979808
1857	Atp6v0d1	9.345147186	9.576524314	9.344084752
1858	Atp6v0e	8.135781115	8.209239245	8.072687759
1859	Atp6v0e2	10.45765441	10.50791776	10.27343556
1860	Atp6v1a	12.51076551	12.45041441	12.27888811
1861	Atp6v1b2	11.67905661	11.51412093	11.54745598
1862	Atp6v1c1	10.35188823	10.27566969	10.21479617
1863	Atp6v1c2	3.658590393	3.426980087	3.98306079
1864	Atp6v1d	11.92329627	11.77915052	11.84918811
1865	Atp6v1e1	11.82074346	11.73640121	11.6825809
1866	Atp6v1f	11.50417888	11.39762255	11.31535613
1867	Atp6v1g1	9.306455825	9.247930337	9.010602126
1868	Atp6v1g2	11.14918481	10.975517	10.84182975
1869	Atp6v1h	9.657783216	9.506575916	9.62207088
1870	Atp7a	6.622619346	7.277410541	6.799865755
1871	Atp8a1	10.37860668	10.28991795	10.49177115
1872	Atp8a2	3.394006525	3.008792422	3.303546194
1873	Atp8b1	4.161465593	4.314850471	3.887188298
1874	Atp8b2	5.633912699	5.798568616	6.151790411
1875	Atp8b3	2.40612108	4.284004151	1.976555989
1876	Atp8b5	2.054136751	2.483097444	1.570161267
1877	Atp9a	11.38084104	11.31159528	11.24501192
1878	Atp9b	7.125296873	7.065117887	7.199206173
1879	Atpaf1	6.310979476	6.057970769	6.609711988
1880	Atpaf2	5.083102542	5.264860598	5.763167204
1881	Atpif1	11.54811903	11.2919579	11.37978679
1882	Atr	5.170709292	5.874333132	5.595613528
1883	Atraid	8.691456303	8.778165337	8.57611331
1884	Atrip	6.648745436	6.954697861	6.565151824
1885	Atrn	8.552476571	8.484393075	8.761453966
1886	Atrnl1	7.605267621	7.768167551	8.185009449
1887	Atrx	12.13721081	12.02756223	12.13163262
1888	Atxn1	9.656299404	9.643162978	10.07152962
1889	Atxn10	10.26671802	10.32672533	10.32761323
1890	Atxn1l	6.364093657	6.42757815	6.302199547
1891	Atxn2	9.372951493	9.492794631	9.594785108
1892	Atxn2l	6.809067035	7.136483111	6.747892872
1893	Atxn3	7.200372473	7.288605197	7.527212217
1894	Atxn7	7.092503018	7.355829134	7.189444878
1895	Atxn7l1	6.056573471	6.200978148	5.886658942
1896	Atxn7l2	4.675456217	5.428745931	5.814907536
1897	Atxn7l3	9.196769293	9.141615434	9.310998533
1898	Atxn7l3b	11.22974116	11.28898221	11.41305842

4149	Dhx40	7.635524308	7.833573704	7.594545671
4150	Dhx57	7.513065344	6.921036658	7.246841691
4151	Dhx58	0.055248401	1.870271065	2.307476007
4152	Dhx8	7.906606208	7.888439325	7.673286477
4153	Dhx9	9.091804436	9.099933333	9.152445023
4154	Diablo	7.089637285	7.089791567	6.70762634
4155	Diap1	8.563693181	8.373047088	8.363673067
4156	Diap2	8.209145881	8.066743778	8.134891282
4157	Diap3	5.024904117	4.358216646	4.888763423
4158	Dicer1	7.80234923	8.237093184	8.007978218
4159	Dido1	7.945181074	8.052306066	8.223366842
4160	Diexf	6.605736763	6.575694605	7.170607964
4161	Dimt1	5.98430901	6.034741817	5.147638832
4162	Dio2	9.457876297	9.467026535	9.418732991
4163	Dio3	5.472020305	3.44011013	1.555491176
4164	Dip2a	7.572633326	7.572119994	7.687456503
4165	Dip2b	8.052227742	8.12361713	8.213751383
4166	Dip2c	8.415358744	8.242771349	8.292892512
4167	Diras1	8.1732567	8.182313425	8.237454784
4168	Diras2	9.738888342	9.93754583	9.874827353
4169	Dirc2	8.011264445	8.100777405	8.114536869
4170	Dis3	5.941551502	5.82929922	6.208871146
4171	Dis3l	6.126791844	5.93839447	6.045386479
4172	Dis3l2	6.053617654	6.132980652	5.623490398
4173	Disp1	3.666955373	3.932594691	3.887609066
4174	Disp2	8.784753114	8.873956378	8.999654551
4175	Dixdc1	8.451395916	8.455972116	8.758282928
4176	Dkc1	8.68006492	8.534630093	8.522985352
4177	Dkk2	2.890101674	2.830247806	2.786190339
4178	Dkk3	10.1002289	10.28727153	10.15989419
4179	Dkkl1	6.289672455	6.281212319	7.069322969
4180	Dlat	8.539693017	8.411150211	8.376515742
4181	Dlc1	8.709497729	8.645572505	8.797137111
4182	Dld	8.702523801	8.760178354	8.771221871
4183	Dleu2	4.936787261	5.242643998	5.339474604
4184	Dleu7	4.250522948	3.250562597	4.889536247
4185	Dlg1	9.466180042	9.257414317	9.48586475
4186	Dlg2	9.993236247	9.77424258	10.03668936
4187	Dlg3	9.695177299	9.709957318	9.632826161
4188	Dlg4	11.36534813	11.38818984	11.31816662
4189	Dlg5	7.278734788	7.249625605	6.886487837
4190	Dlgap1	12.71738811	12.50422245	12.46831808
4191	Dlgap2	8.683565337	8.646107953	8.735063094
4192	Dlgap3	9.351934659	9.542633975	9.418832666
4193	Dlgap4	10.58912338	10.40679685	10.41982716
4194	Dlgap5	1.045667297	1.493087198	1.989546061
4195	Dlk1	8.202239534	6.846495955	6.226057972
4196	Dlk2	5.576159609	6.185683047	5.980416286
4197	Dll1	3.398554189	2.819931443	3.790059341
4198	Dll3	4.983301825	4.641601783	5.110760197

6449	Gpx6	1.632744707	1.48443161	1.572874488
6450	Gpx7	4.464166191	4.008169624	4.441439154
6451	Gpx8	6.120502918	6.258605176	4.883292873
6452	Gramd1a	6.973351186	6.630213309	6.800289506
6453	Gramd1b	4.083614812	4.36600518	4.440348738
6454	Gramd1c	3.763100978	3.317324224	3.568565452
6455	Gramd2	0.01659866	0.915559632	0.989760899
6456	Gramd3	7.155686933	7.199166891	6.980053483
6457	Gramd4	6.23456678	6.536438259	6.838425962
6458	Grap	4.395569553	4.006760194	3.562426623
6459	Grap2	1.636447987	2.916214146	1.987805936
6460	Grasp	7.270948228	7.228514473	7.503792772
6461	Grb10	6.7113882	6.503649782	6.945918562
6462	Grb14	8.447106073	8.636330798	8.426996658
6463	Grb2	10.18386582	10.18764227	10.22435694
6464	Grb7	4.336136841	5.348471149	4.680215778
6465	Grcc10	10.45328054	10.44634182	10.25725441
6466	Greb1	2.635473861	1.454108166	1.569330912
6467	Greb1l	3.784259435	4.091265576	4.683606602
6468	Grem1	2.412143163	3.172390398	2.787043583
6469	Grem2	5.663864529	5.147591762	5.439019798
6470	Grhl1	6.790178359	6.664526258	6.1291746
6471	Grhl2	3.764446872	1.783194281	3.984837149
6472	Grhl3	0.042714502	2.483157624	0.984108277
6473	Grhpr	6.754267872	7.136104189	6.898849636
6474	Gria1	12.15471653	12.15521792	11.89807765
6475	Gria2	12.01389082	11.9195663	11.85801977
6476	Gria3	10.70885351	10.53738408	10.65773014
6477	Gria4	7.816586756	7.660345793	8.561665602
6478	Grid1	6.750042533	6.774524092	6.638652811
6479	Grid2	1.650996442	1.444017661	2.57130998
6480	Grik1	5.913046338	5.990157162	5.679751354
6481	Grik2	6.938407058	6.691909837	6.980101502
6482	Grik3	6.793061636	6.9941342	6.735164818
6483	Grik4	6.690565159	6.457039167	5.562755377
6484	Grik5	8.255133817	8.523482145	8.355209259
6485	Grin1	9.899207424	9.876567563	10.04745559
6486	Grin1os	9.586273152	9.429911291	9.627485735
6487	Grin2a	4.982097585	4.58980084	5.230123849
6488	Grin2b	11.0287319	10.90814233	10.66908586
6489	Grin2c	6.678736567	6.686717218	6.45559729
6490	Grin3a	7.992448449	7.283129023	8.051758896
6491	Grin3b	6.454856995	6.27852623	6.609900265
6492	Grina	10.32250113	10.42778179	10.15362404
6493	Grip1	6.096424428	5.646571813	5.652612346
6494	Grip2	4.339519425	4.524683928	5.710792867
6495	Gripap1	8.770328514	8.723681603	8.774459464
6496	Grk4	5.28997514	6.026725933	4.677745885
6497	Grk5	5.60420666	5.592174124	5.623886217
6498	Grk6	8.529873174	8.209619995	8.088520679

8749	Mok	4.391866568	4.563879482	3.67971150311049
8750	Mon1a	6.142473096	5.530726501	5.56410655111050
8751	Mon1b	6.102184177	6.180813983	6.18986758811051
8752	Mon2	8.102553028	7.929734619	8.09901256811052
8753	Morc2a	4.486808775	4.727899486	5.50458513411053
8754	Morc2b	1.691202469	4.207710819	1.97547802211054
8755	Morc3	7.055879999	6.588526499	6.89900507411055
8756	Morc4	5.209858341	5.230670173	5.37290967511056
8757	Morf4l1	11.45237125	11.46987246	11.5873241811057
8758	Morf4l1-ps1	10.31534287	10.24607575	10.2243317211058
8759	Morf4l2	9.30616549	9.000298523	8.77425745311059
8760	Morn1	3.778105561	4.252049114	4.30490780411060
8761	Morn2	7.489569656	7.698936957	7.4476165811061
8762	Morn4	7.701397668	7.620559242	7.56504234411062
8763	Morn5	4.824451776	4.492265623	4.50495620311063
8764	Mospd1	4.784984427	5.465864276	4.88635217411064
8765	Mospd2	5.437991954	5.916498778	5.6531880711065
8766	Mospd3	6.912143593	6.589557222	6.82560696511066
8767	Mov10	1.67742479	1.81856085	2.30215800411067
8768	Moxd1	4.248944116	4.860593958	3.78531694211068
8769	Mpc1	8.328788309	8.518095269	8.47989857111069
8770	Mpc2	9.544101041	9.499421064	9.4995971711070
8771	Mpdu1	8.780831364	8.852571546	8.65941318811071
8772	Mpdz	7.318428641	7.229953491	7.38109878711072
8773	Mpeg1	5.600730966	5.454934913	5.18873259811073
8774	Mpg	6.697514778	7.068481632	7.0135372711074
8775	Mphosph10	8.847841995	8.509849454	8.48770816811075
8776	Mphosph6	8.108965524	8.480012926	8.37245165411076
8777	Mphosph8	11.05484756	10.85249937	10.728985711077
8778	Mphosph9	6.57516979	6.730276354	7.19980162511078
8779	Mpi	7.611515776	7.678576055	7.70131535111079
8780	Mplkip	6.06181247	6.328106204	6.55020777311080
8781	Mpnd	8.455370417	8.640402267	8.24193249311081
8782	Mpp1	8.050740702	7.756379179	7.85659275211082
8783	Mpp2	8.259379216	8.081547092	8.3597279711083
8784	Mpp3	8.134692743	8.106518042	7.9913242911084
8785	Mpp4	6.871650275	6.480905687	6.389041411085
8786	Mpp5	9.168363053	9.335754726	9.4250550311086
8787	Mpp6	8.907208278	8.785357664	9.0728265811087
8788	Mpp7	4.993973024	5.260348826	5.43962090311088
8789	Mppe1	5.255546068	6.059118111	5.65265698711089
8790	Mpped1	9.746893111	9.458470969	9.44355653111090
8791	Mpped2	8.515247949	8.736976276	8.61668116111091
8792	Mprip	9.957047485	9.980056489	9.99542084111092
8793	Mpst	6.899779236	7.279942032	6.87498205911093
8794	Mpv17	8.615256989	8.452527165	8.45977024911094
8795	Mpv17l	8.234931037	8.29859959	8.0187767611095
8796	Mpv17l2	7.951556942	8.347775948	8.19927912211096
8797	Mpzl1	6.634875102	6.783477622	6.31910335211097
8798	Mpzl2	4.605123913	3.80166365	5.26776309911098

Prrg4	3.227301358	3.204132212	2.568173541
Prrt2	10.36941777	10.49720815	10.40835785
Prrt3	7.627374417	8.041882891	7.687448908
Prrt4	2.090577234	3.183076822	3.569463402
Prrx1	6.034282779	5.662130476	6.3904764
Prrx2	2.874376474	3.038817812	3.306868
Prss12	5.665939016	5.952744535	5.534005138
Prss16	2.862322055	2.191078095	2.570270931
Prss22	1.643322275	1.461116002	2.309026537
Prss23	6.168791194	6.486842008	6.208730068
Prss3	1.043216601	1.497709741	0.989116475
Prss35	4.457850646	3.937252122	3.300177548
Prss36	4.166624964	4.554159046	3.885793954
Prss45	1.070166057	2.701014962	2.987788625
Prss46	1.082112791	3.484153018	2.792937103
Prss50	2.650885528	2.673705549	3.155332329
Prss53	4.066103485	3.153734775	3.983180972
Prss56	0.008381654	1.518469423	0.991898458
Prss57	5.024629313	4.222353896	4.372057656
Prss8	6.399025921	6.460276532	6.247205478
Prtg	4.608144375	5.054525569	5.068431409
Prtn3	3.382972634	3.191636724	2.566302853
Prune	7.12461699	7.349362168	7.088522524
Prune2	7.251156134	7.023147816	7.659954205
Psap	12.50841192	12.50358218	12.36259613
Psat1	8.93536167	9.030734541	8.744739144
Psd	10.92603173	11.11832122	10.94144233
Psd2	7.572821344	7.442866994	7.673518754
Psd3	11.16967439	10.79574327	10.59925731
Psd4	2.051284157	1.891484937	0.985097961
Psen1	7.01112969	6.99780201	7.218821819
Psen2	7.545522892	7.213523898	6.910225293
Psenen	8.141188457	8.311001274	7.99106082
Psg16	1.103095375	2.861602977	2.566696101
Psip1	10.94161107	10.83986295	10.84802723
Pskh1	5.203475646	5.170902803	4.734525911
Psma1	8.502033706	8.162830318	8.241976227
Psma2	10.07491828	10.04598555	10.10927825
Psma3	9.057265483	8.896060903	8.880872265
Psma4	10.04317419	9.802022106	9.842618499
Psma5	7.808419215	7.803679599	7.74810448
Psma6	9.446806813	9.574844414	9.395720014
Psma7	10.80758816	10.6312273	10.76625655
Psma8	2.07044837	2.453332394	2.305246954
Psmb1	10.40129591	10.32965736	10.2814646
Psmb10	8.218556052	7.952119274	7.767725725
Psmb11	0.042793769	1.885155465	2.795689756
Psmb2	7.010807757	6.998420005	6.850138033
Psmb3	8.5519692	8.470774005	8.463788077
Psmb4	9.873185954	9.872936029	9.583534808

13349	Spty2d1	7.595659027	7.58024069	7.471794985
13350	Spx	0.040255007	2.485695839	2.309369163
13351	Sqle	7.643064059	7.990635294	7.940319424
13352	Sqrdl	4.666304971	5.534310555	4.147108775
13353	Sqstm1	10.82873045	10.79274231	10.75063894
13354	Sra1	6.987733947	7.0963217	7.046120536
13355	Srbd1	5.29698362	5.862866667	5.680167963
13356	Src	7.531547118	7.666337383	7.487766507
13357	Srcap	6.682527775	6.813516406	7.228528128
13358	Srcin1	10.21741897	10.32011052	10.206426
13359	Srd5a1	5.330949893	5.324765406	5.788343865
13360	Srd5a3	6.167665366	6.025027377	6.535627094
13361	Srebf1	7.32845744	7.533939272	7.319827657
13362	Srebf2	7.229935598	7.485715957	7.638950341
13363	Srek1	9.257584202	8.99976756	8.939889898
13364	Srek1ip1	8.493687757	8.404185098	8.368007377
13365	Srf	8.974438726	8.692850675	8.564864466
13366	Srfbp1	4.945217824	5.29592678	5.406499654
13367	Srgap1	7.490923944	7.645130106	7.838222776
13368	Srgap2	7.724085422	7.571474002	7.80672465
13369	Srgap3	10.91413537	10.8287129	10.76788438
13370	Srgn	6.504081565	5.917550376	6.456261757
13371	Sri	8.981136164	8.840443534	8.825438456
13372	Srl	5.718544058	5.600823358	5.337641142
13373	Srm	8.063097964	7.956656418	7.996826405
13374	Srp14	10.24271746	9.975880598	10.01063521
13375	Srp19	9.165022836	9.167159502	8.988387819
13376	Srp54a	8.433619878	8.518058292	8.439443133
13377	Srp54b	8.168238739	8.334593765	8.346436247
13378	Srp54c	7.849890194	8.440590086	8.018830208
13379	Srp68	8.198543887	8.02796894	7.727846949
13380	Srp72	10.10686138	9.952886027	9.870221406
13381	Srp9	8.913018626	8.86350475	8.564789357
13382	Srpk1	8.051497123	7.754990408	8.175026627
13383	Srpk2	11.09730353	10.84393128	11.13545801
13384	Srpk3	3.243740295	3.775474297	3.441936637
13385	Srpr	7.75919334	7.645537207	7.397508301
13386	Srprb	5.967557161	6.728088898	6.372415259
13387	Srpx2	3.254167528	3.853819955	3.78909495
13388	Srr	9.539356452	9.227932798	9.265399359
13389	Srrd	7.238729036	7.143448231	6.679702786
13390	Srrm1	10.05929224	10.14520509	10.10799015
13391	Srrm2	11.25978853	11.04530404	11.2070056
13392	Srrm3	7.364524335	6.995167312	7.199213068
13393	Srrm4	7.164808964	6.959702499	6.77438593
13394	Srrm4os	5.275846395	4.757114805	4.439641504
13395	Srrt	8.787531112	8.708452651	8.491594483
13396	Srsf1	9.049147135	9.066884736	8.962942958
13397	Srsf10	8.213592805	7.706054702	7.862555878
13398	Srsf11	9.833593238	9.762770927	9.609294847

15649	Zfp14	5.248532638	4.82775857	5.338340473
15650	Zfp141	5.805592639	5.592579442	5.708286535
15651	Zfp142	6.588002854	6.4949113	6.487617687
15652	Zfp143	5.575737084	5.501816504	5.887118809
15653	Zfp146	2.081726581	2.663535373	3.686653329
15654	Zfp148	10.16807038	10.03464078	10.30644393
15655	Zfp157	6.50502183	6.192283117	6.503979454
15656	Zfp160	7.289080654	7.14129698	7.189486867
15657	Zfp169	0.090386786	2.167131591	3.154981256
15658	Zfp174	3.985050328	3.836374517	4.303913755
15659	Zfp180	8.355750077	8.109054399	8.051352725
15660	Zfp182	5.402979996	5.187818968	5.788537636
15661	Zfp184	1.663144374	2.879275266	1.98177544
15662	Zfp185	2.679873473	4.462113715	2.978279947
15663	Zfp189	6.307152532	6.005186041	5.706601465
15664	Zfp191	8.250414849	8.378095939	8.260783418
15665	Zfp2	6.541436852	6.721036224	6.503102773
15666	Zfp202	1.695114848	3.444400382	3.56822448
15667	Zfp207	7.751910907	7.260263961	7.652530346
15668	Zfp212	7.708100447	7.489762383	7.439451296
15669	Zfp213	4.590671907	4.014466423	3.437252718
15670	Zfp217	3.984082858	4.381334035	3.980775645
15671	Zfp219	8.623941885	8.923718219	8.579778473
15672	Zfp229	3.974277023	3.29782435	3.302043405
15673	Zfp235	5.538484008	5.391036548	5.624499759
15674	Zfp236	6.455876746	6.466863224	6.550007082
15675	Zfp239	6.439822509	6.592431828	6.800604828
15676	Zfp248	5.038938684	5.228196745	5.472505311
15677	Zfp251	7.587938423	7.406290185	7.631200391
15678	Zfp26	6.649139775	6.258732681	6.787961773
15679	Zfp260	9.370086626	9.171049802	9.09368995
15680	Zfp263	5.912280974	6.052777428	5.86275325
15681	Zfp266	6.838642222	7.322801971	7.119323864
15682	Zfp27	5.130568601	5.824169026	5.301243741
15683	Zfp273	3.262116382	4.174467855	5.029380179
15684	Zfp275	7.057845862	6.834285821	7.209084266
15685	Zfp276	4.337272766	5.095566273	4.934805646
15686	Zfp277	6.92645856	7.189750003	6.760893016
15687	Zfp28	4.472074109	4.422350725	5.026820495
15688	Zfp280b	4.786330481	5.005036907	5.338495632
15689	Zfp280c	5.441551651	5.352150211	5.838504504
15690	Zfp280d	8.539745822	8.30833779	8.542289039
15691	Zfp281	9.287531184	9.048750636	9.230262898
15692	Zfp282	6.420576495	6.310974135	6.439423932
15693	Zfp286	5.80691604	5.695863188	5.680382699
15694	Zfp287	4.943959675	5.118696829	5.681900968
15695	Zfp292	10.38537457	10.27032826	10.30078848
15696	Zfp296	2.65253336	2.67030168	3.794469315
15697	Zfp3	6.383241643	6.44095342	6.108771206
15698	Zfp30	7.564696782	7.638967384	7.781113187

1899	AU019823	10.18106914	9.995001105	10.02709687	4199	Dll4	2.079735337	3.041651288	2.303089645	6499	Grm1	8.503188683	8.582126755	8.931465519	8799	Mr1	5.025419662	3.988250498	4.1483122411099	Psmb5	9.702839393	10.00947324	9.906304561	13399	Srsf12	4.843538299	5.573150105	4.978633064	15699	Zfp300	4.66836139	4.468649322	4.980629419
1900	AU021063	5.123020747	4.520772425	5.338808381	4200	Dlst	8.657858427	8.763525047	8.467698096	6500	Grm2	6.057788253	6.384126118	6.373189742	8800	Mrap	1.060790935	2.217136281	1.98662820211100	Psmb6	9.461966738	9.374970337	9.459797306	13400	Srsf2	8.948003217	9.116802509	9.091172643	15700	Zfp316	7.715491755	7.574112647	7.666824544
1901	AU021092	2.084532021	3.191590362	3.306391357	4201	Dlx1	7.152543129	7.084324343	6.68061182	6501	Grm3	9.011075035	8.964715319	9.299886354	8801	Mrap2	5.402238598	5.294474869	5.73607774211101	Psmb7	10.08462627	10.00910083	9.919545363	13401	Srsf3	9.821743181	9.560322895	9.638189697	15701	Zfp317	5.69763069	5.904732759	6.228968299
1902	AU022252	4.659295898	4.487729927	4.066203842	4202	Dlx1as	7.10940503	6.876218806	6.708175509	6502	Grm4	7.262022484	7.118351142	7.881405545	8802	Mras	10.22381802	10.06771183	10.154920211102	Psmb8	4.546729924	4.835354345	4.837669253	13402	Srsf4	7.498807341	7.734569749	7.50345906	15702	Zfp318	9.250135478	9.012312604	9.142398422
1903	AU023762	2.404838208	2.64963199	2.982187972	4203	Dlx2	5.274176622	4.710415215	4.736840272	6503	Grm5	11.20004348	10.84036563	10.9629647	8803	Mrc1	5.166564673	4.732861106	5.37342205111103	Psmb9	2.414533628	3.561365577	3.303713755	13403	Srsf5	8.89502501	9.368153369	9.315462592	15703	Zfp319	5.208411379	5.762309349	4.885885945
1904	AU040320	4.541067212	4.41592795	4.981566592	4204	Dlx5	3.88098963	3.142292382	3.300656393	6504	Grm7	7.83635184	7.966659792	8.247025534	8804	Mrc2	3.868065057	2.406874853	2.98111890611104	Psmc1	10.38771451	10.38577326	10.28260685	13404	Srsf6	7.556335522	7.586286557	7.631136536	15704	Zfp322a	9.057513343	8.977592029	8.960140373
1905	AU040972	1.073712411	2.894075069	2.307186071	4205	Dlx6	2.395694864	2.666938457	2.303095475	6505	Grm8	6.859071713	6.784436649	7.180984074	8805	Mre11a	4.784603927	5.201944148	5.50546330111105	Psmc2	9.715392629	9.735701753	9.738212658	13405	Srsf7	8.957257604	8.989945195	8.945718671	15705	Zfp324	5.28017423	4.796305225	4.300148993
1906	AU041133	5.57219906	5.537662555	5.935409914	4206	Dlx6os1	6.244002037	5.88640846	5.787563324	6506	Grn	8.177292401	8.189084855	7.761158128	8806	Mreg	5.031648303	4.637710348	5.47418079511106	Psmc3	10.42912775	10.30751753	10.22075762	13406	Srsf9	9.066136169	9.112344157	9.013438753	15706	Zfp326	9.110772108	9.003057533	8.951536245
1907	Auh	8.346907746	8.217021213	8.37672439	4207	Dlx6os2	1.071970956	1.444131434	1.984524499	6507	Grp	7.594818643	6.22514418	5.955816823	8807	Mrfap1	11.54098655	11.40808628	11.2584045211107	Psmc3ip	5.695364146	5.383352404	5.76154014	13407	Srxn1	9.132817815	8.997628676	9.002346508	15707	Zfp329	7.491142613	7.317285761	7.701102216
1908	Aunip	3.047254079	2.475573523	1.568942252	4208	Dmap1	6.486396612	6.266310936	6.169826419	6508	Grpel1	8.494518297	8.541430842	8.694316467	8808	Mrgbp	3.392547632	3.562952643	3.98475107311108	Psmc4	8.838212388	8.813166205	8.568627418	13408	Ss18	7.564646453	7.715516956	7.708046244	15708	Zfp330	7.571731064	7.525055612	7.748588153
1909	Aup1	7.278928273	7.249255008	7.159995472	4209	Dmbx1	2.919683079	4.160181798	3.979152084	6509	Grpel2	6.691709208	6.137128519	6.246509514	8809	Mrgpre	4.404874807	4.649221229	5.11161289611109	Psmc5	9.247963041	9.177405037	9.220765247	13409	Ss18l1	7.794934218	7.820843935	7.844204144	15709	Zfp334	6.16469095	6.166065678	5.957461853
1910	Aurka	3.069718426	3.787635359	1.976983577	4210	Dmd	9.217573175	9.108771468	9.487917283	6510	Grpr	2.654119587	3.340032909	2.567326361	8810	Mrgprf	2.084523996	1.816773455	2.98318376511110	Psmc6	8.969074621	8.647046463	9.016244942	13410	Ssb	11.5358612	11.39807761	11.45672783	15710	Zfp335	5.573340426	5.84307893	5.680568593
1911	Aurkaip1	9.066331322	9.153523174	8.945709752	4211	Dmgdh	3.400119403	3.548121338	4.23204992	6511	Grrp1	4.171620663	4.655585259	4.565901154	8811	Mri1	4.322375471	4.667824117	3.56257327811111	Psmd1	10.39332994	10.2807472	10.22313916	13411	Ssbp1	6.272255432	6.452722358	6.56525165	15711	Zfp341	4.660289796	4.5442548	4.372299013
1912	Aurkb	2.357844787	0.915537039	0.989758222	4212	Dmkn	4.325875919	4.367686883	4.625750548	6512	Grsf1	8.661877206	8.705154814	8.85662107	8812	Mrln	1.661773478	2.881152557	1.98208393111112	Psmd10	7.522761568	7.546860094	7.372242182	13412	Ssbp2	8.709085553	8.175501386	8.751708407	15712	Zfp345	0.011141891	1.512815409	0.991147113
1913	Auts2	9.12981191	8.998166586	8.774307059	4213	Dmp1	2.084513196	2.427626917	3.153803169	6513	Grtp1	4.087365865	4.222450386	5.026859916	8813	Mrm1	5.368111444	5.51023281	5.50352460711113	Psmd11	8.28530059	8.377363091	8.447704547	13413	Ssbp3	10.45867197	10.33876669	10.30416828	15713	Zfp346	6.571132733	6.406875015	6.51932607
1914	AV039307	2.074626623	3.049263731	2.568392858	4214	Dmpk	4.258011253	5.213283321	4.680614816	6514	Grwd1	6.187443943	5.900273713	6.265999829	8814	Mro	5.568742303	5.362551569	5.10842124711114	Psmd12	9.059837186	8.487194441	8.806566388	13414	Ssbp4	8.959174306	8.705469333	8.564881201	15714	Zfp35	6.888391105	7.038966952	7.035449746
1915	AV051173	2.069246378	3.354134523	2.79270779	4215	Dmrt3	3.227343673	2.673668845	2.791433473	6515	Gsap	3.079187334	2.627180228	2.979766647	8815	Mroh1	6.604552558	6.683942908	6.8874042511115	Psmd13	9.459500904	9.240769619	9.230268298	13415	Ssc4d	1.644982448	0.862766835	1.985872103	15715	Zfp352	0.024646947	1.488663459	1.989086188
1916	Aven	5.365460692	5.653996317	5.53530167	4216	Dmrta1	2.635514304	0.858596335	1.569316409	6516	Gsdmd	2.063330233	1.437746257	0.980795308	8816	Mroh2a	3.391568165	2.636009607	3.30417647711116	Psmd14	9.151934258	9.095122047	9.213662035	13416	Ssfa2	6.253353629	6.724274327	6.813561769	15716	Zfp354a	5.250581776	5.229233779	5.595908298
1917	Avil	1.050284792	1.905574334	2.574721929	4217	Dmrta2	6.584334583	6.654041833	5.956514834	6517	Gse1	7.912867759	7.979097749	7.637972853	8817	Mroh4	1.054623808	2.72033151	0.98618868811117	Psmd2	10.20968697	10.18235381	10.03796044	13417	Ssh2	8.70139562	8.423193095	8.333098955	15717	Zfp354b	4.245270135	4.166760983	3.980002228
1918	Avl9	8.568435093	8.451699855	8.871998056	4218	Dmrtb1	2.075668102	3.047710571	1.980676923	6518	Gsg1	2.722556031	6.768997226	4.147233818	8818	Mroh5	2.624103355	1.909320078	0.98788341911118	Psmd3	9.58438038	9.717870914	9.616625511	13418	Ssh3	5.397002242	5.464779852	5.505247182	15718	Zfp354c	7.596343283	7.558336483	7.800428965
1919	Avp	1.659320287	2.88451767	2.305784274	4219	Dmrtc1a	5.915356574	6.69608061	5.956932966	6519	Gsg1l	6.507476861	6.974041428	6.519014256	8819	Mroh7	1.066527572	2.902308276	0.98311222111119	Psmd4	10.73874102	10.72737483	10.69336958	13419	Ssmem1	0.029886118	2.723049419	0.986740898	15719	Zfp358	6.873510442	6.506440009	6.707895751
1920	Avpi1	7.327966201	7.194545256	7.169657445	4220	Dmtf1	8.097209814	7.886066817	8.311166996	6520	Gsk3a	8.932175004	8.995756271	9.010736896	8820	Mrpl1	6.54156507	6.630947975	6.94629641211120	Psmd5	7.759153787	7.592548535	7.825777912	13420	Ssna1	7.218383349	7.096702086	6.934052303	15720	Zfp36	4.238131018	3.415918902	3.564899539
1921	Avpr1a	2.370134509	1.468566326	1.57101637	4221	Dmtn	9.485672196	9.538518243	9.758191232	6521	Gsk3b	11.70402489	11.71773515	11.88462896	8821	Mrpl10	7.732573199	7.848429791	8.0516123511121	Psmd6	8.713387834	8.733078434	8.545945013	13421	Sspn	7.068949817	6.997156306	6.933984812	15721	Zfp362	6.923842598	6.929526	6.813173384
1922	Avpr1b	2.062084344	2.198460911	2.307190414	4222	Dmwd	8.835099203	8.809881161	8.655890698	6522	Gskip	7.968094161	7.726412765	7.68034098	8822	Mrpl11	8.193855684	8.379284285	8.05675841211122	Psmd7	10.64930774	10.46349264	10.36581656	13422	Ssr1	9.830465357	9.887779555	9.918118497	15722	Zfp365	10.80075295	10.70814456	10.9573083
1923	AW011738	3.22674862	0.80072633	2.304226514	4223	Dmxl1	8.28084746	8.364783683	8.72169338	6523	Gsn	7.887867324	8.063554697	7.787223101	8823	Mrpl12	8.58832419	8.715196922	8.50740130211123	Psmd8	9.831830803	9.643490205	9.692521834	13423	Ssr2	7.815319023	7.809219018	7.754680625	15723	Zfp367	7.05849014	7.084925315	7.346669963
1924	AW046200	1.078812855	3.062532008	1.56671283	4224	Dmxl2	10.15047003	9.930414096	10.06215649	6524	Gspt1	9.424800386	9.411532021	9.475878703	8824	Mrpl13	7.447738482	7.658853973	7.29240971311124	Psmd9	7.417729417	7.086214239	7.078061055	13424	Ssr3	9.108448871	9.155493426	9.013354008	15724	Zfp369	4.830742261	4.065958339	4.735618323
1925	AW112010	3.52670617	2.837475315	3.151482826	4225	Dna2	1.062804237	0.867183986	0.984077188	6525	Gspt2	8.073728268	7.837817742	7.837964891	8825	Mrpl14	7.472106367	7.547537482	7.04551857811125	Psme1	8.057396013	8.138573942	7.747896172	13425	Ssr4	9.03131387	8.879688037	8.806507397	15725	Zfp36l1	8.289831444	8.22568542	8.242075845
1926	AW146154	5.810519823	6.337928567	5.812385999	4226	Dnaaf1	4.175780318	5.286353638	5.409479919	6526	Gsr	6.439415382	6.55179609	6.887601764	8826	Mrpl15	5.932262633	4.992676875	5.73578531211126	Psme2	6.167936176	6.266375855	5.956652529	13426	Ssrp1	9.242716983	9.139303974	8.951410227	15726	Zfp36l2	6.738437612	6.781707699	6.87518061
1927	AW209491	7.125462467	7.306997869	7.302106389	4227	Dnaaf2	5.858344674	5.478350508	5.371477795	6527	Gss	5.435574782	5.263846955	4.732541107	8827	Mrpl16	8.297961879	7.749031324	7.9744042611127	Psme2b	7.229111076	7.105081537	6.886502531	13427	Sssca1	7.384157226	7.453751959	7.447737581	15727	Zfp37	7.057458733	7.037914089	6.862325482
1928	AW495222	4.40693415	4.844646766	4.371035553	4228	Dnaaf3	2.395551307	3.799572126	0.974688451	6528	Gsta1	3.062606378	1.394396708	3.154631071	8828	Mrpl17	8.239529325	8.100444981	8.19932848511128	Psme3	8.203650934	8.161326837	7.96295492	13428	Sst	10.11227244	9.984046543	9.845705145	15728	Zfp382	4.25132729	4.227409401	4.440076099
1929	AW549877	7.588232034	7.626391544	7.564942665	4229	Dnaaf5	2.898629459	4.112472933	2.977817721	6529	Gsta3	2.39017117	1.411024963	2.568624922	8829	Mrpl18	9.048968656	8.96199838	8.9457646911129	Psme4	7.50857368	7.979020289	7.991438797	13429	Sstr1	7.599965608	6.923773262	7.218762173	15729	Zfp383	4.888172698	5.204657157	4.934306474
1930	AW551984	5.319653082	3.796943314	5.22974402	4230	Dnah1	3.532915951	3.552254672	3.566810373	6530	Gsta4	8.437398951	8.512115035	8.109110966	8830	Mrpl19	7.589129527	7.872801965	7.72818700611130	Psmf1	7.326705766	7.016656298	7.329147122	13430	Sstr2	4.783000421	5.168200731	4.980331425	15730	Zfp384	4.984934751	4.837721143	5.408413076
1931	AW554918	6.651368469	6.816192095	7.024951318	4231	Dnah10	2.869093859	3.20553561	2.304266056	6531	Gstcd	1.680389638	3.460533936	3.444126274	8831	Mrpl2	7.926610774	8.086147561	8.17981060911131	Psmg1	6.367551982	6.076350542	6.45556204	13431	Sstr3	6.73496269	6.624379133	6.503922874	15731	Zfp385a	6.421738032	6.711144644	6.911537889
1932	AW822252	2.391229336	2.874110969	1.980925331	4232	Dnah11	3.529793852	4.588386823	2.78609075	6532	Gstk1	6.010548236	6.288324899	5.934126534	8832	Mrpl20	8.981402018	9.062280365	8.97150669411132	Psmg2	6.651316995	6.816291453	6.875081436	13432	Sstr5	3.047272879	1.450696545	1.568934493	15732	Zfp385b	8.468139582	8.82394748	8.274528281
1933	Awat2	2.046587797	0.889830525	0.986723564	4233	Dnah17	1.056775247	2.717619576	0.98563445	6533	Gstm1	10.51813406	10.38382549	10.27917309	8833	Mrpl21	5.285071948	4.832944839	5.62619120111133	Psmg3	7.410565425	7.053806814	7.34642094	13433	Ssu72	8.835489339	8.985757957	8.904836299	15733	Zfp385c	5.395292614	5.341898852	4.298845867
1934	Axin1	6.23227763	6.34428108	6.471998378	4234	Dnah2	1.082104553	3.214893168	1.566013275	6534	Gstm2	5.076287536	5.243101513	4.980642432	8834	Mrpl22	6.998257223	7.010118972	6.92262726111134	Psmg4	6.620683154	7.002341326	6.652251198	13434	Ssx2ip	8.887807266	8.88239735	8.841065356	15734	Zfp386	6.861952365	6.860095449	7.057188608
1935	Axin2	6.191931072	6.437545701	6.800999758	4235	Dnah5	5.313877288	3.243107205	4.888958814	6535	Gstm3	1.054649327	1.476262382	0.986182118	8835	Mrpl23	7.648539686	7.497785806	7.14954761611135	Pspc1	8.85535422	8.846197473	8.844161878	13435	St13	9.811705062	9.692654031	9.654155516	15735	Zfp389	2.876561644	3.333460054	2.789706833
1936	Axl	7.548846666	7.664402217	7.67348817	4236	Dnah6	3.093498608	4.102321094	4.069419753	6536	Gstm4	5.599381331	5.395804978	5.265335552	8836	Mrpl24	8.129959015	8.037537168	7.84394404511136	Psph	6.650588902	6.840805896	6.471360097	13436	St18	7.555216731	7.540103307	6.956482876	15736	Zfp39	5.498381136	5.009725312	4.886621127
1937	AY512931	0.005608334	0.940318714	0.992698372	4237	Dnah7a	3.062622182	2.866145024	2.567305471	6537	Gstm5	11.05848542	11.0190619	10.73482963	8837	Mrpl27	8.822159991	8.895976797	9.01350702911137	Psrc1	4.603825046	4.536270652	4.888138369	13437	St3gal1	6.328930461	6.650599848	6.108539022	15737	Zfp395	4.081673364	2.949250689	4.505191434
1938	Azgp1	2.887744695	1.782063178	3.303951589	4238	Dnah7b	3.407724657	3.748399096	3.888035164	6538	Gstm6	5.801294869	5.655193974	5.625022503	8838	Mrpl28	7.428030429	7.590347816	7.14973326711138	Pstk	5.664729225	5.564480788	5.813553565	13438	St3gal2	9.031208135	9.003783524	8.771120561	15738	Zfp397	6.823489219	7.007962566	7.119779645
1939	Azi2	8.742306377	8.723476769	8.96314532	4239	Dnah8	2.86234588	2.191015715	2.570264297	6539	Gstm7	7.781643685	8.139178905	7.819082454	8839	Mrpl3	7.438572209	7.503083369	7.46372955611139	Pstpip1	2.872999528	2.162903984	3.154616656	13439	St3gal3	6.075830872	5.745682011	6.210323305	15739	Zfp398	4.662796026	4.288655247	4.839351314
1940	Azin1	9.237917638	9.234402316	9.335386668	4240	Dnah9	5.750786525	6.245681309	4.560359724	6540	Gsto1	7.506134887	7.52585414	7.189077198	8840	Mrpl30	8.531529934	8.414907047	8.60215529511140	Pstpip2	3.789748151	4.158058274	4.066794595	13440	St3gal4	5.08058153	5.337264319	5.266097636	15740	Zfp40	6.189307829	6.282340779	6.302169772
1941	Azin2	5.089821075	6.440368511	5.471142525	4241	Dnaic1	6.385700963	6.686498936	6.594889591	6541	Gsto2	2.076703161	3.59140679	1.563404308	8841	Mrpl32	7.393012709	7.193531539	7.14959273911141	Ptar1	5.0902674	5.794547907	6.131071619	13441	St3gal5	9.974847005	9.834172568	9.632821892	15741	Zfp407	5.940831069	5.830713441	6.302409991
1942	B020004J07Rik	1.032340788	0.933566573	0.991897432	4242	Dnaic2	3.655761172	3.302636562	3.440804772	6542	Gstp1	9.222011966	9.093479958	8.815932414	8842	Mrpl33	8.623169725	8.681335066	8.31966823111142	Ptbp1	6.165217844	6.288699778	5.59354269	13442	St3gal6	5.473746518	5.388221062	5.888046919	15742	Zfp408	3.891221736	4.436399789	2.973334461
1943	B130006D01Rik	2.636612196	1.450591119	0.982605627	4243	Dnaja1	10.94776286	10.73246749	10.78248815	6543	Gstp2	9.16746279	9.091178216	8.774277228	8843	Mrpl34	9.240357373	9.274828564	9.37239186311143	Ptbp2	9.180729341	8.992665364	8.815918692	13443	St5	5.936150438	6.093904739	5.533803647	15743	Zfp41	4.830950764	4.937095094	4.371334048
1944	B130034C11Rik	3.540877175	3.283982101	2.97645377	4244	Dnaja2	10.47709649	10.37594742	10.38192345	6544	Gstt1	5.961097757	5.442652802	6.28519327	8844	Mrpl35	8.124782386	7.939802662	8.09373452311144	Ptbp3	7.849186745	7.802325532	7.85640476	13444	St6gal1	6.290825433	6.469282207	6.246452123	15744	Zfp410	5.986233319	5.904204523	5.910782575
1945	B230110G15Rik	2.647425476	3.210065312	1.981767999	4245	Dnaja3	7.033335894	6.926275684	7.109758161	6545	Gstt2	3.399241576	3.660812094	3.889862125	8845	Mrpl36	7.411423535	7.423033693	7.01271530211145	Ptcd1	8.387462012	8.037314982	7.904617298	13445	St6gal2	1.066563336	1.454135434	2.308386724	15745	Zfp414	5.402337853	5.678486437	5.762611031
1946	B230118H07Rik	8.694859388	8.506275414	8.609371702	4246	Dnaja4	7.72310967	7.891881284	7.455637576	6546	Gstt3	4.896463752	4.952882618	5.472114618	8846	Mrpl37	7.807396163	7.599156698	7.55732368411146	Ptcd2	7.917628053	7.398289849	7.345992813	13446	St6galnac2	2.695049151	5.560077379	3.437282519	15746	Zfp418	3.389082188	1.794542815	1.975844389
1947	B230206H07Rik	3.78054178	4.247690958	3.787468486	4247	Dnajb1	8.467730695	8.264158429	8.199095515	6547	Gstt4	0.005608334	0.940318714	0.992698372	8847	Mrpl38	6.962751482	6.957526767	7.06776413911147	Ptcd3	7.419745804	7.337867124	7.208605511	13447	St6galnac3	4.934168003	4.843171158	4.370832348	15747	Zfp420	7.132943326	6.681438251	6.812927945
1948	B230208H11Rik	1.636462732	2.226191758	1.571916867	4248	Dnajb11	6.420708794	6.662030026	6.580222018	6548	Gstz1	6.875703808	7.058491857	6.991273016	8848	Mrpl39	7.722929174	7.444728426	7.55753261211148	Ptch1	2.657465324	1.818516861	2.983322776	13448	St6galnac4	6.665320481	7.063970386	6.354298767	15748	Zfp422	6.440307101	6.758475679	6.503175558

1949	B230209E15Rik	10.02724432	9.878124959	10.10030126	4249	Dnajb12	6.886511695	6.81657404	7.024999775	6549	Gsx1	1.07199612	1.870239804	0.981690161	8849	Mrpl4	7.659379044	8.053411053	7.96873314611149	Ptchd1	7.457221346	7.550133409	8.03566113	13449	St6galnac5	7.592666147	7.046721954	6.84987946	15749	Zfp423	6.330523282	6.566598137	6.721925621
1950	B230216N24Rik	3.556464932	4.297752717	4.566222791	4250	Dnajb13	3.231243485	3.695163614	0.973920719	6550	Gsx2	1.632761768	0.894741757	2.57471993	8850	Mrpl40	7.487664958	7.204717882	7.1796855611150	Ptchd2	6.035215059	6.233716895	5.812056421	13450	St6galnac6	9.248035623	9.491547841	9.201532931	15750	Zfp426	6.794794235	6.780103256	7.013866694
1951	B230217C12Rik	8.220756301	8.258991005	8.583794115	4251	Dnajb14	9.388744548	9.4118574	9.317607515	6551	Gt(ROSA)26Sor	6.635403981	6.993140291	6.564716561	8851	Mrpl41	8.44221669	8.34546082	8.31978934411151	Ptchd4	6.795896306	6.627222272	6.874708134	13451	St7	6.470570423	6.001901738	6.265405754	15751	Zfp428	9.202156689	9.25155602	9.374526387
1952	B230217O12Rik	5.726735386	5.715760766	6.067962782	4252	Dnajb2	8.68683114	8.496762859	8.2882231	6552	Gtdc1	7.411610265	7.45279722	7.587555809	8852	Mrpl42	8.397484415	8.149706503	8.27460418311152	Ptcra	1.050307405	1.484375143	0.987298527	13452	St7l	6.588681204	6.941336666	6.264470323	15752	Zfp429	4.64350854	3.546543899	3.682137225
1953	B230219D22Rik	10.20975322	10.23251462	10.17228842	4253	Dnajb3	4.253370352	5.758649441	3.784057974	6553	Gtf2a1	8.532166606	8.689836252	8.680450677	8853	Mrpl43	8.708927416	8.279672707	8.45167943611153	Ptdss1	8.486086038	8.4809377	8.561157388	13453	St8sia1	6.708448408	6.672832262	6.826089881	15753	Zfp433	3.99648792	4.65282002	4.839706563
1954	B230307C23Rik	4.255300021	4.539577143	4.503562185	4254	Dnajb4	8.540254213	8.4514621	8.768007134	6554	Gtf2a1l	0.032489996	1.476313179	1.987807724	8854	Mrpl44	6.489283996	6.311189128	6.53484711154	Ptdss2	6.521232414	6.52873882	6.02355919	13454	St8sia2	4.611022259	5.340333677	5.4078481	15754	Zfp438	4.667134584	4.641040497	5.025483246
1955	B230319C09Rik	2.051257219	0.87602971	2.989326242	4255	Dnajb5	8.265887793	8.288370898	8.213443715	6555	Gtf2a2	7.374659517	7.13010387	7.44783521	8855	Mrpl45	8.209273042	8.002025926	8.05677162111155	Pten	9.789600315	9.612142059	9.978657139	13455	St8sia3	10.57031488	10.45341459	10.58641676	15755	Zfp442	5.371404016	5.744930316	6.248303605
1956	B230323A14Rik	1.103083225	1.821119632	2.983530739	4256	Dnajb6	8.858150392	8.732836573	8.793836546	6556	Gtf2b	7.365279772	7.149052658	7.456030895	8856	Mrpl46	7.580290297	7.48801466	7.63830907611156	Pter	8.442097877	8.309289923	8.02951523	13456	St8sia4	6.913822861	6.626617557	6.720945521	15756	Zfp444	7.143632663	6.731719795	6.81293624
1957	B230354K17Rik	7.664067863	7.399655711	7.422912777	4257	Dnajb7	0.04524999	3.23276124	1.985904953	6557	Gtf2e1	5.474374685	6.039173555	5.680783964	8857	Mrpl47	7.967055841	7.579416521	7.58734282111157	Ptgdr	0.083769581	2.445534957	2.568373475	13457	St8sia5	6.76713924	6.884027255	6.800217227	15757	Zfp445	9.207202548	9.070950535	9.304234662
1958	B2m	10.79059196	10.90863924	10.78570752	4258	Dnajb8	0.035101819	2.491170837	2.573693355	6558	Gtf2e2	6.90329005	6.952774182	7.389658017	8858	Mrpl48	7.800668513	7.879399184	7.81313036811158	Ptgdr2	3.983949537	4.173006485	4.229852803	13458	St8sia6	4.081951626	4.082880884	4.840861837	15758	Zfp446	5.280430337	5.247542501	4.564405283
1959	B330016D10Rik	4.084967032	4.546772995	4.440042138	4259	Dnajb9	6.457250412	6.783237982	6.80057469	6559	Gtf2f1	8.472112818	8.412475881	8.184460063	8859	Mrpl49	7.86869566	7.946663637	7.81274806911159	Ptgds	13.72781647	12.90467105	13.68895486	13459	Stab1	5.667415206	6.308792211	5.335839151	15759	Zfp449	4.085078327	3.71978982	4.682055885
1960	B3galnt1	7.649795158	7.451387583	7.728438296	4260	Dnajc1	8.5879917	8.391760208	8.380769346	6560	Gtf2f2	7.3254677	6.813070383	6.76096925	8860	Mrpl50	7.912581822	7.57057433	7.73485035411160	Ptger1	5.277002352	4.703946236	4.624549331	13460	Stab2	2.850057092	0.880480893	0.985627488	15760	Zfp451	7.695323508	7.618245198	7.793886982
1961	B3galnt2	6.523739277	6.523872692	6.405893435	4261	Dnajc10	8.600392555	8.625526669	8.751546636	6561	Gtf2h1	8.451078281	8.353144394	8.511461357	8861	Mrpl51	8.074393479	7.977479224	7.89876603411161	Ptger3	3.855957469	1.410572812	3.155680474	13461	Stac	3.990546158	3.388963094	4.937460932	15761	Zfp454	4.830858551	4.478216007	4.624076285
1962	B3galt1	7.970030556	8.141750821	8.38539421	4262	Dnajc11	7.410648534	7.416884452	7.256048522	6562	Gtf2h2	6.208981652	6.6816609	5.760161897	8862	Mrpl52	8.57976838	8.456566461	8.17943624511162	Ptger4	3.53369662	2.819251624	3.682618307	13462	Stac2	7.320840927	7.851612051	7.869080445	15762	Zfp455	4.244935881	3.923764486	3.886514176
1963	B3galt2	9.736132045	9.363567627	9.357220331	4263	Dnajc12	6.083170216	6.061198787	6.653281614	6563	Gtf2h3	5.250273312	5.547027797	5.227872888	8863	Mrpl53	9.060532761	8.987530074	8.90176332811163	Ptges	4.158554211	4.320561177	2.781980601	13463	Stac3	1.068383063	1.450767251	2.308079458	15763	Zfp456	1.669110964	2.871131746	1.563435582
1964	B3galt4	3.882544779	4.252867069	3.680749256	4264	Dnajc13	7.760394542	7.849292778	7.800131153	6564	Gtf2h4	5.039437842	5.70630367	4.932573511	8864	Mrpl54	7.772190774	7.444889205	7.45570104511164	Ptges2	7.05907697	7.386936534	7.246817882	13464	Stag1	8.425560543	8.491603284	8.771262508	15764	Zfp458	5.123981801	4.518602324	5.913604116
1965	B3galt5	7.643804664	7.675504738	7.637947187	4265	Dnajc14	7.708498095	7.580510931	7.188957841	6565	Gtf2h5	10.36461168	10.23558387	10.25379619	8865	Mrpl55	7.846210742	7.244851626	7.25582358111165	Ptges3	9.884687706	9.872603627	9.790522608	13465	Stag2	9.031949502	9.012468525	9.164916313	15765	Zfp459	3.252472297	3.545832331	4.376736832
1966	B3galt6	6.058183766	6.36744745	5.886258181	4266	Dnajc15	7.229871703	7.222238559	7.139797875	6566	Gtf2i	9.196705143	9.090381455	9.096317865	8866	Mrpl57	8.062607762	8.068466871	7.89289874511166	Ptges3l	6.541338575	6.604660718	6.774618132	13466	Stam	7.887931464	7.904042834	7.800048473	15766	Zfp46	6.635264253	6.794610599	6.609251685
1967	B3gat1	9.668817105	9.48909716	9.391476268	4267	Dnajc16	5.541774272	6.056876093	6.047274604	6567	Gtf2ird1	5.573392324	5.353077124	6.357138743	8867	Mrpl9	6.457363937	6.696981662	6.7216597311167	Ptgfr	2.656562868	2.661965088	3.986995045	13467	Stam2	7.144320897	7.050251738	6.774210808	15767	Zfp462	8.183859246	7.87954461	8.385326654
1968	B3gat2	8.514363968	8.518878789	8.397929402	4268	Dnajc17	5.750021199	5.220619493	5.788178877	6568	Gtf2ird2	6.143961597	5.812242006	6.024680855	8868	Mrps10	6.573972645	6.76913796	6.6802986211168	Ptgfrn	5.866469649	6.002535376	6.801570927	13468	Stambp	7.319152645	7.34561379	7.480047896	15768	Zfp467	6.681269037	6.616506442	6.761333066
1969	B3gat3	8.514727349	8.467722186	8.350633472	4269	Dnajc18	8.397706725	8.325634795	8.380912891	6569	Gtf3a	6.762868037	6.469679207	6.456005323	8869	Mrps11	6.45363873	6.22916996	6.26536411711169	Ptgis	2.660941844	2.652882923	2.78902102	13469	Stambpl1	5.910611542	5.721049757	5.887489923	15769	Zfp472	5.886658276	6.257994497	5.838114241
1970	B3glct	7.886631311	7.638526607	7.844426112	4270	Dnajc19	6.769122972	6.728067494	7.0244125	6570	Gtf3c1	8.235606633	8.238305839	8.41890052	8870	Mrps12	9.010515839	8.835524423	8.6699253411170	Ptgr1	4.157873853	4.103303345	3.680710581	13470	Stamos	3.064688783	1.82102719	3.686515394	15770	Zfp473	0.021988666	0.904796865	0.988487133
1971	B3gnt2	7.732174123	7.926179327	7.874853382	4271	Dnajc2	8.547882453	8.552635712	8.542291015	6571	Gtf3c2	7.473576219	7.776562392	7.594709105	8871	Mrps14	7.912597574	7.838692462	7.71464122711171	Ptgr2	7.605778514	8.023787813	7.916634366	13471	Stap1	1.085346139	1.419414049	3.156263192	15771	Zfp474	1.656608032	3.062477931	3.157062749
1972	B3gnt4	5.53127773	5.202929782	4.733893669	4272	Dnajc21	8.971908391	8.780115838	8.895917481	6572	Gtf3c3	7.268702267	6.834433835	7.160104366	8872	Mrps15	8.611654571	8.606517111	8.45159229411172	Ptgs1	6.231857884	6.689421279	6.788344852	13472	Stap2	2.382182812	2.193392743	2.793619539	15772	Zfp488	4.082211022	4.302146359	4.682668899
1973	B3gnt5	3.37929755	3.341266996	1.562781022	4273	Dnajc24	5.720533641	5.42200987	5.864018957	6573	Gtf3c4	7.17853594	7.290686302	7.364026458	8873	Mrps16	7.801298787	7.768905923	7.74818549211173	Ptgs2	4.728675581	5.049896114	5.109845802	13473	Star	5.597858585	5.082749223	5.864977068	15773	Zfp493	4.657539462	4.230597558	4.302323059
1974	B3gnt8	2.672130629	2.629555531	2.786328453	4274	Dnajc25	5.399894395	5.48903119	5.372829914	6574	Gtf3c5	4.724492618	4.84148799	5.267489982	8874	Mrps17	8.091428865	8.161022108	7.95719634311174	Ptgs2os	1.634648382	1.480252504	1.572384308	13474	Stard10	7.843110009	8.244665923	8.189599037	15774	Zfp503	6.438984812	6.292585317	6.666829137
1975	B3gnt9	6.38362177	6.296791553	6.580563358	4275	Dnajc27	9.201783159	9.049591528	9.111863736	6575	Gtf3c6	8.229732908	8.128080137	8.018811853	8875	Mrps18a	7.641687939	7.4891874	7.32849431311175	Ptgs2os2	2.625533419	1.484350769	1.572865002	13475	Stard13	5.701291844	6.517652048	6.338028767	15775	Zfp507	5.442288337	5.56124022	6.321589165
1976	B3gntl1	6.963869737	7.333765708	7.046075725	4276	Dnajc28	6.604826327	6.618097454	6.74832986	6576	Gtpbp1	8.318519164	8.208486248	8.29295006	8876	Mrps18b	6.692637592	6.467919625	6.59510277311176	Pth1r	5.599860693	5.486680176	4.978624952	13476	Stard3	6.806719665	6.645651957	6.72168817	15776	Zfp51	4.412601236	5.010075054	4.980166547
1977	B430212C06Rik	2.663069607	3.454885853	1.559107853	4277	Dnajc3	9.71316598	9.504948766	9.370089912	6577	Gtpbp10	4.337121431	5.473041729	5.110611781	8877	Mrps18c	7.022969954	7.421022411	6.82509368411177	Pthlh	2.425602822	2.991904932	4.629138229	13477	Stard3nl	6.964029266	6.923091206	7.098990131	15777	Zfp511	5.5757206	5.767452593	5.81288483
1978	B430319G15Rik	2.422199259	4.334943223	2.784842229	4278	Dnajc30	6.998829622	7.277627059	7.209346666	6578	Gtpbp2	6.457593974	6.929971843	6.694320088	8878	Mrps2	7.260788765	7.043600072	7.38099087711178	Ptk2	8.797717633	8.89462927	8.673501487	13478	Stard4	6.48840051	6.637969846	6.888164686	15778	Zfp512	8.033411349	7.75650171	7.838048778
1979	B4galnt1	8.202874344	8.054226086	7.728037153	4279	Dnajc4	6.792691389	6.73562694	6.708227232	6579	Gtpbp3	5.323842796	5.236398967	4.562823748	8879	Mrps21	9.265370326	9.168450955	9.1648200411179	Ptk2b	11.01646403	11.28110631	10.7339849	13479	Stard5	5.506236823	5.676365096	5.503677191	15779	Zfp513	6.211188289	6.409300002	6.565546429
1980	B4galnt2	1.050308657	0.894716843	1.988641066	4280	Dnajc5	10.5643206	10.51468193	10.57058717	6580	Gtpbp4	8.996140332	8.811826754	8.790487393	8880	Mrps22	7.738096416	7.599117032	7.57236913211180	Ptk7	3.401607842	3.946659142	3.301579813	13480	Stard6	4.156270328	4.325042517	3.438928851	15780	Zfp516	6.345278315	6.044540754	6.208932162
1981	B4galnt4	4.463151856	3.83359514	4.150937245	4281	Dnajc5b	0.062373812	4.084982322	1.567089551	6581	Gtpbp6	6.754691983	7.260285231	7.284400513	8881	Mrps23	8.304239249	8.27281695	8.26542625511181	Ptma	11.18845424	11.31974034	11.10667146	13481	Stard7	9.257260607	9.067104312	8.960114856	15781	Zfp518a	6.861620641	7.080225923	7.046522492
1982	B4galt1	5.03487658	5.006510179	4.786231339	4282	Dnajc6	9.682227088	9.597632302	9.662996725	6582	Gtpbp8	4.476046552	4.414235397	4.887904731	8882	Mrps24	7.627009176	7.374817895	7.5947551611182	Ptms	10.61277799	10.58286819	10.4637983	13482	Stard8	5.603211076	5.289853999	5.813788458	15782	Zfp518b	6.664090807	6.37407884	6.666837204
1983	B4galt2	7.447309322	7.646716243	7.70135966	4283	Dnajc7	10.1249838	10.22926635	10.11311677	6583	Gtse1	0.057761482	2.19832779	1.567786304	8883	Mrps25	9.733516184	9.800726047	9.81761946411183	Ptn	10.27512451	9.893887514	10.2432914	13483	Stat1	5.60551127	5.250867751	6.025390373	15783	Zfp52	4.78219713	4.837301968	5.229403358
1984	B4galt3	7.570861654	7.193459493	7.519439313	4284	Dnajc8	10.58832041	10.47974843	10.53168855	6584	Gtsf1	1.126772155	4.753660731	3.791543824	8884	Mrps26	7.680685133	7.732806276	7.83188666511184	Ptov1	8.52776181	8.674601207	8.463717047	13484	Stat2	5.476186864	5.886864084	5.86315042	15784	Zfp521	5.033331884	4.63405161	5.229008399
1985	B4galt4	6.438198575	6.713180115	6.406145374	4285	Dnajc9	7.191237673	7.538660314	7.701372422	6585	Gtsf1l	1.070165639	2.701015473	3.310530878	8885	Mrps27	6.188021704	6.429824226	6.26585860311185	Ptp4a1	8.141808532	8.377799149	8.288341765	13485	Stat3	8.576147833	8.576423079	8.427002634	15785	Zfp523	8.603568187	8.583566059	8.5035946
1986	B4galt5	6.88832549	6.925106263	6.968735458	4286	Dnal1	8.679925027	8.681210049	8.507450103	6586	Guca1a	1.048007965	1.488686396	1.573373981	8886	Mrps28	6.151069384	6.660026595	6.73530488411186	Ptp4a2	10.98063147	10.99780323	10.85572138	13486	Stat4	1.672676786	3.894796154	3.154642725	15786	Zfp524	4.544663857	5.560456706	4.838149491
1987	B4galt6	9.758267265	9.845144791	9.746512576	4287	Dnal4	7.879530368	7.639793948	7.463674168	6587	Guca2a	0.005608334	0.940318714	0.992698372	8887	Mrps30	7.579405278	7.445426139	7.43120819611187	Ptp4a3	5.732207531	6.689914333	6.624536314	13487	Stat5a	2.877133818	3.033392648	2.983043413	15787	Zfp526	2.093327885	3.451457655	3.792269761
1988	B4galt7	0.040283628	0.871611226	1.986624	4288	Dnali1	8.493150081	8.393637225	8.061951087	6588	Gucd1	3.27526262	5.072060622	5.070588083	8888	Mrps31	7.738672833	7.744969564	7.68036848611188	Ptpdc1	6.959619421	6.435118003	6.652606953	13488	Stat5b	6.082240192	6.364350221	6.864187243	15788	Zfp53	3.554892016	3.821602119	4.149593928
1989	B4gat1	8.919682404	8.895849266	8.841037431	4289	Dnase1	6.126886995	6.500224909	6.62467941	6589	Gucy1a2	8.473284139	8.440478064	8.905042304	8889	Mrps33	8.912955171	8.792279499	8.81924133111189	Ptpmt1	8.004559712	7.983813351	7.98578306	13489	Stat6	7.09271913	7.4102721	7.406577127	15789	Zfp532	7.570698393	7.369949834	7.159624568
1990	B630005N14Rik	8.642422674	8.440833542	8.656044747	4290	Dnase1l1	2.874441541	3.195552071	1.979095707	6590	Gucy1a3	8.087193926	8.112284663	8.765007821	8890	Mrps34	7.974682245	8.014422125	7.58697242511190	Ptpn1	9.177958522	8.799993855	8.942884876	13490	Stau1	7.822380368	7.814370124	7.93423646	15790	Zfp536	6.938445377	7.539896637	6.92248954
1991	B630019K06Rik	6.837221045	6.923361963	7.338065499	4291	Dnase2a	4.386853059	4.107197371	3.300753911	6591	Gucy1b3	9.983030853	9.953922561	10.11955856	8891	Mrps35	7.122180653	6.533576445	6.83806311411191	Ptpn11	9.695282173	9.63852625	9.629210899	13491	Stau2	8.69577612	8.734963251	8.931317125	15791	Zfp54	2.653344944	3.698554903	1.562791923
1992	B830017H08Rik	4.774683363	4.422799023	4.681575863	4292	Dnd1	5.16666827	4.681839139	5.37339727	6592	Gucy2c	1.644926699	1.45755574	2.572439689	8892	Mrps36	6.635767828	6.817119933	6.95781985411192	Ptpn12	7.765425478	7.244039979	7.397684671	13492	Stbd1	1.077241914	1.434390235	1.983546534	15792	Zfp541	1.086855672	3.054051951	0.977795285
1993	B930025P03Rik	3.769597192	4.038156608	2.561314687	4293	Dner	9.939671412	9.509681921	9.764654283	6593	Gucy2e	4.406813325	4.748596539	5.151990497	8893	Mrps5	7.597882444	7.842982556	8.00803263111193	Ptpn13	4.887764817	4.636565276	5.110194602	13493	Stc1	6.029737751	5.252547165	5.761733765	15793	Zfp551	5.696642053	5.992343569	6.190620811
1994	B930041F14Rik	7.260422542	7.264228223	7.609917514	4294	Dnlz	8.47630163	8.397291383	8.31095304	6594	Gucy2f	1.634686178	1.480169467	1.572374573	8894	Mrps6	8.015711668	7.701662435	7.75479910511194	Ptpn14	6.611550765	6.051290358	5.563861219	13494	Steap1	4.985150776	5.888372765	3.292369686	15794	Zfp553	6.830887587	6.191598638	6.788350324
1995	B930059L03Rik	3.394368609	3.772914154	3.44159154	4295	Dnm1	10.41812869	10.47466854	10.44250675	6595	Gucy2g	3.658550588	3.427068076	4.153664794	8895	Mrps7	7.035410907	7.308081672	7.20897033911195	Ptpn18	3.890472254	4.307792593	3.297803089	13495	Steap2	3.414968217	4.7715775	4.505949488	15795	Zfp558	2.39398419	2.869244745	1.563071527
1996	B9d1	6.283729835	6.093558105	5.652016031	4296	Dnm1l	9.925816175	9.764087339	9.787344073	6596	Guf1	5.891068637	6.266822705	6.320330999	8896	Mrps9	7.786457132	7.39061208	7.67366817111196	Ptpn2	5.944211007	6.48290692	6.455808358	13496	Steap3	3.675572012	4.494294564	4.566858853	15796	Zfp560	4.413845277	5.237340141	5.441065958
1997	B9d2	6.190319995	6.394393891	6.001492649	4297	Dnm2	7.88165151	7.94132843	7.850264423	6597	Guk1	10.16654446	10.14868402	10.07934194	8897	Mrrf	7.044616003	6.948091189	6.94598918611197	Ptpn21	4.83023957	4.800936797	4.735746059	13497	Stil	2.388351646	2.879192957	1.981761907	15797	Zfp563	6.556803998	6.771472761	6.735165804
1998	Baalc	10.39456	10.25542482	10.42811446	4298	Dnm3	10.21394689	10.22434208	10.78015859	6598	Gulo	1.07190372	0.846734838	1.568187554	8898	Mrs2	3.543840196	3.641529087	4.23023335711198	Ptpn23	6.304259788	6.273122794	6.046594432	13498	Stim1	7.926029117	7.802450655	8.242445184	15798	Zfp566	3.677345659	4.366809334	4.227971638

1999	Baat	0.03770152	1.468542101	0.985113128
2000	Babam1	8.285351961	8.44965216	8.355039152
2001	Bace1	8.987050137	8.901524119	8.862639708
2002	Bace2	4.653463463	3.923089974	3.678869766
2003	Bach1	6.820729692	6.840590327	6.923028783
2004	Bach2	6.488140323	6.228182262	6.389561567
2005	Bach2os	1.052474708	2.229424227	1.572392382
2006	Bad	8.506814926	8.633092673	8.568755461
2007	Bag1	9.284984369	9.23664331	9.085815777
2008	Bag2	4.255258071	5.06544175	4.888524271
2009	Bag3	5.131303699	5.822846643	5.534602342
2010	Bag4	9.149158137	8.958788562	9.124672287
2011	Bag5	8.017700671	8.085411369	8.435626987
2012	Bag6	8.67279672	8.75761718	8.724913084
2013	Bahcc1	4.170770441	4.296620376	3.978301609
2014	Bahd1	6.470572817	6.157516185	6.28383409
2015	Baiap2	9.754722899	9.948290039	9.741538387
2016	Baiap2l1	4.713076662	5.524146352	3.297462909
2017	Baiap2l2	3.522277383	2.848956993	3.152739487
2018	Baiap3	7.766587301	7.799050867	7.844388078
2019	Bak1	4.891926309	5.686352068	5.228317627
2020	Bambi	5.337093552	5.812413552	5.622849539
2021	Bambi-ps1	3.867152813	3.320525552	3.152139185
2022	Banf1	9.074793996	9.16941458	8.965786867
2023	Bank1	3.66959751	4.672480804	4.506385182
2024	Banp	8.467748689	8.108065242	7.979684002
2025	Bap1	7.82768841	7.879257471	7.66652256
2026	Bard1	3.059824803	2.170941375	1.980635522
2027	Barhl1	3.086146447	2.610281797	3.301703909
2028	Barhl2	1.699612625	2.830181171	3.79096874
2029	Barx2	3.085037899	3.548098599	3.98316356
2030	Basp1	13.76028663	13.72051364	13.62191392
2031	Batf3	2.666335294	2.84263156	3.442807248
2032	Bax	4.4842522	5.337271104	5.440755871
2033	Baz1a	5.657645262	5.492384553	4.368735325
2034	Baz1b	10.15731351	10.02373335	10.0714086
2035	Baz2a	8.124788702	8.205130182	8.154983553
2036	Baz2b	9.170996468	9.117766837	9.445619381
2037	BB031773	2.072582579	1.843981109	3.44629025
2038	BB557941	2.864999679	0.819355177	2.30551594
2039	Bbc3	5.244978474	5.469123767	5.024872507
2040	Bbip1	9.933616539	9.875683426	9.738194642
2041	Bbox1	4.774776176	3.903976521	4.788904844
2042	Bbs1	6.103552541	6.044711914	6.625056165
2043	Bbs10	3.991455358	4.550939709	4.737235675
2044	Bbs12	4.334408769	4.697778092	4.787985721
2045	Bbs2	6.166050245	6.048311909	6.284568353
2046	Bbs4	9.062544677	8.747888289	8.655892562
2047	Bbs5	4.184380089	5.372921438	5.190246026
2048	Bbs7	5.208062226	5.004658868	5.024072334

4299	Dnm3os	4.839689873	4.681918202	5.024139785
4300	Dnmbp	4.667232192	5.279940356	4.934904003
4301	Dnmt1	7.603299397	7.467115704	7.781335492
4302	Dnmt3a	8.969070512	8.895873889	8.977226186
4303	Dnmt3b	1.638246045	1.894915437	1.571457145
4304	Dnmt3l	1.040688343	2.247216535	0.989763447
4305	Dnpep	7.250685535	7.194224453	7.456013367
4306	Dnph1	4.654074189	3.830372209	4.790630991
4307	Dntt	1.632745305	1.484430298	0.987306091
4308	Dnttip1	6.618571951	6.342353274	6.748744922
4309	Dnttip2	8.560315913	8.616314978	8.475773541
4310	Doc2a	7.437797189	7.345035302	7.557676155
4311	Doc2b	7.090268748	6.957579885	6.486820854
4312	Doc2g	2.663093335	4.056797248	2.788503579
4313	Dock1	6.874802865	6.937185262	6.787193016
4314	Dock10	7.782235203	8.282941295	7.996759434
4315	Dock11	5.691375773	5.624802617	5.439487262
4316	Dock2	3.784516377	4.009724825	4.441608891
4317	Dock3	8.702627073	8.692611902	8.931434232
4318	Dock4	7.364590651	7.005252598	7.256254431
4319	Dock5	5.633644384	5.356333423	5.33681938
4320	Dock6	4.550492917	5.336361452	4.885923702
4321	Dock7	6.036116079	5.830156553	6.228138737
4322	Dock8	3.251128145	3.428230606	3.440196998
4323	Dock9	8.415827386	8.567025263	8.0724292
4324	Dohh	9.005147415	9.117250167	8.925430526
4325	Dok1	3.532000003	3.554128674	0.963626305
4326	Dok2	0.021970933	1.493104806	1.989547893
4327	Dok3	3.883805115	3.279474289	3.981422297
4328	Dok4	9.220019649	9.009466693	9.283823629
4329	Dok5	6.571648221	6.524863582	6.503564159
4330	Dok6	3.768540219	3.554744365	3.683096437
4331	Dok7	1.669384467	1.834345018	3.687368371
4332	Dolk	6.635802416	6.840161315	6.694207657
4333	Dolpp1	5.085893096	5.57012403	5.148879123
4334	Donson	5.039671282	5.329436825	5.406510723
4335	Dopey1	8.580486803	8.544809452	8.819357412
4336	Dopey2	7.461704969	7.205554554	7.199323405
4337	Dos	10.05765314	9.956853161	9.897346553
4338	Dot1l	6.523053373	6.832915793	6.372025129
4339	Dpagt1	5.634052544	5.749767623	5.503350217
4340	Dpcd	9.240437924	9.361887378	9.144836704
4341	Dpf1	8.851252263	8.813514374	8.616620054
4342	Dpf2	6.606787672	6.978699129	6.934261435
4343	Dpf3	4.72186455	4.699806994	4.438752145
4344	Dph1	5.904627624	5.758028518	4.836139106
4345	Dph2	4.537710372	4.756201592	4.624764831
4346	Dph3	7.177549022	7.066984894	7.078082414
4347	Dph5	5.782133768	5.90482435	5.886938226
4348	Dph6	7.375254265	7.475564769	7.479981292

6599	Gulp1	5.079907772	5.271026807	5.407629877
6600	Gusb	4.538263823	3.903003312	4.839636698
6601	Gvin1	4.776549039	4.479859907	4.839296011
6602	Gxylt1	7.102295009	7.143389996	7.237774379
6603	Gxylt2	2.048171711	1.898349386	0.986178436
6604	Gyg	6.848405162	6.905481168	6.991589793
6605	Gyk	7.548776244	7.715053084	7.60928347
6606	Gykl1	1.636444507	2.720327129	0.986187795
6607	Gypc	1.08996445	2.44759568	0.976974492
6608	Gys1	5.778241673	5.74916783	5.813654373
6609	Gzf1	7.587608094	7.459758827	7.439369033
6610	Gzma	0.013880015	0.921262835	0.990437174
6611	Gzmm	1.085350673	2.453488392	2.305266804
6612	H13	8.194008137	8.252478697	8.124639889
6613	H19	2.891598811	3.770490265	1.55472139
6614	H1f0	9.031676036	9.162955099	8.916588539
6615	H1fx	7.298579069	7.426613009	7.098842609
6616	H2-Aa	4.660118184	5.601607602	4.504188076
6617	H2-Ab1	3.254975018	3.755844504	4.070251489
6618	H2afj	8.591419563	8.543074935	8.288261086
6619	H2afv	8.825595646	8.884901477	8.980042513
6620	H2afx	7.080236802	7.250254433	7.256511291
6621	H2afy	9.299662439	9.25138106	9.288334193
6622	H2afy2	5.667920842	6.072130704	6.024944425
6623	H2afz	9.958591204	9.985145587	9.954403242
6624	H2-D1	7.6752769	8.094963486	8.385394419
6625	H2-DMa	6.228152373	6.400037875	6.18985199
6626	H2-DMb1	2.890088098	3.309285974	2.297782634
6627	H2-Eb1	4.080167587	4.611612516	3.562227167
6628	H2-K1	7.391052761	7.341507885	6.206776854
6629	H2-K2	1.08385921	2.455403035	0.978584443
6630	H2-Ke6	6.860253436	6.54968386	6.9918862
6631	H2-M3	2.656502526	3.037414985	3.686528269
6632	H2-Q10	3.408012778	0.605499524	1.548303581
6633	H2-Q9	2.396538526	2.434684365	3.154471741
6634	H2-T10	3.550245724	4.374293446	5.194055507
6635	H2-T22	5.668980728	6.030208643	5.76082127
6636	H2-T23	2.082548442	2.861746119	1.5616168
6637	H2-T24	5.935657409	5.840944821	5.336141065
6638	H2-T9	5.576477276	5.969618608	5.707381624
6639	H3f3a	8.438222859	8.603610067	8.431163379
6640	H3f3b	10.10868886	10.4684679	10.26074741
6641	H6pd	4.975113713	3.820226443	3.784916044
6642	Haao	1.639938772	0.876149871	1.571020493
6643	Habp2	1.062709923	1.461275751	0.984101598
6644	Habp4	10.07923863	10.12317828	10.25730599
6645	Hacd1	6.077332498	6.111465092	5.404762215
6646	Hacd2	8.380603304	8.599289337	8.602027295
6647	Hacd3	10.34148112	10.30229283	10.30077441
6648	Hacd4	4.239503802	3.644573098	4.507249881

8899	Mrto4	6.795575939	7.27838255	6.85028142111199
8900	Mrvi1	3.667738136	2.789645236	3.88742600211200
8901	Ms4a5	0.013867083	1.926114077	0.99044045511201
8902	Ms4a6b	2.40987339	2.640078448	2.98115524211202
8903	Ms4a6c	3.386952949	2.416194004	3.3053688911203
8904	Ms4a6d	3.077110895	3.011162478	3.44193112911204
8905	Ms4a7	2.864063222	3.484179538	1.56601764911205
8906	Msantd1	3.525820588	2.129034905	3.30442657811206
8907	Msantd2	4.784466381	4.878779811	5.47367832411207
8908	Msantd3	7.000147801	7.26659129	6.97976960811208
8909	Msantd4	9.119337848	8.733977424	9.12722895111209
8910	Msc	4.077702457	4.377123732	3.88662604811210
8911	Msh2	7.588997594	7.486496483	7.838154111211
8912	Msh3	7.521931089	7.280648627	7.25605123311212
8913	Msh6	7.588010785	7.495600437	7.54224206611213
8914	Msi2	9.818947072	10.00395201	10.2315350811214
8915	Msl1	10.43254065	10.41534362	10.3999521511215
8916	Msl2	8.646431272	8.69332989	8.72492528411216
8917	Msl3	7.562054501	7.347294286	7.32889758611217
8918	Msl3l2	6.207574176	6.067777022	5.76051902411218
8919	Msln	1.054684984	0.885138652	0.98617293811219
8920	Msmo1	9.71876822	9.567370594	9.50742450511220
8921	Msn	7.092172694	7.299445877	6.8863742111221
8922	Msr1	1.661963769	1.419238305	0.97816804611222
8923	Msra	7.57187177	7.580405366	7.39769751511223
8924	Msrb1	7.546657797	7.519809588	7.26529236311224
8925	Msrb2	7.555790494	7.679377093	7.25574714611225
8926	Msrb3	5.659123238	5.120003557	5.02357245311226
8927	Mss51	1.070251741	1.447310398	2.30776511311227
8928	Mst1r	1.066633203	2.477894883	0.98308480711228
8929	Msto1	5.598798804	5.26481019	5.22786708311229
8930	Msx1	6.124115849	6.853521135	5.14656378711230
8931	Msx1os	6.166894636	6.841610465	5.18621168411231
8932	Msx2	1.659322072	2.686488626	0.97902321111232
8933	Mt1	12.56516734	12.46296947	12.127152411233
8934	Mt2	11.77625892	11.65345014	11.3169885911234
8935	Mt3	9.678422283	9.739250567	9.25129305111235
8936	Mta1	8.178253569	8.307102048	8.08849350711236
8937	Mta2	6.899563216	6.656028051	6.72134828311237
8938	Mta3	5.369422347	5.955356567	6.00364831611238
8939	Mtag2	9.751369966	9.991788591	9.86721325711239
8940	Mtap	6.188601504	5.632172298	6.50449563411240
8941	Mtbp	3.534990997	2.81586141	4.15374481311241
8942	Mtch1	11.25729711	11.17435102	11.0016327911242
8943	Mtch2	9.48775814	9.508694789	9.38296307711243
8944	Mtcl1	6.902294126	7.085663494	7.5582135611244
8945	Mtcp1	0.055192693	3.069892856	2.30748272711245
8946	Mtdh	10.16268256	10.10335467	10.2100265311246
8947	Mterf1a	1.726323708	4.101265891	4.3754056411247
8948	Mterf1b	2.423599033	3.762987401	3.30205326311248

Ptpn3	6.385979112	6.932115462	6.579989257
Ptpn4	7.180753624	7.21800408	7.800606228
Ptpn5	8.608223481	8.686591131	9.010906395
Ptpn6	3.257073755	3.848884941	3.888478313
Ptpn7	1.054639012	1.476281615	0.986184773
Ptpn9	6.404246171	6.50900939	6.609427067
Ptpra	9.838370068	9.714728619	9.503503831
Ptprb	7.490779133	7.592346933	7.813134945
Ptprc	4.398191761	4.159087782	4.149796894
Ptprd	10.59239429	10.63622902	10.82628981
Ptpre	8.414927347	8.247851321	8.199184654
Ptprf	8.555549753	8.404216258	8.538570105
Ptprg	8.797349532	8.492194055	8.499592674
Ptprh	2.877190103	1.814838468	3.570250006
Ptprj	6.836051121	6.903889763	6.825306016
Ptprk	7.167347236	6.986143796	7.440050925
Ptprm	7.367469109	7.579017802	7.800438988
Ptprn	9.973431614	9.758348238	9.583559824
Ptprn2	9.451002786	9.331673858	9.324275585
Ptpro	6.886017693	6.591116179	6.564679238
Ptprr	6.847573792	6.617343997	6.838228208
Ptprs	9.344513226	9.254099995	9.021575194
Ptprt	9.052054182	8.842486644	9.162458849
Ptpru	7.141340052	6.593638797	6.207916143
Ptprv	1.632741999	1.48443755	0.987307085
Ptprz1	9.986043874	9.936871197	10.22796703
Ptrf	7.957038613	7.778688737	7.996850073
Ptrh1	3.53405146	3.860428507	2.978516724
Ptrh2	7.069482569	7.266450873	7.274924361
Ptrhd1	7.664923037	7.413438557	7.422708987
Pts	6.575216123	6.999678843	6.968862976
Pttg1	9.280539584	9.543902281	9.598452556
Pttg1ip	8.638722591	8.648947972	8.568704226
Ptx3	2.060819335	2.200985087	0.98170835
Ptx4	1.63275884	0.894749239	0.987302019
Puf60	9.652158052	9.619369085	9.57057365
Pum1	9.709745993	9.567295578	9.650657619
Pum2	10.09323029	10.07611061	10.16487209
Pura	11.20192377	11.0234106	11.00305378
Purb	12.55429266	12.5084174	12.56517689
Purg	7.587244676	7.41526267	7.503687587
Pus1	4.938892938	4.833499009	5.2665796
Pus10	4.829607356	4.849517633	4.981988381
Pus3	6.720888563	6.312327083	6.283530614
Pus7	6.538693585	6.208519454	6.33733481
Pus7l	3.78163301	4.096158513	4.229834936
Pusl1	5.700664471	6.301471244	6.024129269
Pvalb	9.212758047	8.962783177	9.64189502
Pvr	4.173679103	4.597587473	4.624588792
Pvrl1	7.343255442	7.080198132	6.734663747

13499	Stim2	9.477278101	9.714958941	9.387182647
13500	Stip1	9.207190204	9.238988543	9.157394146
13501	Stk10	4.538445977	4.75478709	4.371900909
13502	Stk11	9.994676861	9.83647422	9.793756842
13503	Stk11ip	5.468887372	5.966215665	5.108873075
13504	Stk16	11.31058815	11.33819275	11.34958639
13505	Stk17b	5.801710587	5.262258056	5.535346359
13506	Stk19	7.924317926	7.544373249	7.549743839
13507	Stk24	6.986694126	7.009519655	6.874755146
13508	Stk25	8.204368994	8.390613599	7.898579428
13509	Stk26	2.686666601	3.537974312	4.152723629
13510	Stk3	7.344401749	6.847199876	7.013510993
13511	Stk32a	4.39448039	3.832245439	3.886469276
13512	Stk32b	4.154851457	3.54178888	2.783428537
13513	Stk32c	9.103159167	9.170863652	9.555711746
13514	Stk33	4.539626393	5.651578768	5.02645157
13515	Stk35	5.403428264	5.893299817	5.47185865
13516	Stk36	3.980248764	3.937380641	3.300196716
13517	Stk38	7.056841962	6.836197685	6.54897507
13518	Stk38l	6.526225393	6.93795681	6.934444283
13519	Stk39	9.32644887	9.636004426	9.519157197
13520	Stk4	8.871438159	8.899208762	8.834896108
13521	Stk40	7.899362799	7.61216608	7.623800191
13522	Stmn1	11.18453392	11.23068464	11.29117473
13523	Stmn1-rs1	1.655206386	2.193512375	1.983563234
13524	Stmn2	11.74451061	11.62817698	11.49616159
13525	Stmn3	11.63374721	11.64432147	11.68992795
13526	Stmn4	10.3117639	10.38007367	10.29286723
13527	Stmnd1	3.234960174	3.578474253	2.565759295
13528	Stom	5.085358987	5.484673193	5.472179853
13529	Stoml1	7.924605318	7.917274002	7.774554271
13530	Stoml2	7.588016241	7.517114594	7.526869533
13531	Ston1	3.072102259	2.644234646	3.892176209
13532	Ston2	7.46339786	7.526717956	6.979336496
13533	Stox2	10.31903249	10.27432389	10.5790188
13534	Stpg1	3.649778049	3.676307085	2.98086444
13535	Stpg2	0.013869475	1.926110041	0.990439848
13536	Stra13	6.805659926	7.076291104	6.188573734
13537	Stra6	3.404027174	4.630784418	3.148079442
13538	Strada	5.438413767	5.421672459	5.337079843
13539	Stradb	6.997504017	6.81576172	6.838099008
13540	Strap	9.220237337	9.186179446	9.301927674
13541	Strbp	11.9405331	11.97571639	12.14615437
13542	Strc	8.669771154	8.915104594	8.904849833
13543	Strip1	8.745110128	8.776119269	8.659586375
13544	Strip2	6.250391484	6.742346045	5.933261787
13545	Strn	5.860606599	5.615218978	6.025198076
13546	Strn3	10.5592707	10.37188565	10.55269819
13547	Strn4	9.113490946	9.185600768	8.968695786
13548	Stt3a	6.076168115	5.503930419	6.025294185

15799	Zfp568	4.317499827	4.318307259	3.78764308
15800	Zfp57	3.884016651	4.875925161	3.438049239
15801	Zfp574	6.507673546	6.710336683	6.405784819
15802	Zfp575	6.02908021	5.844208996	5.935279589
15803	Zfp579	5.118855417	5.172901767	4.787491656
15804	Zfp58	4.599040664	4.85551878	4.149058914
15805	Zfp580	6.753412407	7.219356635	6.734727216
15806	Zfp583	5.689365863	5.263491486	5.227702082
15807	Zfp59	5.116972132	4.534513501	4.93543223
15808	Zfp592	6.014910205	6.556809278	6.319839004
15809	Zfp593	6.244281512	5.885839368	5.623437599
15810	Zfp595	3.988193917	4.307389845	4.567329918
15811	Zfp597	6.724725179	6.589680967	6.812995317
15812	Zfp598	6.523527	6.580313589	7.014490614
15813	Zfp599	5.806032356	6.154319369	5.336461065
15814	Zfp60	7.068240989	6.626804557	7.284405282
15815	Zfp605	6.73757273	6.564851825	6.652504705
15816	Zfp606	6.17280236	6.522628788	6.623868523
15817	Zfp607	0.081554219	0.804612373	2.791831738
15818	Zfp608	8.097918719	8.405279921	8.114296945
15819	Zfp609	8.280167886	8.305753027	8.292896396
15820	Zfp61	5.433753942	5.043806036	5.023858959
15821	Zfp612	8.178744649	8.050793439	8.464045729
15822	Zfp617	8.716832396	8.677451596	8.670031158
15823	Zfp618	4.714119718	4.765966464	4.228362115
15824	Zfp619	3.66412248	3.937938434	3.564762252
15825	Zfp62	7.012270782	7.055826301	7.088421578
15826	Zfp622	8.724204542	8.760280014	8.714738531
15827	Zfp623	5.908838387	5.537394218	5.264243628
15828	Zfp626	6.83398424	6.73366808	7.003056237
15829	Zfp628	6.076315501	5.972738079	6.110392513
15830	Zfp629	6.189248378	6.380712577	6.565889619
15831	Zfp637	6.555647436	6.510591546	6.406028943
15832	Zfp638	10.04905031	9.753215896	9.912194971
15833	Zfp639	7.51545092	7.619901667	7.701292448
15834	Zfp64	4.605724627	4.412344844	4.787744534
15835	Zfp641	1.693331841	2.6372502	3.568451029
15836	Zfp644	10.71357719	10.52396983	10.81051534
15837	Zfp646	4.596618137	4.431558935	5.027806971
15838	Zfp647	2.66519966	2.41154076	3.792274542
15839	Zfp65	6.720252363	6.280081088	6.189097586
15840	Zfp651	8.607213745	8.478743068	8.402066221
15841	Zfp652	6.145813518	6.006077717	6.320517839
15842	Zfp652os	6.433976991	6.161850401	5.65104938
15843	Zfp653	5.039578674	5.425505586	5.709225557
15844	Zfp654	6.387280677	6.386879267	6.980643945
15845	Zfp655	6.986812418	7.009291992	7.256772469
15846	Zfp658	4.647032435	3.751594783	3.147545509
15847	Zfp661	5.954599706	5.262176162	4.932562486
15848	Zfp664	9.173168652	9.127920998	9.149932423

2049	Bbs9	6.00877932	5.970954097	5.564090737
2050	Bbx	8.61577782	8.667987717	8.663031925
2051	BC002163	9.237818131	9.173104575	9.11179133
2052	BC003331	8.548065673	8.65247153	8.526885659
2053	BC003965	7.731374219	7.751372237	7.666452929
2054	BC004004	8.472666313	8.532118101	8.255956707
2055	BC005537	8.433564023	8.532267333	8.670222749
2056	BC005561	8.848696206	8.647338023	8.83797029
2057	BC005624	7.588644993	7.612340019	7.761518215
2058	BC006965	4.648553848	3.280064629	3.680477525
2059	BC016579	0.035117242	0.880607525	2.310065004
2060	BC017158	5.905882289	5.827635855	4.835772687
2061	BC017643	6.709668357	6.793943736	6.666445737
2062	BC018473	3.266834771	3.73512609	4.150794522
2063	BC020402	2.394002506	2.670136441	2.791022734
2064	BC021891	4.156513908	4.390679513	3.888527608
2065	BC022687	4.936335128	4.277001612	5.068502051
2066	BC023829	3.057495687	2.449475724	2.568831071
2067	BC024139	2.852334234	2.485659553	1.986623082
2068	BC024386	0.032521724	1.476264934	1.571917047
2069	BC024978	4.657485804	3.507313846	4.440461526
2070	BC025920	4.470832108	4.655203772	4.504183651
2071	BC026585	1.052463489	0.890008648	0.986744491
2072	BC027231	6.345206184	6.365832643	6.406927132
2073	BC028528	0.029925702	0.889906005	2.31079981
2074	BC029214	6.554684879	6.483326079	6.128982389
2075	BC029722	8.623172198	8.562735069	8.583679682
2076	BC030336	8.477397057	8.559042455	8.587353831
2077	BC030499	3.412788418	3.836182119	4.303893082
2078	BC030500	4.934008963	4.413172912	4.564457911
2079	BC030867	2.074590545	2.675583214	1.564046402
2080	BC031181	9.736946346	9.755333525	9.719768269
2081	BC031361	3.780878051	4.016658084	4.30431072
2082	BC037032	1.634647265	0.889906296	1.572384595
2083	BC037034	7.963068921	7.71212919	8.083445745
2084	BC037704	1.066578394	2.705418046	1.569330841
2085	BC039966	2.146159001	2.5549259	5.112838486
2086	BC048403	3.420547542	4.158121652	4.625727793
2087	BC048507	3.058206861	3.207114418	1.981226748
2088	BC048546	7.598406714	6.743075805	6.774259655
2089	BC048609	4.784875228	5.435489929	5.473589029
2090	BC049352	3.061216347	2.670194018	2.567724214
2091	BC049762	0.040217583	2.909005053	2.309374131
2092	BC051019	3.523321097	2.846254965	3.792401216
2093	BC051142	3.888477942	4.914494485	3.886713517
2094	BC051226	6.325756653	6.161362285	6.1897266
2095	BC052040	5.169700485	5.601876651	5.681859235
2096	BC055324	4.24522074	3.734760202	3.786376513
2097	BC064078	4.243911739	3.2687325	4.567860256
2098	BC065397	3.985821185	1.698718866	3.786720815

4349	Dph7	5.404364524	5.799102102	5.624172411
4350	Dpm1	7.848735213	7.854713512	7.78726287
4351	Dpm1-adnp	9.509026903	9.522430776	9.670029334
4352	Dpm2	7.773691193	7.889512539	7.623719631
4353	Dpm3	9.721121702	9.958161758	9.909238216
4354	Dpp10	8.76666344	8.307252477	9.295587409
4355	Dpp3	6.573699711	7.185328943	7.046793499
4356	Dpp4	3.404727302	4.256448391	4.070035346
4357	Dpp6	10.18266839	10.3347748	10.30641845
4358	Dpp7	6.058932705	6.601917347	5.592877749
4359	Dpp8	7.951621033	8.032012088	8.14996693
4360	Dpp9	7.758064695	7.474916067	7.447607458
4361	Dpt	0.074320184	1.849112489	2.569309276
4362	Dpy19l1	9.456028705	9.709795842	9.944425282
4363	Dpy19l2	0.047778724	2.902334373	1.569346648
4364	Dpy19l3	7.278825042	7.205777224	7.056732396
4365	Dpy19l4	3.899833254	4.421313027	4.839588002
4366	Dpy30	7.868833222	7.977718837	7.886808964
4367	Dpyd	5.028137445	4.645368691	4.62377917
4368	Dpys	1.650989525	1.870194935	2.571311264
4369	Dpysl2	10.44580812	10.395161	10.52689531
4370	Dpysl3	8.880603694	8.76654836	8.44336605
4371	Dpysl4	6.324962943	6.162931058	6.266081924
4372	Dpysl5	5.834952466	6.053419019	5.761221601
4373	Dqx1	2.386341595	2.18412153	1.565658198
4374	Dr1	7.356675453	7.661700851	7.218154543
4375	Dram1	2.654135936	2.666988398	2.984072278
4376	Dram2	6.650440356	6.591572771	6.748386491
4377	Drap1	10.88134584	10.88038628	10.62471719
4378	Draxin	2.644680607	2.884523907	2.569771291
4379	Drc1	5.21088302	5.757767491	5.265343319
4380	Drc7	5.658059814	6.204161538	4.436090614
4381	Drd1	8.662976961	9.139511476	9.308780669
4382	Drd5	6.167910217	6.333423141	5.622103421
4383	Drg1	8.436323045	8.017654558	7.916492496
4384	Drg2	6.97677815	7.493398464	7.119474117
4385	Drosha	8.393250014	8.233593854	8.389419046
4386	Drp2	7.611791678	7.358645072	7.781158265
4387	Dsc2	1.062766234	1.885120532	0.984087024
4388	Dsc3	3.759790703	3.684167851	1.559107082
4389	Dscam	8.035749088	8.397280921	8.435481214
4390	Dscaml1	8.24376322	8.198184609	7.916659053
4391	Dscr3	6.291672097	6.391352861	6.624284238
4392	Dse	4.334974011	4.696735828	4.735167668
4393	Dsel	7.758417579	7.510493324	7.48777572
4394	Dsg2	5.506968805	6.020397966	5.503496488
4395	Dsn1	2.404047329	3.575977908	1.977168709
4396	Dsp	6.697522453	7.028873322	6.370956544
4397	Dst	10.92220443	10.90172488	11.14742299
4398	Dstn	11.05849616	11.10420357	10.80809054

6649	Hace1	6.753953494	6.989620357	6.898926503
6650	Hacl1	4.5390613	4.59633438	4.681262296
6651	Hadh	7.848522776	7.675662707	7.780850479
6652	Hadha	8.535890168	8.649128145	8.376448085
6653	Hadhb	7.701991622	7.509232928	7.721628795
6654	Hagh	8.152370099	8.289486551	8.174682024
6655	Haghl	7.716829141	7.52989407	7.594607392
6656	Haglr	0.019318268	0.910048994	1.990021162
6657	Hao1	3.04728859	2.475485842	0.98261259
6658	Hap1	8.529696108	7.747930141	8.083318996
6659	Hapln1	6.92607531	6.881446621	6.78703856
6660	Hapln2	5.000237264	5.950378523	5.812960513
6661	Hapln3	2.056935231	1.878982044	1.985537452
6662	Hapln4	7.356730868	7.415920484	7.666955075
6663	Harbi1	5.286757922	5.085780111	5.109372692
6664	Hars	8.406499168	8.112582485	8.246734166
6665	Hars2	5.988421147	6.124535192	6.407187805
6666	Has1	3.98091769	3.280952058	3.680572989
6667	Has2	1.657923457	3.216531882	1.982950868
6668	Has3	2.67556182	3.30453297	3.790480239
6669	Hat1	6.329225676	6.360633258	6.2834624
6670	Haus1	4.884969416	4.210597987	4.438115146
6671	Haus2	7.147219188	7.314797273	7.439783469
6672	Haus3	5.574233511	5.179893487	6.025396594
6673	Haus4	2.691513638	4.019302192	4.627756992
6674	Haus5	2.88545224	3.780002024	3.151760604
6675	Haus6	5.079096253	5.008196363	5.190220336
6676	Haus7	3.879367428	3.853122348	2.977332345
6677	Haus8	4.617187174	5.654115241	5.023138053
6678	Havcr2	2.671337217	3.441753922	3.303713368
6679	Hax1	6.833964528	6.402497344	6.518773604
6680	Hba-a1	12.68119404	12.4613404	11.97322468
6681	Hba-a2	7.177432282	6.702837396	6.065075601
6682	Hbb-b1	11.51255706	11.3123913	10.95790673
6683	Hbb-b2	9.282586238	9.157144315	8.815776893
6684	Hbb-bs	11.52555517	11.32550773	10.97638576
6685	Hbb-bt	9.372759158	9.214619314	8.883615105
6686	Hbegf	6.619812378	6.131372367	6.850846062
6687	Hbp1	8.265234454	8.431919459	8.124545118
6688	Hbs1l	7.238499314	6.740601858	7.002374465
6689	Hc	3.067287061	1.376359531	3.444117463
6690	Hcar1	1.696094718	2.83484824	2.980424779
6691	Hccs	6.939725394	7.249565118	7.464465719
6692	Hcfc1	8.835064584	8.815715065	8.893008311
6693	Hcfc1r1	9.217220673	9.206907119	9.013365051
6694	Hcfc2	5.129945101	5.597062364	5.301393377
6695	Hck	2.101702502	3.440723726	2.980054351
6696	Hcls1	4.338329064	5.206734233	4.837956539
6697	Hcn1	9.799781038	9.462743969	9.897435418
6698	Hcn2	8.465084458	8.92920453	8.91972073

8949	Mterf2	6.696161212	6.780124136	6.533973758
8950	Mterf3	5.747465812	5.361394892	5.565685541
8951	Mterf4	6.540016689	6.92128492	6.405904353
8952	Mtf1	9.474569939	9.321883988	9.315402761
8953	Mtf2	8.433854112	8.312417721	8.623970665
8954	Mtfmt	5.659338129	5.365153343	5.227879146
8955	Mtfp1	8.00424374	8.019992905	7.767861359
8956	Mtfr1	5.174669803	5.748763692	5.264144611
8957	Mtfr1l	8.086074707	8.137684737	8.104153099
8958	Mtg1	8.622994235	8.70675296	8.218164395
8959	Mtg2	5.367197737	5.9594522	5.439382664
8960	Mthfd1	6.346679129	6.156899558	6.653331322
8961	Mthfd1l	6.739561793	6.948334146	7.002717873
8962	Mthfd2	5.480925201	6.143650026	6.302616676
8963	Mthfd2l	4.081218733	3.257810603	4.150070954
8964	Mthfr	4.667316811	4.792819568	5.408689343
8965	Mthfs	5.661228312	5.543253624	5.681530661
8966	Mthfsd	5.499956002	4.683079915	4.369076997
8967	Mthfsl	6.165988116	5.786732966	6.338497756
8968	Mtif2	7.919645921	8.157611227	7.84410539
8969	Mtif3	7.750903621	7.45300385	7.527055064
8970	Mtm1	4.319073146	2.967481272	2.291355834
8971	Mtmr1	7.14545459	7.087136445	7.03534556
8972	Mtmr10	5.329374127	5.542603669	5.503969564
8973	Mtmr11	4.773191884	3.994178837	4.301766849
8974	Mtmr12	6.765240134	6.90975424	6.735244841
8975	Mtmr14	5.887745883	5.990338041	6.130499448
8976	Mtmr2	7.649444992	7.49607796	7.354804365
8977	Mtmr3	7.309344193	7.34616726	7.246671643
8978	Mtmr4	9.05973694	8.578366351	8.751489747
8979	Mtmr6	9.225353172	9.276590034	9.3331579
8980	Mtmr7	8.592557347	8.635305655	8.777733112
8981	Mtmr9	4.600672167	4.653403478	4.565627031
8982	Mto1	6.10120562	5.945936283	6.002475517
8983	Mtor	7.968878324	7.876664033	7.916661926
8984	Mtpap	6.525700202	6.732300052	6.518595713
8985	Mtpn	10.47921763	10.40960748	10.47785754
8986	Mtr	4.940738534	5.086213146	5.407522239
8987	Mtrf1	5.322278022	5.239579738	5.024760446
8988	Mtrf1l	6.145451188	5.922286653	6.002218182
8989	Mtrr	6.325792194	6.123927817	5.86191453
8990	Mtss1	8.129654684	7.881376052	7.939949331
8991	Mtss1l	9.881282146	9.752162779	9.769518764
8992	Mttp	5.23704467	4.072631075	4.065755618
8993	Mturn	9.173023358	9.157981817	9.111876403
8994	Mtus1	7.125973722	7.417275376	7.480191902
8995	Mtus2	8.62724381	8.527577442	8.831944709
8996	Mtx1	6.326742815	6.283126846	6.302268538
8997	Mtx2	7.454611305	7.408960587	7.337583698
8998	Mtx3	6.723186595	6.291181867	6.680624104

11249	Pvrl2	4.472814388	5.105908917	4.68134864
11250	Pvrl3	8.080565298	8.448085685	7.962842463
11251	Pvrl4	5.036166779	5.123235181	5.1897074
11252	Pvt1	4.466326149	4.815313386	4.30278825
11253	Pwp1	7.374937496	7.184463736	7.503812056
11254	Pwp2	5.636427234	5.86361927	5.81315512
11255	Pwwp2a	9.181107069	9.289732506	9.246837102
11256	Pwwp2b	5.914271955	6.401649615	5.593406431
11257	Pxdc1	4.545834969	4.740603772	4.437450913
11258	Pxdn	5.854868475	5.543750302	4.677489689
11259	Pxk	7.157041664	7.231819497	7.189578241
11260	Pxmp2	6.006535409	5.771883995	5.534187152
11261	Pxmp4	4.718140252	4.853960242	4.565832159
11262	Pxn	5.441743259	6.036200192	5.680398878
11263	Pxylp1	6.663239329	6.437746341	6.565266889
11264	Pycard	4.931258347	5.104677891	4.565200502
11265	Pycr2	8.167995157	8.314785939	7.968454578
11266	Pycrl	6.587824165	6.686099463	6.42264486
11267	Pygb	9.996429634	9.993927372	9.948638063
11268	Pygl	4.538003077	4.985040176	4.148510454
11269	Pygm	7.242634021	7.420962759	7.761610279
11270	Pygo1	4.177227722	4.414688336	5.475003321
11271	Pygo2	8.422991215	8.017887017	7.96863121
11272	Pyhin1	0.01929942	1.917340925	0.989112693
11273	Pyroxd1	7.046480855	7.086085662	7.013310066
11274	Pyroxd2	1.058859069	1.468425337	0.985096914
11275	Pyurf	7.499654887	7.81045577	7.787449342
11276	Pzp	3.669343122	2.785204497	4.442053301
11277	Qars	7.347131091	7.369904495	7.169552374
11278	Qdpr	11.14136525	11.50647206	11.15987108
11279	Qk	11.13860611	11.23498641	11.26539393
11280	Qpct	5.171728032	5.849583504	4.677257396
11281	Qpctl	4.882767646	4.647149181	4.839036611
11282	Qrfp	1.630819338	1.909324424	0.987884089
11283	Qrfpr	3.233289746	2.153304227	3.153792469
11284	Qrich1	6.951614151	7.115137035	6.874477256
11285	Qrsl1	6.872721249	6.684312099	6.455292704
11286	Qser1	8.057583027	7.870003781	8.150075305
11287	Qsox1	7.156200182	7.215916124	7.088666018
11288	Qsox2	5.911398543	5.885436374	5.623385787
11289	Qtrt1	5.984160223	5.588815396	5.564364315
11290	Qtrtd1	4.542577733	4.976665062	4.935241278
11291	R3hcc1	7.355617851	7.448338396	7.227949354
11292	R3hcc1l	7.135386655	7.0954823	6.979794655
11293	R3hdm1	9.349553003	9.27657332	9.51148045
11294	R3hdm2	10.00541844	10.10008295	10.265459
11295	R3hdm4	10.39097326	10.447499	10.08585283
11296	R74862	6.222948683	5.975596629	5.863372895
11297	Rab1	9.436059424	9.439340234	9.600245781
11298	Rab10	11.07487866	11.02084108	11.01958881

13549	Stt3b	8.260398579	8.131546269	8.088346178
13550	Stub1	9.650460588	9.821281023	9.687349204
13551	Stx12	9.582336726	9.700981924	9.546037561
13552	Stx16	7.650574732	7.757542517	7.721512746
13553	Stx17	7.299685527	7.043757485	7.439659017
13554	Stx18	10.9640054	10.96252722	10.96225646
13555	Stx1a	9.001630838	8.72954538	9.167630012
13556	Stx1b	7.240810375	7.120837188	7.701724409
13557	Stx2	5.882678838	5.726520254	5.336929508
13558	Stx3	6.913932681	6.99811346	7.099176425
13559	Stx4a	7.836240316	8.062454471	8.134964919
13560	Stx5a	6.925333038	6.546801575	6.837955078
13561	Stx6	7.787898284	8.126484166	7.700972496
13562	Stx7	8.960288921	8.853818752	9.177302066
13563	Stx8	7.309491931	7.57563638	7.397957598
13564	Stxbp1	11.48466916	11.53086916	11.688655
13565	Stxbp2	6.553022423	6.314981613	6.246769436
13566	Stxbp3	5.788747302	6.68849483	6.301612093
13567	Stxbp3-ps	3.541494474	3.937343901	4.152389133
13568	Stxbp4	6.404719013	6.75913493	6.735133599
13569	Stxbp5	8.407760514	8.674183462	8.793897771
13570	Stxbp5l	10.19540806	10.20015077	10.08065845
13571	Stxbp6	8.256571884	8.492122171	8.50743098
13572	Styx	6.007832539	5.560836707	5.787492351
13573	Styxl1	2.398164785	3.79615141	2.56673032
13574	Sub1	11.24293625	11.21014352	11.15801834
13575	Sucla2	9.627391189	9.563951961	9.648894867
13576	Suclg1	9.652666312	9.928104229	9.820744326
13577	Suclg2	6.294099722	6.370919327	6.980617711
13578	Suco	8.619526146	8.592907868	8.538384627
13579	Suds3	9.017340186	9.118822214	9.172338332
13580	Sufu	5.769494426	5.373315922	4.436911556
13581	Sugct	3.780218917	4.248268542	3.680086682
13582	Sugp1	6.330499213	6.888787534	6.735382286
13583	Sugp2	8.084822434	7.81509601	7.767824045
13584	Sugt1	9.063116494	8.837477383	8.97721424
13585	Sulf1	5.963472648	7.107004421	5.225143417
13586	Sulf2	8.53926978	8.529105094	8.359372989
13587	Sult1a1	5.434508199	4.959258484	4.933070178
13588	Sult1e1	1.682344304	1.808858405	3.892886794
13589	Sult2a6	0.021960484	0.904851557	1.573895136
13590	Sult2b1	3.252578958	4.396892929	3.566007488
13591	Sult4a1	11.0663815	10.98941942	11.14804911
13592	Sult5a1	3.224532494	1.846296592	1.981747255
13593	Sult6b1	1.050306014	0.89472261	1.988641579
13594	Sumf1	6.936002516	6.709135757	6.564730445
13595	Sumf2	5.695280062	5.617016312	5.564487281
13596	Sumo1	9.290097068	9.327678361	9.408386738
13597	Sumo2	7.300798339	7.594322058	7.594902826
13598	Sumo3	12.36279793	12.33837878	12.28831473

15849	Zfp667	7.375141618	7.254163763	7.346268833
15850	Zfp668	5.331099309	5.567546559	5.912074057
15851	Zfp672	6.189497506	6.544744442	5.909776398
15852	Zfp677	1.129854581	2.833461592	3.151044476
15853	Zfp68	6.781006888	6.589265998	6.594264082
15854	Zfp687	5.72628856	6.011276984	5.563920862
15855	Zfp688	5.563294558	5.009310684	5.024647491
15856	Zfp689	4.597568217	3.911969863	4.789689747
15857	Zfp69	2.372767942	1.885142621	2.795688607
15858	Zfp691	4.537756683	4.852228535	4.226981683
15859	Zfp692	5.032346926	4.788482584	5.303512166
15860	Zfp697	6.661717617	6.228715883	6.439798208
15861	Zfp7	4.395817699	4.306847243	4.067571702
15862	Zfp703	7.78768325	7.756813349	8.083696811
15863	Zfp704	8.51550287	8.760620238	8.663026067
15864	Zfp706	10.17858257	10.40916666	10.13732201
15865	Zfp707	5.208818397	5.196882833	5.228231517
15866	Zfp708	4.71866937	4.223342576	4.565695515
15867	Zfp709	4.978808949	5.105885812	3.883804885
15868	Zfp710	5.604005885	5.933835486	5.623935592
15869	Zfp711	6.366404307	6.52666094	6.748990564
15870	Zfp712	3.549607934	3.518045301	4.738075758
15871	Zfp715	7.278546733	7.206309499	6.886532932
15872	Zfp719	6.471162484	6.611905762	6.169412229
15873	Zfp72	1.062808982	0.867173416	1.986245338
15874	Zfp729a	5.806144056	5.563729647	5.594549421
15875	Zfp729b	6.537556598	6.598436929	6.504052689
15876	Zfp738	6.101226455	5.376268524	6.320852713
15877	Zfp74	5.372534752	5.861308279	6.321306697
15878	Zfp740	7.199803276	7.22999412	7.363900282
15879	Zfp746	5.251606923	5.486315746	5.149224858
15880	Zfp747	5.163862325	5.167721978	4.679397883
15881	Zfp748	5.78127828	6.092686788	6.110206883
15882	Zfp750	1.638231474	1.894943692	2.310065922
15883	Zfp758	5.434356351	4.916281214	5.372990897
15884	Zfp759	4.665763675	4.889085006	3.782495465
15885	Zfp760	5.936868727	5.791541816	6.150941959
15886	Zfp763	7.338854547	7.723324591	7.687661554
15887	Zfp764	4.725636115	4.408490598	5.441859623
15888	Zfp768	6.723322537	6.952233995	6.638264232
15889	Zfp770	8.045929635	7.924956845	8.209084889
15890	Zfp771	8.290003056	8.43886688	8.194209999
15891	Zfp772	5.404842813	5.537562089	5.981338094
15892	Zfp773	3.874814248	3.432299166	2.785083919
15893	Zfp775	6.166076572	5.762464368	5.957115674
15894	Zfp777	6.289966282	6.193354229	6.319926233
15895	Zfp78	4.539881705	4.217894083	4.226409555
15896	Zfp780b	5.940185477	5.686096921	6.109380382
15897	Zfp781	6.210861703	6.394348459	6.504074526
15898	Zfp783	4.071117353	3.140004154	3.438665296

2099	BC068281	4.981140478	4.351590863	4.788098357	4399	Dstyk	8.313550746	8.243596763	8.213654608	6699	Hcn3	4.77665616	4.479628394	4.438976548	8999	Muc15	3.784034633	3.399364759	3.56297162211299	Rab10os	5.887332153	6.18564802	6.170789595	13599	Sun1	7.472089073	7.361474933	7.527155294	15899	Zfp784	5.03097593	5.171072995	4.146613353
2100	BC089491	1.64327573	2.214284827	0.984093703	4400	Dtd1	8.569174861	8.783107402	8.922577005	6700	Hcn4	2.056887078	0.858936558	1.985549898	9000	Mug1	3.442599343	3.057379969	5.73722425211300	Rab11a	8.968683826	8.682871587	8.913787179	13600	Sun2	7.157376595	7.239857493	7.228039944	15900	Zfp786	3.786667179	4.436288936	4.683134979
2101	Bc1	7.5628522	7.280071979	7.46401489	4401	Dtd2	4.251436828	4.363529935	4.625271282	6701	Hcrt	1.653853495	2.466224008	1.567418257	9001	Mug-ps1	2.668904802	2.40312481	3.44231070311301	Rab11b	10.09310263	10.06311682	10.04203449	13601	Suox	4.723726534	4.589162677	5.069004918	15901	Zfp787	5.834311127	5.951320167	6.025238374
2102	Bc1	8.129852129	7.919129244	8.024394265	4402	Dthd1	1.075461875	2.195921915	2.98708365	6702	Hcrtr1	1.676112473	2.431089409	3.68652277	9002	Mul1	7.092663124	7.230459749	7.10908765411302	Rab11fip1	4.387511398	3.75215514	3.788478501	13602	Supt16	9.822050866	9.786049142	9.977216051	15902	Zfp788	6.573240884	6.550067599	7.057653141
2103	BC100451	1.07553313	2.466184169	2.570775621	4403	Dtl	4.766251618	4.01136785	4.151038835	6703	Hcrtr2	3.768902243	3.160196407	2.979108493	9003	Mum1	6.952043956	7.258070568	7.21862748711303	Rab11fip2	9.299721415	9.351785375	9.418840742	13603	Supt20	4.934383233	4.348400373	5.025741298	15903	Zfp790	6.587060503	6.22523985	6.455706342
2104	Bcam	3.677460549	4.992051856	3.978766806	4404	Dtna	10.75296158	10.75823826	10.63549673	6704	Hcst	2.071467578	2.179692937	1.981770434	9004	Mum1l1	5.209789995	5.683901186	4.73281014711304	Rab11fip3	8.318849559	8.284869842	8.495878994	13604	Supt3	6.451245947	5.964707324	6.320551172	15904	Zfp791	3.658865728	3.151789469	3.982998172
2105	Bcan	9.738828612	9.68775409	9.788955631	4405	Dtnb	6.101316248	6.182504871	5.934066179	6705	Hdac1	6.975707937	7.301362643	7.024395825	9005	Mup1	5.248786806	1.049701041	4.14492443411305	Rab11fip4	7.046823083	6.786979681	7.389875818	13605	Supt4a	8.336868146	8.269772086	8.174834815	15905	Zfp799	6.268245663	6.589690208	5.97966498
2106	Bcap29	8.06377613	8.051628764	8.398132362	4406	Dtnbp1	8.163117	8.238554603	8.359463203	6706	Hdac10	3.980975551	3.533026393	3.299971854	9006	Mup10	5.747092009	2.875544209	4.14398749811306	Rab11fip5	7.723888247	7.530139199	8.008386256	13606	Supt5	8.156925438	8.235926126	8.194452995	15906	Zfp800	8.260677727	8.341885342	8.306409828
2107	Bcap31	8.780505016	8.658196927	8.590979756	4407	Dtwd1	6.18911459	6.412300347	6.265591573	6707	Hdac11	9.140700456	8.895632773	8.96299947	9007	Mup11	5.660830298	1.533408987	3.55461562311307	Rab12	9.988734077	9.775739875	9.844171459	13607	Supt6	9.605960352	9.70101268	9.574324787	15907	Zfp804a	8.481150264	8.590653251	8.350622182
2108	Bcar1	7.664772922	7.566032529	7.354730826	4408	Dtwd2	4.533850548	4.713177572	4.504912468	6708	Hdac2	9.19706186	9.447746972	9.179609938	9008	Mup12	4.837164689	2.714989518	4.14596720411308	Rab13	5.405620847	5.645985185	5.503148707	13608	Supt7l	6.582045152	5.986968831	5.760540258	15908	Zfp804b	4.464870747	4.087680842	4.373863422
2109	Bcar3	4.677826723	5.261097728	5.439711727	4409	Dtx1	7.419912445	7.438907393	7.534874778	6709	Hdac3	6.708050406	6.46616153	6.487703801	9009	Mup13	5.329085335	2.470099946	4.43509518911309	Rab14	11.84293376	11.77074412	11.82743472	13609	Supv3l1	6.306759392	6.475511849	5.469762195	15909	Zfp808	2.096541362	2.839737558	3.442519315
2110	Bcas1	11.32616	11.65705631	11.29681712	4410	Dtx2	5.204994485	4.925809764	5.150880939	6710	Hdac4	7.156210924	6.83503972	7.140107273	9010	Mup14	5.693795125	2.859863317	4.56037489211310	Rab15	10.03576994	9.999682777	9.767893757	13610	Surf1	8.230266554	8.260207453	8.203969567	15910	Zfp809	6.961312245	6.694880779	6.787428915
2111	Bcas1os2	5.2501084	5.11982244	5.108794027	4411	Dtx3	8.332869505	8.305894564	8.174667709	6711	Hdac5	8.878378901	8.949044948	9.062217789	9011	Mup15	7.088921537	2.79863052	5.22294599811311	Rab18	9.377609462	9.403739779	9.50161421	13611	Surf2	7.925373605	7.894072301	8.002609867	15911	Zfp81	4.25679899	4.065472851	5.475217293
2112	Bcas2	7.980783444	7.761701296	7.963112399	4412	Dtx3l	4.886598409	4.791230453	4.838075334	6712	Hdac6	6.249675422	6.14071257	6.246958647	9012	Mup16	4.83088579	1.143208267	3.55922049211312	Rab1b	8.075028502	8.244167637	7.886628374	13612	Surf4	8.485128108	8.584410843	8.341933852	15912	Zfp810	5.59848478	5.489464133	4.785482247
2113	Bcas3	7.418406727	7.222683098	7.256317535	4413	Dtx4	7.634414586	7.659662106	7.519149487	6713	Hdac7	7.71705416	7.943656612	7.666446787	9013	Mup17	4.66014855	0.470897634	2.97109500911313	Rab21	8.245514094	8.255675092	8.402192081	13613	Surf6	6.165289075	6.108491261	5.934156897	15913	Zfp811	6.034909268	5.900347381	6.129973291
2114	Bcat1	7.943902392	7.732104629	7.630826846	4414	Dtymk	6.735875788	6.581952196	6.422874962	6714	Hdac8	6.082491862	6.586118293	6.088057879	9014	Mup19	4.982467276	0.39378498	4.06427282511314	Rab22a	6.310074727	6.739567849	6.653101597	13614	Susd1	0.040267657	0.871638698	2.573051825	15914	Zfp82	4.171308559	4.295567381	4.625115091
2115	Bcat2	4.314587177	3.848964265	4.069852345	4415	Dus1l	5.572753049	5.448312946	5.503212702	6715	Hdac9	7.347277126	7.361703937	7.542556347	9015	Mup2	7.155877141	3.809243509	5.9315058811315	Rab23	6.166381367	5.922674655	5.679280841	13615	Susd2	3.795795144	4.481918229	5.305146707	15915	Zfp820	3.385240455	2.421133012	3.153189986
2116	Bccip	8.271039078	8.422684376	8.283620923	4416	Dus2	4.895513832	5.515238461	5.504149002	6716	Hdc	5.167978519	6.090344722	4.22329423	9016	Mup20	4.827372406	2.011915511	1.96024338211316	Rab24	9.318895924	9.355779431	9.232620773	13616	Susd3	5.110044296	3.724616858	3.435077686	15916	Zfp821	6.807396551	6.657405463	6.957843002
2117	Bcdin3d	5.286976018	4.962691905	5.067327225	4417	Dus3l	5.864061373	6.33638848	6.046120785	6717	Hddc2	5.209009249	5.232354843	5.109070233	9017	Mup21	2.079713757	0.783881374	3.44516201811317	Rab26	6.48566655	6.533326192	6.129816063	13617	Susd4	8.15079609	8.068216269	8.040851732	15917	Zfp825	5.074066447	4.976493767	4.93521996
2118	Bche	5.530312946	5.240777601	4.437282153	4418	Dus4l	5.246463968	4.832220856	4.837281055	6718	Hddc3	6.308966948	6.585528504	6.319963031	9018	Mup3	5.53830517	0.99993991	3.77835515411318	Rab26os	6.508488167	6.852087012	6.503131537	13618	Susd5	4.159484556	2.793162146	4.151949753	15918	Zfp827	8.172635088	8.081498341	8.124755422
2119	Bckdha	6.26768322	5.761652167	6.373234683	4419	Dusp1	6.387695883	6.491811547	6.838227487	6719	Hdgf	10.51935849	10.55126605	10.43837053	9019	Mup5	1.684284231	0.748190881	4.30896430411319	Rab27a	5.434405596	5.12090135	4.979101725	13619	Susd6	6.952731913	7.433661261	7.013092153	15919	Zfp830	6.939987873	7.169468382	6.837403856
2120	Bckdhb	7.078354413	6.504592661	6.774305956	4420	Dusp10	4.171129468	4.656465558	4.06614075	6720	Hdgfl1	1.050288735	2.725833764	0.987303321	9020	Mup7	6.398951841	2.639043205	4.55842606111320	Rab27b	8.057534432	7.813398135	8.306757312	13620	Suv39h1	6.958937889	6.820878875	6.264764546	15920	Zfp831	7.176363234	7.10779775	6.353731041
2121	Bckdk	6.663684036	6.621873816	6.319416718	4421	Dusp11	6.767633509	7.05077611	6.761191774	6721	Hdgfrp2	9.057373767	8.890410259	8.877823414	9021	Mup8	5.363033745	2.4846364	4.56217082411321	Rab28	8.490078526	8.517256864	8.60210192	13621	Suv39h2	4.181081002	5.312118128	4.563770719	15921	Zfp839	5.864329724	6.105830958	6.320590043
2122	Bcl10	6.189997893	6.410596412	6.246708684	4422	Dusp12	5.401017967	5.296936309	5.301896214	6722	Hdgfrp3	10.6060669	10.47968077	10.60112371	9022	Mup9	4.466080112	1.210620627	3.43568748111322	Rab29	5.462790181	4.838710219	5.303828803	13622	Suv420h1	7.694918799	7.664703356	7.793986039	15922	Zfp84	7.807487487	7.658275078	7.511185476
2123	Bcl11a	9.446630876	9.575180537	9.431249216	4423	Dusp13	0.042788042	3.079900514	0.984093729	6723	Hdhd2	7.999719857	7.918683462	8.139943021	9023	Murc	3.531717073	3.554707476	3.1500596711323	Rab2a	10.9299563	10.85035839	10.91735899	13623	Suv420h2	3.535445502	2.611573553	2.978096043	15923	Zfp846	4.781086123	4.743123024	5.26726428
2124	Bcl11b	10.8839789	11.04794455	10.92906799	4424	Dusp14	7.759260119	7.619123427	7.856819488	6724	Hdhd3	4.239710026	4.320440731	3.146973783	9024	Mus81	4.772628328	4.760290596	4.14917592911324	Rab2b	8.405944525	8.195698005	8.002181322	13624	Suz12	9.196691151	9.04208401	9.091109737	15924	Zfp85	3.900879054	4.702467706	4.788552927
2125	Bcl2	7.657066386	7.383202396	7.652626342	4425	Dusp15	5.162713496	4.883580141	5.537151012	6725	Hdlbp	9.4621832	9.459726391	9.5965771	9025	Musk	2.886624869	3.675477051	3.44230237711325	Rab30	4.889522808	5.237929596	4.733782453	13625	Sv2a	11.75343288	11.70969158	11.79817145	15925	Zfp850	4.402223807	4.223694866	4.888974771
2126	Bcl2a1a	1.062752245	0.867299794	1.986256083	4426	Dusp16	5.639004062	6.183772374	6.024701769	6726	Hdx	3.241175817	2.129260694	3.568587142	9026	Mustn1	4.390900494	2.790510223	4.37506026911326	Rab31	8.61978009	8.733229054	8.662999853	13626	Sv2b	11.06178997	10.85913737	10.72315061	15926	Zfp85os	4.725602075	5.208262049	5.191020247
2127	Bcl2a1b	3.066615149	3.033242779	2.983024933	4427	Dusp18	7.905642177	7.895755353	7.526709052	6727	Heatr1	6.737421399	6.372589854	6.336877894	9027	Mut	8.494336282	8.569371852	8.55364779911327	Rab32	4.237292119	3.649958288	2.558937714	13627	Sv2c	2.887271037	1.7835591	3.151343323	15927	Zfp862-ps	4.715631838	5.189899352	4.149552626
2128	Bcl2a1d	2.646530187	2.453447738	2.305261631	4428	Dusp19	5.856936319	5.567738694	5.264514271	6728	Heatr3	6.737203174	6.494496034	6.609424774	9028	Mutyh	3.652445814	3.670827084	2.78633722611328	Rab33a	7.319625061	7.717870922	7.645671798	13628	Svil	6.121144176	5.767245059	6.068217312	15928	Zfp865	3.786932857	4.43583762	3.67835579
2129	Bcl2l1	8.664942125	8.697093925	8.422860989	4429	Dusp2	3.244408999	2.119259262	2.786442545	6729	Heatr5a	5.399527828	4.871039964	5.108868297	9029	Mvb12a	5.435803683	5.516818053	5.06656825611329	Rab33b	7.012983291	7.230124359	7.398121838	13629	Svip	8.455459516	8.308707816	8.568833264	15929	Zfp866	6.780664547	6.616896547	6.761382541
2130	Bcl2l11	4.54843877	5.127182477	5.067723454	4430	Dusp22	6.873121895	6.696255879	6.991904338	6730	Heatr5b	7.919447063	7.743561723	7.844153175	9030	Mvb12b	8.731195328	8.622574249	8.80350779711330	Rab34	5.441671764	5.562434711	6.003367737	13630	Svop	8.653942294	8.696569065	8.728300776	15930	Zfp867	6.184544532	5.742756145	5.651851141
2131	Bcl2l12	2.389231943	2.177655998	2.985397113	4431	Dusp23	6.07552695	5.644064371	5.680409806	6731	Heatr6	6.752666765	6.732888704	6.825711739	9031	Mvd	7.055711146	6.969776756	6.83803487411331	Rab35	7.401814729	7.321869781	7.346377881	13631	Svopl	1.064686683	1.882009045	1.985890378	15931	Zfp868	4.991624817	5.298983676	5.44015966
2132	Bcl2l13	7.894059428	7.947390429	8.073025062	4432	Dusp26	8.709749415	8.661215677	8.694258995	6732	Hebp1	7.308076825	7.274440112	6.945406034	9032	Mvk	6.689913828	6.381231068	6.33796898711332	Rab36	4.786480646	5.303468457	5.266045577	13632	Swap70	5.166798405	4.265875208	5.024097539	15932	Zfp869	7.12269746	7.020433051	7.088989985
2133	Bcl2l2	7.449129773	7.866047082	8.01362922	4433	Dusp27	0.037729192	1.468501611	1.571008807	6733	Hebp2	5.325050036	5.303098813	5.067282466	9033	Mvp	3.094152004	4.678539761	2.97587298211333	Rab37	2.692688218	3.276238208	4.068966336	13633	Swi5	10.47062698	10.4332054	10.30976381	15933	Zfp87	6.709247218	6.618800081	6.774708693
2134	Bcl3	3.386362141	1.36669752	3.152885431	4434	Dusp28	7.279839	7.455547736	7.283867493	6734	Heca	6.203941233	5.588875354	5.787528516	9034	Mx2	0.055329501	1.870177376	1.98451255311334	Rab38	3.243811298	3.011056289	3.30379597	13634	Swsap1	6.250191509	6.298825986	6.423458689	15934	Zfp870	5.889398617	6.303882625	5.933961667
2135	Bcl6	7.611491769	7.217488132	7.149267656	4435	Dusp3	10.0347985	10.23054921	10.04337316	6735	Hectd1	9.523737053	9.620727932	9.738244635	9035	Mxd1	7.420196513	7.304344668	7.48793854511335	Rab39	2.063352615	2.195928945	1.567427998	13635	Swt1	7.337791214	7.053331822	7.372397814	15935	Zfp871	9.55014021	9.533114982	9.673541965
2136	Bcl6b	2.87864597	2.854266887	3.153321899	4436	Dusp4	5.25043695	5.298720718	5.149518977	6736	Hectd2	6.606564506	6.999772974	6.886912157	9036	Mxd3	8.299369533	8.435709857	8.07787189311336	Rab39b	8.057935388	8.051918649	8.324541875	13636	Syap1	7.867740035	7.701654982	7.645195913	15936	Zfp873	2.664467966	2.645551071	3.443168263
2137	Bcl7a	8.680582061	8.76292841	8.916771522	4437	Dusp6	8.683942083	8.196896566	8.515180831	6737	Hectd3	7.056827584	7.029125505	6.886777059	9037	Mxd4	6.975709081	6.945247005	7.15016639111337	Rab3a	11.90431622	11.8556653	11.77682567	13637	Sybu	7.716785929	7.73968394	7.616564363	15937	Zfp874a	5.330324447	5.187396068	5.471917651
2138	Bcl7b	8.046214372	7.818945783	8.184741196	4438	Dusp7	8.950674155	9.154462214	9.021751813	6738	Hecw1	9.265685779	9.149760019	9.699524018	9038	Mxi1	8.429690059	8.718545732	8.79075892511338	Rab3b	7.852805301	7.865082982	7.46385171	13638	Syce1	1.052544609	0.889831111	1.572377071	15938	Zfp874b	6.306988701	5.710731733	5.886216193
2139	Bcl7c	5.286234543	5.402834937	5.067518171	4439	Dusp8	9.402376679	9.55629541	9.457797987	6739	Hecw2	7.071086805	7.202578519	7.775099831	9039	Mxra7	5.7796778	5.61736505	5.70798639811339	Rab3c	11.83954669	11.37767755	11.7214564	13639	Syce1l	0.037709367	1.891570762	1.987006885	15939	Zfp879	4.244295695	4.01096894	4.374354439
2140	Bcl9	7.496553047	7.432979418	7.189368978	4440	Dusp9	1.04071174	0.91553063	1.574992061	6740	Heg1	2.657281951	3.035992548	2.302205872	9040	Mxra8	5.364137976	5.65652118	4.5001245311340	Rab3d	6.032919073	6.220499292	5.734367776	13640	Syce2	5.39122679	5.476255013	3.674900885	15940	Zfp882	6.007729007	5.668985738	5.470908823
2141	Bcl9l	8.697713469	8.656850682	8.319795804	4441	Dut	7.618418671	7.453009362	7.380809199	6741	Helb	5.027595106	4.415758217	4.838966358	9041	Myadm	7.828106864	7.894985071	7.47949428611341	Rab3gap1	9.738777342	9.744730918	9.825508201	13641	Sycp1	1.104390863	4.36047825	0.973137598	15941	Zfp9	6.118478177	5.697998698	6.068877666
2142	Bclaf1	11.21176513	11.10567794	11.25320659	4442	Dvl1	8.016439124	7.843223564	8.019090031	6742	Hells	4.601973251	4.540062243	4.788639166	9042	Myadml2	3.24664063	4.122212948	3.98408422411342	Rab3gap2	8.752714325	8.592245561	8.816148345	13642	Sycp2	3.886720847	4.505064222	4.568155663	15942	Zfp90	6.308592005	6.34790274	6.439949243
2143	Bco2	3.080316395	3.866339308	3.150298296	4443	Dvl2	5.959348346	5.795296108	6.110640538	6743	Helq	4.716156506	4.15588015	4.62548318	9043	Myb	3.970179331	3.308218917	3.56754485611343	Rab3il1	4.534474393	4.549181499	4.066602011	13643	Sycp3	7.589391045	7.883371815	7.748141233	15943	Zfp91	10.27400261	10.16416563	10.2757747
2144	Bcor	6.679202754	6.465539875	6.863341661	4444	Dvl3	1.65929301	2.186414687	2.792931321	6744	Helt	0.021965357	1.493114204	1.573894171	9044	Mybbp1a	7.036053814	7.239193484	6.96822347111344	Rab3ip	7.641906178	7.627241335	7.55750553	13644	Syde1	4.98939242	5.085069585	5.596473468	15944	Zfp91Cntf	10.06463109	9.893290758	9.744823155
2145	Bcorl1	7.616274143	7.11384327	7.293177809	4445	DXBay18	1.628786127	0.904831364	0.988491209	6745	Helz	7.68032607	7.420063834	8.089068528	9045	Mybl1	4.672184242	5.006662894	5.30293270911345	Rab40b	6.539286599	6.52537362	6.372046979	13645	Syde2	2.099596928	4.127495884	3.684058202	15945	Zfp92	4.457645257	3.138784502	3.564734908
2146	Bcr	8.638534312	8.633043803	8.638303973	4446	Dxo	4.889219334	5.340954259	4.886541538	6746	Hemgn	0.032539768	1.476237501	1.571913834	9046	Mybl2	0.021984877	1.49308131	0.98848800111346	Rab40c	7.873904603	7.878798666	7.479536076	13646	Syf2	9.008261449	8.948810473	9.013430064	15946	Zfp93	4.888589294	4.834604359	5.33911431
2147	Bcs1l	6.384916053	6.622696828	6.708555525	4447	Dydc2	2.389230455	3.052464563	0.977394228	6747	Hemk1	5.65966148	6.107292045	4.732592816	9047	Mybpc1	2.404750826	2.649797632	3.56956981411347	Rab42	4.079088357	4.557336367	4.228953546	13647	Syk	2.676189824	3.553947384	2.979108551	15947	Zfp930	5.840973346	6.156969403	6.439827623
2148	Bdh1	8.642677375	8.513189718	8.459641869	4448	Dym	7.066608678	6.970578759	6.694081492	6748	Henmt1	2.864971051	2.18413037	2.305524667	9048	Mybpc3	1.054616731	2.494043824	0.98619050911348	Rab43	5.32002745	4.52885494	4.887291272	13648	Sympk	6.097286354	6.057063092	5.405333297	15948	Zfp931	3.658877705	4.331598467	2.560302911

2149	Bdh2	4.408494943	5.058047102	3.558323879
2150	Bdnf	9.903863832	9.867133425	9.53252236
2151	Bdp1	9.11090992	8.802638452	9.064841459
2152	Bean1	5.439687893	5.449786661	5.888003374
2153	Becn1	9.165160098	9.169154567	9.232740026
2154	Begain	7.986730754	7.791474353	8.035498754
2155	Bend3	3.090817391	3.537905184	3.681127261
2156	Bend4	4.4764881	4.589854475	5.069082081
2157	Bend5	6.897405293	6.241076917	6.787910657
2158	Bend6	10.55652417	10.39457945	11.08135328
2159	Bend7	4.944808125	4.773890824	6.091015676
2160	Best1	3.075532474	2.402792479	3.98495479
2161	Bet1	5.696315624	5.768433632	5.734746854
2162	Bet1l	9.539561372	9.21890707	9.272275378
2163	Bex1	8.607749792	8.503014161	8.728344523
2164	Bex2	10.75289592	10.63058391	10.52877896
2165	Bex4	6.834072181	6.683281223	6.899318287
2166	Bfar	8.481153056	8.345189704	8.363650711
2167	Bfsp1	3.23851673	3.322877616	3.15244109
2168	Bfsp2	5.166528419	4.829521842	5.109401188
2169	Bgn	5.907859479	5.751481809	5.595040386
2170	Bhlha15	2.388359882	2.879178414	3.156062648
2171	Bhlha9	4.709622972	3.836713725	3.980573447
2172	Bhlhb9	8.458764302	8.208798116	8.359551691
2173	Bhlhe22	9.858008646	9.44678706	9.096117908
2174	Bhlhe40	7.385368854	7.070062936	7.893360935
2175	Bhlhe41	7.843627948	8.011472746	8.223223979
2176	Bhmt	1.639964677	0.876081826	1.571013809
2177	Bicc1	5.121420328	5.167795533	5.024853884
2178	Bicd1	8.897415102	8.785826933	8.922548001
2179	Bicd2	7.627013392	7.593156204	7.735131733
2180	Bid	5.208811027	5.711445223	4.885780955
2181	Bik	1.622199973	0.921264841	0.990437412
2182	Bin1	9.85188428	9.911114133	9.687319063
2183	Bin2	4.771757323	4.762064707	3.978625571
2184	Bin3	4.989153916	5.124227526	5.33848385
2185	Birc2	8.10233928	8.124522174	7.968657259
2186	Birc3	2.672746673	3.309191211	3.790967293
2187	Birc5	3.87519447	3.300726866	3.983515684
2188	Birc6	9.70999783	9.631848426	9.897410877
2189	Bivm	6.914177499	6.902356889	7.372982408
2190	Blcap	9.034339946	8.887697598	8.922525882
2191	Blm	4.403596173	4.290665202	5.152668491
2192	Blmh	8.878298734	8.988007487	9.002417949
2193	Blnk	4.34134051	5.272507461	5.596882704
2194	Bloc1s1	9.508843019	9.557539609	9.397802941
2195	Bloc1s2	5.669665588	6.353983238	5.622670603
2196	Bloc1s3	6.172016362	6.723956129	6.851077194
2197	Bloc1s4	7.124467243	7.283921415	6.812364852
2198	Bloc1s5	6.385471452	6.390373524	6.455625676

4449	Dync1h1	11.13981912	10.907802	10.99818205
4450	Dync1i1	9.917893961	9.715822522	9.756426862
4451	Dync1i2	11.39792301	11.27137231	11.20336781
4452	Dync1li1	8.299404523	8.181800465	8.324383833
4453	Dync1li2	10.27544693	10.41152091	10.54131142
4454	Dync2h1	8.511347754	8.683795777	8.670033615
4455	Dync2li1	6.554900773	6.526392576	6.53486081
4456	Dynll1	10.91101409	10.86023895	10.62830057
4457	Dynll2	12.15075228	12.2516524	12.38459495
4458	Dynlrb1	10.85530174	10.71575039	10.8136688
4459	Dynlrb2	6.230148715	7.349244984	5.933335043
4460	Dynlt1a	7.992520856	7.877383451	7.774349832
4461	Dynlt1b	6.126680842	6.157170516	6.406600586
4462	Dynlt1c	7.091494907	7.25860447	7.046057255
4463	Dynlt1f	6.188403643	6.249813349	6.228187196
4464	Dynlt3	11.26470798	11.07620534	11.0433708
4465	Dyrk1a	8.752718413	8.63528138	8.744915168
4466	Dyrk1b	4.251919463	5.071035222	4.889238022
4467	Dyrk2	2.879213139	3.458256131	3.153191815
4468	Dyrk3	5.685901519	5.047223886	4.501035988
4469	Dysf	2.875746109	3.03612364	2.789918173
4470	Dyx1c1	4.937530032	4.882220678	5.190670189
4471	Dzank1	8.805477742	8.620659318	9.134923325
4472	Dzip1	8.777544894	8.465242519	8.991358716
4473	Dzip1l	5.540033423	5.774640944	5.863687936
4474	Dzip3	9.987311262	9.804805108	9.940004107
4475	E030003E18Rik	2.643754562	0.826859581	2.793149667
4476	E030011O05Rik	4.610045966	5.342027839	5.109987503
4477	E030013I19Rik	3.972764737	4.583633231	2.561287181
4478	E030024N20Rik	9.323388812	9.170971471	9.237408893
4479	E130006D01Rik	1.64486345	1.457696228	2.572451491
4480	E130008D07Rik	3.767344995	3.307208218	3.303187059
4481	E130012A19Rik	6.886931962	6.850289221	7.311952227
4482	E130102H24Rik	3.786523051	4.497425999	4.683163224
4483	E130114P18Rik	6.617561615	6.328043696	6.439499908
4484	E130201H02Rik	1.65658455	2.191117951	2.793394962
4485	E130215H24Rik	2.059562462	1.447436511	0.982162155
4486	E130218I03Rik	0.076859536	3.053918351	2.569054425
4487	E130307A14Rik	6.122541745	6.03007122	6.002582447
4488	E130308A19Rik	5.605657864	5.994481757	5.438430371
4489	E130309D02Rik	6.399380434	6.214855969	6.228206452
4490	E130309D14Rik	6.540961818	6.829851148	6.863128808
4491	E130310I04Rik	1.630814094	1.909334608	0.987885659
4492	E130311K13Rik	6.646160519	6.4112188	6.337799793
4493	E130317F20Rik	4.07094171	3.751599296	4.375855545
4494	E230016M11Rik	2.884432205	2.842155672	2.787664632
4495	E230029C05Rik	3.79373908	4.073617983	5.027027583
4496	E2f1	5.539081714	5.568216692	6.090456269
4497	E2f2	2.881849062	3.324015806	2.981898488
4498	E2f3	6.5733593	6.829272429	6.899352307

6749	Hepacam	8.357018147	8.441438647	8.495731172
6750	Hepacam2	1.66561896	2.17543987	1.981218836
6751	Heph	5.610161318	6.086504529	6.067489972
6752	Herc1	10.56342807	10.52063885	10.82865172
6753	Herc2	10.48445658	10.28483878	10.32428222
6754	Herc3	8.029457927	8.22651558	8.511730814
6755	Herc4	7.326880364	7.075715021	7.247020365
6756	Herc6	5.209402079	5.548673141	5.736810855
6757	Herpud1	7.565034643	7.543712126	7.694256939
6758	Herpud2	7.821162961	7.557691452	7.806722733
6759	Hes1	7.390788191	6.923542842	7.035266591
6760	Hes3	0.019275889	0.910133542	1.574435921
6761	Hes5	7.277955754	6.764970593	7.265915396
6762	Hes6	7.019099504	6.88760875	6.50335896
6763	Hexa	7.146685526	7.418864342	7.088391498
6764	Hexb	8.234933805	8.109083268	8.228012517
6765	Hexdc	6.898132518	6.550852219	6.838468929
6766	Hexim1	8.665843739	8.726336719	8.790574047
6767	Hexim2	6.07760939	5.991127959	6.32121901
6768	Hey1	7.571158977	7.641889767	7.265195725
6769	Hey2	3.893623579	4.001030885	4.625853698
6770	Heyl	3.421130712	4.606057502	5.269464713
6771	Hfe	3.671605091	4.439863037	4.374206381
6772	Hfe2	0.05519036	2.200972947	2.794369311
6773	Hfm1	3.651237691	2.122420776	2.786741672
6774	Hgf	4.468150823	3.821486815	4.625642049
6775	Hgh1	5.034924251	5.200090264	4.562756128
6776	Hgs	7.454924262	7.304778821	7.565281389
6777	Hgsnat	6.900095986	7.040915428	6.734669364
6778	Hhat	1.646468034	1.878970614	0.983101976
6779	Hhatl	5.293901584	6.06006915	5.863680643
6780	Hhex	3.405522652	4.025281249	3.788513199
6781	Hhip	6.666543066	6.668833121	6.748108084
6782	Hhipl1	0.011140597	0.927248116	1.991605264
6783	Hiat1	6.877613067	7.112614492	7.406592913
6784	Hiatl1	6.698618263	6.833866279	7.179830815
6785	Hibadh	8.174494787	8.356651734	8.576265269
6786	Hibch	5.723514223	5.41588911	5.958223081
6787	Hic1	2.091560095	3.181695436	3.685221287
6788	Hic2	5.57685183	5.789362854	6.109919206
6789	Hid1	8.687382785	8.693683916	8.519126721
6790	Hif1a	8.193630239	7.783731149	8.284022207
6791	Hif1an	7.218805608	7.017235319	6.922244702
6792	Hif3a	3.082569223	3.301874552	3.566834361
6793	Higd1a	9.279980886	9.332375475	9.142359039
6794	Higd1b	3.891427971	3.828163939	3.562228053
6795	Higd2a	9.858587774	9.932211554	9.767865126
6796	Hilpda	6.958771009	7.017019145	6.128762306
6797	Hils1	1.064665318	3.232703186	1.98589441
6798	Hinfp	3.378064678	0.793932348	3.155137453

9049	Mybph	2.390290604	1.841532826	3.15568687811349
9050	Myc	3.652847623	1.330553648	4.15489876911350
9051	Mycbp	6.232813834	6.79822699	6.51929222311351
9052	Mycbp2	11.22267632	11.08735921	11.1936905611352
9053	Mycbpap	3.766863694	4.123670148	3.44147145911353
9054	Mycl	6.537149913	6.425967621	6.30199588511354
9055	Mycn	4.333593565	4.592486197	5.19180572311355
9056	Myct1	1.656607599	1.431394669	1.98324737411356
9057	Myd88	4.544302986	5.379527049	4.5022895411357
9058	Mydgf	6.796187811	7.153707764	6.69366825711358
9059	Myef2	8.245474884	8.268448276	8.34640574711359
9060	Myeov2	11.14047776	11.0146547	10.812841811360
9061	Myg1	7.707375932	7.393446925	7.20862096511361
9062	Myh1	2.868247278	2.447655884	3.44613588211362
9063	Myh10	10.03481117	9.949950639	10.2397798911363
9064	Myh11	5.669091618	5.739849215	6.1902030211364
9065	Myh13	2.377680267	1.447309668	2.30776504611365
9066	Myh14	7.092279037	7.536315065	7.218503211366
9067	Myh15	3.670569291	3.925778072	4.62694970411367
9068	Myh2	2.075660353	3.204133393	3.15533962111368
9069	Myh3	3.540268935	5.012454713	3.56498520611369
9070	Myh4	2.674543847	0.689086783	5.77025850611370
9071	Myh6	3.522759585	2.647108949	3.56932959711371
9072	Myh7	5.279932878	4.747906218	4.98134999211372
9073	Myh7b	6.950649136	6.903675865	7.2093670211373
9074	Myh8	3.389637443	3.31940856	2.98127222411374
9075	Myh9	8.040304868	8.03300112	7.76761572811375
9076	Myl12a	8.141342832	8.146046765	7.92809061711376
9077	Myl12b	9.582336592	9.584466854	9.59839259311377
9078	Myl2	0.050360774	2.206040656	0.98262157911378
9079	Myl4	5.216313165	5.911035618	6.15152035611379
9080	Myl6	11.16869192	11.26629698	11.1284541211380
9081	Myl6b	5.214185055	5.595617239	5.8881970711381
9082	Myl9	5.835799262	6.071605725	5.59378181311382
9083	Mylip	6.350313936	6.993617161	6.74842623411383
9084	Mylk	8.622359343	8.750846693	8.14975870911384
9085	Mylk3	2.090710541	2.848896172	3.68534464811385
9086	Mylpf	3.401676491	3.856004206	4.37666379311386
9087	Mynn	4.949753875	5.889018281	5.65251399511387
9088	Myo10	7.914023319	8.291231052	8.27026498711388
9089	Myo16	3.397096663	3.767909235	3.79038264611389
9090	Myo18a	9.740298233	9.720891913	9.59837222311390
9091	Myo18b	2.631980697	1.888319584	0.98459625911391
9092	Myo19	6.309839907	6.597520676	6.42311454811392
9093	Myo1a	2.055604135	2.480420465	0.98357735611393
9094	Myo1b	6.458209411	6.68263343	7.08927825411394
9095	Myo1c	4.413383957	4.87888715	5.33892421911395
9096	Myo1d	6.056588186	6.448410689	5.70708945611396
9097	Myo1e	5.252548253	5.678756654	4.88479769611397
9098	Myo1f	3.240152669	2.842368368	3.44278105211398

Rab4a	7.034832248	7.027391385	7.129895159	13649
Rab4b	8.074099153	8.082599181	7.780722481	13650
Rab5a	7.828381129	7.872384815	7.812944593	13651
Rab5b	8.463894526	8.189621147	8.292796246	13652
Rab5c	9.375053477	9.335763469	9.249076086	13653
Rab6a	12.28055448	12.19832904	12.29993742	13654
Rab6b	12.13924586	11.99694852	12.26394753	13655
Rab7	9.981292731	10.03544289	9.918104492	13656
Rab7b	5.274496122	4.296919359	3.884715513	13657
Rab8a	8.290093769	8.373582698	8.410534152	13658
Rab8b	6.440679441	6.590779792	7.014085052	13659
Rab9	7.455369458	7.601545739	7.227588663	13660
Rab9b	7.229646417	7.15083518	7.218529134	13661
Rabac1	9.733318022	9.715990252	9.594657796	13662
Rabep1	11.08959515	10.83624462	10.96938137	13663
Rabep2	4.003526902	4.930752945	5.655642203	13664
Rabepk	7.14612715	7.258230455	7.169979292	13665
Rabgap1	9.49175097	9.210166166	9.239676039	13666
Rabgap1l	10.40225113	10.23558377	10.1882156	13667
Rabgef1	7.010510954	7.181688706	7.150368086	13668
Rabggta	6.808887495	6.601573989	7.035636928	13669
Rabggtb	8.406766804	8.180827501	8.227792865	13670
Rabif	6.522718105	6.568074154	6.875797726	13671
Rabl2	5.916223599	5.920133072	6.566075107	13672
Rabl3	5.603382651	5.822117152	5.680864793	13673
Rabl6	9.805005445	9.894861769	9.874846277	13674
Rac1	10.40813951	10.39617983	10.46783419	13675
Rac2	3.980529347	3.936798639	4.152326462	13676
Rac3	3.891799119	4.496297196	4.150242912	13677
Racgap1	4.265097015	5.435761999	5.536567152	13678
Rad1	5.782922059	5.788502059	6.424321505	13679
Rad17	7.208916814	7.056520126	7.119595484	13680
Rad18	3.894627611	4.712708674	4.566511603	13681
Rad21	9.745991464	9.913628221	10.00519878	13682
Rad21l	1.045678596	0.904786495	0.988485907	13683
Rad23a	7.783225631	8.045536248	8.410585509	13684
Rad23b	9.868331759	9.879767805	9.683848081	13685
Rad50	8.437974307	8.403899515	8.427091706	13686
Rad51	2.649241014	2.447407651	0.976935418	13687
Rad51ap1	3.662995011	4.572820986	3.78853973	13688
Rad51ap2	4.659989799	4.65549889	4.736523382	13689
Rad51c	4.405642775	4.93727465	4.735588459	13690
Rad51d	6.307721155	6.412945292	6.088291155	13691
Rad52	5.978876245	5.329120289	5.3723986	13692
Rad54b	1.690290969	3.177243868	2.981319977	13693
Rad54l	5.471313774	5.484972932	5.981999845	13694
Rad54l2	6.555095809	6.437698142	6.826384947	13695
Rad9a	9.116861297	9.332391442	9.147390788	13696
Rad9b	3.059071311	2.874024343	2.568362567	13697
Radil	3.888093964	3.835062812	3.786761816	13698

Syn1	9.833505464	9.829847184	9.643531237	15949
Syn2	10.48081699	10.05108906	9.771068796	15950
Syn3	6.193551348	6.508399117	7.014297518	15951
Syna	4.163101418	4.670856043	3.29844319	15952
Synb	0.008383876	0.933569984	1.576874431	15953
Syncrip	9.669015505	9.463302811	9.47980818	15954
Syndig1	7.070038231	7.186752353	7.364023761	15955
Syndig1l	6.814031847	7.746713833	7.922989228	15956
Syne1	11.06434384	11.05260028	11.22673304	15957
Syne2	6.82259534	7.1080358	6.787125363	15958
Syne3	3.396520251	3.865200135	2.78554405	15959
Syne4	2.065843863	0.830631789	2.570257459	15960
Syngap1	4.936802113	5.170135437	4.980579389	15961
Syngr1	9.939703343	9.895068191	9.634594369	15962
Syngr2	5.720548074	5.825014259	5.565362302	15963
Syngr3	8.198883826	8.09073517	8.256318426	15964
Syngr4	0.019284426	1.917364167	1.574434172	15965
Synj1	12.82114845	12.51151046	12.49567476	15966
Synj2	6.807874493	6.78101334	7.120316886	15967
Synj2bp	8.871516422	8.765401506	8.796965183	15968
Synj2bp-cox16	8.183755416	8.150612688	8.067344169	15969
Synm	8.858307372	8.806824167	8.777667446	15970
Synpo	10.00051848	9.950865977	9.91815951	15971
Synpo2	4.333005755	4.478416027	5.268136225	15972
Synpr	8.791428467	8.896286308	8.463528292	15973
Synrg	9.386188313	9.195385297	9.196732099	15974
Syp	9.742301324	9.691049102	9.678672873	15975
Sypl	8.124257356	8.201729259	7.868595644	15976
Sypl2	3.251690629	3.761584348	2.560434574	15977
Sys1	7.672904905	7.739621648	7.734985732	15978
Syt1	14.0008565	13.9505212	13.99216442	15979
Syt10	5.46474667	5.584310332	4.437080578	15980
Syt11	13.07864908	13.03897348	12.98945359	15981
Syt12	5.078696013	4.126734975	5.682982152	15982
Syt13	9.500479597	9.572876706	9.694327987	15983
Syt14	6.873227165	6.417761415	6.887327265	15984
Syt15	0.057651357	1.867510494	1.98420264	15985
Syt16	7.436366274	7.33160057	7.265520811	15986
Syt17	5.013894384	3.841992605	3.680163967	15987
Syt2	6.742950274	6.454088186	7.728927051	15988
Syt3	8.151275596	8.138321439	7.962943563	15989
Syt4	11.08403706	10.90240577	11.11506832	15990
Syt5	9.732836175	9.54733351	9.489787237	15991
Syt6	5.610840863	6.489700341	6.723027722	15992
Syt7	11.21495704	11.23986267	11.19491551	15993
Syt9	6.103714583	6.628427624	6.695569017	15994
Sytl1	1.638217937	2.223120571	0.985647803	15995
Sytl2	6.42325471	6.79353473	7.247867899	15996
Sytl3	1.050307095	2.232661863	0.987298606	15997
Sytl4	2.664542047	2.846112769	1.976274697	15998

Zfp932	6.379974961	5.786631159	6.130108597
Zfp933	6.664489601	6.698346666	6.851013774
Zfp934	4.076666327	3.634745989	4.303852905
Zfp935	5.333387689	5.722179742	6.068740134
Zfp936	0.011133568	0.927263826	0.991149321
Zfp937	6.837263833	6.787788568	7.109375791
Zfp938	7.17723386	7.037994952	6.957168542
Zfp939	3.97354235	3.764528543	3.789974006
Zfp94	4.253497198	4.484322059	4.681625941
Zfp940	5.431169637	5.049321864	5.190255808
Zfp941	7.520940145	7.075046122	7.302172498
Zfp942	5.909490283	5.748196516	5.735278179
Zfp943	6.913821173	6.956748687	6.94574494
Zfp944	5.665542903	5.953502529	5.263878751
Zfp945	6.208395649	6.085715848	6.18973497
Zfp946	5.860470241	5.443942723	5.470842194
Zfp947	3.766895622	2.82909251	3.790871983
Zfp948	5.293203364	4.991739949	5.405906638
Zfp949	6.833117894	6.579161044	6.652568244
Zfp950	7.919642146	7.562287163	7.868694461
Zfp951	2.416732612	3.308218407	3.567544798
Zfp952	7.113830554	7.214587481	6.956996205
Zfp953	2.877958565	2.655677672	1.560434954
Zfp954	6.585828043	6.209992295	6.2836029
Zfp955a	7.298302674	7.205546434	7.035128336
Zfp955b	8.005981798	8.099604577	8.139859068
Zfp956	6.471506961	6.513637629	6.406414137
Zfp957	0.016597602	0.91556181	0.989761157
Zfp958	5.981815497	4.989898846	5.838897941
Zfp959	5.53199079	4.632085055	5.109690159
Zfp960	4.882292169	4.937649008	5.191948852
Zfp961	5.638452233	5.836786499	5.734403158
Zfp963	4.836337734	4.881942571	5.190637735
Zfp964	0.040234508	2.217159682	1.986631238
Zfp97	5.159442536	4.93499036	4.564611945
Zfpl1	6.419209367	6.440408207	6.639024338
Zfpm1	7.383973728	7.431570849	7.565322188
Zfpm2	7.012466159	6.855527774	7.34663372
Zfr	12.04557784	11.7966486	11.86834234
Zfr2	6.164246406	5.837256107	5.910886039
Zfx	7.202286895	7.671235622	7.825752563
Zfyve1	4.168141801	4.714066819	4.302503903
Zfyve16	5.836768004	5.811944132	6.209600584
Zfyve19	5.245867153	5.273622972	5.109870404
Zfyve21	6.210974029	6.34635846	5.885613345
Zfyve26	4.46636138	3.62412285	4.373482344
Zfyve27	6.616501267	6.000807549	6.02355909
Zfyve28	5.445797097	6.536064718	6.302825257
Zfyve9	4.656485895	4.15984677	4.228266406
Zglp1	1.050315707	1.484359597	1.572866038

2199	Bloc1s6	6.692721415	6.482545885	6.580249684	4499	E2f4	6.099580438	6.012055629	5.887045256	6799	Hint1	11.27891543	11.03860318	11.00717836	9099	Myo1g	0.029888284	1.480312864	1.98822080311399	Rae1	8.091237857	8.119874392	7.968660264	13699	Sytl5	4.339521427	5.240553238	4.934318577
2200	Blvra	7.168660157	7.330692126	7.447914162	4500	E2f5	7.841613804	7.713958932	7.623665431	6800	Hint2	7.538047872	7.610509157	7.012964265	9100	Myo1h	6.794486858	6.756629692	6.8002530511400	Raf1	6.838274014	7.177561734	7.246881077	13700	Syvn1	7.010467343	7.088488817	6.837810462
2201	Blvrb	6.305237657	5.811498644	6.109728663	4501	E2f6	6.723897759	6.355311158	6.774638876	6801	Hint3	4.403091995	4.221858121	5.475740888	9101	Myo3b	3.230052482	2.436340134	2.3030863511401	Rai1	7.115410659	7.519968849	7.389510665	13701	Szrd1	9.113920427	9.102337219	9.157445862
2202	Blzf1	7.642291763	7.502508402	7.638313887	4502	E2f7	1.058751626	2.488440999	1.571023233	6802	Hip1	7.821946999	7.931812468	7.694079461	9102	Myo5a	11.05862182	11.01382515	11.3165162511402	Rai14	6.002584692	5.571755183	5.472261614	13702	Szt2	4.66557563	5.350276098	4.73515631
2203	Bmf	2.658090065	3.034517539	3.153799759	4503	E2f8	2.052751145	0.871543809	2.573045113	6803	Hip1r	7.635324344	7.638434665	8.073268229	9103	Myo5b	7.806453034	7.729547842	6.84922700411403	Rai2	5.210443637	5.229509888	5.227830678	13703	T2	1.649483261	1.447403466	0.982157506
2204	Bmi1	7.868298708	7.643167422	7.886938702	4504	E330009J07Rik	1.640011685	1.468440606	1.986997646	6804	Hipk1	9.539750266	9.422809298	9.557480444	9104	Myo5c	3.5311941	3.769532216	2.78563924611404	Rala	9.906973756	9.912239193	9.738176625	13704	Tab1	5.245763289	5.341324117	4.980093697
2205	Bmp1	4.417077215	5.196038621	5.625462941	4505	E330011O21Rik	1.646447415	2.208753854	0.983108409	6805	Hipk2	8.091255831	8.156797993	7.979984725	9105	Myo6	10.16403302	10.18773421	10.1955294511405	Ralb	6.693872053	6.660016263	6.638405095	13705	Tab2	9.307195757	9.355278013	9.507508255
2206	Bmp2	5.505435372	4.670465256	5.472052589	4506	E330013P04Rik	2.651822617	3.045951305	1.98036926	6806	Hipk3	7.842374036	8.043809018	8.189784358	9106	Myo7a	6.095214308	6.194459774	4.29615625411406	Ralbp1	10.14512018	9.902285494	10.05948633	13706	Tab3	5.916116886	6.12308591	6.338116527
2207	Bmp2k	6.290167828	6.346490551	6.169433628	4507	E330033B04Rik	5.321854355	4.688234828	4.224579397	6807	Hipk4	5.026279912	4.701828859	4.83930692	9107	Myo7b	0.032481976	2.916232888	1.98780898311407	Ralgapa1	9.671040383	9.599342635	9.650658549	13707	Tac1	7.485358469	8.158907125	8.698055934
2208	Bmp3	5.161983097	5.37890015	5.22962321	4508	E330034G19Rik	0.02995503	1.480209141	0.986726161	6808	Hira	7.135954101	6.953820095	7.237530051	9108	Myo9a	10.62542762	10.344284	10.6166312311408	Ralgapa2	6.2716765	6.453839207	6.246388439	13708	Tac2	5.47564194	6.919069461	6.751129212
2209	Bmp4	6.052308614	5.93300251	5.863402366	4509	E430018J23Rik	2.700567109	3.73314229	4.737974511	6809	Hirip3	6.591378954	6.691961669	7.109643839	9109	Myo9b	5.597712592	5.367300585	5.56639826911409	Ralgapb	8.235810546	8.329594585	8.598603708	13709	Tacc1	10.08615623	9.888579882	10.33211062
2210	Bmp5	0.0402124	1.464898049	1.570587951	4510	E430025E21Rik	6.438618996	6.326416337	6.652810319	6810	Hist1h1a	0.019317667	0.910050193	1.574427361	9110	Myoc	6.914411407	6.442312168	7.07811017411410	Ralgds	8.807646417	8.715351486	8.255837876	13710	Tacc2	8.183316821	8.114365065	7.837694321
2211	Bmp7	5.031998828	4.053157612	5.151034654	4511	E4f1	6.074772988	5.506854089	6.047397907	6811	Hist1h1b	2.871434826	2.869308354	2.567729865	9111	Myocd	1.068346358	1.450835169	2.57187696711411	Ralgps1	6.474791396	6.982710407	6.899362291	13711	Tacc3	5.319446968	5.096676671	4.502398381
2212	Bmp8b	0.013874466	0.9212747	0.990438581	4512	E530001F21Rik	1.70201325	3.00475829	3.567352961	6812	Hist1h1c	4.25117881	4.603644621	4.372692752	9112	Myof	5.285153838	5.089100953	5.10978286811412	Ralgps2	8.323873283	8.388796622	8.292804546	13712	Taco1	4.981340089	3.981764154	4.981466803
2213	Bmper	5.171762477	5.25969931	5.439542097	4513	E530011L22Rik	5.959632164	5.505145828	5.911439347	6813	Hist1h1d	2.882512778	3.784550528	1.558554469	9113	Myom1	2.872985809	2.162940501	1.97959842511413	Raly	3.885725976	3.527426934	4.151650357	13713	Tacr1	3.669814699	2.783897433	4.068383686
2214	Bmpr1a	8.919356448	8.920746361	8.73149171	4514	Eaf1	4.782724031	4.788830586	5.408250368	6814	Hist1h1e	7.278553755	7.066770809	7.199417556	9114	Myom3	4.074622694	3.93122143	4.06885677911414	Ralyl	9.040417544	9.000293758	9.007905321	13714	Tacr3	4.072651344	4.178343636	4.442832652
2215	Bmpr1b	6.525009111	6.74592066	6.503128661	4515	Eaf2	2.859572297	2.198265906	2.307167708	6815	Hist1h2ab	3.973884773	3.429573063	1.97151641	9115	Myoz2	1.062738562	2.214287549	2.30903634111415	Ramp1	8.347580149	8.329113215	8.443542539	13715	Tacstd2	0.011139016	0.927251651	1.576211374
2216	Bmpr2	9.926106013	9.8806308	10.12469731	4516	Eapp	8.224275945	7.795724496	7.985570687	6816	Hist1h2ac	3.242253281	3.778070799	2.787075216	9116	Mypn	3.066618434	2.426011341	3.68611796411416	Ramp2	6.900671749	6.813792118	6.922560741	13716	Tada1	8.274172686	7.940373254	7.945679778
2217	Bms1	8.225186267	7.933438097	8.423131272	4517	Ears2	5.662038142	4.952091169	5.981818364	6817	Hist1h2ad	3.878356614	3.855214404	3.43967451	9117	Mypop	7.713584569	7.07475364	7.39822111211417	Ramp3	6.577298293	7.449410111	7.265625073	13717	Tada2a	7.716657859	7.658357945	7.875141298
2218	Bmx	4.156668474	2.981419706	2.558713551	4518	Ebag9	7.393016725	7.175571581	7.380947366	6818	Hist1h2ae	4.656983698	4.368035908	4.302474584	9118	Myrf	7.514480129	7.539894021	7.47194259811418	Ran	10.09580184	9.976115467	9.918075831	13718	Tada2b	8.255360301	8.318332295	8.251474524
2219	Bmyc	7.62839938	8.054641569	7.916778334	4519	Ebf1	5.678095564	6.386010557	7.150789346	6819	Hist1h2ag	4.066222586	3.547975056	3.440150831	9119	Myrip	7.648894643	7.453077084	7.46388755811419	Ranbp1	8.214091189	8.095890547	8.062183088	13719	Tada3	7.101315355	6.848108484	6.945851965
2220	Bnc2	2.691738274	2.793026423	4.684508821	4520	Ebf2	3.786857383	3.514335283	5.306681041	6820	Hist1h2ah	3.077092486	3.312332391	3.683958246	9120	Mysm1	8.224247005	8.071971748	7.92239209711420	Ranbp10	6.267126372	6.048571826	6.067672117	13720	Taf1	10.09437371	9.753159165	10.0393612
2221	Bnip1	5.472290628	5.753256936	5.5349358	4521	Ebf3	4.355107421	4.710836776	6.424903313	6821	Hist1h2ai	3.398041581	2.999321112	2.785073722	9121	Myt1	3.897078126	4.295827788	4.56600137711421	Ranbp17	3.403400238	3.652935236	4.305691132	13721	Taf10	9.191289707	9.169404116	8.931205698
2222	Bnip2	8.424671415	8.396392853	8.546192493	4522	Ebf4	2.895414665	3.951351367	4.071224389	6822	Hist1h2ak	2.39216095	2.872465472	0.976149212	9122	Myt1l	11.47181568	11.41801541	11.579908811422	Ranbp2	9.102770255	8.856487618	9.277041616	13722	Taf11	7.242361441	7.552341502	7.659657067
2223	Bnip3	9.071934389	9.032673006	9.040697193	4523	Ebi3	2.400685292	3.332419488	2.789555775	6823	Hist1h2an	2.863233329	1.428285505	1.982937664	9123	Mzb1	1.040716832	0.91551964	0.9897561611423	Ranbp3	7.221713415	7.692351756	7.53469961	13723	Taf12	6.707132784	6.311130962	6.188787275
2224	Bnip3l	10.03320507	9.914509719	10.01066799	4524	Ebna1bp2	7.731702366	7.55684414	7.701020579	6824	Hist1h2ao	7.771645539	7.588476128	7.346241404	9124	Mzf1	3.647905151	3.320715156	2.98144983211424	Ranbp3l	5.66527411	5.144607295	6.047331132	13724	Taf13	7.464066888	7.40734971	7.246459396
2225	Boc	5.749859905	5.071657699	5.565080045	4525	Ebp	7.913702243	8.086864211	7.747898481	6825	Hist1h2ap	8.744673076	8.553125374	8.306324362	9125	Mzt1	7.724703522	7.894294029	7.86884870311425	Ranbp6	8.16786933	8.080554381	8.311087094	13725	Taf15	6.440214633	6.631948459	6.899404819
2226	Bod1	8.941485193	8.85727644	9.016247339	4526	Ebpl	7.464721279	7.579798034	7.572496186	6826	Hist1h2bc	8.966079852	8.946584294	8.741422018	9126	Mzt2	7.298801675	7.168683864	7.09878894911426	Ranbp9	7.41082714	7.408873131	7.480098356	13726	Taf1a	5.776802468	5.539977213	5.503640664
2227	Bod1l	10.82285066	10.63441719	10.81050265	4527	Ecd	8.432634955	8.191113323	8.0566728	6827	Hist1h2be	5.081997426	5.520295551	4.678260203	9127	N4bp1	8.763440558	8.581780992	8.94014186811427	Rangap1	10.3944232	10.24293286	10.32762535	13727	Taf1b	6.472239052	6.172573577	6.487972564
2228	Bok	7.743777303	7.952479768	7.077447144	4528	Ece1	7.240032311	7.298800409	7.328963416	6828	Hist1h2bf	1.038055654	0.921258704	0.990436684	9128	N4bp2	4.785504834	4.87674624	5.22865112911428	Rangrf	5.208123326	5.269097586	5.338436551	13728	Taf1c	4.172069688	4.707110556	5.268874472
2229	Bola1	6.250517763	6.572133536	6.302044808	4529	Ece2	4.840759689	4.730537775	5.566478859	6829	Hist1h2bh	0.01387834	1.507509652	0.990437599	9129	N4bp2l1	7.20729411	6.754383583	7.18022310811429	Rap1a	8.52377939	8.592652264	8.519073791	13729	Taf1d	8.27387335	7.736494905	8.124830823
2230	Bola2	8.669350491	8.75407544	8.907922745	4530	Ecel1	4.76563198	3.125151817	4.151220443	6830	Hist1h2bj	0.019309585	0.910066313	0.989110293	9130	N4bp2l2	9.151999358	9.279304815	9.18946601711430	Rap1b	7.93167599	7.700873399	7.850400331	13730	Taf2	6.52543534	6.907019034	6.609077564
2231	Bola3	8.424682274	8.434413537	8.418813511	4531	Ech1	7.523875743	8.032300273	7.447383198	6831	Hist1h2bp	1.048032553	2.236075482	0.9878824	9131	N4bp3	4.667400941	5.015584985	5.11063692711431	Rap1gap	7.672975583	7.47224173	7.754895148	13731	Taf3	8.929102043	9.020846569	8.937112235
2232	Bop1	7.057001786	7.125818651	7.078260092	4532	Echdc1	9.737078971	9.819474169	9.825487329	6832	Hist1h2bq	1.050299657	0.894736477	0.987300516	9132	N6amt1	8.701378581	8.242055061	8.21345765111432	Rap1gap2	8.916325377	8.949674221	8.988531395	13732	Taf4a	4.791183119	5.294949658	5.337409617
2233	Bpgm	8.239599251	8.142359412	8.046063729	4533	Echdc2	4.669562893	4.739413737	5.150944866	6833	Hist1h2br	1.649542375	2.203363192	1.56853703	9133	N6amt2	7.365822354	7.476095212	7.17936917411433	Rap1gds1	9.921504989	10.11414453	10.03797657	13733	Taf4b	5.719319184	5.653138844	5.439697223
2234	Bphl	6.67971469	6.747327672	6.245850925	4534	Echdc3	2.399087809	3.890437821	0.973141351	6834	Hist1h3c	0.019292277	1.497694279	1.574432563	9134	Naa10	7.419378924	7.484422303	7.14969319911434	Rap2a	9.381645889	9.127525469	9.186991943	13734	Taf5	8.687697669	8.721034869	8.587281138
2235	Bpnt1	8.011066725	7.940901926	8.056909374	4535	Echs1	7.886948192	7.695647311	7.564740041	6835	Hist1h4c	1.656636445	0.830755605	0.979886707	9135	Naa15	8.975486695	8.982322267	8.85946516611435	Rap2b	9.638678048	9.545428366	9.50159601	13735	Taf5l	6.249124033	6.17868728	6.302385779
2236	Bptf	10.28014396	10.08428378	10.23265828	4536	Eci1	7.693919592	7.594119876	7.283453508	6836	Hist1h4d	3.772647263	2.609663111	4.685872078	9136	Naa16	5.883657885	5.537316591	5.47148211111436	Rap2c	7.44750463	7.722461774	7.666663645	13736	Taf6	6.081506927	6.503134412	5.956653946
2237	Braf	10.42703743	10.4294273	10.4638193	4537	Eci2	7.001135813	7.695481309	7.169715694	6837	Hist1h4f	1.045660057	0.904826698	2.312003138	9137	Naa20	8.304759657	8.423295543	8.53473932311437	Rapgef1	9.898956894	9.818080138	9.888418586	13737	Taf6l	4.877627112	4.990021012	4.62535682
2238	Brap	7.400951103	7.30708536	7.265528202	4538	Eci3	3.656973572	2.997381399	3.149485795	6838	Hist1h4h	4.411398245	5.168966569	4.063331561	9138	Naa25	7.63549017	7.702102162	7.75485557911438	Rapgef2	8.959914655	8.820506757	9.048876867	13738	Taf7	7.187938416	7.007231374	6.886564082
2239	Brat1	4.541056692	4.979449071	4.788153049	4539	Ecm1	3.543217547	4.100614185	3.438028535	6839	Hist1h4n	3.23690928	2.649594786	2.788640463	9139	Naa30	7.913286186	7.898691131	7.83791108611439	Rapgef3	6.308690668	6.062460333	6.456268378	13739	Taf7l	0.037744599	1.891525497	1.987001594
2240	Brca1	3.237928431	3.572949028	2.300351952	4540	Ecm2	5.882760751	6.282663824	5.065704529	6840	Hist2h2aa1	7.12509957	7.38804332	6.637142583	9140	Naa35	9.219693673	8.958906727	8.98275641511440	Rapgef4	10.12903286	10.37564097	10.24798204	13740	Taf8	4.723004062	5.283648212	4.564577333
2241	Brca2	5.670542171	5.878193627	6.247354243	4541	Ecscr	3.392992339	3.562082132	2.562968667	6841	Hist2h2aa2	7.12509957	7.38804332	6.637142583	9141	Naa38	9.309353237	9.464202283	9.22787496211441	Rapgef5	6.502518311	5.434519175	6.488787507	13741	Taf9	8.506271711	8.518425214	8.16961983
2242	Brcc3	8.114097747	8.123094776	8.237476598	4542	Ecsit	7.514968707	7.559865465	7.495708147	6842	Hist2h2ac	1.132781324	3.166427127	4.445361286	9142	Naa40	4.78718111	5.233039819	5.33830548311442	Rapgef6	8.463928109	8.434439453	8.406278134	13742	Taf9b	7.375514617	7.336547916	7.602309712
2243	Brd1	8.548132201	8.579565926	8.627518019	4543	Ect2	1.066527807	2.478034792	1.985543501	6843	Hist2h2bb	0.081383805	2.175377327	2.791847239	9143	Naa50	8.433812441	8.332765558	8.49965486111443	Rapgefl1	9.605723403	9.584937594	9.493746754	13743	Tagap	6.122670784	5.583375592	6.150540771
2244	Brd2	10.46640621	10.43024934	10.56591512	4544	Eda	2.914938131	3.831689456	4.567531666	6844	Hist2h2be	6.078251215	5.947625699	5.147106322	9144	Naa60	8.884623283	8.77989713	8.85956740111444	Raph1	8.691397979	8.748414534	8.835007782	13744	Tagap1	7.463779649	7.312548659	7.565220908
2245	Brd3	9.472459853	9.289088211	9.335366841	4545	Eda2r	1.641662419	1.888290877	1.570575563	6845	Hist3h2a	3.07040269	1.364098654	3.152724983	9145	Naaa	6.310479004	6.596289332	6.14919048411445	Rapsn	3.073903513	2.407060372	3.568687874	13745	Tagln	5.668050883	5.766360078	5.9109093
2246	Brd4	9.753995328	9.503695987	9.223083983	4546	Edaradd	2.056919175	2.478021392	1.569339558	6846	Hist3h2ba	1.073811791	2.198281306	0.981216703	9146	Nab1	8.338014978	8.438347846	8.43126787111446	Rara	5.537805091	6.003224403	4.677307576	13746	Tagln2	6.725282187	6.969536103	6.850414722
2247	Brd7	8.485089587	8.434805955	8.199025794	4547	Edc3	6.888002579	6.600854582	6.761049804	6847	Hivep1	7.868118112	7.744107773	8.150329624	9147	Nab2	7.36633614	7.608240805	7.48788422311447	Rarb	5.180506251	6.270116521	6.456976112	13747	Tagln3	11.38092797	11.37841413	11.32759575
2248	Brd8	8.455568189	8.538897932	8.414583901	4548	Edc4	6.368111065	6.819484693	6.609427657	6848	Hivep2	10.82277384	10.80881316	10.75642776	9148	Nabp1	5.630534916	5.852062933	4.78507256411448	Rarg	4.986677715	5.406181017	5.190420221	13748	Tal1	4.852383584	4.491113371	6.441047772

15999	Zgpat	4.984502452	5.207609964	4.787228051
16000	Zgrf1	4.976329788	4.94570295	4.149068931
16001	Zhx1	9.496339822	9.501338158	9.43732911
16002	Zhx2	5.366440774	5.826434097	5.624639644
16003	Zhx3	6.083472479	6.499377848	6.439695986
16004	Zic1	7.149431072	7.929263673	7.794101615
16005	Zic2	7.134897043	7.067419629	7.109448129
16006	Zic3	5.781976008	5.716880107	6.321185073
16007	Zic4	4.896535898	6.141650235	6.069599426
16008	Zik1	6.505839304	6.208562287	6.667109155
16009	Zim1	3.513200251	0.797417506	0.976129015
16010	Zim3	7.103250636	6.878147812	7.389804558
16011	Zkscan1	8.592315018	8.466355252	8.707998317
16012	Zkscan14	4.833602167	4.694486719	4.502499539
16013	Zkscan16	5.032202676	5.011884694	5.150985188
16014	Zkscan17	4.40685179	4.935114739	4.502940769
16015	Zkscan2	2.676710002	3.767074203	2.978991529
16016	Zkscan3	6.650698348	6.631347546	6.623709003
16017	Zkscan4	4.171557619	4.425413978	4.565915347
16018	Zkscan5	5.167915753	5.761093605	5.625501942
16019	Zkscan6	5.886563276	5.718716611	5.623290577
16020	Zkscan7	2.063383858	1.437607681	3.157481849
16021	Zkscan8	6.604260347	6.564735406	6.980777121
16022	Zmat1	7.828376355	7.720057752	7.623653678
16023	Zmat2	11.48880397	11.32264018	11.30021663
16024	Zmat3	8.805061664	8.823801461	9.046243417
16025	Zmat4	7.975739957	7.940115492	8.174878855
16026	Zmat5	7.198298153	7.017330801	6.874460896
16027	Zmiz1	8.897323222	8.688816963	9.021781493
16028	Zmiz2	8.702438002	8.827573923	8.797082942
16029	Zmpste24	8.068827629	7.766492222	7.991223102
16030	Zmym1	6.753709549	6.705989634	6.946004244
16031	Zmym2	9.083021771	8.997941229	8.994034008
16032	Zmym3	8.759680707	8.664477374	8.676975281
16033	Zmym4	8.544223057	8.599481863	8.793932813
16034	Zmym5	9.146740438	9.25811257	9.029741959
16035	Zmym6	5.576194569	5.766541357	6.110072834
16036	Zmynd10	3.776637472	4.513959625	3.78844239
16037	Zmynd11	10.87073673	10.6786202	10.55738833
16038	Zmynd12	2.055545311	0.863057427	0.983598228
16039	Zmynd19	4.716250754	4.489828815	4.302115084
16040	Zmynd8	9.745787453	9.612390499	9.683889569
16041	Znf512b	7.112672424	7.125877997	7.00245531
16042	Znfx1	7.999700138	8.137726991	8.246977087
16043	Znhit1	7.900986081	8.15708934	7.837827883
16044	Znhit2	6.848605182	6.83790226	6.721205519
16045	Znhit3	6.950796566	7.029102777	7.078299704
16046	Znhit6	6.938479958	6.978111192	7.024566222
16047	Znrd1	7.42703606	7.160382838	7.045939413
16048	Znrd1as	6.344272221	6.31882435	5.956898261

2249	Brd9	9.483285262	9.388748975	9.433300325
2250	Brdt	6.651139832	6.981590661	6.609067201
2251	Bre	4.932603408	4.846384281	4.064804244
2252	Brf1	6.752637407	6.960867012	6.761447843
2253	Brf2	5.72405757	6.376912939	6.151296404
2254	Bri3	9.32826334	9.361948706	9.227912068
2255	Bri3bp	9.468296438	9.30122053	9.383022231
2256	Bricd5	2.663186706	2.848742298	3.152715954
2257	Brinp1	10.37167073	10.50443581	10.34849998
2258	Brinp2	8.971972472	8.972954045	8.78084097
2259	Brinp3	7.589055776	7.048789865	8.266067107
2260	Brip1	1.085341699	2.682736897	1.982059264
2261	Brip1os	6.011041582	5.662272161	6.390493247
2262	Brix1	7.318972296	7.416494862	7.108893146
2263	Brk1	9.927441438	10.0442726	9.822338812
2264	Brms1	5.080110073	5.552564302	5.266210643
2265	Brms1l	7.482623838	7.708528221	7.787490395
2266	Brox	7.702349102	7.515704019	7.781055597
2267	Brpf1	7.158182687	7.083160423	7.503937114
2268	Brpf3	6.208879157	6.249900685	6.423733716
2269	Brsk1	9.766894051	9.773326803	9.871850323
2270	Brsk2	10.1519515	10.14057585	10.25382894
2271	Brwd1	9.416004676	9.128886303	9.485817709
2272	Brwd3	7.373740607	7.418528323	7.09878583
2273	Bscl2	10.56916607	10.39731428	10.28944604
2274	Bsdc1	6.351211107	7.02252271	6.980516366
2275	Bsg	9.810075413	9.924499637	9.420714249
2276	Bsn	9.318230523	9.222481519	9.256251339
2277	Bspry	2.651738758	2.671943431	0.975770036
2278	Bst1	0.008383876	0.933569984	1.576874431
2279	Bst2	3.255466984	3.942263439	3.148049249
2280	Btaf1	7.773789013	7.786812092	7.887085277
2281	Btbd1	9.455515696	9.435966819	9.471866505
2282	Btbd10	8.205241094	8.392798891	8.649146322
2283	Btbd11	6.939826745	6.84511551	7.431782936
2284	Btbd16	2.890539918	3.558549152	3.150593317
2285	Btbd17	7.179396055	7.043814218	7.25605167
2286	Btbd19	4.531142463	4.163987329	4.303099936
2287	Btbd2	7.840058563	7.496058687	7.526929457
2288	Btbd3	10.4027412	10.64192702	10.76136608
2289	Btbd6	7.471857906	7.401094507	7.159610087
2290	Btbd7	5.668242519	5.990570445	5.335627464
2291	Btbd8	2.884929753	2.40718918	1.975436084
2292	Btbd9	8.812022843	8.898732134	8.988500986
2293	Btc	2.874412922	2.159137031	2.79026356
2294	Btd	4.665338213	4.977522406	4.735214387
2295	Btf3	8.494127877	8.463441922	8.319690291
2296	Btf3l4	8.147001569	8.314235344	8.468058622
2297	Btg1	7.061410527	7.76470273	7.389085678
2298	Btg1-ps2	0.037639987	3.238887945	1.987017308

4549	Edem1	5.212469131	5.152809661	6.026487613
4550	Edem2	5.573156211	5.564035969	5.471286613
4551	Edem3	7.82157253	7.514830354	7.856656967
4552	Edf1	10.21738017	10.21861392	10.01200709
4553	Edil3	8.67702287	8.698539126	8.91378415
4554	Edn1	4.601556313	3.81055089	4.439302656
4555	Edn3	4.092147737	4.213155791	5.025909491
4556	Ednra	4.834462945	4.74338505	4.146757576
4557	Ednrb	7.481843756	7.421297415	7.503482956
4558	Edrf1	6.490516729	6.579925901	6.65266482
4559	Eea1	10.05771944	9.872358683	10.08331059
4560	Eed	6.3856676	6.466291023	6.503526952
4561	Eef1a1	11.89953569	11.88495671	11.67953685
4562	Eef1a2	11.08481433	11.21514109	11.21061827
4563	Eef1b2	10.66361288	10.57498139	10.46879193
4564	Eef1d	8.649840392	8.593428371	8.43937471
4565	Eef1e1	6.695121653	6.50702647	7.120458622
4566	Eef1g	9.531813856	9.572605834	9.631071203
4567	Eef2	11.9953492	11.99037499	11.95582252
4568	Eef2k	6.553388884	6.229023062	6.33769112
4569	Eef2kmt	7.688053709	7.927431116	7.75482418
4570	Eefsec	5.398578255	4.827092494	5.504863079
4571	Eepd1	6.949026656	6.851020943	6.94622159
4572	Efcab1	4.938188972	5.204181362	4.734063516
4573	Efcab10	4.664216802	5.319371665	4.226120778
4574	Efcab12	4.075044918	4.825709567	3.563719976
4575	Efcab14	8.805175627	8.904273011	8.81601904
4576	Efcab2	5.720119543	5.188346125	5.472031226
4577	Efcab5	5.633720383	5.957077597	5.534572018
4578	Efcab6	3.251624983	4.332305458	3.440079977
4579	Efcab7	3.659731231	3.424457839	3.148670298
4580	Efcab9	1.668099756	3.345349974	2.791432554
4581	Efcc1	5.830389394	5.186913555	5.439319683
4582	Efemp1	5.169700113	5.920906781	5.504387641
4583	Efemp2	4.243103821	4.672675383	3.679497894
4584	Efhb	1.669172443	3.202726776	1.98042008
4585	Efhc1	2.669478814	4.592521722	2.980618603
4586	Efhc2	4.069317392	3.85187672	3.300962978
4587	Efhd1	6.57260335	6.963152118	6.534434244
4588	Efhd2	9.969442322	10.19679238	10.25383814
4589	Efna1	3.408979427	4.019420364	3.680290371
4590	Efna2	6.190112449	6.263738084	6.5505377
4591	Efna4	1.062767778	1.885118028	1.986253141
4592	Efna5	8.085007971	7.755737496	8.500339576
4593	Efnb1	6.720616171	6.329327615	6.439661248
4594	Efnb2	7.569750146	7.112931819	7.496213175
4595	Efnb3	9.948969328	9.909950043	9.399814881
4596	Efr3a	10.3402747	10.17290962	10.34412795
4597	Efr3b	8.580076689	8.569695538	8.523047559
4598	Efs	4.471123417	5.02864319	4.624928647

6849	Hivep3	8.57117462	8.177063337	8.427236248
6850	Hjurp	6.886433518	6.447385324	7.002948762
6851	Hk1	9.925779884	9.870219823	9.766258982
6852	Hk1os	3.510537392	1.846487415	2.569062013
6853	Hk2	4.722353742	5.101384507	4.438622136
6854	Hk3	6.189485305	6.298841123	6.372703268
6855	Hkdc1	2.891132191	3.668280978	3.890711328
6856	Hlcs	5.28935856	5.264517457	5.789203093
6857	Hlf	10.64778355	10.67272966	11.1943537
6858	Hltf	7.506632656	7.444767258	7.526956932
6859	Hlx	3.051813021	2.193406272	1.983549467
6860	Hmbox1	4.255222751	4.356089749	4.62441229
6861	Hmbs	4.891975632	5.660077952	4.621581771
6862	Hmces	6.896800414	6.63513285	6.422777814
6863	Hmcn1	1.674000697	1.39149665	2.983865283
6864	Hmg20a	8.129902162	7.98726521	8.17491303
6865	Hmg20b	7.068473183	7.058284796	6.991246801
6866	Hmga1	6.695489137	6.28890456	6.85063917
6867	Hmga1-rs1	6.709936722	6.22042373	6.850654364
6868	Hmga2	4.472396771	4.803943196	4.788802651
6869	Hmga2-ps1	2.882990727	2.136051303	2.299990303
6870	Hmgb1	8.572242751	8.572862346	8.435234454
6871	Hmgb1-rs17	3.066690716	2.657172202	3.57022988
6872	Hmgb2	5.503605197	5.8295082	5.504327541
6873	Hmgb3	8.016970515	7.940842545	8.007935412
6874	Hmgb4	1.052545106	1.480189848	0.986723505
6875	Hmgcl	6.863142592	7.096552081	7.265869584
6876	Hmgcll1	6.076209311	5.72001366	5.957968956
6877	Hmgcr	8.773700128	8.827471568	8.666486579
6878	Hmgcs1	10.95554016	10.97356947	11.02916664
6879	Hmgcs2	5.497540792	4.881820707	4.563456749
6880	Hmgn1	9.167348967	8.985402533	8.744737166
6881	Hmgn2	7.438444791	7.847429671	7.389216916
6882	Hmgn3	8.910665749	9.310674064	8.724598263
6883	Hmgn5	9.054029546	8.777674673	8.389094422
6884	Hmgxb3	6.948818216	6.769547191	6.825724897
6885	Hmgxb4	7.393409522	7.07191876	7.64571781
6886	Hmha1	3.398478891	3.662256285	2.56112944
6887	Hmmr	3.876580423	3.548241087	3.789725659
6888	Hmox1	2.671418895	3.671824869	3.56790005
6889	Hmox2	9.64255471	9.547045453	9.283654658
6890	Hn1	8.294731426	8.442832999	8.103944593
6891	Hn1l	3.870542049	3.169306793	3.303793914
6892	Hnf1a	3.383574656	3.190304776	2.789528571
6893	Hnf4a	2.081511986	1.823682562	2.790298014
6894	Hnmt	7.981953458	8.017072487	8.228057733
6895	Hnrnpa0	10.05076992	10.12322002	10.28837224
6896	Hnrnpa1	5.081871829	5.121922796	5.596261589
6897	Hnrnpa2b1	8.029054736	7.950625053	8.07804221
6898	Hnrnpa3	8.727953361	8.615347808	8.945943742

9149	Nabp2	7.081622197	7.48512656	7.337739264
9150	Naca	8.938287577	8.857462187	8.764558537
9151	Nacad	6.677357019	6.469135109	6.66721503
9152	Nacc1	7.956850856	7.920053913	7.916628659
9153	Nacc2	9.596569029	9.714152534	9.858066998
9154	Nadk	7.68641823	7.383898022	7.208400076
9155	Nadk2	7.057075713	6.935403892	7.088754559
9156	Nadsyn1	2.4262982	4.031792283	4.070657482
9157	Nae1	8.162735574	8.217606589	8.199185891
9158	Naf1	6.402885083	6.29424109	6.389243588
9159	Naga	4.59921094	4.855193016	4.736623055
9160	Nagk	7.846687325	7.600563139	7.439323085
9161	Naglu	4.986584315	5.46812387	4.298838707
9162	Nagpa	6.1441976	6.288491749	5.862367367
9163	Naif1	2.884374931	1.352366636	3.89193778
9164	Naip1	0.021967083	2.239662734	0.988492082
9165	Naip2	2.087955924	3.97517055	2.789037688
9166	Naip5	2.857630192	0.85037451	0.982132854
9167	Naip6	0.024644378	0.899706268	0.987886147
9168	Nalcn	8.523622555	8.534650464	8.54983462
9169	Nampt	8.77345821	8.562135359	8.483717078
9170	Nanog	3.241857838	2.637046432	3.151576648
9171	Nanos1	6.901431349	6.764991319	7.088715041
9172	Nanos3	3.385839572	2.651261727	2.788833349
9173	Nanp	6.808019591	6.629963976	7.099631755
9174	Nans	6.160717328	5.69871604	5.336603501
9175	Nap1l1	9.422377126	9.365563861	9.42713287
9176	Nap1l2	8.71097048	8.706213064	9.370312578
9177	Nap1l3	9.089073217	8.822190293	9.37244333
9178	Nap1l4	9.853599162	9.689269316	9.919613203
9179	Nap1l5	11.21382097	10.95559644	11.54272088
9180	Napa	9.258065549	9.496834938	9.179593659
9181	Napb	12.38829765	12.15658343	12.19125839
9182	Napepld	7.091796991	6.954660148	6.874366745
9183	Napg	9.742129341	9.654921633	9.616607121
9184	Naprt	4.529459778	4.376612409	3.436584124
9185	Narf	7.319694068	7.634710256	7.503667886
9186	Narfl	7.938354274	7.859791597	7.862579423
9187	Nars	10.08624098	10.09631889	10.08063239
9188	Nars2	4.781740691	4.638582972	5.190892293
9189	Nasp	7.854739171	8.3351333	7.714655436
9190	Nat1	3.056687395	2.680925678	2.305019287
9191	Nat10	5.748776184	5.511930134	5.814100343
9192	Nat14	8.672815105	8.839193375	8.663020669
9193	Nat2	2.071529415	1.846358152	2.792258248
9194	Nat6	5.576788434	5.989993997	6.002703447
9195	Nat8	3.886269186	3.637967129	4.068692193
9196	Nat8l	9.652665094	9.674876403	10.08989305
9197	Nat9	5.509407381	5.415099287	5.813289957
9198	Natd1	5.327270506	5.682638138	5.889032449

11449	Rarres1	3.230728564	2.161017203	0.974219736
11450	Rarres2	6.795058109	7.337403701	6.734677392
11451	Rars	8.017811306	8.056191778	7.880543079
11452	Rars2	5.370532779	5.476129378	5.680443432
11453	Rasa1	9.691360553	9.570857967	9.587324336
11454	Rasa2	8.028928973	7.987198253	8.372668854
11455	Rasa3	8.009523556	7.543655055	7.899139239
11456	Rasa4	2.101773213	2.831534552	2.786349531
11457	Rasal1	7.478693628	7.515862987	6.786707624
11458	Rasal2	9.572474335	9.615944093	9.639996138
11459	Rasal3	1.062726134	0.867357948	0.984097402
11460	Rasd1	6.193281519	6.759954163	6.301201493
11461	Rasd2	8.00788312	8.742203363	9.099241776
11462	Rasef	0.057628155	3.22341461	0.981250014
11463	Rasgef1a	10.97453385	10.99283614	10.7921486
11464	Rasgef1b	7.001777106	7.491139271	8.019934674
11465	Rasgef1c	3.666023086	3.531309469	4.375346469
11466	Rasgrf1	10.32499806	10.26383038	10.37444349
11467	Rasgrf2	8.987646509	8.78478393	9.402245024
11468	Rasgrp1	10.85780754	10.95172673	10.75968722
11469	Rasgrp2	7.003592083	7.413027082	7.813169314
11470	Rasgrp3	7.177504848	7.076800531	6.665694238
11471	Rasgrp4	8.210098514	8.309806634	8.355107593
11472	Rasip1	6.203801819	5.616390393	5.911338924
11473	Rasl10a	7.428384964	7.354271757	7.119218735
11474	Rasl10b	9.500095028	9.50397302	9.246743443
11475	Rasl11a	2.09252655	3.180337895	2.300118252
11476	Rasl11b	9.094535643	9.116467763	9.059459415
11477	Rasl12	4.321823759	4.66885619	4.229120785
11478	Rasl2-9	1.638226185	2.491169792	1.571462266
11479	Rassf1	5.604350294	5.911284054	5.438759279
11480	Rassf10	4.233024584	3.549371355	3.56646072
11481	Rassf2	8.184024037	8.346568155	8.406429184
11482	Rassf3	7.242754482	7.530571147	8.046673082
11483	Rassf4	5.642663364	5.733071201	6.519955713
11484	Rassf5	7.347054518	7.346155033	7.464061899
11485	Rassf7	3.761712856	1.794625241	3.684855727
11486	Rassf8	5.211633737	5.544455696	5.301826636
11487	Rassf9	3.400897387	3.760933718	2.295369572
11488	Raver1	5.478267897	6.340687668	6.08922963
11489	Raver2	7.426222508	7.198065907	6.454460607
11490	Rb1	7.53036651	7.092052758	7.397966133
11491	Rb1cc1	10.61782869	10.53281833	10.78333288
11492	Rbak	5.601437744	4.994287684	5.653232826
11493	Rbbp4	10.14664255	10.13588199	10.13480142
11494	Rbbp5	7.987981051	8.046817635	8.218516812
11495	Rbbp6	9.772088253	9.669211456	9.776078303
11496	Rbbp7	9.199897121	9.331494993	9.431221968
11497	Rbbp8	5.248868863	5.30181783	5.504856721
11498	Rbbp9	6.836089755	7.088520164	7.209384866

13749	Taldo1	9.431505749	9.663613333	9.319777693
13750	Tamm41	6.537669158	6.690500321	6.472236919
13751	Tanc1	8.485569907	8.499952759	8.208574297
13752	Tanc2	9.757946346	9.624282696	9.819294151
13753	Tango2	7.829737413	8.062149667	8.165015609
13754	Tango6	4.777898091	3.804191239	4.680758374
13755	Tank	7.231123123	7.304894931	7.363718943
13756	Taok1	12.07243821	11.92618566	12.0033867
13757	Taok2	8.695530121	8.83248818	8.84111757
13758	Taok3	10.22109108	10.01654326	9.996792892
13759	Tap1	3.659516769	4.031886001	3.789377574
13760	Tap2	5.576470094	6.129433884	5.91086624
13761	Tapbp	7.731900608	8.030040921	7.81291318
13762	Tapbpl	6.026418707	5.453967437	5.33729101
13763	Tapt1	7.888782126	8.178633183	8.265601771
13764	Tarbp2	4.726689599	4.583029911	5.068319659
13765	Tardbp	8.544451608	8.683511004	8.819317034
13766	Tars	8.25532243	8.079873877	8.260800872
13767	Tars2	6.054043561	5.929538001	5.652050046
13768	Tarsl2	7.346319633	7.131417895	7.480297547
13769	Tas1r3	7.259157343	7.249542788	7.31130904
13770	Tasp1	6.192890138	6.435701923	6.53456145
13771	Tat	0.027248332	1.484466001	0.987310987
13772	Tatdn1	3.264584166	4.093912519	4.506341365
13773	Tatdn2	8.033879198	7.779535398	7.800086724
13774	Tatdn3	5.536073652	5.263710246	5.023358513
13775	Tax1bp1	10.70166209	10.52321135	10.70280134
13776	Tax1bp3	4.471526749	4.359722198	4.372162986
13777	Taz	6.307912737	6.316484656	6.320221419
13778	Tbata	4.660543241	5.423422048	4.301504942
13779	Tbc1d1	5.988180799	6.143801529	6.150211407
13780	Tbc1d10a	5.854112964	5.395296373	5.108524406
13781	Tbc1d10b	8.365507947	8.354293746	8.199097056
13782	Tbc1d12	6.666684756	6.743893879	6.812921891
13783	Tbc1d13	7.135671174	7.308149574	6.874328326
13784	Tbc1d14	7.228222169	6.880885926	7.013506619
13785	Tbc1d15	8.113661178	8.133218098	7.91647262
13786	Tbc1d16	6.648765387	6.832825409	6.354689768
13787	Tbc1d17	7.18793457	7.027447131	7.140045409
13788	Tbc1d19	6.924653263	6.694531239	6.838120771
13789	Tbc1d2	3.08500627	3.949274185	3.149149148
13790	Tbc1d20	6.05452746	5.950154406	6.046852642
13791	Tbc1d22a	7.513560113	7.322276645	7.363840915
13792	Tbc1d22b	5.167798035	5.122146867	4.979260582
13793	Tbc1d23	7.011471833	6.912763703	7.603020434
13794	Tbc1d24	8.497653247	8.497456379	8.418887707
13795	Tbc1d25	6.680106335	6.645229135	6.863120904
13796	Tbc1d2b	5.362633279	4.917621984	5.654207577
13797	Tbc1d30	8.162939722	8.075071028	8.694675904
13798	Tbc1d31	4.332493035	4.701311888	4.438949553

16049	Znrf1	9.063570761	9.100068085	9.230378856
16050	Znrf2	4.475717294	4.698185036	4.438539351
16051	Znrf3	6.105069998	6.211196053	6.624692733
16052	Zp3	1.045649958	1.913287153	1.573894834
16053	Zpbp	1.649509436	2.700933047	0.982149274
16054	Zpr1	7.308476881	7.00511102	7.415009051
16055	Zranb1	8.210109386	8.229808196	8.41891791
16056	Zranb2	10.5047796	10.30902829	10.63105385
16057	Zranb3	5.77568286	5.360216958	5.565543034
16058	Zrsr1	10.07347043	9.952616537	9.982777034
16059	Zrsr2	9.365252695	9.111827657	8.974278876
16060	Zscan12	6.059970205	6.572465237	6.283890298
16061	Zscan18	6.323648705	5.637931238	5.622860872
16062	Zscan2	2.869896832	1.405174497	2.568159362
16063	Zscan20	4.473340285	4.650347116	4.439135465
16064	Zscan21	6.166855364	5.660324436	6.228365899
16065	Zscan22	4.726776645	4.740584093	4.837874893
16066	Zscan25	1.656591469	1.85930718	3.157065265
16067	Zscan26	5.755837216	5.834448644	6.266240365
16068	Zscan29	4.667613236	5.244340107	5.267279284
16069	Zswim1	6.141000356	5.53380821	5.226148251
16070	Zswim3	5.533416749	5.551220951	5.109310641
16071	Zswim4	4.668083249	4.838726812	5.190952404
16072	Zswim5	4.824716258	4.300547501	3.561387761
16073	Zswim6	8.630960705	8.477714414	8.598427196
16074	Zswim7	5.285560422	5.201330408	4.933873285
16075	Zswim8	7.358677614	7.822349173	7.844396906
16076	Zufsp	4.83669023	4.881243375	5.110080171
16077	Zw10	5.911558266	5.641787423	6.110314328
16078	Zwilch	3.978388428	3.850562257	3.888680818
16079	Zwint	11.85781009	11.57528092	11.77398779
16080	Zxda	5.288923108	5.428818913	5.265558876
16081	Zxdb	5.889666167	6.181174036	6.440556259
16082	Zxdc	5.751048392	5.564398468	5.838714192
16083	Zyg11b	10.63807828	10.61905893	10.71554508
16084	Zyx	6.210313536	6.691940699	6.302051047
16085	Zzef1	7.513612954	7.272325691	7.595199399
16086	Zzz3	8.447502361	8.742435693	8.694286732
