# Supplementary material for: Zingiberis Rhizoma Ethanol Extract Attenuates Recognition Memory Deficits and Neuroinflammation in a Mouse Model of Bilateral Common Carotid Artery Stenosis
Source: Molecules. 2026 Oct 12;31(20):3634. doi: 10.3390/molecules31203634 (PMC13707571; doi:10.3390/molecules31203634)
Supplement: Supplementary file 1 [file molecules-31-03634-s001.zip › Supplementary Material S2-2 Log5 transformed and BCAS.pdf]

Filter: 479	Gene symbol	Sham	BCAS control	BCAS+ZRex-300
	Prss56	0	3.230576886	2.965989229
	Tex21	0	3.230576886	2.965989229
	Gm10789	0	3.230576886	2.965989229
	Ampd1	0	3.230576886	2.965989229
	Chil3	0	3.230576886	2.965989229
	Dfnb59	0	3.203381302	2.789045624
	Cst13	0	3.203381302	2.789045624
	C920009B18Rik	0	3.182455897	3.216137214
	Gstt4	0	3.182455897	3.216137214
	Gm11346	0	3.182455897	3.216137214
	Cma1	0	3.182455897	3.216137214
	Cyp2d34	0	3.182455897	3.216137214
	Hoxc9	0	3.182455897	3.216137214
	Gm37013	0	3.182455897	3.216137214
	Pcdha4	0	3.182455897	3.216137214
	Sohlh1	0	3.182455897	3.216137214
	Guca2a	0	3.182455897	3.216137214
	1700012P22Rik	0	3.182455897	3.216137214
	AA792892	0	3.182455897	3.216137214
	Gm5294	0	3.182455897	3.216137214
	Tex37	0	3.182455897	3.216137214
	4930590J08Rik	0	3.182455897	3.216137214
	Adprhl1	0	3.182455897	3.216137214
	Adam24	0	3.182455897	3.216137214
	AY512931	0	3.182455897	3.216137214
	4930461G14Rik	0	3.182455897	3.216137214
	Cacna1f	0	3.182455897	3.216137214
	Fgf8	0	3.162398256	2.652217756
	Aknad1	0	3.162398256	2.652217756
	Ms4a5	0	3.065506213	2.65225009
	Stpg2	0	3.065397763	2.652142561
	Catsperg2	0	3.065397763	2.652142561
	Slfnl1	0	3.065309061	2.940500306
	Pnpla1	0	3.065106218	2.651853493
	Capns2	0	3.051823828	2.789078159
	4921539E11Rik	0	3.051714384	2.788969974
	5730435O14Rik	0	3.051420354	2.788679325
	Prdm12	0	3.051385659	2.78864503
	Zfp345	0	3.051385659	2.78864503
	4930544G11Rik	0	3.004181827	2.294367328
	Ccdc178	0	2.968466032	2.583380552
	1700101O22Rik	0	2.968268519	2.294011109
	Ngp	0	2.955177485	2.446356542
	Hoxaas2	0	2.952956622	2.540722825
	Hoxa3	0	2.952956622	2.540722825
	Hsfy2	0	2.95276038	2.829165132
	Tpo	0	2.95263204	2.54040139
	2810047C21Rik1	0	2.952524052	2.540294449
	Nms	0	2.952469999	2.828878096

Fam71d	0	2.952267873	2.540040756
4930447F24Rik	0	2.952267873	2.540040756
Glrx3	0	2.952233564	2.540006779
Sgcd	0	2.952199252	2.5399728
4931409K22Rik	0	2.943044104	2.364951376
4933402C06Rik	0	2.928259292	2.965913121
Foxl1	0	2.928259292	2.965913121
Trhr2	0	2.928259292	2.965913121
1700001J11Rik	0	2.928259292	2.965913121
Gm5127	0	2.928259292	2.965913121
Ntn4	0	2.928168655	3.253862504
2210407C18Rik	0	2.928168655	3.253862504
Sost	0	2.928168655	3.253862504
Synb	0	2.928168655	3.253862504
Slc15a1	0	2.928168655	3.253862504
Bst1	0	2.928168655	3.253862504
Wee2	0	2.928168655	3.253862504
9430018G01Rik	0	2.928168655	3.253862504
9030624G23Rik	0	2.927961579	2.965620889
1700011E24Rik	0	2.927961579	2.965620889
Nutm1	0	2.927961579	2.965620889
Padi1	0	2.927961579	2.965620889
1600010M07Rik	0	2.927961579	2.965620889
A530032D15Rik	0	2.927926468	2.965586425
Adgre4	0	2.927926468	2.965586425
Tmc2	0	2.927926468	2.965586425
Gm13807	0	2.925517053	2.294220519
Tbx21	0	2.925430721	2.583307322
Tyrp1	0	2.925146567	2.583025155
Krt19	0	2.913292445	2.652282421
2610037D02Rik	0	2.912985238	2.940425211
Wdr64	0	2.912896053	3.085751126
Lypd8	0	2.912896053	3.085751126
Oas1a	0	2.912890394	2.651885845
Hist1h2bh	0	2.912746717	2.651744125
Cnfn	0	2.912657498	2.940102448
Trim80	0	2.873797977	2.654603761
Icos	0	2.873497869	2.799919664
Naip1	0	2.873385452	2.365195648
Aplnr	0	2.873205136	2.365016789
Fkbp6	0	2.872811579	2.36462641
Ube2d2b	0	2.871351838	2.29368627
Iqch	0	2.861082323	2.665159887
Syngr4	0	2.857773416	2.735335974
Ltb	0	2.85775295	2.446494784
Serpinb6c	0	2.857396299	2.880513199
Pyhin1	0	2.857282957	2.446029876
Rnf133	0	2.856637467	2.02909909
Spaca3	0	2.815578157	1.988009533
Pla2g4c	0	2.807720043	2.230462124

Adad1	0	2.803638051	2.172920339
Ssmem1	0	2.803533771	2.172816585
4930544D05Rik	0	2.802970874	2.172256532
Fam71f2	0	2.802870861	2.172157026
Vsx1	0	2.801506629	2.822129108
Cd163l1	0	2.800006457	2.540648078
Lilra5	0	2.799720159	2.974486237
Nxf2	0	2.799387681	2.54003895
Slc22a30	0	2.798986589	2.539644115
Myo7b	0	2.794368494	2.55623588
Ubqln3	0	2.776362092	1.987594057
Rhbdl2	0	2.775733112	2.365405065
Slc25a2	0	2.775572722	2.654246328
Gm2109	0	2.774949139	2.799245042
Btg1-ps2	0	2.76799641	2.464415038
Actl7a	0	2.75327366	2.51722693
Smim23	0	2.75109139	2.556164052
Gm10536	0	2.747712965	2.789110693
Zfp936	0	2.747712965	2.789110693
Tmem253	0	2.747491439	2.788894314
Tmco5b	0	2.747491439	2.788894314
Tacstd2	0	2.747400865	3.077052839
Ccl11	0	2.747310278	3.222305348
Gm5662	0	2.747310278	3.222305348
Hhipl1	0	2.747310278	3.222305348
Olfr239	0	2.747310278	3.222305348
Cylc1	0	2.747304662	2.788711878
Sned1	0	2.747158722	2.788569331
Gm884	0	2.747103221	3.0767625
1700120K04Rik	0	2.747103221	3.0767625
Plscr5	0	2.747068101	3.076728242
Gm11780	0	2.746146813	2.601893968
Iqcf1	0	2.745363766	2.507639318
4933411K16Rik	0	2.737121414	2.027985712
1700008K24Rik	0	2.736851343	2.463661605
Cubn	0	2.736765291	2.82532717
Mc3r	0	2.736620091	2.664700608
Gm6760	0	2.722616502	2.203809187
1700027A15Rik	0	2.707373461	1.847829971
Serpina6	0	2.704739409	2.446952612
Abcg5	0	2.704542847	2.735579914
Oxgr1	0	2.704306921	2.446527668
Hist1h3c	0	2.704035479	2.735082437
A230020J21Rik	0	2.704001078	2.735048707
Neurog1	0	2.70389286	2.7349426
Thegl	0	2.702879612	2.582627759
Snord17	0	2.70279244	2.728216758
Gm5936	0	2.702773148	2.582522935
AI463170	0	2.702739219	2.582489529
Noxred1	0	2.70271855	2.293294286

Tdh	0	2.70259273	2.582345298
Nkpd1	0	2.702578123	2.293155669
A630001G21Rik	0	2.702384383	2.582140163
Rsph14	0	2.696824098	2.410016383
Csl	0	2.696634929	2.555677406
1700013G24Rik	0	2.692218906	1.847859675
Colq	0	2.679117809	2.607976218
Il25	0	2.678618989	2.172181926
Csnka2ip	0	2.67837384	2.171939224
4732456N10Rik	0	2.678058938	2.606932152
BC049762	0	2.660097706	2.516672125
Dusp13	0	2.657072932	1.948172377
Prr22	0	2.656665449	1.947766128
Dnajb7	0	2.652410165	2.3496573
Armc12	0	2.648943368	2.508560627
Dnajb8	0	2.648288028	2.668536808
Asb17	0	2.638640297	2.665149652
Sgol2b	0	2.638494507	2.22992871
Pramel4	0	2.638460723	2.229895394
Adam18	0	2.638242743	2.229680433
Gm6756	0	2.638215073	2.664731889
Snord100	0	2.637963212	2.229404776
Snx31	0	2.637463736	2.228912225
Igbp1b	0	2.626592564	2.410359636
Lpar3	0	2.622094569	1.91330127
Helt	0	2.621507008	2.654244464
Dok2	0	2.621345377	2.799699389
4931406B18Rik	0	2.621231642	2.36497464
Uba1y	0	2.621163037	2.364907354
Gnat2	0	2.621129845	2.799488666
Nlrp1a	0	2.62105525	2.364801639
Mybl2	0	2.620941407	2.364689986
Btn1a1	0	2.620913071	2.79927673
Nme8	0	2.620514818	2.798887368
4930407I10Rik	0	2.615636042	2.276106567
Tmprss4	0	2.607347882	2.652314752
Cks1brt	0	2.607237296	2.652207234
Btla	0	2.607146846	2.940564885
Nlrp1b	0	2.606940007	2.651918195
Bmp8b	0	2.606940007	2.651918195
Gm38437	0	2.606835093	3.085676056
1700049L16Rik	0	2.606744586	3.178812764
Pou5f1	0	2.606718675	2.651703008
1700020A23Rik	0	2.606718675	2.651703008
9630028I04Rik	0	2.606703735	2.940134759
Gzma	0	2.606683566	2.651668874
Fam124b	0	2.606260318	2.939704342
Slc12a1	0	2.606018046	2.65102184
Dnajc5b	0	2.59837745	2.003078133
Ppp1r2-ps9	0	2.579965578	2.026707727

Gm15910	0	2.579483646	2.171762623
Fbxl13	0	2.577502982	2.072131778
Tcp10c	0	2.576740889	2.507116035
Man2c1os	0	2.576597372	2.071236242
Scin	0	2.57630214	2.360779666
Spx	0	2.561809615	2.516092885
Dpy19l2	0	2.551630095	2.169598592
Gm30505	0	2.548748791	2.583119061
Pnky	0	2.548459776	2.582836814
Cyp4b1	0	2.548224318	2.29343233
Zfp352	0	2.548082734	2.728143499
4833417C18Rik	0	2.547975369	2.728038745
D030045P18Rik	0	2.547160684	2.581568172
Tectb	0	2.528036802	2.080001476
Tekt2	0	2.527453666	2.12052473
Gbp11	0	2.527374084	2.649415414
Gm7244	0	2.526822633	2.119903587
4933406C10Rik	0	2.526579311	2.555186177
Grhl3	0	2.524327122	1.949250364
Cst8	0	2.523607406	1.89498821
Rasef	0	2.500366885	1.761370231
Mtcp1	0	2.496876296	2.319494207
Hypm	0	2.493720699	1.875562174
Cst12	0	2.492279928	2.540680217
2610307P16Rik	0	2.492099242	2.974624965
4930474M22Rik	0	2.492099242	2.974624965
Cpz	0	2.492058303	2.540465691
4930455G09Rik	0	2.49198878	2.974518309
1700026J14Rik	0	2.491947833	2.540358761
Ccl24	0	2.491787906	3.067585324
Smok4a	0	2.491761931	2.540178817
Cngb3	0	2.491761931	2.540178817
Ccl21b	0	2.491746946	2.828801628
Zfp957	0	2.491726837	2.540144847
Gm15708	0	2.491703286	1.987330505
Scp2d1	0	2.491697501	3.134508729
Gramd2	0	2.491685766	2.540105093
Snora19	0	2.491545997	2.974090792
A4galt	0	2.491462638	1.987092787
Idi2	0	2.491449688	2.82851443
Lyg2	0	2.491414576	2.828480507
Pdxk-ps	0	2.491167292	2.539603243
Zfp964	0	2.491091562	2.422877315
Onecut3	0	2.491006535	2.82808628
4933417O13Rik	0	2.490971399	2.828052334
Nyx	0	2.489665893	1.817343492
Foxs1	0	2.489551538	2.275352764
Cmtm2a	0	2.489185606	2.347659918
Tat	0	2.48398404	2.230587923
Nod2	0	2.483589438	2.230202219

Pax9	0	2.483343321	2.229961655
9630013K17Rik	0	2.482556108	2.229192214
Snora75	0	2.48248945	2.664207776
Tssk4	0	2.482468491	2.518452451
Klhl31	0	2.482401824	2.757514587
Ap1m2	0	2.478189948	2.720014935
Vmn1r32	0	2.477724014	2.361429055
1110028F11Rik	0	2.477322783	2.071201333
Egf	0	2.477282988	2.071162197
1700017N19Rik	0	2.47610941	2.134524285
Katnal2	0	2.475292453	2.37710954
Ins2	0	2.474941483	1.686222821
1700015G11Rik	0	2.464225751	2.217080459
Ggn	0	2.4590982	2.077957045
1700020L24Rik	0	2.453177166	2.315503761
4932413F04Rik	0	2.451736608	2.407695972
Chrnb4	0	2.449946716	2.42650242
1700123L14Rik	0	2.448888113	2.00144008
Acad10	0	2.444689356	1.81613887
A930016O22Rik	0	2.433569924	2.71789745
Slc5a8	0	2.433526098	2.464090678
Far1os	0	2.433328344	2.31794074
Syce1l	0	2.432684292	2.463267549
Col20a1	0	2.432422076	2.317053476
Taf7l	0	2.432089181	2.462685652
Pde6g	0	2.430602404	2.049753886
Piwil1	0	2.429881648	1.933009302
Myo1g	0	2.424785797	2.608071345
Vmn2r46	0	2.424117966	2.860990187
E330034G19Rik	0	2.423356233	2.171376265
1700011I03Rik	0	2.42085315	2.626812926
Rln1	0	2.420811302	1.846699311
Foxf1	0	2.420107089	1.846002002
Phf11d	0	2.415568827	2.202604996
Aldh1a7	0	2.40720547	1.735302927
Arhgap28	0	2.405699776	1.73380514
Gm10509	0	2.405686952	2.265229628
Hes3	0	2.395082337	2.735611784
Ugt2b35	0	2.394840878	2.446558683
Cd22	0	2.39467544	2.735220411
Gm13152	0	2.39419812	2.734761312
Gm10334	0	2.394163922	2.973573877
Otop2	0	2.394067695	2.445813459
6330407A03Rik	0	2.394038511	3.040485657
Cyp4f17	0	2.393997423	2.880118059
Hist1h2bj	0	2.393951228	2.445701206
Clec4a4	0	2.393881026	2.445633544
Hsd3b4	0	2.393790668	2.734369422
Hist1h1a	0	2.393680231	2.734263204
Haglr	0	2.393660079	2.879793846

Gm2022	0	2.393438249	2.445206795
0610039K10Rik	0	2.393105724	2.444886312
C530044C16Rik	0	2.393070578	2.444852439
Ces1d	0	2.391749299	1.817926182
Npc1l1	0	2.390944789	2.276467286
Il7r	0	2.39044643	2.201708661
Trim65	0	2.389542725	2.541459573
Gm5086	0	2.381921237	2.169553768
Gtf2a1l	0	2.371244578	2.556082091
Des	0	2.370641741	1.80290424
BC024386	0	2.37061782	2.409625674
Ly6d	0	2.370338117	2.648653493
Hemgn	0	2.370261637	2.409279767
A530050N04Rik	0	2.370066686	2.554939448
Klhl10	0	2.370000118	1.873502122
Gm31763	0	2.369960629	2.554836567
1700031M16Rik	0	2.366677346	2.011006746
1700018B24Rik	0	2.36042521	1.61714141
Eqtn	0	2.35238791	2.238777417
Prm3	0	2.352212522	2.180087905
Psmb11	0	2.351984529	2.596833096
Pla2r1	0	2.351170392	2.383649955
Vsig8	0	2.348877921	2.240605238
Myl2	0	2.348485781	1.845993238
Rpl39l	0	2.346285916	2.113775948
Snora16a	0	2.336902522	2.35982107
Snora64	0	2.322079124	2.362527691
Zar1	0	2.321142338	2.507523036
Chit1	0	2.321090062	2.87239794
Onecut1	0	2.319255494	2.572165827
Ggt1	0	2.317247508	2.129283426
Spag6l	0	2.315951537	2.349306752
Mnd1-ps	0	2.314051173	2.347454649
Hspb2	0	2.313821351	2.218093811
Sult2a6	0	2.31045025	2.654382685
Ehf	0	2.310098863	2.365045968
Cfhr1	0	2.309973624	2.892868416
Ces2e	0	2.30991248	2.364867067
Fam169b	0	2.309747229	2.653709448
Gm4792	0	2.309697089	2.799274866
Zfp473	0	2.309615868	2.364582366
A930011G23Rik	0	2.309525671	2.653497282
9930111J21Rik1	0	2.308711325	2.652717479
Tssk2	0	2.30671933	1.997460266
L3mbtl1	0	2.306413391	2.024797929
Plekhn1	0	2.290460155	1.663080526
Hfe2	0	2.290157611	2.43847456
E130218I03Rik	0	2.287880242	2.180459156
A730046J19Rik	0	2.278948699	2.474099635
Baat	0	2.275530461	2.027450724

Dusp27	0	2.275057465	2.316982286
Trim7	0	2.274832342	2.462722469
1810024B03Rik	0	2.270112989	2.214724824
Gtse1	0	2.261118665	2.051087357
Pcsk2os2	0	2.259163852	2.21860113
Ccr2	0	2.250747409	2.237260548
Ddx11	0	2.247538113	2.376333854
Slc18a3	0	2.235728559	2.322373095
Naip6	0	2.235264559	2.293358825
D730005E14Rik	0	2.235098192	2.582374919
2310081J21Rik	0	2.235000679	2.93994408
Iqgap3	0	2.23491803	2.332860303
9230102K24Rik	0	2.234808111	2.582098323
Gbx2	0	2.234643051	2.727616308
Gm5177	0	2.234305176	2.29244171
Fam57a	0	2.233979611	2.726984306
Bmp5	0	2.233926208	2.277211128
Snord110	0	2.233517314	2.516358145
Ghsr	0	2.232999005	2.263690543
Tex26	0	2.232496635	2.634177556
2410124H12Rik	0	2.216701852	2.506843044
Tigd3	0	2.21526628	2.238447898
Igsf9b	0	2.213793294	2.293255109
Tdgf1	0	2.213280239	2.079846045
Fam83f	0	2.193214421	1.947615538
Gm14393	0	2.192820041	2.477153754
Elf5	0	2.192181613	2.236916196
Dhx58	0	2.188341175	2.318865573
Mx2	0	2.187398656	2.224268695
Fam229a	0	2.174180262	2.127585299
Nmrk2	0	2.169475246	2.230587553
G630093K05Rik	0	2.169475246	2.230587553
1010001N08Rik	0	2.169090026	2.519563519
Kynu	0	2.16887708	2.758486991
Pah	0	2.168428664	2.518935527
Hs3st6	0	2.168415155	2.664657158
4930592I03Rik	0	2.16841045	2.229573654
3830403N18Rik	0	2.168112893	2.229290333
Dao	0	2.168077868	2.229256984
Ankfn1	0	2.162471164	2.313994798
Slfn8	0	2.161353653	2.312907463
Gm16897	0	2.16114943	2.359790749
Syt15	0	2.160970435	2.198630094
Leap2	0	2.157368602	2.603424175
Gm8787	0	2.156459243	2.509398544
1700065J18Rik	0	2.122464257	2.409554869
Xirp2	0	2.12035295	2.602371046
Tnfsf9	0	2.119075597	2.31269329
Cga	0	2.116628934	1.930583323
Tph1	0	2.11598592	1.617209969

Cyb5d1	0	2.114946487	2.077528833
Gm5803	0	2.10808279	2.172242383
BC028528	0	2.107815464	2.700713847
4931406H21Rik	0	2.107533234	2.607025476
Krt85	0	2.107112678	2.460835119
Ppp1r1c	0	2.107016356	2.171230964
Kcna3	0	2.105436411	1.77266851
Gabrr1	0	2.099492596	2.257780038
Trib3	0	2.098889052	2.382963442
Ptgdr	0	2.096352521	2.126803471
Snord15a	0	2.07775109	2.202083733
4930562F07Rik	0	2.075064075	2.059325174
Lrrc9	0	2.073080546	1.917657675
Pcdhb2	0	2.072535768	2.126088902
Apol10b	0	2.057363888	2.347219925
Gm21119	0	2.056701906	2.570780521
Slain1os	0	2.055835052	2.10583917
Ceacam10	0	2.053276196	2.809513705
Snord49a	0	2.052701592	2.648789087
Hist2h2bb	0	2.041570594	2.19659449
Gm19897	0	2.040960535	2.076808778
Gm14827	0	2.037883291	1.932983358
Slc22a1	0	2.02918154	1.789303791
Rgn	0	2.0275417	2.225056388
Akap4	0	2.025194953	1.75511832
Eya4	0	2.025043452	2.256711072
Gm13031	0	2.022586637	2.178205991
Cyp4x1	0	2.002150931	2.601361537
BC016579	0	2.001891323	2.60111848
Crip3	0	2.000897764	2.360804501
Slc12a8	0	1.99970208	2.411172789
Gm5741	0	1.999507737	1.936927051
Dpt	0	1.99701925	2.201395764
Rmrp	0	1.994090623	1.890677122
A530046M15Rik	0	1.974923755	2.018891115
Zfp169	0	1.974019385	2.207379185
F13b	0	1.954805661	2.317661977
Snord15b	0	1.95453609	2.463367395
Galr1	0	1.945304963	2.435516792
Gm10433	0	1.911699445	2.584191372
Tnfrsf13c	0	1.911312731	2.277113139
A630020A06	0	1.911132044	2.635289579
Susd1	0	1.910497064	2.583075325
Nrg3os	0	1.910477645	2.634682353
6720468P15Rik	0	1.910448369	1.986164848
B4galt7	0	1.910231103	2.422116964
5830415F09Rik	0	1.897414949	2.35570197
A930003A15Rik	0	1.885663036	2.368085153
Cd6	0	1.870942582	2.598070152
1700109K24Rik	0	1.869268623	1.947776449

Ckm	0	1.869160608	2.786765855
Cyp2c66	0	1.831847383	2.510365547
Xkrx	0	1.831500857	2.561692628
Gp9	0	1.829936643	1.890835843
A330102I10Rik	0	1.829559364	2.601412639
Ankrd2	0	1.811790313	2.114285882
Tnnc2	0	1.793861498	2.699743625
Map3k8	0	1.793794192	2.168407558
A330015K06Rik	0	1.772801176	2.327694299
Vill	0	1.772585898	2.302112479
Apom	0	1.761327789	2.284824456
Otc	0	1.727327026	2.253797129
Insl5	0	1.637100587	2.515513054
Gm12216	0	1.55784576	2.195034352
Hsd17b2	0	1.446113425	2.257549616
Zfp607	0	1.422293184	2.195291353
Mup1	0	-1.000033336	-0.146704887
F10	0	-1.000564211	0.026781943
Pcdhb11	0	-1.010451661	-0.125843885
Gm13483	0	-1.01195946	0.16306194
Slco5a1	0	-1.061710445	-0.111412305
Mup3	0	-1.063569216	-0.237598328
H2-Q10	0	-1.073561675	-0.490214307
Serpina3k	0	-1.105025455	-0.121377125
Apoa4	0	-1.165571634	-0.207541277
Saa2	0	-1.354893132	-0.396253957
Mup17	0	-1.424200247	-0.279673262
Serpina1d	0	-1.523724021	-0.31495046
LOC100048884	0	-1.541727991	-0.065625542
Mup19	0	-1.576870683	-0.12655994
