# Supplementary material for: Zingiberis Rhizoma Ethanol Extract Attenuates Recognition Memory Deficits and Neuroinflammation in a Mouse Model of Bilateral Common Carotid Artery Stenosis
Source: Molecules. 2026 Oct 12;31(20):3634. doi: 10.3390/molecules31203634 (PMC13707571; doi:10.3390/molecules31203634)
Supplement: Supplementary file 1 [file molecules-31-03634-s001.zip › Supplementary Material S2-3 BCAS+ZRex-300.pdf]

Filter: 13	Gene symbol	Sham	BCAS control	BCAS+ZRex-300
	Mup1	0	-1.000033336	-0.146704887
	F10	0	-1.000564211	0.026781943
	Pcdhb11	0	-1.010451661	-0.125843885
	Gm13483	0	-1.01195946	0.16306194
	Slco5a1	0	-1.061710445	-0.111412305
	Mup3	0	-1.063569216	-0.237598328
	H2-Q10	0	-1.073561675	-0.490214307
	Serpina3k	0	-1.105025455	-0.121377125
	Apoa4	0	-1.165571634	-0.207541277
	Saa2	0	-1.354893132	-0.396253957
	Mup17	0	-1.424200247	-0.279673262
	Serpina1d	0	-1.523724021	-0.31495046
	Mup19	0	-1.576870683	-0.12655994
